# The Argos-CLS Kalman Filter: Error Structures and State-Space Modelling Relative to Fastloc GPS Data

**DOI:** 10.1371/journal.pone.0124754

**Published:** 2015-04-23

**Authors:** Andrew D. Lowther, Christian Lydersen, Mike A. Fedak, Phil Lovell, Kit M. Kovacs

**Affiliations:** 1 Norwegian Polar Institute, Fram Centre, N-9296, Tromsø, Norway; 2 Sea Mammal Research Unit, Scottish Oceans Institute, University of St Andrews, Fife, Scotland, United Kingdom; Sonoma State University, UNITED STATES

## Abstract

Understanding how an animal utilises its surroundings requires its movements through space to be described accurately. Satellite telemetry is the only means of acquiring movement data for many species however data are prone to varying amounts of spatial error; the recent application of state-space models (SSMs) to the location estimation problem have provided a means to incorporate spatial errors when characterising animal movements. The predominant platform for collecting satellite telemetry data on free-ranging animals, Service Argos, recently provided an alternative Doppler location estimation algorithm that is purported to be more accurate and generate a greater number of locations that its predecessor. We provide a comprehensive assessment of this new estimation process performance on data from free-ranging animals relative to concurrently collected Fastloc GPS data. Additionally, we test the efficacy of three readily-available SSM in predicting the movement of two focal animals. Raw Argos location estimates generated by the new algorithm were greatly improved compared to the old system. Approximately twice as many Argos locations were derived compared to GPS on the devices used. Root Mean Square Errors (RMSE) for each optimal SSM were less than 4.25km with some producing RMSE of less than 2.50km. Differences in the biological plausibility of the tracks between the two focal animals used to investigate the utility of SSM highlights the importance of considering animal behaviour in movement studies. The ability to reprocess Argos data collected since 2008 with the new algorithm should permit questions of animal movement to be revisited at a finer resolution.

## Introduction

The accurate depiction of animal trajectories is central to understanding their ecology, from predicting habitat types critical to their persistence [1] to realistically portraying the degree of individual variability in space-use within a population or species [2]. Technological developments over the past 30 years have led to a number of different electronic tagging options, ranging from simple VHF radio telemetry and light geolocation to satellite telemetry which can involve both tracking and a host of different sensors. Recently, incorporation of Global Positioning System (GPS) technology into animal-borne tags has led to significant advances in the scale and resolution of questions that can now be answered [3]. However, the ability to answer questions is contingent upon the methods used to collect such data and the biology of the animal as well as limitations related to feasible logistics and financial constraints. For large animals in situations where they can be recaptured, the use of archival Fastloc GPS tags are a typical compromise between cost and location accuracy. These instruments provide highly accurate location estimates at very high temporal resolution [4]. Historically though, and for animals for which recapture probabilities are low or non-existent such as whales [5] and green turtles [6], satellite telemetry has been the dominant means of acquiring information on wide-ranging movements.

The Argos System is the most commonly used method for acquiring satellite telemetry data from animals. This system uses the Doppler shift in frequencies between the transmitter (animal telemetry device) and Argos transceiver modules mounted on low-orbiting satellites to make Doppler-based location estimations [7]. Location estimates require a minimum of two received messages during a single satellite pass and until very recently this estimation process was performed by a non-linear Least Squares (LS) technique [8]. The resulting estimates are classified into one of seven location classes (LCs) each of which has a measure of uncertainty associated with it. The errors associated with the highest quality LCs (3,2 and 1) have a 68^th^ percentile error described by Argos as ranging from 0.25 to 1.5 km while the remaining LCs (0, A, B and Z) have no estimated errors. In the last decade reasonable estimates of error associated with the lower quality LCs have been made, using empirical data collected from animal-borne telemetry devices [9,10]. More accurate measures of Argos error have been conducted that capture the issues inherent with collecting telemetry data from wide-ranging animals by ‘double-tagging’ individuals with Fastloc GPS and Argos telemetry devices, and measuring the differences in temporally-matched locations along the animal’s trajectory [9,11,12,13]. In addition, Argos provide estimates of the true error around modelled positions using a 2-dimensional ellipse, derived from the covariance matrix of messages received by the Argos module [8].

Deriving empirical values of error and precision in location estimation have proved critical in addressing the issues surrounding animal track reconstruction. Recent improvements in computing power and advances in statistical techniques have given rise to a suite of modelling techniques that predict the likely path of an animal, which incorporate measures of uncertainty of the location estimates. These state space models (SSM) typically consist of two conditional stochastic models, one that describes the theoretical movement of an animal (process model) and another that describes the actual data collected including some estimate of the uncertainty (error) in the estimation of locations (measurement model). The utility of SSM to the location estimation problem has led to the development of numerous techniques to reconstruct an animals’ path, ranging from Kalman filters that treat time as a continuous variable [14,15] to Bayesian methods that estimate the probability of an animal being in a certain location at discrete time intervals [16,17]. The most common estimates of the error structure surrounding location estimates in these models are currently based on the empirically-derived measurements of error conducted by [10].

In spite of the obvious importance of accurate location estimation to studies of animal habitat use and preference, there are only four studies that have assessed the actual performance of SSMs [9,11,15,18]. Three of these studies examined Argos location estimates calculated using the LS algorithm, with two of them using SSMs that are readily-available to researchers. Implementing a Bayesian SSM [16], [9] estimated a mean distance of 2.2 ±2.4 km separation between modelled and true tracks of hawksbill turtles *Eremtmochelys imbricata*. Error rates from a custom-written Kalman filter applied by [15] to data from two grey seals (*Halichoerus grypus*) demonstrated marked sensitivity to pre-processing of telemetry data at varying speed thresholds. Optimal root-mean-square-errors (RMSE) for each animal (5.90 km and 12.76 km, respectively) demonstrated that ~80% of all locations produced were less than 10 km from the true location. Using the same continuous-time correlated random walk (crawl) model employed in the current study, [19] estimated a mean error of 3.2 km (±0.1 km) to concurrently-collected archival Fastloc GPS locations, stating that 79% of predicted locations were less than 5 km from the true location.

Since 2011, Service Argos has offered the research community the option to receive their telemetry data processed by a different algorithm. This new Square Root Unscented Kalman Filter (SRUKF), implemented in an interacting multiple model algorithm, provides a location estimate for satellite overpass. Service Argos claim that mean spatial errors of poor-quality LC location estimates are reduced by up to 76% using the new SRUKF algorithm and that up to 12.7% more derived location estimates are generated [13,20]. Indeed, the fourth study examining SSM performance used raw Argos data generated using the SRUKF method [18] and described accuracies of 5.6km (±5.6km) from interpolated GPS locations of seven harbour seals (*Phoca vitulina*) and six fin whales (*Balenoptera physalus*).

The ability to re-process Argos Least Squares telemetry data collected after January 2008 using the new SRUKF algorithm provides researchers with the opportunity to revisit questions of movement behaviour at a higher spatial resolution than before, at minimal additional cost. In light of this, and the fact that Service Argos will probably remain the most widely-used means for collecting telemetry data on animals that are unlikely to be recaptured, there is a critical need to assess the performance of SSM techniques on SRUKF-derived Argos data. In this study we use individuals from two coastally-resident arctic marine mammal species from Svalbard, the bearded seal *Erignathus barbatus* and the ringed seal *Pusa hispida* to achieve two interdependent aims 1) to derive error estimates of Argos location estimates generated by the new SRUKF algorithm relative to Fastloc GPS locations and 2) to assess the performance of different SSMs in estimating locations.

## Materials and Methods

Prototype Satellite Relay Data-Loggers (SRDLs, Sea Mammal Research Unit, University of St Andrews, Scotland) were attached to six adult female bearded seals (August 2011 N = 4; August 2012 N = 2) and 10 adult female ringed seals (August 2012 only), in Svalbard Norway. Programming details for telemetry devices are outlined in Supplementary Materials. All research activities including both animal care and field site permitting during this study were approved and carried out under permits from the Norwegian Animal Care Authority (Forsøksdyrutvalget ref. 2010/45416 and 2011/42085) and the Governor of Svalbard (Sysselmannen på Svalbard Ref. 2011/00488-52). In addition to the standard sensors for measuring dive data, each SRDL contained an Argos-linked Fastloc GPS transmitter. All tags were programmed to the same specifications (S1 File), and the default speed parameter was used for the Argos Kalman filtering process (10ms^-1^). We are unaware of any other speed parameter setting being used in the marine mammal research community, and can thus not comment on the effects of varying this threshold with respect to the outcome of the Argos SRUKF process. Capturing and handling of these animals was conducted using the same methods as those outlined in [21]. Argos locations are derived as a by-product of all messages transmitted from the SRDL to the satellite system, while transmitted GPS data consist of a random subset of locations collected by the Fastloc GPS receiver on the animal, resulting in temporally decoupled Argos and GPS positions. Calibration studies of Fastloc GPS data show errors (at the 95^th^ percentile level) between 24.2 m and 140 m for locations estimated by eight and five satellite acquisitions, respectively [22]. We removed all GPS locations with less than five satellite acquisitions, and henceforth assume the remaining GPS locations reflect the true position of the animal [23].

### Kalman-filtered Argos error estimation—accuracy and precision

To facilitate comparison with earlier studies estimating Argos location errors from the LS positioning algorithm, we identified SRUKF-derived Argos locations that were temporally-proximate (i.e. within 5 min) to GPS positions using the methods outlined in [12] and [9]. This subset of data was used to calculate the distance (km) and direction (degrees) of error between each Kalman location and its matched true location using the great circle method. LS derived Argos location estimates exhibit a longitudinal bias [10,15]. Consequently, Rayleighs Tests of mean directionality were used to determine whether the same patterns existed for locations derived by the new Argos method. Errors were further decomposed into their longitudinal and latitudinal estimates for each Argos location class (LC) and we present the overall 68^th^ and 95^th^ percentile values of error (km) as well as those for latitude and longitude for each LC.

In terms of modelling error distributions, lognormal distributions have positive-only values, which in terms of spatial error distributions would suggest a continuous bias in one direction (for example, longitudinal errors were always to the east of the true location). As such, this distribution is appropriate for describing absolute error magnitude (precision) but not errors that have a directional component (accuracy). Conversely, the T distribution is commonly used to model spatial error in the context of location filtering and smoothing models as it is robust to extreme values [9,10,14]. To measure the spatial accuracy of the SRUKF data we estimated the parameters of the T distribution (scale τ and degrees of freedom ʋ) for errors in each LC using maximum likelihood methods and using the resulting estimates to generate the error structures for subsequent modelling.

To assess the precision of errors within each LC, we employed the adjusted quantile method of outlier detection to estimate the proportion of errors that are greater than expected, given the ideal distribution of the data using the R package ‘mvoutlier’ [24]. For each LC, robust estimates of Mahalanobis Distances (rMD) between paired latitudinal and longitudinal error estimates were computed. This metric was chosen because it is a scale-invariant measure of the similarity between two datasets but is very sensitive to the presence of outliers [15] and robust estimates were calculated using the R package ‘robust’. We then computed the empirical cumulative distribution function (ECDF) of pairwise rMD, which we assumed contained outliers. Given that rMD are best described by a X^2^ distribution [25], we constructed a cumulative distribution function (CDF) from 100 simulated datasets generated using the X^2^ distribution. To avoid mistaking extreme values as outliers, we calculate an adaptive threshold using the supremum of differences between the rMD ECDF and X^2^ CDF for each point over the 95^th^ quantile [24]. Thus, samples from the ECDF that have an abnormally large deviation (P<0.001) are declared as outliers.

### Location modelling

To test the efficacy of readily-available location-processing models with the new SRUKF Argos data, we utilised telemetry data from two focal animals (one bearded seal and one ringed seal). These individuals were selected based on the duration of their tracking records and how far they travelled. Pre-processing Argos data to remove the most aberrant location estimates is common practice [15] with several ‘destructive’ (data removal) techniques being available [26,27]. We pre-processed our data with the speed filter (SF) implemented in the R package ‘trip’, which is a forwards-backwards speed averaging algorithm that determines the required velocity to move between consecutive points and removes points which require a speed greater than a predetermined threshold [27]. Raw Argos location data were filtered at four speed thresholds (1.5, 2.5, 15 and 27 ms^-1^) prior to being used as input to the location-processing models to test the sensitivity of model performance to pre-processed data [15]. We fitted three different location filtering models that are available in the open-source statistical framework of ‘R’ [28] to each of the four speed-filtered Argos dataset from the two focal animals. Each model was fitted twice; once with the error structures that are presented by the authors of each of the respective models and once with error structures derived from the current study; henceforth these are referred to as the old and new models, respectively.

#### Model (1) crawl Package

The continuous-time correlated random walk model developed by [14] makes inferences using pre-processed data by considering time as a continuous process, modelling the irregular temporal spacing of location estimates as a series of discrete time samples. The output of this analytical framework is a continuous movement path model from which location predictions can be made at any time. An error model that allows the location estimate to vary with respect to its LC is constructed from a ratio of the 95% percentiles of the latitudinal and longitudinal error of the most accurate LC relative to the remaining LCs as reported in [10]. We then constructed the same error model and used ratios of our derived error estimates as input, fitting the model over each focal track with the original error matrix and then with our estimates. We then generated an estimated Argos location for each GPS location timestamp to facilitate comparison using the same 5-minute rule outlined previously.

#### Model (2) bsam Package

The method of estimating the location of an animal from Argos data developed by [17,29] treats time as a discrete process, predicting spatial locations at regular time intervals from irregularly-collected telemetry data under an empirical Bayesian state space framework. This approach implements a Markov Chain Monte Carlo algorithm to estimate the posterior probability of an animal being at a location, given the estimate of that location and the error associated with it. Briefly, a first-order random correlated walk and the Argos data are used as process and measurement models, respectively. The uncertainty of location estimates *ɛ* is incorporated into the measurement model as a three-parameter T distributed error term *ɛ*_*t*_ *~* t(0, τ_t,_ ʋ_t_) with mean 0, scale parameter τ_t_ and degrees of freedom ʋ_t_ for each component of location error at time t, respectively. In the original code, estimates of τ and ʋ are derived from [10] and are in units of km. We use these estimates, and subsequently constructed our own as outlined above. Prior to modelling, τ must be converted into degrees using appropriate conversions based on the latitude and longitude at which the data were collected. We thereby incorporate the fact that the distance in km of one degree of longitude at 80^0^ N is ~17% of the same measure at the equator (19 km cf. 111 km). Given the large number of Argos location estimates provided per day by the new SRUKF algorithm, we estimated a new location every hour. Models were allowed to ‘burn-in’ for 30,000 samples across four chains after which a further 10,000 samples were taken from the posterior distribution after convergence had been assumed. Temporal autocorrelation between samples was reduced by taking every 10^th^ sample after burn-in, and model convergence was checked from trace plots and by calculating the Gelman-Rubin convergence statistic for each model fit [30].

#### Model (3) tripEstimation package

[31] propose a Bayesian solution that permits the estimation of an animal’s path, as opposed to point estimates of its location at discrete times. In this modelling system, secondary information such as bathymetric data and estimates of travelling speed can be incorporated into the estimate of the path that serve to guide it in a biologically meaningful manner. We employed a digital elevation model land mask against which location proposals were matched [32]. Proposed locations that were on land were rejected and new proposals were generated until they met the criteria of the land mask, similar to the procedures employed by [31]. posterior estimates of locations **y**_*i*_ follow a bivariate (lat/lon) normal distribution centred on the true location **μ**_i_, such that **y**_*i*_ ~ N(**μ**_i_, σ^2^(r_i_)) where the distribution variance σ^2^ is a function of the location error estimate r. However [31] acknowledge that in the case of the Argos location service, a longer-tailed t distribution may be more appropriate. Thus, we input our estimates of positional variance σ^2^(r_i_) for the alternate error model. Posterior estimates were sampled sequentially using a block-updated Metropolis-Hastings algorithm run over three chains of 40,000 iterations, thinned to every 10^th^ estimate to reduce serial autocorrelation. Point estimates representing those that fall along the most likely path were derived by extracting the most likely location and these were used for estimating error magnitudes. We present the model output as composite images of the time spent along the full path estimate [31,33].

### Model location accuracy

For model (1) SSM-estimated locations for each model fit were available at the time of GPS locations. For outputs from the other two models we repeated the process used to construct Argos error structures, isolating SSM locations that were temporally coincident with the true path from which distance metrics could then be calculated. The performance of each SSM is described in two ways; first, the distribution of location error magnitude (km) was quantified by computing its empirical cumulative distribution function (ECDF). Secondly, we repeated the adjusted quantile method of outlier detection described earlier for the latitudinal and longitudinal error components of the modelled locations.

## Results

A total of 78,521 Argos location estimates were received from all bearded seals (N = 6) and ringed seals (N = 10) over their tracking periods, which ranged from 18.8–283.5 d, with each individual providing a mean of 21.6 (± 0.94) Argos locations per day. Pre-processing with a 27 ms^-1^ speed filter removed 2.1% of the Argos location estimates. In concordance with other marine mammal tracking studies, the distribution of LCs was heavily skewed towards classes ‘A’ and ‘B’ (S1 Fig) though the entire dataset contained only 31 LC ‘Z’ location estimates with 12 remaining after coarse speed filtering. The same individuals provided a total of 30,711 GPS locations over the same tracking periods, averaging 8.3 (± 0.01) GPS locations per day. Each animal had approximately 280.4 (± 79.2; range 45 to 1210) Argos and GPS location estimates that were within 5 mins of each other. Derived 68^th^ percentile estimates for LCs 0, A and B were considerably improved compared to those generated from similar studies using LS Argos data (Table 1), with the SRUKF Argos data from LCs ‘A’ and ‘B’ errors estimated at 1.71 km and 2.19 km, respectively. Except for LC 3, less than 6% of all location estimates were classified as outliers (S2 Fig) and there was no latitudinal offset (Rayleighs Test Z = 0.01, p = 0.45; S3A Fig). However, the 95^th^ percentile of all estimated errors was less than 5.5 km from the true location for both species combined and followed a circular-normal distribution (Rayleighs Test Z = 0.01, p = 0.74; S3B Fig).

**Table 1:** Summary statistics for 68^th^ and 95^th^ percentiles of spatial errors (and their latitudinal and longitudinal components) associated with each Argos Location Class (LC) for Kalman-filtered Argos telemetry data, collected from bearded seals (N = 6) and ringed seals (N = 10) between 19^th^ July 2011 and 18^th^ April 2013 along the west coast of Svalbard.

Argos location class		68th percentile			95th percentile			Fitted T Distribution parameters							
	N	Distance (km)	Latitude	Longitude	Distance (km)	Latitude	Longitude	Distance		Latitude			Longitude		
								Theta	DF	Theta	DF	Theta	DF
**Bearded seals**													
**3**	10	0.58	0.25	0.40	0.82	0.53	0.79	-	-	-	-	-	-
**2**	37	0.61	0.44	0.34	1.33	1.09	0.84	0.30	4.63	0.27	2.18	0.21	1.73
**1**	40	1.14	0.86	0.62	2.19	2.01	1.27	0.45	2.59	0.55	2.18	0.62	9.13
**0**	37	2.42	1.89	1.47	4.63	3.64	2.96	1.05	10.01	1.78	50.21	1.47	1.56
**A**	222	1.38	0.84	0.89	5.61	2.77	3.63	0.46	1.27	0.69	1.91	0.64	1.75
**B**	1938	2.00	1.31	1.17	4.61	3.58	3.36	0.90	2.63	1.05	2.55	0.94	2.64
**Ringed Seals**													
**3**	16	0.47	0.27	0.31	0.92	0.69	0.77	0.10	0.57	0.15	0.66	0.16	0.68
**2**	29	0.74	0.49	0.47	1.47	1.35	0.88	-	-	0.44	3.18	-	-
**1**	28	1.56	1.24	0.77	2.67	2.15	1.58	0.80	9.28	1.30	3.93	0.83	7.07
**0**	14	2.58	1.83	1.70	26.43	26.13	5.65	0.85	1.00	0.78	0.81	0.76	1.15
**A**	174	1.65	1.12	0.99	4.90	2.78	3.71	0.69	1.74	0.77	1.85	0.69	1.72
**B**	2014	2.04	1.27	1.28	5.75	4.16	4.13	0.78	1.42	0.88	1.51	0.90	1.66
**Combined**													
**3**	26	0.57 (0.51, 0.49)	0.26	0.36	0.96	0.82	0.91	0.15	0.87	0.18	0.92	0.17	0.81
**2**	66	0.74(0.67, 1.01)	0.45	0.43	1.47	1.31	0.89	0.36	6.73	0.34	2.53	0.39	8.35
**1**	68	1.32 (1.02, 1.20)	1.00	0.64	2.67	2.10	1.48	0.61	4.34	0.79	3.56	0.71	14.19
**0**	51	2.42 (4.15, 4.18)	1.88	1.61	4.67	3.73	3.07	0.85	1.84	1.35	2.01	1.26	3.44
**A**	396	1.51 (10.19, 6.19)	1.01	0.95	5.07	2.82	3.71	0.58	1.52	0.72	1.87	0.66	1.72
**B**	3952	2.02 (9.24, 10.28)	1.30	1.22	5.29	3.96	3.66	0.82	1.75	0.95	1.82	0.90	1.95

Values in parentheses in the combined 68^th^ percentile estimates represent data derived from Least Squares processed Argos telemetry data taken from [9] and [12], respectively. Dashed lines represent a failure to converge on a distribution parameter by maximum likelihood. The 68^th^ Percentile error estimates from LC 3,2 and 1 were comparable to those described elsewhere, however the estimates for the lower classes were considerably smaller.

The distributions of longitudinal and latitudinal LC errors between each species were similar (Kolmogorov-Smirnov two-sample test D > 0.32, p > 0.06 in each case) suggesting that combining the error estimates for further analysis was appropriate (Table 1). Descriptors of LC errors (overall error estimates and their longitudinal and latitudinal components) for each species were fitted with T distributions (Kolmogrov-Smirnov one-sample test D < 0.21, p>0.07 in all cases) and the parameters of τ and ʋ were estimated by maximum likelihood; these are presented in Table 1.

**Fig 1:**
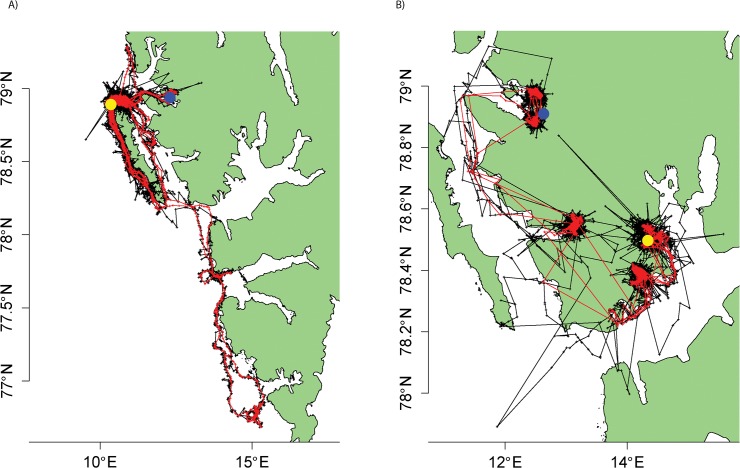
Raw Kalman filtered Argos tracks (black) and GPS tracks (red) for the focal A) bearded seal and B) ringed seal. Dots highlight instrumentation site (blue) and the final location (yellow).

**Fig 2:**
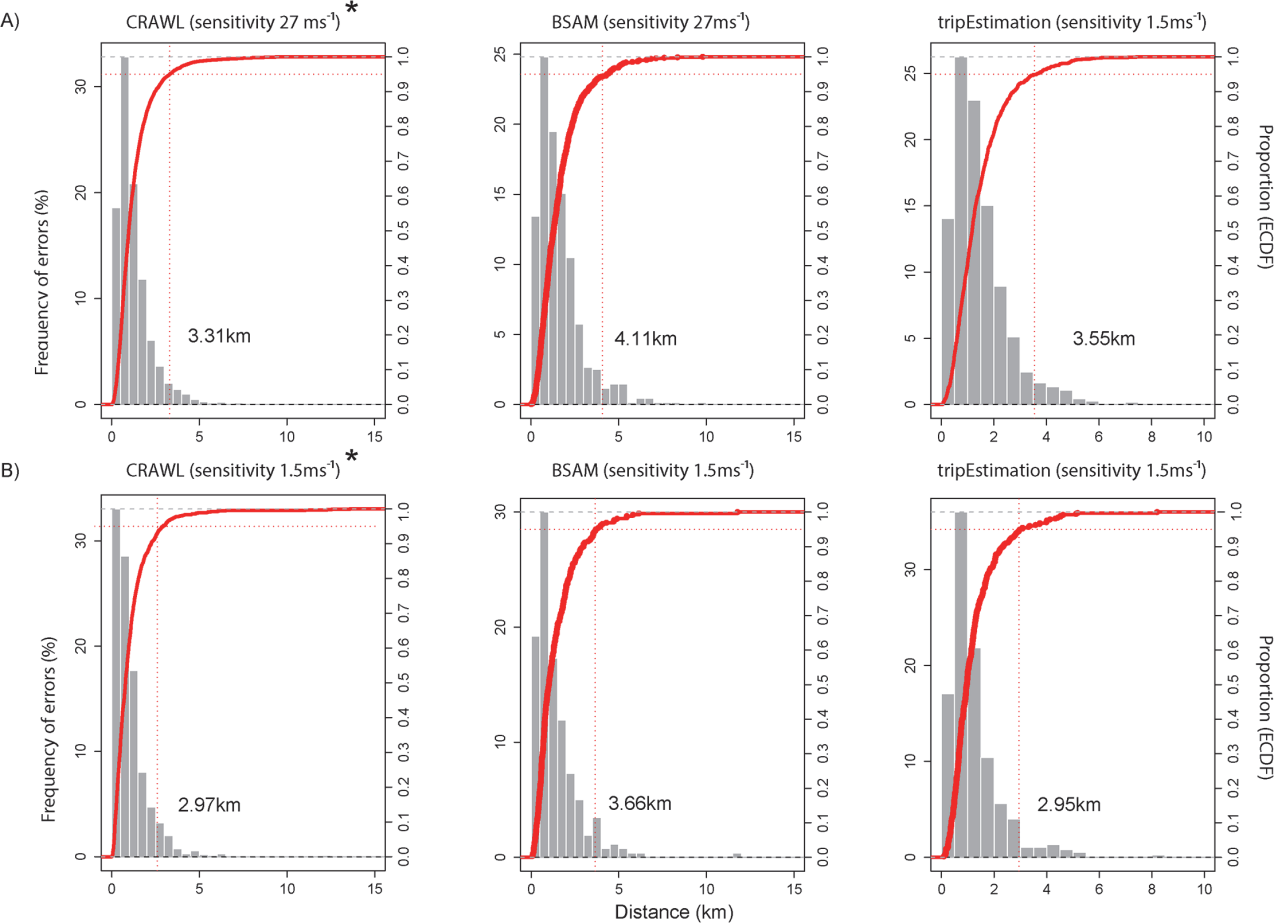
Spatial errors of the best modelled Argos location estimates relative to the true (GPS) position for the focal A) bearded seal and B) ringed seal using three different location error correction methods. * denotes use of the original error structure provided the most accurate modelled locations. Red dotted lines signify the 95% percentile of the empirical cumulative distribution function (right side axis) for each suite of errors. Generally, 95% of all errors were less than 4.2 km from the true position, with modelled locations being most accurate for the ringed seal.

**Fig 3:**
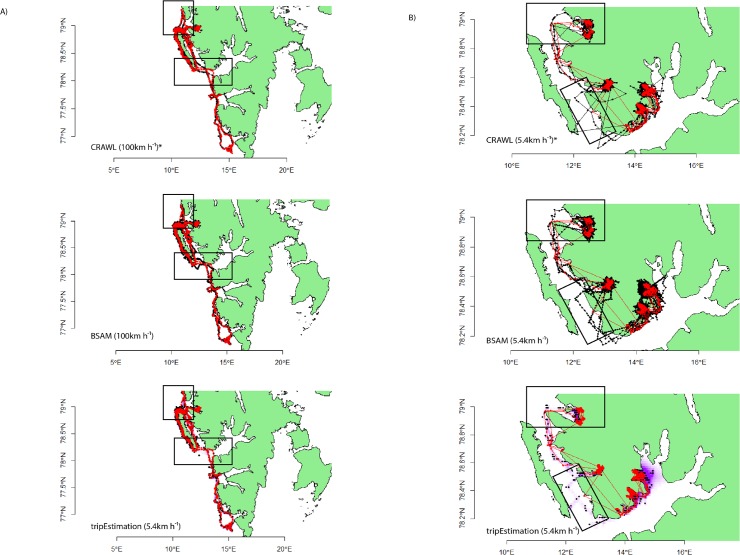
Optimal state-space modelled (SSM) Argos location data (black) overlaid with GPS locations (red) for the focal A) bearded seal and B) ringed seal. * denotes optimal model was constructed using the error structures derived from data in [10]. Modelled location and GPS point estimates are shown as black and red dots, respectively. For each tripEstimation model, the underlying time-spent along the full path estimate is shown in purple (obscured in the bearded seal plot in favour of displaying point estimates). Black boxes highlight areas of departure by each model from the true path. Modelled location estimates for the bearded seal fitted well with GPS locations with the exception of two areas, at the very northerly edge of its trajectory, and just south of Prins Karls Forland. Note the sparse numbers of GPS location estimates for the ringed seal, particularly during transit movements between fjords. Although there were ~50% fewer Argos location estimates for the ringed seal, all models reconstructed some aspects of these transit movements despite showing a number of erroneous land locations. The exception was the tripEstimation modelled data, presumably due to the effects of incorporating a land mask during the modelling process.

Both focal animals remained in the coastal waters of western Svalbard throughout the duration of tracking but displayed different movement patterns (Fig 1). Location acquisition rates for the bearded and ringed seal averaged 57.9 ± 0.7 tx d^-1^and 31.3 ± 0.84 tx d^-1^ (range 19–85 tx d^-1^ and 1–70 tx d^-1^), respectively, with ringed seal transmission rates appearing to decline throughout the tracking period (S4 Fig). The lowest speed threshold (1.5 ms^-1^) retained 77.3% and 86.9% of the raw Argos locations for the focal bearded and ringed seal, respectively (Table 2). Overall RMSE derived from modelled locations ranged from 2.42–5.54 km across both animals, with the continuous-time crawl package and tripEstimation package providing location estimates closest to the true path for the bearded and ringed seal, respectively. For the bearded seal, the crawl package performed best with data pre-processed at the highest speed threshold. Sensitivity to altering speed thresholds and alternate error structures on RMSE values for the bearded seal was minimal; however, for the ringed seal the pattern was reversed (Table 1). Both Bayesian methods performed best with the new error structures, improving location estimate accuracy by as much as 0.82 km (Table 2). For the ringed seal, the tripEstimation package performed best after raw data were pre-processed at the lowest speed threshold, providing location estimates as accurate as 2.42 km from true locations (Table 2). There were contrasting differences between the two animals in the sensitivity of the bsam model, which performed best at high thresholds for bearded seal Argos data, but worst for the ringed seal (Table 2). The empirical cumulative distributions of error magnitude showed that, for models with the lowest RMSE, 95% of all errors were less than 4.11 km (Fig 2). Based on idealised lognormal distributions generated from the data (S1 Table), typically less than 4.6% of the error estimates from these models could be classified as outliers, irrespective of the error structure used (S5 Fig). The optimal model for each sensitivity level for the bearded seal fitted well, with the exception of two regions in which all models departed from the GPS trajectories (Fig 3). However, plotting a realistic trajectory for the ringed seal using GPS data was not feasible given the lack of GPS location estimates during (presumed) transiting movements between fjords. Modelled Argos location data provided more detail regarding these transit paths, particularly at the southern end of its trajectory. Even though a greater number of Argos locations were available than GPS for the ringed seal dataset, the generally poor transmission rate of location estimates from either source prevented models from estimating a biologically reasonable path for this focal animal (Fig 3).

**Table 2:** Root Mean Square Error (RMSE) estimates (km) between modelled Argos locations and the true (GPS) position at varying sensitivities for three commonly-used location error correction models freely-available within the R statistical framework; crawl [14], bsam [17] and tripEstimation [31].

			RMSE (km)						
			crawl (N = 4,184)		bsam (N = 677)		tripEstimation		
Species	Speed threshold (ms^-1^)	Locations retained (%)	Old	New	Old	New	N	Old	New
**Bearded seal**	**100**	**95.9**	***2*.*79***	2.83	4.61	***4*.*25***	1321	4.59	4
	**54**	**94**	2.79	2.94	4.6	4.78	1319	4.69	3.97
	**10**	**85.1**	2.93	2.88	4.81	4.63	1319	4.15	3.92
	**5**	**77.3**	2.97	2.92	4.82	4.71	1097	3.99	***3*.*17***
			**N = 1,435**		**N = 260**				
**Ringed seal**	**100**	**99**	3.01	4.44	5.53	5.23	407	3.21	2.43
	**54**	**98.1**	3.15	5.05	5.54	5.33	405	3.05	2.45
	**10**	**91.5**	2.48	3.51	3.99	3.82	386	2.84	2.56
	**5**	**86.9**	***2*.*53***	3.32	3.81	***3*.*7***	375	2.51	***2*.*42***

‘Old’ and ‘New’ reflect the effect of applying the original error structures derived from data in [10] and errors estimated from data in the present study, respectively. Variable ‘N’ for the tripEstimation results reflects the reduction in the pre-processed dataset. For all models, the new error structures incorporated a correction for differences in the distance covered by one degree of longitude at high latitude was applied. Optimal model results are highlighted in ***bold italics***. Both bsam and tripEstimation models performed better with error structures derived from the current study, providing the most accurate location estimates at the lowest speed threshold with the exception of the bearded seal bsam model. Sensitivity of models to different speed thresholds was apparent, though the differences in error estimates were minimal.

## Discussion

The new SKURF filtering process claimed to provide a greatly increased number of more accurately estimated locations and more precisely described LC error structures than locations derived using the Least Squares (LS) algorithm that was used previously. We have documented the performance of Argos telemetry data derived from the SRUKF algorithm, relative to concurrently-collected Fastloc GPS data from wild animals as they moved through their environment. Our study shows that the 68^th^ percentile of errors from high-quality LCs (3,2 and 1) SRUKF-derived locations are comparable to those from similar studies using LS [9,10,12]. However there was a dramatic increase in the accuracy of lower-quality LCs (0,A and B) using SRUKF, with errors between 50–80% of the magnitude determined for LS Argos data and in-line with those reported by Argos [8]. Similarly, the precision of location estimates within each LC was also high with less than 6% of errors outside the theoretical distributions determined by the data and no directional bias in errors, which is commonly reported from LS Argos locations [10,12], Typically, researchers collecting telemetric data on marine wildlife collect data that are heavily skewed to low-quality LCs because of the behaviour of these animals and the environment in which they move. Thus, the increased accuracy of SRUKF will likely be of greatest benefit to researchers whose study organisms (and environments) tend to return low quality location estimates, such as most marine taxa [34,35,36]. However, given that the two focal animals in this study were tracked under the same extrinsic conditions the importance of animal behaviour in determining data quality and subsequent ability to accurately characterise movement should not be ignored.

### State-space model performance

Service Argos has been providing researchers with the ability to remotely collect wildlife telemetry data for over 30 years. The issues surrounding location accuracy and precision have been documented for almost as long [37]. However, the implementation of state space models (SSM) to the location estimation problem has permitted the incorporation of location uncertainty into movement models [17]. We demonstrate the sensitivity of three different, readily-available movement models to differences in data pre-processing and error covariance specification. The lowest speed threshold used (1.5ms^-1^) retained over 70% of raw location data, contrasting strongly with the ~30–40% retention shown in other studies using the Least Squares algorithm on Argos data [9,15], highlighting the lower number of aberrant location estimates provided by the SRUKF algorithm. The sensitivity of models to speed thresholds was evident in estimates of RMSE which varied by as much as 1 km; however, the majority were considerably less. For both Bayesian models tested, the most accurate location estimates were generated using the error structures derived from the data in this study, though RMSE values tended to differ between error structures by less than 900 m.

To our knowledge, there have been only three studies that have examined the performance of SSM in refining location estimates, despite the rapid increase in the application of SSM to telemetry data [17]. Our RMSE estimates are comparable to, or lower than, these studies with 95% of all location estimates being less than 4.11 km from the true position. The better performance of the SSM likely reflects the increased accuracy of lower quality LCs from SRUKF Argos data relative to the earlier LS filtering method, improved quantification of location error structures or a combination of both factors. In light of the improved accuracy of SRUKF Argos locations we described, the magnitude of differences in RMSE between old and new error structures was predictable. However, it is worth noting that our error structure also corrected for the conversion of error distance to degrees longitude at high latitude, an aspect that the original error structures failed to address. As such we recommend that attention is paid to longitudinal correction when collecting telemetry data at high latitudes; this might be particularly important when modelling Least Squares Argos data given its larger error estimates.

Interestingly, for both focal animals the old error structure performed best for the continuous-time CRAWL model though for the bearded seal the RMSE differences between error structures were often less than 150 m. Additionally, the difference in RMSE for the ringed seal CRAWL model results at all speed thresholds were up to an order of magnitude greater than seen in the bearded seal. The error structure in this model is simplistic relative to the other two and the ability to manipulate its effect on the location estimation process is limited. Most Argos and Fastloc GPS location estimates for the ringed seal were received while the animal was resident in one of three fjords. The relatively low number of Argos location estimates during transit movements between fjords coupled with the tighter error estimates we provide likely resulted in the poorer track model, relative to the greater flexibility afforded by the original error structure. This is evident in both the raw and modelled tracks presented in Fig 2 and S5 Fig.

Each ringed seal modelled track deviated noticeably from the linearly interpolated GPS track, primarily during periods of presumed transit between fjords. Relying exclusively on Fastloc GPS data during these transits to reconstruct movement paths would lead to either biologically meaningless trajectories (moving across considerable tracts of land) or the paths would overlook key areas of habitat use, best demonstrated at the northern-most extent of the ringed seal track. Similarly, the bearded seal’s modelled tracks deviated from GPS-derived track in several regions even though there appeared to be a sufficient number of Argos Kalman locations to adequately infer a realistic trajectory. The reasons for this are unclear, and may simply be an artefact of the error modelling process.

### Implications for ecological inference

Accurately characterising the movement of animals is an essential first step to further studies which attempt to assess habitat usage and areas of ecological significance [38]. For example, there are several methods available to estimate the hidden (unobserved) behavioural state of an animal based on derived aspects of its movement parameters such as changes in turning angle or speed [39,40] which are underpinned by accurate location estimation. Commonly, increased track tortuosity and slower travelling speeds are assumed to reflect animals searching for food whereas directed, higher speed movements are interpreted as transiting behaviour between food patches [41]. From a management and conservation perspective, the utilisation of space (habitats) by animals is central to the decision-making process [42]. Multi-sensor telemetry devices can now collect high resolution environmental data that can be linked to animal movement patterns to infer preferred habitat structures at individual, population and species levels [43] as well as characterising areas of ecological significance to guilds of predators [38]. These animal-borne sensors can be supplemented by additional remote-sensed data from satellites; oceanographic anomalies and terrestrial biophysical parameterisation are widely available in varying spatial resolutions.

The differences in temporal scale over which Argos and Fastloc GPS locations are collected would determine the spatial scale over which state-space modelled locations can be estimated. The increased number of sensors being integrated into telemetry instruments places considerable demands on available satellite bandwidth, forcing data to be summarised, compressed and prioritised into the messages that are sent via Argos [44]. The SRUKF algorithm returns estimated locations from any number of messages, unlike the earlier Least Squares method which required a minimum of two messages. Conversely, Fastloc GPS locations are returned as a random sample of the total number of fixes conditional on the user-programmed settings of the instrument. The mean number of daily Argos locations received in this study was about one per hour. In comparison, Fastloc GPS locations were received approximately one-third as often (one every three hours). Thus, given that errors around SRUKF Argos location estimates can now be appropriately modelled, the greater number of estimates may be tractable to reconstructing biologically-meaningful tracks. Consequently, Argos-derived estimates of animal movement may be more amenable to further behavioural-based study at a higher resolution than less-frequent but undoubtedly more accurate Fastloc GPS point estimates.

## Conclusions

We show that the new Argos SRUKF algorithm generates location estimates with accuracies that are often less than 4 km from true positions when compared to concurrently-collected Fastloc GPS data, and that these data can be used to reconstruct realistic animal trajectories. Furthermore, the quantitative estimates of error provided in the current study can be used as input for numerous location-correction models. Although only two focal animals were used to demonstrate the utility of SSM with the new Argos location data, we discussed the relative importance of animal behaviour in determining the quality of track reconstruction. However, we recommend that telemetry users consider the influence of environmental factors, including latitudinal coverage of satellites and the effects of temperature on transmitter function with respect to the performance of Argos location estimation algorithms and to consider if, in this context, our findings are appropriate to use in their studies.

## Supporting Information

S1 Fig Distribution of Argos Location Classes (LCs) in the entire dataset. Similar to other marine mammal telemetry studies, raw Argos locations were heavily skewed towards the least accurate LCs (‘A’ and ‘B’), though only 31 LC ‘Z’ were present in the dataset.
(TIF)

S2 Fig Assessment of Service Argos Location Class (LC) precision for telemetry data derived from the Square Root Unscented Kalman Filter (SRUKF) algorithm. ‘N’ represents number of paired Argos-GPS locations used to quantify the errors. Robust estimates of Mahalanobis Distances were constructed from latitudinal and longitudinal errors for each paired location. Black lines indicate cumulative distribution functions (Y axis) of 100 simulated datasets (grey) generated from an ‘ideal’ X^2^ distribution. Dotted grey lines highlight the adaptive cut-off used to define outliers described in Filzmoser et al. (2005). Estimated error outliers typically made up < 6% of all Argos-GPS paired location estimates. Note the effect of smaller sample sizes at the higher quality LCs on the simulated distributions.
(TIF)

S3 Fig Direction and magniture of spatial errors compared to true (GPS) locations of A) all Kalman-filtered Argos location estimates and B) the 95^th^ percentile of error estimates collected from bearded seals (N = 6) and ringed seals (N = 10) between 19^th^ July 2011 and 18^th^ April 2013 along the west coast of Svalbard. North is represented by ‘0’. Radial values reflect error magnitude in km. Large outliers were observed which did not follow a circular normal distribution, following a north-south offset. When only the 95^th^ percentile of errors was considered, the directional bias disappeared and the error distribution followed a circular normal pattern.
(TIF)

S4 Fig Number of daily Argos location estimates received for the focal animals (bearded seal = black; ringed seal = red). Smoothed LOESS curves are fitted to highlight the temporal trend in transmission rates. In total, 13,415 and 7,937 location estimates were received over 240 d and 255 d for the bearded and ringed seal, respectively. The bearded seal transmitted almost twice as many locations over a similar time period, with a relatively constant rate of transmission while the number of daily location estimates received from the ringed seal decreased throughout the tracking period.
(TIF)

S5 Fig Adaptive outlier detection of modelled SRUKF Argos telemetry data relative to concurrently-collected GPS data from a focal A) Bearded seal and b) Ringed seal. (TIF)

S1 File Electronic tag program settings. (DOCX)

S2 File Telemetry data for focal animals. (PDF)

S1 Table Parameters describing idealised (outlier-free) mean and standard deviation (±SD of estimates) of lognormal probability distributions of Mahalanobis distances derived from location error magnitude between optimally-modelled Argos location and true (GPS) locations. The background and implementation of each state space model is detailed in text and is explained in further detail in Johnson *et al*. (2008), Jonsen *et al*. (2005) and Sumner *et al*. (2009). Data collected from two focal animals tracked using GPS-CTD-SRDL instruments between 19^th^ July 2011 and 18^th^ April 2013 along the west coast of Svalbard. These data were used to determine the precision of modelled location estimates using the adjusted quantile outlier detection method (S5 Fig)
(DOCX)
