# Supplementary figures and images for: The Argos-CLS Kalman Filter: Error Structures and State-Space Modelling Relative to Fastloc GPS Data
Source: PLoS One. 2015 Apr 23;10(4):e0124754. doi: 10.1371/journal.pone.0124754 (PMC4408085; doi:10.1371/journal.pone.0124754)

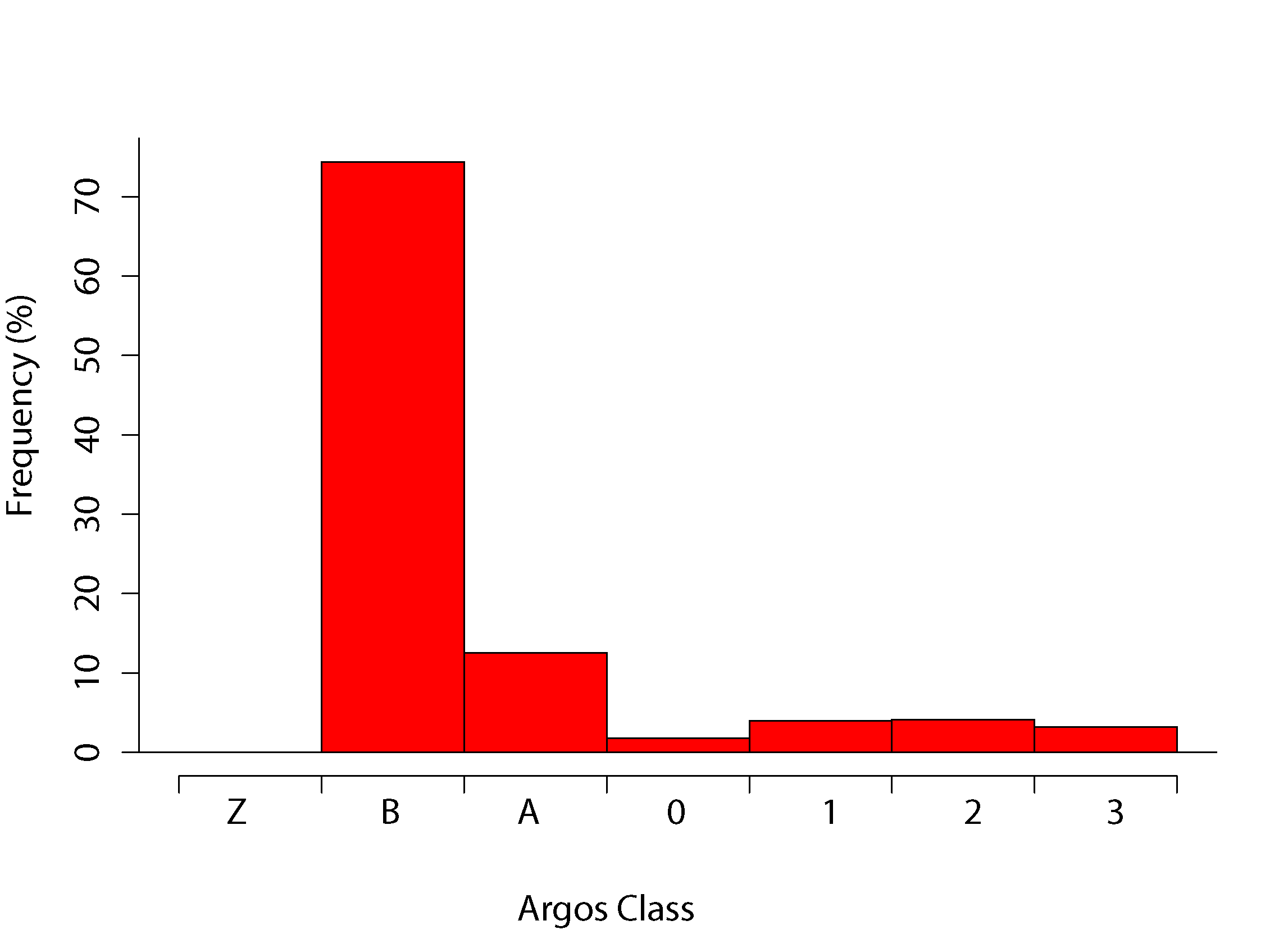

Supplement: S1 Fig — Similar to other marine mammal telemetry studies, raw Argos locations were heavily skewed towards the least accurate LCs (‘A’ and ‘B’), though only 31 LC ‘Z’ were present in the dataset. (TIF) [file pone.0124754.s001.tif]

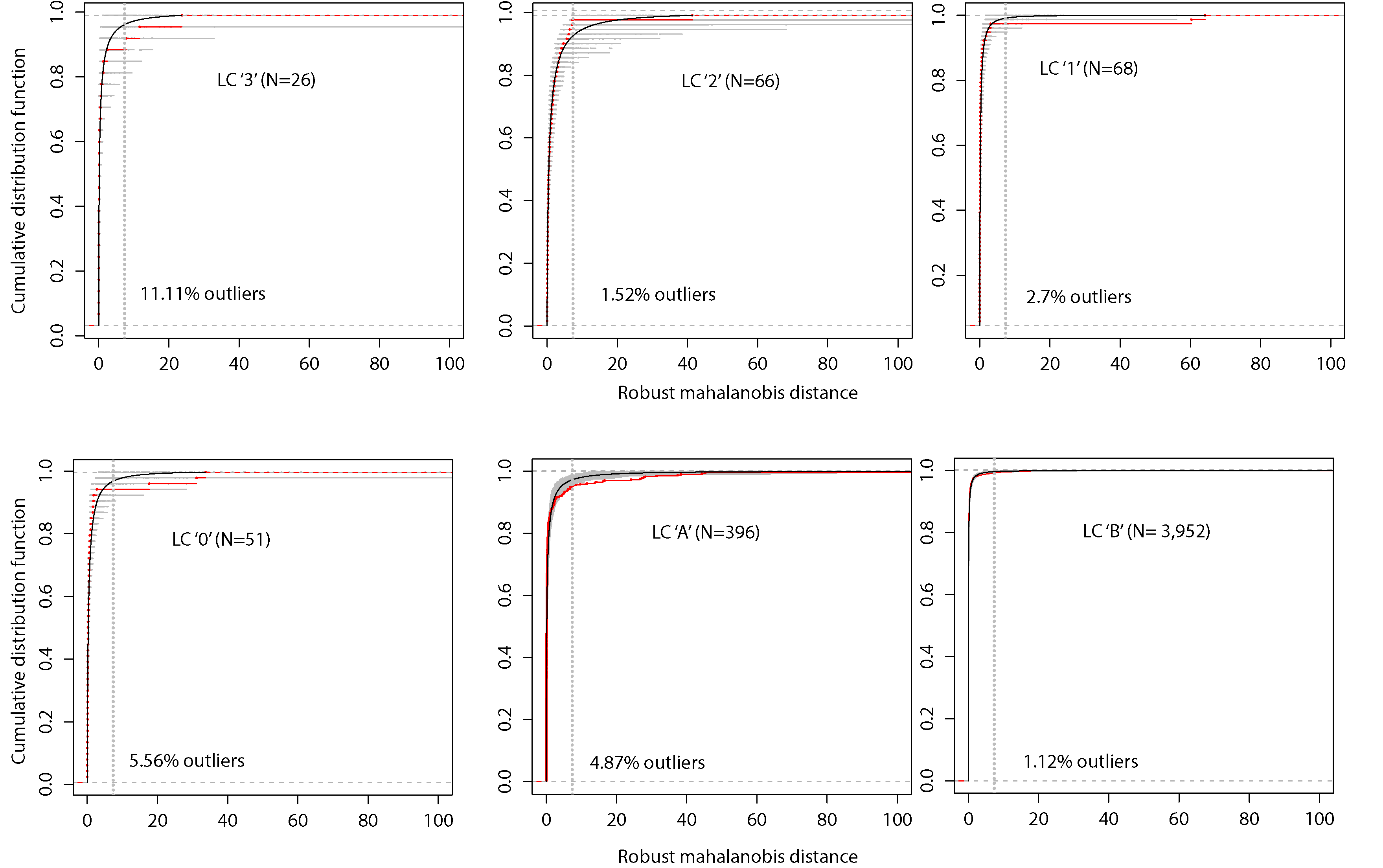

Supplement: S2 Fig — ‘N’ represents number of paired Argos-GPS locations used to quantify the errors. Robust estimates of Mahalanobis Distances were constructed from latitudinal and longitudinal errors for each paired location. Black lines indicate cumulative distribution functions (Y axis) of 100 simulated datasets (grey) generated from an ‘ideal’ X2 distribution. Dotted grey lines highlight the adaptive cut-off used to define outliers described in Filzmoser et al. (2005). Estimated error outliers typically made up < 6% of all Argos-GPS paired location estimates. Note the effect of smaller sample sizes at the higher quality LCs on the simulated distributions. (TIF) [file pone.0124754.s002.tif]

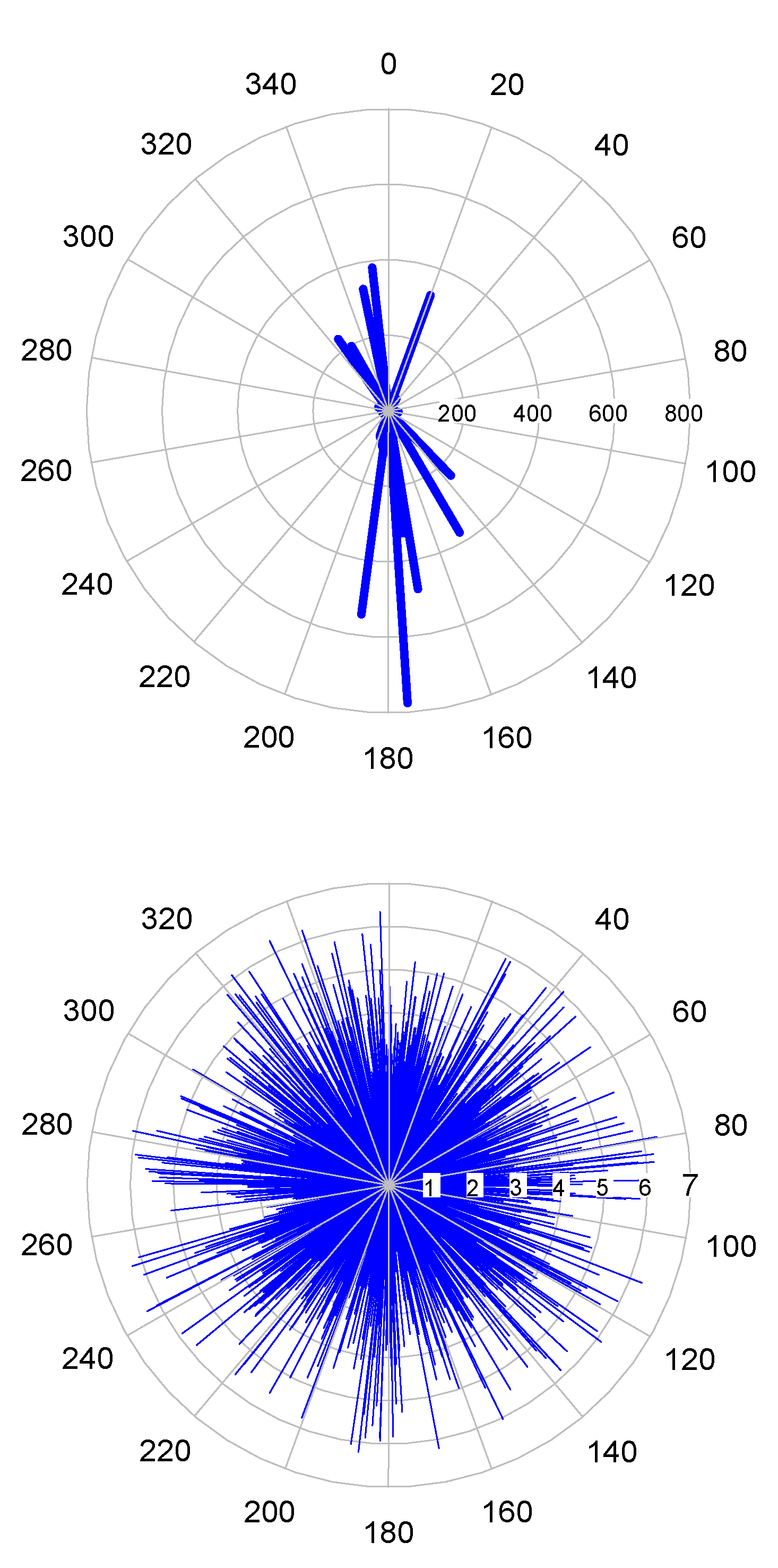

Supplement: S3 Fig — North is represented by ‘0’. Radial values reflect error magnitude in km. Large outliers were observed which did not follow a circular normal distribution, following a north-south offset. When only the 95th percentile of errors was considered, the directional bias disappeared and the error distribution followed a circular normal pattern. (TIF) [file pone.0124754.s003.tif]

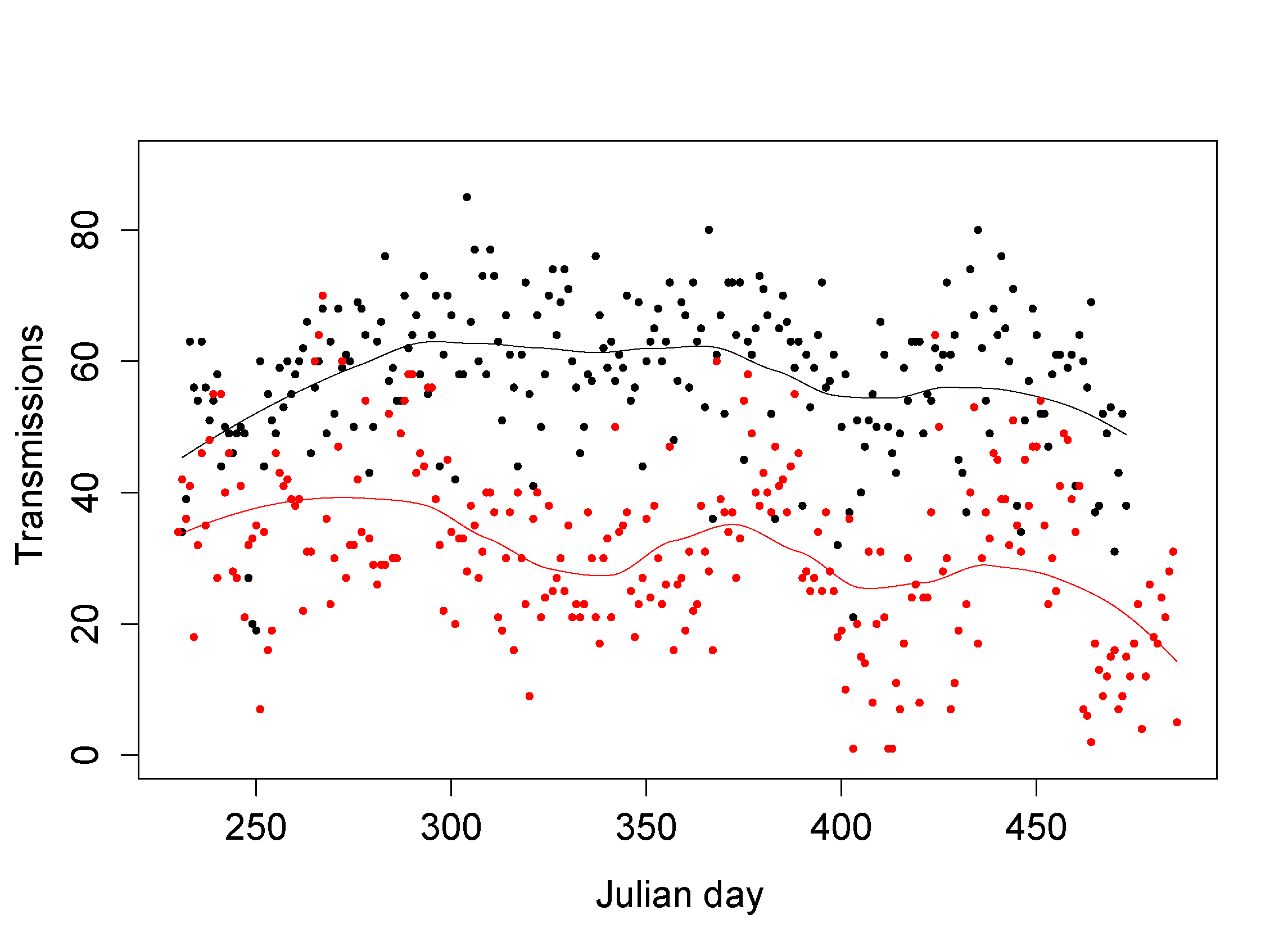

Supplement: S4 Fig — Smoothed LOESS curves are fitted to highlight the temporal trend in transmission rates. In total, 13,415 and 7,937 location estimates were received over 240 d and 255 d for the bearded and ringed seal, respectively. The bearded seal transmitted almost twice as many locations over a similar time period, with a relatively constant rate of transmission while the number of daily location estimates received from the ringed seal decreased throughout the tracking period. (TIF) [file pone.0124754.s004.tif]

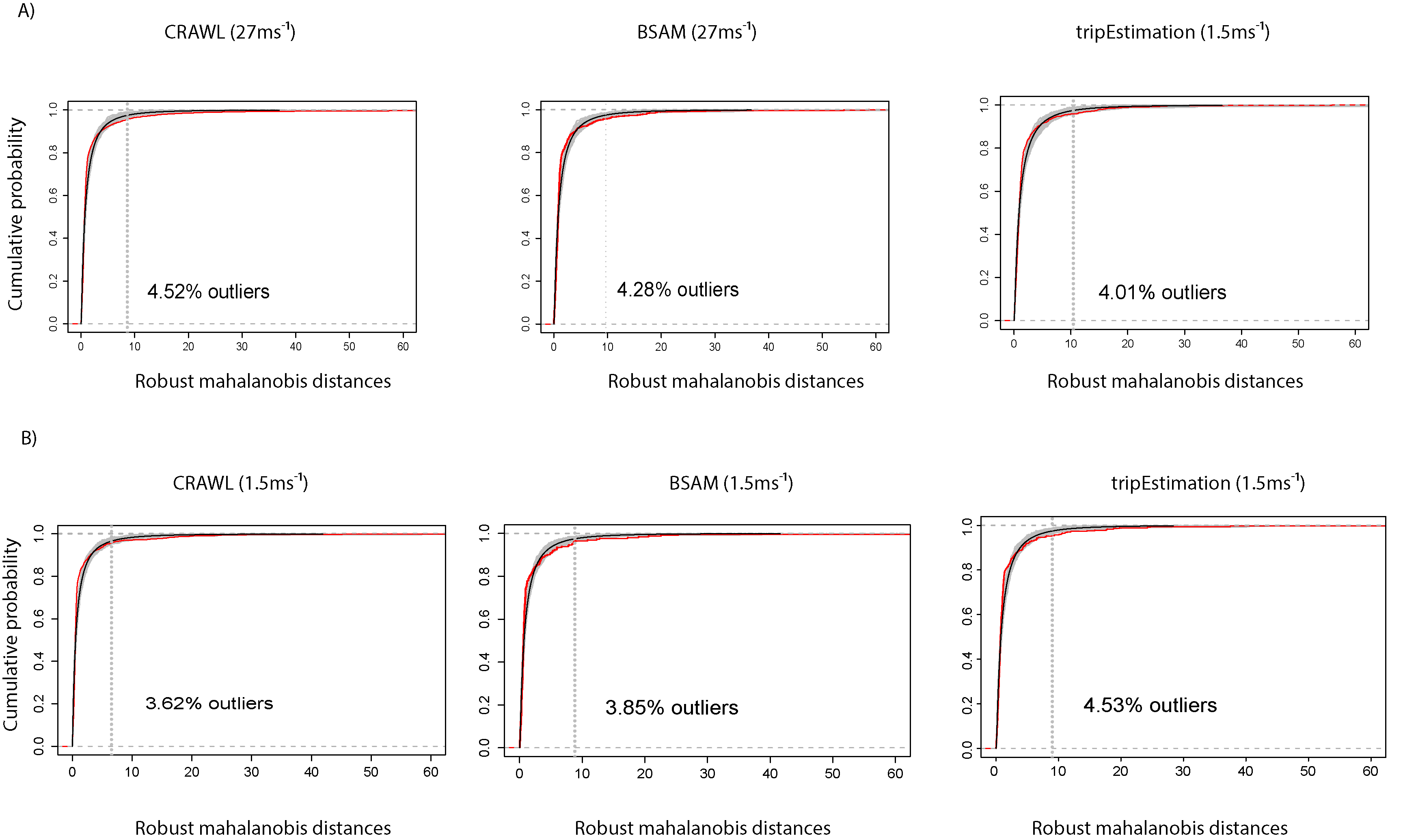

Supplement: S5 Fig — (TIF) [file pone.0124754.s005.tif]
