# Supplementary material for: The Argos-CLS Kalman Filter: Error Structures and State-Space Modelling Relative to Fastloc GPS Data
Source: PLoS One. 2015 Apr 23;10(4):e0124754. doi: 10.1371/journal.pone.0124754 (PMC4408085; doi:10.1371/journal.pone.0124754)
Supplement: S1 File — (DOCX) [file pone.0124754.s006.docx]

**Supplementary Information : GPS-CTD-SRDL Tag program parameters**

The information below outlines the parameters programmed into each tag deployed on adult female bearded seals (N=6) and ringed seals (N=11) between August 2011 and May 2013.

Transmission targets:

100000 transmissions after 300 days

In Haulouts: ON (one tx every 1 min) for first 9 hours

then cycling OFF for 2 hours, ON for 2 hours

Check sensors every 4 secs

When near surface (shallower than 6m), check wet/dry every 1 sec

Consider wet/dry sensor failed if wet for 7 days or dry for 99 days

Dives start when wet and below 1.5m for 8 secs

and end when dry, or above 1.5m

Do not separate 'Deep' dives

A cruise begins if there has been no dive for 9 mins

A haulout begins when dry for 10 mins

and ends when wet for 40 secs

Dive shape (normal dives):

4 points per dive using broken-stick algorithm

Dive shape (deep dives):

none

CTD profiles: max 500 dbar up to 2 dbar in 1 dbar bins.

Temperature: Collected, Stored. Valid range: -3 to 13.3 degC

Conductivity: Collected, Not stored.

Salinity: Calculated, Stored. Valid range: 6 to 38.7

Fluorescence: Not collected.

Oxygen: Not collected.

Send the deepest upcast in each 4-hour period.

An upcast is collected if the depth exceeds:

200m in hour 1

100m in hour 2

50m in hour 3

30m in hour 4

In addition, a replacement upcast is collected if the depth exceeds the depth of the current upcast by 20%.

During profile, sample CTD sensor every 1 seconds.

Each profile contains 16 cut points

consisting of 8 fixed points, minimum depth, maximum depth, 6 broken-stick points

A set of fixed point is selected according to depth:

10,12,14,16,18,20,24,22

10,14,20,26,30,40,50,36

10,20,30,40,50,60,100,80

10,20,30,40,50,100,150,60

10,20,30,50,100,150,200,80

10,20,50,100,150,200,300,30

10,20,50,100,200,300,400,150

10,20,50,100,200,300,500,400

GPS fixes:

Number of GPS attempts allowed: unlimited

Cut-off date for GPS attempts: unlimited

Discard results with fewer than 5 satellites

Haulouts: Increase interval to 10x normal after first success in haulout

TRANSMISSION BUFFERS (in RAM):

Dives in groups of 3 (6.25 days @ 10mins/dive): 300 = 1200 bytes

No 'deep' dives

Haulouts: 30 = 120 bytes

6-hour Summaries in groups of 3 (7.5 days): 10 = 40 bytes

No Timelines

Cruises: 30 = 120 bytes

No Diving periods

No Spot depths

No Emergence records

No Dive duration histograms

No Max depth histograms

No Depth & Temperature histograms

CTD casts (10 days): 60 = 240 bytes

GPS fixes (variable: 6.94444 days if interval is 20 mins): 500 = 2000 bytes

No Spot CTD's

TOTAL 3720 bytes (of about 21000 available)
