# Supplementary material for: The Argos-CLS Kalman Filter: Error Structures and State-Space Modelling Relative to Fastloc GPS Data
Source: PLoS One. 2015 Apr 23;10(4):e0124754. doi: 10.1371/journal.pone.0124754 (PMC4408085; doi:10.1371/journal.pone.0124754)
Supplement: S2 File — (PDF) [file pone.0124754.s007.pdf]

ref	PTT	D_DATE	LQ	LAT	LON
ct92-M305Pete-12	120362	18/08/2012	0	78.989	12.526
ct92-M305Pete-12	120362	18/08/2012	3	78.932	12.308
ct92-M305Pete-12	120362	18/08/2012	2	78.929	12.331
ct92-M305Pete-12	120362	18/08/2012	-1	78.925	12.326
ct92-M305Pete-12	120362	18/08/2012	-1	78.929	12.446
ct92-M305Pete-12	120362	18/08/2012	-1	78.927	12.451
ct92-M305Pete-12	120362	18/08/2012	1	78.95	12.436
ct92-M305Pete-12	120362	18/08/2012	3	78.958	12.45
ct92-M305Pete-12	120362	18/08/2012	2	78.963	12.428
ct92-M305Pete-12	120362	18/08/2012	1	78.98	12.355
ct92-M305Pete-12	120362	18/08/2012	3	78.973	12.407
ct92-M305Pete-12	120362	18/08/2012	1	78.97	12.414
ct92-M305Pete-12	120362	18/08/2012	3	78.964	12.444
ct92-M305Pete-12	120362	18/08/2012	2	78.969	12.417
ct92-M305Pete-12	120362	18/08/2012	2	78.97	12.433
ct92-M305Pete-12	120362	18/08/2012	2	78.969	12.445
ct92-M305Pete-12	120362	18/08/2012	2	78.972	12.443
ct92-M305Pete-12	120362	18/08/2012	2	78.974	12.443
ct92-M305Pete-12	120362	18/08/2012	2	78.976	12.357
ct92-M305Pete-12	120362	18/08/2012	2	78.971	12.404
ct92-M305Pete-12	120362	18/08/2012	2	78.972	12.428
ct92-M305Pete-12	120362	18/08/2012	3	78.967	12.432
ct92-M305Pete-12	120362	18/08/2012	3	78.968	12.429
ct92-M305Pete-12	120362	18/08/2012	2	78.968	12.423
ct92-M305Pete-12	120362	18/08/2012	1	78.967	12.409
ct92-M305Pete-12	120362	19/08/2012	-1	78.973	12.448
ct92-M305Pete-12	120362	19/08/2012	-2	78.973	12.323
ct92-M305Pete-12	120362	19/08/2012	1	78.975	12.461
ct92-M305Pete-12	120362	19/08/2012	3	78.975	12.45
ct92-M305Pete-12	120362	19/08/2012	-2	78.972	12.287
ct92-M305Pete-12	120362	19/08/2012	2	78.968	12.398
ct92-M305Pete-12	120362	19/08/2012	3	78.968	12.423
ct92-M305Pete-12	120362	19/08/2012	-2	78.971	12.425
ct92-M305Pete-12	120362	19/08/2012	1	78.993	12.429
ct92-M305Pete-12	120362	19/08/2012	-2	78.967	12.43
ct92-M305Pete-12	120362	19/08/2012	1	78.968	12.439
ct92-M305Pete-12	120362	19/08/2012	-1	78.963	12.405
ct92-M305Pete-12	120362	19/08/2012	3	78.959	12.405
ct92-M305Pete-12	120362	19/08/2012	1	78.971	12.383
ct92-M305Pete-12	120362	19/08/2012	1	78.967	12.371
ct92-M305Pete-12	120362	19/08/2012	-2	79.001	12.693
ct92-M305Pete-12	120362	19/08/2012	-2	78.969	12.479
ct92-M305Pete-12	120362	19/08/2012	-2	78.969	12.483
ct92-M305Pete-12	120362	19/08/2012	-2	78.969	12.484
ct92-M305Pete-12	120362	19/08/2012	-2	78.962	12.454
ct92-M305Pete-12	120362	19/08/2012	-2	78.01	8.719
ct92-M305Pete-12	120362	19/08/2012	-1	78.297	11.5
ct92-M305Pete-12	120362	19/08/2012	-1	78.973	12.574
ct92-M305Pete-12	120362	19/08/2012	2	78.984	12.419
ct92-M305Pete-12	120362	19/08/2012	-2	78.983	12.419
ct92-M305Pete-12	120362	20/08/2012	-2	78.986	12.423
ct92-M305Pete-12	120362	20/08/2012	-2	78.976	12.403
ct92-M305Pete-12	120362	20/08/2012	-2	78.975	12.458
ct92-M305Pete-12	120362	20/08/2012	-2	78.987	12.425
ct92-M305Pete-12	120362	20/08/2012	-2	78.986	12.432
ct92-M305Pete-12	120362	20/08/2012	-2	78.991	12.454
ct92-M305Pete-12	120362	20/08/2012	-2	78.959	12.564
ct92-M305Pete-12	120362	20/08/2012	-2	78.958	12.552
ct92-M305Pete-12	120362	20/08/2012	-1	78.962	12.474
ct92-M305Pete-12	120362	20/08/2012	-2	78.974	12.471
ct92-M305Pete-12	120362	20/08/2012	-2	78.974	12.471
ct92-M305Pete-12	120362	20/08/2012	-1	78.991	12.43
ct92-M305Pete-12	120362	20/08/2012	-2	78.972	12.425
ct92-M305Pete-12	120362	20/08/2012	-2	78.991	12.43
ct92-M305Pete-12	120362	20/08/2012	-2	78.967	12.488
ct92-M305Pete-12	120362	20/08/2012	-2	78.972	12.487
ct92-M305Pete-12	120362	20/08/2012	-2	78.972	12.488

ct92-M305Pete-12	120362	20/08/2012	-2	78.969	12.48
ct92-M305Pete-12	120362	20/08/2012	-2	78.972	12.453
ct92-M305Pete-12	120362	20/08/2012	-1	78.966	12.394
ct92-M305Pete-12	120362	20/08/2012	-2	78.968	12.399
ct92-M305Pete-12	120362	20/08/2012	-2	78.966	12.393
ct92-M305Pete-12	120362	20/08/2012	0	78.976	12.347
ct92-M305Pete-12	120362	20/08/2012	-2	78.974	12.329
ct92-M305Pete-12	120362	20/08/2012	-2	78.964	12.332
ct92-M305Pete-12	120362	20/08/2012	-2	78.956	12.312
ct92-M305Pete-12	120362	20/08/2012	-2	78.956	12.307
ct92-M305Pete-12	120362	20/08/2012	-2	78.953	12.267
ct92-M305Pete-12	120362	20/08/2012	-1	78.948	12.239
ct92-M305Pete-12	120362	20/08/2012	-2	78.945	12.196
ct92-M305Pete-12	120362	20/08/2012	-1	78.94	12.198
ct92-M305Pete-12	120362	20/08/2012	-2	78.941	12.17
ct92-M305Pete-12	120362	20/08/2012	-1	78.919	12.067
ct92-M305Pete-12	120362	20/08/2012	-2	78.924	12.002
ct92-M305Pete-12	120362	20/08/2012	-2	78.922	11.986
ct92-M305Pete-12	120362	20/08/2012	-2	78.936	11.951
ct92-M305Pete-12	120362	20/08/2012	-2	78.945	11.928
ct92-M305Pete-12	120362	20/08/2012	-2	78.942	12.101
ct92-M305Pete-12	120362	20/08/2012	-2	78.935	11.906
ct92-M305Pete-12	120362	20/08/2012	-2	78.933	11.867
ct92-M305Pete-12	120362	20/08/2012	-2	78.942	11.805
ct92-M305Pete-12	120362	20/08/2012	-2	78.939	11.801
ct92-M305Pete-12	120362	20/08/2012	-2	78.942	11.799
ct92-M305Pete-12	120362	20/08/2012	-2	78.956	11.767
ct92-M305Pete-12	120362	20/08/2012	-2	78.957	11.74
ct92-M305Pete-12	120362	20/08/2012	-2	78.958	11.658
ct92-M305Pete-12	120362	20/08/2012	-2	78.964	11.682
ct92-M305Pete-12	120362	20/08/2012	-2	78.96	11.685
ct92-M305Pete-12	120362	20/08/2012	-2	78.955	11.596
ct92-M305Pete-12	120362	20/08/2012	-2	78.964	11.541
ct92-M305Pete-12	120362	20/08/2012	-2	78.965	11.503
ct92-M305Pete-12	120362	20/08/2012	-2	78.974	11.351
ct92-M305Pete-12	120362	20/08/2012	-2	78.971	11.349
ct92-M305Pete-12	120362	20/08/2012	-1	78.99	11.356
ct92-M305Pete-12	120362	20/08/2012	-2	78.978	11.497
ct92-M305Pete-12	120362	20/08/2012	-2	78.957	11.355
ct92-M305Pete-12	120362	20/08/2012	-2	78.967	11.439
ct92-M305Pete-12	120362	20/08/2012	-2	78.967	11.438
ct92-M305Pete-12	120362	20/08/2012	-2	78.968	11.434
ct92-M305Pete-12	120362	21/08/2012	-2	78.979	11.475
ct92-M305Pete-12	120362	21/08/2012	-2	78.978	11.477
ct92-M305Pete-12	120362	21/08/2012	2	78.977	11.42
ct92-M305Pete-12	120362	21/08/2012	-2	78.979	11.428
ct92-M305Pete-12	120362	21/08/2012	-1	78.965	11.399
ct92-M305Pete-12	120362	21/08/2012	-2	78.966	11.401
ct92-M305Pete-12	120362	21/08/2012	-1	78.981	11.447
ct92-M305Pete-12	120362	21/08/2012	-2	78.979	11.426
ct92-M305Pete-12	120362	21/08/2012	-2	78.979	11.423
ct92-M305Pete-12	120362	21/08/2012	-2	78.969	11.447
ct92-M305Pete-12	120362	21/08/2012	-2	78.966	11.42
ct92-M305Pete-12	120362	21/08/2012	-2	78.965	11.263
ct92-M305Pete-12	120362	21/08/2012	2	78.957	11.249
ct92-M305Pete-12	120362	21/08/2012	2	78.95	11.265
ct92-M305Pete-12	120362	21/08/2012	1	78.944	11.294
ct92-M305Pete-12	120362	21/08/2012	0	78.954	11.273
ct92-M305Pete-12	120362	21/08/2012	-1	78.94	11.284
ct92-M305Pete-12	120362	21/08/2012	1	78.936	11.331
ct92-M305Pete-12	120362	21/08/2012	-1	78.94	11.329
ct92-M305Pete-12	120362	21/08/2012	-1	78.936	11.326
ct92-M305Pete-12	120362	21/08/2012	0	78.939	11.319
ct92-M305Pete-12	120362	21/08/2012	-2	78.939	11.329
ct92-M305Pete-12	120362	21/08/2012	-2	78.951	11.274
ct92-M305Pete-12	120362	21/08/2012	-2	78.932	11.192
ct92-M305Pete-12	120362	21/08/2012	-2	78.938	11.25
ct92-M305Pete-12	120362	21/08/2012	-2	78.942	11.173

ct92-M305Pete-12	120362	21/08/2012	-1	78.935	11.268
ct92-M305Pete-12	120362	21/08/2012	-2	78.931	11.261
ct92-M305Pete-12	120362	21/08/2012	-2	78.93	11.253
ct92-M305Pete-12	120362	21/08/2012	-2	78.934	11.37
ct92-M305Pete-12	120362	21/08/2012	-2	78.931	11.365
ct92-M305Pete-12	120362	21/08/2012	-2	78.931	11.367
ct92-M305Pete-12	120362	21/08/2012	-2	78.929	11.357
ct92-M305Pete-12	120362	21/08/2012	-2	78.928	11.388
ct92-M305Pete-12	120362	21/08/2012	-2	78.928	11.387
ct92-M305Pete-12	120362	21/08/2012	-2	78.922	11.408
ct92-M305Pete-12	120362	21/08/2012	0	78.895	11.366
ct92-M305Pete-12	120362	21/08/2012	-2	78.918	11.376
ct92-M305Pete-12	120362	21/08/2012	-2	78.919	11.377
ct92-M305Pete-12	120362	21/08/2012	-2	78.918	11.368
ct92-M305Pete-12	120362	21/08/2012	-2	78.929	11.352
ct92-M305Pete-12	120362	21/08/2012	-2	78.926	11.275
ct92-M305Pete-12	120362	21/08/2012	-2	78.895	11.115
ct92-M305Pete-12	120362	21/08/2012	-1	78.895	11.138
ct92-M305Pete-12	120362	21/08/2012	-1	78.893	11.082
ct92-M305Pete-12	120362	21/08/2012	-2	78.906	11.074
ct92-M305Pete-12	120362	21/08/2012	-2	78.905	11.07
ct92-M305Pete-12	120362	21/08/2012	-2	78.894	11.097
ct92-M305Pete-12	120362	21/08/2012	0	78.895	11.09
ct92-M305Pete-12	120362	21/08/2012	2	78.889	11.048
ct92-M305Pete-12	120362	21/08/2012	-2	78.892	11.056
ct92-M305Pete-12	120362	21/08/2012	-2	78.892	11.061
ct92-M305Pete-12	120362	21/08/2012	-2	78.892	11.07
ct92-M305Pete-12	120362	21/08/2012	-2	78.886	11.048
ct92-M305Pete-12	120362	21/08/2012	-2	78.886	11.061
ct92-M305Pete-12	120362	21/08/2012	-2	78.888	11.071
ct92-M305Pete-12	120362	21/08/2012	-2	78.888	11.033
ct92-M305Pete-12	120362	21/08/2012	-1	78.887	11.058
ct92-M305Pete-12	120362	21/08/2012	1	78.885	11.018
ct92-M305Pete-12	120362	21/08/2012	-2	78.894	10.98
ct92-M305Pete-12	120362	21/08/2012	-2	78.886	11.015
ct92-M305Pete-12	120362	21/08/2012	-2	78.886	11.021
ct92-M305Pete-12	120362	21/08/2012	-2	78.884	10.994
ct92-M305Pete-12	120362	22/08/2012	-1	78.893	11.029
ct92-M305Pete-12	120362	22/08/2012	-2	78.889	11.084
ct92-M305Pete-12	120362	22/08/2012	-2	78.889	11.08
ct92-M305Pete-12	120362	22/08/2012	-2	78.892	10.984
ct92-M305Pete-12	120362	22/08/2012	-2	78.891	10.967
ct92-M305Pete-12	120362	22/08/2012	-2	78.885	11.005
ct92-M305Pete-12	120362	22/08/2012	-1	78.881	11.076
ct92-M305Pete-12	120362	22/08/2012	-2	78.882	11.074
ct92-M305Pete-12	120362	22/08/2012	-1	78.892	11.088
ct92-M305Pete-12	120362	22/08/2012	1	78.889	11.058
ct92-M305Pete-12	120362	22/08/2012	-2	78.896	11.044
ct92-M305Pete-12	120362	22/08/2012	-2	78.905	10.999
ct92-M305Pete-12	120362	22/08/2012	-2	78.889	10.944
ct92-M305Pete-12	120362	22/08/2012	-2	78.906	10.974
ct92-M305Pete-12	120362	22/08/2012	-2	78.917	10.826
ct92-M305Pete-12	120362	22/08/2012	-1	78.911	10.814
ct92-M305Pete-12	120362	22/08/2012	-1	78.913	10.804
ct92-M305Pete-12	120362	22/08/2012	-2	78.913	10.803
ct92-M305Pete-12	120362	22/08/2012	-2	78.89	10.848
ct92-M305Pete-12	120362	22/08/2012	-2	78.913	10.804
ct92-M305Pete-12	120362	22/08/2012	0	78.917	10.777
ct92-M305Pete-12	120362	22/08/2012	-2	78.916	10.773
ct92-M305Pete-12	120362	22/08/2012	0	78.933	10.637
ct92-M305Pete-12	120362	22/08/2012	1	78.921	10.667
ct92-M305Pete-12	120362	22/08/2012	1	78.919	10.661
ct92-M305Pete-12	120362	22/08/2012	1	78.921	10.664
ct92-M305Pete-12	120362	22/08/2012	-2	78.931	10.597
ct92-M305Pete-12	120362	22/08/2012	-2	78.907	10.66
ct92-M305Pete-12	120362	22/08/2012	-2	78.908	10.659
ct92-M305Pete-12	120362	22/08/2012	-2	78.91	10.645
ct92-M305Pete-12	120362	22/08/2012	-2	78.916	10.672

ct92-M305Pete-12	120362	22/08/2012	-2	78.922	10.681
ct92-M305Pete-12	120362	22/08/2012	-2	78.91	10.648
ct92-M305Pete-12	120362	22/08/2012	1	78.941	10.707
ct92-M305Pete-12	120362	22/08/2012	-2	78.939	10.706
ct92-M305Pete-12	120362	22/08/2012	-2	78.942	10.69
ct92-M305Pete-12	120362	22/08/2012	-2	78.909	10.736
ct92-M305Pete-12	120362	22/08/2012	-2	78.909	10.735
ct92-M305Pete-12	120362	22/08/2012	-2	78.932	10.666
ct92-M305Pete-12	120362	22/08/2012	-2	78.908	10.727
ct92-M305Pete-12	120362	22/08/2012	-1	78.913	10.728
ct92-M305Pete-12	120362	22/08/2012	-2	78.912	10.709
ct92-M305Pete-12	120362	22/08/2012	-2	78.909	10.722
ct92-M305Pete-12	120362	22/08/2012	-2	78.91	10.71
ct92-M305Pete-12	120362	22/08/2012	-2	78.911	10.707
ct92-M305Pete-12	120362	22/08/2012	-2	78.911	10.707
ct92-M305Pete-12	120362	22/08/2012	-2	78.909	10.708
ct92-M305Pete-12	120362	22/08/2012	-2	78.909	10.714
ct92-M305Pete-12	120362	22/08/2012	-2	78.908	10.716
ct92-M305Pete-12	120362	22/08/2012	-2	78.906	10.72
ct92-M305Pete-12	120362	22/08/2012	-2	78.909	10.695
ct92-M305Pete-12	120362	22/08/2012	-2	78.913	10.595
ct92-M305Pete-12	120362	22/08/2012	-2	78.916	10.728
ct92-M305Pete-12	120362	22/08/2012	-2	78.913	10.594
ct92-M305Pete-12	120362	22/08/2012	-2	78.926	10.555
ct92-M305Pete-12	120362	22/08/2012	-2	78.927	10.56
ct92-M305Pete-12	120362	22/08/2012	-2	78.947	10.667
ct92-M305Pete-12	120362	22/08/2012	-2	78.938	10.712
ct92-M305Pete-12	120362	22/08/2012	-1	78.911	10.675
ct92-M305Pete-12	120362	22/08/2012	-2	78.925	10.711
ct92-M305Pete-12	120362	23/08/2012	-2	78.928	10.742
ct92-M305Pete-12	120362	23/08/2012	-2	78.913	10.811
ct92-M305Pete-12	120362	23/08/2012	-2	78.91	10.809
ct92-M305Pete-12	120362	23/08/2012	-2	78.904	10.727
ct92-M305Pete-12	120362	23/08/2012	-2	78.908	10.795
ct92-M305Pete-12	120362	23/08/2012	-2	78.906	10.82
ct92-M305Pete-12	120362	23/08/2012	-2	78.908	10.805
ct92-M305Pete-12	120362	23/08/2012	-2	78.909	10.816
ct92-M305Pete-12	120362	23/08/2012	-2	78.91	10.807
ct92-M305Pete-12	120362	23/08/2012	-2	78.922	10.818
ct92-M305Pete-12	120362	23/08/2012	-2	78.942	10.72
ct92-M305Pete-12	120362	23/08/2012	-2	78.925	10.902
ct92-M305Pete-12	120362	23/08/2012	-1	78.939	10.718
ct92-M305Pete-12	120362	23/08/2012	-2	78.941	10.739
ct92-M305Pete-12	120362	23/08/2012	-1	78.942	10.744
ct92-M305Pete-12	120362	23/08/2012	-1	78.93	10.781
ct92-M305Pete-12	120362	23/08/2012	0	78.934	10.801
ct92-M305Pete-12	120362	23/08/2012	-2	78.937	10.838
ct92-M305Pete-12	120362	23/08/2012	-2	78.923	10.846
ct92-M305Pete-12	120362	23/08/2012	1	78.918	10.882
ct92-M305Pete-12	120362	23/08/2012	-1	78.915	10.866
ct92-M305Pete-12	120362	23/08/2012	-2	78.919	10.895
ct92-M305Pete-12	120362	23/08/2012	-2	78.922	10.856
ct92-M305Pete-12	120362	23/08/2012	-2	78.923	10.878
ct92-M305Pete-12	120362	23/08/2012	-2	78.924	10.856
ct92-M305Pete-12	120362	23/08/2012	-2	78.937	10.766
ct92-M305Pete-12	120362	23/08/2012	-1	78.94	10.659
ct92-M305Pete-12	120362	23/08/2012	-1	78.909	10.649
ct92-M305Pete-12	120362	23/08/2012	-2	78.932	10.563
ct92-M305Pete-12	120362	23/08/2012	-2	78.929	10.593
ct92-M305Pete-12	120362	23/08/2012	1	78.939	10.625
ct92-M305Pete-12	120362	23/08/2012	0	78.927	10.647
ct92-M305Pete-12	120362	23/08/2012	-1	78.924	10.638
ct92-M305Pete-12	120362	23/08/2012	-2	78.923	10.634
ct92-M305Pete-12	120362	23/08/2012	-2	78.922	10.62
ct92-M305Pete-12	120362	23/08/2012	-2	78.923	10.68
ct92-M305Pete-12	120362	23/08/2012	3	78.924	10.715
ct92-M305Pete-12	120362	23/08/2012	2	78.926	10.695
ct92-M305Pete-12	120362	23/08/2012	2	78.921	10.694

ct92-M305Pete-12	120362	23/08/2012	-2	78.922	10.724
ct92-M305Pete-12	120362	23/08/2012	-2	78.923	10.716
ct92-M305Pete-12	120362	23/08/2012	-2	78.912	10.773
ct92-M305Pete-12	120362	23/08/2012	-2	78.91	10.771
ct92-M305Pete-12	120362	23/08/2012	-2	78.91	10.763
ct92-M305Pete-12	120362	23/08/2012	-2	78.912	10.756
ct92-M305Pete-12	120362	23/08/2012	-2	78.914	10.754
ct92-M305Pete-12	120362	23/08/2012	-2	78.911	10.783
ct92-M305Pete-12	120362	23/08/2012	-2	78.911	10.771
ct92-M305Pete-12	120362	23/08/2012	-2	78.911	10.772
ct92-M305Pete-12	120362	23/08/2012	-1	78.91	10.815
ct92-M305Pete-12	120362	23/08/2012	-2	78.91	10.804
ct92-M305Pete-12	120362	23/08/2012	-2	78.934	10.737
ct92-M305Pete-12	120362	23/08/2012	-2	78.908	10.817
ct92-M305Pete-12	120362	23/08/2012	-2	78.914	10.806
ct92-M305Pete-12	120362	23/08/2012	-1	78.903	10.783
ct92-M305Pete-12	120362	23/08/2012	-2	78.903	10.813
ct92-M305Pete-12	120362	23/08/2012	-2	78.903	10.811
ct92-M305Pete-12	120362	23/08/2012	-2	78.908	10.765
ct92-M305Pete-12	120362	23/08/2012	-2	78.91	10.775
ct92-M305Pete-12	120362	23/08/2012	-2	78.91	10.819
ct92-M305Pete-12	120362	24/08/2012	-1	78.904	10.863
ct92-M305Pete-12	120362	24/08/2012	-2	78.914	10.844
ct92-M305Pete-12	120362	24/08/2012	-2	78.906	10.879
ct92-M305Pete-12	120362	24/08/2012	-2	78.923	10.755
ct92-M305Pete-12	120362	24/08/2012	-1	78.904	10.831
ct92-M305Pete-12	120362	24/08/2012	-2	78.905	10.849
ct92-M305Pete-12	120362	24/08/2012	-2	78.907	10.877
ct92-M305Pete-12	120362	24/08/2012	-1	78.892	10.896
ct92-M305Pete-12	120362	24/08/2012	-2	78.893	10.904
ct92-M305Pete-12	120362	24/08/2012	-2	78.892	10.903
ct92-M305Pete-12	120362	24/08/2012	-2	78.894	10.896
ct92-M305Pete-12	120362	24/08/2012	-2	78.903	10.912
ct92-M305Pete-12	120362	24/08/2012	-1	78.894	10.923
ct92-M305Pete-12	120362	24/08/2012	-2	78.904	10.841
ct92-M305Pete-12	120362	24/08/2012	-2	78.901	10.858
ct92-M305Pete-12	120362	24/08/2012	-2	78.901	10.858
ct92-M305Pete-12	120362	24/08/2012	-2	78.928	10.775
ct92-M305Pete-12	120362	24/08/2012	-2	78.939	10.875
ct92-M305Pete-12	120362	24/08/2012	-2	78.933	10.814
ct92-M305Pete-12	120362	24/08/2012	-1	78.912	10.847
ct92-M305Pete-12	120362	24/08/2012	-2	78.908	10.86
ct92-M305Pete-12	120362	24/08/2012	-2	78.909	10.874
ct92-M305Pete-12	120362	24/08/2012	-1	78.931	10.801
ct92-M305Pete-12	120362	24/08/2012	-2	78.912	10.86
ct92-M305Pete-12	120362	24/08/2012	-2	78.927	10.836
ct92-M305Pete-12	120362	24/08/2012	-2	78.868	10.88
ct92-M305Pete-12	120362	24/08/2012	-2	78.878	10.878
ct92-M305Pete-12	120362	24/08/2012	-1	78.95	10.764
ct92-M305Pete-12	120362	24/08/2012	-2	78.937	10.812
ct92-M305Pete-12	120362	24/08/2012	-1	78.926	10.787
ct92-M305Pete-12	120362	24/08/2012	-2	78.931	10.779
ct92-M305Pete-12	120362	24/08/2012	0	78.94	10.618
ct92-M305Pete-12	120362	24/08/2012	-2	78.955	10.591
ct92-M305Pete-12	120362	24/08/2012	-2	78.967	10.578
ct92-M305Pete-12	120362	24/08/2012	-1	78.94	10.638
ct92-M305Pete-12	120362	24/08/2012	-1	78.933	10.672
ct92-M305Pete-12	120362	24/08/2012	-2	78.933	10.664
ct92-M305Pete-12	120362	24/08/2012	-1	78.934	10.687
ct92-M305Pete-12	120362	24/08/2012	-1	78.924	10.69
ct92-M305Pete-12	120362	24/08/2012	-2	78.919	10.707
ct92-M305Pete-12	120362	24/08/2012	-1	78.93	10.676
ct92-M305Pete-12	120362	24/08/2012	-2	78.964	10.62
ct92-M305Pete-12	120362	24/08/2012	-2	78.954	10.637
ct92-M305Pete-12	120362	24/08/2012	-2	78.92	10.7
ct92-M305Pete-12	120362	24/08/2012	-2	78.93	10.688
ct92-M305Pete-12	120362	24/08/2012	-2	78.919	10.696
ct92-M305Pete-12	120362	24/08/2012	-2	78.926	10.794

ct92-M305Pete-12	120362	24/08/2012	-2	78.923	10.799
ct92-M305Pete-12	120362	24/08/2012	-2	78.934	10.786
ct92-M305Pete-12	120362	24/08/2012	-2	78.927	10.905
ct92-M305Pete-12	120362	24/08/2012	-1	78.912	10.871
ct92-M305Pete-12	120362	24/08/2012	-2	78.912	10.867
ct92-M305Pete-12	120362	24/08/2012	-2	78.897	10.861
ct92-M305Pete-12	120362	24/08/2012	-2	78.96	11.02
ct92-M305Pete-12	120362	24/08/2012	-1	78.887	11.069
ct92-M305Pete-12	120362	24/08/2012	-2	78.889	11.071
ct92-M305Pete-12	120362	24/08/2012	2	78.89	11.043
ct92-M305Pete-12	120362	25/08/2012	-2	78.898	11.084
ct92-M305Pete-12	120362	25/08/2012	-2	78.899	11.093
ct92-M305Pete-12	120362	25/08/2012	-2	78.902	11.126
ct92-M305Pete-12	120362	25/08/2012	-2	78.899	11.139
ct92-M305Pete-12	120362	25/08/2012	-1	78.887	11.106
ct92-M305Pete-12	120362	25/08/2012	-2	78.888	11.066
ct92-M305Pete-12	120362	25/08/2012	-2	78.891	11.061
ct92-M305Pete-12	120362	25/08/2012	-2	78.897	11.077
ct92-M305Pete-12	120362	25/08/2012	-1	78.889	11.234
ct92-M305Pete-12	120362	25/08/2012	0	78.89	11.175
ct92-M305Pete-12	120362	25/08/2012	-2	78.898	11.096
ct92-M305Pete-12	120362	25/08/2012	-2	78.903	11.132
ct92-M305Pete-12	120362	25/08/2012	-2	78.904	11.156
ct92-M305Pete-12	120362	25/08/2012	-2	78.892	11.112
ct92-M305Pete-12	120362	25/08/2012	2	78.896	11.211
ct92-M305Pete-12	120362	25/08/2012	1	78.903	11.224
ct92-M305Pete-12	120362	25/08/2012	-1	78.908	11.241
ct92-M305Pete-12	120362	25/08/2012	2	78.925	11.292
ct92-M305Pete-12	120362	25/08/2012	-2	78.925	11.312
ct92-M305Pete-12	120362	25/08/2012	-2	78.925	11.316
ct92-M305Pete-12	120362	25/08/2012	3	78.954	11.366
ct92-M305Pete-12	120362	25/08/2012	-2	78.953	11.363
ct92-M305Pete-12	120362	25/08/2012	-2	78.97	11.528
ct92-M305Pete-12	120362	25/08/2012	-2	78.965	11.483
ct92-M305Pete-12	120362	25/08/2012	-2	78.967	11.485
ct92-M305Pete-12	120362	25/08/2012	-2	78.971	11.57
ct92-M305Pete-12	120362	25/08/2012	-2	78.975	11.607
ct92-M305Pete-12	120362	25/08/2012	2	78.97	11.65
ct92-M305Pete-12	120362	25/08/2012	-2	78.97	11.653
ct92-M305Pete-12	120362	25/08/2012	-1	78.97	11.728
ct92-M305Pete-12	120362	25/08/2012	1	78.975	11.674
ct92-M305Pete-12	120362	25/08/2012	-1	78.959	11.696
ct92-M305Pete-12	120362	25/08/2012	-1	78.956	11.74
ct92-M305Pete-12	120362	25/08/2012	1	78.95	11.736
ct92-M305Pete-12	120362	25/08/2012	-1	78.986	11.752
ct92-M305Pete-12	120362	25/08/2012	-2	78.977	11.902
ct92-M305Pete-12	120362	25/08/2012	-2	78.972	11.873
ct92-M305Pete-12	120362	25/08/2012	-2	78.981	11.969
ct92-M305Pete-12	120362	25/08/2012	-2	78.99	12.146
ct92-M305Pete-12	120362	25/08/2012	-2	78.962	12.208
ct92-M305Pete-12	120362	25/08/2012	2	78.966	12.205
ct92-M305Pete-12	120362	25/08/2012	-2	78.97	12.3
ct92-M305Pete-12	120362	25/08/2012	3	78.972	12.359
ct92-M305Pete-12	120362	25/08/2012	3	78.975	12.354
ct92-M305Pete-12	120362	25/08/2012	-1	78.97	12.36
ct92-M305Pete-12	120362	25/08/2012	-2	79	12.839
ct92-M305Pete-12	120362	25/08/2012	1	78.968	12.449
ct92-M305Pete-12	120362	25/08/2012	3	78.976	12.442
ct92-M305Pete-12	120362	25/08/2012	-1	78.976	12.44
ct92-M305Pete-12	120362	25/08/2012	3	78.977	12.437
ct92-M305Pete-12	120362	26/08/2012	-1	78.989	12.668
ct92-M305Pete-12	120362	26/08/2012	-2	79.036	13.244
ct92-M305Pete-12	120362	26/08/2012	-2	79.038	13.339
ct92-M305Pete-12	120362	26/08/2012	-2	78.982	12.408
ct92-M305Pete-12	120362	26/08/2012	2	78.974	12.43
ct92-M305Pete-12	120362	26/08/2012	-2	79.001	12.139
ct92-M305Pete-12	120362	26/08/2012	-2	79.004	12.176
ct92-M305Pete-12	120362	26/08/2012	-2	79.006	12.208

ct92-M305Pete-12	120362	26/08/2012	-2	78.953	12.335
ct92-M305Pete-12	120362	26/08/2012	-2	78.952	12.328
ct92-M305Pete-12	120362	26/08/2012	-1	78.957	12.27
ct92-M305Pete-12	120362	26/08/2012	-2	78.949	12.193
ct92-M305Pete-12	120362	26/08/2012	-2	78.934	12.28
ct92-M305Pete-12	120362	26/08/2012	-1	78.93	12.239
ct92-M305Pete-12	120362	26/08/2012	-2	78.926	12.386
ct92-M305Pete-12	120362	26/08/2012	-2	78.929	12.36
ct92-M305Pete-12	120362	26/08/2012	0	78.936	12.305
ct92-M305Pete-12	120362	26/08/2012	-2	78.923	12.328
ct92-M305Pete-12	120362	26/08/2012	-1	78.921	12.345
ct92-M305Pete-12	120362	26/08/2012	-2	78.936	12.411
ct92-M305Pete-12	120362	26/08/2012	-2	78.931	12.3
ct92-M305Pete-12	120362	26/08/2012	-1	78.924	12.379
ct92-M305Pete-12	120362	26/08/2012	-2	78.924	12.412
ct92-M305Pete-12	120362	26/08/2012	-2	78.925	12.423
ct92-M305Pete-12	120362	26/08/2012	-2	78.916	12.416
ct92-M305Pete-12	120362	26/08/2012	-2	78.907	12.4
ct92-M305Pete-12	120362	26/08/2012	-2	78.903	12.407
ct92-M305Pete-12	120362	26/08/2012	-2	78.904	12.403
ct92-M305Pete-12	120362	26/08/2012	-2	78.897	12.423
ct92-M305Pete-12	120362	26/08/2012	-1	78.882	12.204
ct92-M305Pete-12	120362	26/08/2012	-1	78.902	12.119
ct92-M305Pete-12	120362	26/08/2012	-1	78.877	12.108
ct92-M305Pete-12	120362	26/08/2012	-2	78.914	11.981
ct92-M305Pete-12	120362	26/08/2012	-2	78.916	11.981
ct92-M305Pete-12	120362	26/08/2012	-2	78.903	12.035
ct92-M305Pete-12	120362	26/08/2012	-2	78.927	12.29
ct92-M305Pete-12	120362	26/08/2012	-1	78.894	11.933
ct92-M305Pete-12	120362	26/08/2012	-2	78.919	11.881
ct92-M305Pete-12	120362	26/08/2012	-2	78.929	11.879
ct92-M305Pete-12	120362	26/08/2012	-2	78.948	11.829
ct92-M305Pete-12	120362	26/08/2012	-1	78.938	11.803
ct92-M305Pete-12	120362	26/08/2012	-2	78.943	11.781
ct92-M305Pete-12	120362	26/08/2012	-1	78.946	11.756
ct92-M305Pete-12	120362	26/08/2012	-2	78.953	11.709
ct92-M305Pete-12	120362	26/08/2012	-1	78.951	11.701
ct92-M305Pete-12	120362	26/08/2012	-1	78.971	11.642
ct92-M305Pete-12	120362	26/08/2012	-2	78.971	11.649
ct92-M305Pete-12	120362	26/08/2012	2	78.967	11.642
ct92-M305Pete-12	120362	26/08/2012	-1	78.981	11.6
ct92-M305Pete-12	120362	26/08/2012	-2	78.99	11.632
ct92-M305Pete-12	120362	26/08/2012	-2	78.985	11.614
ct92-M305Pete-12	120362	26/08/2012	-1	78.983	11.67
ct92-M305Pete-12	120362	26/08/2012	-2	78.978	11.648
ct92-M305Pete-12	120362	26/08/2012	-1	78.986	11.635
ct92-M305Pete-12	120362	26/08/2012	-1	78.97	11.648
ct92-M305Pete-12	120362	26/08/2012	-1	78.975	11.657
ct92-M305Pete-12	120362	26/08/2012	-2	78.975	11.615
ct92-M305Pete-12	120362	26/08/2012	-2	78.974	11.638
ct92-M305Pete-12	120362	26/08/2012	-2	78.977	11.635
ct92-M305Pete-12	120362	26/08/2012	-2	78.978	11.636
ct92-M305Pete-12	120362	26/08/2012	-1	78.966	11.605
ct92-M305Pete-12	120362	26/08/2012	-1	78.973	11.665
ct92-M305Pete-12	120362	26/08/2012	-2	78.968	11.617
ct92-M305Pete-12	120362	26/08/2012	-2	78.966	11.62
ct92-M305Pete-12	120362	26/08/2012	-2	78.968	11.641
ct92-M305Pete-12	120362	26/08/2012	-2	78.967	11.619
ct92-M305Pete-12	120362	26/08/2012	-1	78.963	11.659
ct92-M305Pete-12	120362	26/08/2012	-1	78.97	11.71
ct92-M305Pete-12	120362	26/08/2012	-2	78.976	11.69
ct92-M305Pete-12	120362	27/08/2012	-2	78.982	11.65
ct92-M305Pete-12	120362	27/08/2012	-2	78.971	11.587
ct92-M305Pete-12	120362	27/08/2012	-2	78.978	11.635
ct92-M305Pete-12	120362	27/08/2012	-2	78.965	11.594
ct92-M305Pete-12	120362	27/08/2012	-2	78.97	11.639
ct92-M305Pete-12	120362	27/08/2012	-2	78.971	11.638
ct92-M305Pete-12	120362	27/08/2012	-1	78.964	11.681

ct92-M305Pete-12	120362	27/08/2012	-2	78.96	11.623
ct92-M305Pete-12	120362	27/08/2012	-2	78.963	11.612
ct92-M305Pete-12	120362	27/08/2012	-1	78.972	11.632
ct92-M305Pete-12	120362	27/08/2012	-2	78.972	11.632
ct92-M305Pete-12	120362	27/08/2012	0	78.965	11.608
ct92-M305Pete-12	120362	27/08/2012	-2	78.966	11.608
ct92-M305Pete-12	120362	27/08/2012	-2	78.966	11.594
ct92-M305Pete-12	120362	27/08/2012	-2	78.971	11.616
ct92-M305Pete-12	120362	27/08/2012	-2	78.97	11.617
ct92-M305Pete-12	120362	27/08/2012	-1	78.966	11.638
ct92-M305Pete-12	120362	27/08/2012	-2	78.967	11.637
ct92-M305Pete-12	120362	27/08/2012	-2	78.964	11.688
ct92-M305Pete-12	120362	27/08/2012	-2	78.963	11.673
ct92-M305Pete-12	120362	27/08/2012	-2	78.966	11.636
ct92-M305Pete-12	120362	27/08/2012	-1	78.959	11.594
ct92-M305Pete-12	120362	27/08/2012	-2	78.969	11.612
ct92-M305Pete-12	120362	27/08/2012	-2	78.958	11.593
ct92-M305Pete-12	120362	27/08/2012	-2	78.963	11.599
ct92-M305Pete-12	120362	27/08/2012	-2	78.96	11.64
ct92-M305Pete-12	120362	27/08/2012	-2	78.963	11.666
ct92-M305Pete-12	120362	27/08/2012	-2	78.958	11.635
ct92-M305Pete-12	120362	27/08/2012	-2	78.961	11.653
ct92-M305Pete-12	120362	27/08/2012	1	78.958	11.661
ct92-M305Pete-12	120362	27/08/2012	-2	78.958	11.675
ct92-M305Pete-12	120362	27/08/2012	-2	78.958	11.676
ct92-M305Pete-12	120362	27/08/2012	-2	78.962	11.646
ct92-M305Pete-12	120362	27/08/2012	-2	78.96	11.65
ct92-M305Pete-12	120362	27/08/2012	1	78.966	11.678
ct92-M305Pete-12	120362	27/08/2012	-2	78.951	11.702
ct92-M305Pete-12	120362	27/08/2012	-1	78.966	11.67
ct92-M305Pete-12	120362	27/08/2012	-2	78.964	11.689
ct92-M305Pete-12	120362	27/08/2012	-2	78.964	11.679
ct92-M305Pete-12	120362	27/08/2012	-2	78.963	11.692
ct92-M305Pete-12	120362	27/08/2012	-2	78.962	11.697
ct92-M305Pete-12	120362	27/08/2012	-2	78.971	11.683
ct92-M305Pete-12	120362	27/08/2012	-2	78.958	11.665
ct92-M305Pete-12	120362	27/08/2012	-2	78.963	11.634
ct92-M305Pete-12	120362	27/08/2012	-2	78.958	11.665
ct92-M305Pete-12	120362	27/08/2012	-2	78.973	11.71
ct92-M305Pete-12	120362	27/08/2012	-2	78.952	11.658
ct92-M305Pete-12	120362	27/08/2012	-2	78.964	11.679
ct92-M305Pete-12	120362	27/08/2012	-1	78.974	11.63
ct92-M305Pete-12	120362	27/08/2012	-2	78.978	11.652
ct92-M305Pete-12	120362	27/08/2012	-2	78.978	11.595
ct92-M305Pete-12	120362	27/08/2012	-2	78.972	11.525
ct92-M305Pete-12	120362	27/08/2012	-2	78.973	11.527
ct92-M305Pete-12	120362	27/08/2012	-2	78.972	11.467
ct92-M305Pete-12	120362	27/08/2012	-2	78.972	11.464
ct92-M305Pete-12	120362	27/08/2012	-2	78.976	11.444
ct92-M305Pete-12	120362	28/08/2012	-2	78.967	11.424
ct92-M305Pete-12	120362	28/08/2012	-2	78.968	11.421
ct92-M305Pete-12	120362	28/08/2012	-2	78.972	11.361
ct92-M305Pete-12	120362	28/08/2012	-2	78.971	11.343
ct92-M305Pete-12	120362	28/08/2012	-2	78.966	11.331
ct92-M305Pete-12	120362	28/08/2012	-2	78.958	11.281
ct92-M305Pete-12	120362	28/08/2012	-2	78.955	11.241
ct92-M305Pete-12	120362	28/08/2012	3	78.949	11.203
ct92-M305Pete-12	120362	28/08/2012	-2	78.948	11.206
ct92-M305Pete-12	120362	28/08/2012	-2	78.945	11.19
ct92-M305Pete-12	120362	28/08/2012	-2	78.939	11.111
ct92-M305Pete-12	120362	28/08/2012	-2	78.934	11.059
ct92-M305Pete-12	120362	28/08/2012	-2	78.929	11.087
ct92-M305Pete-12	120362	28/08/2012	-2	78.928	11.078
ct92-M305Pete-12	120362	28/08/2012	-2	78.938	11.055
ct92-M305Pete-12	120362	28/08/2012	-2	78.927	11.032
ct92-M305Pete-12	120362	28/08/2012	0	78.92	10.981
ct92-M305Pete-12	120362	28/08/2012	-2	78.915	10.922
ct92-M305Pete-12	120362	28/08/2012	-2	78.918	10.943

ct92-M305Pete-12	120362	28/08/2012	0	78.919	10.933
ct92-M305Pete-12	120362	28/08/2012	-2	78.91	10.934
ct92-M305Pete-12	120362	28/08/2012	-2	78.913	10.929
ct92-M305Pete-12	120362	28/08/2012	-1	78.923	10.827
ct92-M305Pete-12	120362	28/08/2012	-2	78.916	10.76
ct92-M305Pete-12	120362	28/08/2012	1	78.945	10.738
ct92-M305Pete-12	120362	28/08/2012	-2	78.938	10.711
ct92-M305Pete-12	120362	28/08/2012	1	78.922	10.708
ct92-M305Pete-12	120362	28/08/2012	-2	78.916	10.644
ct92-M305Pete-12	120362	28/08/2012	-2	78.915	10.692
ct92-M305Pete-12	120362	28/08/2012	-2	78.911	10.651
ct92-M305Pete-12	120362	28/08/2012	-2	78.912	10.66
ct92-M305Pete-12	120362	28/08/2012	-2	78.91	10.613
ct92-M305Pete-12	120362	28/08/2012	-2	78.925	10.654
ct92-M305Pete-12	120362	28/08/2012	-1	78.915	10.689
ct92-M305Pete-12	120362	28/08/2012	-1	78.92	10.65
ct92-M305Pete-12	120362	28/08/2012	-2	78.92	10.633
ct92-M305Pete-12	120362	28/08/2012	-2	78.919	10.629
ct92-M305Pete-12	120362	28/08/2012	1	78.929	10.6
ct92-M305Pete-12	120362	28/08/2012	-2	78.929	10.597
ct92-M305Pete-12	120362	28/08/2012	-2	78.92	10.613
ct92-M305Pete-12	120362	28/08/2012	-2	78.922	10.602
ct92-M305Pete-12	120362	28/08/2012	-1	78.924	10.699
ct92-M305Pete-12	120362	28/08/2012	-1	78.921	10.695
ct92-M305Pete-12	120362	28/08/2012	-2	78.919	10.689
ct92-M305Pete-12	120362	28/08/2012	-2	78.923	10.677
ct92-M305Pete-12	120362	28/08/2012	-1	78.908	10.621
ct92-M305Pete-12	120362	28/08/2012	-1	78.923	10.732
ct92-M305Pete-12	120362	28/08/2012	-2	78.918	10.772
ct92-M305Pete-12	120362	28/08/2012	-1	78.883	10.704
ct92-M305Pete-12	120362	28/08/2012	-2	78.914	10.808
ct92-M305Pete-12	120362	28/08/2012	-2	78.912	10.802
ct92-M305Pete-12	120362	28/08/2012	-1	78.909	10.836
ct92-M305Pete-12	120362	28/08/2012	-2	78.909	10.87
ct92-M305Pete-12	120362	28/08/2012	1	78.902	10.904
ct92-M305Pete-12	120362	28/08/2012	1	78.904	10.939
ct92-M305Pete-12	120362	28/08/2012	-2	78.903	10.943
ct92-M305Pete-12	120362	28/08/2012	-1	78.888	10.932
ct92-M305Pete-12	120362	28/08/2012	1	78.897	10.982
ct92-M305Pete-12	120362	28/08/2012	-2	78.903	11.017
ct92-M305Pete-12	120362	28/08/2012	-1	78.887	10.97
ct92-M305Pete-12	120362	28/08/2012	-1	78.889	11.114
ct92-M305Pete-12	120362	28/08/2012	2	78.883	11.067
ct92-M305Pete-12	120362	28/08/2012	-2	78.883	11.049
ct92-M305Pete-12	120362	28/08/2012	-1	78.891	11.071
ct92-M305Pete-12	120362	28/08/2012	-1	78.886	11.027
ct92-M305Pete-12	120362	28/08/2012	-2	78.884	11.012
ct92-M305Pete-12	120362	29/08/2012	-1	78.878	11.036
ct92-M305Pete-12	120362	29/08/2012	-2	78.877	11.023
ct92-M305Pete-12	120362	29/08/2012	-2	78.878	11.024
ct92-M305Pete-12	120362	29/08/2012	-2	78.877	11.031
ct92-M305Pete-12	120362	29/08/2012	-2	78.873	10.961
ct92-M305Pete-12	120362	29/08/2012	-2	78.859	10.68
ct92-M305Pete-12	120362	29/08/2012	-2	78.856	10.701
ct92-M305Pete-12	120362	29/08/2012	-2	78.852	10.697
ct92-M305Pete-12	120362	29/08/2012	-2	78.853	10.677
ct92-M305Pete-12	120362	29/08/2012	-2	78.924	10.806
ct92-M305Pete-12	120362	29/08/2012	-2	78.849	10.627
ct92-M305Pete-12	120362	29/08/2012	-2	78.849	10.649
ct92-M305Pete-12	120362	29/08/2012	-2	78.894	10.747
ct92-M305Pete-12	120362	29/08/2012	-2	78.897	10.757
ct92-M305Pete-12	120362	29/08/2012	-2	78.897	10.734
ct92-M305Pete-12	120362	29/08/2012	-2	78.898	10.734
ct92-M305Pete-12	120362	29/08/2012	-2	78.899	10.738
ct92-M305Pete-12	120362	29/08/2012	0	78.925	10.796
ct92-M305Pete-12	120362	29/08/2012	-1	78.9	10.729
ct92-M305Pete-12	120362	29/08/2012	-2	78.912	10.776
ct92-M305Pete-12	120362	29/08/2012	-2	78.916	10.78

ct92-M305Pete-12	120362	29/08/2012	-2	78.912	10.68
ct92-M305Pete-12	120362	29/08/2012	-2	78.912	10.683
ct92-M305Pete-12	120362	29/08/2012	-2	78.957	10.75
ct92-M305Pete-12	120362	29/08/2012	-2	78.93	10.643
ct92-M305Pete-12	120362	29/08/2012	-2	78.93	10.629
ct92-M305Pete-12	120362	29/08/2012	-2	78.926	10.645
ct92-M305Pete-12	120362	29/08/2012	-2	78.926	10.639
ct92-M305Pete-12	120362	29/08/2012	-2	78.925	10.64
ct92-M305Pete-12	120362	29/08/2012	-2	78.917	10.66
ct92-M305Pete-12	120362	29/08/2012	-1	78.913	10.646
ct92-M305Pete-12	120362	29/08/2012	-1	78.912	10.64
ct92-M305Pete-12	120362	29/08/2012	-2	78.92	10.611
ct92-M305Pete-12	120362	29/08/2012	-1	78.913	10.776
ct92-M305Pete-12	120362	29/08/2012	-2	78.918	10.619
ct92-M305Pete-12	120362	29/08/2012	-1	78.907	10.81
ct92-M305Pete-12	120362	29/08/2012	2	78.908	10.817
ct92-M305Pete-12	120362	29/08/2012	1	78.912	10.82
ct92-M305Pete-12	120362	29/08/2012	0	78.909	10.858
ct92-M305Pete-12	120362	29/08/2012	-2	78.904	10.889
ct92-M305Pete-12	120362	29/08/2012	2	78.902	10.884
ct92-M305Pete-12	120362	29/08/2012	-1	78.902	10.892
ct92-M305Pete-12	120362	29/08/2012	-2	78.901	10.889
ct92-M305Pete-12	120362	29/08/2012	2	78.905	10.897
ct92-M305Pete-12	120362	29/08/2012	-2	78.907	10.931
ct92-M305Pete-12	120362	29/08/2012	-2	78.889	10.973
ct92-M305Pete-12	120362	29/08/2012	-2	78.887	10.983
ct92-M305Pete-12	120362	29/08/2012	-2	78.905	10.836
ct92-M305Pete-12	120362	30/08/2012	-1	78.898	10.83
ct92-M305Pete-12	120362	30/08/2012	-1	78.909	10.777
ct92-M305Pete-12	120362	30/08/2012	-2	78.91	10.908
ct92-M305Pete-12	120362	30/08/2012	2	78.915	10.906
ct92-M305Pete-12	120362	30/08/2012	-1	78.91	10.951
ct92-M305Pete-12	120362	30/08/2012	-2	78.919	10.937
ct92-M305Pete-12	120362	30/08/2012	-2	78.897	10.888
ct92-M305Pete-12	120362	30/08/2012	-2	78.912	10.959
ct92-M305Pete-12	120362	30/08/2012	-2	78.912	10.931
ct92-M305Pete-12	120362	30/08/2012	0	78.911	10.764
ct92-M305Pete-12	120362	30/08/2012	-2	78.91	10.787
ct92-M305Pete-12	120362	30/08/2012	0	78.905	10.785
ct92-M305Pete-12	120362	30/08/2012	-1	78.9	10.788
ct92-M305Pete-12	120362	30/08/2012	-1	78.9	10.785
ct92-M305Pete-12	120362	30/08/2012	-2	78.905	10.815
ct92-M305Pete-12	120362	30/08/2012	1	78.905	10.769
ct92-M305Pete-12	120362	30/08/2012	-2	78.898	10.701
ct92-M305Pete-12	120362	30/08/2012	-2	78.901	10.752
ct92-M305Pete-12	120362	30/08/2012	-2	78.901	10.752
ct92-M305Pete-12	120362	30/08/2012	-2	78.905	10.687
ct92-M305Pete-12	120362	30/08/2012	-2	78.906	10.724
ct92-M305Pete-12	120362	30/08/2012	-2	78.908	10.694
ct92-M305Pete-12	120362	30/08/2012	-2	78.904	10.685
ct92-M305Pete-12	120362	30/08/2012	-2	78.905	10.651
ct92-M305Pete-12	120362	30/08/2012	-2	78.899	10.681
ct92-M305Pete-12	120362	30/08/2012	-2	78.894	10.639
ct92-M305Pete-12	120362	30/08/2012	-2	78.892	10.618
ct92-M305Pete-12	120362	30/08/2012	-2	78.89	10.624
ct92-M305Pete-12	120362	30/08/2012	-2	78.89	10.622
ct92-M305Pete-12	120362	30/08/2012	-2	78.908	10.726
ct92-M305Pete-12	120362	30/08/2012	-2	78.916	10.689
ct92-M305Pete-12	120362	30/08/2012	-2	78.916	10.697
ct92-M305Pete-12	120362	30/08/2012	-2	78.923	10.658
ct92-M305Pete-12	120362	30/08/2012	-2	78.913	10.713
ct92-M305Pete-12	120362	30/08/2012	0	78.929	10.697
ct92-M305Pete-12	120362	30/08/2012	-2	78.92	10.708
ct92-M305Pete-12	120362	30/08/2012	0	78.928	10.617
ct92-M305Pete-12	120362	30/08/2012	-2	78.92	10.669
ct92-M305Pete-12	120362	30/08/2012	-2	78.918	10.678
ct92-M305Pete-12	120362	30/08/2012	-2	78.92	10.689
ct92-M305Pete-12	120362	30/08/2012	-2	78.922	10.676

ct92-M305Pete-12	120362	30/08/2012	-2	78.918	10.586
ct92-M305Pete-12	120362	30/08/2012	-2	78.894	10.637
ct92-M305Pete-12	120362	30/08/2012	-2	78.9	10.521
ct92-M305Pete-12	120362	30/08/2012	-2	78.901	10.515
ct92-M305Pete-12	120362	30/08/2012	-2	78.898	10.516
ct92-M305Pete-12	120362	30/08/2012	-2	78.901	10.502
ct92-M305Pete-12	120362	30/08/2012	-2	78.888	10.529
ct92-M305Pete-12	120362	30/08/2012	-2	78.89	10.505
ct92-M305Pete-12	120362	30/08/2012	-2	78.9	10.593
ct92-M305Pete-12	120362	30/08/2012	-2	78.899	10.592
ct92-M305Pete-12	120362	30/08/2012	-1	78.915	10.633
ct92-M305Pete-12	120362	30/08/2012	-1	78.932	10.76
ct92-M305Pete-12	120362	30/08/2012	-2	78.935	10.642
ct92-M305Pete-12	120362	30/08/2012	-2	78.951	10.597
ct92-M305Pete-12	120362	31/08/2012	-2	78.953	10.608
ct92-M305Pete-12	120362	31/08/2012	2	78.948	10.684
ct92-M305Pete-12	120362	31/08/2012	-2	78.94	10.707
ct92-M305Pete-12	120362	31/08/2012	-2	78.925	10.708
ct92-M305Pete-12	120362	31/08/2012	-2	78.949	10.697
ct92-M305Pete-12	120362	31/08/2012	-2	78.941	10.693
ct92-M305Pete-12	120362	31/08/2012	-2	78.94	10.735
ct92-M305Pete-12	120362	31/08/2012	-2	78.922	10.708
ct92-M305Pete-12	120362	31/08/2012	-2	78.939	10.723
ct92-M305Pete-12	120362	31/08/2012	-1	78.941	10.676
ct92-M305Pete-12	120362	31/08/2012	-1	78.92	10.61
ct92-M305Pete-12	120362	31/08/2012	0	78.914	10.567
ct92-M305Pete-12	120362	31/08/2012	-1	78.913	10.592
ct92-M305Pete-12	120362	31/08/2012	-2	78.914	10.589
ct92-M305Pete-12	120362	31/08/2012	-2	78.913	10.59
ct92-M305Pete-12	120362	31/08/2012	-1	78.91	10.571
ct92-M305Pete-12	120362	31/08/2012	-2	78.911	10.6
ct92-M305Pete-12	120362	31/08/2012	-2	78.907	10.579
ct92-M305Pete-12	120362	31/08/2012	-1	78.908	10.599
ct92-M305Pete-12	120362	31/08/2012	-2	78.903	10.551
ct92-M305Pete-12	120362	31/08/2012	-1	78.899	10.549
ct92-M305Pete-12	120362	31/08/2012	-2	78.908	10.548
ct92-M305Pete-12	120362	31/08/2012	-2	78.905	10.565
ct92-M305Pete-12	120362	31/08/2012	-1	78.898	10.519
ct92-M305Pete-12	120362	31/08/2012	-2	78.894	10.523
ct92-M305Pete-12	120362	31/08/2012	-2	78.903	10.595
ct92-M305Pete-12	120362	31/08/2012	-2	78.898	10.579
ct92-M305Pete-12	120362	31/08/2012	-2	78.898	10.579
ct92-M305Pete-12	120362	31/08/2012	-2	78.909	10.637
ct92-M305Pete-12	120362	31/08/2012	-2	78.912	10.559
ct92-M305Pete-12	120362	31/08/2012	-2	78.913	10.557
ct92-M305Pete-12	120362	31/08/2012	-2	78.913	10.56
ct92-M305Pete-12	120362	31/08/2012	-2	78.924	10.552
ct92-M305Pete-12	120362	31/08/2012	-2	78.921	10.657
ct92-M305Pete-12	120362	31/08/2012	-2	78.921	10.538
ct92-M305Pete-12	120362	31/08/2012	-2	78.921	10.542
ct92-M305Pete-12	120362	31/08/2012	-2	78.919	10.655
ct92-M305Pete-12	120362	31/08/2012	-2	78.929	10.565
ct92-M305Pete-12	120362	31/08/2012	-2	78.916	10.622
ct92-M305Pete-12	120362	31/08/2012	-2	78.927	10.613
ct92-M305Pete-12	120362	31/08/2012	-2	78.921	10.655
ct92-M305Pete-12	120362	31/08/2012	-2	78.928	10.644
ct92-M305Pete-12	120362	31/08/2012	-2	78.932	10.649
ct92-M305Pete-12	120362	31/08/2012	-2	78.933	10.624
ct92-M305Pete-12	120362	31/08/2012	-2	78.939	10.567
ct92-M305Pete-12	120362	31/08/2012	-2	78.929	10.625
ct92-M305Pete-12	120362	01/09/2012	-2	78.932	10.628
ct92-M305Pete-12	120362	01/09/2012	-2	78.932	10.633
ct92-M305Pete-12	120362	01/09/2012	-2	78.935	10.634
ct92-M305Pete-12	120362	01/09/2012	-2	78.944	10.695
ct92-M305Pete-12	120362	01/09/2012	-2	78.935	10.719
ct92-M305Pete-12	120362	01/09/2012	-2	78.937	10.74
ct92-M305Pete-12	120362	01/09/2012	-2	78.935	10.72
ct92-M305Pete-12	120362	01/09/2012	-2	78.936	10.724

ct92-M305Pete-12	120362	01/09/2012	-2	78.939	10.741
ct92-M305Pete-12	120362	01/09/2012	1	78.938	10.706
ct92-M305Pete-12	120362	01/09/2012	-2	78.935	10.68
ct92-M305Pete-12	120362	01/09/2012	-1	78.934	10.699
ct92-M305Pete-12	120362	01/09/2012	-2	78.934	10.698
ct92-M305Pete-12	120362	01/09/2012	-2	78.923	10.66
ct92-M305Pete-12	120362	01/09/2012	-2	78.922	10.671
ct92-M305Pete-12	120362	01/09/2012	-2	78.924	10.652
ct92-M305Pete-12	120362	01/09/2012	-2	78.922	10.687
ct92-M305Pete-12	120362	01/09/2012	-2	78.929	10.655
ct92-M305Pete-12	120362	01/09/2012	-1	78.927	10.627
ct92-M305Pete-12	120362	01/09/2012	-1	78.93	10.658
ct92-M305Pete-12	120362	01/09/2012	-2	78.93	10.648
ct92-M305Pete-12	120362	01/09/2012	1	78.933	10.712
ct92-M305Pete-12	120362	01/09/2012	-2	78.935	10.701
ct92-M305Pete-12	120362	01/09/2012	-2	78.932	10.706
ct92-M305Pete-12	120362	01/09/2012	-2	78.936	10.668
ct92-M305Pete-12	120362	01/09/2012	-1	78.94	10.598
ct92-M305Pete-12	120362	01/09/2012	-1	78.916	10.673
ct92-M305Pete-12	120362	01/09/2012	-2	78.914	10.674
ct92-M305Pete-12	120362	01/09/2012	-1	78.911	10.68
ct92-M305Pete-12	120362	01/09/2012	-1	78.913	10.637
ct92-M305Pete-12	120362	01/09/2012	-2	78.91	10.679
ct92-M305Pete-12	120362	01/09/2012	1	78.913	10.647
ct92-M305Pete-12	120362	01/09/2012	1	78.918	10.666
ct92-M305Pete-12	120362	01/09/2012	-1	78.91	10.662
ct92-M305Pete-12	120362	01/09/2012	-2	78.901	10.691
ct92-M305Pete-12	120362	01/09/2012	-2	78.914	10.639
ct92-M305Pete-12	120362	01/09/2012	-2	78.915	10.642
ct92-M305Pete-12	120362	01/09/2012	-2	78.918	10.666
ct92-M305Pete-12	120362	01/09/2012	-2	78.92	10.653
ct92-M305Pete-12	120362	01/09/2012	-1	78.907	10.606
ct92-M305Pete-12	120362	01/09/2012	1	78.909	10.657
ct92-M305Pete-12	120362	01/09/2012	-1	78.91	10.661
ct92-M305Pete-12	120362	01/09/2012	-2	78.909	10.669
ct92-M305Pete-12	120362	01/09/2012	-2	78.909	10.663
ct92-M305Pete-12	120362	01/09/2012	-2	78.914	10.666
ct92-M305Pete-12	120362	01/09/2012	-2	78.912	10.665
ct92-M305Pete-12	120362	01/09/2012	-2	78.912	10.67
ct92-M305Pete-12	120362	01/09/2012	-1	78.913	10.545
ct92-M305Pete-12	120362	01/09/2012	-2	78.914	10.539
ct92-M305Pete-12	120362	01/09/2012	-2	78.917	10.514
ct92-M305Pete-12	120362	01/09/2012	-2	78.913	10.672
ct92-M305Pete-12	120362	01/09/2012	-2	78.914	10.673
ct92-M305Pete-12	120362	01/09/2012	-2	78.932	10.628
ct92-M305Pete-12	120362	02/09/2012	-2	78.934	10.589
ct92-M305Pete-12	120362	02/09/2012	-2	78.932	10.633
ct92-M305Pete-12	120362	02/09/2012	-2	78.929	10.631
ct92-M305Pete-12	120362	02/09/2012	-2	78.932	10.633
ct92-M305Pete-12	120362	02/09/2012	-2	78.925	10.667
ct92-M305Pete-12	120362	02/09/2012	-2	78.929	10.597
ct92-M305Pete-12	120362	02/09/2012	-2	78.93	10.647
ct92-M305Pete-12	120362	02/09/2012	-1	78.941	10.514
ct92-M305Pete-12	120362	02/09/2012	-1	78.93	10.621
ct92-M305Pete-12	120362	02/09/2012	-2	78.92	10.676
ct92-M305Pete-12	120362	02/09/2012	-1	78.927	10.658
ct92-M305Pete-12	120362	02/09/2012	-2	78.939	10.64
ct92-M305Pete-12	120362	02/09/2012	-2	78.941	10.565
ct92-M305Pete-12	120362	02/09/2012	-2	78.939	10.642
ct92-M305Pete-12	120362	02/09/2012	-2	78.927	10.654
ct92-M305Pete-12	120362	02/09/2012	-2	78.923	10.655
ct92-M305Pete-12	120362	02/09/2012	-1	78.917	10.607
ct92-M305Pete-12	120362	02/09/2012	-2	78.903	10.646
ct92-M305Pete-12	120362	02/09/2012	-2	78.91	10.634
ct92-M305Pete-12	120362	02/09/2012	0	78.937	10.588
ct92-M305Pete-12	120362	02/09/2012	0	78.954	10.657
ct92-M305Pete-12	120362	02/09/2012	-1	78.937	10.638
ct92-M305Pete-12	120362	02/09/2012	-2	78.934	10.658

ct92-M305Pete-12	120362	02/09/2012	-2	78.935	10.75
ct92-M305Pete-12	120362	02/09/2012	-1	78.921	10.697
ct92-M305Pete-12	120362	02/09/2012	-1	78.962	10.455
ct92-M305Pete-12	120362	02/09/2012	-2	78.921	10.73
ct92-M305Pete-12	120362	02/09/2012	1	78.937	10.719
ct92-M305Pete-12	120362	02/09/2012	-2	78.932	10.755
ct92-M305Pete-12	120362	02/09/2012	-2	78.908	10.729
ct92-M305Pete-12	120362	02/09/2012	-2	78.907	10.731
ct92-M305Pete-12	120362	02/09/2012	-2	78.9	10.655
ct92-M305Pete-12	120362	02/09/2012	-2	78.849	10.978
ct92-M305Pete-12	120362	02/09/2012	-2	78.87	10.716
ct92-M305Pete-12	120362	02/09/2012	-2	78.876	10.705
ct92-M305Pete-12	120362	02/09/2012	-2	78.891	10.706
ct92-M305Pete-12	120362	02/09/2012	-2	78.939	10.826
ct92-M305Pete-12	120362	02/09/2012	-2	78.909	10.861
ct92-M305Pete-12	120362	02/09/2012	-2	78.914	10.803
ct92-M305Pete-12	120362	02/09/2012	-2	78.915	10.804
ct92-M305Pete-12	120362	02/09/2012	-2	78.917	10.786
ct92-M305Pete-12	120362	02/09/2012	-2	78.934	10.812
ct92-M305Pete-12	120362	02/09/2012	-2	78.936	10.788
ct92-M305Pete-12	120362	02/09/2012	-2	78.936	10.789
ct92-M305Pete-12	120362	02/09/2012	-2	78.938	10.675
ct92-M305Pete-12	120362	02/09/2012	-2	78.922	10.665
ct92-M305Pete-12	120362	02/09/2012	-2	78.933	10.603
ct92-M305Pete-12	120362	02/09/2012	-1	78.945	10.617
ct92-M305Pete-12	120362	02/09/2012	-2	78.943	10.628
ct92-M305Pete-12	120362	02/09/2012	-2	78.944	10.574
ct92-M305Pete-12	120362	02/09/2012	-1	78.944	10.622
ct92-M305Pete-12	120362	02/09/2012	-2	78.931	10.636
ct92-M305Pete-12	120362	02/09/2012	-2	78.928	10.613
ct92-M305Pete-12	120362	03/09/2012	-2	78.938	10.576
ct92-M305Pete-12	120362	03/09/2012	-2	78.936	10.595
ct92-M305Pete-12	120362	03/09/2012	-2	78.937	10.582
ct92-M305Pete-12	120362	03/09/2012	-2	78.942	10.546
ct92-M305Pete-12	120362	03/09/2012	-2	78.944	10.519
ct92-M305Pete-12	120362	03/09/2012	-2	78.941	10.452
ct92-M305Pete-12	120362	03/09/2012	-2	78.95	10.359
ct92-M305Pete-12	120362	03/09/2012	-2	78.949	10.576
ct92-M305Pete-12	120362	03/09/2012	-2	78.94	10.621
ct92-M305Pete-12	120362	03/09/2012	-2	78.937	10.63
ct92-M305Pete-12	120362	03/09/2012	1	78.916	10.691
ct92-M305Pete-12	120362	03/09/2012	-2	78.931	10.701
ct92-M305Pete-12	120362	03/09/2012	-2	78.949	10.597
ct92-M305Pete-12	120362	03/09/2012	-1	78.925	10.706
ct92-M305Pete-12	120362	03/09/2012	-2	78.916	10.657
ct92-M305Pete-12	120362	03/09/2012	-1	78.92	10.693
ct92-M305Pete-12	120362	03/09/2012	-2	78.921	10.692
ct92-M305Pete-12	120362	03/09/2012	-2	78.924	10.688
ct92-M305Pete-12	120362	03/09/2012	-1	78.919	10.74
ct92-M305Pete-12	120362	03/09/2012	-2	78.92	10.706
ct92-M305Pete-12	120362	03/09/2012	-2	78.925	10.616
ct92-M305Pete-12	120362	03/09/2012	-2	78.92	10.703
ct92-M305Pete-12	120362	03/09/2012	-2	78.909	10.642
ct92-M305Pete-12	120362	03/09/2012	-2	78.911	10.588
ct92-M305Pete-12	120362	03/09/2012	-2	78.914	10.591
ct92-M305Pete-12	120362	03/09/2012	-1	78.911	10.551
ct92-M305Pete-12	120362	03/09/2012	-2	78.911	10.547
ct92-M305Pete-12	120362	03/09/2012	-2	78.911	10.545
ct92-M305Pete-12	120362	03/09/2012	-2	78.908	10.525
ct92-M305Pete-12	120362	03/09/2012	-1	78.901	10.588
ct92-M305Pete-12	120362	03/09/2012	-2	78.893	10.524
ct92-M305Pete-12	120362	03/09/2012	-2	78.898	10.558
ct92-M305Pete-12	120362	03/09/2012	-2	78.894	10.551
ct92-M305Pete-12	120362	03/09/2012	-2	78.914	10.563
ct92-M305Pete-12	120362	03/09/2012	-2	78.909	10.529
ct92-M305Pete-12	120362	03/09/2012	-2	78.899	10.49
ct92-M305Pete-12	120362	03/09/2012	-2	78.912	10.586
ct92-M305Pete-12	120362	03/09/2012	-2	78.911	10.567

ct92-M305Pete-12	120362	03/09/2012	-2	78.928	10.584
ct92-M305Pete-12	120362	03/09/2012	-2	78.928	10.626
ct92-M305Pete-12	120362	03/09/2012	-1	78.951	10.7
ct92-M305Pete-12	120362	03/09/2012	-2	78.934	10.688
ct92-M305Pete-12	120362	03/09/2012	-1	78.94	10.626
ct92-M305Pete-12	120362	03/09/2012	-2	78.936	10.622
ct92-M305Pete-12	120362	03/09/2012	-2	78.937	10.622
ct92-M305Pete-12	120362	03/09/2012	-2	78.937	10.622
ct92-M305Pete-12	120362	03/09/2012	-2	78.94	10.644
ct92-M305Pete-12	120362	03/09/2012	-2	78.936	10.643
ct92-M305Pete-12	120362	04/09/2012	-2	78.957	10.725
ct92-M305Pete-12	120362	04/09/2012	-2	78.953	10.733
ct92-M305Pete-12	120362	04/09/2012	-1	78.928	10.706
ct92-M305Pete-12	120362	04/09/2012	-2	78.929	10.734
ct92-M305Pete-12	120362	04/09/2012	-1	78.939	10.698
ct92-M305Pete-12	120362	04/09/2012	-2	78.947	10.707
ct92-M305Pete-12	120362	04/09/2012	-2	78.939	10.736
ct92-M305Pete-12	120362	04/09/2012	-2	78.946	10.783
ct92-M305Pete-12	120362	04/09/2012	-1	78.94	10.8
ct92-M305Pete-12	120362	04/09/2012	-2	78.922	10.758
ct92-M305Pete-12	120362	04/09/2012	-1	78.925	10.849
ct92-M305Pete-12	120362	04/09/2012	-2	78.94	10.801
ct92-M305Pete-12	120362	04/09/2012	1	78.897	10.941
ct92-M305Pete-12	120362	04/09/2012	-1	78.896	10.912
ct92-M305Pete-12	120362	04/09/2012	-1	78.899	10.955
ct92-M305Pete-12	120362	04/09/2012	-2	78.898	11.006
ct92-M305Pete-12	120362	04/09/2012	0	78.898	10.974
ct92-M305Pete-12	120362	04/09/2012	-1	78.897	11.074
ct92-M305Pete-12	120362	04/09/2012	1	78.896	11.063
ct92-M305Pete-12	120362	04/09/2012	-2	78.894	11.084
ct92-M305Pete-12	120362	04/09/2012	-2	78.898	11.127
ct92-M305Pete-12	120362	04/09/2012	-2	78.89	11.12
ct92-M305Pete-12	120362	04/09/2012	2	78.901	11.155
ct92-M305Pete-12	120362	04/09/2012	-2	78.9	11.198
ct92-M305Pete-12	120362	04/09/2012	-1	78.954	11.37
ct92-M305Pete-12	120362	04/09/2012	-1	78.961	11.3
ct92-M305Pete-12	120362	04/09/2012	-2	78.962	11.321
ct92-M305Pete-12	120362	04/09/2012	-2	78.962	11.314
ct92-M305Pete-12	120362	04/09/2012	-2	78.961	11.334
ct92-M305Pete-12	120362	04/09/2012	-2	78.973	11.344
ct92-M305Pete-12	120362	04/09/2012	-2	78.968	11.398
ct92-M305Pete-12	120362	04/09/2012	-2	78.972	11.453
ct92-M305Pete-12	120362	04/09/2012	-2	78.993	11.643
ct92-M305Pete-12	120362	04/09/2012	1	78.961	11.646
ct92-M305Pete-12	120362	04/09/2012	3	78.968	11.625
ct92-M305Pete-12	120362	04/09/2012	1	78.962	11.63
ct92-M305Pete-12	120362	04/09/2012	2	78.961	11.801
ct92-M305Pete-12	120362	04/09/2012	-1	78.949	11.835
ct92-M305Pete-12	120362	04/09/2012	-2	78.948	11.896
ct92-M305Pete-12	120362	04/09/2012	-2	78.95	11.892
ct92-M305Pete-12	120362	04/09/2012	2	78.948	11.944
ct92-M305Pete-12	120362	04/09/2012	1	78.948	12.019
ct92-M305Pete-12	120362	04/09/2012	1	78.952	12.142
ct92-M305Pete-12	120362	04/09/2012	2	78.964	12.225
ct92-M305Pete-12	120362	04/09/2012	3	78.961	12.208
ct92-M305Pete-12	120362	04/09/2012	0	78.96	12.195
ct92-M305Pete-12	120362	04/09/2012	-1	78.963	12.161
ct92-M305Pete-12	120362	04/09/2012	1	78.966	12.191
ct92-M305Pete-12	120362	04/09/2012	-2	78.965	12.181
ct92-M305Pete-12	120362	05/09/2012	1	78.956	12.23
ct92-M305Pete-12	120362	05/09/2012	1	78.953	12.167
ct92-M305Pete-12	120362	05/09/2012	1	78.947	12.205
ct92-M305Pete-12	120362	05/09/2012	-2	78.957	12.291
ct92-M305Pete-12	120362	05/09/2012	1	78.955	12.226
ct92-M305Pete-12	120362	05/09/2012	2	78.968	12.286
ct92-M305Pete-12	120362	05/09/2012	2	78.97	12.436
ct92-M305Pete-12	120362	05/09/2012	1	78.964	12.433
ct92-M305Pete-12	120362	05/09/2012	3	78.965	12.421

ct92-M305Pete-12	120362	05/09/2012	2	78.965	12.421
ct92-M305Pete-12	120362	05/09/2012	3	78.967	12.419
ct92-M305Pete-12	120362	05/09/2012	3	78.977	12.317
ct92-M305Pete-12	120362	05/09/2012	1	78.982	12.423
ct92-M305Pete-12	120362	05/09/2012	2	78.974	12.37
ct92-M305Pete-12	120362	05/09/2012	-2	78.954	12.31
ct92-M305Pete-12	120362	05/09/2012	1	78.982	12.332
ct92-M305Pete-12	120362	05/09/2012	3	78.98	12.314
ct92-M305Pete-12	120362	05/09/2012	3	78.98	12.334
ct92-M305Pete-12	120362	05/09/2012	3	78.983	12.318
ct92-M305Pete-12	120362	05/09/2012	-1	78.982	12.323
ct92-M305Pete-12	120362	05/09/2012	2	78.977	12.342
ct92-M305Pete-12	120362	05/09/2012	2	78.979	12.344
ct92-M305Pete-12	120362	05/09/2012	3	78.976	12.303
ct92-M305Pete-12	120362	05/09/2012	-2	78.977	12.318
ct92-M305Pete-12	120362	06/09/2012	3	78.976	12.314
ct92-M305Pete-12	120362	06/09/2012	-2	78.978	12.318
ct92-M305Pete-12	120362	06/09/2012	-2	78.974	12.324
ct92-M305Pete-12	120362	06/09/2012	1	78.97	12.314
ct92-M305Pete-12	120362	06/09/2012	-1	78.958	12.184
ct92-M305Pete-12	120362	06/09/2012	0	78.97	12.335
ct92-M305Pete-12	120362	06/09/2012	1	78.956	12.394
ct92-M305Pete-12	120362	06/09/2012	-1	78.964	12.301
ct92-M305Pete-12	120362	06/09/2012	1	78.966	12.294
ct92-M305Pete-12	120362	06/09/2012	3	78.986	12.326
ct92-M305Pete-12	120362	06/09/2012	2	78.987	12.336
ct92-M305Pete-12	120362	06/09/2012	1	78.982	12.385
ct92-M305Pete-12	120362	06/09/2012	2	78.979	12.341
ct92-M305Pete-12	120362	06/09/2012	-2	78.979	12.274
ct92-M305Pete-12	120362	06/09/2012	-2	78.972	12.305
ct92-M305Pete-12	120362	06/09/2012	1	78.972	12.358
ct92-M305Pete-12	120362	07/09/2012	-2	78.965	12.408
ct92-M305Pete-12	120362	07/09/2012	3	78.972	12.353
ct92-M305Pete-12	120362	07/09/2012	1	78.968	12.357
ct92-M305Pete-12	120362	07/09/2012	-2	78.955	12.389
ct92-M305Pete-12	120362	07/09/2012	2	78.975	12.361
ct92-M305Pete-12	120362	07/09/2012	0	78.937	12.399
ct92-M305Pete-12	120362	07/09/2012	2	78.954	12.324
ct92-M305Pete-12	120362	07/09/2012	1	78.977	12.316
ct92-M305Pete-12	120362	07/09/2012	-2	78.976	12.305
ct92-M305Pete-12	120362	07/09/2012	-2	78.976	12.308
ct92-M305Pete-12	120362	07/09/2012	1	78.978	12.316
ct92-M305Pete-12	120362	07/09/2012	-2	78.977	12.308
ct92-M305Pete-12	120362	07/09/2012	-2	78.986	12.305
ct92-M305Pete-12	120362	07/09/2012	1	78.96	12.392
ct92-M305Pete-12	120362	07/09/2012	2	78.982	12.301
ct92-M305Pete-12	120362	07/09/2012	3	78.982	12.3
ct92-M305Pete-12	120362	07/09/2012	3	78.982	12.306
ct92-M305Pete-12	120362	07/09/2012	2	78.98	12.287
ct92-M305Pete-12	120362	07/09/2012	-2	78.977	12.284
ct92-M305Pete-12	120362	07/09/2012	-2	78.994	12.302
ct92-M305Pete-12	120362	07/09/2012	1	78.977	12.338
ct92-M305Pete-12	120362	07/09/2012	-2	78.982	12.276
ct92-M305Pete-12	120362	07/09/2012	-2	78.982	12.273
ct92-M305Pete-12	120362	07/09/2012	-2	78.972	12.217
ct92-M305Pete-12	120362	07/09/2012	-2	78.969	12.2
ct92-M305Pete-12	120362	07/09/2012	-2	78.957	12.073
ct92-M305Pete-12	120362	07/09/2012	-2	78.957	12.071
ct92-M305Pete-12	120362	07/09/2012	-2	78.947	12.027
ct92-M305Pete-12	120362	07/09/2012	-2	78.947	12.026
ct92-M305Pete-12	120362	07/09/2012	-2	78.949	12.026
ct92-M305Pete-12	120362	07/09/2012	-2	78.951	12.011
ct92-M305Pete-12	120362	07/09/2012	-2	78.952	12.009
ct92-M305Pete-12	120362	07/09/2012	-2	78.953	12.004
ct92-M305Pete-12	120362	07/09/2012	-2	78.953	12.001
ct92-M305Pete-12	120362	07/09/2012	-2	78.971	11.736
ct92-M305Pete-12	120362	07/09/2012	-2	78.971	11.735
ct92-M305Pete-12	120362	07/09/2012	-2	78.962	11.841

ct92-M305Pete-12	120362	07/09/2012	-2	78.963	11.84
ct92-M305Pete-12	120362	07/09/2012	-1	78.976	11.592
ct92-M305Pete-12	120362	07/09/2012	-2	78.975	11.602
ct92-M305Pete-12	120362	07/09/2012	-2	78.974	11.585
ct92-M305Pete-12	120362	07/09/2012	-2	78.969	11.638
ct92-M305Pete-12	120362	08/09/2012	-2	78.977	11.508
ct92-M305Pete-12	120362	08/09/2012	-1	78.966	11.493
ct92-M305Pete-12	120362	08/09/2012	-1	78.967	11.478
ct92-M305Pete-12	120362	08/09/2012	-2	78.967	11.476
ct92-M305Pete-12	120362	08/09/2012	-2	78.961	11.336
ct92-M305Pete-12	120362	08/09/2012	-1	78.962	11.377
ct92-M305Pete-12	120362	08/09/2012	-2	78.962	11.356
ct92-M305Pete-12	120362	08/09/2012	-1	78.962	11.357
ct92-M305Pete-12	120362	08/09/2012	2	78.979	11.386
ct92-M305Pete-12	120362	08/09/2012	-2	78.975	11.408
ct92-M305Pete-12	120362	08/09/2012	-2	78.968	11.364
ct92-M305Pete-12	120362	08/09/2012	-1	78.99	11.394
ct92-M305Pete-12	120362	08/09/2012	-2	78.997	11.392
ct92-M305Pete-12	120362	08/09/2012	-2	78.997	11.388
ct92-M305Pete-12	120362	08/09/2012	-2	78.999	11.368
ct92-M305Pete-12	120362	08/09/2012	-1	78.994	11.322
ct92-M305Pete-12	120362	08/09/2012	-2	79.005	11.341
ct92-M305Pete-12	120362	08/09/2012	-2	78.994	11.302
ct92-M305Pete-12	120362	08/09/2012	-2	78.993	11.299
ct92-M305Pete-12	120362	08/09/2012	-2	78.98	11.323
ct92-M305Pete-12	120362	08/09/2012	-2	79.001	11.348
ct92-M305Pete-12	120362	08/09/2012	-2	78.998	11.336
ct92-M305Pete-12	120362	08/09/2012	0	78.98	11.274
ct92-M305Pete-12	120362	08/09/2012	-2	79.008	11.339
ct92-M305Pete-12	120362	08/09/2012	-2	79.006	11.179
ct92-M305Pete-12	120362	08/09/2012	3	78.978	11.307
ct92-M305Pete-12	120362	08/09/2012	-2	78.994	11.404
ct92-M305Pete-12	120362	08/09/2012	-2	79.004	11.211
ct92-M305Pete-12	120362	08/09/2012	-2	78.975	11.357
ct92-M305Pete-12	120362	08/09/2012	-2	78.959	11.291
ct92-M305Pete-12	120362	08/09/2012	0	78.973	11.221
ct92-M305Pete-12	120362	08/09/2012	0	78.976	11.335
ct92-M305Pete-12	120362	08/09/2012	1	78.94	11.22
ct92-M305Pete-12	120362	08/09/2012	-2	78.939	11.21
ct92-M305Pete-12	120362	08/09/2012	2	78.922	11.162
ct92-M305Pete-12	120362	08/09/2012	0	78.926	11.203
ct92-M305Pete-12	120362	08/09/2012	1	78.935	11.186
ct92-M305Pete-12	120362	08/09/2012	-2	78.935	11.186
ct92-M305Pete-12	120362	08/09/2012	-1	78.906	11.012
ct92-M305Pete-12	120362	08/09/2012	-2	78.911	11.007
ct92-M305Pete-12	120362	08/09/2012	-2	78.918	11.06
ct92-M305Pete-12	120362	08/09/2012	-2	78.899	10.984
ct92-M305Pete-12	120362	08/09/2012	-2	78.884	10.917
ct92-M305Pete-12	120362	08/09/2012	-2	78.891	10.953
ct92-M305Pete-12	120362	08/09/2012	-2	78.882	10.912
ct92-M305Pete-12	120362	08/09/2012	-2	78.881	10.85
ct92-M305Pete-12	120362	08/09/2012	-2	78.883	10.86
ct92-M305Pete-12	120362	08/09/2012	-2	78.884	10.842
ct92-M305Pete-12	120362	08/09/2012	-2	78.91	10.902
ct92-M305Pete-12	120362	08/09/2012	-2	78.901	10.926
ct92-M305Pete-12	120362	08/09/2012	-2	78.897	10.924
ct92-M305Pete-12	120362	08/09/2012	-2	78.903	10.949
ct92-M305Pete-12	120362	08/09/2012	-2	78.901	10.94
ct92-M305Pete-12	120362	08/09/2012	-2	78.908	10.791
ct92-M305Pete-12	120362	08/09/2012	-2	78.902	10.784
ct92-M305Pete-12	120362	08/09/2012	-2	78.904	10.799
ct92-M305Pete-12	120362	08/09/2012	-2	78.906	10.877
ct92-M305Pete-12	120362	08/09/2012	-2	78.914	10.909
ct92-M305Pete-12	120362	08/09/2012	-2	78.915	10.906
ct92-M305Pete-12	120362	08/09/2012	-2	78.916	10.903
ct92-M305Pete-12	120362	08/09/2012	-2	78.917	10.856
ct92-M305Pete-12	120362	08/09/2012	-2	78.915	10.851
ct92-M305Pete-12	120362	08/09/2012	-2	78.917	10.84

ct92-M305Pete-12	120362	08/09/2012	-2	78.917	10.842
ct92-M305Pete-12	120362	08/09/2012	-2	78.916	10.844
ct92-M305Pete-12	120362	08/09/2012	-2	78.913	10.815
ct92-M305Pete-12	120362	08/09/2012	-2	78.913	10.818
ct92-M305Pete-12	120362	09/09/2012	-1	78.898	10.748
ct92-M305Pete-12	120362	09/09/2012	-1	78.914	10.784
ct92-M305Pete-12	120362	09/09/2012	2	78.909	10.761
ct92-M305Pete-12	120362	09/09/2012	-1	78.923	10.755
ct92-M305Pete-12	120362	09/09/2012	-1	78.904	10.77
ct92-M305Pete-12	120362	09/09/2012	-2	78.877	10.863
ct92-M305Pete-12	120362	09/09/2012	-2	78.915	10.806
ct92-M305Pete-12	120362	09/09/2012	-2	78.918	10.795
ct92-M305Pete-12	120362	09/09/2012	1	78.909	10.807
ct92-M305Pete-12	120362	09/09/2012	2	78.907	10.786
ct92-M305Pete-12	120362	09/09/2012	-1	78.908	10.79
ct92-M305Pete-12	120362	09/09/2012	-2	78.906	10.796
ct92-M305Pete-12	120362	09/09/2012	-1	78.908	10.803
ct92-M305Pete-12	120362	09/09/2012	-2	78.908	10.79
ct92-M305Pete-12	120362	09/09/2012	1	78.906	10.783
ct92-M305Pete-12	120362	09/09/2012	-2	78.909	10.783
ct92-M305Pete-12	120362	09/09/2012	-2	78.909	10.797
ct92-M305Pete-12	120362	09/09/2012	-1	78.919	10.793
ct92-M305Pete-12	120362	09/09/2012	-2	78.905	10.758
ct92-M305Pete-12	120362	09/09/2012	-2	78.92	10.778
ct92-M305Pete-12	120362	09/09/2012	-2	78.896	10.765
ct92-M305Pete-12	120362	09/09/2012	-2	78.898	10.778
ct92-M305Pete-12	120362	09/09/2012	-1	78.898	10.797
ct92-M305Pete-12	120362	09/09/2012	-1	78.905	10.837
ct92-M305Pete-12	120362	09/09/2012	-2	78.898	10.803
ct92-M305Pete-12	120362	09/09/2012	-2	78.902	10.804
ct92-M305Pete-12	120362	09/09/2012	-2	78.903	10.806
ct92-M305Pete-12	120362	09/09/2012	-2	78.902	10.816
ct92-M305Pete-12	120362	09/09/2012	-2	78.903	10.82
ct92-M305Pete-12	120362	09/09/2012	-2	78.904	10.833
ct92-M305Pete-12	120362	09/09/2012	-2	78.904	10.832
ct92-M305Pete-12	120362	09/09/2012	-2	78.906	10.845
ct92-M305Pete-12	120362	09/09/2012	-2	78.905	10.842
ct92-M305Pete-12	120362	09/09/2012	-2	78.904	10.838
ct92-M305Pete-12	120362	09/09/2012	-2	78.907	10.857
ct92-M305Pete-12	120362	09/09/2012	-2	78.907	10.84
ct92-M305Pete-12	120362	09/09/2012	-2	78.909	10.847
ct92-M305Pete-12	120362	09/09/2012	-2	78.909	10.856
ct92-M305Pete-12	120362	09/09/2012	-2	78.94	10.913
ct92-M305Pete-12	120362	09/09/2012	-1	78.92	10.831
ct92-M305Pete-12	120362	09/09/2012	-2	78.931	10.912
ct92-M305Pete-12	120362	09/09/2012	1	78.926	10.819
ct92-M305Pete-12	120362	09/09/2012	-2	78.927	10.824
ct92-M305Pete-12	120362	09/09/2012	-2	78.927	10.828
ct92-M305Pete-12	120362	09/09/2012	-1	78.919	10.779
ct92-M305Pete-12	120362	09/09/2012	-2	78.918	10.777
ct92-M305Pete-12	120362	09/09/2012	-2	78.923	10.807
ct92-M305Pete-12	120362	09/09/2012	-2	78.925	10.815
ct92-M305Pete-12	120362	09/09/2012	-2	78.926	10.818
ct92-M305Pete-12	120362	09/09/2012	-1	78.92	10.777
ct92-M305Pete-12	120362	09/09/2012	-1	78.91	10.81
ct92-M305Pete-12	120362	09/09/2012	-2	78.928	10.832
ct92-M305Pete-12	120362	09/09/2012	-2	78.938	10.829
ct92-M305Pete-12	120362	10/09/2012	-2	78.93	10.735
ct92-M305Pete-12	120362	10/09/2012	-2	78.94	10.68
ct92-M305Pete-12	120362	10/09/2012	-1	78.934	10.771
ct92-M305Pete-12	120362	10/09/2012	-2	78.931	10.766
ct92-M305Pete-12	120362	10/09/2012	-2	78.933	10.792
ct92-M305Pete-12	120362	10/09/2012	2	78.94	10.701
ct92-M305Pete-12	120362	10/09/2012	-2	78.944	10.682
ct92-M305Pete-12	120362	10/09/2012	-2	78.942	10.683
ct92-M305Pete-12	120362	10/09/2012	-2	78.952	10.675
ct92-M305Pete-12	120362	10/09/2012	-2	78.956	10.621
ct92-M305Pete-12	120362	10/09/2012	-2	78.958	10.586

ct92-M305Pete-12	120362	10/09/2012	-2	78.957	10.601
ct92-M305Pete-12	120362	10/09/2012	-1	78.955	10.605
ct92-M305Pete-12	120362	10/09/2012	-2	78.958	10.602
ct92-M305Pete-12	120362	10/09/2012	-2	78.958	10.601
ct92-M305Pete-12	120362	10/09/2012	-2	78.926	10.605
ct92-M305Pete-12	120362	10/09/2012	-1	78.944	10.625
ct92-M305Pete-12	120362	10/09/2012	-1	78.928	10.647
ct92-M305Pete-12	120362	10/09/2012	-1	78.932	10.685
ct92-M305Pete-12	120362	10/09/2012	-2	78.933	10.685
ct92-M305Pete-12	120362	10/09/2012	-1	78.933	10.664
ct92-M305Pete-12	120362	10/09/2012	1	78.923	10.654
ct92-M305Pete-12	120362	10/09/2012	-2	78.933	10.685
ct92-M305Pete-12	120362	10/09/2012	-2	78.934	10.671
ct92-M305Pete-12	120362	10/09/2012	-2	78.928	10.613
ct92-M305Pete-12	120362	10/09/2012	-2	78.927	10.6
ct92-M305Pete-12	120362	10/09/2012	-1	78.933	10.585
ct92-M305Pete-12	120362	10/09/2012	-2	78.931	10.579
ct92-M305Pete-12	120362	10/09/2012	-2	78.932	10.691
ct92-M305Pete-12	120362	10/09/2012	-2	78.914	10.686
ct92-M305Pete-12	120362	10/09/2012	-1	78.908	10.718
ct92-M305Pete-12	120362	10/09/2012	-2	78.927	10.694
ct92-M305Pete-12	120362	10/09/2012	-2	78.927	10.699
ct92-M305Pete-12	120362	10/09/2012	-2	78.924	10.728
ct92-M305Pete-12	120362	10/09/2012	-2	78.924	10.735
ct92-M305Pete-12	120362	10/09/2012	-2	78.924	10.728
ct92-M305Pete-12	120362	10/09/2012	1	78.923	10.791
ct92-M305Pete-12	120362	10/09/2012	1	78.945	10.747
ct92-M305Pete-12	120362	10/09/2012	1	78.911	10.691
ct92-M305Pete-12	120362	10/09/2012	-2	78.913	10.697
ct92-M305Pete-12	120362	10/09/2012	-1	78.923	10.721
ct92-M305Pete-12	120362	10/09/2012	-1	78.948	10.878
ct92-M305Pete-12	120362	10/09/2012	-2	78.925	10.7
ct92-M305Pete-12	120362	10/09/2012	-2	78.924	10.703
ct92-M305Pete-12	120362	10/09/2012	-1	78.927	10.688
ct92-M305Pete-12	120362	10/09/2012	-2	78.928	10.699
ct92-M305Pete-12	120362	10/09/2012	-2	78.929	10.712
ct92-M305Pete-12	120362	10/09/2012	-2	78.927	10.705
ct92-M305Pete-12	120362	10/09/2012	-2	78.925	10.71
ct92-M305Pete-12	120362	10/09/2012	-1	78.952	10.877
ct92-M305Pete-12	120362	10/09/2012	-1	78.923	10.728
ct92-M305Pete-12	120362	10/09/2012	-2	78.928	10.809
ct92-M305Pete-12	120362	10/09/2012	-2	78.93	10.793
ct92-M305Pete-12	120362	10/09/2012	-2	78.913	10.744
ct92-M305Pete-12	120362	10/09/2012	-2	78.929	10.788
ct92-M305Pete-12	120362	10/09/2012	-2	78.923	10.715
ct92-M305Pete-12	120362	10/09/2012	-2	78.936	10.73
ct92-M305Pete-12	120362	10/09/2012	1	78.94	10.724
ct92-M305Pete-12	120362	10/09/2012	2	78.941	10.731
ct92-M305Pete-12	120362	10/09/2012	-1	78.936	10.686
ct92-M305Pete-12	120362	10/09/2012	-2	78.939	10.703
ct92-M305Pete-12	120362	11/09/2012	-2	78.936	10.733
ct92-M305Pete-12	120362	11/09/2012	-2	78.937	10.782
ct92-M305Pete-12	120362	11/09/2012	1	78.944	10.684
ct92-M305Pete-12	120362	11/09/2012	-2	78.943	10.683
ct92-M305Pete-12	120362	11/09/2012	1	78.944	10.639
ct92-M305Pete-12	120362	11/09/2012	2	78.931	10.664
ct92-M305Pete-12	120362	11/09/2012	-2	78.928	10.682
ct92-M305Pete-12	120362	11/09/2012	-2	78.927	10.672
ct92-M305Pete-12	120362	11/09/2012	-2	78.928	10.679
ct92-M305Pete-12	120362	11/09/2012	-1	78.921	10.731
ct92-M305Pete-12	120362	11/09/2012	-2	78.91	10.761
ct92-M305Pete-12	120362	11/09/2012	-2	78.911	10.76
ct92-M305Pete-12	120362	11/09/2012	-2	78.913	10.796
ct92-M305Pete-12	120362	11/09/2012	-2	78.906	10.83
ct92-M305Pete-12	120362	11/09/2012	-1	78.901	10.747
ct92-M305Pete-12	120362	11/09/2012	-2	78.914	10.788
ct92-M305Pete-12	120362	11/09/2012	-2	78.91	10.74
ct92-M305Pete-12	120362	11/09/2012	-2	78.901	10.748

ct92-M305Pete-12	120362	11/09/2012	-2	78.901	10.762
ct92-M305Pete-12	120362	11/09/2012	-2	78.909	10.831
ct92-M305Pete-12	120362	11/09/2012	-2	78.912	10.786
ct92-M305Pete-12	120362	11/09/2012	-2	78.909	10.777
ct92-M305Pete-12	120362	11/09/2012	-2	78.909	10.756
ct92-M305Pete-12	120362	11/09/2012	-2	78.908	10.753
ct92-M305Pete-12	120362	11/09/2012	-2	78.904	10.802
ct92-M305Pete-12	120362	11/09/2012	-2	78.906	10.801
ct92-M305Pete-12	120362	11/09/2012	-2	78.91	10.775
ct92-M305Pete-12	120362	11/09/2012	-2	78.896	10.725
ct92-M305Pete-12	120362	11/09/2012	-2	78.91	10.775
ct92-M305Pete-12	120362	11/09/2012	-2	78.91	10.768
ct92-M305Pete-12	120362	11/09/2012	-2	78.905	10.771
ct92-M305Pete-12	120362	11/09/2012	-2	78.907	10.79
ct92-M305Pete-12	120362	11/09/2012	-2	78.905	10.787
ct92-M305Pete-12	120362	11/09/2012	-2	78.905	10.772
ct92-M305Pete-12	120362	11/09/2012	-2	78.904	10.769
ct92-M305Pete-12	120362	11/09/2012	-2	78.916	10.746
ct92-M305Pete-12	120362	11/09/2012	-2	78.921	10.671
ct92-M305Pete-12	120362	11/09/2012	-2	78.925	10.676
ct92-M305Pete-12	120362	11/09/2012	-2	78.929	10.663
ct92-M305Pete-12	120362	11/09/2012	-2	78.933	10.65
ct92-M305Pete-12	120362	11/09/2012	-2	78.932	10.622
ct92-M305Pete-12	120362	11/09/2012	-2	78.932	10.714
ct92-M305Pete-12	120362	11/09/2012	-2	78.924	10.725
ct92-M305Pete-12	120362	11/09/2012	-2	78.92	10.659
ct92-M305Pete-12	120362	11/09/2012	-2	78.944	10.76
ct92-M305Pete-12	120362	11/09/2012	-2	78.941	10.748
ct92-M305Pete-12	120362	11/09/2012	-2	78.918	10.665
ct92-M305Pete-12	120362	11/09/2012	-2	78.909	10.758
ct92-M305Pete-12	120362	11/09/2012	-1	78.937	10.752
ct92-M305Pete-12	120362	11/09/2012	-2	78.939	10.751
ct92-M305Pete-12	120362	11/09/2012	-2	78.936	10.738
ct92-M305Pete-12	120362	11/09/2012	-2	78.935	10.751
ct92-M305Pete-12	120362	11/09/2012	-1	78.926	10.75
ct92-M305Pete-12	120362	11/09/2012	-2	78.927	10.73
ct92-M305Pete-12	120362	11/09/2012	-1	78.929	10.695
ct92-M305Pete-12	120362	11/09/2012	-2	78.933	10.744
ct92-M305Pete-12	120362	11/09/2012	-2	78.929	10.692
ct92-M305Pete-12	120362	11/09/2012	-1	78.938	10.679
ct92-M305Pete-12	120362	11/09/2012	-2	78.956	10.686
ct92-M305Pete-12	120362	11/09/2012	-2	78.956	10.679
ct92-M305Pete-12	120362	11/09/2012	-2	78.956	10.685
ct92-M305Pete-12	120362	12/09/2012	-2	78.955	10.671
ct92-M305Pete-12	120362	12/09/2012	-2	78.956	10.636
ct92-M305Pete-12	120362	12/09/2012	-2	78.952	10.638
ct92-M305Pete-12	120362	12/09/2012	1	78.928	10.782
ct92-M305Pete-12	120362	12/09/2012	1	78.949	10.608
ct92-M305Pete-12	120362	12/09/2012	-2	78.963	10.739
ct92-M305Pete-12	120362	12/09/2012	-2	78.933	10.736
ct92-M305Pete-12	120362	12/09/2012	-1	78.928	10.687
ct92-M305Pete-12	120362	12/09/2012	1	78.925	10.664
ct92-M305Pete-12	120362	12/09/2012	-2	78.925	10.664
ct92-M305Pete-12	120362	12/09/2012	-2	78.925	10.66
ct92-M305Pete-12	120362	12/09/2012	3	78.919	10.643
ct92-M305Pete-12	120362	12/09/2012	-2	78.919	10.647
ct92-M305Pete-12	120362	12/09/2012	-2	78.918	10.629
ct92-M305Pete-12	120362	12/09/2012	-2	78.929	10.613
ct92-M305Pete-12	120362	12/09/2012	-2	78.92	10.628
ct92-M305Pete-12	120362	12/09/2012	-1	78.922	10.622
ct92-M305Pete-12	120362	12/09/2012	-2	78.922	10.611
ct92-M305Pete-12	120362	12/09/2012	1	78.919	10.666
ct92-M305Pete-12	120362	12/09/2012	-2	78.919	10.667
ct92-M305Pete-12	120362	12/09/2012	-1	78.939	10.698
ct92-M305Pete-12	120362	12/09/2012	1	78.94	10.765
ct92-M305Pete-12	120362	12/09/2012	-2	78.939	10.749
ct92-M305Pete-12	120362	12/09/2012	0	78.931	10.684
ct92-M305Pete-12	120362	12/09/2012	-2	78.921	10.612

ct92-M305Pete-12	120362	12/09/2012	-2	78.92	10.614
ct92-M305Pete-12	120362	12/09/2012	-2	78.913	10.616
ct92-M305Pete-12	120362	12/09/2012	-2	78.917	10.581
ct92-M305Pete-12	120362	12/09/2012	-2	78.918	10.597
ct92-M305Pete-12	120362	12/09/2012	-2	78.926	10.698
ct92-M305Pete-12	120362	12/09/2012	-1	78.919	10.687
ct92-M305Pete-12	120362	12/09/2012	-1	78.917	10.614
ct92-M305Pete-12	120362	12/09/2012	-2	78.917	10.611
ct92-M305Pete-12	120362	12/09/2012	-2	78.931	10.596
ct92-M305Pete-12	120362	12/09/2012	-2	78.929	10.599
ct92-M305Pete-12	120362	12/09/2012	-2	78.929	10.6
ct92-M305Pete-12	120362	12/09/2012	-2	78.93	10.623
ct92-M305Pete-12	120362	12/09/2012	-2	78.93	10.626
ct92-M305Pete-12	120362	12/09/2012	-1	78.928	10.645
ct92-M305Pete-12	120362	12/09/2012	-1	78.94	10.619
ct92-M305Pete-12	120362	12/09/2012	-2	78.947	10.717
ct92-M305Pete-12	120362	12/09/2012	-1	78.943	10.654
ct92-M305Pete-12	120362	12/09/2012	-2	78.943	10.655
ct92-M305Pete-12	120362	12/09/2012	1	78.945	10.677
ct92-M305Pete-12	120362	12/09/2012	-2	78.946	10.679
ct92-M305Pete-12	120362	12/09/2012	-2	78.941	10.765
ct92-M305Pete-12	120362	12/09/2012	-2	78.923	10.752
ct92-M305Pete-12	120362	13/09/2012	-2	78.923	10.75
ct92-M305Pete-12	120362	13/09/2012	-1	78.925	10.655
ct92-M305Pete-12	120362	13/09/2012	-2	78.925	10.654
ct92-M305Pete-12	120362	13/09/2012	-2	78.925	10.648
ct92-M305Pete-12	120362	13/09/2012	-2	78.925	10.649
ct92-M305Pete-12	120362	13/09/2012	-2	78.933	10.674
ct92-M305Pete-12	120362	13/09/2012	-1	78.919	10.654
ct92-M305Pete-12	120362	13/09/2012	1	78.916	10.809
ct92-M305Pete-12	120362	13/09/2012	-2	78.915	10.789
ct92-M305Pete-12	120362	13/09/2012	-1	78.918	10.801
ct92-M305Pete-12	120362	13/09/2012	-2	78.916	10.784
ct92-M305Pete-12	120362	13/09/2012	-1	78.909	10.81
ct92-M305Pete-12	120362	13/09/2012	2	78.912	10.809
ct92-M305Pete-12	120362	13/09/2012	0	78.902	10.852
ct92-M305Pete-12	120362	13/09/2012	1	78.928	10.786
ct92-M305Pete-12	120362	13/09/2012	-2	78.924	10.764
ct92-M305Pete-12	120362	13/09/2012	-2	78.918	10.779
ct92-M305Pete-12	120362	13/09/2012	-2	78.916	10.783
ct92-M305Pete-12	120362	13/09/2012	-2	78.922	10.752
ct92-M305Pete-12	120362	13/09/2012	-2	78.907	10.805
ct92-M305Pete-12	120362	13/09/2012	-2	78.908	10.783
ct92-M305Pete-12	120362	13/09/2012	0	78.888	10.781
ct92-M305Pete-12	120362	13/09/2012	-1	78.902	10.783
ct92-M305Pete-12	120362	13/09/2012	-2	78.907	10.773
ct92-M305Pete-12	120362	13/09/2012	-1	78.916	10.822
ct92-M305Pete-12	120362	13/09/2012	-2	78.908	10.801
ct92-M305Pete-12	120362	13/09/2012	-2	78.914	10.817
ct92-M305Pete-12	120362	13/09/2012	1	78.906	10.817
ct92-M305Pete-12	120362	13/09/2012	-1	78.908	10.879
ct92-M305Pete-12	120362	13/09/2012	-1	78.901	10.842
ct92-M305Pete-12	120362	13/09/2012	-1	78.901	10.823
ct92-M305Pete-12	120362	13/09/2012	-1	78.889	10.821
ct92-M305Pete-12	120362	13/09/2012	-2	78.891	10.821
ct92-M305Pete-12	120362	13/09/2012	-2	78.894	10.778
ct92-M305Pete-12	120362	13/09/2012	-2	78.89	10.737
ct92-M305Pete-12	120362	13/09/2012	-2	78.892	10.725
ct92-M305Pete-12	120362	13/09/2012	-2	78.88	10.841
ct92-M305Pete-12	120362	13/09/2012	-2	78.879	10.836
ct92-M305Pete-12	120362	13/09/2012	-2	78.882	10.817
ct92-M305Pete-12	120362	13/09/2012	-2	78.883	10.809
ct92-M305Pete-12	120362	13/09/2012	-2	78.882	10.811
ct92-M305Pete-12	120362	13/09/2012	-1	78.897	10.738
ct92-M305Pete-12	120362	13/09/2012	-2	78.899	10.768
ct92-M305Pete-12	120362	13/09/2012	-1	78.911	10.779
ct92-M305Pete-12	120362	13/09/2012	-2	78.922	10.812
ct92-M305Pete-12	120362	13/09/2012	-1	78.923	10.764

ct92-M305Pete-12	120362	13/09/2012	-1	78.922	10.718
ct92-M305Pete-12	120362	13/09/2012	-2	78.932	10.69
ct92-M305Pete-12	120362	13/09/2012	-2	78.915	10.651
ct92-M305Pete-12	120362	13/09/2012	-2	78.927	10.619
ct92-M305Pete-12	120362	13/09/2012	-2	78.926	10.618
ct92-M305Pete-12	120362	13/09/2012	-1	78.931	10.572
ct92-M305Pete-12	120362	13/09/2012	-2	78.926	10.62
ct92-M305Pete-12	120362	13/09/2012	-2	78.93	10.551
ct92-M305Pete-12	120362	14/09/2012	-2	78.927	10.561
ct92-M305Pete-12	120362	14/09/2012	-2	78.93	10.645
ct92-M305Pete-12	120362	14/09/2012	-2	78.932	10.524
ct92-M305Pete-12	120362	14/09/2012	-2	78.931	10.536
ct92-M305Pete-12	120362	14/09/2012	-2	78.929	10.563
ct92-M305Pete-12	120362	14/09/2012	-1	78.944	10.675
ct92-M305Pete-12	120362	14/09/2012	-1	78.942	10.624
ct92-M305Pete-12	120362	14/09/2012	-1	78.92	10.792
ct92-M305Pete-12	120362	14/09/2012	-2	78.952	10.739
ct92-M305Pete-12	120362	14/09/2012	-2	78.948	10.726
ct92-M305Pete-12	120362	14/09/2012	-2	78.944	10.769
ct92-M305Pete-12	120362	14/09/2012	-1	78.915	10.798
ct92-M305Pete-12	120362	14/09/2012	-1	78.91	10.815
ct92-M305Pete-12	120362	14/09/2012	-2	78.914	10.775
ct92-M305Pete-12	120362	14/09/2012	-2	78.915	10.832
ct92-M305Pete-12	120362	14/09/2012	-2	78.915	10.806
ct92-M305Pete-12	120362	14/09/2012	1	78.906	10.805
ct92-M305Pete-12	120362	14/09/2012	-1	78.911	10.766
ct92-M305Pete-12	120362	14/09/2012	-2	78.907	10.74
ct92-M305Pete-12	120362	14/09/2012	-2	78.908	10.725
ct92-M305Pete-12	120362	14/09/2012	-1	78.902	10.862
ct92-M305Pete-12	120362	14/09/2012	-2	78.914	10.798
ct92-M305Pete-12	120362	14/09/2012	-1	78.911	10.789
ct92-M305Pete-12	120362	14/09/2012	-2	78.914	10.81
ct92-M305Pete-12	120362	14/09/2012	-1	78.915	10.75
ct92-M305Pete-12	120362	14/09/2012	1	78.923	10.778
ct92-M305Pete-12	120362	14/09/2012	-2	78.918	10.764
ct92-M305Pete-12	120362	14/09/2012	1	78.919	10.757
ct92-M305Pete-12	120362	14/09/2012	-1	78.929	10.774
ct92-M305Pete-12	120362	14/09/2012	0	78.922	10.668
ct92-M305Pete-12	120362	14/09/2012	-2	78.922	10.668
ct92-M305Pete-12	120362	14/09/2012	-1	78.92	10.764
ct92-M305Pete-12	120362	14/09/2012	-2	78.917	10.761
ct92-M305Pete-12	120362	14/09/2012	-2	78.932	10.774
ct92-M305Pete-12	120362	14/09/2012	-1	78.914	10.803
ct92-M305Pete-12	120362	14/09/2012	0	78.908	10.812
ct92-M305Pete-12	120362	14/09/2012	-1	78.911	10.768
ct92-M305Pete-12	120362	14/09/2012	-2	78.918	10.784
ct92-M305Pete-12	120362	14/09/2012	-1	78.928	10.77
ct92-M305Pete-12	120362	14/09/2012	-1	78.925	10.752
ct92-M305Pete-12	120362	14/09/2012	-2	78.925	10.752
ct92-M305Pete-12	120362	14/09/2012	1	78.925	10.757
ct92-M305Pete-12	120362	14/09/2012	-2	78.925	10.761
ct92-M305Pete-12	120362	14/09/2012	-2	78.931	10.766
ct92-M305Pete-12	120362	14/09/2012	-2	78.927	10.756
ct92-M305Pete-12	120362	14/09/2012	2	78.939	10.785
ct92-M305Pete-12	120362	14/09/2012	2	78.939	10.784
ct92-M305Pete-12	120362	14/09/2012	-2	78.938	10.719
ct92-M305Pete-12	120362	14/09/2012	1	78.942	10.785
ct92-M305Pete-12	120362	14/09/2012	1	78.941	10.69
ct92-M305Pete-12	120362	14/09/2012	1	78.935	10.768
ct92-M305Pete-12	120362	14/09/2012	2	78.928	10.756
ct92-M305Pete-12	120362	14/09/2012	-1	78.918	10.763
ct92-M305Pete-12	120362	14/09/2012	1	78.923	10.767
ct92-M305Pete-12	120362	14/09/2012	-1	78.926	10.788
ct92-M305Pete-12	120362	14/09/2012	3	78.929	10.722
ct92-M305Pete-12	120362	14/09/2012	-2	78.931	10.704
ct92-M305Pete-12	120362	14/09/2012	1	78.933	10.654
ct92-M305Pete-12	120362	14/09/2012	1	78.936	10.666
ct92-M305Pete-12	120362	14/09/2012	-1	78.938	10.651

ct92-M305Pete-12	120362	14/09/2012	-2	78.941	10.623
ct92-M305Pete-12	120362	14/09/2012	-2	78.93	10.643
ct92-M305Pete-12	120362	14/09/2012	-2	78.941	10.694
ct92-M305Pete-12	120362	15/09/2012	-2	78.934	10.627
ct92-M305Pete-12	120362	15/09/2012	-1	78.935	10.759
ct92-M305Pete-12	120362	15/09/2012	-2	78.928	10.721
ct92-M305Pete-12	120362	15/09/2012	-2	78.931	10.697
ct92-M305Pete-12	120362	15/09/2012	-1	78.922	10.704
ct92-M305Pete-12	120362	15/09/2012	-2	78.924	10.687
ct92-M305Pete-12	120362	15/09/2012	-2	78.932	10.69
ct92-M305Pete-12	120362	15/09/2012	-2	78.927	10.768
ct92-M305Pete-12	120362	15/09/2012	1	78.926	10.758
ct92-M305Pete-12	120362	15/09/2012	-2	78.924	10.758
ct92-M305Pete-12	120362	15/09/2012	-2	78.924	10.762
ct92-M305Pete-12	120362	15/09/2012	-1	78.915	10.79
ct92-M305Pete-12	120362	15/09/2012	-1	78.918	10.799
ct92-M305Pete-12	120362	15/09/2012	1	78.917	10.763
ct92-M305Pete-12	120362	15/09/2012	-2	78.917	10.761
ct92-M305Pete-12	120362	15/09/2012	-2	78.918	10.764
ct92-M305Pete-12	120362	15/09/2012	1	78.915	10.742
ct92-M305Pete-12	120362	15/09/2012	-2	78.916	10.735
ct92-M305Pete-12	120362	15/09/2012	-2	78.922	10.689
ct92-M305Pete-12	120362	15/09/2012	-2	78.919	10.652
ct92-M305Pete-12	120362	15/09/2012	-2	78.918	10.651
ct92-M305Pete-12	120362	15/09/2012	-2	78.923	10.648
ct92-M305Pete-12	120362	15/09/2012	1	78.895	10.621
ct92-M305Pete-12	120362	15/09/2012	-2	78.918	10.646
ct92-M305Pete-12	120362	15/09/2012	2	78.919	10.622
ct92-M305Pete-12	120362	15/09/2012	1	78.918	10.622
ct92-M305Pete-12	120362	15/09/2012	-1	78.915	10.626
ct92-M305Pete-12	120362	15/09/2012	-2	78.915	10.625
ct92-M305Pete-12	120362	15/09/2012	-2	78.914	10.589
ct92-M305Pete-12	120362	15/09/2012	-2	78.918	10.581
ct92-M305Pete-12	120362	15/09/2012	-1	78.927	10.497
ct92-M305Pete-12	120362	15/09/2012	-2	78.919	10.579
ct92-M305Pete-12	120362	15/09/2012	1	78.912	10.591
ct92-M305Pete-12	120362	15/09/2012	-2	78.912	10.586
ct92-M305Pete-12	120362	15/09/2012	-1	78.916	10.591
ct92-M305Pete-12	120362	15/09/2012	-2	78.917	10.588
ct92-M305Pete-12	120362	15/09/2012	-2	78.915	10.665
ct92-M305Pete-12	120362	15/09/2012	-2	78.917	10.585
ct92-M305Pete-12	120362	15/09/2012	-2	78.918	10.58
ct92-M305Pete-12	120362	15/09/2012	-2	78.918	10.577
ct92-M305Pete-12	120362	15/09/2012	-2	78.913	10.698
ct92-M305Pete-12	120362	15/09/2012	-2	78.91	10.696
ct92-M305Pete-12	120362	15/09/2012	-2	78.914	10.696
ct92-M305Pete-12	120362	15/09/2012	-2	78.909	10.721
ct92-M305Pete-12	120362	15/09/2012	-2	78.916	10.594
ct92-M305Pete-12	120362	15/09/2012	-2	78.917	10.601
ct92-M305Pete-12	120362	15/09/2012	-2	78.915	10.736
ct92-M305Pete-12	120362	15/09/2012	-1	78.904	10.588
ct92-M305Pete-12	120362	15/09/2012	-2	78.92	10.666
ct92-M305Pete-12	120362	15/09/2012	-2	78.926	10.613
ct92-M305Pete-12	120362	15/09/2012	-2	78.926	10.64
ct92-M305Pete-12	120362	15/09/2012	-2	78.926	10.626
ct92-M305Pete-12	120362	15/09/2012	-2	78.921	10.641
ct92-M305Pete-12	120362	15/09/2012	-2	78.923	10.656
ct92-M305Pete-12	120362	15/09/2012	-2	78.922	10.679
ct92-M305Pete-12	120362	15/09/2012	-2	78.928	10.671
ct92-M305Pete-12	120362	15/09/2012	-2	78.929	10.675
ct92-M305Pete-12	120362	15/09/2012	-1	78.916	10.628
ct92-M305Pete-12	120362	15/09/2012	-2	78.918	10.659
ct92-M305Pete-12	120362	15/09/2012	-2	78.932	10.632
ct92-M305Pete-12	120362	15/09/2012	-1	78.936	10.752
ct92-M305Pete-12	120362	15/09/2012	-2	78.935	10.741
ct92-M305Pete-12	120362	15/09/2012	-2	78.939	10.73
ct92-M305Pete-12	120362	15/09/2012	-1	78.93	10.714
ct92-M305Pete-12	120362	15/09/2012	-2	78.93	10.74

ct92-M305Pete-12	120362	16/09/2012	-2	78.927	10.778
ct92-M305Pete-12	120362	16/09/2012	-2	78.928	10.753
ct92-M305Pete-12	120362	16/09/2012	1	78.946	10.737
ct92-M305Pete-12	120362	16/09/2012	1	78.923	10.802
ct92-M305Pete-12	120362	16/09/2012	-2	78.944	10.685
ct92-M305Pete-12	120362	16/09/2012	-1	78.917	10.815
ct92-M305Pete-12	120362	16/09/2012	-2	78.923	10.858
ct92-M305Pete-12	120362	16/09/2012	-2	78.922	10.851
ct92-M305Pete-12	120362	16/09/2012	-1	78.927	10.794
ct92-M305Pete-12	120362	16/09/2012	-2	78.93	10.834
ct92-M305Pete-12	120362	16/09/2012	-2	78.922	10.795
ct92-M305Pete-12	120362	16/09/2012	-2	78.925	10.838
ct92-M305Pete-12	120362	16/09/2012	-2	78.921	10.859
ct92-M305Pete-12	120362	16/09/2012	0	78.922	10.836
ct92-M305Pete-12	120362	16/09/2012	-2	78.919	10.855
ct92-M305Pete-12	120362	16/09/2012	-2	78.919	10.857
ct92-M305Pete-12	120362	16/09/2012	-2	78.92	10.814
ct92-M305Pete-12	120362	16/09/2012	-2	78.918	10.854
ct92-M305Pete-12	120362	16/09/2012	1	78.92	10.814
ct92-M305Pete-12	120362	16/09/2012	1	78.921	10.799
ct92-M305Pete-12	120362	16/09/2012	-2	78.941	10.799
ct92-M305Pete-12	120362	16/09/2012	-2	78.927	10.703
ct92-M305Pete-12	120362	16/09/2012	-2	78.926	10.723
ct92-M305Pete-12	120362	16/09/2012	-2	78.924	10.744
ct92-M305Pete-12	120362	16/09/2012	-2	78.925	10.746
ct92-M305Pete-12	120362	16/09/2012	-2	78.931	10.735
ct92-M305Pete-12	120362	16/09/2012	-2	78.924	10.72
ct92-M305Pete-12	120362	16/09/2012	-1	78.918	10.732
ct92-M305Pete-12	120362	16/09/2012	-2	78.92	10.734
ct92-M305Pete-12	120362	16/09/2012	-1	78.918	10.755
ct92-M305Pete-12	120362	16/09/2012	-2	78.913	10.748
ct92-M305Pete-12	120362	16/09/2012	-2	78.911	10.787
ct92-M305Pete-12	120362	16/09/2012	1	78.915	10.812
ct92-M305Pete-12	120362	16/09/2012	-2	78.913	10.761
ct92-M305Pete-12	120362	16/09/2012	-2	78.903	10.871
ct92-M305Pete-12	120362	16/09/2012	-2	78.922	10.846
ct92-M305Pete-12	120362	16/09/2012	-2	78.916	10.804
ct92-M305Pete-12	120362	16/09/2012	-2	78.913	10.746
ct92-M305Pete-12	120362	16/09/2012	-2	78.917	10.818
ct92-M305Pete-12	120362	16/09/2012	-2	78.918	10.813
ct92-M305Pete-12	120362	16/09/2012	-2	78.918	10.84
ct92-M305Pete-12	120362	16/09/2012	-2	78.919	10.821
ct92-M305Pete-12	120362	16/09/2012	-2	78.918	10.84
ct92-M305Pete-12	120362	16/09/2012	-2	78.918	10.84
ct92-M305Pete-12	120362	16/09/2012	-2	78.927	10.82
ct92-M305Pete-12	120362	16/09/2012	-1	78.945	10.846
ct92-M305Pete-12	120362	16/09/2012	1	78.934	10.76
ct92-M305Pete-12	120362	16/09/2012	-2	78.935	10.764
ct92-M305Pete-12	120362	16/09/2012	-2	78.926	10.661
ct92-M305Pete-12	120362	16/09/2012	-2	78.924	10.632
ct92-M305Pete-12	120362	16/09/2012	-2	78.95	10.667
ct92-M305Pete-12	120362	16/09/2012	-2	78.941	10.79
ct92-M305Pete-12	120362	16/09/2012	-1	78.924	10.649
ct92-M305Pete-12	120362	16/09/2012	-2	78.927	10.665
ct92-M305Pete-12	120362	16/09/2012	-2	78.941	10.701
ct92-M305Pete-12	120362	16/09/2012	-1	78.927	10.602
ct92-M305Pete-12	120362	16/09/2012	-2	78.926	10.609
ct92-M305Pete-12	120362	16/09/2012	-2	78.926	10.723
ct92-M305Pete-12	120362	16/09/2012	-2	78.928	10.715
ct92-M305Pete-12	120362	16/09/2012	-2	78.929	10.653
ct92-M305Pete-12	120362	16/09/2012	-2	78.941	10.628
ct92-M305Pete-12	120362	16/09/2012	-2	78.923	10.596
ct92-M305Pete-12	120362	17/09/2012	-2	78.926	10.563
ct92-M305Pete-12	120362	17/09/2012	-2	78.929	10.569
ct92-M305Pete-12	120362	17/09/2012	-2	78.94	10.666
ct92-M305Pete-12	120362	17/09/2012	-2	78.946	10.677
ct92-M305Pete-12	120362	17/09/2012	-2	78.943	10.694
ct92-M305Pete-12	120362	17/09/2012	2	78.941	10.663

ct92-M305Pete-12	120362	17/09/2012	-1	78.942	10.693
ct92-M305Pete-12	120362	17/09/2012	-2	78.941	10.663
ct92-M305Pete-12	120362	17/09/2012	-2	78.946	10.695
ct92-M305Pete-12	120362	17/09/2012	-2	78.948	10.698
ct92-M305Pete-12	120362	17/09/2012	-1	78.935	10.729
ct92-M305Pete-12	120362	17/09/2012	-2	78.942	10.691
ct92-M305Pete-12	120362	17/09/2012	-1	78.926	10.766
ct92-M305Pete-12	120362	17/09/2012	-2	78.935	10.729
ct92-M305Pete-12	120362	17/09/2012	-2	78.934	10.708
ct92-M305Pete-12	120362	17/09/2012	-2	78.935	10.705
ct92-M305Pete-12	120362	17/09/2012	2	78.928	10.695
ct92-M305Pete-12	120362	17/09/2012	-1	78.923	10.667
ct92-M305Pete-12	120362	17/09/2012	-2	78.923	10.665
ct92-M305Pete-12	120362	17/09/2012	-2	78.924	10.666
ct92-M305Pete-12	120362	17/09/2012	-2	78.924	10.668
ct92-M305Pete-12	120362	17/09/2012	-1	78.922	10.655
ct92-M305Pete-12	120362	17/09/2012	-2	78.919	10.659
ct92-M305Pete-12	120362	17/09/2012	-1	78.908	10.798
ct92-M305Pete-12	120362	17/09/2012	-2	78.922	10.71
ct92-M305Pete-12	120362	17/09/2012	2	78.921	10.706
ct92-M305Pete-12	120362	17/09/2012	-2	78.922	10.71
ct92-M305Pete-12	120362	17/09/2012	-2	78.917	10.724
ct92-M305Pete-12	120362	17/09/2012	-2	78.914	10.743
ct92-M305Pete-12	120362	17/09/2012	-2	78.916	10.749
ct92-M305Pete-12	120362	17/09/2012	-2	78.916	10.749
ct92-M305Pete-12	120362	17/09/2012	-2	78.915	10.755
ct92-M305Pete-12	120362	17/09/2012	-2	78.915	10.742
ct92-M305Pete-12	120362	17/09/2012	-2	78.905	10.719
ct92-M305Pete-12	120362	17/09/2012	-2	78.906	10.71
ct92-M305Pete-12	120362	17/09/2012	-2	78.902	10.698
ct92-M305Pete-12	120362	17/09/2012	-1	78.9	10.724
ct92-M305Pete-12	120362	17/09/2012	-2	78.897	10.722
ct92-M305Pete-12	120362	17/09/2012	-2	78.895	10.721
ct92-M305Pete-12	120362	17/09/2012	-2	78.911	10.708
ct92-M305Pete-12	120362	17/09/2012	-2	78.909	10.684
ct92-M305Pete-12	120362	17/09/2012	-2	78.917	10.675
ct92-M305Pete-12	120362	17/09/2012	-1	78.922	10.729
ct92-M305Pete-12	120362	17/09/2012	-2	78.901	10.698
ct92-M305Pete-12	120362	17/09/2012	-2	78.922	10.717
ct92-M305Pete-12	120362	17/09/2012	-2	78.921	10.715
ct92-M305Pete-12	120362	17/09/2012	-2	78.922	10.712
ct92-M305Pete-12	120362	17/09/2012	-1	78.936	10.614
ct92-M305Pete-12	120362	17/09/2012	-2	78.936	10.603
ct92-M305Pete-12	120362	17/09/2012	-2	78.926	10.77
ct92-M305Pete-12	120362	17/09/2012	-2	78.926	10.772
ct92-M305Pete-12	120362	17/09/2012	-2	78.924	10.771
ct92-M305Pete-12	120362	17/09/2012	-2	78.921	10.693
ct92-M305Pete-12	120362	17/09/2012	-1	78.925	10.747
ct92-M305Pete-12	120362	17/09/2012	-2	78.926	10.798
ct92-M305Pete-12	120362	17/09/2012	-1	78.925	10.747
ct92-M305Pete-12	120362	17/09/2012	-2	78.926	10.798
ct92-M305Pete-12	120362	17/09/2012	-2	78.914	10.664
ct92-M305Pete-12	120362	18/09/2012	-2	78.921	10.623
ct92-M305Pete-12	120362	18/09/2012	-2	78.938	10.578
ct92-M305Pete-12	120362	18/09/2012	-2	78.954	10.599
ct92-M305Pete-12	120362	18/09/2012	-1	78.953	10.601
ct92-M305Pete-12	120362	18/09/2012	-2	78.963	10.64
ct92-M305Pete-12	120362	18/09/2012	-2	78.963	10.644
ct92-M305Pete-12	120362	18/09/2012	-1	78.956	10.746
ct92-M305Pete-12	120362	18/09/2012	-2	78.958	10.756
ct92-M305Pete-12	120362	18/09/2012	-2	78.957	10.75
ct92-M305Pete-12	120362	18/09/2012	-2	78.963	10.703
ct92-M305Pete-12	120362	18/09/2012	-1	78.927	10.758
ct92-M305Pete-12	120362	18/09/2012	-2	78.934	10.757
ct92-M305Pete-12	120362	18/09/2012	-2	78.924	10.709
ct92-M305Pete-12	120362	18/09/2012	-1	78.933	10.666
ct92-M305Pete-12	120362	18/09/2012	-2	78.922	10.714
ct92-M305Pete-12	120362	18/09/2012	-2	78.922	10.713

ct92-M305Pete-12	120362	18/09/2012	-2	78.922	10.681
ct92-M305Pete-12	120362	18/09/2012	-2	78.916	10.622
ct92-M305Pete-12	120362	18/09/2012	1	78.912	10.635
ct92-M305Pete-12	120362	18/09/2012	-2	78.929	10.59
ct92-M305Pete-12	120362	18/09/2012	-1	78.925	10.6
ct92-M305Pete-12	120362	18/09/2012	-1	78.925	10.596
ct92-M305Pete-12	120362	18/09/2012	-2	78.925	10.604
ct92-M305Pete-12	120362	18/09/2012	-1	78.927	10.642
ct92-M305Pete-12	120362	18/09/2012	-2	78.917	10.74
ct92-M305Pete-12	120362	18/09/2012	-2	78.917	10.752
ct92-M305Pete-12	120362	18/09/2012	0	78.914	10.771
ct92-M305Pete-12	120362	18/09/2012	2	78.938	10.734
ct92-M305Pete-12	120362	18/09/2012	2	78.921	10.802
ct92-M305Pete-12	120362	18/09/2012	-1	78.919	10.822
ct92-M305Pete-12	120362	18/09/2012	2	78.901	10.803
ct92-M305Pete-12	120362	18/09/2012	-2	78.917	10.823
ct92-M305Pete-12	120362	18/09/2012	-2	78.915	10.797
ct92-M305Pete-12	120362	18/09/2012	-1	78.917	10.781
ct92-M305Pete-12	120362	18/09/2012	2	78.917	10.79
ct92-M305Pete-12	120362	18/09/2012	-2	78.912	10.822
ct92-M305Pete-12	120362	18/09/2012	-1	78.92	10.727
ct92-M305Pete-12	120362	18/09/2012	-1	78.923	10.771
ct92-M305Pete-12	120362	18/09/2012	-2	78.921	10.778
ct92-M305Pete-12	120362	18/09/2012	-2	78.92	10.767
ct92-M305Pete-12	120362	18/09/2012	-1	78.922	10.758
ct92-M305Pete-12	120362	18/09/2012	-2	78.924	10.81
ct92-M305Pete-12	120362	18/09/2012	-1	78.936	10.837
ct92-M305Pete-12	120362	18/09/2012	-2	78.926	10.822
ct92-M305Pete-12	120362	18/09/2012	-2	78.936	10.839
ct92-M305Pete-12	120362	18/09/2012	-2	78.931	10.796
ct92-M305Pete-12	120362	18/09/2012	-2	78.93	10.798
ct92-M305Pete-12	120362	18/09/2012	-2	78.932	10.83
ct92-M305Pete-12	120362	18/09/2012	-2	78.914	10.834
ct92-M305Pete-12	120362	18/09/2012	-2	78.925	10.839
ct92-M305Pete-12	120362	18/09/2012	-2	78.916	10.825
ct92-M305Pete-12	120362	18/09/2012	-2	78.917	10.821
ct92-M305Pete-12	120362	18/09/2012	-2	78.923	10.777
ct92-M305Pete-12	120362	18/09/2012	-1	78.924	10.798
ct92-M305Pete-12	120362	18/09/2012	-2	78.921	10.812
ct92-M305Pete-12	120362	18/09/2012	0	78.931	10.813
ct92-M305Pete-12	120362	18/09/2012	-2	78.919	10.81
ct92-M305Pete-12	120362	18/09/2012	-1	78.926	10.78
ct92-M305Pete-12	120362	18/09/2012	2	78.924	10.81
ct92-M305Pete-12	120362	18/09/2012	-2	78.922	10.815
ct92-M305Pete-12	120362	18/09/2012	-2	78.928	10.787
ct92-M305Pete-12	120362	18/09/2012	-2	78.925	10.794
ct92-M305Pete-12	120362	18/09/2012	-2	78.943	10.721
ct92-M305Pete-12	120362	18/09/2012	-2	78.937	10.725
ct92-M305Pete-12	120362	19/09/2012	-2	78.939	10.714
ct92-M305Pete-12	120362	19/09/2012	-1	78.934	10.603
ct92-M305Pete-12	120362	19/09/2012	2	78.937	10.609
ct92-M305Pete-12	120362	19/09/2012	1	78.931	10.558
ct92-M305Pete-12	120362	19/09/2012	-1	78.933	10.588
ct92-M305Pete-12	120362	19/09/2012	-1	78.933	10.666
ct92-M305Pete-12	120362	19/09/2012	-2	78.937	10.681
ct92-M305Pete-12	120362	19/09/2012	0	78.931	10.697
ct92-M305Pete-12	120362	19/09/2012	-1	78.926	10.741
ct92-M305Pete-12	120362	19/09/2012	-2	78.926	10.748
ct92-M305Pete-12	120362	19/09/2012	-2	78.933	10.675
ct92-M305Pete-12	120362	19/09/2012	-2	78.936	10.704
ct92-M305Pete-12	120362	19/09/2012	-2	78.913	10.814
ct92-M305Pete-12	120362	19/09/2012	-1	78.937	10.755
ct92-M305Pete-12	120362	19/09/2012	-2	78.937	10.758
ct92-M305Pete-12	120362	19/09/2012	0	78.906	10.867
ct92-M305Pete-12	120362	19/09/2012	-2	78.906	10.868
ct92-M305Pete-12	120362	19/09/2012	1	78.91	10.854
ct92-M305Pete-12	120362	19/09/2012	1	78.908	10.829
ct92-M305Pete-12	120362	19/09/2012	-2	78.924	10.784

ct92-M305Pete-12	120362	19/09/2012	-2	78.924	10.789
ct92-M305Pete-12	120362	19/09/2012	1	78.903	10.804
ct92-M305Pete-12	120362	19/09/2012	-1	78.9	10.848
ct92-M305Pete-12	120362	19/09/2012	-2	78.894	10.838
ct92-M305Pete-12	120362	19/09/2012	-2	78.898	10.84
ct92-M305Pete-12	120362	19/09/2012	-2	78.905	10.707
ct92-M305Pete-12	120362	19/09/2012	-2	78.909	10.717
ct92-M305Pete-12	120362	19/09/2012	2	78.92	10.753
ct92-M305Pete-12	120362	19/09/2012	-2	78.919	10.751
ct92-M305Pete-12	120362	19/09/2012	0	78.921	10.738
ct92-M305Pete-12	120362	19/09/2012	-2	78.918	10.819
ct92-M305Pete-12	120362	19/09/2012	-2	78.917	10.745
ct92-M305Pete-12	120362	19/09/2012	0	78.922	10.753
ct92-M305Pete-12	120362	19/09/2012	2	78.923	10.756
ct92-M305Pete-12	120362	19/09/2012	-2	78.921	10.756
ct92-M305Pete-12	120362	19/09/2012	-1	78.919	10.751
ct92-M305Pete-12	120362	19/09/2012	-2	78.917	10.771
ct92-M305Pete-12	120362	19/09/2012	-2	78.917	10.747
ct92-M305Pete-12	120362	19/09/2012	-2	78.917	10.73
ct92-M305Pete-12	120362	19/09/2012	-2	78.917	10.747
ct92-M305Pete-12	120362	19/09/2012	-2	78.914	10.732
ct92-M305Pete-12	120362	19/09/2012	-2	78.912	10.722
ct92-M305Pete-12	120362	19/09/2012	-2	78.914	10.704
ct92-M305Pete-12	120362	19/09/2012	-2	78.903	10.739
ct92-M305Pete-12	120362	19/09/2012	-2	78.921	10.686
ct92-M305Pete-12	120362	19/09/2012	-1	78.931	10.769
ct92-M305Pete-12	120362	19/09/2012	-2	78.919	10.713
ct92-M305Pete-12	120362	19/09/2012	-2	78.925	10.725
ct92-M305Pete-12	120362	19/09/2012	-2	78.921	10.73
ct92-M305Pete-12	120362	19/09/2012	-2	78.921	10.737
ct92-M305Pete-12	120362	19/09/2012	-2	78.921	10.736
ct92-M305Pete-12	120362	19/09/2012	-2	78.927	10.75
ct92-M305Pete-12	120362	19/09/2012	-2	78.923	10.765
ct92-M305Pete-12	120362	19/09/2012	-2	78.924	10.761
ct92-M305Pete-12	120362	19/09/2012	-2	78.919	10.762
ct92-M305Pete-12	120362	19/09/2012	-1	78.914	10.805
ct92-M305Pete-12	120362	19/09/2012	-2	78.919	10.824
ct92-M305Pete-12	120362	19/09/2012	-2	78.925	10.819
ct92-M305Pete-12	120362	19/09/2012	-2	78.92	10.796
ct92-M305Pete-12	120362	19/09/2012	-2	78.922	10.791
ct92-M305Pete-12	120362	19/09/2012	-2	78.926	10.815
ct92-M305Pete-12	120362	19/09/2012	-2	78.942	10.751
ct92-M305Pete-12	120362	19/09/2012	-2	78.925	10.818
ct92-M305Pete-12	120362	20/09/2012	-2	78.887	10.389
ct92-M305Pete-12	120362	20/09/2012	-2	78.921	10.481
ct92-M305Pete-12	120362	20/09/2012	-2	78.922	10.497
ct92-M305Pete-12	120362	20/09/2012	-2	78.922	10.458
ct92-M305Pete-12	120362	20/09/2012	1	78.941	10.621
ct92-M305Pete-12	120362	20/09/2012	2	78.943	10.602
ct92-M305Pete-12	120362	20/09/2012	-2	78.943	10.602
ct92-M305Pete-12	120362	20/09/2012	-1	78.944	10.619
ct92-M305Pete-12	120362	20/09/2012	-1	78.93	10.695
ct92-M305Pete-12	120362	20/09/2012	-1	78.932	10.679
ct92-M305Pete-12	120362	20/09/2012	-2	78.927	10.697
ct92-M305Pete-12	120362	20/09/2012	-2	78.927	10.688
ct92-M305Pete-12	120362	20/09/2012	2	78.92	10.813
ct92-M305Pete-12	120362	20/09/2012	-1	78.922	10.766
ct92-M305Pete-12	120362	20/09/2012	-1	78.922	10.767
ct92-M305Pete-12	120362	20/09/2012	-2	78.919	10.772
ct92-M305Pete-12	120362	20/09/2012	-2	78.92	10.774
ct92-M305Pete-12	120362	20/09/2012	-2	78.92	10.789
ct92-M305Pete-12	120362	20/09/2012	-2	78.92	10.783
ct92-M305Pete-12	120362	20/09/2012	-2	78.919	10.749
ct92-M305Pete-12	120362	20/09/2012	-2	78.881	10.753
ct92-M305Pete-12	120362	20/09/2012	-2	78.881	10.754
ct92-M305Pete-12	120362	20/09/2012	0	78.874	10.847
ct92-M305Pete-12	120362	20/09/2012	-2	78.883	10.855
ct92-M305Pete-12	120362	20/09/2012	-2	78.917	10.831

ct92-M305Pete-12	120362	20/09/2012	-1	78.922	10.665
ct92-M305Pete-12	120362	20/09/2012	-1	78.922	10.663
ct92-M305Pete-12	120362	20/09/2012	2	78.919	10.766
ct92-M305Pete-12	120362	20/09/2012	-1	78.912	10.751
ct92-M305Pete-12	120362	20/09/2012	-2	78.923	10.753
ct92-M305Pete-12	120362	20/09/2012	-2	78.881	10.891
ct92-M305Pete-12	120362	20/09/2012	-2	78.883	10.77
ct92-M305Pete-12	120362	20/09/2012	0	78.926	10.706
ct92-M305Pete-12	120362	20/09/2012	-2	78.92	10.698
ct92-M305Pete-12	120362	20/09/2012	-2	78.923	10.706
ct92-M305Pete-12	120362	20/09/2012	1	78.918	10.752
ct92-M305Pete-12	120362	20/09/2012	-1	78.921	10.655
ct92-M305Pete-12	120362	20/09/2012	2	78.927	10.717
ct92-M305Pete-12	120362	20/09/2012	1	78.923	10.727
ct92-M305Pete-12	120362	20/09/2012	-1	78.93	10.726
ct92-M305Pete-12	120362	20/09/2012	1	78.928	10.734
ct92-M305Pete-12	120362	20/09/2012	2	78.929	10.718
ct92-M305Pete-12	120362	20/09/2012	1	78.94	10.746
ct92-M305Pete-12	120362	20/09/2012	0	78.932	10.701
ct92-M305Pete-12	120362	20/09/2012	1	78.939	10.707
ct92-M305Pete-12	120362	20/09/2012	-2	78.943	10.709
ct92-M305Pete-12	120362	20/09/2012	-2	78.928	10.764
ct92-M305Pete-12	120362	20/09/2012	-2	78.928	10.788
ct92-M305Pete-12	120362	20/09/2012	1	78.93	10.696
ct92-M305Pete-12	120362	20/09/2012	-2	78.935	10.711
ct92-M305Pete-12	120362	20/09/2012	-2	78.93	10.713
ct92-M305Pete-12	120362	20/09/2012	-2	78.933	10.72
ct92-M305Pete-12	120362	20/09/2012	-2	78.949	10.74
ct92-M305Pete-12	120362	21/09/2012	-2	78.943	10.676
ct92-M305Pete-12	120362	21/09/2012	-1	78.946	10.667
ct92-M305Pete-12	120362	21/09/2012	-2	78.926	10.622
ct92-M305Pete-12	120362	21/09/2012	-2	78.929	10.599
ct92-M305Pete-12	120362	21/09/2012	-1	78.931	10.622
ct92-M305Pete-12	120362	21/09/2012	-2	78.933	10.582
ct92-M305Pete-12	120362	21/09/2012	-2	78.923	10.664
ct92-M305Pete-12	120362	21/09/2012	-1	78.922	10.759
ct92-M305Pete-12	120362	21/09/2012	-1	78.922	10.757
ct92-M305Pete-12	120362	21/09/2012	0	78.922	10.787
ct92-M305Pete-12	120362	21/09/2012	-1	78.919	10.762
ct92-M305Pete-12	120362	21/09/2012	-2	78.925	10.769
ct92-M305Pete-12	120362	21/09/2012	-2	78.921	10.765
ct92-M305Pete-12	120362	21/09/2012	-2	78.924	10.721
ct92-M305Pete-12	120362	21/09/2012	-2	78.92	10.744
ct92-M305Pete-12	120362	21/09/2012	-2	78.92	10.763
ct92-M305Pete-12	120362	21/09/2012	-2	78.922	10.753
ct92-M305Pete-12	120362	21/09/2012	-1	78.941	10.639
ct92-M305Pete-12	120362	21/09/2012	0	78.942	10.634
ct92-M305Pete-12	120362	21/09/2012	-2	78.936	10.658
ct92-M305Pete-12	120362	21/09/2012	-2	78.949	10.652
ct92-M305Pete-12	120362	21/09/2012	1	78.929	10.694
ct92-M305Pete-12	120362	21/09/2012	-1	78.92	10.656
ct92-M305Pete-12	120362	21/09/2012	-2	78.93	10.69
ct92-M305Pete-12	120362	21/09/2012	-2	78.93	10.69
ct92-M305Pete-12	120362	21/09/2012	-2	78.931	10.702
ct92-M305Pete-12	120362	21/09/2012	-2	78.92	10.68
ct92-M305Pete-12	120362	21/09/2012	-2	78.916	10.699
ct92-M305Pete-12	120362	21/09/2012	-2	78.917	10.694
ct92-M305Pete-12	120362	21/09/2012	-2	78.927	10.691
ct92-M305Pete-12	120362	21/09/2012	-1	78.928	10.698
ct92-M305Pete-12	120362	21/09/2012	-2	78.929	10.748
ct92-M305Pete-12	120362	21/09/2012	-2	78.929	10.742
ct92-M305Pete-12	120362	21/09/2012	-2	78.923	10.666
ct92-M305Pete-12	120362	21/09/2012	-1	78.924	10.636
ct92-M305Pete-12	120362	21/09/2012	-2	78.923	10.696
ct92-M305Pete-12	120362	21/09/2012	-1	78.918	10.689
ct92-M305Pete-12	120362	21/09/2012	-1	78.913	10.741
ct92-M305Pete-12	120362	21/09/2012	-1	78.94	10.733
ct92-M305Pete-12	120362	21/09/2012	-2	78.936	10.623

ct92-M305Pete-12	120362	21/09/2012	-1	78.928	10.665
ct92-M305Pete-12	120362	21/09/2012	-2	78.927	10.652
ct92-M305Pete-12	120362	21/09/2012	-2	78.926	10.724
ct92-M305Pete-12	120362	21/09/2012	-2	78.927	10.705
ct92-M305Pete-12	120362	21/09/2012	-2	78.937	10.795
ct92-M305Pete-12	120362	21/09/2012	-2	78.934	10.723
ct92-M305Pete-12	120362	21/09/2012	-2	78.929	10.694
ct92-M305Pete-12	120362	21/09/2012	0	78.938	10.723
ct92-M305Pete-12	120362	21/09/2012	2	78.933	10.698
ct92-M305Pete-12	120362	21/09/2012	2	78.928	10.701
ct92-M305Pete-12	120362	21/09/2012	-2	78.926	10.702
ct92-M305Pete-12	120362	21/09/2012	0	78.92	10.766
ct92-M305Pete-12	120362	21/09/2012	1	78.919	10.756
ct92-M305Pete-12	120362	21/09/2012	-2	78.917	10.775
ct92-M305Pete-12	120362	21/09/2012	1	78.918	10.795
ct92-M305Pete-12	120362	21/09/2012	-1	78.948	10.812
ct92-M305Pete-12	120362	21/09/2012	2	78.927	10.786
ct92-M305Pete-12	120362	21/09/2012	-2	78.94	10.88
ct92-M305Pete-12	120362	21/09/2012	0	78.919	10.677
ct92-M305Pete-12	120362	21/09/2012	-2	78.922	10.669
ct92-M305Pete-12	120362	21/09/2012	-2	78.92	10.688
ct92-M305Pete-12	120362	21/09/2012	-2	78.92	10.694
ct92-M305Pete-12	120362	21/09/2012	-2	78.943	10.568
ct92-M305Pete-12	120362	21/09/2012	-1	78.938	10.635
ct92-M305Pete-12	120362	21/09/2012	-1	78.938	10.635
ct92-M305Pete-12	120362	21/09/2012	0	78.928	10.558
ct92-M305Pete-12	120362	21/09/2012	-2	78.931	10.557
ct92-M305Pete-12	120362	21/09/2012	0	78.925	10.596
ct92-M305Pete-12	120362	22/09/2012	-2	78.914	10.477
ct92-M305Pete-12	120362	22/09/2012	-2	78.918	10.532
ct92-M305Pete-12	120362	22/09/2012	-2	78.923	10.815
ct92-M305Pete-12	120362	22/09/2012	-2	78.919	10.806
ct92-M305Pete-12	120362	22/09/2012	-2	78.923	10.783
ct92-M305Pete-12	120362	22/09/2012	-2	78.914	10.766
ct92-M305Pete-12	120362	22/09/2012	-2	78.91	10.791
ct92-M305Pete-12	120362	22/09/2012	-2	78.918	10.704
ct92-M305Pete-12	120362	22/09/2012	-2	78.921	10.692
ct92-M305Pete-12	120362	22/09/2012	-1	78.919	10.725
ct92-M305Pete-12	120362	22/09/2012	-2	78.93	10.677
ct92-M305Pete-12	120362	22/09/2012	-2	78.93	10.674
ct92-M305Pete-12	120362	22/09/2012	1	78.92	10.656
ct92-M305Pete-12	120362	22/09/2012	1	78.926	10.611
ct92-M305Pete-12	120362	22/09/2012	-2	78.905	10.703
ct92-M305Pete-12	120362	22/09/2012	-2	78.906	10.657
ct92-M305Pete-12	120362	22/09/2012	-2	78.927	10.672
ct92-M305Pete-12	120362	22/09/2012	-2	78.927	10.67
ct92-M305Pete-12	120362	22/09/2012	-1	78.933	10.595
ct92-M305Pete-12	120362	22/09/2012	-2	78.937	10.528
ct92-M305Pete-12	120362	22/09/2012	-2	78.927	10.667
ct92-M305Pete-12	120362	22/09/2012	-2	78.928	10.66
ct92-M305Pete-12	120362	22/09/2012	-2	78.932	10.678
ct92-M305Pete-12	120362	22/09/2012	-2	78.932	10.662
ct92-M305Pete-12	120362	22/09/2012	-2	78.932	10.663
ct92-M305Pete-12	120362	22/09/2012	-2	78.933	10.664
ct92-M305Pete-12	120362	22/09/2012	-2	78.932	10.673
ct92-M305Pete-12	120362	22/09/2012	-1	78.938	10.576
ct92-M305Pete-12	120362	22/09/2012	-1	78.925	10.702
ct92-M305Pete-12	120362	22/09/2012	-2	78.923	10.719
ct92-M305Pete-12	120362	22/09/2012	-2	78.934	10.685
ct92-M305Pete-12	120362	22/09/2012	0	78.956	10.602
ct92-M305Pete-12	120362	22/09/2012	-2	78.93	10.725
ct92-M305Pete-12	120362	22/09/2012	-2	78.93	10.721
ct92-M305Pete-12	120362	22/09/2012	-1	78.912	10.669
ct92-M305Pete-12	120362	22/09/2012	-2	78.931	10.72
ct92-M305Pete-12	120362	22/09/2012	-1	78.944	10.673
ct92-M305Pete-12	120362	22/09/2012	-2	78.931	10.698
ct92-M305Pete-12	120362	22/09/2012	-2	78.936	10.661
ct92-M305Pete-12	120362	22/09/2012	-2	78.937	10.652

ct92-M305Pete-12	120362	22/09/2012	-1	78.92	10.742
ct92-M305Pete-12	120362	22/09/2012	-2	78.938	10.651
ct92-M305Pete-12	120362	22/09/2012	-2	78.933	10.675
ct92-M305Pete-12	120362	22/09/2012	-2	78.926	10.715
ct92-M305Pete-12	120362	22/09/2012	0	78.931	10.739
ct92-M305Pete-12	120362	22/09/2012	2	78.919	10.77
ct92-M305Pete-12	120362	22/09/2012	-1	78.92	10.789
ct92-M305Pete-12	120362	22/09/2012	1	78.922	10.779
ct92-M305Pete-12	120362	22/09/2012	-2	78.923	10.784
ct92-M305Pete-12	120362	22/09/2012	-2	78.913	10.836
ct92-M305Pete-12	120362	22/09/2012	-1	78.927	10.882
ct92-M305Pete-12	120362	22/09/2012	-1	78.916	10.781
ct92-M305Pete-12	120362	22/09/2012	-2	78.933	11.007
ct92-M305Pete-12	120362	22/09/2012	-1	78.916	10.887
ct92-M305Pete-12	120362	22/09/2012	-2	78.917	10.85
ct92-M305Pete-12	120362	22/09/2012	1	78.914	10.808
ct92-M305Pete-12	120362	22/09/2012	-1	78.915	10.722
ct92-M305Pete-12	120362	22/09/2012	-2	78.92	10.768
ct92-M305Pete-12	120362	22/09/2012	-2	78.92	10.773
ct92-M305Pete-12	120362	22/09/2012	1	78.92	10.797
ct92-M305Pete-12	120362	22/09/2012	-1	78.922	10.755
ct92-M305Pete-12	120362	22/09/2012	-2	78.932	10.774
ct92-M305Pete-12	120362	22/09/2012	-2	78.94	10.805
ct92-M305Pete-12	120362	22/09/2012	-2	78.94	10.81
ct92-M305Pete-12	120362	22/09/2012	-1	78.933	10.824
ct92-M305Pete-12	120362	22/09/2012	-1	78.95	10.744
ct92-M305Pete-12	120362	22/09/2012	1	78.907	10.671
ct92-M305Pete-12	120362	22/09/2012	2	78.921	10.745
ct92-M305Pete-12	120362	23/09/2012	-2	78.927	10.749
ct92-M305Pete-12	120362	23/09/2012	-2	78.926	10.753
ct92-M305Pete-12	120362	23/09/2012	-1	78.923	10.768
ct92-M305Pete-12	120362	23/09/2012	1	78.92	10.74
ct92-M305Pete-12	120362	23/09/2012	-2	78.93	10.751
ct92-M305Pete-12	120362	23/09/2012	-2	78.927	10.755
ct92-M305Pete-12	120362	23/09/2012	-2	78.93	10.713
ct92-M305Pete-12	120362	23/09/2012	-2	78.935	10.723
ct92-M305Pete-12	120362	23/09/2012	2	78.919	10.812
ct92-M305Pete-12	120362	23/09/2012	2	78.919	10.809
ct92-M305Pete-12	120362	23/09/2012	1	78.928	10.781
ct92-M305Pete-12	120362	23/09/2012	-2	78.925	10.772
ct92-M305Pete-12	120362	23/09/2012	-1	78.912	10.827
ct92-M305Pete-12	120362	23/09/2012	-1	78.911	10.827
ct92-M305Pete-12	120362	23/09/2012	-2	78.911	10.841
ct92-M305Pete-12	120362	23/09/2012	-2	78.907	10.882
ct92-M305Pete-12	120362	23/09/2012	-2	78.912	10.886
ct92-M305Pete-12	120362	23/09/2012	-2	78.914	10.848
ct92-M305Pete-12	120362	23/09/2012	-2	78.915	10.859
ct92-M305Pete-12	120362	23/09/2012	-2	78.916	10.88
ct92-M305Pete-12	120362	23/09/2012	-2	78.917	10.895
ct92-M305Pete-12	120362	23/09/2012	-2	78.916	10.87
ct92-M305Pete-12	120362	23/09/2012	-2	78.919	10.933
ct92-M305Pete-12	120362	23/09/2012	-2	78.919	10.92
ct92-M305Pete-12	120362	23/09/2012	-2	78.928	10.878
ct92-M305Pete-12	120362	23/09/2012	-2	78.914	10.851
ct92-M305Pete-12	120362	23/09/2012	-2	78.915	10.86
ct92-M305Pete-12	120362	23/09/2012	0	78.905	10.76
ct92-M305Pete-12	120362	23/09/2012	0	78.903	10.769
ct92-M305Pete-12	120362	23/09/2012	-2	78.904	10.761
ct92-M305Pete-12	120362	23/09/2012	-2	78.922	10.759
ct92-M305Pete-12	120362	23/09/2012	-2	78.922	10.783
ct92-M305Pete-12	120362	23/09/2012	-2	78.921	10.704
ct92-M305Pete-12	120362	23/09/2012	-2	78.925	10.819
ct92-M305Pete-12	120362	23/09/2012	-2	78.922	10.815
ct92-M305Pete-12	120362	23/09/2012	-2	78.92	10.823
ct92-M305Pete-12	120362	23/09/2012	-2	78.926	10.821
ct92-M305Pete-12	120362	23/09/2012	-1	78.938	10.763
ct92-M305Pete-12	120362	23/09/2012	1	78.927	10.835
ct92-M305Pete-12	120362	23/09/2012	-2	78.921	10.823

ct92-M305Pete-12	120362	23/09/2012	1	78.916	10.814
ct92-M305Pete-12	120362	23/09/2012	0	78.917	10.78
ct92-M305Pete-12	120362	23/09/2012	-2	78.922	10.802
ct92-M305Pete-12	120362	23/09/2012	-1	78.928	10.813
ct92-M305Pete-12	120362	23/09/2012	-2	78.928	10.81
ct92-M305Pete-12	120362	23/09/2012	-1	78.922	10.785
ct92-M305Pete-12	120362	23/09/2012	-1	78.921	10.852
ct92-M305Pete-12	120362	23/09/2012	-2	78.922	10.8
ct92-M305Pete-12	120362	23/09/2012	1	78.934	10.769
ct92-M305Pete-12	120362	23/09/2012	-2	78.924	10.795
ct92-M305Pete-12	120362	23/09/2012	-1	78.926	10.754
ct92-M305Pete-12	120362	23/09/2012	-2	78.927	10.768
ct92-M305Pete-12	120362	23/09/2012	-2	78.915	10.492
ct92-M305Pete-12	120362	23/09/2012	-2	78.926	10.479
ct92-M305Pete-12	120362	23/09/2012	-2	78.934	10.604
ct92-M305Pete-12	120362	23/09/2012	-2	78.944	10.659
ct92-M305Pete-12	120362	23/09/2012	-2	78.927	10.654
ct92-M305Pete-12	120362	23/09/2012	-1	78.932	10.661
ct92-M305Pete-12	120362	23/09/2012	-2	78.934	10.608
ct92-M305Pete-12	120362	24/09/2012	-2	78.93	10.67
ct92-M305Pete-12	120362	24/09/2012	-1	78.941	10.662
ct92-M305Pete-12	120362	24/09/2012	-2	78.942	10.674
ct92-M305Pete-12	120362	24/09/2012	-2	78.943	10.676
ct92-M305Pete-12	120362	24/09/2012	-2	78.943	10.671
ct92-M305Pete-12	120362	24/09/2012	-2	78.938	10.698
ct92-M305Pete-12	120362	24/09/2012	-2	78.941	10.666
ct92-M305Pete-12	120362	24/09/2012	-2	78.922	10.68
ct92-M305Pete-12	120362	24/09/2012	-2	78.924	10.704
ct92-M305Pete-12	120362	24/09/2012	-2	78.923	10.717
ct92-M305Pete-12	120362	24/09/2012	1	78.912	10.83
ct92-M305Pete-12	120362	24/09/2012	-2	78.926	10.795
ct92-M305Pete-12	120362	24/09/2012	-2	78.924	10.79
ct92-M305Pete-12	120362	24/09/2012	-2	78.921	10.822
ct92-M305Pete-12	120362	24/09/2012	-2	78.923	10.799
ct92-M305Pete-12	120362	24/09/2012	-2	78.923	10.809
ct92-M305Pete-12	120362	24/09/2012	-2	78.919	10.838
ct92-M305Pete-12	120362	24/09/2012	-2	78.923	10.811
ct92-M305Pete-12	120362	24/09/2012	-2	78.923	10.76
ct92-M305Pete-12	120362	24/09/2012	-1	78.997	10.722
ct92-M305Pete-12	120362	24/09/2012	-1	78.939	10.726
ct92-M305Pete-12	120362	24/09/2012	-2	78.919	10.763
ct92-M305Pete-12	120362	24/09/2012	-1	78.918	10.769
ct92-M305Pete-12	120362	24/09/2012	-2	78.923	10.736
ct92-M305Pete-12	120362	24/09/2012	-2	78.923	10.738
ct92-M305Pete-12	120362	24/09/2012	-2	78.917	10.756
ct92-M305Pete-12	120362	24/09/2012	-2	78.918	10.755
ct92-M305Pete-12	120362	24/09/2012	-1	78.919	10.748
ct92-M305Pete-12	120362	24/09/2012	-2	78.92	10.764
ct92-M305Pete-12	120362	24/09/2012	-2	78.926	10.738
ct92-M305Pete-12	120362	24/09/2012	-2	78.924	10.741
ct92-M305Pete-12	120362	24/09/2012	-2	78.92	10.729
ct92-M305Pete-12	120362	24/09/2012	-1	78.945	10.825
ct92-M305Pete-12	120362	24/09/2012	0	78.923	10.715
ct92-M305Pete-12	120362	24/09/2012	-1	78.906	10.741
ct92-M305Pete-12	120362	24/09/2012	-1	78.915	10.751
ct92-M305Pete-12	120362	24/09/2012	2	78.913	10.742
ct92-M305Pete-12	120362	24/09/2012	0	78.922	10.787
ct92-M305Pete-12	120362	24/09/2012	-1	78.902	10.709
ct92-M305Pete-12	120362	24/09/2012	0	78.906	10.81
ct92-M305Pete-12	120362	24/09/2012	-1	78.913	10.811
ct92-M305Pete-12	120362	24/09/2012	-2	78.912	10.812
ct92-M305Pete-12	120362	24/09/2012	-1	78.912	10.668
ct92-M305Pete-12	120362	24/09/2012	0	78.916	10.775
ct92-M305Pete-12	120362	24/09/2012	-2	78.911	10.787
ct92-M305Pete-12	120362	24/09/2012	-1	78.907	10.745
ct92-M305Pete-12	120362	24/09/2012	-2	78.907	10.848
ct92-M305Pete-12	120362	24/09/2012	-2	78.933	10.828
ct92-M305Pete-12	120362	24/09/2012	-1	78.939	10.915

ct92-M305Pete-12	120362	24/09/2012	2	78.929	10.696
ct92-M305Pete-12	120362	24/09/2012	-1	78.934	10.665
ct92-M305Pete-12	120362	24/09/2012	1	78.928	10.706
ct92-M305Pete-12	120362	24/09/2012	0	78.915	10.711
ct92-M305Pete-12	120362	24/09/2012	-2	78.919	10.755
ct92-M305Pete-12	120362	24/09/2012	-1	78.924	10.734
ct92-M305Pete-12	120362	24/09/2012	-2	78.924	10.721
ct92-M305Pete-12	120362	24/09/2012	-2	78.928	10.691
ct92-M305Pete-12	120362	25/09/2012	-2	78.908	10.652
ct92-M305Pete-12	120362	25/09/2012	-2	78.898	10.708
ct92-M305Pete-12	120362	25/09/2012	-2	78.894	10.672
ct92-M305Pete-12	120362	25/09/2012	-2	78.894	10.679
ct92-M305Pete-12	120362	25/09/2012	-2	78.895	10.629
ct92-M305Pete-12	120362	25/09/2012	-2	78.894	10.626
ct92-M305Pete-12	120362	25/09/2012	-2	78.897	10.612
ct92-M305Pete-12	120362	25/09/2012	-2	78.899	10.614
ct92-M305Pete-12	120362	25/09/2012	-2	78.896	10.619
ct92-M305Pete-12	120362	25/09/2012	-2	78.904	10.638
ct92-M305Pete-12	120362	25/09/2012	-2	78.931	10.597
ct92-M305Pete-12	120362	25/09/2012	-2	78.906	10.679
ct92-M305Pete-12	120362	25/09/2012	-2	78.924	10.73
ct92-M305Pete-12	120362	25/09/2012	-2	78.918	10.733
ct92-M305Pete-12	120362	25/09/2012	-2	78.924	10.728
ct92-M305Pete-12	120362	25/09/2012	-2	78.915	10.742
ct92-M305Pete-12	120362	25/09/2012	-2	78.92	10.773
ct92-M305Pete-12	120362	25/09/2012	-2	78.918	10.788
ct92-M305Pete-12	120362	25/09/2012	-2	78.915	10.808
ct92-M305Pete-12	120362	25/09/2012	-2	78.916	10.833
ct92-M305Pete-12	120362	25/09/2012	-2	78.92	10.87
ct92-M305Pete-12	120362	25/09/2012	-2	78.918	10.847
ct92-M305Pete-12	120362	25/09/2012	-2	78.906	10.918
ct92-M305Pete-12	120362	25/09/2012	-2	78.898	10.867
ct92-M305Pete-12	120362	25/09/2012	-1	78.903	10.85
ct92-M305Pete-12	120362	25/09/2012	-2	78.899	10.884
ct92-M305Pete-12	120362	25/09/2012	-2	78.906	10.866
ct92-M305Pete-12	120362	25/09/2012	-2	78.914	10.781
ct92-M305Pete-12	120362	25/09/2012	-2	78.911	10.761
ct92-M305Pete-12	120362	25/09/2012	-2	78.908	10.736
ct92-M305Pete-12	120362	25/09/2012	0	78.911	10.763
ct92-M305Pete-12	120362	25/09/2012	-1	78.915	10.745
ct92-M305Pete-12	120362	25/09/2012	-2	78.916	10.752
ct92-M305Pete-12	120362	25/09/2012	-2	78.917	10.755
ct92-M305Pete-12	120362	25/09/2012	-2	78.917	10.754
ct92-M305Pete-12	120362	25/09/2012	-2	78.917	10.752
ct92-M305Pete-12	120362	25/09/2012	-2	78.917	10.752
ct92-M305Pete-12	120362	25/09/2012	1	78.935	10.748
ct92-M305Pete-12	120362	25/09/2012	-1	78.924	10.749
ct92-M305Pete-12	120362	25/09/2012	-2	78.935	10.689
ct92-M305Pete-12	120362	25/09/2012	-2	78.927	10.795
ct92-M305Pete-12	120362	25/09/2012	-2	78.933	10.77
ct92-M305Pete-12	120362	25/09/2012	-2	78.929	10.757
ct92-M305Pete-12	120362	25/09/2012	-2	78.925	10.755
ct92-M305Pete-12	120362	25/09/2012	-2	78.924	10.742
ct92-M305Pete-12	120362	25/09/2012	-2	78.926	10.714
ct92-M305Pete-12	120362	25/09/2012	-2	78.925	10.764
ct92-M305Pete-12	120362	25/09/2012	-2	78.922	10.748
ct92-M305Pete-12	120362	25/09/2012	-2	78.924	10.769
ct92-M305Pete-12	120362	25/09/2012	-2	78.92	10.778
ct92-M305Pete-12	120362	25/09/2012	-2	78.919	10.771
ct92-M305Pete-12	120362	25/09/2012	1	78.918	10.809
ct92-M305Pete-12	120362	25/09/2012	-2	78.929	10.784
ct92-M305Pete-12	120362	25/09/2012	-2	78.924	10.796
ct92-M305Pete-12	120362	25/09/2012	-2	78.936	10.799
ct92-M305Pete-12	120362	25/09/2012	-1	78.936	10.855
ct92-M305Pete-12	120362	25/09/2012	-2	78.942	10.8
ct92-M305Pete-12	120362	25/09/2012	-2	78.937	10.889
ct92-M305Pete-12	120362	25/09/2012	-2	78.925	10.739
ct92-M305Pete-12	120362	25/09/2012	1	78.922	10.735

ct92-M305Pete-12	120362	25/09/2012	-2	78.935	10.893
ct92-M305Pete-12	120362	25/09/2012	-1	78.944	10.937
ct92-M305Pete-12	120362	25/09/2012	-2	78.944	10.928
ct92-M305Pete-12	120362	25/09/2012	-2	78.943	10.917
ct92-M305Pete-12	120362	25/09/2012	-2	78.936	10.899
ct92-M305Pete-12	120362	25/09/2012	-1	78.929	10.818
ct92-M305Pete-12	120362	25/09/2012	-2	78.924	10.676
ct92-M305Pete-12	120362	26/09/2012	-2	78.952	10.806
ct92-M305Pete-12	120362	26/09/2012	-2	78.957	10.827
ct92-M305Pete-12	120362	26/09/2012	-2	78.942	10.763
ct92-M305Pete-12	120362	26/09/2012	-2	78.955	10.851
ct92-M305Pete-12	120362	26/09/2012	-2	78.939	10.722
ct92-M305Pete-12	120362	26/09/2012	-2	78.954	10.853
ct92-M305Pete-12	120362	26/09/2012	-2	78.933	10.781
ct92-M305Pete-12	120362	26/09/2012	-2	78.949	10.764
ct92-M305Pete-12	120362	26/09/2012	-2	78.908	10.791
ct92-M305Pete-12	120362	26/09/2012	0	78.91	10.806
ct92-M305Pete-12	120362	26/09/2012	-1	78.934	10.739
ct92-M305Pete-12	120362	26/09/2012	-1	78.94	10.67
ct92-M305Pete-12	120362	26/09/2012	-2	78.907	10.799
ct92-M305Pete-12	120362	26/09/2012	-2	78.919	10.801
ct92-M305Pete-12	120362	26/09/2012	-1	78.924	10.774
ct92-M305Pete-12	120362	26/09/2012	-1	78.924	10.787
ct92-M305Pete-12	120362	26/09/2012	-2	78.916	10.769
ct92-M305Pete-12	120362	26/09/2012	-1	78.912	10.804
ct92-M305Pete-12	120362	26/09/2012	-2	78.914	10.8
ct92-M305Pete-12	120362	26/09/2012	-1	78.913	10.844
ct92-M305Pete-12	120362	26/09/2012	1	78.908	10.8
ct92-M305Pete-12	120362	26/09/2012	1	78.912	10.773
ct92-M305Pete-12	120362	26/09/2012	-1	78.906	10.856
ct92-M305Pete-12	120362	26/09/2012	-2	78.92	10.804
ct92-M305Pete-12	120362	26/09/2012	-2	78.915	10.769
ct92-M305Pete-12	120362	26/09/2012	1	78.924	10.82
ct92-M305Pete-12	120362	26/09/2012	-2	78.914	10.784
ct92-M305Pete-12	120362	26/09/2012	-2	78.926	10.837
ct92-M305Pete-12	120362	26/09/2012	-2	78.911	10.823
ct92-M305Pete-12	120362	26/09/2012	-1	78.916	10.834
ct92-M305Pete-12	120362	26/09/2012	2	78.902	10.818
ct92-M305Pete-12	120362	26/09/2012	-1	78.906	10.831
ct92-M305Pete-12	120362	26/09/2012	-2	78.905	10.799
ct92-M305Pete-12	120362	26/09/2012	-1	78.905	10.799
ct92-M305Pete-12	120362	26/09/2012	-2	78.91	10.831
ct92-M305Pete-12	120362	26/09/2012	-2	78.901	10.803
ct92-M305Pete-12	120362	26/09/2012	-2	78.903	10.801
ct92-M305Pete-12	120362	26/09/2012	0	78.896	10.878
ct92-M305Pete-12	120362	26/09/2012	-2	78.899	10.818
ct92-M305Pete-12	120362	26/09/2012	-2	78.898	10.864
ct92-M305Pete-12	120362	26/09/2012	-2	78.899	10.865
ct92-M305Pete-12	120362	26/09/2012	0	78.875	10.756
ct92-M305Pete-12	120362	26/09/2012	-2	78.92	10.785
ct92-M305Pete-12	120362	26/09/2012	2	78.912	10.798
ct92-M305Pete-12	120362	26/09/2012	-2	78.906	10.896
ct92-M305Pete-12	120362	26/09/2012	0	78.889	10.695
ct92-M305Pete-12	120362	26/09/2012	-2	78.834	10.479
ct92-M305Pete-12	120362	26/09/2012	1	78.908	10.759
ct92-M305Pete-12	120362	26/09/2012	-2	78.914	10.798
ct92-M305Pete-12	120362	26/09/2012	-1	78.92	10.763
ct92-M305Pete-12	120362	26/09/2012	-1	78.928	10.762
ct92-M305Pete-12	120362	26/09/2012	-2	78.929	10.77
ct92-M305Pete-12	120362	26/09/2012	-2	78.937	10.788
ct92-M305Pete-12	120362	26/09/2012	-2	78.932	10.809
ct92-M305Pete-12	120362	26/09/2012	-1	78.883	10.523
ct92-M305Pete-12	120362	26/09/2012	-1	78.923	10.719
ct92-M305Pete-12	120362	26/09/2012	-1	78.911	10.725
ct92-M305Pete-12	120362	26/09/2012	-2	78.91	10.723
ct92-M305Pete-12	120362	26/09/2012	1	78.887	10.707
ct92-M305Pete-12	120362	26/09/2012	2	78.887	10.725
ct92-M305Pete-12	120362	26/09/2012	-2	78.883	10.7

ct92-M305Pete-12	120362	26/09/2012	-2	78.88	10.71
ct92-M305Pete-12	120362	27/09/2012	0	78.868	10.749
ct92-M305Pete-12	120362	27/09/2012	-2	78.859	10.723
ct92-M305Pete-12	120362	27/09/2012	-2	78.863	10.732
ct92-M305Pete-12	120362	27/09/2012	-2	78.824	10.647
ct92-M305Pete-12	120362	27/09/2012	2	78.884	10.711
ct92-M305Pete-12	120362	27/09/2012	3	78.891	10.737
ct92-M305Pete-12	120362	27/09/2012	-2	78.891	10.737
ct92-M305Pete-12	120362	27/09/2012	-2	78.891	10.745
ct92-M305Pete-12	120362	27/09/2012	-2	78.893	10.748
ct92-M305Pete-12	120362	27/09/2012	-2	78.892	10.748
ct92-M305Pete-12	120362	27/09/2012	-1	78.878	10.793
ct92-M305Pete-12	120362	27/09/2012	-2	78.898	10.797
ct92-M305Pete-12	120362	27/09/2012	-2	78.902	10.793
ct92-M305Pete-12	120362	27/09/2012	-2	78.916	10.749
ct92-M305Pete-12	120362	27/09/2012	-2	78.917	10.788
ct92-M305Pete-12	120362	27/09/2012	-1	78.907	10.713
ct92-M305Pete-12	120362	27/09/2012	-2	78.903	10.702
ct92-M305Pete-12	120362	27/09/2012	-2	78.901	10.727
ct92-M305Pete-12	120362	27/09/2012	-2	78.903	10.699
ct92-M305Pete-12	120362	27/09/2012	-2	78.907	10.704
ct92-M305Pete-12	120362	27/09/2012	-2	78.908	10.717
ct92-M305Pete-12	120362	27/09/2012	-2	78.907	10.694
ct92-M305Pete-12	120362	27/09/2012	-1	78.929	10.692
ct92-M305Pete-12	120362	27/09/2012	1	78.929	10.675
ct92-M305Pete-12	120362	27/09/2012	-2	78.933	10.63
ct92-M305Pete-12	120362	27/09/2012	-2	78.928	10.665
ct92-M305Pete-12	120362	27/09/2012	-1	78.922	10.738
ct92-M305Pete-12	120362	27/09/2012	-1	78.934	10.71
ct92-M305Pete-12	120362	27/09/2012	-2	78.917	10.726
ct92-M305Pete-12	120362	27/09/2012	-2	78.917	10.696
ct92-M305Pete-12	120362	27/09/2012	-2	78.932	10.668
ct92-M305Pete-12	120362	27/09/2012	-2	78.929	10.66
ct92-M305Pete-12	120362	27/09/2012	-2	78.929	10.655
ct92-M305Pete-12	120362	27/09/2012	-2	78.944	10.728
ct92-M305Pete-12	120362	27/09/2012	-2	78.935	10.68
ct92-M305Pete-12	120362	27/09/2012	-2	78.935	10.68
ct92-M305Pete-12	120362	27/09/2012	-2	78.934	10.686
ct92-M305Pete-12	120362	27/09/2012	-2	78.938	10.709
ct92-M305Pete-12	120362	27/09/2012	-2	78.939	10.718
ct92-M305Pete-12	120362	27/09/2012	-2	78.932	10.621
ct92-M305Pete-12	120362	27/09/2012	-2	78.939	10.735
ct92-M305Pete-12	120362	27/09/2012	-2	78.923	10.748
ct92-M305Pete-12	120362	27/09/2012	-1	78.927	10.786
ct92-M305Pete-12	120362	27/09/2012	-2	78.928	10.776
ct92-M305Pete-12	120362	27/09/2012	-2	78.954	10.802
ct92-M305Pete-12	120362	27/09/2012	-2	78.928	10.749
ct92-M305Pete-12	120362	27/09/2012	-2	78.929	10.758
ct92-M305Pete-12	120362	27/09/2012	-2	78.929	10.762
ct92-M305Pete-12	120362	27/09/2012	-1	78.937	10.759
ct92-M305Pete-12	120362	27/09/2012	-2	78.95	10.748
ct92-M305Pete-12	120362	27/09/2012	-2	78.949	10.751
ct92-M305Pete-12	120362	27/09/2012	-2	78.948	10.745
ct92-M305Pete-12	120362	27/09/2012	-1	78.937	10.525
ct92-M305Pete-12	120362	27/09/2012	-1	78.927	10.575
ct92-M305Pete-12	120362	27/09/2012	2	78.924	10.667
ct92-M305Pete-12	120362	27/09/2012	-2	78.933	10.603
ct92-M305Pete-12	120362	27/09/2012	-2	78.937	10.612
ct92-M305Pete-12	120362	27/09/2012	-2	78.945	10.621
ct92-M305Pete-12	120362	27/09/2012	-2	78.929	10.596
ct92-M305Pete-12	120362	27/09/2012	-2	78.919	10.65
ct92-M305Pete-12	120362	27/09/2012	-1	78.92	10.576
ct92-M305Pete-12	120362	27/09/2012	-2	78.919	10.611
ct92-M305Pete-12	120362	27/09/2012	-2	78.927	10.643
ct92-M305Pete-12	120362	27/09/2012	-1	78.922	10.644
ct92-M305Pete-12	120362	27/09/2012	-2	78.923	10.637
ct92-M305Pete-12	120362	28/09/2012	-1	78.928	10.547
ct92-M305Pete-12	120362	28/09/2012	-2	78.928	10.549

ct92-M305Pete-12	120362	28/09/2012	-2	78.928	10.549
ct92-M305Pete-12	120362	28/09/2012	-2	78.933	10.566
ct92-M305Pete-12	120362	28/09/2012	-2	78.927	10.593
ct92-M305Pete-12	120362	28/09/2012	2	78.926	10.73
ct92-M305Pete-12	120362	28/09/2012	-2	78.928	10.705
ct92-M305Pete-12	120362	28/09/2012	-2	78.938	10.74
ct92-M305Pete-12	120362	28/09/2012	-2	78.93	10.763
ct92-M305Pete-12	120362	28/09/2012	-1	78.928	10.695
ct92-M305Pete-12	120362	28/09/2012	-2	78.91	10.716
ct92-M305Pete-12	120362	28/09/2012	-2	78.91	10.721
ct92-M305Pete-12	120362	28/09/2012	-2	78.929	10.708
ct92-M305Pete-12	120362	28/09/2012	-2	78.93	10.701
ct92-M305Pete-12	120362	28/09/2012	-1	78.926	10.61
ct92-M305Pete-12	120362	28/09/2012	-2	78.922	10.617
ct92-M305Pete-12	120362	28/09/2012	-1	78.916	10.706
ct92-M305Pete-12	120362	28/09/2012	-2	78.895	10.651
ct92-M305Pete-12	120362	28/09/2012	-2	78.915	10.705
ct92-M305Pete-12	120362	28/09/2012	-2	78.895	10.679
ct92-M305Pete-12	120362	28/09/2012	-1	78.919	10.521
ct92-M305Pete-12	120362	28/09/2012	-2	78.895	10.628
ct92-M305Pete-12	120362	28/09/2012	-2	78.918	10.527
ct92-M305Pete-12	120362	28/09/2012	-1	78.912	10.571
ct92-M305Pete-12	120362	28/09/2012	-2	78.919	10.596
ct92-M305Pete-12	120362	28/09/2012	-2	78.906	10.581
ct92-M305Pete-12	120362	28/09/2012	1	78.908	10.559
ct92-M305Pete-12	120362	28/09/2012	-2	78.906	10.573
ct92-M305Pete-12	120362	28/09/2012	0	78.924	10.6
ct92-M305Pete-12	120362	28/09/2012	-1	78.924	10.646
ct92-M305Pete-12	120362	28/09/2012	-1	78.912	10.607
ct92-M305Pete-12	120362	28/09/2012	-1	78.918	10.681
ct92-M305Pete-12	120362	28/09/2012	-2	78.919	10.67
ct92-M305Pete-12	120362	28/09/2012	-1	78.921	10.72
ct92-M305Pete-12	120362	28/09/2012	-2	78.916	10.718
ct92-M305Pete-12	120362	28/09/2012	-2	78.924	10.7
ct92-M305Pete-12	120362	28/09/2012	-2	78.926	10.689
ct92-M305Pete-12	120362	28/09/2012	-2	78.926	10.685
ct92-M305Pete-12	120362	28/09/2012	-1	78.926	10.712
ct92-M305Pete-12	120362	28/09/2012	-2	78.923	10.728
ct92-M305Pete-12	120362	28/09/2012	-1	78.92	10.711
ct92-M305Pete-12	120362	28/09/2012	-1	78.921	10.717
ct92-M305Pete-12	120362	28/09/2012	-1	78.914	10.682
ct92-M305Pete-12	120362	28/09/2012	-2	78.927	10.753
ct92-M305Pete-12	120362	28/09/2012	-1	78.912	10.683
ct92-M305Pete-12	120362	28/09/2012	-2	78.925	10.77
ct92-M305Pete-12	120362	28/09/2012	1	78.916	10.818
ct92-M305Pete-12	120362	28/09/2012	3	78.91	10.827
ct92-M305Pete-12	120362	28/09/2012	-2	78.912	10.823
ct92-M305Pete-12	120362	28/09/2012	1	78.918	10.756
ct92-M305Pete-12	120362	28/09/2012	-2	78.915	10.771
ct92-M305Pete-12	120362	28/09/2012	-2	78.918	10.762
ct92-M305Pete-12	120362	28/09/2012	-1	78.927	10.773
ct92-M305Pete-12	120362	28/09/2012	-2	78.927	10.768
ct92-M305Pete-12	120362	28/09/2012	-2	78.926	10.771
ct92-M305Pete-12	120362	28/09/2012	-2	78.929	10.778
ct92-M305Pete-12	120362	28/09/2012	-2	78.926	10.695
ct92-M305Pete-12	120362	28/09/2012	-1	78.921	10.7
ct92-M305Pete-12	120362	28/09/2012	-2	78.954	10.736
ct92-M305Pete-12	120362	28/09/2012	-2	78.943	10.666
ct92-M305Pete-12	120362	28/09/2012	-1	78.924	10.714
ct92-M305Pete-12	120362	28/09/2012	-2	78.921	10.705
ct92-M305Pete-12	120362	28/09/2012	-2	78.924	10.701
ct92-M305Pete-12	120362	28/09/2012	-1	78.945	10.586
ct92-M305Pete-12	120362	28/09/2012	-2	78.948	10.578
ct92-M305Pete-12	120362	28/09/2012	-2	78.949	10.572
ct92-M305Pete-12	120362	28/09/2012	-2	78.925	10.635
ct92-M305Pete-12	120362	29/09/2012	-2	78.977	10.501
ct92-M305Pete-12	120362	29/09/2012	-2	78.966	10.521
ct92-M305Pete-12	120362	29/09/2012	-2	78.938	10.517

ct92-M305Pete-12	120362	29/09/2012	-2	78.977	10.458
ct92-M305Pete-12	120362	29/09/2012	-2	78.955	10.505
ct92-M305Pete-12	120362	29/09/2012	-2	78.977	10.442
ct92-M305Pete-12	120362	29/09/2012	-2	78.955	10.552
ct92-M305Pete-12	120362	29/09/2012	-2	78.977	10.512
ct92-M305Pete-12	120362	29/09/2012	-2	78.972	10.568
ct92-M305Pete-12	120362	29/09/2012	-1	78.932	10.647
ct92-M305Pete-12	120362	29/09/2012	-2	78.932	10.655
ct92-M305Pete-12	120362	29/09/2012	-2	78.966	10.573
ct92-M305Pete-12	120362	29/09/2012	-2	78.932	10.663
ct92-M305Pete-12	120362	29/09/2012	-2	78.962	10.586
ct92-M305Pete-12	120362	29/09/2012	-1	78.896	10.667
ct92-M305Pete-12	120362	29/09/2012	-1	78.906	10.649
ct92-M305Pete-12	120362	29/09/2012	-1	78.899	10.689
ct92-M305Pete-12	120362	29/09/2012	-2	78.914	10.744
ct92-M305Pete-12	120362	29/09/2012	-2	78.907	10.75
ct92-M305Pete-12	120362	29/09/2012	-2	78.913	10.799
ct92-M305Pete-12	120362	29/09/2012	-2	78.936	10.661
ct92-M305Pete-12	120362	29/09/2012	-2	78.859	10.687
ct92-M305Pete-12	120362	29/09/2012	-2	78.936	10.689
ct92-M305Pete-12	120362	29/09/2012	-2	78.906	10.807
ct92-M305Pete-12	120362	29/09/2012	1	78.912	10.771
ct92-M305Pete-12	120362	29/09/2012	-2	78.918	10.795
ct92-M305Pete-12	120362	29/09/2012	-2	78.904	10.774
ct92-M305Pete-12	120362	29/09/2012	-2	78.903	10.798
ct92-M305Pete-12	120362	29/09/2012	-2	78.902	10.798
ct92-M305Pete-12	120362	29/09/2012	-2	78.904	10.8
ct92-M305Pete-12	120362	29/09/2012	1	78.906	10.791
ct92-M305Pete-12	120362	29/09/2012	1	78.905	10.792
ct92-M305Pete-12	120362	29/09/2012	-1	78.913	10.808
ct92-M305Pete-12	120362	29/09/2012	1	78.917	10.761
ct92-M305Pete-12	120362	29/09/2012	-1	78.913	10.742
ct92-M305Pete-12	120362	29/09/2012	-2	78.926	10.725
ct92-M305Pete-12	120362	29/09/2012	-2	78.922	10.729
ct92-M305Pete-12	120362	29/09/2012	-1	78.92	10.729
ct92-M305Pete-12	120362	29/09/2012	-2	78.921	10.768
ct92-M305Pete-12	120362	29/09/2012	-2	78.921	10.771
ct92-M305Pete-12	120362	29/09/2012	-2	78.918	10.746
ct92-M305Pete-12	120362	29/09/2012	-2	78.915	10.758
ct92-M305Pete-12	120362	29/09/2012	-2	78.936	10.669
ct92-M305Pete-12	120362	29/09/2012	-2	78.923	10.746
ct92-M305Pete-12	120362	29/09/2012	-2	78.921	10.741
ct92-M305Pete-12	120362	29/09/2012	-2	78.911	10.691
ct92-M305Pete-12	120362	29/09/2012	-2	78.91	10.764
ct92-M305Pete-12	120362	29/09/2012	-1	78.924	10.708
ct92-M305Pete-12	120362	29/09/2012	-2	78.923	10.733
ct92-M305Pete-12	120362	29/09/2012	-2	78.923	10.743
ct92-M305Pete-12	120362	29/09/2012	-1	78.919	10.678
ct92-M305Pete-12	120362	29/09/2012	-1	78.93	10.76
ct92-M305Pete-12	120362	29/09/2012	-2	78.928	10.77
ct92-M305Pete-12	120362	29/09/2012	1	78.926	10.754
ct92-M305Pete-12	120362	29/09/2012	-2	78.928	10.77
ct92-M305Pete-12	120362	29/09/2012	-2	78.941	10.762
ct92-M305Pete-12	120362	29/09/2012	-1	78.928	10.749
ct92-M305Pete-12	120362	29/09/2012	-2	78.928	10.753
ct92-M305Pete-12	120362	29/09/2012	0	78.926	10.751
ct92-M305Pete-12	120362	29/09/2012	-2	78.926	10.744
ct92-M305Pete-12	120362	29/09/2012	-1	78.91	10.681
ct92-M305Pete-12	120362	29/09/2012	1	78.923	10.691
ct92-M305Pete-12	120362	29/09/2012	-1	78.934	10.72
ct92-M305Pete-12	120362	30/09/2012	-2	78.933	10.615
ct92-M305Pete-12	120362	30/09/2012	-2	78.932	10.609
ct92-M305Pete-12	120362	30/09/2012	-2	78.94	10.662
ct92-M305Pete-12	120362	30/09/2012	-1	78.95	10.595
ct92-M305Pete-12	120362	30/09/2012	-2	78.944	10.576
ct92-M305Pete-12	120362	30/09/2012	-2	78.936	10.684
ct92-M305Pete-12	120362	30/09/2012	1	78.932	10.642
ct92-M305Pete-12	120362	30/09/2012	-2	78.932	10.644

ct92-M305Pete-12	120362	30/09/2012	-1	78.918	10.644
ct92-M305Pete-12	120362	30/09/2012	-2	78.931	10.629
ct92-M305Pete-12	120362	30/09/2012	-2	78.93	10.619
ct92-M305Pete-12	120362	30/09/2012	-2	78.928	10.686
ct92-M305Pete-12	120362	30/09/2012	-2	78.941	10.605
ct92-M305Pete-12	120362	30/09/2012	-2	78.931	10.668
ct92-M305Pete-12	120362	30/09/2012	-2	78.935	10.635
ct92-M305Pete-12	120362	30/09/2012	1	78.924	10.674
ct92-M305Pete-12	120362	30/09/2012	-2	78.925	10.675
ct92-M305Pete-12	120362	30/09/2012	0	78.938	10.616
ct92-M305Pete-12	120362	30/09/2012	-2	78.924	10.573
ct92-M305Pete-12	120362	30/09/2012	-2	78.935	10.575
ct92-M305Pete-12	120362	30/09/2012	-1	78.931	10.543
ct92-M305Pete-12	120362	30/09/2012	-1	78.929	10.611
ct92-M305Pete-12	120362	30/09/2012	-2	78.897	10.571
ct92-M305Pete-12	120362	30/09/2012	-1	78.893	10.507
ct92-M305Pete-12	120362	30/09/2012	-2	78.898	10.561
ct92-M305Pete-12	120362	30/09/2012	-2	78.898	10.564
ct92-M305Pete-12	120362	30/09/2012	-2	78.898	10.48
ct92-M305Pete-12	120362	30/09/2012	-1	78.906	10.525
ct92-M305Pete-12	120362	30/09/2012	1	78.916	10.605
ct92-M305Pete-12	120362	30/09/2012	-2	78.915	10.611
ct92-M305Pete-12	120362	30/09/2012	-1	78.92	10.628
ct92-M305Pete-12	120362	30/09/2012	-2	78.904	10.542
ct92-M305Pete-12	120362	30/09/2012	-2	78.908	10.625
ct92-M305Pete-12	120362	30/09/2012	-1	78.923	10.709
ct92-M305Pete-12	120362	30/09/2012	-2	78.903	10.612
ct92-M305Pete-12	120362	30/09/2012	2	78.917	10.647
ct92-M305Pete-12	120362	30/09/2012	-2	78.915	10.648
ct92-M305Pete-12	120362	30/09/2012	-1	78.912	10.637
ct92-M305Pete-12	120362	30/09/2012	-2	78.911	10.635
ct92-M305Pete-12	120362	30/09/2012	-1	78.895	10.691
ct92-M305Pete-12	120362	30/09/2012	-2	78.913	10.651
ct92-M305Pete-12	120362	30/09/2012	-2	78.885	10.634
ct92-M305Pete-12	120362	30/09/2012	-2	78.902	10.597
ct92-M305Pete-12	120362	30/09/2012	-2	78.896	10.629
ct92-M305Pete-12	120362	30/09/2012	-2	78.904	10.642
ct92-M305Pete-12	120362	30/09/2012	-2	78.899	10.643
ct92-M305Pete-12	120362	30/09/2012	-2	78.886	10.631
ct92-M305Pete-12	120362	30/09/2012	-2	78.9	10.727
ct92-M305Pete-12	120362	30/09/2012	-2	78.899	10.728
ct92-M305Pete-12	120362	30/09/2012	-2	78.904	10.724
ct92-M305Pete-12	120362	30/09/2012	-2	78.925	10.692
ct92-M305Pete-12	120362	30/09/2012	-2	78.901	10.723
ct92-M305Pete-12	120362	30/09/2012	-2	78.9	10.716
ct92-M305Pete-12	120362	30/09/2012	-2	78.937	10.717
ct92-M305Pete-12	120362	30/09/2012	-2	78.94	10.715
ct92-M305Pete-12	120362	30/09/2012	-2	78.935	10.715
ct92-M305Pete-12	120362	30/09/2012	-1	78.915	10.631
ct92-M305Pete-12	120362	30/09/2012	-2	78.914	10.629
ct92-M305Pete-12	120362	30/09/2012	-2	78.929	10.648
ct92-M305Pete-12	120362	30/09/2012	-2	78.93	10.645
ct92-M305Pete-12	120362	30/09/2012	-2	78.929	10.666
ct92-M305Pete-12	120362	30/09/2012	-2	78.926	10.73
ct92-M305Pete-12	120362	30/09/2012	-2	78.921	10.606
ct92-M305Pete-12	120362	30/09/2012	-2	78.93	10.693
ct92-M305Pete-12	120362	30/09/2012	-1	78.927	10.679
ct92-M305Pete-12	120362	30/09/2012	-2	78.927	10.679
ct92-M305Pete-12	120362	30/09/2012	-1	78.934	10.638
ct92-M305Pete-12	120362	30/09/2012	-2	78.93	10.628
ct92-M305Pete-12	120362	01/10/2012	-2	78.918	10.626
ct92-M305Pete-12	120362	01/10/2012	-2	78.918	10.629
ct92-M305Pete-12	120362	01/10/2012	-2	78.927	10.612
ct92-M305Pete-12	120362	01/10/2012	-2	78.927	10.612
ct92-M305Pete-12	120362	01/10/2012	-1	78.965	10.577
ct92-M305Pete-12	120362	01/10/2012	-2	78.973	10.553
ct92-M305Pete-12	120362	01/10/2012	-2	78.972	10.525
ct92-M305Pete-12	120362	01/10/2012	-1	78.943	10.21

ct92-M305Pete-12	120362	01/10/2012	-2	78.973	10.578
ct92-M305Pete-12	120362	01/10/2012	-2	78.97	10.578
ct92-M305Pete-12	120362	01/10/2012	-2	78.97	10.509
ct92-M305Pete-12	120362	01/10/2012	-2	78.988	10.572
ct92-M305Pete-12	120362	01/10/2012	-2	79.016	10.511
ct92-M305Pete-12	120362	01/10/2012	-2	79.028	10.43
ct92-M305Pete-12	120362	01/10/2012	-2	78.995	10.676
ct92-M305Pete-12	120362	01/10/2012	-2	79.041	10.443
ct92-M305Pete-12	120362	01/10/2012	-2	78.957	10.803
ct92-M305Pete-12	120362	01/10/2012	-2	78.988	10.799
ct92-M305Pete-12	120362	01/10/2012	-2	78.942	10.794
ct92-M305Pete-12	120362	01/10/2012	-2	78.961	10.776
ct92-M305Pete-12	120362	01/10/2012	-1	78.929	10.837
ct92-M305Pete-12	120362	01/10/2012	-1	78.913	10.835
ct92-M305Pete-12	120362	01/10/2012	-1	78.91	10.799
ct92-M305Pete-12	120362	01/10/2012	-2	78.915	10.806
ct92-M305Pete-12	120362	01/10/2012	-1	78.905	10.826
ct92-M305Pete-12	120362	01/10/2012	0	78.893	10.828
ct92-M305Pete-12	120362	01/10/2012	-2	78.9	10.825
ct92-M305Pete-12	120362	01/10/2012	-2	78.899	10.83
ct92-M305Pete-12	120362	01/10/2012	-2	78.93	10.774
ct92-M305Pete-12	120362	01/10/2012	-1	78.93	10.761
ct92-M305Pete-12	120362	01/10/2012	-1	78.915	10.801
ct92-M305Pete-12	120362	01/10/2012	-2	78.914	10.841
ct92-M305Pete-12	120362	01/10/2012	-2	78.912	10.888
ct92-M305Pete-12	120362	01/10/2012	-2	78.912	10.889
ct92-M305Pete-12	120362	01/10/2012	-2	78.907	10.877
ct92-M305Pete-12	120362	01/10/2012	-2	78.898	10.74
ct92-M305Pete-12	120362	01/10/2012	-2	78.906	10.786
ct92-M305Pete-12	120362	01/10/2012	-2	78.899	10.75
ct92-M305Pete-12	120362	01/10/2012	-2	78.924	10.757
ct92-M305Pete-12	120362	01/10/2012	-2	78.901	10.716
ct92-M305Pete-12	120362	01/10/2012	-2	78.901	10.716
ct92-M305Pete-12	120362	01/10/2012	-1	78.889	10.715
ct92-M305Pete-12	120362	01/10/2012	-2	78.907	10.769
ct92-M305Pete-12	120362	01/10/2012	-2	78.903	10.763
ct92-M305Pete-12	120362	01/10/2012	-2	78.905	10.767
ct92-M305Pete-12	120362	01/10/2012	-2	78.896	10.715
ct92-M305Pete-12	120362	01/10/2012	-1	78.902	10.734
ct92-M305Pete-12	120362	01/10/2012	-1	78.896	10.733
ct92-M305Pete-12	120362	01/10/2012	-2	78.897	10.7
ct92-M305Pete-12	120362	01/10/2012	-2	78.897	10.694
ct92-M305Pete-12	120362	01/10/2012	-2	78.917	10.686
ct92-M305Pete-12	120362	01/10/2012	-2	78.919	10.673
ct92-M305Pete-12	120362	01/10/2012	-2	78.919	10.668
ct92-M305Pete-12	120362	01/10/2012	-2	78.917	10.645
ct92-M305Pete-12	120362	01/10/2012	-2	78.919	10.625
ct92-M305Pete-12	120362	01/10/2012	-2	78.919	10.637
ct92-M305Pete-12	120362	01/10/2012	-1	78.925	10.623
ct92-M305Pete-12	120362	01/10/2012	-2	78.928	10.676
ct92-M305Pete-12	120362	01/10/2012	-2	78.929	10.673
ct92-M305Pete-12	120362	01/10/2012	1	78.924	10.731
ct92-M305Pete-12	120362	01/10/2012	-1	78.921	10.693
ct92-M305Pete-12	120362	02/10/2012	-2	78.927	10.679
ct92-M305Pete-12	120362	02/10/2012	-2	78.927	10.678
ct92-M305Pete-12	120362	02/10/2012	-2	78.925	10.687
ct92-M305Pete-12	120362	02/10/2012	-2	78.919	10.714
ct92-M305Pete-12	120362	02/10/2012	-2	78.918	10.746
ct92-M305Pete-12	120362	02/10/2012	-2	78.912	10.741
ct92-M305Pete-12	120362	02/10/2012	-2	78.936	10.772
ct92-M305Pete-12	120362	02/10/2012	-2	78.943	10.855
ct92-M305Pete-12	120362	02/10/2012	-2	78.936	10.878
ct92-M305Pete-12	120362	02/10/2012	-2	78.939	10.747
ct92-M305Pete-12	120362	02/10/2012	0	78.93	10.799
ct92-M305Pete-12	120362	02/10/2012	-2	78.924	10.766
ct92-M305Pete-12	120362	02/10/2012	-2	78.915	10.793
ct92-M305Pete-12	120362	02/10/2012	-2	78.915	10.792
ct92-M305Pete-12	120362	02/10/2012	-1	78.926	10.787

ct92-M305Pete-12	120362	02/10/2012	-1	78.915	10.798
ct92-M305Pete-12	120362	02/10/2012	-2	78.913	10.824
ct92-M305Pete-12	120362	02/10/2012	-1	78.923	10.75
ct92-M305Pete-12	120362	02/10/2012	-2	78.922	10.74
ct92-M305Pete-12	120362	02/10/2012	-2	78.922	10.786
ct92-M305Pete-12	120362	02/10/2012	-2	78.916	10.819
ct92-M305Pete-12	120362	02/10/2012	-2	78.912	10.86
ct92-M305Pete-12	120362	02/10/2012	0	78.917	10.809
ct92-M305Pete-12	120362	02/10/2012	-2	78.911	10.869
ct92-M305Pete-12	120362	02/10/2012	-2	78.906	10.88
ct92-M305Pete-12	120362	02/10/2012	-2	78.911	10.87
ct92-M305Pete-12	120362	02/10/2012	-2	78.906	10.881
ct92-M305Pete-12	120362	02/10/2012	-2	78.909	10.893
ct92-M305Pete-12	120362	02/10/2012	-2	78.89	10.86
ct92-M305Pete-12	120362	02/10/2012	-2	78.894	10.853
ct92-M305Pete-12	120362	02/10/2012	-2	78.893	10.853
ct92-M305Pete-12	120362	02/10/2012	-2	78.889	10.781
ct92-M305Pete-12	120362	02/10/2012	-2	78.897	10.762
ct92-M305Pete-12	120362	02/10/2012	0	78.905	10.761
ct92-M305Pete-12	120362	02/10/2012	0	78.918	10.767
ct92-M305Pete-12	120362	02/10/2012	-2	78.919	10.765
ct92-M305Pete-12	120362	02/10/2012	0	78.902	10.794
ct92-M305Pete-12	120362	02/10/2012	-2	78.922	10.697
ct92-M305Pete-12	120362	02/10/2012	-2	78.922	10.681
ct92-M305Pete-12	120362	02/10/2012	-2	78.933	10.685
ct92-M305Pete-12	120362	02/10/2012	-2	78.935	10.687
ct92-M305Pete-12	120362	02/10/2012	-2	78.895	10.717
ct92-M305Pete-12	120362	02/10/2012	-2	78.928	10.694
ct92-M305Pete-12	120362	02/10/2012	-2	78.929	10.679
ct92-M305Pete-12	120362	02/10/2012	-2	78.925	10.727
ct92-M305Pete-12	120362	02/10/2012	-2	78.928	10.724
ct92-M305Pete-12	120362	02/10/2012	-2	78.928	10.717
ct92-M305Pete-12	120362	02/10/2012	-2	78.924	10.775
ct92-M305Pete-12	120362	02/10/2012	-2	78.919	10.793
ct92-M305Pete-12	120362	02/10/2012	-2	78.917	10.783
ct92-M305Pete-12	120362	02/10/2012	-2	78.932	10.814
ct92-M305Pete-12	120362	02/10/2012	-2	78.934	10.772
ct92-M305Pete-12	120362	02/10/2012	-2	78.937	10.754
ct92-M305Pete-12	120362	02/10/2012	-2	78.935	10.768
ct92-M305Pete-12	120362	02/10/2012	2	78.925	10.665
ct92-M305Pete-12	120362	02/10/2012	-2	78.937	10.686
ct92-M305Pete-12	120362	02/10/2012	-2	78.91	10.681
ct92-M305Pete-12	120362	02/10/2012	-2	78.926	10.792
ct92-M305Pete-12	120362	02/10/2012	-2	78.927	10.797
ct92-M305Pete-12	120362	02/10/2012	-2	78.924	10.848
ct92-M305Pete-12	120362	02/10/2012	-1	78.912	10.793
ct92-M305Pete-12	120362	02/10/2012	-2	78.919	10.869
ct92-M305Pete-12	120362	02/10/2012	-1	78.922	10.839
ct92-M305Pete-12	120362	02/10/2012	-2	78.92	10.814
ct92-M305Pete-12	120362	02/10/2012	-2	78.923	10.805
ct92-M305Pete-12	120362	02/10/2012	-2	78.922	10.784
ct92-M305Pete-12	120362	03/10/2012	-2	78.922	10.783
ct92-M305Pete-12	120362	03/10/2012	-2	78.893	10.776
ct92-M305Pete-12	120362	03/10/2012	-2	78.887	10.783
ct92-M305Pete-12	120362	03/10/2012	-1	78.924	10.749
ct92-M305Pete-12	120362	03/10/2012	-2	78.92	10.84
ct92-M305Pete-12	120362	03/10/2012	-1	78.925	10.836
ct92-M305Pete-12	120362	03/10/2012	-2	78.932	10.817
ct92-M305Pete-12	120362	03/10/2012	-2	78.924	10.772
ct92-M305Pete-12	120362	03/10/2012	-2	78.923	10.769
ct92-M305Pete-12	120362	03/10/2012	-2	78.92	10.766
ct92-M305Pete-12	120362	03/10/2012	1	78.914	10.755
ct92-M305Pete-12	120362	03/10/2012	-2	78.91	10.746
ct92-M305Pete-12	120362	03/10/2012	-2	78.91	10.748
ct92-M305Pete-12	120362	03/10/2012	-1	78.92	10.802
ct92-M305Pete-12	120362	03/10/2012	-2	78.928	10.77
ct92-M305Pete-12	120362	03/10/2012	-1	78.908	10.795
ct92-M305Pete-12	120362	03/10/2012	-2	78.903	10.798

ct92-M305Pete-12	120362	03/10/2012	-2	78.901	10.841
ct92-M305Pete-12	120362	03/10/2012	-2	78.898	10.793
ct92-M305Pete-12	120362	03/10/2012	-2	78.9	10.793
ct92-M305Pete-12	120362	03/10/2012	-1	78.886	10.903
ct92-M305Pete-12	120362	03/10/2012	-1	78.89	10.786
ct92-M305Pete-12	120362	03/10/2012	-2	78.884	10.788
ct92-M305Pete-12	120362	03/10/2012	-1	78.881	10.802
ct92-M305Pete-12	120362	03/10/2012	-1	78.92	10.935
ct92-M305Pete-12	120362	03/10/2012	-1	78.884	10.81
ct92-M305Pete-12	120362	03/10/2012	1	78.895	10.892
ct92-M305Pete-12	120362	03/10/2012	1	78.898	10.887
ct92-M305Pete-12	120362	03/10/2012	-1	78.882	10.884
ct92-M305Pete-12	120362	03/10/2012	-1	78.881	10.912
ct92-M305Pete-12	120362	03/10/2012	1	78.873	10.873
ct92-M305Pete-12	120362	03/10/2012	-2	78.877	10.871
ct92-M305Pete-12	120362	03/10/2012	-2	78.877	10.87
ct92-M305Pete-12	120362	03/10/2012	-1	78.875	10.87
ct92-M305Pete-12	120362	03/10/2012	-2	78.876	10.819
ct92-M305Pete-12	120362	03/10/2012	1	78.886	10.905
ct92-M305Pete-12	120362	03/10/2012	0	78.901	10.857
ct92-M305Pete-12	120362	03/10/2012	-1	78.889	10.838
ct92-M305Pete-12	120362	03/10/2012	0	78.867	10.873
ct92-M305Pete-12	120362	03/10/2012	-1	78.899	10.817
ct92-M305Pete-12	120362	03/10/2012	-2	78.89	10.827
ct92-M305Pete-12	120362	03/10/2012	-2	78.896	10.795
ct92-M305Pete-12	120362	03/10/2012	-2	78.892	10.726
ct92-M305Pete-12	120362	03/10/2012	-2	78.892	10.727
ct92-M305Pete-12	120362	03/10/2012	-2	78.891	10.732
ct92-M305Pete-12	120362	03/10/2012	-2	78.887	10.716
ct92-M305Pete-12	120362	03/10/2012	-2	78.893	10.73
ct92-M305Pete-12	120362	03/10/2012	-2	78.897	10.75
ct92-M305Pete-12	120362	03/10/2012	-2	78.893	10.729
ct92-M305Pete-12	120362	03/10/2012	-2	78.903	10.739
ct92-M305Pete-12	120362	03/10/2012	-2	78.901	10.75
ct92-M305Pete-12	120362	03/10/2012	-2	78.903	10.742
ct92-M305Pete-12	120362	03/10/2012	-2	78.9	10.754
ct92-M305Pete-12	120362	03/10/2012	-2	78.906	10.747
ct92-M305Pete-12	120362	03/10/2012	-2	78.889	10.766
ct92-M305Pete-12	120362	03/10/2012	-2	78.898	10.67
ct92-M305Pete-12	120362	03/10/2012	-2	78.887	10.743
ct92-M305Pete-12	120362	03/10/2012	-2	78.888	10.746
ct92-M305Pete-12	120362	03/10/2012	-2	78.89	10.747
ct92-M305Pete-12	120362	03/10/2012	-2	78.885	10.739
ct92-M305Pete-12	120362	03/10/2012	-1	78.918	10.823
ct92-M305Pete-12	120362	03/10/2012	-1	78.915	10.817
ct92-M305Pete-12	120362	03/10/2012	-2	78.889	10.766
ct92-M305Pete-12	120362	03/10/2012	-2	78.909	10.838
ct92-M305Pete-12	120362	03/10/2012	-2	78.91	10.845
ct92-M305Pete-12	120362	03/10/2012	-1	78.915	10.892
ct92-M305Pete-12	120362	03/10/2012	-1	78.907	10.77
ct92-M305Pete-12	120362	03/10/2012	-2	78.907	10.771
ct92-M305Pete-12	120362	03/10/2012	-2	78.916	10.821
ct92-M305Pete-12	120362	03/10/2012	-2	78.918	10.793
ct92-M305Pete-12	120362	04/10/2012	-1	78.906	10.84
ct92-M305Pete-12	120362	04/10/2012	-2	78.908	10.774
ct92-M305Pete-12	120362	04/10/2012	-2	78.922	10.748
ct92-M305Pete-12	120362	04/10/2012	-2	78.922	10.75
ct92-M305Pete-12	120362	04/10/2012	-2	78.925	10.767
ct92-M305Pete-12	120362	04/10/2012	-2	78.924	10.77
ct92-M305Pete-12	120362	04/10/2012	-2	78.923	10.786
ct92-M305Pete-12	120362	04/10/2012	-2	78.923	10.775
ct92-M305Pete-12	120362	04/10/2012	-1	78.927	10.805
ct92-M305Pete-12	120362	04/10/2012	-2	78.917	10.806
ct92-M305Pete-12	120362	04/10/2012	2	78.916	10.791
ct92-M305Pete-12	120362	04/10/2012	-1	78.915	10.795
ct92-M305Pete-12	120362	04/10/2012	-2	78.91	10.814
ct92-M305Pete-12	120362	04/10/2012	-2	78.911	10.811
ct92-M305Pete-12	120362	04/10/2012	-2	78.923	10.812

ct92-M305Pete-12	120362	04/10/2012	-2	78.909	10.807
ct92-M305Pete-12	120362	04/10/2012	0	78.912	10.73
ct92-M305Pete-12	120362	04/10/2012	-2	78.908	10.818
ct92-M305Pete-12	120362	04/10/2012	1	78.902	10.767
ct92-M305Pete-12	120362	04/10/2012	-2	78.914	10.771
ct92-M305Pete-12	120362	04/10/2012	-2	78.914	10.785
ct92-M305Pete-12	120362	04/10/2012	-2	78.917	10.802
ct92-M305Pete-12	120362	04/10/2012	-2	78.917	10.773
ct92-M305Pete-12	120362	04/10/2012	-2	78.913	10.814
ct92-M305Pete-12	120362	04/10/2012	-2	78.902	10.854
ct92-M305Pete-12	120362	04/10/2012	-1	78.916	10.844
ct92-M305Pete-12	120362	04/10/2012	-2	78.921	10.843
ct92-M305Pete-12	120362	04/10/2012	-1	78.912	10.825
ct92-M305Pete-12	120362	04/10/2012	-2	78.889	10.878
ct92-M305Pete-12	120362	04/10/2012	-2	78.889	10.865
ct92-M305Pete-12	120362	04/10/2012	-2	78.883	10.918
ct92-M305Pete-12	120362	04/10/2012	-2	78.883	10.917
ct92-M305Pete-12	120362	04/10/2012	-2	78.905	10.968
ct92-M305Pete-12	120362	04/10/2012	-2	78.88	10.886
ct92-M305Pete-12	120362	04/10/2012	-2	78.901	10.894
ct92-M305Pete-12	120362	04/10/2012	-1	78.895	10.867
ct92-M305Pete-12	120362	04/10/2012	0	78.874	10.927
ct92-M305Pete-12	120362	04/10/2012	0	78.904	10.879
ct92-M305Pete-12	120362	04/10/2012	-1	78.914	10.893
ct92-M305Pete-12	120362	04/10/2012	1	78.882	10.824
ct92-M305Pete-12	120362	04/10/2012	-1	78.915	10.815
ct92-M305Pete-12	120362	04/10/2012	1	78.917	10.825
ct92-M305Pete-12	120362	04/10/2012	-2	78.914	10.809
ct92-M305Pete-12	120362	04/10/2012	1	78.911	10.827
ct92-M305Pete-12	120362	04/10/2012	-1	78.918	10.806
ct92-M305Pete-12	120362	04/10/2012	-2	78.92	10.807
ct92-M305Pete-12	120362	04/10/2012	-2	78.919	10.806
ct92-M305Pete-12	120362	04/10/2012	-2	78.927	10.835
ct92-M305Pete-12	120362	04/10/2012	-2	78.922	10.844
ct92-M305Pete-12	120362	04/10/2012	-1	78.912	10.826
ct92-M305Pete-12	120362	04/10/2012	-2	78.912	10.82
ct92-M305Pete-12	120362	04/10/2012	-2	78.92	10.802
ct92-M305Pete-12	120362	04/10/2012	-2	78.92	10.818
ct92-M305Pete-12	120362	04/10/2012	0	78.92	10.732
ct92-M305Pete-12	120362	04/10/2012	-2	78.916	10.719
ct92-M305Pete-12	120362	04/10/2012	1	78.916	10.718
ct92-M305Pete-12	120362	04/10/2012	-2	78.917	10.826
ct92-M305Pete-12	120362	04/10/2012	-1	78.927	10.808
ct92-M305Pete-12	120362	04/10/2012	-2	78.921	10.796
ct92-M305Pete-12	120362	04/10/2012	0	78.921	10.808
ct92-M305Pete-12	120362	04/10/2012	-1	78.911	10.77
ct92-M305Pete-12	120362	04/10/2012	-2	78.934	10.837
ct92-M305Pete-12	120362	04/10/2012	-2	78.938	10.842
ct92-M305Pete-12	120362	04/10/2012	-2	78.94	10.829
ct92-M305Pete-12	120362	04/10/2012	-1	78.93	10.847
ct92-M305Pete-12	120362	04/10/2012	-2	78.931	10.838
ct92-M305Pete-12	120362	04/10/2012	-2	78.922	10.814
ct92-M305Pete-12	120362	04/10/2012	-2	78.928	10.799
ct92-M305Pete-12	120362	04/10/2012	-2	78.929	10.802
ct92-M305Pete-12	120362	04/10/2012	-2	78.949	10.672
ct92-M305Pete-12	120362	04/10/2012	-2	78.936	10.653
ct92-M305Pete-12	120362	04/10/2012	-1	78.938	10.807
ct92-M305Pete-12	120362	04/10/2012	-2	78.931	10.765
ct92-M305Pete-12	120362	04/10/2012	-2	78.927	10.768
ct92-M305Pete-12	120362	04/10/2012	-2	78.93	10.768
ct92-M305Pete-12	120362	04/10/2012	-1	78.897	10.837
ct92-M305Pete-12	120362	04/10/2012	-1	78.914	10.776
ct92-M305Pete-12	120362	04/10/2012	-2	78.896	10.838
ct92-M305Pete-12	120362	04/10/2012	-2	78.88	10.871
ct92-M305Pete-12	120362	05/10/2012	-2	78.873	10.904
ct92-M305Pete-12	120362	05/10/2012	-2	78.866	10.899
ct92-M305Pete-12	120362	05/10/2012	-2	78.889	10.873
ct92-M305Pete-12	120362	05/10/2012	-2	78.892	10.748

ct92-M305Pete-12	120362	05/10/2012	-2	78.89	10.748
ct92-M305Pete-12	120362	05/10/2012	-2	78.928	10.728
ct92-M305Pete-12	120362	05/10/2012	-2	78.929	10.716
ct92-M305Pete-12	120362	05/10/2012	-2	78.931	10.716
ct92-M305Pete-12	120362	05/10/2012	-2	78.93	10.715
ct92-M305Pete-12	120362	05/10/2012	-2	78.937	10.767
ct92-M305Pete-12	120362	05/10/2012	-2	78.936	10.766
ct92-M305Pete-12	120362	05/10/2012	-2	78.932	10.762
ct92-M305Pete-12	120362	05/10/2012	-2	78.931	10.765
ct92-M305Pete-12	120362	05/10/2012	-2	78.916	10.76
ct92-M305Pete-12	120362	05/10/2012	-1	78.917	10.754
ct92-M305Pete-12	120362	05/10/2012	-2	78.921	10.83
ct92-M305Pete-12	120362	05/10/2012	-2	78.924	10.822
ct92-M305Pete-12	120362	05/10/2012	-2	78.901	10.796
ct92-M305Pete-12	120362	05/10/2012	-2	78.903	10.765
ct92-M305Pete-12	120362	05/10/2012	-2	78.906	10.762
ct92-M305Pete-12	120362	05/10/2012	-2	78.918	10.753
ct92-M305Pete-12	120362	05/10/2012	-1	78.907	10.741
ct92-M305Pete-12	120362	05/10/2012	-2	78.917	10.75
ct92-M305Pete-12	120362	05/10/2012	-2	78.919	10.723
ct92-M305Pete-12	120362	05/10/2012	-2	78.915	10.716
ct92-M305Pete-12	120362	05/10/2012	-2	78.925	10.724
ct92-M305Pete-12	120362	05/10/2012	-2	78.923	10.717
ct92-M305Pete-12	120362	05/10/2012	-2	78.924	10.715
ct92-M305Pete-12	120362	05/10/2012	-2	78.92	10.737
ct92-M305Pete-12	120362	05/10/2012	-2	78.927	10.757
ct92-M305Pete-12	120362	05/10/2012	-2	78.918	10.73
ct92-M305Pete-12	120362	05/10/2012	-2	78.917	10.726
ct92-M305Pete-12	120362	05/10/2012	1	78.91	10.756
ct92-M305Pete-12	120362	05/10/2012	-2	78.91	10.751
ct92-M305Pete-12	120362	05/10/2012	-2	78.914	10.732
ct92-M305Pete-12	120362	05/10/2012	-1	78.912	10.649
ct92-M305Pete-12	120362	05/10/2012	1	78.909	10.627
ct92-M305Pete-12	120362	05/10/2012	-1	78.911	10.664
ct92-M305Pete-12	120362	05/10/2012	-2	78.922	10.719
ct92-M305Pete-12	120362	05/10/2012	-1	78.911	10.752
ct92-M305Pete-12	120362	05/10/2012	-2	78.917	10.734
ct92-M305Pete-12	120362	05/10/2012	-1	78.914	10.703
ct92-M305Pete-12	120362	05/10/2012	-2	78.915	10.698
ct92-M305Pete-12	120362	05/10/2012	-2	78.915	10.689
ct92-M305Pete-12	120362	05/10/2012	-2	78.909	10.677
ct92-M305Pete-12	120362	05/10/2012	-2	78.914	10.68
ct92-M305Pete-12	120362	05/10/2012	-2	78.911	10.705
ct92-M305Pete-12	120362	05/10/2012	-2	78.912	10.709
ct92-M305Pete-12	120362	05/10/2012	-2	78.906	10.711
ct92-M305Pete-12	120362	05/10/2012	-2	78.905	10.71
ct92-M305Pete-12	120362	05/10/2012	1	78.931	10.821
ct92-M305Pete-12	120362	05/10/2012	-2	78.905	10.728
ct92-M305Pete-12	120362	05/10/2012	-2	78.907	10.742
ct92-M305Pete-12	120362	05/10/2012	-1	78.928	10.77
ct92-M305Pete-12	120362	05/10/2012	-2	78.929	10.772
ct92-M305Pete-12	120362	05/10/2012	-1	78.919	10.714
ct92-M305Pete-12	120362	05/10/2012	-2	78.913	10.585
ct92-M305Pete-12	120362	05/10/2012	-1	78.914	10.683
ct92-M305Pete-12	120362	05/10/2012	-2	78.914	10.734
ct92-M305Pete-12	120362	05/10/2012	-2	78.917	10.732
ct92-M305Pete-12	120362	05/10/2012	-2	78.909	10.645
ct92-M305Pete-12	120362	05/10/2012	-2	78.89	10.375
ct92-M305Pete-12	120362	05/10/2012	-1	78.932	10.635
ct92-M305Pete-12	120362	05/10/2012	-1	78.927	10.622
ct92-M305Pete-12	120362	05/10/2012	-2	78.892	10.366
ct92-M305Pete-12	120362	05/10/2012	-2	78.892	10.37
ct92-M305Pete-12	120362	05/10/2012	0	78.922	10.605
ct92-M305Pete-12	120362	06/10/2012	-2	78.935	10.567
ct92-M305Pete-12	120362	06/10/2012	-2	78.929	10.591
ct92-M305Pete-12	120362	06/10/2012	-1	78.914	10.648
ct92-M305Pete-12	120362	06/10/2012	-2	78.932	10.613
ct92-M305Pete-12	120362	06/10/2012	-1	78.92	10.57

ct92-M305Pete-12	120362	06/10/2012	-2	78.927	10.562
ct92-M305Pete-12	120362	06/10/2012	1	78.936	10.658
ct92-M305Pete-12	120362	06/10/2012	1	78.935	10.642
ct92-M305Pete-12	120362	06/10/2012	-2	78.936	10.658
ct92-M305Pete-12	120362	06/10/2012	-2	78.936	10.656
ct92-M305Pete-12	120362	06/10/2012	0	78.935	10.704
ct92-M305Pete-12	120362	06/10/2012	-1	78.923	10.74
ct92-M305Pete-12	120362	06/10/2012	-2	78.905	10.706
ct92-M305Pete-12	120362	06/10/2012	-1	78.901	10.74
ct92-M305Pete-12	120362	06/10/2012	-2	78.899	10.725
ct92-M305Pete-12	120362	06/10/2012	-2	78.899	10.733
ct92-M305Pete-12	120362	06/10/2012	-1	78.939	10.744
ct92-M305Pete-12	120362	06/10/2012	-2	78.898	10.739
ct92-M305Pete-12	120362	06/10/2012	-2	78.898	10.741
ct92-M305Pete-12	120362	06/10/2012	-2	78.897	10.74
ct92-M305Pete-12	120362	06/10/2012	-1	78.875	10.662
ct92-M305Pete-12	120362	06/10/2012	-2	78.878	10.67
ct92-M305Pete-12	120362	06/10/2012	-2	78.848	10.612
ct92-M305Pete-12	120362	06/10/2012	-2	78.862	10.664
ct92-M305Pete-12	120362	06/10/2012	-2	78.857	10.662
ct92-M305Pete-12	120362	06/10/2012	-2	78.849	10.65
ct92-M305Pete-12	120362	06/10/2012	-2	78.879	10.676
ct92-M305Pete-12	120362	06/10/2012	-2	78.886	10.671
ct92-M305Pete-12	120362	06/10/2012	-2	78.888	10.674
ct92-M305Pete-12	120362	06/10/2012	-2	78.895	10.674
ct92-M305Pete-12	120362	06/10/2012	-2	78.888	10.675
ct92-M305Pete-12	120362	06/10/2012	-2	78.896	10.673
ct92-M305Pete-12	120362	06/10/2012	-2	78.888	10.614
ct92-M305Pete-12	120362	07/10/2012	-2	78.886	10.646
ct92-M305Pete-12	120362	07/10/2012	-2	78.885	10.687
ct92-M305Pete-12	120362	07/10/2012	-2	78.887	10.688
ct92-M305Pete-12	120362	07/10/2012	-1	78.895	10.678
ct92-M305Pete-12	120362	07/10/2012	-2	78.897	10.676
ct92-M305Pete-12	120362	07/10/2012	-2	78.899	10.676
ct92-M305Pete-12	120362	07/10/2012	-2	78.899	10.676
ct92-M305Pete-12	120362	07/10/2012	-2	78.905	10.693
ct92-M305Pete-12	120362	07/10/2012	-2	78.904	10.703
ct92-M305Pete-12	120362	07/10/2012	-2	78.917	10.662
ct92-M305Pete-12	120362	07/10/2012	1	78.934	10.778
ct92-M305Pete-12	120362	07/10/2012	-2	78.933	10.778
ct92-M305Pete-12	120362	07/10/2012	-2	78.932	10.775
ct92-M305Pete-12	120362	07/10/2012	-2	78.934	10.784
ct92-M305Pete-12	120362	07/10/2012	-2	78.943	10.81
ct92-M305Pete-12	120362	07/10/2012	-2	78.939	10.814
ct92-M305Pete-12	120362	07/10/2012	-2	78.933	10.826
ct92-M305Pete-12	120362	07/10/2012	-2	78.926	10.797
ct92-M305Pete-12	120362	07/10/2012	-2	78.928	10.797
ct92-M305Pete-12	120362	07/10/2012	-2	78.964	10.862
ct92-M305Pete-12	120362	07/10/2012	-1	78.909	10.812
ct92-M305Pete-12	120362	07/10/2012	-2	78.929	10.795
ct92-M305Pete-12	120362	07/10/2012	-2	78.914	10.849
ct92-M305Pete-12	120362	07/10/2012	-1	78.938	10.808
ct92-M305Pete-12	120362	07/10/2012	-2	78.927	10.809
ct92-M305Pete-12	120362	07/10/2012	-2	78.922	10.822
ct92-M305Pete-12	120362	07/10/2012	-2	78.92	10.823
ct92-M305Pete-12	120362	07/10/2012	-2	78.902	10.834
ct92-M305Pete-12	120362	07/10/2012	0	78.88	10.676
ct92-M305Pete-12	120362	07/10/2012	0	78.898	10.919
ct92-M305Pete-12	120362	07/10/2012	-2	78.9	10.906
ct92-M305Pete-12	120362	07/10/2012	-2	78.872	10.645
ct92-M305Pete-12	120362	07/10/2012	-2	78.901	10.695
ct92-M305Pete-12	120362	07/10/2012	-2	78.901	10.695
ct92-M305Pete-12	120362	07/10/2012	-2	78.878	10.659
ct92-M305Pete-12	120362	07/10/2012	-2	78.871	10.708
ct92-M305Pete-12	120362	07/10/2012	-2	78.871	10.698
ct92-M305Pete-12	120362	07/10/2012	-2	78.886	10.757
ct92-M305Pete-12	120362	07/10/2012	-2	78.913	10.623
ct92-M305Pete-12	120362	07/10/2012	-2	78.89	10.76

ct92-M305Pete-12	120362	07/10/2012	-2	78.897	10.755
ct92-M305Pete-12	120362	07/10/2012	-2	78.895	10.764
ct92-M305Pete-12	120362	07/10/2012	-2	78.905	10.748
ct92-M305Pete-12	120362	07/10/2012	-2	78.921	10.682
ct92-M305Pete-12	120362	07/10/2012	-2	78.904	10.736
ct92-M305Pete-12	120362	07/10/2012	-1	78.92	10.694
ct92-M305Pete-12	120362	07/10/2012	-2	78.927	10.711
ct92-M305Pete-12	120362	07/10/2012	-2	78.924	10.791
ct92-M305Pete-12	120362	07/10/2012	-2	78.933	10.791
ct92-M305Pete-12	120362	07/10/2012	-2	78.929	10.79
ct92-M305Pete-12	120362	07/10/2012	-2	78.936	10.785
ct92-M305Pete-12	120362	07/10/2012	-2	78.932	10.789
ct92-M305Pete-12	120362	07/10/2012	-2	78.931	10.774
ct92-M305Pete-12	120362	07/10/2012	-2	78.933	10.691
ct92-M305Pete-12	120362	07/10/2012	-2	78.931	10.693
ct92-M305Pete-12	120362	07/10/2012	-1	78.931	10.717
ct92-M305Pete-12	120362	07/10/2012	-2	78.914	10.648
ct92-M305Pete-12	120362	07/10/2012	-2	78.919	10.594
ct92-M305Pete-12	120362	07/10/2012	-2	78.919	10.576
ct92-M305Pete-12	120362	07/10/2012	-2	78.922	10.589
ct92-M305Pete-12	120362	07/10/2012	-2	78.924	10.553
ct92-M305Pete-12	120362	07/10/2012	-2	78.904	10.721
ct92-M305Pete-12	120362	07/10/2012	-2	78.925	10.647
ct92-M305Pete-12	120362	07/10/2012	-2	78.925	10.741
ct92-M305Pete-12	120362	07/10/2012	-2	78.926	10.751
ct92-M305Pete-12	120362	07/10/2012	-2	78.923	10.746
ct92-M305Pete-12	120362	08/10/2012	-2	78.925	10.712
ct92-M305Pete-12	120362	08/10/2012	-2	78.932	10.678
ct92-M305Pete-12	120362	08/10/2012	-2	78.928	10.769
ct92-M305Pete-12	120362	08/10/2012	-2	78.924	10.835
ct92-M305Pete-12	120362	08/10/2012	1	78.922	10.751
ct92-M305Pete-12	120362	08/10/2012	-1	78.923	10.817
ct92-M305Pete-12	120362	08/10/2012	-2	78.917	10.832
ct92-M305Pete-12	120362	08/10/2012	-2	78.918	10.822
ct92-M305Pete-12	120362	08/10/2012	-2	78.918	10.839
ct92-M305Pete-12	120362	08/10/2012	-2	78.916	10.858
ct92-M305Pete-12	120362	08/10/2012	-2	78.913	10.883
ct92-M305Pete-12	120362	08/10/2012	-2	78.914	10.832
ct92-M305Pete-12	120362	08/10/2012	-2	78.909	10.856
ct92-M305Pete-12	120362	08/10/2012	-2	78.91	10.835
ct92-M305Pete-12	120362	08/10/2012	-2	78.905	10.857
ct92-M305Pete-12	120362	08/10/2012	-2	78.908	10.834
ct92-M305Pete-12	120362	08/10/2012	-2	78.904	10.864
ct92-M305Pete-12	120362	08/10/2012	-2	78.919	10.756
ct92-M305Pete-12	120362	08/10/2012	1	78.922	10.849
ct92-M305Pete-12	120362	08/10/2012	-1	78.927	10.773
ct92-M305Pete-12	120362	08/10/2012	-2	78.93	10.817
ct92-M305Pete-12	120362	08/10/2012	1	78.932	10.809
ct92-M305Pete-12	120362	08/10/2012	-2	78.915	10.823
ct92-M305Pete-12	120362	08/10/2012	-2	78.912	10.868
ct92-M305Pete-12	120362	08/10/2012	-2	78.907	10.857
ct92-M305Pete-12	120362	08/10/2012	-2	78.911	10.889
ct92-M305Pete-12	120362	08/10/2012	-1	78.883	10.904
ct92-M305Pete-12	120362	08/10/2012	2	78.904	10.875
ct92-M305Pete-12	120362	08/10/2012	-1	78.895	10.906
ct92-M305Pete-12	120362	08/10/2012	-2	78.895	10.903
ct92-M305Pete-12	120362	08/10/2012	-2	78.895	10.891
ct92-M305Pete-12	120362	08/10/2012	-2	78.881	10.922
ct92-M305Pete-12	120362	08/10/2012	-2	78.882	10.922
ct92-M305Pete-12	120362	08/10/2012	0	78.89	10.942
ct92-M305Pete-12	120362	08/10/2012	-2	78.882	10.895
ct92-M305Pete-12	120362	08/10/2012	-1	78.847	10.738
ct92-M305Pete-12	120362	08/10/2012	-2	78.883	10.896
ct92-M305Pete-12	120362	08/10/2012	-1	78.888	10.916
ct92-M305Pete-12	120362	08/10/2012	-2	78.907	10.879
ct92-M305Pete-12	120362	08/10/2012	-1	78.906	10.873
ct92-M305Pete-12	120362	08/10/2012	-2	78.896	10.852
ct92-M305Pete-12	120362	08/10/2012	-1	78.903	10.815

ct92-M305Pete-12	120362	08/10/2012	-2	78.88	10.861
ct92-M305Pete-12	120362	08/10/2012	-2	78.9	10.851
ct92-M305Pete-12	120362	08/10/2012	-1	78.891	10.807
ct92-M305Pete-12	120362	08/10/2012	-1	78.896	10.888
ct92-M305Pete-12	120362	08/10/2012	-2	78.888	10.831
ct92-M305Pete-12	120362	08/10/2012	1	78.885	10.844
ct92-M305Pete-12	120362	08/10/2012	-2	78.889	10.83
ct92-M305Pete-12	120362	08/10/2012	-2	78.891	10.844
ct92-M305Pete-12	120362	08/10/2012	-1	78.888	10.809
ct92-M305Pete-12	120362	08/10/2012	-1	78.922	10.852
ct92-M305Pete-12	120362	08/10/2012	0	78.916	10.809
ct92-M305Pete-12	120362	08/10/2012	-1	78.919	10.756
ct92-M305Pete-12	120362	08/10/2012	2	78.919	10.775
ct92-M305Pete-12	120362	08/10/2012	1	78.921	10.781
ct92-M305Pete-12	120362	08/10/2012	-2	78.933	10.815
ct92-M305Pete-12	120362	08/10/2012	2	78.918	10.718
ct92-M305Pete-12	120362	08/10/2012	-2	78.938	10.805
ct92-M305Pete-12	120362	08/10/2012	-1	78.924	10.727
ct92-M305Pete-12	120362	08/10/2012	-2	78.931	10.753
ct92-M305Pete-12	120362	08/10/2012	1	78.929	10.727
ct92-M305Pete-12	120362	08/10/2012	-2	78.928	10.73
ct92-M305Pete-12	120362	08/10/2012	2	78.918	10.684
ct92-M305Pete-12	120362	08/10/2012	-2	78.917	10.689
ct92-M305Pete-12	120362	08/10/2012	-1	78.911	10.678
ct92-M305Pete-12	120362	08/10/2012	-2	78.908	10.678
ct92-M305Pete-12	120362	08/10/2012	-2	78.907	10.682
ct92-M305Pete-12	120362	08/10/2012	-2	78.907	10.679
ct92-M305Pete-12	120362	08/10/2012	-2	78.907	10.672
ct92-M305Pete-12	120362	08/10/2012	-2	78.905	10.63
ct92-M305Pete-12	120362	08/10/2012	-2	78.916	10.603
ct92-M305Pete-12	120362	09/10/2012	-2	78.912	10.555
ct92-M305Pete-12	120362	09/10/2012	-2	78.914	10.552
ct92-M305Pete-12	120362	09/10/2012	-2	78.903	10.623
ct92-M305Pete-12	120362	09/10/2012	-2	78.908	10.681
ct92-M305Pete-12	120362	09/10/2012	-1	78.902	10.662
ct92-M305Pete-12	120362	09/10/2012	-2	78.905	10.651
ct92-M305Pete-12	120362	09/10/2012	-2	78.902	10.644
ct92-M305Pete-12	120362	09/10/2012	-2	78.906	10.606
ct92-M305Pete-12	120362	09/10/2012	-2	78.908	10.626
ct92-M305Pete-12	120362	09/10/2012	-1	78.907	10.66
ct92-M305Pete-12	120362	09/10/2012	-2	78.906	10.638
ct92-M305Pete-12	120362	09/10/2012	-2	78.906	10.641
ct92-M305Pete-12	120362	09/10/2012	-2	78.906	10.637
ct92-M305Pete-12	120362	09/10/2012	-2	78.907	10.624
ct92-M305Pete-12	120362	09/10/2012	-1	78.913	10.569
ct92-M305Pete-12	120362	09/10/2012	-2	78.915	10.549
ct92-M305Pete-12	120362	09/10/2012	-2	78.919	10.598
ct92-M305Pete-12	120362	09/10/2012	-1	78.916	10.605
ct92-M305Pete-12	120362	09/10/2012	-2	78.915	10.602
ct92-M305Pete-12	120362	09/10/2012	-1	78.919	10.589
ct92-M305Pete-12	120362	09/10/2012	-2	78.924	10.565
ct92-M305Pete-12	120362	09/10/2012	-1	78.921	10.6
ct92-M305Pete-12	120362	09/10/2012	-2	78.924	10.578
ct92-M305Pete-12	120362	09/10/2012	-2	78.921	10.626
ct92-M305Pete-12	120362	09/10/2012	-2	78.923	10.622
ct92-M305Pete-12	120362	09/10/2012	-2	78.924	10.626
ct92-M305Pete-12	120362	09/10/2012	-2	78.916	10.617
ct92-M305Pete-12	120362	09/10/2012	-2	78.921	10.616
ct92-M305Pete-12	120362	09/10/2012	-2	78.929	10.54
ct92-M305Pete-12	120362	09/10/2012	-1	78.908	10.585
ct92-M305Pete-12	120362	09/10/2012	-2	78.908	10.584
ct92-M305Pete-12	120362	09/10/2012	-2	78.922	10.57
ct92-M305Pete-12	120362	09/10/2012	0	78.902	10.507
ct92-M305Pete-12	120362	09/10/2012	-2	78.903	10.507
ct92-M305Pete-12	120362	09/10/2012	-2	78.917	10.553
ct92-M305Pete-12	120362	09/10/2012	-1	78.909	10.583
ct92-M305Pete-12	120362	09/10/2012	-2	78.91	10.57
ct92-M305Pete-12	120362	09/10/2012	0	78.92	10.568

ct92-M305Pete-12	120362	09/10/2012	0	78.962	10.433
ct92-M305Pete-12	120362	09/10/2012	-2	78.897	10.575
ct92-M305Pete-12	120362	09/10/2012	-2	78.899	10.61
ct92-M305Pete-12	120362	09/10/2012	-2	78.899	10.611
ct92-M305Pete-12	120362	09/10/2012	-2	78.905	10.544
ct92-M305Pete-12	120362	09/10/2012	-2	78.918	10.503
ct92-M305Pete-12	120362	09/10/2012	-2	78.905	10.552
ct92-M305Pete-12	120362	09/10/2012	-2	78.903	10.57
ct92-M305Pete-12	120362	09/10/2012	-2	78.904	10.572
ct92-M305Pete-12	120362	09/10/2012	-2	78.906	10.611
ct92-M305Pete-12	120362	09/10/2012	-2	78.913	10.623
ct92-M305Pete-12	120362	09/10/2012	-1	78.919	10.676
ct92-M305Pete-12	120362	09/10/2012	-2	78.913	10.636
ct92-M305Pete-12	120362	09/10/2012	-2	78.91	10.631
ct92-M305Pete-12	120362	09/10/2012	-2	78.91	10.648
ct92-M305Pete-12	120362	09/10/2012	-1	78.938	10.702
ct92-M305Pete-12	120362	09/10/2012	-2	78.917	10.696
ct92-M305Pete-12	120362	09/10/2012	-2	78.936	10.697
ct92-M305Pete-12	120362	09/10/2012	-2	78.917	10.676
ct92-M305Pete-12	120362	09/10/2012	-2	78.918	10.692
ct92-M305Pete-12	120362	09/10/2012	-2	78.926	10.7
ct92-M305Pete-12	120362	09/10/2012	-1	78.936	10.733
ct92-M305Pete-12	120362	09/10/2012	-1	78.929	10.734
ct92-M305Pete-12	120362	09/10/2012	-2	78.929	10.73
ct92-M305Pete-12	120362	09/10/2012	-2	78.93	10.743
ct92-M305Pete-12	120362	09/10/2012	-1	78.904	10.714
ct92-M305Pete-12	120362	09/10/2012	-1	78.905	10.712
ct92-M305Pete-12	120362	09/10/2012	-1	78.905	10.648
ct92-M305Pete-12	120362	09/10/2012	-2	78.905	10.647
ct92-M305Pete-12	120362	09/10/2012	2	78.898	10.665
ct92-M305Pete-12	120362	09/10/2012	-2	78.897	10.651
ct92-M305Pete-12	120362	09/10/2012	-2	78.9	10.657
ct92-M305Pete-12	120362	09/10/2012	-2	78.885	10.644
ct92-M305Pete-12	120362	09/10/2012	1	78.887	10.692
ct92-M305Pete-12	120362	09/10/2012	2	78.886	10.719
ct92-M305Pete-12	120362	09/10/2012	-2	78.882	10.716
ct92-M305Pete-12	120362	09/10/2012	-2	78.889	10.699
ct92-M305Pete-12	120362	10/10/2012	-2	78.879	10.657
ct92-M305Pete-12	120362	10/10/2012	-1	78.892	10.645
ct92-M305Pete-12	120362	10/10/2012	-1	78.892	10.626
ct92-M305Pete-12	120362	10/10/2012	-2	78.892	10.646
ct92-M305Pete-12	120362	10/10/2012	-2	78.893	10.642
ct92-M305Pete-12	120362	10/10/2012	-2	78.9	10.623
ct92-M305Pete-12	120362	10/10/2012	-2	78.893	10.613
ct92-M305Pete-12	120362	10/10/2012	2	78.893	10.609
ct92-M305Pete-12	120362	10/10/2012	-1	78.892	10.631
ct92-M305Pete-12	120362	10/10/2012	-2	78.893	10.605
ct92-M305Pete-12	120362	10/10/2012	-2	78.894	10.616
ct92-M305Pete-12	120362	10/10/2012	-2	78.894	10.622
ct92-M305Pete-12	120362	10/10/2012	-2	78.895	10.611
ct92-M305Pete-12	120362	10/10/2012	-2	78.893	10.614
ct92-M305Pete-12	120362	10/10/2012	-2	78.888	10.631
ct92-M305Pete-12	120362	10/10/2012	-2	78.888	10.626
ct92-M305Pete-12	120362	10/10/2012	-2	78.891	10.63
ct92-M305Pete-12	120362	10/10/2012	-2	78.879	10.603
ct92-M305Pete-12	120362	10/10/2012	-2	78.875	10.622
ct92-M305Pete-12	120362	10/10/2012	-2	78.878	10.64
ct92-M305Pete-12	120362	10/10/2012	-2	78.885	10.553
ct92-M305Pete-12	120362	10/10/2012	-2	78.883	10.554
ct92-M305Pete-12	120362	10/10/2012	-2	78.882	10.555
ct92-M305Pete-12	120362	10/10/2012	-1	78.91	10.579
ct92-M305Pete-12	120362	10/10/2012	-1	78.913	10.583
ct92-M305Pete-12	120362	10/10/2012	-2	78.919	10.578
ct92-M305Pete-12	120362	10/10/2012	-2	78.919	10.58
ct92-M305Pete-12	120362	10/10/2012	-2	78.913	10.633
ct92-M305Pete-12	120362	10/10/2012	-2	78.912	10.629
ct92-M305Pete-12	120362	10/10/2012	-2	78.909	10.634
ct92-M305Pete-12	120362	10/10/2012	-2	78.913	10.635

ct92-M305Pete-12	120362	10/10/2012	-2	78.915	10.618
ct92-M305Pete-12	120362	10/10/2012	1	78.915	10.592
ct92-M305Pete-12	120362	10/10/2012	-2	78.897	10.597
ct92-M305Pete-12	120362	10/10/2012	-2	78.896	10.592
ct92-M305Pete-12	120362	10/10/2012	-1	78.898	10.558
ct92-M305Pete-12	120362	10/10/2012	0	78.904	10.614
ct92-M305Pete-12	120362	10/10/2012	2	78.905	10.606
ct92-M305Pete-12	120362	10/10/2012	-1	78.907	10.599
ct92-M305Pete-12	120362	10/10/2012	-2	78.908	10.631
ct92-M305Pete-12	120362	10/10/2012	-2	78.904	10.603
ct92-M305Pete-12	120362	10/10/2012	-2	78.904	10.609
ct92-M305Pete-12	120362	10/10/2012	-2	78.909	10.633
ct92-M305Pete-12	120362	10/10/2012	-2	78.906	10.614
ct92-M305Pete-12	120362	10/10/2012	-2	78.911	10.636
ct92-M305Pete-12	120362	10/10/2012	-1	78.919	10.614
ct92-M305Pete-12	120362	10/10/2012	-2	78.912	10.634
ct92-M305Pete-12	120362	10/10/2012	-1	78.925	10.661
ct92-M305Pete-12	120362	10/10/2012	-2	78.917	10.659
ct92-M305Pete-12	120362	10/10/2012	-2	78.918	10.682
ct92-M305Pete-12	120362	10/10/2012	-2	78.928	10.668
ct92-M305Pete-12	120362	10/10/2012	-2	78.916	10.692
ct92-M305Pete-12	120362	10/10/2012	-2	78.917	10.712
ct92-M305Pete-12	120362	10/10/2012	-2	78.918	10.717
ct92-M305Pete-12	120362	10/10/2012	-2	78.918	10.711
ct92-M305Pete-12	120362	10/10/2012	-1	78.924	10.697
ct92-M305Pete-12	120362	10/10/2012	-1	78.925	10.699
ct92-M305Pete-12	120362	10/10/2012	-2	78.925	10.699
ct92-M305Pete-12	120362	10/10/2012	-2	78.935	10.738
ct92-M305Pete-12	120362	10/10/2012	-2	78.932	10.799
ct92-M305Pete-12	120362	10/10/2012	-2	78.924	10.691
ct92-M305Pete-12	120362	10/10/2012	-2	78.932	10.796
ct92-M305Pete-12	120362	10/10/2012	-2	78.932	10.811
ct92-M305Pete-12	120362	10/10/2012	-2	78.934	10.891
ct92-M305Pete-12	120362	11/10/2012	-2	78.931	11.011
ct92-M305Pete-12	120362	11/10/2012	-2	78.931	11.006
ct92-M305Pete-12	120362	11/10/2012	-2	78.923	10.95
ct92-M305Pete-12	120362	11/10/2012	-2	78.932	10.973
ct92-M305Pete-12	120362	11/10/2012	-2	78.907	10.911
ct92-M305Pete-12	120362	11/10/2012	-2	78.902	10.907
ct92-M305Pete-12	120362	11/10/2012	-2	78.903	10.844
ct92-M305Pete-12	120362	11/10/2012	-2	78.911	10.778
ct92-M305Pete-12	120362	11/10/2012	-2	78.912	10.776
ct92-M305Pete-12	120362	11/10/2012	-2	78.908	10.785
ct92-M305Pete-12	120362	11/10/2012	-2	78.907	10.787
ct92-M305Pete-12	120362	11/10/2012	-2	78.909	10.785
ct92-M305Pete-12	120362	11/10/2012	-2	78.903	10.806
ct92-M305Pete-12	120362	11/10/2012	-2	78.904	10.818
ct92-M305Pete-12	120362	11/10/2012	-2	78.904	10.812
ct92-M305Pete-12	120362	11/10/2012	-1	78.905	10.835
ct92-M305Pete-12	120362	11/10/2012	-2	78.9	10.846
ct92-M305Pete-12	120362	11/10/2012	-2	78.903	10.833
ct92-M305Pete-12	120362	11/10/2012	0	78.923	10.818
ct92-M305Pete-12	120362	11/10/2012	-2	78.922	10.818
ct92-M305Pete-12	120362	11/10/2012	-2	78.923	10.819
ct92-M305Pete-12	120362	11/10/2012	-2	78.898	10.835
ct92-M305Pete-12	120362	11/10/2012	-2	78.896	10.838
ct92-M305Pete-12	120362	11/10/2012	-1	78.911	10.876
ct92-M305Pete-12	120362	11/10/2012	-2	78.905	10.864
ct92-M305Pete-12	120362	11/10/2012	-2	78.911	10.876
ct92-M305Pete-12	120362	11/10/2012	-2	78.89	10.853
ct92-M305Pete-12	120362	11/10/2012	-2	78.902	10.876
ct92-M305Pete-12	120362	11/10/2012	-2	78.9	10.876
ct92-M305Pete-12	120362	11/10/2012	-1	78.895	10.847
ct92-M305Pete-12	120362	11/10/2012	-1	78.893	10.862
ct92-M305Pete-12	120362	11/10/2012	-1	78.887	10.889
ct92-M305Pete-12	120362	11/10/2012	-2	78.887	10.843
ct92-M305Pete-12	120362	11/10/2012	-1	78.868	10.911
ct92-M305Pete-12	120362	11/10/2012	-1	78.889	10.844

ct92-M305Pete-12	120362	11/10/2012	-2	78.884	10.843
ct92-M305Pete-12	120362	11/10/2012	-2	78.881	10.837
ct92-M305Pete-12	120362	11/10/2012	-2	78.884	10.825
ct92-M305Pete-12	120362	11/10/2012	1	78.881	10.791
ct92-M305Pete-12	120362	11/10/2012	-1	78.88	10.819
ct92-M305Pete-12	120362	11/10/2012	-1	78.878	10.824
ct92-M305Pete-12	120362	11/10/2012	2	78.884	10.811
ct92-M305Pete-12	120362	11/10/2012	2	78.888	10.827
ct92-M305Pete-12	120362	11/10/2012	-2	78.892	10.828
ct92-M305Pete-12	120362	11/10/2012	-2	78.878	10.852
ct92-M305Pete-12	120362	11/10/2012	-1	78.903	10.773
ct92-M305Pete-12	120362	11/10/2012	-1	78.904	10.773
ct92-M305Pete-12	120362	11/10/2012	-2	78.893	10.814
ct92-M305Pete-12	120362	11/10/2012	-1	78.902	10.855
ct92-M305Pete-12	120362	11/10/2012	0	78.919	10.808
ct92-M305Pete-12	120362	11/10/2012	-1	78.929	10.782
ct92-M305Pete-12	120362	11/10/2012	-2	78.929	10.782
ct92-M305Pete-12	120362	11/10/2012	-2	78.912	10.729
ct92-M305Pete-12	120362	11/10/2012	-2	78.912	10.733
ct92-M305Pete-12	120362	11/10/2012	1	78.938	10.848
ct92-M305Pete-12	120362	11/10/2012	-2	78.934	10.817
ct92-M305Pete-12	120362	11/10/2012	-2	78.934	10.826
ct92-M305Pete-12	120362	11/10/2012	-2	78.933	10.831
ct92-M305Pete-12	120362	11/10/2012	-2	78.938	10.79
ct92-M305Pete-12	120362	11/10/2012	-2	78.93	10.684
ct92-M305Pete-12	120362	11/10/2012	-2	78.935	10.713
ct92-M305Pete-12	120362	11/10/2012	-2	78.944	10.697
ct92-M305Pete-12	120362	11/10/2012	-2	78.96	10.774
ct92-M305Pete-12	120362	11/10/2012	-2	78.941	10.703
ct92-M305Pete-12	120362	11/10/2012	0	78.904	10.619
ct92-M305Pete-12	120362	11/10/2012	-1	78.905	10.594
ct92-M305Pete-12	120362	11/10/2012	-1	78.893	10.571
ct92-M305Pete-12	120362	11/10/2012	1	78.882	10.558
ct92-M305Pete-12	120362	11/10/2012	-2	78.9	10.54
ct92-M305Pete-12	120362	12/10/2012	-2	78.914	10.611
ct92-M305Pete-12	120362	12/10/2012	-2	78.919	10.686
ct92-M305Pete-12	120362	12/10/2012	-2	78.921	10.681
ct92-M305Pete-12	120362	12/10/2012	-2	78.922	10.686
ct92-M305Pete-12	120362	12/10/2012	-2	78.931	10.7
ct92-M305Pete-12	120362	12/10/2012	-2	78.926	10.715
ct92-M305Pete-12	120362	12/10/2012	-2	78.93	10.737
ct92-M305Pete-12	120362	12/10/2012	-2	78.93	10.737
ct92-M305Pete-12	120362	12/10/2012	-2	78.93	10.744
ct92-M305Pete-12	120362	12/10/2012	-2	78.931	10.747
ct92-M305Pete-12	120362	12/10/2012	1	78.92	10.796
ct92-M305Pete-12	120362	12/10/2012	-1	78.916	10.813
ct92-M305Pete-12	120362	12/10/2012	0	78.899	10.843
ct92-M305Pete-12	120362	12/10/2012	-2	78.892	10.85
ct92-M305Pete-12	120362	12/10/2012	-1	78.916	10.854
ct92-M305Pete-12	120362	12/10/2012	-2	78.916	10.851
ct92-M305Pete-12	120362	12/10/2012	-1	78.908	10.737
ct92-M305Pete-12	120362	12/10/2012	-2	78.914	10.794
ct92-M305Pete-12	120362	12/10/2012	-2	78.923	10.776
ct92-M305Pete-12	120362	12/10/2012	-2	78.888	10.905
ct92-M305Pete-12	120362	12/10/2012	-2	78.92	10.77
ct92-M305Pete-12	120362	12/10/2012	-2	78.923	10.753
ct92-M305Pete-12	120362	12/10/2012	-2	78.924	10.748
ct92-M305Pete-12	120362	12/10/2012	-2	78.929	10.761
ct92-M305Pete-12	120362	12/10/2012	0	78.923	10.739
ct92-M305Pete-12	120362	12/10/2012	-2	78.921	10.731
ct92-M305Pete-12	120362	12/10/2012	0	78.914	10.78
ct92-M305Pete-12	120362	12/10/2012	1	78.924	10.723
ct92-M305Pete-12	120362	12/10/2012	-2	78.919	10.74
ct92-M305Pete-12	120362	12/10/2012	-2	78.921	10.764
ct92-M305Pete-12	120362	12/10/2012	-1	78.919	10.761
ct92-M305Pete-12	120362	12/10/2012	1	78.914	10.737
ct92-M305Pete-12	120362	12/10/2012	-2	78.91	10.742
ct92-M305Pete-12	120362	12/10/2012	-2	78.91	10.743

ct92-M305Pete-12	120362	12/10/2012	-2	78.917	10.762
ct92-M305Pete-12	120362	12/10/2012	-2	78.917	10.77
ct92-M305Pete-12	120362	12/10/2012	-1	78.927	10.802
ct92-M305Pete-12	120362	12/10/2012	-2	78.926	10.79
ct92-M305Pete-12	120362	12/10/2012	0	78.926	10.768
ct92-M305Pete-12	120362	12/10/2012	-1	78.936	10.758
ct92-M305Pete-12	120362	12/10/2012	-1	78.917	10.801
ct92-M305Pete-12	120362	12/10/2012	-2	78.917	10.803
ct92-M305Pete-12	120362	12/10/2012	-2	78.917	10.808
ct92-M305Pete-12	120362	12/10/2012	-2	78.92	10.896
ct92-M305Pete-12	120362	12/10/2012	1	78.92	10.718
ct92-M305Pete-12	120362	12/10/2012	-1	78.923	10.747
ct92-M305Pete-12	120362	12/10/2012	-2	78.922	10.725
ct92-M305Pete-12	120362	12/10/2012	-1	78.924	10.723
ct92-M305Pete-12	120362	12/10/2012	-2	78.931	10.723
ct92-M305Pete-12	120362	12/10/2012	-1	78.929	10.703
ct92-M305Pete-12	120362	12/10/2012	-2	78.938	10.68
ct92-M305Pete-12	120362	12/10/2012	-2	78.928	10.724
ct92-M305Pete-12	120362	12/10/2012	-1	78.924	10.642
ct92-M305Pete-12	120362	12/10/2012	1	78.918	10.644
ct92-M305Pete-12	120362	12/10/2012	-1	78.916	10.625
ct92-M305Pete-12	120362	12/10/2012	-2	78.918	10.584
ct92-M305Pete-12	120362	12/10/2012	-2	78.927	10.744
ct92-M305Pete-12	120362	13/10/2012	-2	78.932	10.778
ct92-M305Pete-12	120362	13/10/2012	-2	78.936	10.65
ct92-M305Pete-12	120362	13/10/2012	-2	78.931	10.648
ct92-M305Pete-12	120362	13/10/2012	-2	78.943	10.646
ct92-M305Pete-12	120362	13/10/2012	-2	78.94	10.662
ct92-M305Pete-12	120362	13/10/2012	-2	78.935	10.621
ct92-M305Pete-12	120362	13/10/2012	-2	78.933	10.671
ct92-M305Pete-12	120362	13/10/2012	-2	78.92	10.631
ct92-M305Pete-12	120362	13/10/2012	-2	78.919	10.636
ct92-M305Pete-12	120362	13/10/2012	-2	78.92	10.68
ct92-M305Pete-12	120362	13/10/2012	-2	78.924	10.66
ct92-M305Pete-12	120362	13/10/2012	-2	78.919	10.665
ct92-M305Pete-12	120362	13/10/2012	-1	78.92	10.636
ct92-M305Pete-12	120362	13/10/2012	0	78.92	10.61
ct92-M305Pete-12	120362	13/10/2012	-2	78.921	10.561
ct92-M305Pete-12	120362	13/10/2012	-2	78.931	10.569
ct92-M305Pete-12	120362	13/10/2012	-2	78.919	10.539
ct92-M305Pete-12	120362	13/10/2012	-2	78.933	10.563
ct92-M305Pete-12	120362	13/10/2012	-2	78.91	10.637
ct92-M305Pete-12	120362	13/10/2012	-2	78.911	10.605
ct92-M305Pete-12	120362	13/10/2012	-2	78.912	10.602
ct92-M305Pete-12	120362	13/10/2012	-2	78.922	10.603
ct92-M305Pete-12	120362	13/10/2012	0	78.906	10.713
ct92-M305Pete-12	120362	13/10/2012	-2	78.91	10.592
ct92-M305Pete-12	120362	13/10/2012	-2	78.912	10.615
ct92-M305Pete-12	120362	13/10/2012	-2	78.912	10.618
ct92-M305Pete-12	120362	13/10/2012	-2	78.912	10.618
ct92-M305Pete-12	120362	13/10/2012	-1	78.909	10.598
ct92-M305Pete-12	120362	13/10/2012	0	78.928	10.639
ct92-M305Pete-12	120362	13/10/2012	-2	78.92	10.687
ct92-M305Pete-12	120362	13/10/2012	-2	78.915	10.563
ct92-M305Pete-12	120362	13/10/2012	-2	78.889	10.705
ct92-M305Pete-12	120362	13/10/2012	-1	78.927	10.554
ct92-M305Pete-12	120362	13/10/2012	-2	78.909	10.67
ct92-M305Pete-12	120362	13/10/2012	-2	78.911	10.599
ct92-M305Pete-12	120362	13/10/2012	-1	78.905	10.538
ct92-M305Pete-12	120362	13/10/2012	-2	78.911	10.628
ct92-M305Pete-12	120362	13/10/2012	-1	78.907	10.614
ct92-M305Pete-12	120362	13/10/2012	1	78.92	10.703
ct92-M305Pete-12	120362	13/10/2012	-1	78.916	10.731
ct92-M305Pete-12	120362	13/10/2012	-1	78.916	10.731
ct92-M305Pete-12	120362	13/10/2012	-1	78.915	10.731
ct92-M305Pete-12	120362	13/10/2012	-1	78.914	10.71
ct92-M305Pete-12	120362	13/10/2012	-2	78.916	10.746
ct92-M305Pete-12	120362	13/10/2012	-1	78.888	10.547

ct92-M305Pete-12	120362	13/10/2012	-2	78.915	10.757
ct92-M305Pete-12	120362	13/10/2012	-2	78.921	10.734
ct92-M305Pete-12	120362	13/10/2012	-2	78.916	10.767
ct92-M305Pete-12	120362	13/10/2012	1	78.918	10.757
ct92-M305Pete-12	120362	13/10/2012	-2	78.912	10.749
ct92-M305Pete-12	120362	13/10/2012	-2	78.911	10.76
ct92-M305Pete-12	120362	13/10/2012	-2	78.925	10.81
ct92-M305Pete-12	120362	13/10/2012	1	78.923	10.812
ct92-M305Pete-12	120362	13/10/2012	-2	78.931	10.835
ct92-M305Pete-12	120362	13/10/2012	-2	78.93	10.825
ct92-M305Pete-12	120362	13/10/2012	-2	78.911	10.615
ct92-M305Pete-12	120362	13/10/2012	-2	78.922	10.832
ct92-M305Pete-12	120362	13/10/2012	-2	78.92	10.84
ct92-M305Pete-12	120362	13/10/2012	-2	78.92	10.832
ct92-M305Pete-12	120362	13/10/2012	-1	78.909	10.818
ct92-M305Pete-12	120362	13/10/2012	-1	78.905	10.756
ct92-M305Pete-12	120362	13/10/2012	-2	78.883	10.809
ct92-M305Pete-12	120362	13/10/2012	-2	78.892	10.833
ct92-M305Pete-12	120362	13/10/2012	-2	78.894	10.838
ct92-M305Pete-12	120362	13/10/2012	-1	78.889	10.843
ct92-M305Pete-12	120362	13/10/2012	-2	78.892	10.865
ct92-M305Pete-12	120362	13/10/2012	-2	78.899	10.878
ct92-M305Pete-12	120362	14/10/2012	-2	78.903	10.911
ct92-M305Pete-12	120362	14/10/2012	-2	78.896	10.937
ct92-M305Pete-12	120362	14/10/2012	-2	78.895	10.899
ct92-M305Pete-12	120362	14/10/2012	-2	78.895	10.889
ct92-M305Pete-12	120362	14/10/2012	-2	78.891	10.868
ct92-M305Pete-12	120362	14/10/2012	-2	78.875	10.739
ct92-M305Pete-12	120362	14/10/2012	-2	78.868	10.689
ct92-M305Pete-12	120362	14/10/2012	-2	78.868	10.687
ct92-M305Pete-12	120362	14/10/2012	-2	78.853	10.799
ct92-M305Pete-12	120362	14/10/2012	-2	78.867	10.694
ct92-M305Pete-12	120362	14/10/2012	-2	78.888	10.675
ct92-M305Pete-12	120362	14/10/2012	-2	78.873	10.716
ct92-M305Pete-12	120362	14/10/2012	-2	78.877	10.705
ct92-M305Pete-12	120362	14/10/2012	-2	78.881	10.694
ct92-M305Pete-12	120362	14/10/2012	-2	78.894	10.724
ct92-M305Pete-12	120362	14/10/2012	-2	78.89	10.728
ct92-M305Pete-12	120362	14/10/2012	-1	78.885	10.869
ct92-M305Pete-12	120362	14/10/2012	-2	78.892	10.726
ct92-M305Pete-12	120362	14/10/2012	-2	78.888	10.714
ct92-M305Pete-12	120362	14/10/2012	-2	78.837	10.688
ct92-M305Pete-12	120362	14/10/2012	-2	78.892	10.682
ct92-M305Pete-12	120362	14/10/2012	-2	78.903	10.682
ct92-M305Pete-12	120362	14/10/2012	-2	78.894	10.659
ct92-M305Pete-12	120362	14/10/2012	-2	78.909	10.674
ct92-M305Pete-12	120362	14/10/2012	-2	78.908	10.675
ct92-M305Pete-12	120362	14/10/2012	-2	78.905	10.744
ct92-M305Pete-12	120362	14/10/2012	-2	78.921	10.754
ct92-M305Pete-12	120362	14/10/2012	-2	78.92	10.761
ct92-M305Pete-12	120362	14/10/2012	-2	78.92	10.762
ct92-M305Pete-12	120362	14/10/2012	-1	78.918	10.72
ct92-M305Pete-12	120362	14/10/2012	-2	78.915	10.75
ct92-M305Pete-12	120362	14/10/2012	-2	78.914	10.753
ct92-M305Pete-12	120362	14/10/2012	-2	78.915	10.753
ct92-M305Pete-12	120362	14/10/2012	-2	78.917	10.726
ct92-M305Pete-12	120362	14/10/2012	-1	78.917	10.747
ct92-M305Pete-12	120362	14/10/2012	-2	78.916	10.768
ct92-M305Pete-12	120362	14/10/2012	-2	78.914	10.778
ct92-M305Pete-12	120362	14/10/2012	-2	78.916	10.764
ct92-M305Pete-12	120362	14/10/2012	-1	78.93	10.792
ct92-M305Pete-12	120362	14/10/2012	-2	78.923	10.728
ct92-M305Pete-12	120362	14/10/2012	-2	78.927	10.755
ct92-M305Pete-12	120362	14/10/2012	-2	78.926	10.764
ct92-M305Pete-12	120362	14/10/2012	-2	78.921	10.75
ct92-M305Pete-12	120362	14/10/2012	-2	78.925	10.764
ct92-M305Pete-12	120362	14/10/2012	-1	78.918	10.753
ct92-M305Pete-12	120362	14/10/2012	-2	78.917	10.751

ct92-M305Pete-12	120362	14/10/2012	-2	78.91	10.726
ct92-M305Pete-12	120362	14/10/2012	-1	78.916	10.686
ct92-M305Pete-12	120362	14/10/2012	-2	78.912	10.706
ct92-M305Pete-12	120362	14/10/2012	1	78.916	10.763
ct92-M305Pete-12	120362	14/10/2012	-2	78.913	10.709
ct92-M305Pete-12	120362	14/10/2012	-2	78.912	10.722
ct92-M305Pete-12	120362	14/10/2012	-2	78.941	10.611
ct92-M305Pete-12	120362	14/10/2012	-2	78.904	10.716
ct92-M305Pete-12	120362	14/10/2012	-2	78.893	10.593
ct92-M305Pete-12	120362	14/10/2012	-2	78.906	10.696
ct92-M305Pete-12	120362	14/10/2012	-2	78.907	10.691
ct92-M305Pete-12	120362	14/10/2012	-1	78.929	10.826
ct92-M305Pete-12	120362	14/10/2012	-1	78.928	10.765
ct92-M305Pete-12	120362	14/10/2012	-2	78.925	10.77
ct92-M305Pete-12	120362	14/10/2012	-1	78.975	10.808
ct92-M305Pete-12	120362	14/10/2012	-1	78.96	10.773
ct92-M305Pete-12	120362	15/10/2012	-2	78.933	10.795
ct92-M305Pete-12	120362	15/10/2012	-2	78.978	10.787
ct92-M305Pete-12	120362	15/10/2012	-2	78.973	10.77
ct92-M305Pete-12	120362	15/10/2012	-2	78.975	10.794
ct92-M305Pete-12	120362	15/10/2012	-1	78.917	10.727
ct92-M305Pete-12	120362	15/10/2012	-2	78.934	10.669
ct92-M305Pete-12	120362	15/10/2012	-2	78.912	10.734
ct92-M305Pete-12	120362	15/10/2012	-2	78.908	10.739
ct92-M305Pete-12	120362	15/10/2012	-2	78.915	10.731
ct92-M305Pete-12	120362	15/10/2012	-2	78.909	10.741
ct92-M305Pete-12	120362	15/10/2012	-2	78.916	10.742
ct92-M305Pete-12	120362	15/10/2012	-2	78.916	10.739
ct92-M305Pete-12	120362	15/10/2012	-2	78.915	10.746
ct92-M305Pete-12	120362	15/10/2012	-2	78.918	10.647
ct92-M305Pete-12	120362	15/10/2012	-1	78.915	10.645
ct92-M305Pete-12	120362	15/10/2012	-2	78.928	10.643
ct92-M305Pete-12	120362	15/10/2012	-2	78.925	10.728
ct92-M305Pete-12	120362	15/10/2012	-2	78.924	10.731
ct92-M305Pete-12	120362	15/10/2012	-2	78.936	10.687
ct92-M305Pete-12	120362	15/10/2012	-2	78.937	10.675
ct92-M305Pete-12	120362	15/10/2012	-1	78.933	10.702
ct92-M305Pete-12	120362	15/10/2012	1	78.94	10.613
ct92-M305Pete-12	120362	15/10/2012	-2	78.934	10.606
ct92-M305Pete-12	120362	15/10/2012	-2	78.963	10.575
ct92-M305Pete-12	120362	15/10/2012	0	78.938	10.657
ct92-M305Pete-12	120362	15/10/2012	1	78.932	10.725
ct92-M305Pete-12	120362	15/10/2012	-2	78.923	10.722
ct92-M305Pete-12	120362	15/10/2012	1	78.909	10.721
ct92-M305Pete-12	120362	15/10/2012	2	78.915	10.736
ct92-M305Pete-12	120362	15/10/2012	-1	78.914	10.776
ct92-M305Pete-12	120362	15/10/2012	-2	78.91	10.746
ct92-M305Pete-12	120362	15/10/2012	-1	78.907	10.745
ct92-M305Pete-12	120362	15/10/2012	-1	78.92	10.719
ct92-M305Pete-12	120362	15/10/2012	-2	78.915	10.81
ct92-M305Pete-12	120362	15/10/2012	-2	78.918	10.798
ct92-M305Pete-12	120362	15/10/2012	-2	78.918	10.746
ct92-M305Pete-12	120362	15/10/2012	-1	78.917	10.726
ct92-M305Pete-12	120362	15/10/2012	1	78.934	10.738
ct92-M305Pete-12	120362	15/10/2012	-1	78.924	10.72
ct92-M305Pete-12	120362	15/10/2012	0	78.908	10.741
ct92-M305Pete-12	120362	15/10/2012	-2	78.917	10.736
ct92-M305Pete-12	120362	15/10/2012	0	78.934	10.829
ct92-M305Pete-12	120362	15/10/2012	-2	78.929	10.833
ct92-M305Pete-12	120362	15/10/2012	-1	78.926	10.797
ct92-M305Pete-12	120362	15/10/2012	-2	78.92	10.766
ct92-M305Pete-12	120362	15/10/2012	-2	78.91	10.774
ct92-M305Pete-12	120362	15/10/2012	-2	78.919	10.711
ct92-M305Pete-12	120362	15/10/2012	0	78.929	10.755
ct92-M305Pete-12	120362	15/10/2012	-2	78.93	10.708
ct92-M305Pete-12	120362	15/10/2012	-2	78.926	10.734
ct92-M305Pete-12	120362	15/10/2012	-2	78.93	10.696
ct92-M305Pete-12	120362	15/10/2012	-2	78.919	10.695

ct92-M305Pete-12	120362	15/10/2012	-1	78.928	10.615
ct92-M305Pete-12	120362	15/10/2012	-1	78.928	10.671
ct92-M305Pete-12	120362	15/10/2012	-2	78.925	10.637
ct92-M305Pete-12	120362	15/10/2012	-1	78.932	10.646
ct92-M305Pete-12	120362	15/10/2012	-2	78.926	10.647
ct92-M305Pete-12	120362	15/10/2012	-2	78.923	10.706
ct92-M305Pete-12	120362	15/10/2012	-2	78.927	10.642
ct92-M305Pete-12	120362	15/10/2012	-2	78.925	10.641
ct92-M305Pete-12	120362	15/10/2012	-2	78.925	10.644
ct92-M305Pete-12	120362	15/10/2012	-1	78.934	10.6
ct92-M305Pete-12	120362	15/10/2012	-2	78.911	10.668
ct92-M305Pete-12	120362	15/10/2012	-2	78.916	10.676
ct92-M305Pete-12	120362	15/10/2012	1	78.916	10.589
ct92-M305Pete-12	120362	15/10/2012	-2	78.91	10.571
ct92-M305Pete-12	120362	15/10/2012	-2	78.903	10.545
ct92-M305Pete-12	120362	15/10/2012	-2	78.902	10.557
ct92-M305Pete-12	120362	16/10/2012	-2	78.903	10.556
ct92-M305Pete-12	120362	16/10/2012	-2	78.902	10.552
ct92-M305Pete-12	120362	16/10/2012	-2	78.9	10.548
ct92-M305Pete-12	120362	16/10/2012	-2	78.91	10.586
ct92-M305Pete-12	120362	16/10/2012	-2	78.908	10.579
ct92-M305Pete-12	120362	16/10/2012	-2	78.904	10.56
ct92-M305Pete-12	120362	16/10/2012	-1	78.9	10.783
ct92-M305Pete-12	120362	16/10/2012	-2	78.907	10.748
ct92-M305Pete-12	120362	16/10/2012	-2	78.907	10.747
ct92-M305Pete-12	120362	16/10/2012	-2	78.923	10.697
ct92-M305Pete-12	120362	16/10/2012	-2	78.937	10.632
ct92-M305Pete-12	120362	16/10/2012	-2	78.938	10.617
ct92-M305Pete-12	120362	16/10/2012	-2	78.92	10.628
ct92-M305Pete-12	120362	16/10/2012	-2	78.924	10.714
ct92-M305Pete-12	120362	16/10/2012	-2	78.921	10.731
ct92-M305Pete-12	120362	16/10/2012	-1	78.909	10.742
ct92-M305Pete-12	120362	16/10/2012	-2	78.933	10.684
ct92-M305Pete-12	120362	16/10/2012	-2	78.932	10.686
ct92-M305Pete-12	120362	16/10/2012	-2	78.921	10.702
ct92-M305Pete-12	120362	16/10/2012	-1	78.919	10.759
ct92-M305Pete-12	120362	16/10/2012	1	78.903	10.701
ct92-M305Pete-12	120362	16/10/2012	-2	78.908	10.718
ct92-M305Pete-12	120362	16/10/2012	-2	78.91	10.715
ct92-M305Pete-12	120362	16/10/2012	-2	78.934	10.649
ct92-M305Pete-12	120362	16/10/2012	-2	78.933	10.654
ct92-M305Pete-12	120362	16/10/2012	-1	78.888	10.645
ct92-M305Pete-12	120362	16/10/2012	-1	78.917	10.714
ct92-M305Pete-12	120362	16/10/2012	-2	78.916	10.707
ct92-M305Pete-12	120362	16/10/2012	-2	78.911	10.71
ct92-M305Pete-12	120362	16/10/2012	-1	78.912	10.663
ct92-M305Pete-12	120362	16/10/2012	-1	78.913	10.677
ct92-M305Pete-12	120362	16/10/2012	-1	78.906	10.706
ct92-M305Pete-12	120362	16/10/2012	-1	78.906	10.707
ct92-M305Pete-12	120362	16/10/2012	-1	78.905	10.683
ct92-M305Pete-12	120362	16/10/2012	-2	78.909	10.699
ct92-M305Pete-12	120362	16/10/2012	-2	78.908	10.692
ct92-M305Pete-12	120362	16/10/2012	-2	78.91	10.76
ct92-M305Pete-12	120362	16/10/2012	-2	78.916	10.69
ct92-M305Pete-12	120362	16/10/2012	-1	78.916	10.69
ct92-M305Pete-12	120362	16/10/2012	-1	78.912	10.712
ct92-M305Pete-12	120362	16/10/2012	-2	78.914	10.684
ct92-M305Pete-12	120362	16/10/2012	-2	78.917	10.693
ct92-M305Pete-12	120362	16/10/2012	1	78.908	10.79
ct92-M305Pete-12	120362	16/10/2012	-1	78.919	10.753
ct92-M305Pete-12	120362	16/10/2012	-1	78.919	10.774
ct92-M305Pete-12	120362	16/10/2012	0	78.922	10.814
ct92-M305Pete-12	120362	16/10/2012	1	78.903	10.736
ct92-M305Pete-12	120362	16/10/2012	-1	78.906	10.748
ct92-M305Pete-12	120362	16/10/2012	1	78.915	10.696
ct92-M305Pete-12	120362	16/10/2012	-1	78.927	10.734
ct92-M305Pete-12	120362	16/10/2012	-1	78.928	10.733
ct92-M305Pete-12	120362	16/10/2012	-2	78.91	10.835

ct92-M305Pete-12	120362	16/10/2012	1	78.92	10.705
ct92-M305Pete-12	120362	16/10/2012	-1	78.923	10.729
ct92-M305Pete-12	120362	16/10/2012	-2	78.915	10.707
ct92-M305Pete-12	120362	16/10/2012	-2	78.925	10.691
ct92-M305Pete-12	120362	16/10/2012	-2	78.927	10.712
ct92-M305Pete-12	120362	16/10/2012	-1	78.931	10.612
ct92-M305Pete-12	120362	16/10/2012	-2	78.936	10.661
ct92-M305Pete-12	120362	16/10/2012	-2	78.933	10.6
ct92-M305Pete-12	120362	16/10/2012	0	78.936	10.612
ct92-M305Pete-12	120362	16/10/2012	-2	78.929	10.599
ct92-M305Pete-12	120362	16/10/2012	-2	78.942	10.607
ct92-M305Pete-12	120362	16/10/2012	-2	78.92	10.552
ct92-M305Pete-12	120362	16/10/2012	-2	78.92	10.547
ct92-M305Pete-12	120362	16/10/2012	-1	78.918	10.465
ct92-M305Pete-12	120362	16/10/2012	-1	78.917	10.463
ct92-M305Pete-12	120362	16/10/2012	-2	78.924	10.429
ct92-M305Pete-12	120362	16/10/2012	-2	78.913	10.437
ct92-M305Pete-12	120362	16/10/2012	-2	78.914	10.445
ct92-M305Pete-12	120362	16/10/2012	-2	78.911	10.462
ct92-M305Pete-12	120362	17/10/2012	-2	78.946	10.384
ct92-M305Pete-12	120362	17/10/2012	-2	78.912	10.462
ct92-M305Pete-12	120362	17/10/2012	-2	78.904	10.46
ct92-M305Pete-12	120362	17/10/2012	-2	78.914	10.55
ct92-M305Pete-12	120362	17/10/2012	-2	78.941	10.617
ct92-M305Pete-12	120362	17/10/2012	-2	78.917	10.585
ct92-M305Pete-12	120362	17/10/2012	-2	78.932	10.625
ct92-M305Pete-12	120362	17/10/2012	-2	78.919	10.602
ct92-M305Pete-12	120362	17/10/2012	-2	78.944	10.643
ct92-M305Pete-12	120362	17/10/2012	-1	78.933	10.751
ct92-M305Pete-12	120362	17/10/2012	-2	78.92	10.735
ct92-M305Pete-12	120362	17/10/2012	-2	78.921	10.762
ct92-M305Pete-12	120362	17/10/2012	-2	78.913	10.792
ct92-M305Pete-12	120362	17/10/2012	-2	78.915	10.816
ct92-M305Pete-12	120362	17/10/2012	-1	78.906	10.875
ct92-M305Pete-12	120362	17/10/2012	-1	78.905	10.873
ct92-M305Pete-12	120362	17/10/2012	-1	78.907	10.786
ct92-M305Pete-12	120362	17/10/2012	-2	78.908	10.796
ct92-M305Pete-12	120362	17/10/2012	-2	78.895	10.829
ct92-M305Pete-12	120362	17/10/2012	-1	78.902	10.808
ct92-M305Pete-12	120362	17/10/2012	-2	78.903	10.827
ct92-M305Pete-12	120362	17/10/2012	-1	78.907	10.792
ct92-M305Pete-12	120362	17/10/2012	-1	78.916	10.799
ct92-M305Pete-12	120362	17/10/2012	-1	78.914	10.823
ct92-M305Pete-12	120362	17/10/2012	-2	78.913	10.828
ct92-M305Pete-12	120362	17/10/2012	-2	78.913	10.817
ct92-M305Pete-12	120362	17/10/2012	-2	78.907	10.802
ct92-M305Pete-12	120362	17/10/2012	-1	78.918	10.773
ct92-M305Pete-12	120362	17/10/2012	-2	78.919	10.772
ct92-M305Pete-12	120362	17/10/2012	-2	78.91	10.761
ct92-M305Pete-12	120362	17/10/2012	-1	78.915	10.76
ct92-M305Pete-12	120362	17/10/2012	-1	78.898	10.826
ct92-M305Pete-12	120362	17/10/2012	0	78.905	10.773
ct92-M305Pete-12	120362	17/10/2012	-1	78.911	10.757
ct92-M305Pete-12	120362	17/10/2012	1	78.914	10.741
ct92-M305Pete-12	120362	17/10/2012	-2	78.909	10.765
ct92-M305Pete-12	120362	17/10/2012	1	78.916	10.756
ct92-M305Pete-12	120362	17/10/2012	-1	78.912	10.808
ct92-M305Pete-12	120362	17/10/2012	0	78.914	10.792
ct92-M305Pete-12	120362	17/10/2012	0	78.914	10.792
ct92-M305Pete-12	120362	17/10/2012	-2	78.916	10.79
ct92-M305Pete-12	120362	17/10/2012	-2	78.924	10.798
ct92-M305Pete-12	120362	17/10/2012	-1	78.931	10.656
ct92-M305Pete-12	120362	17/10/2012	-1	78.916	10.695
ct92-M305Pete-12	120362	17/10/2012	-1	78.925	10.732
ct92-M305Pete-12	120362	17/10/2012	-2	78.924	10.731
ct92-M305Pete-12	120362	17/10/2012	-1	78.925	10.707
ct92-M305Pete-12	120362	17/10/2012	0	78.903	10.727
ct92-M305Pete-12	120362	17/10/2012	-1	78.911	10.764

ct92-M305Pete-12	120362	17/10/2012	2	78.921	10.774
ct92-M305Pete-12	120362	17/10/2012	1	78.913	10.767
ct92-M305Pete-12	120362	17/10/2012	-2	78.914	10.743
ct92-M305Pete-12	120362	17/10/2012	-2	78.915	10.74
ct92-M305Pete-12	120362	17/10/2012	-2	78.902	10.753
ct92-M305Pete-12	120362	17/10/2012	-1	78.929	10.971
ct92-M305Pete-12	120362	17/10/2012	-2	78.926	10.97
ct92-M305Pete-12	120362	17/10/2012	-2	78.938	10.903
ct92-M305Pete-12	120362	17/10/2012	-1	78.927	10.916
ct92-M305Pete-12	120362	17/10/2012	-2	78.924	10.891
ct92-M305Pete-12	120362	17/10/2012	-2	78.914	10.883
ct92-M305Pete-12	120362	17/10/2012	-2	78.913	10.885
ct92-M305Pete-12	120362	17/10/2012	-2	78.904	10.914
ct92-M305Pete-12	120362	17/10/2012	-1	78.884	10.864
ct92-M305Pete-12	120362	17/10/2012	-2	78.889	10.917
ct92-M305Pete-12	120362	18/10/2012	-2	78.887	10.92
ct92-M305Pete-12	120362	18/10/2012	-2	78.888	10.918
ct92-M305Pete-12	120362	18/10/2012	-1	78.878	10.866
ct92-M305Pete-12	120362	18/10/2012	0	78.876	10.781
ct92-M305Pete-12	120362	18/10/2012	-2	78.871	10.784
ct92-M305Pete-12	120362	18/10/2012	-2	78.863	10.816
ct92-M305Pete-12	120362	18/10/2012	-2	78.871	10.816
ct92-M305Pete-12	120362	18/10/2012	-2	78.864	10.805
ct92-M305Pete-12	120362	18/10/2012	-2	78.897	10.687
ct92-M305Pete-12	120362	18/10/2012	-2	78.864	10.785
ct92-M305Pete-12	120362	18/10/2012	-1	78.902	10.741
ct92-M305Pete-12	120362	18/10/2012	-2	78.905	10.734
ct92-M305Pete-12	120362	18/10/2012	-2	78.899	10.742
ct92-M305Pete-12	120362	18/10/2012	-2	78.91	10.755
ct92-M305Pete-12	120362	18/10/2012	-2	78.911	10.747
ct92-M305Pete-12	120362	18/10/2012	-1	78.915	10.733
ct92-M305Pete-12	120362	18/10/2012	-2	78.917	10.73
ct92-M305Pete-12	120362	18/10/2012	-2	78.906	10.77
ct92-M305Pete-12	120362	18/10/2012	-2	78.917	10.702
ct92-M305Pete-12	120362	18/10/2012	-2	78.926	10.735
ct92-M305Pete-12	120362	18/10/2012	-2	78.928	10.74
ct92-M305Pete-12	120362	18/10/2012	-2	78.929	10.733
ct92-M305Pete-12	120362	18/10/2012	-2	78.925	10.755
ct92-M305Pete-12	120362	18/10/2012	-2	78.912	10.749
ct92-M305Pete-12	120362	18/10/2012	-2	78.907	10.73
ct92-M305Pete-12	120362	18/10/2012	-2	78.912	10.735
ct92-M305Pete-12	120362	18/10/2012	-1	78.912	10.743
ct92-M305Pete-12	120362	18/10/2012	-2	78.914	10.744
ct92-M305Pete-12	120362	18/10/2012	-1	78.921	10.769
ct92-M305Pete-12	120362	18/10/2012	-2	78.918	10.778
ct92-M305Pete-12	120362	18/10/2012	-2	78.92	10.769
ct92-M305Pete-12	120362	18/10/2012	0	78.912	10.665
ct92-M305Pete-12	120362	18/10/2012	0	78.94	10.676
ct92-M305Pete-12	120362	18/10/2012	1	78.917	10.745
ct92-M305Pete-12	120362	18/10/2012	-2	78.919	10.663
ct92-M305Pete-12	120362	18/10/2012	0	78.93	10.697
ct92-M305Pete-12	120362	18/10/2012	-2	78.92	10.716
ct92-M305Pete-12	120362	18/10/2012	-1	78.908	10.701
ct92-M305Pete-12	120362	18/10/2012	-2	78.903	10.692
ct92-M305Pete-12	120362	18/10/2012	-1	78.909	10.731
ct92-M305Pete-12	120362	18/10/2012	-1	78.913	10.776
ct92-M305Pete-12	120362	18/10/2012	-2	78.91	10.752
ct92-M305Pete-12	120362	18/10/2012	-2	78.912	10.765
ct92-M305Pete-12	120362	18/10/2012	-2	78.913	10.741
ct92-M305Pete-12	120362	18/10/2012	-1	78.912	10.769
ct92-M305Pete-12	120362	18/10/2012	-2	78.912	10.782
ct92-M305Pete-12	120362	18/10/2012	-2	78.921	10.859
ct92-M305Pete-12	120362	18/10/2012	0	78.921	10.82
ct92-M305Pete-12	120362	18/10/2012	-2	78.906	10.727
ct92-M305Pete-12	120362	18/10/2012	-2	78.907	10.728
ct92-M305Pete-12	120362	18/10/2012	-2	78.913	10.737
ct92-M305Pete-12	120362	18/10/2012	-1	78.915	10.77
ct92-M305Pete-12	120362	18/10/2012	-2	78.913	10.736

ct92-M305Pete-12	120362	18/10/2012	-2	78.914	10.745
ct92-M305Pete-12	120362	18/10/2012	-2	78.924	10.699
ct92-M305Pete-12	120362	18/10/2012	-1	78.946	10.768
ct92-M305Pete-12	120362	18/10/2012	-2	78.939	10.816
ct92-M305Pete-12	120362	18/10/2012	2	78.917	10.71
ct92-M305Pete-12	120362	18/10/2012	-2	78.917	10.706
ct92-M305Pete-12	120362	18/10/2012	-2	78.926	10.752
ct92-M305Pete-12	120362	18/10/2012	-1	78.921	10.708
ct92-M305Pete-12	120362	18/10/2012	-1	78.904	10.726
ct92-M305Pete-12	120362	18/10/2012	-2	78.905	10.721
ct92-M305Pete-12	120362	18/10/2012	-1	78.921	10.607
ct92-M305Pete-12	120362	18/10/2012	-2	78.875	10.761
ct92-M305Pete-12	120362	18/10/2012	-2	78.874	10.777
ct92-M305Pete-12	120362	19/10/2012	-2	78.884	10.768
ct92-M305Pete-12	120362	19/10/2012	-2	78.863	10.815
ct92-M305Pete-12	120362	19/10/2012	-2	78.866	10.796
ct92-M305Pete-12	120362	19/10/2012	-2	78.886	10.795
ct92-M305Pete-12	120362	19/10/2012	-2	78.879	10.733
ct92-M305Pete-12	120362	19/10/2012	-2	78.88	10.734
ct92-M305Pete-12	120362	19/10/2012	-2	78.88	10.781
ct92-M305Pete-12	120362	19/10/2012	-2	78.879	10.808
ct92-M305Pete-12	120362	19/10/2012	-2	78.909	10.84
ct92-M305Pete-12	120362	19/10/2012	-2	78.891	10.739
ct92-M305Pete-12	120362	19/10/2012	-1	78.911	10.77
ct92-M305Pete-12	120362	19/10/2012	-2	78.913	10.784
ct92-M305Pete-12	120362	19/10/2012	-2	78.914	10.802
ct92-M305Pete-12	120362	19/10/2012	-1	78.933	10.783
ct92-M305Pete-12	120362	19/10/2012	-2	78.934	10.806
ct92-M305Pete-12	120362	19/10/2012	-2	78.9	10.799
ct92-M305Pete-12	120362	19/10/2012	-2	78.901	10.781
ct92-M305Pete-12	120362	19/10/2012	-1	78.921	10.718
ct92-M305Pete-12	120362	19/10/2012	-2	78.915	10.792
ct92-M305Pete-12	120362	19/10/2012	-2	78.913	10.777
ct92-M305Pete-12	120362	19/10/2012	-2	78.916	10.787
ct92-M305Pete-12	120362	19/10/2012	-2	78.92	10.801
ct92-M305Pete-12	120362	19/10/2012	-2	78.921	10.79
ct92-M305Pete-12	120362	19/10/2012	-2	78.917	10.735
ct92-M305Pete-12	120362	19/10/2012	-1	78.918	10.718
ct92-M305Pete-12	120362	19/10/2012	-2	78.917	10.747
ct92-M305Pete-12	120362	19/10/2012	0	78.914	10.746
ct92-M305Pete-12	120362	19/10/2012	-2	78.916	10.74
ct92-M305Pete-12	120362	19/10/2012	-1	78.921	10.744
ct92-M305Pete-12	120362	19/10/2012	-1	78.928	10.773
ct92-M305Pete-12	120362	19/10/2012	-1	78.906	10.739
ct92-M305Pete-12	120362	19/10/2012	-1	78.921	10.734
ct92-M305Pete-12	120362	19/10/2012	1	78.908	10.744
ct92-M305Pete-12	120362	19/10/2012	-1	78.924	10.718
ct92-M305Pete-12	120362	19/10/2012	2	78.921	10.703
ct92-M305Pete-12	120362	19/10/2012	1	78.92	10.781
ct92-M305Pete-12	120362	19/10/2012	1	78.919	10.776
ct92-M305Pete-12	120362	19/10/2012	-1	78.912	10.685
ct92-M305Pete-12	120362	19/10/2012	-1	78.92	10.761
ct92-M305Pete-12	120362	19/10/2012	-1	78.908	10.777
ct92-M305Pete-12	120362	19/10/2012	0	78.911	10.788
ct92-M305Pete-12	120362	19/10/2012	-2	78.91	10.801
ct92-M305Pete-12	120362	19/10/2012	-2	78.908	10.815
ct92-M305Pete-12	120362	19/10/2012	-1	78.895	10.842
ct92-M305Pete-12	120362	19/10/2012	-2	78.911	10.805
ct92-M305Pete-12	120362	19/10/2012	-2	78.921	10.892
ct92-M305Pete-12	120362	19/10/2012	0	78.899	10.784
ct92-M305Pete-12	120362	19/10/2012	-2	78.899	10.805
ct92-M305Pete-12	120362	19/10/2012	-2	78.902	10.921
ct92-M305Pete-12	120362	19/10/2012	-1	78.898	10.831
ct92-M305Pete-12	120362	19/10/2012	-1	78.887	10.938
ct92-M305Pete-12	120362	19/10/2012	-1	78.903	10.947
ct92-M305Pete-12	120362	19/10/2012	-2	78.888	10.996
ct92-M305Pete-12	120362	19/10/2012	-1	78.891	10.895
ct92-M305Pete-12	120362	19/10/2012	-1	78.898	10.97

ct92-M305Pete-12	120362	19/10/2012	-1	78.905	10.886
ct92-M305Pete-12	120362	19/10/2012	-2	78.895	10.974
ct92-M305Pete-12	120362	19/10/2012	-2	78.9	10.96
ct92-M305Pete-12	120362	19/10/2012	-2	78.892	10.955
ct92-M305Pete-12	120362	19/10/2012	-2	78.902	10.871
ct92-M305Pete-12	120362	19/10/2012	-2	78.899	11.003
ct92-M305Pete-12	120362	19/10/2012	-2	78.902	10.883
ct92-M305Pete-12	120362	19/10/2012	-2	78.887	10.858
ct92-M305Pete-12	120362	20/10/2012	-2	78.891	10.897
ct92-M305Pete-12	120362	20/10/2012	-2	78.891	10.852
ct92-M305Pete-12	120362	20/10/2012	-1	78.88	10.807
ct92-M305Pete-12	120362	20/10/2012	-2	78.884	10.81
ct92-M305Pete-12	120362	20/10/2012	-1	78.881	10.834
ct92-M305Pete-12	120362	20/10/2012	-2	78.878	10.741
ct92-M305Pete-12	120362	20/10/2012	-2	78.88	10.748
ct92-M305Pete-12	120362	20/10/2012	-2	78.882	10.791
ct92-M305Pete-12	120362	20/10/2012	-2	78.872	10.775
ct92-M305Pete-12	120362	20/10/2012	-2	78.884	10.802
ct92-M305Pete-12	120362	20/10/2012	-2	78.886	10.793
ct92-M305Pete-12	120362	20/10/2012	-2	78.886	10.812
ct92-M305Pete-12	120362	20/10/2012	-2	78.885	10.779
ct92-M305Pete-12	120362	20/10/2012	-2	78.887	10.803
ct92-M305Pete-12	120362	20/10/2012	-2	78.894	10.752
ct92-M305Pete-12	120362	20/10/2012	-2	78.906	10.817
ct92-M305Pete-12	120362	20/10/2012	-2	78.915	10.809
ct92-M305Pete-12	120362	20/10/2012	-2	78.918	10.817
ct92-M305Pete-12	120362	20/10/2012	-2	78.911	10.827
ct92-M305Pete-12	120362	20/10/2012	-1	78.919	10.833
ct92-M305Pete-12	120362	20/10/2012	-1	78.913	10.792
ct92-M305Pete-12	120362	20/10/2012	-2	78.918	10.829
ct92-M305Pete-12	120362	20/10/2012	-2	78.923	10.81
ct92-M305Pete-12	120362	20/10/2012	-2	78.92	10.804
ct92-M305Pete-12	120362	20/10/2012	-2	78.92	10.8
ct92-M305Pete-12	120362	20/10/2012	0	78.913	10.851
ct92-M305Pete-12	120362	20/10/2012	-2	78.921	10.806
ct92-M305Pete-12	120362	20/10/2012	-1	78.922	10.782
ct92-M305Pete-12	120362	20/10/2012	-2	78.913	10.761
ct92-M305Pete-12	120362	20/10/2012	-2	78.923	10.819
ct92-M305Pete-12	120362	20/10/2012	-2	78.916	10.786
ct92-M305Pete-12	120362	20/10/2012	-2	78.922	10.815
ct92-M305Pete-12	120362	20/10/2012	-2	78.941	10.901
ct92-M305Pete-12	120362	20/10/2012	-2	78.922	10.744
ct92-M305Pete-12	120362	20/10/2012	-2	78.921	10.773
ct92-M305Pete-12	120362	20/10/2012	-1	78.93	10.832
ct92-M305Pete-12	120362	20/10/2012	-1	78.934	10.841
ct92-M305Pete-12	120362	20/10/2012	0	78.903	10.684
ct92-M305Pete-12	120362	20/10/2012	0	78.902	10.686
ct92-M305Pete-12	120362	20/10/2012	0	78.899	10.736
ct92-M305Pete-12	120362	20/10/2012	0	78.903	10.779
ct92-M305Pete-12	120362	20/10/2012	-1	78.907	10.787
ct92-M305Pete-12	120362	20/10/2012	-1	78.937	10.82
ct92-M305Pete-12	120362	20/10/2012	-2	78.908	10.784
ct92-M305Pete-12	120362	20/10/2012	-2	78.927	10.786
ct92-M305Pete-12	120362	20/10/2012	-1	78.918	10.79
ct92-M305Pete-12	120362	20/10/2012	-1	78.916	10.777
ct92-M305Pete-12	120362	20/10/2012	-1	78.911	10.764
ct92-M305Pete-12	120362	20/10/2012	-1	78.909	10.757
ct92-M305Pete-12	120362	20/10/2012	-1	78.91	10.805
ct92-M305Pete-12	120362	20/10/2012	-1	78.916	10.789
ct92-M305Pete-12	120362	20/10/2012	-1	78.912	10.758
ct92-M305Pete-12	120362	20/10/2012	-1	78.915	10.785
ct92-M305Pete-12	120362	20/10/2012	-1	78.918	10.877
ct92-M305Pete-12	120362	20/10/2012	-2	78.91	10.804
ct92-M305Pete-12	120362	20/10/2012	-2	78.907	10.764
ct92-M305Pete-12	120362	20/10/2012	-1	78.908	10.755
ct92-M305Pete-12	120362	20/10/2012	-2	78.91	10.81
ct92-M305Pete-12	120362	20/10/2012	-2	78.91	10.816
ct92-M305Pete-12	120362	20/10/2012	-2	78.921	10.562

ct92-M305Pete-12	120362	20/10/2012	-2	78.897	10.82
ct92-M305Pete-12	120362	20/10/2012	-1	78.895	10.545
ct92-M305Pete-12	120362	20/10/2012	-2	78.927	10.739
ct92-M305Pete-12	120362	20/10/2012	-2	78.897	10.56
ct92-M305Pete-12	120362	20/10/2012	-2	78.902	10.601
ct92-M305Pete-12	120362	20/10/2012	0	78.908	10.479
ct92-M305Pete-12	120362	20/10/2012	-2	78.915	10.48
ct92-M305Pete-12	120362	20/10/2012	-1	78.926	10.657
ct92-M305Pete-12	120362	20/10/2012	2	78.915	10.656
ct92-M305Pete-12	120362	20/10/2012	-1	78.911	10.63
ct92-M305Pete-12	120362	20/10/2012	-2	78.915	10.734
ct92-M305Pete-12	120362	20/10/2012	-2	78.871	10.541
ct92-M305Pete-12	120362	20/10/2012	-2	78.894	10.667
ct92-M305Pete-12	120362	20/10/2012	-2	78.872	10.575
ct92-M305Pete-12	120362	20/10/2012	-2	78.895	10.783
ct92-M305Pete-12	120362	20/10/2012	-1	78.879	10.743
ct92-M305Pete-12	120362	20/10/2012	-2	78.879	10.745
ct92-M305Pete-12	120362	20/10/2012	-2	78.88	10.747
ct92-M305Pete-12	120362	20/10/2012	-2	78.887	10.761
ct92-M305Pete-12	120362	21/10/2012	-2	78.889	10.694
ct92-M305Pete-12	120362	21/10/2012	-2	78.888	10.725
ct92-M305Pete-12	120362	21/10/2012	-2	78.889	10.668
ct92-M305Pete-12	120362	21/10/2012	-2	78.889	10.667
ct92-M305Pete-12	120362	21/10/2012	-2	78.9	10.654
ct92-M305Pete-12	120362	21/10/2012	-2	78.896	10.646
ct92-M305Pete-12	120362	21/10/2012	-1	78.94	10.697
ct92-M305Pete-12	120362	21/10/2012	-2	78.897	10.596
ct92-M305Pete-12	120362	21/10/2012	-2	78.957	10.704
ct92-M305Pete-12	120362	21/10/2012	-2	78.939	10.762
ct92-M305Pete-12	120362	21/10/2012	-2	78.894	10.717
ct92-M305Pete-12	120362	21/10/2012	-2	78.938	10.743
ct92-M305Pete-12	120362	21/10/2012	-2	78.933	10.722
ct92-M305Pete-12	120362	21/10/2012	-2	78.936	10.707
ct92-M305Pete-12	120362	21/10/2012	-2	78.915	10.742
ct92-M305Pete-12	120362	21/10/2012	-2	78.896	10.696
ct92-M305Pete-12	120362	21/10/2012	-2	78.905	10.733
ct92-M305Pete-12	120362	21/10/2012	-2	78.907	10.718
ct92-M305Pete-12	120362	21/10/2012	-2	78.939	10.691
ct92-M305Pete-12	120362	21/10/2012	-2	78.938	10.69
ct92-M305Pete-12	120362	21/10/2012	-2	78.928	10.701
ct92-M305Pete-12	120362	21/10/2012	-2	78.917	10.672
ct92-M305Pete-12	120362	21/10/2012	-2	78.917	10.661
ct92-M305Pete-12	120362	21/10/2012	-2	78.918	10.677
ct92-M305Pete-12	120362	21/10/2012	-2	78.905	10.77
ct92-M305Pete-12	120362	21/10/2012	-2	78.906	10.765
ct92-M305Pete-12	120362	21/10/2012	-2	78.926	10.656
ct92-M305Pete-12	120362	21/10/2012	-2	78.906	10.772
ct92-M305Pete-12	120362	21/10/2012	-2	78.904	10.772
ct92-M305Pete-12	120362	21/10/2012	-2	78.902	10.776
ct92-M305Pete-12	120362	21/10/2012	-2	78.902	10.807
ct92-M305Pete-12	120362	21/10/2012	-2	78.904	10.831
ct92-M305Pete-12	120362	21/10/2012	-2	78.9	10.798
ct92-M305Pete-12	120362	21/10/2012	-2	78.89	10.889
ct92-M305Pete-12	120362	21/10/2012	-2	78.925	10.922
ct92-M305Pete-12	120362	21/10/2012	-2	78.898	10.937
ct92-M305Pete-12	120362	21/10/2012	-2	78.904	11.061
ct92-M305Pete-12	120362	21/10/2012	-2	78.924	10.918
ct92-M305Pete-12	120362	21/10/2012	-2	78.921	10.914
ct92-M305Pete-12	120362	21/10/2012	-1	78.927	10.874
ct92-M305Pete-12	120362	21/10/2012	-2	78.925	10.939
ct92-M305Pete-12	120362	21/10/2012	-2	78.936	11.014
ct92-M305Pete-12	120362	21/10/2012	-2	78.96	10.938
ct92-M305Pete-12	120362	21/10/2012	-1	78.923	10.773
ct92-M305Pete-12	120362	21/10/2012	-2	78.922	10.763
ct92-M305Pete-12	120362	21/10/2012	-1	78.92	10.768
ct92-M305Pete-12	120362	21/10/2012	-2	78.919	10.758
ct92-M305Pete-12	120362	21/10/2012	-2	78.92	10.756
ct92-M305Pete-12	120362	21/10/2012	-2	78.926	10.749

ct92-M305Pete-12	120362	21/10/2012	-2	78.922	10.757
ct92-M305Pete-12	120362	21/10/2012	-2	78.923	10.761
ct92-M305Pete-12	120362	21/10/2012	-2	78.924	10.768
ct92-M305Pete-12	120362	21/10/2012	-2	78.927	10.775
ct92-M305Pete-12	120362	21/10/2012	-2	78.929	10.701
ct92-M305Pete-12	120362	21/10/2012	-1	78.92	10.618
ct92-M305Pete-12	120362	21/10/2012	-2	78.92	10.667
ct92-M305Pete-12	120362	21/10/2012	-1	78.93	10.681
ct92-M305Pete-12	120362	21/10/2012	-2	78.933	10.657
ct92-M305Pete-12	120362	21/10/2012	-2	78.933	10.656
ct92-M305Pete-12	120362	21/10/2012	-2	78.935	10.669
ct92-M305Pete-12	120362	21/10/2012	-2	78.933	10.668
ct92-M305Pete-12	120362	21/10/2012	-2	78.933	10.665
ct92-M305Pete-12	120362	21/10/2012	-2	78.933	10.668
ct92-M305Pete-12	120362	21/10/2012	-2	78.933	10.679
ct92-M305Pete-12	120362	22/10/2012	-2	78.934	10.677
ct92-M305Pete-12	120362	22/10/2012	-2	78.932	10.667
ct92-M305Pete-12	120362	22/10/2012	-2	78.925	10.735
ct92-M305Pete-12	120362	22/10/2012	-2	78.922	10.702
ct92-M305Pete-12	120362	22/10/2012	-2	78.921	10.693
ct92-M305Pete-12	120362	22/10/2012	-2	78.922	10.699
ct92-M305Pete-12	120362	22/10/2012	-2	78.92	10.699
ct92-M305Pete-12	120362	22/10/2012	-2	78.92	10.709
ct92-M305Pete-12	120362	22/10/2012	-2	78.905	10.731
ct92-M305Pete-12	120362	22/10/2012	-2	78.909	10.751
ct92-M305Pete-12	120362	22/10/2012	-2	78.908	10.741
ct92-M305Pete-12	120362	22/10/2012	-2	78.909	10.745
ct92-M305Pete-12	120362	22/10/2012	-2	78.911	10.737
ct92-M305Pete-12	120362	22/10/2012	-2	78.912	10.74
ct92-M305Pete-12	120362	22/10/2012	-2	78.911	10.702
ct92-M305Pete-12	120362	22/10/2012	-2	78.914	10.714
ct92-M305Pete-12	120362	22/10/2012	-2	78.928	10.681
ct92-M305Pete-12	120362	22/10/2012	-2	78.915	10.701
ct92-M305Pete-12	120362	22/10/2012	-1	78.921	10.66
ct92-M305Pete-12	120362	22/10/2012	0	78.929	10.746
ct92-M305Pete-12	120362	22/10/2012	-2	78.911	10.679
ct92-M305Pete-12	120362	22/10/2012	-2	78.914	10.682
ct92-M305Pete-12	120362	22/10/2012	-2	78.926	10.731
ct92-M305Pete-12	120362	22/10/2012	-2	78.927	10.727
ct92-M305Pete-12	120362	22/10/2012	-2	78.926	10.74
ct92-M305Pete-12	120362	22/10/2012	-2	78.925	10.738
ct92-M305Pete-12	120362	22/10/2012	-2	78.924	10.726
ct92-M305Pete-12	120362	22/10/2012	-2	78.923	10.717
ct92-M305Pete-12	120362	22/10/2012	-2	78.923	10.718
ct92-M305Pete-12	120362	22/10/2012	-2	78.923	10.707
ct92-M305Pete-12	120362	22/10/2012	-2	78.931	10.761
ct92-M305Pete-12	120362	22/10/2012	-2	78.931	10.758
ct92-M305Pete-12	120362	22/10/2012	-2	78.932	10.753
ct92-M305Pete-12	120362	22/10/2012	-2	78.932	10.745
ct92-M305Pete-12	120362	22/10/2012	-2	78.931	10.742
ct92-M305Pete-12	120362	22/10/2012	-2	78.931	10.736
ct92-M305Pete-12	120362	22/10/2012	-2	78.931	10.716
ct92-M305Pete-12	120362	22/10/2012	-2	78.932	10.731
ct92-M305Pete-12	120362	22/10/2012	-2	78.935	10.679
ct92-M305Pete-12	120362	22/10/2012	-2	78.925	10.643
ct92-M305Pete-12	120362	22/10/2012	-2	78.924	10.658
ct92-M305Pete-12	120362	22/10/2012	-2	78.919	10.736
ct92-M305Pete-12	120362	22/10/2012	-2	78.919	10.741
ct92-M305Pete-12	120362	22/10/2012	-2	78.92	10.742
ct92-M305Pete-12	120362	22/10/2012	-1	78.931	10.667
ct92-M305Pete-12	120362	22/10/2012	-2	78.91	11.136
ct92-M305Pete-12	120362	22/10/2012	0	78.934	10.855
ct92-M305Pete-12	120362	22/10/2012	-1	78.953	10.849
ct92-M305Pete-12	120362	22/10/2012	-1	78.943	10.807
ct92-M305Pete-12	120362	22/10/2012	-1	78.936	10.704
ct92-M305Pete-12	120362	22/10/2012	-2	78.921	10.514
ct92-M305Pete-12	120362	22/10/2012	0	78.919	10.632
ct92-M305Pete-12	120362	22/10/2012	-2	78.913	10.598

ct92-M305Pete-12	120362	22/10/2012	-2	78.912	10.602
ct92-M305Pete-12	120362	22/10/2012	-2	78.912	10.598
ct92-M305Pete-12	120362	22/10/2012	-2	78.912	10.583
ct92-M305Pete-12	120362	22/10/2012	-2	78.911	10.559
ct92-M305Pete-12	120362	22/10/2012	-2	78.913	10.564
ct92-M305Pete-12	120362	22/10/2012	2	78.921	10.621
ct92-M305Pete-12	120362	22/10/2012	-2	78.92	10.625
ct92-M305Pete-12	120362	22/10/2012	-2	78.921	10.606
ct92-M305Pete-12	120362	22/10/2012	-2	78.921	10.612
ct92-M305Pete-12	120362	22/10/2012	-2	78.917	10.562
ct92-M305Pete-12	120362	22/10/2012	-2	78.914	10.533
ct92-M305Pete-12	120362	22/10/2012	-2	78.918	10.581
ct92-M305Pete-12	120362	23/10/2012	-2	78.908	10.523
ct92-M305Pete-12	120362	23/10/2012	-2	78.91	10.748
ct92-M305Pete-12	120362	23/10/2012	-2	78.91	10.748
ct92-M305Pete-12	120362	23/10/2012	-2	78.892	10.745
ct92-M305Pete-12	120362	23/10/2012	-2	78.892	10.753
ct92-M305Pete-12	120362	23/10/2012	-2	78.886	10.613
ct92-M305Pete-12	120362	23/10/2012	-2	78.895	10.616
ct92-M305Pete-12	120362	23/10/2012	-2	78.896	10.61
ct92-M305Pete-12	120362	23/10/2012	-2	78.909	10.619
ct92-M305Pete-12	120362	23/10/2012	-2	78.909	10.62
ct92-M305Pete-12	120362	23/10/2012	-2	78.904	10.63
ct92-M305Pete-12	120362	23/10/2012	-2	78.906	10.617
ct92-M305Pete-12	120362	23/10/2012	-2	78.865	10.604
ct92-M305Pete-12	120362	23/10/2012	-2	78.87	10.735
ct92-M305Pete-12	120362	23/10/2012	-2	78.889	10.723
ct92-M305Pete-12	120362	23/10/2012	-2	78.872	10.735
ct92-M305Pete-12	120362	23/10/2012	-2	78.909	10.573
ct92-M305Pete-12	120362	23/10/2012	-2	78.939	10.436
ct92-M305Pete-12	120362	23/10/2012	-2	78.939	10.44
ct92-M305Pete-12	120362	23/10/2012	-2	78.937	10.526
ct92-M305Pete-12	120362	23/10/2012	-2	78.929	10.527
ct92-M305Pete-12	120362	23/10/2012	0	78.861	10.71
ct92-M305Pete-12	120362	23/10/2012	-2	78.906	10.558
ct92-M305Pete-12	120362	23/10/2012	-2	78.872	10.604
ct92-M305Pete-12	120362	23/10/2012	-2	78.874	10.576
ct92-M305Pete-12	120362	23/10/2012	-2	78.928	10.525
ct92-M305Pete-12	120362	23/10/2012	-2	78.911	10.587
ct92-M305Pete-12	120362	23/10/2012	2	78.899	10.581
ct92-M305Pete-12	120362	23/10/2012	-1	78.914	10.569
ct92-M305Pete-12	120362	23/10/2012	-2	78.904	10.581
ct92-M305Pete-12	120362	23/10/2012	0	78.909	10.604
ct92-M305Pete-12	120362	23/10/2012	-2	78.909	10.602
ct92-M305Pete-12	120362	23/10/2012	1	78.907	10.596
ct92-M305Pete-12	120362	23/10/2012	-2	78.917	10.631
ct92-M305Pete-12	120362	23/10/2012	-1	78.899	10.525
ct92-M305Pete-12	120362	23/10/2012	-2	78.9	10.52
ct92-M305Pete-12	120362	23/10/2012	-2	78.889	10.519
ct92-M305Pete-12	120362	23/10/2012	-2	78.889	10.54
ct92-M305Pete-12	120362	23/10/2012	-1	78.907	10.607
ct92-M305Pete-12	120362	23/10/2012	-2	78.903	10.554
ct92-M305Pete-12	120362	23/10/2012	-2	78.905	10.559
ct92-M305Pete-12	120362	23/10/2012	-2	78.924	10.596
ct92-M305Pete-12	120362	23/10/2012	-2	78.924	10.565
ct92-M305Pete-12	120362	23/10/2012	-2	78.927	10.563
ct92-M305Pete-12	120362	23/10/2012	-1	78.91	10.594
ct92-M305Pete-12	120362	23/10/2012	-2	78.917	10.593
ct92-M305Pete-12	120362	23/10/2012	-2	78.917	10.601
ct92-M305Pete-12	120362	23/10/2012	1	78.932	10.614
ct92-M305Pete-12	120362	23/10/2012	-2	78.923	10.504
ct92-M305Pete-12	120362	23/10/2012	-2	78.923	10.502
ct92-M305Pete-12	120362	23/10/2012	-2	78.953	10.604
ct92-M305Pete-12	120362	23/10/2012	-2	78.945	10.59
ct92-M305Pete-12	120362	23/10/2012	-1	78.925	10.603
ct92-M305Pete-12	120362	23/10/2012	-2	78.916	10.585
ct92-M305Pete-12	120362	23/10/2012	2	78.91	10.575
ct92-M305Pete-12	120362	23/10/2012	1	78.91	10.567

ct92-M305Pete-12	120362	23/10/2012	-1	78.915	10.559
ct92-M305Pete-12	120362	23/10/2012	1	78.908	10.573
ct92-M305Pete-12	120362	23/10/2012	1	78.908	10.573
ct92-M305Pete-12	120362	24/10/2012	-1	78.906	10.582
ct92-M305Pete-12	120362	24/10/2012	-2	78.864	10.42
ct92-M305Pete-12	120362	24/10/2012	-2	78.864	10.409
ct92-M305Pete-12	120362	24/10/2012	-2	78.909	10.56
ct92-M305Pete-12	120362	24/10/2012	-2	78.893	10.564
ct92-M305Pete-12	120362	24/10/2012	-2	78.913	10.436
ct92-M305Pete-12	120362	24/10/2012	-2	78.911	10.435
ct92-M305Pete-12	120362	24/10/2012	-2	78.914	10.454
ct92-M305Pete-12	120362	24/10/2012	-2	78.917	10.552
ct92-M305Pete-12	120362	24/10/2012	-2	78.915	10.576
ct92-M305Pete-12	120362	24/10/2012	-2	78.927	10.56
ct92-M305Pete-12	120362	24/10/2012	-1	78.91	10.625
ct92-M305Pete-12	120362	24/10/2012	-2	78.906	10.531
ct92-M305Pete-12	120362	24/10/2012	-1	78.914	10.633
ct92-M305Pete-12	120362	24/10/2012	-2	78.895	10.594
ct92-M305Pete-12	120362	24/10/2012	-2	78.898	10.591
ct92-M305Pete-12	120362	24/10/2012	-2	78.897	10.598
ct92-M305Pete-12	120362	24/10/2012	0	78.921	10.713
ct92-M305Pete-12	120362	24/10/2012	-2	78.878	10.639
ct92-M305Pete-12	120362	24/10/2012	-2	78.878	10.657
ct92-M305Pete-12	120362	24/10/2012	-2	78.905	10.539
ct92-M305Pete-12	120362	24/10/2012	-2	78.921	10.588
ct92-M305Pete-12	120362	24/10/2012	-2	78.921	10.585
ct92-M305Pete-12	120362	24/10/2012	-2	78.917	10.562
ct92-M305Pete-12	120362	24/10/2012	0	78.884	10.562
ct92-M305Pete-12	120362	24/10/2012	-1	78.899	10.721
ct92-M305Pete-12	120362	24/10/2012	-1	78.896	10.686
ct92-M305Pete-12	120362	24/10/2012	-1	78.892	10.682
ct92-M305Pete-12	120362	24/10/2012	-2	78.896	10.668
ct92-M305Pete-12	120362	24/10/2012	-2	78.906	10.62
ct92-M305Pete-12	120362	24/10/2012	-2	78.921	10.577
ct92-M305Pete-12	120362	24/10/2012	-2	78.914	10.558
ct92-M305Pete-12	120362	24/10/2012	-2	78.914	10.587
ct92-M305Pete-12	120362	24/10/2012	-2	78.885	10.615
ct92-M305Pete-12	120362	24/10/2012	-1	78.901	10.64
ct92-M305Pete-12	120362	24/10/2012	-2	78.901	10.615
ct92-M305Pete-12	120362	24/10/2012	-2	78.903	10.645
ct92-M305Pete-12	120362	24/10/2012	-2	78.89	10.697
ct92-M305Pete-12	120362	24/10/2012	-2	78.894	10.699
ct92-M305Pete-12	120362	24/10/2012	-2	78.886	10.672
ct92-M305Pete-12	120362	24/10/2012	-1	78.927	10.683
ct92-M305Pete-12	120362	24/10/2012	-2	78.899	10.722
ct92-M305Pete-12	120362	24/10/2012	-1	78.901	10.712
ct92-M305Pete-12	120362	24/10/2012	-2	78.901	10.713
ct92-M305Pete-12	120362	24/10/2012	2	78.915	10.678
ct92-M305Pete-12	120362	24/10/2012	-2	78.916	10.683
ct92-M305Pete-12	120362	24/10/2012	-2	78.909	10.695
ct92-M305Pete-12	120362	24/10/2012	-2	78.913	10.687
ct92-M305Pete-12	120362	24/10/2012	-1	78.916	10.611
ct92-M305Pete-12	120362	24/10/2012	-2	78.914	10.688
ct92-M305Pete-12	120362	24/10/2012	2	78.92	10.581
ct92-M305Pete-12	120362	24/10/2012	-2	78.92	10.581
ct92-M305Pete-12	120362	24/10/2012	-1	78.919	10.598
ct92-M305Pete-12	120362	24/10/2012	-2	78.907	10.593
ct92-M305Pete-12	120362	24/10/2012	-1	78.926	10.527
ct92-M305Pete-12	120362	24/10/2012	-2	78.919	10.533
ct92-M305Pete-12	120362	24/10/2012	0	78.931	10.665
ct92-M305Pete-12	120362	24/10/2012	-2	78.916	10.517
ct92-M305Pete-12	120362	24/10/2012	-2	78.92	10.534
ct92-M305Pete-12	120362	24/10/2012	-2	78.92	10.457
ct92-M305Pete-12	120362	25/10/2012	-2	78.918	10.531
ct92-M305Pete-12	120362	25/10/2012	-2	78.92	10.538
ct92-M305Pete-12	120362	25/10/2012	-2	78.907	10.585
ct92-M305Pete-12	120362	25/10/2012	-2	78.901	10.557
ct92-M305Pete-12	120362	25/10/2012	-2	78.907	10.686

ct92-M305Pete-12	120362	25/10/2012	-2	78.909	10.664
ct92-M305Pete-12	120362	25/10/2012	-2	78.916	10.61
ct92-M305Pete-12	120362	25/10/2012	-2	78.911	10.637
ct92-M305Pete-12	120362	25/10/2012	-2	78.909	10.642
ct92-M305Pete-12	120362	25/10/2012	-1	78.934	10.624
ct92-M305Pete-12	120362	25/10/2012	-2	78.931	10.63
ct92-M305Pete-12	120362	25/10/2012	-2	78.932	10.625
ct92-M305Pete-12	120362	25/10/2012	-1	78.935	10.624
ct92-M305Pete-12	120362	25/10/2012	-2	78.93	10.631
ct92-M305Pete-12	120362	25/10/2012	-2	78.925	10.628
ct92-M305Pete-12	120362	25/10/2012	-2	78.937	10.681
ct92-M305Pete-12	120362	25/10/2012	-2	78.936	10.695
ct92-M305Pete-12	120362	25/10/2012	-2	78.938	10.72
ct92-M305Pete-12	120362	25/10/2012	-2	78.938	10.728
ct92-M305Pete-12	120362	25/10/2012	-1	78.928	10.726
ct92-M305Pete-12	120362	25/10/2012	-2	78.931	10.758
ct92-M305Pete-12	120362	25/10/2012	-2	78.926	10.755
ct92-M305Pete-12	120362	25/10/2012	-2	78.926	10.772
ct92-M305Pete-12	120362	25/10/2012	-1	78.909	10.798
ct92-M305Pete-12	120362	25/10/2012	-1	78.907	10.832
ct92-M305Pete-12	120362	25/10/2012	-1	78.918	10.775
ct92-M305Pete-12	120362	25/10/2012	-2	78.914	10.776
ct92-M305Pete-12	120362	25/10/2012	-2	78.915	10.763
ct92-M305Pete-12	120362	25/10/2012	-1	78.925	10.74
ct92-M305Pete-12	120362	25/10/2012	-1	78.921	10.774
ct92-M305Pete-12	120362	25/10/2012	-2	78.92	10.756
ct92-M305Pete-12	120362	25/10/2012	-1	78.915	10.793
ct92-M305Pete-12	120362	25/10/2012	-1	78.911	10.806
ct92-M305Pete-12	120362	25/10/2012	0	78.906	10.752
ct92-M305Pete-12	120362	25/10/2012	-2	78.882	10.779
ct92-M305Pete-12	120362	25/10/2012	-2	78.929	10.749
ct92-M305Pete-12	120362	25/10/2012	-2	78.929	10.725
ct92-M305Pete-12	120362	25/10/2012	-2	78.91	10.729
ct92-M305Pete-12	120362	25/10/2012	2	78.909	10.727
ct92-M305Pete-12	120362	25/10/2012	0	78.938	10.782
ct92-M305Pete-12	120362	25/10/2012	-2	78.933	10.806
ct92-M305Pete-12	120362	25/10/2012	-2	78.922	10.728
ct92-M305Pete-12	120362	25/10/2012	-2	78.927	10.828
ct92-M305Pete-12	120362	25/10/2012	-1	78.922	10.836
ct92-M305Pete-12	120362	25/10/2012	0	78.906	10.784
ct92-M305Pete-12	120362	25/10/2012	0	78.906	10.784
ct92-M305Pete-12	120362	25/10/2012	-2	78.911	10.831
ct92-M305Pete-12	120362	25/10/2012	1	78.923	10.811
ct92-M305Pete-12	120362	25/10/2012	-2	78.937	10.778
ct92-M305Pete-12	120362	25/10/2012	-1	78.93	10.787
ct92-M305Pete-12	120362	25/10/2012	-1	78.927	10.702
ct92-M305Pete-12	120362	25/10/2012	-2	78.926	10.705
ct92-M305Pete-12	120362	25/10/2012	-2	78.928	10.759
ct92-M305Pete-12	120362	25/10/2012	-2	78.929	10.754
ct92-M305Pete-12	120362	25/10/2012	-2	78.906	10.708
ct92-M305Pete-12	120362	25/10/2012	-2	78.92	10.671
ct92-M305Pete-12	120362	25/10/2012	-1	78.915	10.576
ct92-M305Pete-12	120362	25/10/2012	-2	78.912	10.579
ct92-M305Pete-12	120362	25/10/2012	-2	78.898	10.754
ct92-M305Pete-12	120362	25/10/2012	-2	78.907	10.757
ct92-M305Pete-12	120362	25/10/2012	-2	78.902	10.832
ct92-M305Pete-12	120362	25/10/2012	-2	78.917	10.748
ct92-M305Pete-12	120362	25/10/2012	-2	78.917	10.857
ct92-M305Pete-12	120362	25/10/2012	-2	78.916	10.758
ct92-M305Pete-12	120362	25/10/2012	1	78.908	10.802
ct92-M305Pete-12	120362	25/10/2012	-1	78.907	10.778
ct92-M305Pete-12	120362	25/10/2012	0	78.906	10.757
ct92-M305Pete-12	120362	25/10/2012	-2	78.906	10.757
ct92-M305Pete-12	120362	25/10/2012	-1	78.909	10.8
ct92-M305Pete-12	120362	25/10/2012	-2	78.908	10.802
ct92-M305Pete-12	120362	25/10/2012	-2	78.885	10.839
ct92-M305Pete-12	120362	25/10/2012	-2	78.888	10.881
ct92-M305Pete-12	120362	26/10/2012	-2	78.89	10.873

ct92-M305Pete-12	120362	26/10/2012	-2	78.89	10.873
ct92-M305Pete-12	120362	26/10/2012	-2	78.895	10.747
ct92-M305Pete-12	120362	26/10/2012	-2	78.895	10.747
ct92-M305Pete-12	120362	26/10/2012	-2	78.886	10.815
ct92-M305Pete-12	120362	26/10/2012	-2	78.891	10.812
ct92-M305Pete-12	120362	26/10/2012	-2	78.892	10.81
ct92-M305Pete-12	120362	26/10/2012	-2	78.893	10.811
ct92-M305Pete-12	120362	26/10/2012	-2	78.891	10.822
ct92-M305Pete-12	120362	26/10/2012	-2	78.903	10.814
ct92-M305Pete-12	120362	26/10/2012	-2	78.887	10.845
ct92-M305Pete-12	120362	26/10/2012	-1	78.887	10.822
ct92-M305Pete-12	120362	26/10/2012	-2	78.94	10.732
ct92-M305Pete-12	120362	26/10/2012	-2	78.928	10.782
ct92-M305Pete-12	120362	26/10/2012	-2	78.924	10.813
ct92-M305Pete-12	120362	26/10/2012	-2	78.903	10.813
ct92-M305Pete-12	120362	26/10/2012	-2	78.919	10.82
ct92-M305Pete-12	120362	26/10/2012	-2	78.926	10.845
ct92-M305Pete-12	120362	26/10/2012	-2	78.924	10.851
ct92-M305Pete-12	120362	26/10/2012	-1	78.919	10.867
ct92-M305Pete-12	120362	26/10/2012	-1	78.913	10.903
ct92-M305Pete-12	120362	26/10/2012	-2	78.9	10.933
ct92-M305Pete-12	120362	26/10/2012	-2	78.899	10.929
ct92-M305Pete-12	120362	26/10/2012	-2	78.896	10.936
ct92-M305Pete-12	120362	26/10/2012	-2	78.91	10.866
ct92-M305Pete-12	120362	26/10/2012	-2	78.9	10.884
ct92-M305Pete-12	120362	26/10/2012	-2	78.896	10.88
ct92-M305Pete-12	120362	26/10/2012	-1	78.894	10.884
ct92-M305Pete-12	120362	26/10/2012	-2	78.891	10.886
ct92-M305Pete-12	120362	26/10/2012	-2	78.884	10.886
ct92-M305Pete-12	120362	26/10/2012	-2	78.903	10.846
ct92-M305Pete-12	120362	26/10/2012	-1	78.92	10.727
ct92-M305Pete-12	120362	26/10/2012	-1	78.914	10.758
ct92-M305Pete-12	120362	26/10/2012	1	78.918	10.743
ct92-M305Pete-12	120362	26/10/2012	-2	78.914	10.759
ct92-M305Pete-12	120362	26/10/2012	-2	78.897	10.687
ct92-M305Pete-12	120362	26/10/2012	-1	78.922	10.753
ct92-M305Pete-12	120362	26/10/2012	-1	78.907	10.753
ct92-M305Pete-12	120362	26/10/2012	-2	78.905	10.769
ct92-M305Pete-12	120362	26/10/2012	-2	78.905	10.763
ct92-M305Pete-12	120362	26/10/2012	-2	78.908	10.804
ct92-M305Pete-12	120362	26/10/2012	-2	78.907	10.765
ct92-M305Pete-12	120362	26/10/2012	-1	78.955	10.79
ct92-M305Pete-12	120362	26/10/2012	1	78.94	10.86
ct92-M305Pete-12	120362	26/10/2012	-1	78.976	10.914
ct92-M305Pete-12	120362	26/10/2012	-1	78.963	10.876
ct92-M305Pete-12	120362	26/10/2012	-2	78.982	10.893
ct92-M305Pete-12	120362	26/10/2012	-1	78.92	10.793
ct92-M305Pete-12	120362	26/10/2012	-1	78.949	10.843
ct92-M305Pete-12	120362	26/10/2012	-1	78.933	10.763
ct92-M305Pete-12	120362	26/10/2012	-2	78.943	10.763
ct92-M305Pete-12	120362	26/10/2012	-1	78.916	10.63
ct92-M305Pete-12	120362	26/10/2012	-2	78.924	10.633
ct92-M305Pete-12	120362	26/10/2012	-2	78.948	10.796
ct92-M305Pete-12	120362	26/10/2012	-1	78.935	10.597
ct92-M305Pete-12	120362	26/10/2012	-2	78.935	10.603
ct92-M305Pete-12	120362	26/10/2012	-2	78.924	10.638
ct92-M305Pete-12	120362	26/10/2012	1	78.947	10.686
ct92-M305Pete-12	120362	26/10/2012	-2	78.935	10.65
ct92-M305Pete-12	120362	26/10/2012	-2	78.958	10.705
ct92-M305Pete-12	120362	26/10/2012	-1	78.956	10.742
ct92-M305Pete-12	120362	26/10/2012	-2	78.953	10.745
ct92-M305Pete-12	120362	26/10/2012	-2	78.963	10.729
ct92-M305Pete-12	120362	26/10/2012	-2	78.971	10.616
ct92-M305Pete-12	120362	26/10/2012	-2	78.921	10.672
ct92-M305Pete-12	120362	26/10/2012	-2	78.919	10.678
ct92-M305Pete-12	120362	26/10/2012	-2	78.937	10.676
ct92-M305Pete-12	120362	26/10/2012	-2	78.925	10.737
ct92-M305Pete-12	120362	26/10/2012	-2	78.941	10.686

ct92-M305Pete-12	120362	26/10/2012	-1	78.946	10.585
ct92-M305Pete-12	120362	26/10/2012	-1	78.924	10.65
ct92-M305Pete-12	120362	26/10/2012	-1	78.92	10.653
ct92-M305Pete-12	120362	26/10/2012	-2	78.923	10.657
ct92-M305Pete-12	120362	27/10/2012	-2	78.919	10.621
ct92-M305Pete-12	120362	27/10/2012	-2	78.92	10.724
ct92-M305Pete-12	120362	27/10/2012	-2	78.922	10.724
ct92-M305Pete-12	120362	27/10/2012	-2	78.909	10.774
ct92-M305Pete-12	120362	27/10/2012	2	78.91	10.692
ct92-M305Pete-12	120362	27/10/2012	-2	78.904	10.667
ct92-M305Pete-12	120362	27/10/2012	-1	78.905	10.652
ct92-M305Pete-12	120362	27/10/2012	-2	78.908	10.695
ct92-M305Pete-12	120362	27/10/2012	-2	78.902	10.664
ct92-M305Pete-12	120362	27/10/2012	-2	78.895	10.704
ct92-M305Pete-12	120362	27/10/2012	-2	78.9	10.756
ct92-M305Pete-12	120362	27/10/2012	-1	78.893	10.728
ct92-M305Pete-12	120362	27/10/2012	-2	78.895	10.728
ct92-M305Pete-12	120362	27/10/2012	-2	78.901	10.683
ct92-M305Pete-12	120362	27/10/2012	-2	78.901	10.686
ct92-M305Pete-12	120362	27/10/2012	-2	78.879	10.796
ct92-M305Pete-12	120362	27/10/2012	-2	78.92	10.734
ct92-M305Pete-12	120362	27/10/2012	-2	78.924	10.766
ct92-M305Pete-12	120362	27/10/2012	-1	78.904	10.655
ct92-M305Pete-12	120362	27/10/2012	-1	78.899	10.659
ct92-M305Pete-12	120362	27/10/2012	-2	78.904	10.655
ct92-M305Pete-12	120362	27/10/2012	-2	78.906	10.656
ct92-M305Pete-12	120362	27/10/2012	-1	78.911	10.698
ct92-M305Pete-12	120362	27/10/2012	-2	78.926	10.783
ct92-M305Pete-12	120362	27/10/2012	-1	78.899	10.735
ct92-M305Pete-12	120362	27/10/2012	-2	78.917	10.752
ct92-M305Pete-12	120362	27/10/2012	-2	78.923	10.77
ct92-M305Pete-12	120362	27/10/2012	1	78.915	10.825
ct92-M305Pete-12	120362	27/10/2012	-2	78.944	10.752
ct92-M305Pete-12	120362	27/10/2012	-2	78.921	10.823
ct92-M305Pete-12	120362	27/10/2012	-2	78.916	10.785
ct92-M305Pete-12	120362	27/10/2012	1	78.906	10.847
ct92-M305Pete-12	120362	27/10/2012	-1	78.928	10.966
ct92-M305Pete-12	120362	27/10/2012	1	78.894	10.9
ct92-M305Pete-12	120362	27/10/2012	2	78.896	10.897
ct92-M305Pete-12	120362	27/10/2012	-1	78.879	10.925
ct92-M305Pete-12	120362	27/10/2012	-2	78.896	10.904
ct92-M305Pete-12	120362	27/10/2012	-1	78.878	10.929
ct92-M305Pete-12	120362	27/10/2012	-2	78.893	10.972
ct92-M305Pete-12	120362	27/10/2012	-2	78.886	10.892
ct92-M305Pete-12	120362	27/10/2012	-2	78.891	10.975
ct92-M305Pete-12	120362	27/10/2012	-2	78.893	10.982
ct92-M305Pete-12	120362	27/10/2012	-2	78.905	10.943
ct92-M305Pete-12	120362	27/10/2012	-2	78.893	10.855
ct92-M305Pete-12	120362	27/10/2012	-2	78.898	10.851
ct92-M305Pete-12	120362	27/10/2012	-2	78.899	10.851
ct92-M305Pete-12	120362	27/10/2012	-2	78.893	10.832
ct92-M305Pete-12	120362	27/10/2012	-2	78.897	10.822
ct92-M305Pete-12	120362	27/10/2012	-2	78.907	10.771
ct92-M305Pete-12	120362	27/10/2012	-2	78.907	10.767
ct92-M305Pete-12	120362	27/10/2012	-2	78.897	10.849
ct92-M305Pete-12	120362	27/10/2012	-2	78.913	10.719
ct92-M305Pete-12	120362	27/10/2012	-2	78.909	10.719
ct92-M305Pete-12	120362	27/10/2012	-2	78.908	10.755
ct92-M305Pete-12	120362	27/10/2012	0	78.919	10.691
ct92-M305Pete-12	120362	27/10/2012	-2	78.925	10.764
ct92-M305Pete-12	120362	27/10/2012	0	78.919	10.691
ct92-M305Pete-12	120362	27/10/2012	-2	78.922	10.686
ct92-M305Pete-12	120362	27/10/2012	-2	78.918	10.688
ct92-M305Pete-12	120362	28/10/2012	-2	78.927	10.671
ct92-M305Pete-12	120362	28/10/2012	-2	78.931	10.656
ct92-M305Pete-12	120362	28/10/2012	-2	78.924	10.674
ct92-M305Pete-12	120362	28/10/2012	-2	78.913	10.66
ct92-M305Pete-12	120362	28/10/2012	-2	78.91	10.633

ct92-M305Pete-12	120362	28/10/2012	-2	78.909	10.624
ct92-M305Pete-12	120362	28/10/2012	-2	78.911	10.644
ct92-M305Pete-12	120362	28/10/2012	-2	78.91	10.636
ct92-M305Pete-12	120362	28/10/2012	-2	78.908	10.658
ct92-M305Pete-12	120362	28/10/2012	-2	78.907	10.644
ct92-M305Pete-12	120362	28/10/2012	-2	78.906	10.673
ct92-M305Pete-12	120362	28/10/2012	-2	78.902	10.664
ct92-M305Pete-12	120362	28/10/2012	-2	78.9	10.651
ct92-M305Pete-12	120362	28/10/2012	-2	78.895	10.647
ct92-M305Pete-12	120362	28/10/2012	-2	78.894	10.669
ct92-M305Pete-12	120362	28/10/2012	-2	78.896	10.671
ct92-M305Pete-12	120362	28/10/2012	-2	78.895	10.669
ct92-M305Pete-12	120362	28/10/2012	-2	78.904	10.699
ct92-M305Pete-12	120362	28/10/2012	-2	78.903	10.696
ct92-M305Pete-12	120362	28/10/2012	-2	78.899	10.76
ct92-M305Pete-12	120362	28/10/2012	-2	78.897	10.747
ct92-M305Pete-12	120362	28/10/2012	-1	78.913	10.733
ct92-M305Pete-12	120362	28/10/2012	-2	78.915	10.732
ct92-M305Pete-12	120362	28/10/2012	-2	78.915	10.725
ct92-M305Pete-12	120362	28/10/2012	-2	78.914	10.727
ct92-M305Pete-12	120362	28/10/2012	-2	78.914	10.727
ct92-M305Pete-12	120362	28/10/2012	-2	78.915	10.747
ct92-M305Pete-12	120362	28/10/2012	-2	78.924	10.731
ct92-M305Pete-12	120362	28/10/2012	-2	78.926	10.757
ct92-M305Pete-12	120362	28/10/2012	-2	78.927	10.794
ct92-M305Pete-12	120362	28/10/2012	-2	78.917	10.813
ct92-M305Pete-12	120362	28/10/2012	-2	78.919	10.816
ct92-M305Pete-12	120362	28/10/2012	-2	78.922	10.785
ct92-M305Pete-12	120362	28/10/2012	0	78.928	10.722
ct92-M305Pete-12	120362	28/10/2012	0	78.931	10.665
ct92-M305Pete-12	120362	28/10/2012	-1	78.924	10.688
ct92-M305Pete-12	120362	28/10/2012	0	78.916	10.613
ct92-M305Pete-12	120362	28/10/2012	-2	78.907	10.561
ct92-M305Pete-12	120362	28/10/2012	-2	78.921	10.599
ct92-M305Pete-12	120362	28/10/2012	-2	78.923	10.603
ct92-M305Pete-12	120362	28/10/2012	-2	78.929	10.682
ct92-M305Pete-12	120362	28/10/2012	-1	78.919	10.726
ct92-M305Pete-12	120362	28/10/2012	-2	78.904	10.618
ct92-M305Pete-12	120362	28/10/2012	-2	78.918	10.721
ct92-M305Pete-12	120362	28/10/2012	-2	78.924	10.655
ct92-M305Pete-12	120362	28/10/2012	-1	78.926	10.716
ct92-M305Pete-12	120362	28/10/2012	-2	78.927	10.733
ct92-M305Pete-12	120362	28/10/2012	-1	78.94	10.686
ct92-M305Pete-12	120362	28/10/2012	-2	78.944	10.769
ct92-M305Pete-12	120362	28/10/2012	-2	78.943	10.778
ct92-M305Pete-12	120362	28/10/2012	-1	78.937	10.604
ct92-M305Pete-12	120362	28/10/2012	-1	78.91	10.746
ct92-M305Pete-12	120362	28/10/2012	-1	78.954	10.752
ct92-M305Pete-12	120362	28/10/2012	-2	78.924	10.737
ct92-M305Pete-12	120362	28/10/2012	-2	78.924	10.738
ct92-M305Pete-12	120362	28/10/2012	0	78.924	10.661
ct92-M305Pete-12	120362	28/10/2012	-1	78.919	10.57
ct92-M305Pete-12	120362	29/10/2012	-2	78.933	10.614
ct92-M305Pete-12	120362	29/10/2012	-2	78.932	10.613
ct92-M305Pete-12	120362	29/10/2012	2	78.938	10.576
ct92-M305Pete-12	120362	29/10/2012	0	78.925	10.666
ct92-M305Pete-12	120362	29/10/2012	-1	78.931	10.671
ct92-M305Pete-12	120362	29/10/2012	-2	78.93	10.671
ct92-M305Pete-12	120362	29/10/2012	-2	78.942	10.673
ct92-M305Pete-12	120362	29/10/2012	-1	78.987	10.83
ct92-M305Pete-12	120362	29/10/2012	-2	78.942	10.988
ct92-M305Pete-12	120362	29/10/2012	-2	78.938	10.908
ct92-M305Pete-12	120362	29/10/2012	-2	78.929	10.715
ct92-M305Pete-12	120362	29/10/2012	-2	78.963	10.58
ct92-M305Pete-12	120362	29/10/2012	-2	78.961	10.577
ct92-M305Pete-12	120362	29/10/2012	-2	78.956	10.552
ct92-M305Pete-12	120362	29/10/2012	-2	78.951	10.503
ct92-M305Pete-12	120362	29/10/2012	-2	78.935	10.486

ct92-M305Pete-12	120362	29/10/2012	-2	78.924	10.405
ct92-M305Pete-12	120362	29/10/2012	-2	78.891	10.556
ct92-M305Pete-12	120362	29/10/2012	-2	78.883	10.547
ct92-M305Pete-12	120362	29/10/2012	-2	78.885	10.56
ct92-M305Pete-12	120362	29/10/2012	-2	78.888	10.571
ct92-M305Pete-12	120362	29/10/2012	-2	78.875	10.52
ct92-M305Pete-12	120362	29/10/2012	-2	78.894	10.643
ct92-M305Pete-12	120362	29/10/2012	-2	78.918	10.665
ct92-M305Pete-12	120362	29/10/2012	-2	78.87	10.439
ct92-M305Pete-12	120362	29/10/2012	-2	78.891	10.695
ct92-M305Pete-12	120362	29/10/2012	-2	78.889	10.687
ct92-M305Pete-12	120362	29/10/2012	-2	78.89	10.69
ct92-M305Pete-12	120362	29/10/2012	-2	78.892	10.741
ct92-M305Pete-12	120362	29/10/2012	-2	78.899	10.741
ct92-M305Pete-12	120362	29/10/2012	-2	78.895	10.723
ct92-M305Pete-12	120362	29/10/2012	-2	78.896	10.708
ct92-M305Pete-12	120362	29/10/2012	-2	78.9	10.65
ct92-M305Pete-12	120362	29/10/2012	-2	78.895	10.633
ct92-M305Pete-12	120362	29/10/2012	-2	78.909	10.633
ct92-M305Pete-12	120362	29/10/2012	-2	78.925	10.703
ct92-M305Pete-12	120362	29/10/2012	-2	78.916	10.767
ct92-M305Pete-12	120362	29/10/2012	-2	78.919	10.782
ct92-M305Pete-12	120362	29/10/2012	-2	78.915	10.806
ct92-M305Pete-12	120362	29/10/2012	-2	78.909	10.783
ct92-M305Pete-12	120362	29/10/2012	-2	78.909	10.775
ct92-M305Pete-12	120362	29/10/2012	-2	78.909	10.769
ct92-M305Pete-12	120362	29/10/2012	-2	78.91	10.773
ct92-M305Pete-12	120362	29/10/2012	-2	78.92	10.666
ct92-M305Pete-12	120362	29/10/2012	-2	78.924	10.661
ct92-M305Pete-12	120362	29/10/2012	-2	78.924	10.631
ct92-M305Pete-12	120362	29/10/2012	-2	78.907	10.641
ct92-M305Pete-12	120362	29/10/2012	-1	78.928	10.638
ct92-M305Pete-12	120362	29/10/2012	-2	78.93	10.632
ct92-M305Pete-12	120362	29/10/2012	-2	78.928	10.62
ct92-M305Pete-12	120362	29/10/2012	-2	78.922	10.64
ct92-M305Pete-12	120362	29/10/2012	-2	78.921	10.636
ct92-M305Pete-12	120362	29/10/2012	-2	78.92	10.637
ct92-M305Pete-12	120362	29/10/2012	-2	78.92	10.619
ct92-M305Pete-12	120362	30/10/2012	-2	78.922	10.617
ct92-M305Pete-12	120362	30/10/2012	-2	78.921	10.602
ct92-M305Pete-12	120362	30/10/2012	-2	78.921	10.596
ct92-M305Pete-12	120362	30/10/2012	-2	78.916	10.596
ct92-M305Pete-12	120362	30/10/2012	-2	78.915	10.574
ct92-M305Pete-12	120362	30/10/2012	-2	78.916	10.603
ct92-M305Pete-12	120362	30/10/2012	-2	78.926	10.668
ct92-M305Pete-12	120362	30/10/2012	-2	78.92	10.683
ct92-M305Pete-12	120362	30/10/2012	-2	78.92	10.684
ct92-M305Pete-12	120362	30/10/2012	-2	78.922	10.692
ct92-M305Pete-12	120362	30/10/2012	-2	78.921	10.697
ct92-M305Pete-12	120362	30/10/2012	-2	78.924	10.707
ct92-M305Pete-12	120362	30/10/2012	-1	78.921	10.693
ct92-M305Pete-12	120362	30/10/2012	-2	78.92	10.715
ct92-M305Pete-12	120362	30/10/2012	-2	78.918	10.745
ct92-M305Pete-12	120362	30/10/2012	-2	78.926	10.752
ct92-M305Pete-12	120362	30/10/2012	-2	78.917	10.847
ct92-M305Pete-12	120362	30/10/2012	-2	78.917	10.863
ct92-M305Pete-12	120362	30/10/2012	-2	78.915	10.789
ct92-M305Pete-12	120362	30/10/2012	-2	78.914	10.812
ct92-M305Pete-12	120362	30/10/2012	-2	78.914	10.82
ct92-M305Pete-12	120362	30/10/2012	-2	78.92	10.85
ct92-M305Pete-12	120362	30/10/2012	-2	78.924	10.882
ct92-M305Pete-12	120362	30/10/2012	-2	78.919	10.822
ct92-M305Pete-12	120362	30/10/2012	0	78.919	10.799
ct92-M305Pete-12	120362	30/10/2012	1	78.92	10.785
ct92-M305Pete-12	120362	30/10/2012	-1	78.908	10.786
ct92-M305Pete-12	120362	30/10/2012	-2	78.909	10.787
ct92-M305Pete-12	120362	30/10/2012	-2	78.908	10.787
ct92-M305Pete-12	120362	30/10/2012	-2	78.916	10.764

ct92-M305Pete-12	120362	30/10/2012	-2	78.915	10.742
ct92-M305Pete-12	120362	30/10/2012	-2	78.915	10.744
ct92-M305Pete-12	120362	30/10/2012	2	78.92	10.754
ct92-M305Pete-12	120362	30/10/2012	2	78.92	10.754
ct92-M305Pete-12	120362	30/10/2012	1	78.919	10.774
ct92-M305Pete-12	120362	30/10/2012	-2	78.921	10.767
ct92-M305Pete-12	120362	30/10/2012	-2	78.919	10.759
ct92-M305Pete-12	120362	30/10/2012	-2	78.92	10.751
ct92-M305Pete-12	120362	30/10/2012	-1	78.897	10.696
ct92-M305Pete-12	120362	30/10/2012	-2	78.92	10.752
ct92-M305Pete-12	120362	30/10/2012	-2	78.923	10.727
ct92-M305Pete-12	120362	30/10/2012	-1	78.916	10.712
ct92-M305Pete-12	120362	30/10/2012	-2	78.924	10.737
ct92-M305Pete-12	120362	30/10/2012	-2	78.924	10.701
ct92-M305Pete-12	120362	30/10/2012	-2	78.928	10.687
ct92-M305Pete-12	120362	30/10/2012	-2	78.927	10.698
ct92-M305Pete-12	120362	30/10/2012	-2	78.927	10.677
ct92-M305Pete-12	120362	30/10/2012	-2	78.928	10.669
ct92-M305Pete-12	120362	30/10/2012	-2	78.925	10.661
ct92-M305Pete-12	120362	30/10/2012	-2	78.927	10.658
ct92-M305Pete-12	120362	30/10/2012	-2	78.929	10.657
ct92-M305Pete-12	120362	30/10/2012	-2	78.913	10.691
ct92-M305Pete-12	120362	30/10/2012	-2	78.911	10.686
ct92-M305Pete-12	120362	30/10/2012	-2	78.905	10.676
ct92-M305Pete-12	120362	30/10/2012	-1	78.911	10.566
ct92-M305Pete-12	120362	30/10/2012	-2	78.91	10.566
ct92-M305Pete-12	120362	30/10/2012	-1	78.911	10.566
ct92-M305Pete-12	120362	30/10/2012	-2	78.923	10.584
ct92-M305Pete-12	120362	30/10/2012	-2	78.922	10.582
ct92-M305Pete-12	120362	30/10/2012	-2	78.923	10.579
ct92-M305Pete-12	120362	30/10/2012	-2	78.912	10.751
ct92-M305Pete-12	120362	30/10/2012	-2	78.92	10.549
ct92-M305Pete-12	120362	30/10/2012	-2	78.917	10.557
ct92-M305Pete-12	120362	30/10/2012	-2	78.921	10.546
ct92-M305Pete-12	120362	30/10/2012	-2	78.927	10.65
ct92-M305Pete-12	120362	30/10/2012	-2	78.925	10.654
ct92-M305Pete-12	120362	30/10/2012	-1	78.927	10.769
ct92-M305Pete-12	120362	30/10/2012	-2	78.924	10.721
ct92-M305Pete-12	120362	30/10/2012	-2	78.907	10.649
ct92-M305Pete-12	120362	30/10/2012	-1	78.921	10.71
ct92-M305Pete-12	120362	31/10/2012	-2	78.926	10.726
ct92-M305Pete-12	120362	31/10/2012	-2	78.931	10.613
ct92-M305Pete-12	120362	31/10/2012	-2	78.916	10.805
ct92-M305Pete-12	120362	31/10/2012	-2	78.911	10.809
ct92-M305Pete-12	120362	31/10/2012	-2	78.905	10.818
ct92-M305Pete-12	120362	31/10/2012	-2	78.917	10.765
ct92-M305Pete-12	120362	31/10/2012	-2	78.917	10.764
ct92-M305Pete-12	120362	31/10/2012	-1	78.917	10.771
ct92-M305Pete-12	120362	31/10/2012	-1	78.917	10.77
ct92-M305Pete-12	120362	31/10/2012	-2	78.919	10.78
ct92-M305Pete-12	120362	31/10/2012	-2	78.917	10.773
ct92-M305Pete-12	120362	31/10/2012	-2	78.918	10.771
ct92-M305Pete-12	120362	31/10/2012	-2	78.915	10.777
ct92-M305Pete-12	120362	31/10/2012	-2	78.916	10.771
ct92-M305Pete-12	120362	31/10/2012	-1	78.911	10.756
ct92-M305Pete-12	120362	31/10/2012	1	78.911	10.824
ct92-M305Pete-12	120362	31/10/2012	-2	78.911	10.756
ct92-M305Pete-12	120362	31/10/2012	2	78.908	10.79
ct92-M305Pete-12	120362	31/10/2012	-1	78.906	10.816
ct92-M305Pete-12	120362	31/10/2012	2	78.913	10.766
ct92-M305Pete-12	120362	31/10/2012	-1	78.915	10.788
ct92-M305Pete-12	120362	31/10/2012	-2	78.901	10.778
ct92-M305Pete-12	120362	31/10/2012	-1	78.901	10.838
ct92-M305Pete-12	120362	31/10/2012	2	78.902	10.848
ct92-M305Pete-12	120362	31/10/2012	2	78.902	10.844
ct92-M305Pete-12	120362	31/10/2012	1	78.911	10.834
ct92-M305Pete-12	120362	31/10/2012	0	78.913	10.81
ct92-M305Pete-12	120362	31/10/2012	1	78.916	10.813

ct92-M305Pete-12	120362	31/10/2012	-2	78.917	10.813
ct92-M305Pete-12	120362	31/10/2012	-1	78.909	10.801
ct92-M305Pete-12	120362	31/10/2012	-2	78.918	10.782
ct92-M305Pete-12	120362	31/10/2012	-1	78.923	10.765
ct92-M305Pete-12	120362	31/10/2012	-2	78.917	10.821
ct92-M305Pete-12	120362	31/10/2012	-2	78.904	10.857
ct92-M305Pete-12	120362	31/10/2012	-1	78.903	10.774
ct92-M305Pete-12	120362	31/10/2012	0	78.907	10.824
ct92-M305Pete-12	120362	31/10/2012	0	78.923	10.725
ct92-M305Pete-12	120362	31/10/2012	-2	78.918	10.786
ct92-M305Pete-12	120362	31/10/2012	-1	78.914	10.784
ct92-M305Pete-12	120362	31/10/2012	-2	78.91	10.819
ct92-M305Pete-12	120362	31/10/2012	-2	78.91	10.816
ct92-M305Pete-12	120362	31/10/2012	-1	78.919	10.81
ct92-M305Pete-12	120362	31/10/2012	-2	78.912	10.821
ct92-M305Pete-12	120362	31/10/2012	-2	78.912	10.792
ct92-M305Pete-12	120362	31/10/2012	-1	78.907	10.812
ct92-M305Pete-12	120362	31/10/2012	-1	78.912	10.759
ct92-M305Pete-12	120362	31/10/2012	-1	78.912	10.809
ct92-M305Pete-12	120362	31/10/2012	-2	78.913	10.807
ct92-M305Pete-12	120362	31/10/2012	-2	78.914	10.809
ct92-M305Pete-12	120362	31/10/2012	-2	78.914	10.799
ct92-M305Pete-12	120362	31/10/2012	-2	78.915	10.785
ct92-M305Pete-12	120362	31/10/2012	-2	78.915	10.762
ct92-M305Pete-12	120362	31/10/2012	-2	78.921	10.763
ct92-M305Pete-12	120362	31/10/2012	-2	78.921	10.764
ct92-M305Pete-12	120362	31/10/2012	-1	78.929	10.727
ct92-M305Pete-12	120362	31/10/2012	-2	78.916	10.781
ct92-M305Pete-12	120362	31/10/2012	-2	78.915	10.791
ct92-M305Pete-12	120362	31/10/2012	-2	78.92	10.727
ct92-M305Pete-12	120362	31/10/2012	-2	78.926	10.768
ct92-M305Pete-12	120362	31/10/2012	-1	78.929	10.757
ct92-M305Pete-12	120362	31/10/2012	-2	78.926	10.75
ct92-M305Pete-12	120362	31/10/2012	-2	78.934	10.741
ct92-M305Pete-12	120362	31/10/2012	-2	78.929	10.732
ct92-M305Pete-12	120362	31/10/2012	-2	78.931	10.734
ct92-M305Pete-12	120362	31/10/2012	-2	78.934	10.689
ct92-M305Pete-12	120362	31/10/2012	0	78.929	10.766
ct92-M305Pete-12	120362	31/10/2012	0	78.93	10.765
ct92-M305Pete-12	120362	31/10/2012	-2	78.931	10.768
ct92-M305Pete-12	120362	31/10/2012	-1	78.914	10.666
ct92-M305Pete-12	120362	31/10/2012	-1	78.914	10.667
ct92-M305Pete-12	120362	31/10/2012	-2	78.918	10.684
ct92-M305Pete-12	120362	31/10/2012	0	78.936	10.742
ct92-M305Pete-12	120362	31/10/2012	0	78.936	10.747
ct92-M305Pete-12	120362	31/10/2012	-2	78.948	10.781
ct92-M305Pete-12	120362	31/10/2012	-2	78.925	10.768
ct92-M305Pete-12	120362	31/10/2012	-1	78.952	10.937
ct92-M305Pete-12	120362	31/10/2012	-2	78.917	10.744
ct92-M305Pete-12	120362	31/10/2012	-1	78.948	10.918
ct92-M305Pete-12	120362	31/10/2012	-1	78.935	10.839
ct92-M305Pete-12	120362	31/10/2012	-1	78.929	10.782
ct92-M305Pete-12	120362	31/10/2012	-2	78.936	10.855
ct92-M305Pete-12	120362	31/10/2012	-2	78.92	10.789
ct92-M305Pete-12	120362	31/10/2012	-2	78.921	10.725
ct92-M305Pete-12	120362	31/10/2012	-2	78.939	10.776
ct92-M305Pete-12	120362	31/10/2012	-2	78.941	10.78
ct92-M305Pete-12	120362	31/10/2012	-2	78.929	10.639
ct92-M305Pete-12	120362	31/10/2012	-1	78.93	10.691
ct92-M305Pete-12	120362	31/10/2012	2	78.922	10.766
ct92-M305Pete-12	120362	31/10/2012	-1	78.921	10.785
ct92-M305Pete-12	120362	31/10/2012	-1	78.925	10.778
ct92-M305Pete-12	120362	31/10/2012	-2	78.921	10.837
ct92-M305Pete-12	120362	31/10/2012	-1	78.919	10.752
ct92-M305Pete-12	120362	31/10/2012	0	78.909	10.707
ct92-M305Pete-12	120362	31/10/2012	-1	78.924	10.713
ct92-M305Pete-12	120362	31/10/2012	-2	78.909	10.707
ct92-M305Pete-12	120362	01/11/2012	-1	78.924	10.674

ct92-M305Pete-12	120362	01/11/2012	-1	78.911	10.712
ct92-M305Pete-12	120362	01/11/2012	-2	78.911	10.71
ct92-M305Pete-12	120362	01/11/2012	-2	78.897	10.665
ct92-M305Pete-12	120362	01/11/2012	-1	78.902	10.514
ct92-M305Pete-12	120362	01/11/2012	-2	78.919	10.633
ct92-M305Pete-12	120362	01/11/2012	-2	78.921	10.602
ct92-M305Pete-12	120362	01/11/2012	-2	78.922	10.617
ct92-M305Pete-12	120362	01/11/2012	-2	78.905	10.658
ct92-M305Pete-12	120362	01/11/2012	-2	78.908	10.697
ct92-M305Pete-12	120362	01/11/2012	-2	78.904	10.755
ct92-M305Pete-12	120362	01/11/2012	-2	78.903	10.757
ct92-M305Pete-12	120362	01/11/2012	-2	78.907	10.648
ct92-M305Pete-12	120362	01/11/2012	-2	78.883	10.686
ct92-M305Pete-12	120362	01/11/2012	-2	78.882	10.666
ct92-M305Pete-12	120362	01/11/2012	-2	78.887	10.649
ct92-M305Pete-12	120362	01/11/2012	-2	78.891	10.648
ct92-M305Pete-12	120362	01/11/2012	-2	78.903	10.671
ct92-M305Pete-12	120362	01/11/2012	-2	78.868	10.753
ct92-M305Pete-12	120362	01/11/2012	-2	78.879	10.709
ct92-M305Pete-12	120362	01/11/2012	-2	78.879	10.71
ct92-M305Pete-12	120362	01/11/2012	-2	78.906	10.795
ct92-M305Pete-12	120362	01/11/2012	-2	78.914	10.749
ct92-M305Pete-12	120362	01/11/2012	1	78.914	10.675
ct92-M305Pete-12	120362	01/11/2012	-2	78.911	10.691
ct92-M305Pete-12	120362	01/11/2012	-2	78.915	10.737
ct92-M305Pete-12	120362	01/11/2012	1	78.923	10.735
ct92-M305Pete-12	120362	01/11/2012	1	78.924	10.769
ct92-M305Pete-12	120362	01/11/2012	0	78.942	10.734
ct92-M305Pete-12	120362	01/11/2012	-2	78.939	10.725
ct92-M305Pete-12	120362	01/11/2012	-2	78.935	10.814
ct92-M305Pete-12	120362	01/11/2012	-2	78.933	10.809
ct92-M305Pete-12	120362	01/11/2012	-1	78.911	10.835
ct92-M305Pete-12	120362	01/11/2012	-2	78.911	10.833
ct92-M305Pete-12	120362	01/11/2012	0	78.906	10.784
ct92-M305Pete-12	120362	01/11/2012	-2	78.907	10.782
ct92-M305Pete-12	120362	01/11/2012	-2	78.904	10.811
ct92-M305Pete-12	120362	01/11/2012	-2	78.934	10.868
ct92-M305Pete-12	120362	01/11/2012	-1	78.896	10.851
ct92-M305Pete-12	120362	01/11/2012	-2	78.926	10.738
ct92-M305Pete-12	120362	01/11/2012	-1	78.914	10.722
ct92-M305Pete-12	120362	01/11/2012	-2	78.904	10.744
ct92-M305Pete-12	120362	01/11/2012	-2	78.923	10.713
ct92-M305Pete-12	120362	01/11/2012	1	78.924	10.649
ct92-M305Pete-12	120362	01/11/2012	-2	78.917	10.679
ct92-M305Pete-12	120362	01/11/2012	-2	78.918	10.661
ct92-M305Pete-12	120362	01/11/2012	-1	78.922	10.692
ct92-M305Pete-12	120362	01/11/2012	-2	78.917	10.707
ct92-M305Pete-12	120362	01/11/2012	-2	78.914	10.772
ct92-M305Pete-12	120362	01/11/2012	-1	78.922	10.738
ct92-M305Pete-12	120362	01/11/2012	1	78.922	10.683
ct92-M305Pete-12	120362	01/11/2012	-2	78.915	10.637
ct92-M305Pete-12	120362	01/11/2012	-1	78.926	10.647
ct92-M305Pete-12	120362	01/11/2012	-2	78.938	10.589
ct92-M305Pete-12	120362	01/11/2012	-2	78.944	10.529
ct92-M305Pete-12	120362	01/11/2012	-2	78.95	10.576
ct92-M305Pete-12	120362	01/11/2012	-2	78.955	10.532
ct92-M305Pete-12	120362	01/11/2012	-2	78.946	10.561
ct92-M305Pete-12	120362	01/11/2012	-2	78.947	10.523
ct92-M305Pete-12	120362	01/11/2012	-2	78.944	10.558
ct92-M305Pete-12	120362	01/11/2012	-2	78.944	10.521
ct92-M305Pete-12	120362	01/11/2012	1	78.928	10.558
ct92-M305Pete-12	120362	01/11/2012	-2	78.928	10.552
ct92-M305Pete-12	120362	01/11/2012	-2	78.928	10.548
ct92-M305Pete-12	120362	01/11/2012	-2	78.935	10.632
ct92-M305Pete-12	120362	01/11/2012	-1	78.947	10.652
ct92-M305Pete-12	120362	01/11/2012	-2	78.943	10.64
ct92-M305Pete-12	120362	01/11/2012	-2	78.945	10.621
ct92-M305Pete-12	120362	01/11/2012	-1	78.938	10.616

ct92-M305Pete-12	120362	01/11/2012	-2	78.938	10.609
ct92-M305Pete-12	120362	01/11/2012	-2	78.943	10.621
ct92-M305Pete-12	120362	01/11/2012	-2	78.93	10.683
ct92-M305Pete-12	120362	02/11/2012	-2	78.938	10.635
ct92-M305Pete-12	120362	02/11/2012	-2	78.93	10.622
ct92-M305Pete-12	120362	02/11/2012	-2	78.929	10.637
ct92-M305Pete-12	120362	02/11/2012	-2	78.933	10.693
ct92-M305Pete-12	120362	02/11/2012	-2	78.923	10.705
ct92-M305Pete-12	120362	02/11/2012	-2	78.924	10.711
ct92-M305Pete-12	120362	02/11/2012	-2	78.924	10.689
ct92-M305Pete-12	120362	02/11/2012	-1	78.919	10.731
ct92-M305Pete-12	120362	02/11/2012	-1	78.93	10.704
ct92-M305Pete-12	120362	02/11/2012	-2	78.912	10.824
ct92-M305Pete-12	120362	02/11/2012	-2	78.908	10.817
ct92-M305Pete-12	120362	02/11/2012	-1	78.907	10.835
ct92-M305Pete-12	120362	02/11/2012	-2	78.907	10.832
ct92-M305Pete-12	120362	02/11/2012	-2	78.911	10.786
ct92-M305Pete-12	120362	02/11/2012	-2	78.912	10.799
ct92-M305Pete-12	120362	02/11/2012	0	78.898	10.755
ct92-M305Pete-12	120362	02/11/2012	-2	78.932	10.783
ct92-M305Pete-12	120362	02/11/2012	0	78.897	10.853
ct92-M305Pete-12	120362	02/11/2012	-2	78.896	10.897
ct92-M305Pete-12	120362	02/11/2012	-2	78.901	10.83
ct92-M305Pete-12	120362	02/11/2012	-1	78.897	10.876
ct92-M305Pete-12	120362	02/11/2012	-2	78.902	10.813
ct92-M305Pete-12	120362	02/11/2012	-1	78.921	10.787
ct92-M305Pete-12	120362	02/11/2012	-2	78.903	10.814
ct92-M305Pete-12	120362	02/11/2012	-2	78.919	10.809
ct92-M305Pete-12	120362	02/11/2012	-2	78.917	10.764
ct92-M305Pete-12	120362	02/11/2012	-2	78.919	10.807
ct92-M305Pete-12	120362	02/11/2012	-2	78.921	10.78
ct92-M305Pete-12	120362	02/11/2012	-2	78.917	10.774
ct92-M305Pete-12	120362	02/11/2012	-2	78.915	10.812
ct92-M305Pete-12	120362	02/11/2012	-2	78.914	10.808
ct92-M305Pete-12	120362	02/11/2012	-2	78.933	10.747
ct92-M305Pete-12	120362	02/11/2012	-2	78.928	10.753
ct92-M305Pete-12	120362	02/11/2012	-2	78.927	10.802
ct92-M305Pete-12	120362	02/11/2012	-1	78.922	10.723
ct92-M305Pete-12	120362	02/11/2012	-1	78.927	10.787
ct92-M305Pete-12	120362	02/11/2012	-2	78.917	10.753
ct92-M305Pete-12	120362	02/11/2012	-1	78.929	10.763
ct92-M305Pete-12	120362	02/11/2012	-2	78.928	10.785
ct92-M305Pete-12	120362	02/11/2012	-1	78.919	10.756
ct92-M305Pete-12	120362	02/11/2012	-1	78.946	10.616
ct92-M305Pete-12	120362	02/11/2012	-1	78.919	10.702
ct92-M305Pete-12	120362	02/11/2012	-2	78.912	10.719
ct92-M305Pete-12	120362	02/11/2012	0	78.922	10.667
ct92-M305Pete-12	120362	02/11/2012	1	78.932	10.699
ct92-M305Pete-12	120362	02/11/2012	-2	78.931	10.678
ct92-M305Pete-12	120362	02/11/2012	-2	78.937	10.703
ct92-M305Pete-12	120362	02/11/2012	-2	78.936	10.674
ct92-M305Pete-12	120362	02/11/2012	-2	78.934	10.624
ct92-M305Pete-12	120362	02/11/2012	-1	78.942	10.615
ct92-M305Pete-12	120362	02/11/2012	1	78.939	10.652
ct92-M305Pete-12	120362	02/11/2012	-2	78.928	10.543
ct92-M305Pete-12	120362	02/11/2012	-2	78.927	10.57
ct92-M305Pete-12	120362	02/11/2012	-2	78.934	10.574
ct92-M305Pete-12	120362	02/11/2012	-1	78.928	10.57
ct92-M305Pete-12	120362	02/11/2012	-2	78.94	10.576
ct92-M305Pete-12	120362	02/11/2012	-2	78.934	10.575
ct92-M305Pete-12	120362	02/11/2012	-1	78.95	10.592
ct92-M305Pete-12	120362	02/11/2012	-2	78.956	10.605
ct92-M305Pete-12	120362	02/11/2012	-2	78.955	10.596
ct92-M305Pete-12	120362	02/11/2012	-2	78.95	10.596
ct92-M305Pete-12	120362	02/11/2012	-2	78.921	10.53
ct92-M305Pete-12	120362	02/11/2012	-2	78.922	10.536
ct92-M305Pete-12	120362	02/11/2012	-2	78.922	10.536
ct92-M305Pete-12	120362	02/11/2012	-2	78.934	10.505

ct92-M305Pete-12	120362	02/11/2012	-2	78.934	10.505
ct92-M305Pete-12	120362	02/11/2012	-1	78.931	10.596
ct92-M305Pete-12	120362	02/11/2012	-2	78.937	10.591
ct92-M305Pete-12	120362	02/11/2012	-2	78.936	10.596
ct92-M305Pete-12	120362	02/11/2012	-2	78.925	10.549
ct92-M305Pete-12	120362	02/11/2012	-2	78.918	10.635
ct92-M305Pete-12	120362	02/11/2012	1	78.918	10.637
ct92-M305Pete-12	120362	02/11/2012	-2	78.918	10.595
ct92-M305Pete-12	120362	02/11/2012	-2	78.919	10.598
ct92-M305Pete-12	120362	02/11/2012	-2	78.92	10.594
ct92-M305Pete-12	120362	02/11/2012	-2	78.924	10.591
ct92-M305Pete-12	120362	02/11/2012	-2	78.923	10.611
ct92-M305Pete-12	120362	02/11/2012	-2	78.914	10.633
ct92-M305Pete-12	120362	02/11/2012	-2	78.911	10.642
ct92-M305Pete-12	120362	02/11/2012	-2	78.936	10.58
ct92-M305Pete-12	120362	03/11/2012	-2	78.916	10.66
ct92-M305Pete-12	120362	03/11/2012	-2	78.913	10.656
ct92-M305Pete-12	120362	03/11/2012	-2	78.915	10.662
ct92-M305Pete-12	120362	03/11/2012	-2	78.914	10.662
ct92-M305Pete-12	120362	03/11/2012	-2	78.917	10.664
ct92-M305Pete-12	120362	03/11/2012	-2	78.921	10.676
ct92-M305Pete-12	120362	03/11/2012	-2	78.895	10.634
ct92-M305Pete-12	120362	03/11/2012	-2	78.906	10.717
ct92-M305Pete-12	120362	03/11/2012	-2	78.902	10.689
ct92-M305Pete-12	120362	03/11/2012	-2	78.908	10.565
ct92-M305Pete-12	120362	03/11/2012	0	78.926	10.558
ct92-M305Pete-12	120362	03/11/2012	-2	78.922	10.628
ct92-M305Pete-12	120362	03/11/2012	-2	78.92	10.634
ct92-M305Pete-12	120362	03/11/2012	-2	78.933	10.581
ct92-M305Pete-12	120362	03/11/2012	-1	78.921	10.552
ct92-M305Pete-12	120362	03/11/2012	-2	78.923	10.553
ct92-M305Pete-12	120362	03/11/2012	-2	78.93	10.569
ct92-M305Pete-12	120362	03/11/2012	-2	78.917	10.618
ct92-M305Pete-12	120362	03/11/2012	-1	78.936	10.564
ct92-M305Pete-12	120362	03/11/2012	-2	78.932	10.589
ct92-M305Pete-12	120362	03/11/2012	-2	78.931	10.531
ct92-M305Pete-12	120362	03/11/2012	-1	78.935	10.589
ct92-M305Pete-12	120362	03/11/2012	-2	78.927	10.479
ct92-M305Pete-12	120362	03/11/2012	-2	78.928	10.488
ct92-M305Pete-12	120362	03/11/2012	-2	78.916	10.513
ct92-M305Pete-12	120362	03/11/2012	-2	78.917	10.551
ct92-M305Pete-12	120362	03/11/2012	1	78.922	10.539
ct92-M305Pete-12	120362	03/11/2012	-1	78.927	10.534
ct92-M305Pete-12	120362	03/11/2012	1	78.927	10.542
ct92-M305Pete-12	120362	03/11/2012	-2	78.92	10.546
ct92-M305Pete-12	120362	03/11/2012	-2	78.922	10.572
ct92-M305Pete-12	120362	03/11/2012	-1	78.92	10.571
ct92-M305Pete-12	120362	03/11/2012	-2	78.916	10.628
ct92-M305Pete-12	120362	03/11/2012	-2	78.934	10.659
ct92-M305Pete-12	120362	03/11/2012	-2	78.933	10.621
ct92-M305Pete-12	120362	03/11/2012	-2	78.916	10.632
ct92-M305Pete-12	120362	03/11/2012	-2	78.918	10.663
ct92-M305Pete-12	120362	03/11/2012	-2	78.916	10.674
ct92-M305Pete-12	120362	03/11/2012	-2	78.916	10.673
ct92-M305Pete-12	120362	03/11/2012	-2	78.885	10.737
ct92-M305Pete-12	120362	03/11/2012	0	78.914	10.697
ct92-M305Pete-12	120362	03/11/2012	-2	78.881	10.821
ct92-M305Pete-12	120362	03/11/2012	-2	78.889	10.764
ct92-M305Pete-12	120362	03/11/2012	-1	78.884	10.71
ct92-M305Pete-12	120362	03/11/2012	-2	78.886	10.684
ct92-M305Pete-12	120362	03/11/2012	-2	78.889	10.665
ct92-M305Pete-12	120362	03/11/2012	-2	78.892	10.672
ct92-M305Pete-12	120362	03/11/2012	-2	78.856	10.629
ct92-M305Pete-12	120362	03/11/2012	-2	78.888	10.701
ct92-M305Pete-12	120362	03/11/2012	-2	78.896	10.691
ct92-M305Pete-12	120362	03/11/2012	-2	78.898	10.707
ct92-M305Pete-12	120362	03/11/2012	-2	78.895	10.708
ct92-M305Pete-12	120362	03/11/2012	-2	78.897	10.791

ct92-M305Pete-12	120362	03/11/2012	-2	78.907	10.774
ct92-M305Pete-12	120362	03/11/2012	-2	78.928	10.802
ct92-M305Pete-12	120362	03/11/2012	-2	78.914	10.768
ct92-M305Pete-12	120362	03/11/2012	-1	78.932	10.871
ct92-M305Pete-12	120362	03/11/2012	-2	78.926	10.815
ct92-M305Pete-12	120362	03/11/2012	-2	78.928	10.889
ct92-M305Pete-12	120362	03/11/2012	-2	78.931	10.795
ct92-M305Pete-12	120362	03/11/2012	-2	78.931	10.801
ct92-M305Pete-12	120362	03/11/2012	-2	78.93	10.79
ct92-M305Pete-12	120362	03/11/2012	-1	78.926	10.843
ct92-M305Pete-12	120362	03/11/2012	-2	78.927	10.821
ct92-M305Pete-12	120362	03/11/2012	-2	78.928	10.823
ct92-M305Pete-12	120362	03/11/2012	-2	78.924	10.845
ct92-M305Pete-12	120362	03/11/2012	-2	78.924	10.854
ct92-M305Pete-12	120362	03/11/2012	-2	78.924	10.842
ct92-M305Pete-12	120362	03/11/2012	-2	78.924	10.887
ct92-M305Pete-12	120362	03/11/2012	-1	78.911	10.723
ct92-M305Pete-12	120362	03/11/2012	-2	78.901	10.744
ct92-M305Pete-12	120362	03/11/2012	-2	78.909	10.705
ct92-M305Pete-12	120362	04/11/2012	-1	78.925	10.761
ct92-M305Pete-12	120362	04/11/2012	-2	78.922	10.768
ct92-M305Pete-12	120362	04/11/2012	-2	78.918	10.766
ct92-M305Pete-12	120362	04/11/2012	-1	78.915	10.772
ct92-M305Pete-12	120362	04/11/2012	-2	78.896	10.815
ct92-M305Pete-12	120362	04/11/2012	-2	78.895	10.822
ct92-M305Pete-12	120362	04/11/2012	-2	78.888	10.824
ct92-M305Pete-12	120362	04/11/2012	1	78.887	10.833
ct92-M305Pete-12	120362	04/11/2012	-2	78.879	10.849
ct92-M305Pete-12	120362	04/11/2012	-2	78.901	10.847
ct92-M305Pete-12	120362	04/11/2012	-1	78.885	10.871
ct92-M305Pete-12	120362	04/11/2012	-2	78.899	10.857
ct92-M305Pete-12	120362	04/11/2012	-1	78.907	10.827
ct92-M305Pete-12	120362	04/11/2012	2	78.904	10.827
ct92-M305Pete-12	120362	04/11/2012	-2	78.906	10.826
ct92-M305Pete-12	120362	04/11/2012	-2	78.9	10.823
ct92-M305Pete-12	120362	04/11/2012	-1	78.903	10.842
ct92-M305Pete-12	120362	04/11/2012	-1	78.907	10.802
ct92-M305Pete-12	120362	04/11/2012	-2	78.907	10.812
ct92-M305Pete-12	120362	04/11/2012	-2	78.909	10.832
ct92-M305Pete-12	120362	04/11/2012	-2	78.909	10.811
ct92-M305Pete-12	120362	04/11/2012	-2	78.916	10.77
ct92-M305Pete-12	120362	04/11/2012	0	78.912	10.784
ct92-M305Pete-12	120362	04/11/2012	-1	78.923	10.765
ct92-M305Pete-12	120362	04/11/2012	1	78.91	10.765
ct92-M305Pete-12	120362	04/11/2012	-2	78.91	10.752
ct92-M305Pete-12	120362	04/11/2012	-2	78.909	10.759
ct92-M305Pete-12	120362	04/11/2012	-2	78.917	10.741
ct92-M305Pete-12	120362	04/11/2012	-1	78.913	10.726
ct92-M305Pete-12	120362	04/11/2012	-1	78.912	10.746
ct92-M305Pete-12	120362	04/11/2012	1	78.916	10.75
ct92-M305Pete-12	120362	04/11/2012	-2	78.912	10.746
ct92-M305Pete-12	120362	04/11/2012	-2	78.893	10.684
ct92-M305Pete-12	120362	04/11/2012	1	78.919	10.737
ct92-M305Pete-12	120362	04/11/2012	-2	78.903	10.622
ct92-M305Pete-12	120362	04/11/2012	1	78.928	10.745
ct92-M305Pete-12	120362	04/11/2012	0	78.921	10.768
ct92-M305Pete-12	120362	04/11/2012	-1	78.926	10.735
ct92-M305Pete-12	120362	04/11/2012	1	78.924	10.74
ct92-M305Pete-12	120362	04/11/2012	1	78.913	10.753
ct92-M305Pete-12	120362	04/11/2012	-1	78.916	10.771
ct92-M305Pete-12	120362	04/11/2012	-2	78.917	10.772
ct92-M305Pete-12	120362	04/11/2012	-2	78.919	10.741
ct92-M305Pete-12	120362	04/11/2012	1	78.917	10.756
ct92-M305Pete-12	120362	04/11/2012	-2	78.915	10.77
ct92-M305Pete-12	120362	04/11/2012	-2	78.917	10.76
ct92-M305Pete-12	120362	04/11/2012	-2	78.933	10.735
ct92-M305Pete-12	120362	04/11/2012	-2	78.932	10.715
ct92-M305Pete-12	120362	04/11/2012	-2	78.932	10.732

ct92-M305Pete-12	120362	04/11/2012	-1	78.926	10.722
ct92-M305Pete-12	120362	04/11/2012	-1	78.933	10.732
ct92-M305Pete-12	120362	04/11/2012	2	78.915	10.666
ct92-M305Pete-12	120362	04/11/2012	-2	78.942	10.711
ct92-M305Pete-12	120362	04/11/2012	-2	78.914	10.674
ct92-M305Pete-12	120362	04/11/2012	-2	78.972	10.793
ct92-M305Pete-12	120362	04/11/2012	-2	78.962	10.747
ct92-M305Pete-12	120362	04/11/2012	-2	78.961	10.755
ct92-M305Pete-12	120362	04/11/2012	-2	78.953	10.73
ct92-M305Pete-12	120362	04/11/2012	-2	78.924	10.705
ct92-M305Pete-12	120362	04/11/2012	-2	78.916	10.695
ct92-M305Pete-12	120362	04/11/2012	-2	78.899	10.619
ct92-M305Pete-12	120362	04/11/2012	-2	78.899	10.62
ct92-M305Pete-12	120362	04/11/2012	-2	78.899	10.608
ct92-M305Pete-12	120362	04/11/2012	-2	78.903	10.649
ct92-M305Pete-12	120362	04/11/2012	-2	78.913	10.622
ct92-M305Pete-12	120362	04/11/2012	-2	78.909	10.614
ct92-M305Pete-12	120362	04/11/2012	-2	78.902	10.64
ct92-M305Pete-12	120362	05/11/2012	-2	78.896	10.636
ct92-M305Pete-12	120362	05/11/2012	-2	78.922	10.49
ct92-M305Pete-12	120362	05/11/2012	-2	78.93	10.467
ct92-M305Pete-12	120362	05/11/2012	-2	78.931	10.466
ct92-M305Pete-12	120362	05/11/2012	-2	78.929	10.502
ct92-M305Pete-12	120362	05/11/2012	-2	78.929	10.5
ct92-M305Pete-12	120362	05/11/2012	-2	78.914	10.476
ct92-M305Pete-12	120362	05/11/2012	-2	78.921	10.534
ct92-M305Pete-12	120362	05/11/2012	-2	78.913	10.474
ct92-M305Pete-12	120362	05/11/2012	-2	78.893	10.573
ct92-M305Pete-12	120362	05/11/2012	-2	78.918	10.592
ct92-M305Pete-12	120362	05/11/2012	-2	78.92	10.622
ct92-M305Pete-12	120362	05/11/2012	-2	78.922	10.684
ct92-M305Pete-12	120362	05/11/2012	-2	78.938	10.669
ct92-M305Pete-12	120362	05/11/2012	-2	78.938	10.709
ct92-M305Pete-12	120362	05/11/2012	-2	78.934	10.713
ct92-M305Pete-12	120362	05/11/2012	-2	78.921	10.684
ct92-M305Pete-12	120362	05/11/2012	-2	78.923	10.678
ct92-M305Pete-12	120362	05/11/2012	-2	78.923	10.685
ct92-M305Pete-12	120362	05/11/2012	-2	78.917	10.706
ct92-M305Pete-12	120362	05/11/2012	-2	78.922	10.704
ct92-M305Pete-12	120362	05/11/2012	-2	78.922	10.71
ct92-M305Pete-12	120362	05/11/2012	-2	78.936	10.784
ct92-M305Pete-12	120362	05/11/2012	-2	78.935	10.786
ct92-M305Pete-12	120362	05/11/2012	-2	78.92	10.774
ct92-M305Pete-12	120362	05/11/2012	-2	78.917	10.834
ct92-M305Pete-12	120362	05/11/2012	-2	78.914	10.827
ct92-M305Pete-12	120362	05/11/2012	-1	78.92	10.822
ct92-M305Pete-12	120362	05/11/2012	-2	78.93	10.788
ct92-M305Pete-12	120362	05/11/2012	-2	78.924	10.836
ct92-M305Pete-12	120362	05/11/2012	-2	78.92	10.837
ct92-M305Pete-12	120362	05/11/2012	-2	78.913	10.753
ct92-M305Pete-12	120362	05/11/2012	-2	78.913	10.754
ct92-M305Pete-12	120362	05/11/2012	-2	78.915	10.752
ct92-M305Pete-12	120362	05/11/2012	-2	78.927	10.824
ct92-M305Pete-12	120362	05/11/2012	-2	78.929	10.815
ct92-M305Pete-12	120362	05/11/2012	-2	78.912	10.77
ct92-M305Pete-12	120362	05/11/2012	-2	78.913	10.763
ct92-M305Pete-12	120362	05/11/2012	-2	78.913	10.765
ct92-M305Pete-12	120362	05/11/2012	-1	78.902	10.724
ct92-M305Pete-12	120362	05/11/2012	-2	78.912	10.764
ct92-M305Pete-12	120362	05/11/2012	-2	78.913	10.776
ct92-M305Pete-12	120362	05/11/2012	0	78.906	10.645
ct92-M305Pete-12	120362	05/11/2012	-2	78.921	10.803
ct92-M305Pete-12	120362	05/11/2012	-2	78.924	10.782
ct92-M305Pete-12	120362	05/11/2012	1	78.917	10.772
ct92-M305Pete-12	120362	05/11/2012	-2	78.923	10.778
ct92-M305Pete-12	120362	05/11/2012	-2	78.922	10.778
ct92-M305Pete-12	120362	05/11/2012	-2	78.921	10.78
ct92-M305Pete-12	120362	05/11/2012	-2	78.947	10.819

ct92-M305Pete-12	120362	05/11/2012	0	78.925	10.799
ct92-M305Pete-12	120362	05/11/2012	-2	78.938	10.828
ct92-M305Pete-12	120362	05/11/2012	-2	78.918	10.788
ct92-M305Pete-12	120362	05/11/2012	-2	78.903	10.751
ct92-M305Pete-12	120362	05/11/2012	-1	78.896	10.726
ct92-M305Pete-12	120362	05/11/2012	2	78.92	10.624
ct92-M305Pete-12	120362	05/11/2012	-1	78.902	10.696
ct92-M305Pete-12	120362	05/11/2012	-2	78.917	10.793
ct92-M305Pete-12	120362	05/11/2012	-2	78.924	10.806
ct92-M305Pete-12	120362	05/11/2012	-2	78.91	10.77
ct92-M305Pete-12	120362	05/11/2012	-2	78.9	10.73
ct92-M305Pete-12	120362	05/11/2012	-2	78.902	10.717
ct92-M305Pete-12	120362	05/11/2012	-2	78.897	10.752
ct92-M305Pete-12	120362	05/11/2012	-2	78.906	10.75
ct92-M305Pete-12	120362	05/11/2012	-2	78.897	10.755
ct92-M305Pete-12	120362	05/11/2012	-2	78.898	10.762
ct92-M305Pete-12	120362	05/11/2012	-2	78.881	10.718
ct92-M305Pete-12	120362	05/11/2012	-2	78.883	10.695
ct92-M305Pete-12	120362	05/11/2012	-2	78.906	10.736
ct92-M305Pete-12	120362	05/11/2012	-1	78.908	10.738
ct92-M305Pete-12	120362	05/11/2012	-2	78.905	10.734
ct92-M305Pete-12	120362	05/11/2012	-2	78.906	10.738
ct92-M305Pete-12	120362	05/11/2012	-2	78.917	10.644
ct92-M305Pete-12	120362	05/11/2012	-2	78.908	10.84
ct92-M305Pete-12	120362	05/11/2012	-2	78.91	10.83
ct92-M305Pete-12	120362	05/11/2012	2	78.926	10.743
ct92-M305Pete-12	120362	05/11/2012	-1	78.923	10.726
ct92-M305Pete-12	120362	05/11/2012	-2	78.905	10.802
ct92-M305Pete-12	120362	06/11/2012	-2	78.909	10.696
ct92-M305Pete-12	120362	06/11/2012	-1	78.927	10.907
ct92-M305Pete-12	120362	06/11/2012	-2	78.931	10.865
ct92-M305Pete-12	120362	06/11/2012	-1	78.933	10.7
ct92-M305Pete-12	120362	06/11/2012	-2	78.928	10.676
ct92-M305Pete-12	120362	06/11/2012	-2	78.914	10.75
ct92-M305Pete-12	120362	06/11/2012	-2	78.914	10.752
ct92-M305Pete-12	120362	06/11/2012	-1	78.912	10.68
ct92-M305Pete-12	120362	06/11/2012	-2	78.913	10.692
ct92-M305Pete-12	120362	06/11/2012	-2	78.906	10.647
ct92-M305Pete-12	120362	06/11/2012	-2	78.904	10.646
ct92-M305Pete-12	120362	06/11/2012	-2	78.906	10.647
ct92-M305Pete-12	120362	06/11/2012	-2	78.904	10.642
ct92-M305Pete-12	120362	06/11/2012	-2	78.898	10.593
ct92-M305Pete-12	120362	06/11/2012	-2	78.898	10.582
ct92-M305Pete-12	120362	06/11/2012	-2	78.897	10.606
ct92-M305Pete-12	120362	06/11/2012	-2	78.889	10.554
ct92-M305Pete-12	120362	06/11/2012	-2	78.935	10.439
ct92-M305Pete-12	120362	06/11/2012	-2	78.935	10.554
ct92-M305Pete-12	120362	06/11/2012	-2	78.94	10.546
ct92-M305Pete-12	120362	06/11/2012	0	78.931	10.567
ct92-M305Pete-12	120362	06/11/2012	-2	78.923	10.518
ct92-M305Pete-12	120362	06/11/2012	0	78.918	10.545
ct92-M305Pete-12	120362	06/11/2012	-2	78.906	10.563
ct92-M305Pete-12	120362	06/11/2012	-2	78.903	10.549
ct92-M305Pete-12	120362	06/11/2012	-2	78.897	10.513
ct92-M305Pete-12	120362	06/11/2012	-2	78.901	10.552
ct92-M305Pete-12	120362	06/11/2012	-2	78.898	10.589
ct92-M305Pete-12	120362	06/11/2012	-2	78.923	10.582
ct92-M305Pete-12	120362	06/11/2012	-2	78.923	10.604
ct92-M305Pete-12	120362	06/11/2012	-2	78.914	10.566
ct92-M305Pete-12	120362	06/11/2012	-2	78.918	10.691
ct92-M305Pete-12	120362	06/11/2012	-2	78.91	10.586
ct92-M305Pete-12	120362	06/11/2012	-2	78.91	10.601
ct92-M305Pete-12	120362	06/11/2012	-2	78.925	10.646
ct92-M305Pete-12	120362	06/11/2012	-2	78.923	10.7
ct92-M305Pete-12	120362	06/11/2012	1	78.924	10.688
ct92-M305Pete-12	120362	06/11/2012	-2	78.916	10.713
ct92-M305Pete-12	120362	06/11/2012	-2	78.909	10.751
ct92-M305Pete-12	120362	06/11/2012	-2	78.918	10.757

ct92-M305Pete-12	120362	06/11/2012	-2	78.916	10.752
ct92-M305Pete-12	120362	06/11/2012	-2	78.924	10.729
ct92-M305Pete-12	120362	06/11/2012	-2	78.901	10.706
ct92-M305Pete-12	120362	06/11/2012	-1	78.913	10.671
ct92-M305Pete-12	120362	06/11/2012	-2	78.908	10.67
ct92-M305Pete-12	120362	06/11/2012	-2	78.914	10.698
ct92-M305Pete-12	120362	06/11/2012	0	78.885	10.702
ct92-M305Pete-12	120362	06/11/2012	-2	78.888	10.853
ct92-M305Pete-12	120362	06/11/2012	1	78.895	10.888
ct92-M305Pete-12	120362	06/11/2012	-1	78.893	10.872
ct92-M305Pete-12	120362	06/11/2012	-2	78.893	10.869
ct92-M305Pete-12	120362	06/11/2012	-1	78.891	10.848
ct92-M305Pete-12	120362	06/11/2012	-2	78.892	10.842
ct92-M305Pete-12	120362	06/11/2012	-2	78.908	10.869
ct92-M305Pete-12	120362	06/11/2012	-2	78.907	10.879
ct92-M305Pete-12	120362	06/11/2012	-2	78.908	10.863
ct92-M305Pete-12	120362	06/11/2012	-2	78.896	10.854
ct92-M305Pete-12	120362	06/11/2012	0	78.897	10.847
ct92-M305Pete-12	120362	06/11/2012	2	78.901	10.893
ct92-M305Pete-12	120362	06/11/2012	-2	78.899	10.894
ct92-M305Pete-12	120362	06/11/2012	-2	78.894	10.876
ct92-M305Pete-12	120362	06/11/2012	0	78.91	10.828
ct92-M305Pete-12	120362	06/11/2012	-2	78.911	10.828
ct92-M305Pete-12	120362	06/11/2012	-2	78.908	10.82
ct92-M305Pete-12	120362	06/11/2012	-1	78.897	10.807
ct92-M305Pete-12	120362	06/11/2012	-1	78.902	10.779
ct92-M305Pete-12	120362	06/11/2012	-2	78.903	10.753
ct92-M305Pete-12	120362	06/11/2012	-2	78.901	10.773
ct92-M305Pete-12	120362	06/11/2012	-2	78.902	10.756
ct92-M305Pete-12	120362	06/11/2012	1	78.891	10.689
ct92-M305Pete-12	120362	06/11/2012	-2	78.904	10.753
ct92-M305Pete-12	120362	06/11/2012	-2	78.897	10.716
ct92-M305Pete-12	120362	06/11/2012	-2	78.899	10.74
ct92-M305Pete-12	120362	06/11/2012	-2	78.896	10.643
ct92-M305Pete-12	120362	06/11/2012	-2	78.888	10.672
ct92-M305Pete-12	120362	06/11/2012	-2	78.883	10.64
ct92-M305Pete-12	120362	06/11/2012	-2	78.894	10.657
ct92-M305Pete-12	120362	06/11/2012	-1	78.901	10.635
ct92-M305Pete-12	120362	06/11/2012	-2	78.909	10.658
ct92-M305Pete-12	120362	06/11/2012	-2	78.909	10.656
ct92-M305Pete-12	120362	07/11/2012	-2	78.907	10.656
ct92-M305Pete-12	120362	07/11/2012	-2	78.905	10.66
ct92-M305Pete-12	120362	07/11/2012	-2	78.907	10.669
ct92-M305Pete-12	120362	07/11/2012	-2	78.907	10.668
ct92-M305Pete-12	120362	07/11/2012	-2	78.917	10.665
ct92-M305Pete-12	120362	07/11/2012	1	78.905	10.665
ct92-M305Pete-12	120362	07/11/2012	-2	78.904	10.665
ct92-M305Pete-12	120362	07/11/2012	-2	78.897	10.652
ct92-M305Pete-12	120362	07/11/2012	2	78.889	10.694
ct92-M305Pete-12	120362	07/11/2012	-2	78.885	10.693
ct92-M305Pete-12	120362	07/11/2012	-2	78.886	10.697
ct92-M305Pete-12	120362	07/11/2012	-2	78.884	10.709
ct92-M305Pete-12	120362	07/11/2012	-2	78.889	10.704
ct92-M305Pete-12	120362	07/11/2012	-2	78.884	10.713
ct92-M305Pete-12	120362	07/11/2012	-2	78.881	10.72
ct92-M305Pete-12	120362	07/11/2012	-2	78.883	10.713
ct92-M305Pete-12	120362	07/11/2012	-1	78.895	10.679
ct92-M305Pete-12	120362	07/11/2012	-1	78.898	10.554
ct92-M305Pete-12	120362	07/11/2012	-2	78.908	10.631
ct92-M305Pete-12	120362	07/11/2012	-2	78.908	10.624
ct92-M305Pete-12	120362	07/11/2012	-1	78.897	10.653
ct92-M305Pete-12	120362	07/11/2012	-2	78.895	10.616
ct92-M305Pete-12	120362	07/11/2012	-2	78.895	10.617
ct92-M305Pete-12	120362	07/11/2012	-2	78.9	10.614
ct92-M305Pete-12	120362	07/11/2012	-2	78.905	10.581
ct92-M305Pete-12	120362	07/11/2012	-2	78.905	10.582
ct92-M305Pete-12	120362	07/11/2012	-2	78.905	10.585
ct92-M305Pete-12	120362	07/11/2012	-2	78.902	10.606

ct92-M305Pete-12	120362	07/11/2012	-2	78.902	10.608
ct92-M305Pete-12	120362	07/11/2012	-2	78.896	10.574
ct92-M305Pete-12	120362	07/11/2012	-1	78.898	10.572
ct92-M305Pete-12	120362	07/11/2012	-2	78.897	10.561
ct92-M305Pete-12	120362	07/11/2012	-2	78.898	10.578
ct92-M305Pete-12	120362	07/11/2012	-1	78.901	10.598
ct92-M305Pete-12	120362	07/11/2012	-2	78.899	10.588
ct92-M305Pete-12	120362	07/11/2012	-2	78.912	10.597
ct92-M305Pete-12	120362	07/11/2012	-2	78.911	10.592
ct92-M305Pete-12	120362	07/11/2012	-2	78.895	10.576
ct92-M305Pete-12	120362	07/11/2012	-2	78.91	10.59
ct92-M305Pete-12	120362	07/11/2012	-2	78.911	10.575
ct92-M305Pete-12	120362	07/11/2012	-2	78.912	10.579
ct92-M305Pete-12	120362	07/11/2012	-2	78.912	10.579
ct92-M305Pete-12	120362	07/11/2012	-1	78.909	10.603
ct92-M305Pete-12	120362	07/11/2012	-2	78.893	10.457
ct92-M305Pete-12	120362	07/11/2012	-2	78.909	10.62
ct92-M305Pete-12	120362	07/11/2012	-2	78.912	10.621
ct92-M305Pete-12	120362	07/11/2012	-2	78.905	10.649
ct92-M305Pete-12	120362	07/11/2012	-2	78.912	10.61
ct92-M305Pete-12	120362	07/11/2012	-2	78.905	10.652
ct92-M305Pete-12	120362	07/11/2012	-2	78.913	10.719
ct92-M305Pete-12	120362	07/11/2012	-2	78.897	10.498
ct92-M305Pete-12	120362	07/11/2012	-2	78.91	10.719
ct92-M305Pete-12	120362	07/11/2012	-2	78.925	10.68
ct92-M305Pete-12	120362	07/11/2012	-2	78.913	10.639
ct92-M305Pete-12	120362	07/11/2012	-2	78.916	10.724
ct92-M305Pete-12	120362	07/11/2012	-2	78.902	10.685
ct92-M305Pete-12	120362	07/11/2012	-2	78.924	10.668
ct92-M305Pete-12	120362	07/11/2012	-2	78.924	10.667
ct92-M305Pete-12	120362	07/11/2012	-2	78.923	10.662
ct92-M305Pete-12	120362	07/11/2012	-2	78.923	10.66
ct92-M305Pete-12	120362	07/11/2012	-2	78.925	10.659
ct92-M305Pete-12	120362	07/11/2012	-2	78.936	10.608
ct92-M305Pete-12	120362	07/11/2012	-2	78.922	10.598
ct92-M305Pete-12	120362	07/11/2012	-1	78.919	10.661
ct92-M305Pete-12	120362	07/11/2012	-1	78.919	10.656
ct92-M305Pete-12	120362	07/11/2012	-1	78.911	10.608
ct92-M305Pete-12	120362	07/11/2012	-2	78.913	10.614
ct92-M305Pete-12	120362	07/11/2012	-2	78.904	10.628
ct92-M305Pete-12	120362	07/11/2012	-2	78.904	10.634
ct92-M305Pete-12	120362	07/11/2012	-2	78.913	10.636
ct92-M305Pete-12	120362	07/11/2012	-2	78.922	10.66
ct92-M305Pete-12	120362	07/11/2012	-2	78.923	10.679
ct92-M305Pete-12	120362	07/11/2012	-2	78.924	10.678
ct92-M305Pete-12	120362	07/11/2012	-2	78.925	10.691
ct92-M305Pete-12	120362	07/11/2012	-2	78.941	10.776
ct92-M305Pete-12	120362	07/11/2012	-2	78.935	10.726
ct92-M305Pete-12	120362	07/11/2012	-2	78.934	10.756
ct92-M305Pete-12	120362	07/11/2012	-2	78.938	10.733
ct92-M305Pete-12	120362	08/11/2012	-2	78.937	10.764
ct92-M305Pete-12	120362	08/11/2012	-2	78.936	10.709
ct92-M305Pete-12	120362	08/11/2012	-2	78.935	10.767
ct92-M305Pete-12	120362	08/11/2012	-2	78.927	10.767
ct92-M305Pete-12	120362	08/11/2012	-2	78.925	10.743
ct92-M305Pete-12	120362	08/11/2012	-2	78.925	10.743
ct92-M305Pete-12	120362	08/11/2012	-2	78.935	10.777
ct92-M305Pete-12	120362	08/11/2012	-2	78.919	10.723
ct92-M305Pete-12	120362	08/11/2012	-2	78.931	10.7
ct92-M305Pete-12	120362	08/11/2012	-2	78.911	10.672
ct92-M305Pete-12	120362	08/11/2012	-2	78.91	10.67
ct92-M305Pete-12	120362	08/11/2012	-2	78.913	10.592
ct92-M305Pete-12	120362	08/11/2012	-2	78.922	10.57
ct92-M305Pete-12	120362	08/11/2012	-2	78.922	10.525
ct92-M305Pete-12	120362	08/11/2012	-2	78.906	10.611
ct92-M305Pete-12	120362	08/11/2012	-2	78.905	10.593
ct92-M305Pete-12	120362	08/11/2012	-2	78.903	10.591
ct92-M305Pete-12	120362	08/11/2012	-2	78.898	10.582

ct92-M305Pete-12	120362	08/11/2012	-2	78.903	10.584
ct92-M305Pete-12	120362	08/11/2012	0	78.932	10.589
ct92-M305Pete-12	120362	08/11/2012	2	78.912	10.583
ct92-M305Pete-12	120362	08/11/2012	-1	78.909	10.587
ct92-M305Pete-12	120362	08/11/2012	1	78.93	10.592
ct92-M305Pete-12	120362	08/11/2012	-2	78.919	10.573
ct92-M305Pete-12	120362	08/11/2012	-2	78.91	10.582
ct92-M305Pete-12	120362	08/11/2012	-1	78.912	10.533
ct92-M305Pete-12	120362	08/11/2012	-1	78.919	10.657
ct92-M305Pete-12	120362	08/11/2012	-1	78.908	10.639
ct92-M305Pete-12	120362	08/11/2012	-1	78.895	10.619
ct92-M305Pete-12	120362	08/11/2012	-2	78.92	10.604
ct92-M305Pete-12	120362	08/11/2012	-2	78.902	10.601
ct92-M305Pete-12	120362	08/11/2012	-2	78.902	10.593
ct92-M305Pete-12	120362	08/11/2012	-2	78.915	10.625
ct92-M305Pete-12	120362	08/11/2012	-2	78.905	10.598
ct92-M305Pete-12	120362	08/11/2012	-1	78.893	10.575
ct92-M305Pete-12	120362	08/11/2012	-1	78.892	10.529
ct92-M305Pete-12	120362	08/11/2012	-2	78.898	10.552
ct92-M305Pete-12	120362	08/11/2012	-1	78.904	10.583
ct92-M305Pete-12	120362	08/11/2012	2	78.905	10.588
ct92-M305Pete-12	120362	08/11/2012	-1	78.913	10.556
ct92-M305Pete-12	120362	08/11/2012	-1	78.923	10.607
ct92-M305Pete-12	120362	08/11/2012	-2	78.91	10.545
ct92-M305Pete-12	120362	08/11/2012	-2	78.897	10.589
ct92-M305Pete-12	120362	08/11/2012	-2	78.913	10.546
ct92-M305Pete-12	120362	08/11/2012	-2	78.915	10.537
ct92-M305Pete-12	120362	08/11/2012	-2	78.915	10.539
ct92-M305Pete-12	120362	08/11/2012	-1	78.9	10.447
ct92-M305Pete-12	120362	08/11/2012	-2	78.915	10.53
ct92-M305Pete-12	120362	08/11/2012	-1	78.897	10.551
ct92-M305Pete-12	120362	08/11/2012	-2	78.895	10.566
ct92-M305Pete-12	120362	08/11/2012	-2	78.894	10.568
ct92-M305Pete-12	120362	08/11/2012	0	78.888	10.573
ct92-M305Pete-12	120362	08/11/2012	-1	78.902	10.625
ct92-M305Pete-12	120362	08/11/2012	-1	78.91	10.589
ct92-M305Pete-12	120362	08/11/2012	-1	78.907	10.588
ct92-M305Pete-12	120362	08/11/2012	-2	78.893	10.675
ct92-M305Pete-12	120362	08/11/2012	-2	78.893	10.696
ct92-M305Pete-12	120362	08/11/2012	-2	78.896	10.687
ct92-M305Pete-12	120362	08/11/2012	-2	78.908	10.708
ct92-M305Pete-12	120362	08/11/2012	-1	78.909	10.68
ct92-M305Pete-12	120362	08/11/2012	-1	78.909	10.682
ct92-M305Pete-12	120362	08/11/2012	-1	78.91	10.68
ct92-M305Pete-12	120362	08/11/2012	1	78.908	10.631
ct92-M305Pete-12	120362	09/11/2012	-2	78.897	10.634
ct92-M305Pete-12	120362	09/11/2012	2	78.911	10.585
ct92-M305Pete-12	120362	09/11/2012	1	78.91	10.539
ct92-M305Pete-12	120362	09/11/2012	2	78.914	10.581
ct92-M305Pete-12	120362	09/11/2012	-2	78.929	10.505
ct92-M305Pete-12	120362	09/11/2012	-2	78.921	10.551
ct92-M305Pete-12	120362	09/11/2012	0	78.905	10.474
ct92-M305Pete-12	120362	09/11/2012	-2	78.902	10.689
ct92-M305Pete-12	120362	09/11/2012	-2	78.904	10.641
ct92-M305Pete-12	120362	09/11/2012	-2	78.907	10.575
ct92-M305Pete-12	120362	09/11/2012	-2	78.904	10.627
ct92-M305Pete-12	120362	09/11/2012	-2	78.906	10.654
ct92-M305Pete-12	120362	09/11/2012	-2	78.901	10.661
ct92-M305Pete-12	120362	09/11/2012	-2	78.9	10.688
ct92-M305Pete-12	120362	09/11/2012	-1	78.915	10.721
ct92-M305Pete-12	120362	09/11/2012	-2	78.924	10.727
ct92-M305Pete-12	120362	09/11/2012	-2	78.92	10.717
ct92-M305Pete-12	120362	09/11/2012	-1	78.931	10.673
ct92-M305Pete-12	120362	09/11/2012	-2	78.918	10.724
ct92-M305Pete-12	120362	09/11/2012	0	78.913	10.729
ct92-M305Pete-12	120362	09/11/2012	-2	78.926	10.8
ct92-M305Pete-12	120362	09/11/2012	-2	78.925	10.786
ct92-M305Pete-12	120362	09/11/2012	0	78.922	10.76

ct92-M305Pete-12	120362	09/11/2012	-2	78.919	10.767
ct92-M305Pete-12	120362	09/11/2012	1	78.924	10.78
ct92-M305Pete-12	120362	09/11/2012	1	78.918	10.701
ct92-M305Pete-12	120362	09/11/2012	-2	78.914	10.693
ct92-M305Pete-12	120362	09/11/2012	-2	78.914	10.687
ct92-M305Pete-12	120362	09/11/2012	-2	78.905	10.763
ct92-M305Pete-12	120362	09/11/2012	0	78.893	10.876
ct92-M305Pete-12	120362	09/11/2012	-2	78.911	10.768
ct92-M305Pete-12	120362	09/11/2012	-1	78.914	10.675
ct92-M305Pete-12	120362	09/11/2012	-2	78.915	10.687
ct92-M305Pete-12	120362	09/11/2012	-2	78.92	10.662
ct92-M305Pete-12	120362	09/11/2012	-2	78.923	10.672
ct92-M305Pete-12	120362	09/11/2012	-1	78.914	10.692
ct92-M305Pete-12	120362	09/11/2012	-2	78.919	10.646
ct92-M305Pete-12	120362	09/11/2012	1	78.905	10.572
ct92-M305Pete-12	120362	09/11/2012	-2	78.905	10.671
ct92-M305Pete-12	120362	09/11/2012	-2	78.908	10.657
ct92-M305Pete-12	120362	09/11/2012	-2	78.908	10.659
ct92-M305Pete-12	120362	09/11/2012	-1	78.902	10.729
ct92-M305Pete-12	120362	09/11/2012	-1	78.921	10.665
ct92-M305Pete-12	120362	09/11/2012	-2	78.926	10.642
ct92-M305Pete-12	120362	09/11/2012	-1	78.94	10.586
ct92-M305Pete-12	120362	09/11/2012	0	78.917	10.554
ct92-M305Pete-12	120362	09/11/2012	-2	78.91	10.561
ct92-M305Pete-12	120362	09/11/2012	-2	78.931	10.628
ct92-M305Pete-12	120362	09/11/2012	-2	78.923	10.493
ct92-M305Pete-12	120362	09/11/2012	-1	78.9	10.465
ct92-M305Pete-12	120362	09/11/2012	0	78.895	10.558
ct92-M305Pete-12	120362	09/11/2012	-1	78.901	10.622
ct92-M305Pete-12	120362	09/11/2012	-2	78.902	10.527
ct92-M305Pete-12	120362	09/11/2012	-2	78.902	10.536
ct92-M305Pete-12	120362	09/11/2012	-2	78.917	10.468
ct92-M305Pete-12	120362	09/11/2012	-2	78.901	10.537
ct92-M305Pete-12	120362	09/11/2012	-2	78.911	10.488
ct92-M305Pete-12	120362	09/11/2012	-1	78.917	10.58
ct92-M305Pete-12	120362	09/11/2012	-2	78.926	10.556
ct92-M305Pete-12	120362	09/11/2012	-2	78.895	10.528
ct92-M305Pete-12	120362	09/11/2012	-2	78.899	10.508
ct92-M305Pete-12	120362	09/11/2012	-2	78.901	10.504
ct92-M305Pete-12	120362	09/11/2012	-2	78.907	10.504
ct92-M305Pete-12	120362	09/11/2012	-2	78.908	10.517
ct92-M305Pete-12	120362	09/11/2012	-2	78.91	10.495
ct92-M305Pete-12	120362	09/11/2012	-2	78.918	10.509
ct92-M305Pete-12	120362	09/11/2012	-2	78.911	10.521
ct92-M305Pete-12	120362	10/11/2012	-2	78.904	10.585
ct92-M305Pete-12	120362	10/11/2012	-2	78.912	10.577
ct92-M305Pete-12	120362	10/11/2012	-2	78.904	10.558
ct92-M305Pete-12	120362	10/11/2012	-2	78.915	10.525
ct92-M305Pete-12	120362	10/11/2012	-2	78.92	10.511
ct92-M305Pete-12	120362	10/11/2012	-2	78.917	10.515
ct92-M305Pete-12	120362	10/11/2012	-2	78.925	10.455
ct92-M305Pete-12	120362	10/11/2012	-1	78.911	10.622
ct92-M305Pete-12	120362	10/11/2012	-2	78.909	10.604
ct92-M305Pete-12	120362	10/11/2012	-2	78.919	10.582
ct92-M305Pete-12	120362	10/11/2012	-2	78.915	10.594
ct92-M305Pete-12	120362	10/11/2012	-2	78.932	10.592
ct92-M305Pete-12	120362	10/11/2012	-2	78.934	10.617
ct92-M305Pete-12	120362	10/11/2012	-1	78.922	10.614
ct92-M305Pete-12	120362	10/11/2012	-2	78.923	10.634
ct92-M305Pete-12	120362	10/11/2012	-1	78.912	10.6
ct92-M305Pete-12	120362	10/11/2012	-2	78.941	10.735
ct92-M305Pete-12	120362	10/11/2012	-2	78.895	10.679
ct92-M305Pete-12	120362	10/11/2012	-2	78.919	10.622
ct92-M305Pete-12	120362	10/11/2012	-2	78.916	10.626
ct92-M305Pete-12	120362	10/11/2012	-2	78.919	10.691
ct92-M305Pete-12	120362	10/11/2012	1	78.91	10.775
ct92-M305Pete-12	120362	10/11/2012	-2	78.914	10.752
ct92-M305Pete-12	120362	10/11/2012	-2	78.914	10.762

ct92-M305Pete-12	120362	10/11/2012	0	78.931	10.766
ct92-M305Pete-12	120362	10/11/2012	-2	78.912	10.752
ct92-M305Pete-12	120362	10/11/2012	-2	78.926	10.711
ct92-M305Pete-12	120362	10/11/2012	-2	78.913	10.67
ct92-M305Pete-12	120362	10/11/2012	-2	78.915	10.706
ct92-M305Pete-12	120362	10/11/2012	-2	78.914	10.746
ct92-M305Pete-12	120362	10/11/2012	-1	78.923	10.746
ct92-M305Pete-12	120362	10/11/2012	-2	78.921	10.735
ct92-M305Pete-12	120362	10/11/2012	0	78.92	10.693
ct92-M305Pete-12	120362	10/11/2012	-2	78.923	10.715
ct92-M305Pete-12	120362	10/11/2012	-2	78.923	10.753
ct92-M305Pete-12	120362	10/11/2012	-2	78.909	10.703
ct92-M305Pete-12	120362	10/11/2012	-1	78.902	10.646
ct92-M305Pete-12	120362	10/11/2012	-2	78.922	10.694
ct92-M305Pete-12	120362	10/11/2012	-2	78.9	10.62
ct92-M305Pete-12	120362	10/11/2012	-2	78.903	10.607
ct92-M305Pete-12	120362	10/11/2012	-1	78.93	10.665
ct92-M305Pete-12	120362	10/11/2012	1	78.89	10.634
ct92-M305Pete-12	120362	10/11/2012	-2	78.89	10.641
ct92-M305Pete-12	120362	10/11/2012	-1	78.891	10.662
ct92-M305Pete-12	120362	10/11/2012	-2	78.89	10.692
ct92-M305Pete-12	120362	10/11/2012	-2	78.875	10.705
ct92-M305Pete-12	120362	10/11/2012	-2	78.896	10.652
ct92-M305Pete-12	120362	10/11/2012	1	78.899	10.626
ct92-M305Pete-12	120362	10/11/2012	-2	78.898	10.592
ct92-M305Pete-12	120362	10/11/2012	-2	78.931	10.61
ct92-M305Pete-12	120362	10/11/2012	-2	78.93	10.601
ct92-M305Pete-12	120362	10/11/2012	-2	78.93	10.604
ct92-M305Pete-12	120362	10/11/2012	-2	78.923	10.671
ct92-M305Pete-12	120362	10/11/2012	-2	78.909	10.654
ct92-M305Pete-12	120362	10/11/2012	-2	78.912	10.578
ct92-M305Pete-12	120362	10/11/2012	-1	78.918	10.575
ct92-M305Pete-12	120362	10/11/2012	0	78.919	10.562
ct92-M305Pete-12	120362	10/11/2012	-2	78.919	10.566
ct92-M305Pete-12	120362	10/11/2012	-1	78.936	10.567
ct92-M305Pete-12	120362	10/11/2012	-2	78.943	10.475
ct92-M305Pete-12	120362	10/11/2012	-1	78.885	10.29
ct92-M305Pete-12	120362	10/11/2012	-2	78.909	10.354
ct92-M305Pete-12	120362	10/11/2012	-2	78.905	10.37
ct92-M305Pete-12	120362	10/11/2012	-2	78.917	10.603
ct92-M305Pete-12	120362	10/11/2012	-2	78.911	10.693
ct92-M305Pete-12	120362	10/11/2012	-2	78.91	10.669
ct92-M305Pete-12	120362	10/11/2012	-2	78.888	10.665
ct92-M305Pete-12	120362	10/11/2012	-2	78.891	10.751
ct92-M305Pete-12	120362	10/11/2012	-2	78.889	10.73
ct92-M305Pete-12	120362	10/11/2012	-2	78.899	10.738
ct92-M305Pete-12	120362	10/11/2012	-2	78.891	10.809
ct92-M305Pete-12	120362	11/11/2012	-2	78.901	10.746
ct92-M305Pete-12	120362	11/11/2012	-2	78.927	10.559
ct92-M305Pete-12	120362	11/11/2012	-2	78.907	10.711
ct92-M305Pete-12	120362	11/11/2012	-2	78.896	10.572
ct92-M305Pete-12	120362	11/11/2012	-2	78.883	10.611
ct92-M305Pete-12	120362	11/11/2012	-2	78.879	10.636
ct92-M305Pete-12	120362	11/11/2012	-2	78.88	10.833
ct92-M305Pete-12	120362	11/11/2012	-2	78.858	10.666
ct92-M305Pete-12	120362	11/11/2012	-1	78.905	10.638
ct92-M305Pete-12	120362	11/11/2012	-1	78.904	10.689
ct92-M305Pete-12	120362	11/11/2012	-2	78.905	10.677
ct92-M305Pete-12	120362	11/11/2012	-2	78.908	10.665
ct92-M305Pete-12	120362	11/11/2012	-2	78.908	10.664
ct92-M305Pete-12	120362	11/11/2012	-1	78.917	10.671
ct92-M305Pete-12	120362	11/11/2012	-2	78.906	10.669
ct92-M305Pete-12	120362	11/11/2012	-2	78.925	10.638
ct92-M305Pete-12	120362	11/11/2012	-2	78.92	10.669
ct92-M305Pete-12	120362	11/11/2012	-2	78.929	10.671
ct92-M305Pete-12	120362	11/11/2012	-2	78.924	10.669
ct92-M305Pete-12	120362	11/11/2012	0	78.926	10.65
ct92-M305Pete-12	120362	11/11/2012	-2	78.926	10.638

ct92-M305Pete-12	120362	11/11/2012	-2	78.928	10.644
ct92-M305Pete-12	120362	11/11/2012	-2	78.921	10.65
ct92-M305Pete-12	120362	11/11/2012	-2	78.923	10.675
ct92-M305Pete-12	120362	11/11/2012	-2	78.923	10.656
ct92-M305Pete-12	120362	11/11/2012	-2	78.918	10.781
ct92-M305Pete-12	120362	11/11/2012	-2	78.904	10.729
ct92-M305Pete-12	120362	11/11/2012	0	78.912	10.695
ct92-M305Pete-12	120362	11/11/2012	-2	78.924	10.709
ct92-M305Pete-12	120362	11/11/2012	-2	78.919	10.674
ct92-M305Pete-12	120362	11/11/2012	-2	78.923	10.683
ct92-M305Pete-12	120362	11/11/2012	-2	78.932	10.67
ct92-M305Pete-12	120362	11/11/2012	-2	78.926	10.635
ct92-M305Pete-12	120362	11/11/2012	-2	78.901	10.715
ct92-M305Pete-12	120362	11/11/2012	-2	78.892	10.734
ct92-M305Pete-12	120362	11/11/2012	-2	78.914	10.589
ct92-M305Pete-12	120362	11/11/2012	-2	78.911	10.586
ct92-M305Pete-12	120362	11/11/2012	1	78.927	10.63
ct92-M305Pete-12	120362	11/11/2012	1	78.924	10.612
ct92-M305Pete-12	120362	11/11/2012	-1	78.926	10.609
ct92-M305Pete-12	120362	11/11/2012	0	78.93	10.62
ct92-M305Pete-12	120362	11/11/2012	-1	78.907	10.541
ct92-M305Pete-12	120362	11/11/2012	-2	78.9	10.534
ct92-M305Pete-12	120362	11/11/2012	-2	78.899	10.548
ct92-M305Pete-12	120362	11/11/2012	-1	78.906	10.593
ct92-M305Pete-12	120362	11/11/2012	-2	78.889	10.662
ct92-M305Pete-12	120362	11/11/2012	-2	78.89	10.652
ct92-M305Pete-12	120362	11/11/2012	-2	78.904	10.64
ct92-M305Pete-12	120362	11/11/2012	-2	78.907	10.534
ct92-M305Pete-12	120362	11/11/2012	-2	78.904	10.622
ct92-M305Pete-12	120362	11/11/2012	-2	78.91	10.577
ct92-M305Pete-12	120362	11/11/2012	-2	78.899	10.59
ct92-M305Pete-12	120362	11/11/2012	-2	78.895	10.603
ct92-M305Pete-12	120362	11/11/2012	-2	78.913	10.57
ct92-M305Pete-12	120362	11/11/2012	-1	78.925	10.64
ct92-M305Pete-12	120362	11/11/2012	2	78.918	10.578
ct92-M305Pete-12	120362	11/11/2012	-2	78.918	10.584
ct92-M305Pete-12	120362	11/11/2012	-2	78.918	10.585
ct92-M305Pete-12	120362	11/11/2012	-2	78.932	10.623
ct92-M305Pete-12	120362	11/11/2012	-2	78.932	10.635
ct92-M305Pete-12	120362	11/11/2012	-1	78.931	10.629
ct92-M305Pete-12	120362	11/11/2012	-2	78.92	10.608
ct92-M305Pete-12	120362	11/11/2012	-2	78.934	10.619
ct92-M305Pete-12	120362	11/11/2012	-2	78.916	10.562
ct92-M305Pete-12	120362	11/11/2012	-2	78.933	10.627
ct92-M305Pete-12	120362	11/11/2012	-2	78.934	10.628
ct92-M305Pete-12	120362	11/11/2012	-2	78.899	10.643
ct92-M305Pete-12	120362	11/11/2012	-2	78.92	10.683
ct92-M305Pete-12	120362	12/11/2012	-2	78.916	10.711
ct92-M305Pete-12	120362	12/11/2012	-2	78.915	10.72
ct92-M305Pete-12	120362	12/11/2012	-2	78.924	10.802
ct92-M305Pete-12	120362	12/11/2012	-2	78.919	10.726
ct92-M305Pete-12	120362	12/11/2012	-2	78.911	10.773
ct92-M305Pete-12	120362	12/11/2012	-2	78.913	10.764
ct92-M305Pete-12	120362	12/11/2012	-2	78.908	10.798
ct92-M305Pete-12	120362	12/11/2012	-2	78.914	10.784
ct92-M305Pete-12	120362	12/11/2012	-2	78.912	10.791
ct92-M305Pete-12	120362	12/11/2012	-2	78.908	10.787
ct92-M305Pete-12	120362	12/11/2012	-2	78.905	10.813
ct92-M305Pete-12	120362	12/11/2012	-2	78.91	10.835
ct92-M305Pete-12	120362	12/11/2012	-2	78.903	10.82
ct92-M305Pete-12	120362	12/11/2012	-2	78.911	10.807
ct92-M305Pete-12	120362	12/11/2012	-2	78.907	10.805
ct92-M305Pete-12	120362	12/11/2012	-2	78.907	10.871
ct92-M305Pete-12	120362	12/11/2012	-2	78.908	10.837
ct92-M305Pete-12	120362	12/11/2012	-2	78.912	10.817
ct92-M305Pete-12	120362	12/11/2012	-2	78.905	10.868
ct92-M305Pete-12	120362	12/11/2012	-2	78.909	10.862
ct92-M305Pete-12	120362	12/11/2012	-2	78.907	10.875

ct92-M305Pete-12	120362	12/11/2012	1	78.893	10.936
ct92-M305Pete-12	120362	12/11/2012	-2	78.906	10.94
ct92-M305Pete-12	120362	12/11/2012	-2	78.907	10.9
ct92-M305Pete-12	120362	12/11/2012	-2	78.905	10.908
ct92-M305Pete-12	120362	12/11/2012	-1	78.897	10.871
ct92-M305Pete-12	120362	12/11/2012	-2	78.895	10.897
ct92-M305Pete-12	120362	12/11/2012	-2	78.895	10.906
ct92-M305Pete-12	120362	12/11/2012	-2	78.896	10.889
ct92-M305Pete-12	120362	12/11/2012	-2	78.915	10.885
ct92-M305Pete-12	120362	12/11/2012	-1	78.899	10.821
ct92-M305Pete-12	120362	12/11/2012	-2	78.897	10.929
ct92-M305Pete-12	120362	12/11/2012	-2	78.899	10.826
ct92-M305Pete-12	120362	12/11/2012	-2	78.904	10.78
ct92-M305Pete-12	120362	12/11/2012	-1	78.916	10.734
ct92-M305Pete-12	120362	12/11/2012	-1	78.922	10.758
ct92-M305Pete-12	120362	12/11/2012	-2	78.933	10.919
ct92-M305Pete-12	120362	12/11/2012	-2	78.91	10.887
ct92-M305Pete-12	120362	12/11/2012	-2	78.905	10.803
ct92-M305Pete-12	120362	12/11/2012	-2	78.937	10.863
ct92-M305Pete-12	120362	12/11/2012	-2	78.918	10.644
ct92-M305Pete-12	120362	12/11/2012	-2	78.915	10.756
ct92-M305Pete-12	120362	12/11/2012	-2	78.931	10.798
ct92-M305Pete-12	120362	12/11/2012	-2	78.88	10.457
ct92-M305Pete-12	120362	12/11/2012	-2	78.932	10.77
ct92-M305Pete-12	120362	12/11/2012	-2	78.901	10.467
ct92-M305Pete-12	120362	12/11/2012	-2	78.928	10.518
ct92-M305Pete-12	120362	12/11/2012	-2	78.888	10.231
ct92-M305Pete-12	120362	12/11/2012	-2	78.889	10.382
ct92-M305Pete-12	120362	12/11/2012	-2	78.923	10.64
ct92-M305Pete-12	120362	12/11/2012	-2	78.924	10.613
ct92-M305Pete-12	120362	12/11/2012	-2	78.939	10.302
ct92-M305Pete-12	120362	12/11/2012	-2	78.914	10.415
ct92-M305Pete-12	120362	12/11/2012	-2	78.912	10.37
ct92-M305Pete-12	120362	13/11/2012	-2	78.903	10.496
ct92-M305Pete-12	120362	13/11/2012	-2	78.875	10.184
ct92-M305Pete-12	120362	13/11/2012	-2	78.909	10.51
ct92-M305Pete-12	120362	13/11/2012	-2	78.894	10.393
ct92-M305Pete-12	120362	13/11/2012	-2	78.903	10.413
ct92-M305Pete-12	120362	13/11/2012	-2	78.904	10.418
ct92-M305Pete-12	120362	13/11/2012	-1	78.911	10.486
ct92-M305Pete-12	120362	13/11/2012	-1	78.932	10.561
ct92-M305Pete-12	120362	13/11/2012	-2	78.895	10.439
ct92-M305Pete-12	120362	13/11/2012	-2	78.897	10.438
ct92-M305Pete-12	120362	13/11/2012	-2	78.943	10.608
ct92-M305Pete-12	120362	13/11/2012	-1	79.02	10.226
ct92-M305Pete-12	120362	13/11/2012	-2	78.933	10.54
ct92-M305Pete-12	120362	13/11/2012	-2	78.89	10.545
ct92-M305Pete-12	120362	13/11/2012	-1	78.978	10.583
ct92-M305Pete-12	120362	13/11/2012	-2	78.949	10.606
ct92-M305Pete-12	120362	13/11/2012	-1	78.935	10.762
ct92-M305Pete-12	120362	13/11/2012	-2	78.932	10.686
ct92-M305Pete-12	120362	13/11/2012	-2	78.937	10.702
ct92-M305Pete-12	120362	13/11/2012	-2	78.96	10.717
ct92-M305Pete-12	120362	13/11/2012	-1	78.939	10.644
ct92-M305Pete-12	120362	13/11/2012	0	78.951	10.616
ct92-M305Pete-12	120362	13/11/2012	-2	78.952	10.624
ct92-M305Pete-12	120362	13/11/2012	-2	78.951	10.653
ct92-M305Pete-12	120362	13/11/2012	-2	78.947	10.639
ct92-M305Pete-12	120362	13/11/2012	-1	78.947	10.656
ct92-M305Pete-12	120362	13/11/2012	-2	78.924	10.543
ct92-M305Pete-12	120362	13/11/2012	-2	78.933	10.576
ct92-M305Pete-12	120362	13/11/2012	-2	78.925	10.542
ct92-M305Pete-12	120362	13/11/2012	-2	78.924	10.546
ct92-M305Pete-12	120362	13/11/2012	0	78.916	10.476
ct92-M305Pete-12	120362	13/11/2012	-2	78.911	10.451
ct92-M305Pete-12	120362	13/11/2012	-2	78.912	10.447
ct92-M305Pete-12	120362	13/11/2012	-2	78.912	10.435
ct92-M305Pete-12	120362	13/11/2012	-1	78.91	10.466

ct92-M305Pete-12	120362	13/11/2012	1	78.928	10.516
ct92-M305Pete-12	120362	13/11/2012	-2	78.924	10.507
ct92-M305Pete-12	120362	13/11/2012	-2	78.918	10.5
ct92-M305Pete-12	120362	13/11/2012	-2	78.918	10.503
ct92-M305Pete-12	120362	13/11/2012	-2	78.919	10.52
ct92-M305Pete-12	120362	13/11/2012	-2	78.918	10.501
ct92-M305Pete-12	120362	13/11/2012	-1	78.908	10.517
ct92-M305Pete-12	120362	13/11/2012	-2	78.914	10.567
ct92-M305Pete-12	120362	13/11/2012	-2	78.926	10.584
ct92-M305Pete-12	120362	13/11/2012	-2	78.924	10.592
ct92-M305Pete-12	120362	13/11/2012	-2	78.921	10.597
ct92-M305Pete-12	120362	13/11/2012	-2	78.926	10.601
ct92-M305Pete-12	120362	13/11/2012	-2	78.919	10.607
ct92-M305Pete-12	120362	13/11/2012	-2	78.926	10.633
ct92-M305Pete-12	120362	13/11/2012	1	78.923	10.649
ct92-M305Pete-12	120362	13/11/2012	-2	78.923	10.648
ct92-M305Pete-12	120362	13/11/2012	-1	78.915	10.614
ct92-M305Pete-12	120362	13/11/2012	0	78.927	10.686
ct92-M305Pete-12	120362	13/11/2012	-2	78.924	10.689
ct92-M305Pete-12	120362	13/11/2012	-2	78.923	10.689
ct92-M305Pete-12	120362	13/11/2012	-2	78.919	10.693
ct92-M305Pete-12	120362	14/11/2012	-2	78.91	10.695
ct92-M305Pete-12	120362	14/11/2012	-2	78.903	10.78
ct92-M305Pete-12	120362	14/11/2012	-2	78.879	10.66
ct92-M305Pete-12	120362	14/11/2012	-2	78.887	10.649
ct92-M305Pete-12	120362	14/11/2012	-2	78.89	10.64
ct92-M305Pete-12	120362	14/11/2012	-2	78.876	10.62
ct92-M305Pete-12	120362	14/11/2012	-2	78.946	10.44
ct92-M305Pete-12	120362	14/11/2012	-2	78.964	10.601
ct92-M305Pete-12	120362	14/11/2012	-2	78.913	10.609
ct92-M305Pete-12	120362	14/11/2012	-2	78.933	10.688
ct92-M305Pete-12	120362	14/11/2012	-2	78.923	10.614
ct92-M305Pete-12	120362	14/11/2012	-2	78.939	10.643
ct92-M305Pete-12	120362	14/11/2012	-2	78.917	10.598
ct92-M305Pete-12	120362	14/11/2012	-2	78.926	10.667
ct92-M305Pete-12	120362	14/11/2012	-2	78.922	10.658
ct92-M305Pete-12	120362	14/11/2012	-2	78.929	10.667
ct92-M305Pete-12	120362	14/11/2012	-2	78.915	10.662
ct92-M305Pete-12	120362	14/11/2012	-2	78.933	10.764
ct92-M305Pete-12	120362	14/11/2012	-2	78.924	10.821
ct92-M305Pete-12	120362	14/11/2012	-2	78.929	10.744
ct92-M305Pete-12	120362	14/11/2012	-2	78.908	10.76
ct92-M305Pete-12	120362	14/11/2012	-1	78.929	10.784
ct92-M305Pete-12	120362	14/11/2012	-2	78.911	10.752
ct92-M305Pete-12	120362	14/11/2012	-2	78.92	10.793
ct92-M305Pete-12	120362	14/11/2012	-2	78.92	10.784
ct92-M305Pete-12	120362	14/11/2012	-1	78.898	10.835
ct92-M305Pete-12	120362	14/11/2012	-2	78.884	10.736
ct92-M305Pete-12	120362	14/11/2012	-1	78.892	10.587
ct92-M305Pete-12	120362	14/11/2012	-2	78.904	10.617
ct92-M305Pete-12	120362	14/11/2012	-1	78.91	10.78
ct92-M305Pete-12	120362	14/11/2012	-1	78.921	10.802
ct92-M305Pete-12	120362	14/11/2012	-2	78.878	10.846
ct92-M305Pete-12	120362	14/11/2012	-2	78.884	10.735
ct92-M305Pete-12	120362	14/11/2012	0	78.937	10.78
ct92-M305Pete-12	120362	14/11/2012	-1	78.935	10.713
ct92-M305Pete-12	120362	14/11/2012	-2	78.934	10.762
ct92-M305Pete-12	120362	14/11/2012	-1	78.922	10.738
ct92-M305Pete-12	120362	14/11/2012	-2	78.937	10.695
ct92-M305Pete-12	120362	14/11/2012	-2	78.934	10.674
ct92-M305Pete-12	120362	14/11/2012	-2	78.926	10.666
ct92-M305Pete-12	120362	14/11/2012	-2	78.932	10.618
ct92-M305Pete-12	120362	14/11/2012	-2	78.926	10.636
ct92-M305Pete-12	120362	14/11/2012	-2	78.934	10.662
ct92-M305Pete-12	120362	14/11/2012	-2	78.934	10.674
ct92-M305Pete-12	120362	14/11/2012	-2	78.933	10.651
ct92-M305Pete-12	120362	14/11/2012	-2	78.929	10.645
ct92-M305Pete-12	120362	14/11/2012	-2	78.932	10.613

ct92-M305Pete-12	120362	14/11/2012	-2	78.932	10.613
ct92-M305Pete-12	120362	14/11/2012	-2	78.931	10.63
ct92-M305Pete-12	120362	14/11/2012	-2	78.936	10.642
ct92-M305Pete-12	120362	14/11/2012	-2	78.943	10.512
ct92-M305Pete-12	120362	14/11/2012	-2	78.935	10.51
ct92-M305Pete-12	120362	14/11/2012	-2	78.93	10.549
ct92-M305Pete-12	120362	14/11/2012	-2	78.91	10.489
ct92-M305Pete-12	120362	14/11/2012	-2	78.909	10.486
ct92-M305Pete-12	120362	14/11/2012	-2	78.935	10.624
ct92-M305Pete-12	120362	14/11/2012	-2	78.91	10.562
ct92-M305Pete-12	120362	14/11/2012	-2	78.912	10.691
ct92-M305Pete-12	120362	14/11/2012	-2	78.912	10.691
ct92-M305Pete-12	120362	14/11/2012	-2	78.912	10.691
ct92-M305Pete-12	120362	14/11/2012	-1	78.928	10.684
ct92-M305Pete-12	120362	14/11/2012	-1	78.909	10.636
ct92-M305Pete-12	120362	14/11/2012	-2	78.905	10.561
ct92-M305Pete-12	120362	14/11/2012	-2	78.906	10.564
ct92-M305Pete-12	120362	14/11/2012	-2	78.898	10.704
ct92-M305Pete-12	120362	14/11/2012	-1	78.9	10.597
ct92-M305Pete-12	120362	14/11/2012	-1	78.917	10.586
ct92-M305Pete-12	120362	14/11/2012	-2	78.896	10.569
ct92-M305Pete-12	120362	15/11/2012	-2	78.907	10.642
ct92-M305Pete-12	120362	15/11/2012	-2	78.902	10.653
ct92-M305Pete-12	120362	15/11/2012	-2	78.923	10.574
ct92-M305Pete-12	120362	15/11/2012	-2	78.926	10.592
ct92-M305Pete-12	120362	15/11/2012	-2	78.912	10.674
ct92-M305Pete-12	120362	15/11/2012	-2	78.912	10.674
ct92-M305Pete-12	120362	15/11/2012	-2	78.91	10.675
ct92-M305Pete-12	120362	15/11/2012	-2	78.91	10.686
ct92-M305Pete-12	120362	15/11/2012	-2	78.909	10.624
ct92-M305Pete-12	120362	15/11/2012	-2	78.904	10.726
ct92-M305Pete-12	120362	15/11/2012	-2	78.901	10.689
ct92-M305Pete-12	120362	15/11/2012	-2	78.912	10.664
ct92-M305Pete-12	120362	15/11/2012	-2	78.912	10.668
ct92-M305Pete-12	120362	15/11/2012	-2	78.912	10.651
ct92-M305Pete-12	120362	15/11/2012	-2	78.909	10.658
ct92-M305Pete-12	120362	15/11/2012	-2	78.909	10.658
ct92-M305Pete-12	120362	15/11/2012	-2	78.904	10.66
ct92-M305Pete-12	120362	15/11/2012	-2	78.906	10.65
ct92-M305Pete-12	120362	15/11/2012	-2	78.899	10.65
ct92-M305Pete-12	120362	15/11/2012	-2	78.902	10.633
ct92-M305Pete-12	120362	15/11/2012	-2	78.903	10.632
ct92-M305Pete-12	120362	15/11/2012	-2	78.901	10.637
ct92-M305Pete-12	120362	15/11/2012	-2	78.896	10.641
ct92-M305Pete-12	120362	15/11/2012	-2	78.887	10.641
ct92-M305Pete-12	120362	15/11/2012	-2	78.889	10.651
ct92-M305Pete-12	120362	15/11/2012	-2	78.901	10.622
ct92-M305Pete-12	120362	15/11/2012	-2	78.915	10.662
ct92-M305Pete-12	120362	15/11/2012	-1	78.911	10.66
ct92-M305Pete-12	120362	15/11/2012	-2	78.888	10.677
ct92-M305Pete-12	120362	15/11/2012	-2	78.906	10.644
ct92-M305Pete-12	120362	15/11/2012	-2	78.912	10.651
ct92-M305Pete-12	120362	15/11/2012	-2	78.912	10.654
ct92-M305Pete-12	120362	15/11/2012	-2	78.911	10.65
ct92-M305Pete-12	120362	15/11/2012	-2	78.914	10.757
ct92-M305Pete-12	120362	15/11/2012	-2	78.918	10.699
ct92-M305Pete-12	120362	15/11/2012	-2	78.918	10.699
ct92-M305Pete-12	120362	15/11/2012	-2	78.919	10.702
ct92-M305Pete-12	120362	15/11/2012	-1	78.919	10.697
ct92-M305Pete-12	120362	15/11/2012	-2	78.915	10.702
ct92-M305Pete-12	120362	15/11/2012	-2	78.917	10.765
ct92-M305Pete-12	120362	15/11/2012	-2	78.916	10.728
ct92-M305Pete-12	120362	15/11/2012	-2	78.912	10.695
ct92-M305Pete-12	120362	15/11/2012	-2	78.915	10.724
ct92-M305Pete-12	120362	15/11/2012	-2	78.915	10.734
ct92-M305Pete-12	120362	15/11/2012	-2	78.915	10.731
ct92-M305Pete-12	120362	15/11/2012	-2	78.915	10.729
ct92-M305Pete-12	120362	15/11/2012	2	78.911	10.72

ct92-M305Pete-12	120362	15/11/2012	-2	78.911	10.721
ct92-M305Pete-12	120362	15/11/2012	-1	78.91	10.719
ct92-M305Pete-12	120362	15/11/2012	-1	78.912	10.72
ct92-M305Pete-12	120362	15/11/2012	-2	78.91	10.722
ct92-M305Pete-12	120362	15/11/2012	-2	78.911	10.724
ct92-M305Pete-12	120362	15/11/2012	-2	78.928	10.741
ct92-M305Pete-12	120362	15/11/2012	-2	78.925	10.726
ct92-M305Pete-12	120362	15/11/2012	-2	78.924	10.733
ct92-M305Pete-12	120362	15/11/2012	-2	78.92	10.727
ct92-M305Pete-12	120362	15/11/2012	-2	78.927	10.848
ct92-M305Pete-12	120362	15/11/2012	-2	78.922	10.836
ct92-M305Pete-12	120362	15/11/2012	-2	78.926	10.839
ct92-M305Pete-12	120362	15/11/2012	-2	78.932	10.812
ct92-M305Pete-12	120362	15/11/2012	-2	78.934	10.809
ct92-M305Pete-12	120362	15/11/2012	-2	78.929	10.817
ct92-M305Pete-12	120362	15/11/2012	-2	78.928	10.807
ct92-M305Pete-12	120362	15/11/2012	-2	78.934	10.831
ct92-M305Pete-12	120362	15/11/2012	-2	78.934	10.83
ct92-M305Pete-12	120362	15/11/2012	-2	78.933	10.829
ct92-M305Pete-12	120362	15/11/2012	-2	78.935	10.848
ct92-M305Pete-12	120362	15/11/2012	-2	78.937	10.843
ct92-M305Pete-12	120362	15/11/2012	-2	78.937	10.824
ct92-M305Pete-12	120362	15/11/2012	-2	78.915	10.809
ct92-M305Pete-12	120362	15/11/2012	-2	78.928	10.788
ct92-M305Pete-12	120362	15/11/2012	-2	78.925	10.796
ct92-M305Pete-12	120362	15/11/2012	-2	78.934	10.765
ct92-M305Pete-12	120362	15/11/2012	-2	78.932	10.746
ct92-M305Pete-12	120362	15/11/2012	-2	78.939	10.658
ct92-M305Pete-12	120362	15/11/2012	-2	78.917	10.756
ct92-M305Pete-12	120362	15/11/2012	-2	78.916	10.77
ct92-M305Pete-12	120362	16/11/2012	-2	78.925	10.765
ct92-M305Pete-12	120362	16/11/2012	-2	78.927	10.742
ct92-M305Pete-12	120362	16/11/2012	-2	78.929	10.736
ct92-M305Pete-12	120362	16/11/2012	-2	78.919	10.761
ct92-M305Pete-12	120362	16/11/2012	-2	78.918	10.76
ct92-M305Pete-12	120362	16/11/2012	-2	78.921	10.769
ct92-M305Pete-12	120362	16/11/2012	-2	78.924	10.77
ct92-M305Pete-12	120362	16/11/2012	-2	78.923	10.77
ct92-M305Pete-12	120362	16/11/2012	-2	78.925	10.723
ct92-M305Pete-12	120362	16/11/2012	-2	78.947	10.747
ct92-M305Pete-12	120362	16/11/2012	-1	78.939	10.63
ct92-M305Pete-12	120362	16/11/2012	-2	78.941	10.639
ct92-M305Pete-12	120362	16/11/2012	-2	78.945	10.662
ct92-M305Pete-12	120362	16/11/2012	-2	78.937	10.587
ct92-M305Pete-12	120362	16/11/2012	-2	78.936	10.602
ct92-M305Pete-12	120362	16/11/2012	-2	78.937	10.605
ct92-M305Pete-12	120362	16/11/2012	-2	78.94	10.608
ct92-M305Pete-12	120362	16/11/2012	-2	78.952	10.607
ct92-M305Pete-12	120362	16/11/2012	-2	78.931	10.706
ct92-M305Pete-12	120362	16/11/2012	-2	78.922	10.654
ct92-M305Pete-12	120362	16/11/2012	1	78.931	10.714
ct92-M305Pete-12	120362	16/11/2012	-2	78.932	10.605
ct92-M305Pete-12	120362	16/11/2012	0	78.929	10.673
ct92-M305Pete-12	120362	16/11/2012	-2	78.943	10.752
ct92-M305Pete-12	120362	16/11/2012	-2	78.94	10.754
ct92-M305Pete-12	120362	16/11/2012	2	78.945	10.734
ct92-M305Pete-12	120362	16/11/2012	2	78.942	10.737
ct92-M305Pete-12	120362	16/11/2012	2	78.944	10.734
ct92-M305Pete-12	120362	16/11/2012	1	78.926	10.793
ct92-M305Pete-12	120362	16/11/2012	0	78.964	10.872
ct92-M305Pete-12	120362	16/11/2012	-2	78.949	10.853
ct92-M305Pete-12	120362	16/11/2012	-2	78.949	10.86
ct92-M305Pete-12	120362	16/11/2012	-2	78.94	10.88
ct92-M305Pete-12	120362	16/11/2012	-2	78.945	10.875
ct92-M305Pete-12	120362	16/11/2012	-2	78.937	10.886
ct92-M305Pete-12	120362	16/11/2012	-2	78.949	10.916
ct92-M305Pete-12	120362	16/11/2012	-2	78.936	10.884
ct92-M305Pete-12	120362	16/11/2012	-2	78.929	10.677

ct92-M305Pete-12	120362	16/11/2012	-2	78.915	10.855
ct92-M305Pete-12	120362	16/11/2012	-2	78.921	10.733
ct92-M305Pete-12	120362	16/11/2012	-2	78.926	10.786
ct92-M305Pete-12	120362	16/11/2012	-2	78.931	10.697
ct92-M305Pete-12	120362	16/11/2012	-2	78.93	10.713
ct92-M305Pete-12	120362	16/11/2012	-2	78.916	10.637
ct92-M305Pete-12	120362	16/11/2012	-2	78.916	10.644
ct92-M305Pete-12	120362	16/11/2012	-1	78.928	10.619
ct92-M305Pete-12	120362	16/11/2012	-2	78.918	10.623
ct92-M305Pete-12	120362	16/11/2012	-1	78.924	10.634
ct92-M305Pete-12	120362	16/11/2012	-2	78.925	10.609
ct92-M305Pete-12	120362	16/11/2012	-2	78.922	10.407
ct92-M305Pete-12	120362	16/11/2012	-2	78.917	10.434
ct92-M305Pete-12	120362	16/11/2012	-2	78.909	10.399
ct92-M305Pete-12	120362	16/11/2012	-2	78.908	10.54
ct92-M305Pete-12	120362	16/11/2012	-2	78.911	10.434
ct92-M305Pete-12	120362	16/11/2012	-2	78.911	10.446
ct92-M305Pete-12	120362	16/11/2012	-2	78.909	10.386
ct92-M305Pete-12	120362	16/11/2012	-2	78.917	10.427
ct92-M305Pete-12	120362	16/11/2012	-2	78.91	10.398
ct92-M305Pete-12	120362	16/11/2012	-1	78.915	10.53
ct92-M305Pete-12	120362	16/11/2012	-2	78.92	10.551
ct92-M305Pete-12	120362	16/11/2012	-2	78.918	10.58
ct92-M305Pete-12	120362	17/11/2012	-2	78.92	10.571
ct92-M305Pete-12	120362	17/11/2012	-2	78.921	10.572
ct92-M305Pete-12	120362	17/11/2012	-2	78.922	10.569
ct92-M305Pete-12	120362	17/11/2012	-2	78.91	10.743
ct92-M305Pete-12	120362	17/11/2012	-2	78.906	10.599
ct92-M305Pete-12	120362	17/11/2012	-2	78.903	10.795
ct92-M305Pete-12	120362	17/11/2012	-2	78.906	10.652
ct92-M305Pete-12	120362	17/11/2012	-2	78.895	10.631
ct92-M305Pete-12	120362	17/11/2012	-1	78.93	10.762
ct92-M305Pete-12	120362	17/11/2012	0	78.924	10.685
ct92-M305Pete-12	120362	17/11/2012	-2	78.923	10.697
ct92-M305Pete-12	120362	17/11/2012	-2	78.933	10.677
ct92-M305Pete-12	120362	17/11/2012	-2	78.935	10.683
ct92-M305Pete-12	120362	17/11/2012	-2	78.942	10.741
ct92-M305Pete-12	120362	17/11/2012	-2	78.934	10.772
ct92-M305Pete-12	120362	17/11/2012	-2	78.933	10.769
ct92-M305Pete-12	120362	17/11/2012	-2	78.916	10.796
ct92-M305Pete-12	120362	17/11/2012	1	78.927	10.869
ct92-M305Pete-12	120362	17/11/2012	-2	78.916	10.796
ct92-M305Pete-12	120362	17/11/2012	-2	78.916	10.798
ct92-M305Pete-12	120362	17/11/2012	-2	78.917	10.751
ct92-M305Pete-12	120362	17/11/2012	-2	78.916	10.75
ct92-M305Pete-12	120362	17/11/2012	-2	78.92	10.815
ct92-M305Pete-12	120362	17/11/2012	-2	78.926	10.835
ct92-M305Pete-12	120362	17/11/2012	-2	78.92	10.811
ct92-M305Pete-12	120362	17/11/2012	-2	78.92	10.815
ct92-M305Pete-12	120362	17/11/2012	-2	78.924	10.801
ct92-M305Pete-12	120362	17/11/2012	-2	78.923	10.801
ct92-M305Pete-12	120362	17/11/2012	-2	78.924	10.801
ct92-M305Pete-12	120362	17/11/2012	-2	78.921	10.832
ct92-M305Pete-12	120362	17/11/2012	-2	78.924	10.77
ct92-M305Pete-12	120362	17/11/2012	-2	78.923	10.766
ct92-M305Pete-12	120362	17/11/2012	-2	78.914	10.78
ct92-M305Pete-12	120362	17/11/2012	-2	78.918	10.806
ct92-M305Pete-12	120362	17/11/2012	-1	78.926	10.757
ct92-M305Pete-12	120362	17/11/2012	-2	78.927	10.764
ct92-M305Pete-12	120362	17/11/2012	-2	78.906	10.737
ct92-M305Pete-12	120362	17/11/2012	-2	78.916	10.713
ct92-M305Pete-12	120362	17/11/2012	-2	78.919	10.702
ct92-M305Pete-12	120362	17/11/2012	-2	78.923	10.798
ct92-M305Pete-12	120362	17/11/2012	-2	78.914	10.659
ct92-M305Pete-12	120362	17/11/2012	-2	78.918	10.735
ct92-M305Pete-12	120362	17/11/2012	-2	78.917	10.74
ct92-M305Pete-12	120362	17/11/2012	-2	78.944	10.959
ct92-M305Pete-12	120362	17/11/2012	-1	78.95	10.761

ct92-M305Pete-12	120362	17/11/2012	-2	78.941	10.754
ct92-M305Pete-12	120362	17/11/2012	-2	78.925	10.699
ct92-M305Pete-12	120362	17/11/2012	-2	78.942	10.757
ct92-M305Pete-12	120362	17/11/2012	-1	78.941	10.662
ct92-M305Pete-12	120362	17/11/2012	-1	78.928	10.757
ct92-M305Pete-12	120362	17/11/2012	-2	78.95	10.815
ct92-M305Pete-12	120362	17/11/2012	-2	78.926	10.769
ct92-M305Pete-12	120362	17/11/2012	-2	78.915	10.818
ct92-M305Pete-12	120362	17/11/2012	-2	78.912	10.799
ct92-M305Pete-12	120362	17/11/2012	-2	78.906	10.719
ct92-M305Pete-12	120362	17/11/2012	-1	78.903	10.864
ct92-M305Pete-12	120362	17/11/2012	-2	78.901	10.859
ct92-M305Pete-12	120362	17/11/2012	-2	78.903	10.853
ct92-M305Pete-12	120362	17/11/2012	-1	78.904	10.787
ct92-M305Pete-12	120362	17/11/2012	-2	78.904	10.779
ct92-M305Pete-12	120362	17/11/2012	-2	78.903	10.782
ct92-M305Pete-12	120362	17/11/2012	-2	78.905	10.809
ct92-M305Pete-12	120362	18/11/2012	-2	78.905	10.8
ct92-M305Pete-12	120362	18/11/2012	-2	78.915	10.799
ct92-M305Pete-12	120362	18/11/2012	-2	78.905	10.824
ct92-M305Pete-12	120362	18/11/2012	-2	78.911	10.82
ct92-M305Pete-12	120362	18/11/2012	-2	78.908	10.782
ct92-M305Pete-12	120362	18/11/2012	0	78.937	10.748
ct92-M305Pete-12	120362	18/11/2012	-2	78.94	10.685
ct92-M305Pete-12	120362	18/11/2012	-2	78.973	10.744
ct92-M305Pete-12	120362	18/11/2012	-2	78.932	10.826
ct92-M305Pete-12	120362	18/11/2012	-2	78.924	10.821
ct92-M305Pete-12	120362	18/11/2012	-2	78.928	10.785
ct92-M305Pete-12	120362	18/11/2012	-2	78.923	10.777
ct92-M305Pete-12	120362	18/11/2012	-2	78.936	10.771
ct92-M305Pete-12	120362	18/11/2012	-2	78.937	10.758
ct92-M305Pete-12	120362	18/11/2012	-2	78.924	10.638
ct92-M305Pete-12	120362	18/11/2012	-2	78.946	10.759
ct92-M305Pete-12	120362	18/11/2012	-2	78.945	10.665
ct92-M305Pete-12	120362	18/11/2012	-2	78.936	10.64
ct92-M305Pete-12	120362	18/11/2012	-2	78.933	10.648
ct92-M305Pete-12	120362	18/11/2012	-2	78.948	10.74
ct92-M305Pete-12	120362	18/11/2012	-2	78.929	10.797
ct92-M305Pete-12	120362	18/11/2012	-2	78.934	10.715
ct92-M305Pete-12	120362	18/11/2012	-2	78.933	10.738
ct92-M305Pete-12	120362	18/11/2012	-2	78.948	10.719
ct92-M305Pete-12	120362	18/11/2012	-2	78.937	10.66
ct92-M305Pete-12	120362	18/11/2012	-2	78.94	10.712
ct92-M305Pete-12	120362	18/11/2012	-2	78.939	10.714
ct92-M305Pete-12	120362	18/11/2012	-2	78.93	10.75
ct92-M305Pete-12	120362	18/11/2012	-2	78.932	10.748
ct92-M305Pete-12	120362	18/11/2012	-2	78.929	10.804
ct92-M305Pete-12	120362	18/11/2012	-1	78.931	10.806
ct92-M305Pete-12	120362	18/11/2012	1	78.92	10.783
ct92-M305Pete-12	120362	18/11/2012	-2	78.923	10.785
ct92-M305Pete-12	120362	18/11/2012	-1	78.92	10.799
ct92-M305Pete-12	120362	18/11/2012	-2	78.924	10.802
ct92-M305Pete-12	120362	18/11/2012	1	78.918	10.793
ct92-M305Pete-12	120362	18/11/2012	-2	78.918	10.794
ct92-M305Pete-12	120362	18/11/2012	-2	78.918	10.791
ct92-M305Pete-12	120362	18/11/2012	-2	78.925	10.777
ct92-M305Pete-12	120362	18/11/2012	-2	78.932	10.764
ct92-M305Pete-12	120362	18/11/2012	-2	78.922	10.783
ct92-M305Pete-12	120362	18/11/2012	-2	78.915	10.723
ct92-M305Pete-12	120362	18/11/2012	-2	78.92	10.671
ct92-M305Pete-12	120362	18/11/2012	-1	78.925	10.72
ct92-M305Pete-12	120362	18/11/2012	-2	78.924	10.682
ct92-M305Pete-12	120362	18/11/2012	-2	78.914	10.714
ct92-M305Pete-12	120362	18/11/2012	-2	78.915	10.702
ct92-M305Pete-12	120362	18/11/2012	-2	78.917	10.685
ct92-M305Pete-12	120362	18/11/2012	-2	78.918	10.693
ct92-M305Pete-12	120362	18/11/2012	-2	78.921	10.718
ct92-M305Pete-12	120362	18/11/2012	-2	78.922	10.713

ct92-M305Pete-12	120362	18/11/2012	-2	78.922	10.714
ct92-M305Pete-12	120362	18/11/2012	-2	78.923	10.693
ct92-M305Pete-12	120362	18/11/2012	-2	78.925	10.704
ct92-M305Pete-12	120362	18/11/2012	-2	78.925	10.681
ct92-M305Pete-12	120362	18/11/2012	-2	78.925	10.679
ct92-M305Pete-12	120362	19/11/2012	-2	78.931	10.568
ct92-M305Pete-12	120362	19/11/2012	-2	78.924	10.667
ct92-M305Pete-12	120362	19/11/2012	-1	78.935	10.498
ct92-M305Pete-12	120362	19/11/2012	-2	78.94	10.518
ct92-M305Pete-12	120362	19/11/2012	-2	78.94	10.523
ct92-M305Pete-12	120362	19/11/2012	-2	78.941	10.507
ct92-M305Pete-12	120362	19/11/2012	-2	78.938	10.56
ct92-M305Pete-12	120362	19/11/2012	-2	78.937	10.578
ct92-M305Pete-12	120362	19/11/2012	-2	78.931	10.537
ct92-M305Pete-12	120362	19/11/2012	-2	78.909	10.654
ct92-M305Pete-12	120362	19/11/2012	-1	78.939	10.678
ct92-M305Pete-12	120362	19/11/2012	-2	78.927	10.742
ct92-M305Pete-12	120362	19/11/2012	-2	78.925	10.734
ct92-M305Pete-12	120362	19/11/2012	-2	78.937	10.683
ct92-M305Pete-12	120362	19/11/2012	-2	78.932	10.703
ct92-M305Pete-12	120362	19/11/2012	-2	78.932	10.705
ct92-M305Pete-12	120362	19/11/2012	-2	78.923	10.714
ct92-M305Pete-12	120362	19/11/2012	-2	78.923	10.715
ct92-M305Pete-12	120362	19/11/2012	-2	78.923	10.708
ct92-M305Pete-12	120362	19/11/2012	-2	78.952	10.711
ct92-M305Pete-12	120362	19/11/2012	-2	78.953	10.714
ct92-M305Pete-12	120362	19/11/2012	-2	78.952	10.717
ct92-M305Pete-12	120362	19/11/2012	-2	78.92	10.693
ct92-M305Pete-12	120362	19/11/2012	-2	78.922	10.692
ct92-M305Pete-12	120362	19/11/2012	-2	78.922	10.695
ct92-M305Pete-12	120362	19/11/2012	-2	78.915	10.724
ct92-M305Pete-12	120362	19/11/2012	-2	78.917	10.727
ct92-M305Pete-12	120362	19/11/2012	-2	78.916	10.702
ct92-M305Pete-12	120362	19/11/2012	-2	78.929	10.717
ct92-M305Pete-12	120362	19/11/2012	-2	78.933	10.686
ct92-M305Pete-12	120362	19/11/2012	-2	78.935	10.716
ct92-M305Pete-12	120362	19/11/2012	-2	78.91	10.729
ct92-M305Pete-12	120362	19/11/2012	-2	78.935	10.689
ct92-M305Pete-12	120362	19/11/2012	-2	78.931	10.668
ct92-M305Pete-12	120362	19/11/2012	-2	78.922	10.66
ct92-M305Pete-12	120362	19/11/2012	-2	78.927	10.736
ct92-M305Pete-12	120362	19/11/2012	-2	78.951	10.775
ct92-M305Pete-12	120362	19/11/2012	-2	78.936	10.745
ct92-M305Pete-12	120362	19/11/2012	-1	78.921	10.724
ct92-M305Pete-12	120362	19/11/2012	-2	78.922	10.731
ct92-M305Pete-12	120362	19/11/2012	-2	78.926	10.707
ct92-M305Pete-12	120362	19/11/2012	-2	78.94	10.854
ct92-M305Pete-12	120362	19/11/2012	-2	78.904	10.645
ct92-M305Pete-12	120362	19/11/2012	-2	78.909	10.633
ct92-M305Pete-12	120362	19/11/2012	-2	78.938	10.684
ct92-M305Pete-12	120362	19/11/2012	-2	78.911	10.6
ct92-M305Pete-12	120362	19/11/2012	-2	78.943	10.673
ct92-M305Pete-12	120362	19/11/2012	-2	78.942	10.65
ct92-M305Pete-12	120362	19/11/2012	-2	78.93	10.602
ct92-M305Pete-12	120362	19/11/2012	-2	78.907	10.62
ct92-M305Pete-12	120362	19/11/2012	-2	78.911	10.575
ct92-M305Pete-12	120362	19/11/2012	-2	78.914	10.551
ct92-M305Pete-12	120362	19/11/2012	-2	78.951	10.629
ct92-M305Pete-12	120362	19/11/2012	-2	78.938	10.611
ct92-M305Pete-12	120362	19/11/2012	-2	78.954	10.63
ct92-M305Pete-12	120362	19/11/2012	-2	78.955	10.631
ct92-M305Pete-12	120362	19/11/2012	-2	78.966	10.623
ct92-M305Pete-12	120362	19/11/2012	-2	78.943	10.634
ct92-M305Pete-12	120362	19/11/2012	-2	78.937	10.627
ct92-M305Pete-12	120362	19/11/2012	-2	78.969	10.64
ct92-M305Pete-12	120362	19/11/2012	-2	78.968	10.639
ct92-M305Pete-12	120362	19/11/2012	-2	78.952	10.633
ct92-M305Pete-12	120362	19/11/2012	-2	78.943	10.639

ct92-M305Pete-12	120362	19/11/2012	-2	78.929	10.517
ct92-M305Pete-12	120362	19/11/2012	-2	78.928	10.513
ct92-M305Pete-12	120362	19/11/2012	-2	78.928	10.643
ct92-M305Pete-12	120362	19/11/2012	-1	78.937	10.581
ct92-M305Pete-12	120362	19/11/2012	-2	78.934	10.582
ct92-M305Pete-12	120362	20/11/2012	-2	78.921	10.496
ct92-M305Pete-12	120362	20/11/2012	-2	78.92	10.536
ct92-M305Pete-12	120362	20/11/2012	-2	78.917	10.545
ct92-M305Pete-12	120362	20/11/2012	-2	78.922	10.519
ct92-M305Pete-12	120362	20/11/2012	-1	78.925	10.669
ct92-M305Pete-12	120362	20/11/2012	-1	78.912	10.603
ct92-M305Pete-12	120362	20/11/2012	-2	78.915	10.578
ct92-M305Pete-12	120362	20/11/2012	-2	78.941	10.482
ct92-M305Pete-12	120362	20/11/2012	-2	78.942	10.483
ct92-M305Pete-12	120362	20/11/2012	-2	78.922	10.638
ct92-M305Pete-12	120362	20/11/2012	-2	78.933	10.607
ct92-M305Pete-12	120362	20/11/2012	-2	78.944	10.577
ct92-M305Pete-12	120362	20/11/2012	-2	78.938	10.609
ct92-M305Pete-12	120362	20/11/2012	-2	78.963	10.674
ct92-M305Pete-12	120362	20/11/2012	-2	78.949	10.604
ct92-M305Pete-12	120362	20/11/2012	-2	78.962	10.538
ct92-M305Pete-12	120362	20/11/2012	-2	78.954	10.549
ct92-M305Pete-12	120362	20/11/2012	-2	78.933	10.605
ct92-M305Pete-12	120362	20/11/2012	-2	78.966	10.528
ct92-M305Pete-12	120362	20/11/2012	-2	78.945	10.531
ct92-M305Pete-12	120362	20/11/2012	-2	78.937	10.668
ct92-M305Pete-12	120362	20/11/2012	-2	78.936	10.681
ct92-M305Pete-12	120362	20/11/2012	-2	78.979	10.638
ct92-M305Pete-12	120362	20/11/2012	0	78.972	10.645
ct92-M305Pete-12	120362	20/11/2012	-2	78.939	10.698
ct92-M305Pete-12	120362	20/11/2012	-1	78.944	10.773
ct92-M305Pete-12	120362	20/11/2012	-2	78.947	10.778
ct92-M305Pete-12	120362	20/11/2012	-2	78.947	10.792
ct92-M305Pete-12	120362	20/11/2012	-2	78.945	10.788
ct92-M305Pete-12	120362	20/11/2012	-2	78.949	10.786
ct92-M305Pete-12	120362	20/11/2012	-2	78.947	10.797
ct92-M305Pete-12	120362	20/11/2012	-2	78.945	10.784
ct92-M305Pete-12	120362	20/11/2012	-1	78.952	10.794
ct92-M305Pete-12	120362	20/11/2012	-2	78.941	10.755
ct92-M305Pete-12	120362	20/11/2012	-1	78.942	10.747
ct92-M305Pete-12	120362	20/11/2012	-2	78.95	10.713
ct92-M305Pete-12	120362	20/11/2012	-2	78.958	10.707
ct92-M305Pete-12	120362	20/11/2012	-2	78.955	10.687
ct92-M305Pete-12	120362	20/11/2012	-1	78.932	10.713
ct92-M305Pete-12	120362	20/11/2012	-1	78.942	10.698
ct92-M305Pete-12	120362	20/11/2012	1	78.932	10.688
ct92-M305Pete-12	120362	20/11/2012	-2	78.955	10.716
ct92-M305Pete-12	120362	20/11/2012	-2	78.938	10.694
ct92-M305Pete-12	120362	20/11/2012	-2	78.918	10.778
ct92-M305Pete-12	120362	20/11/2012	2	78.934	10.669
ct92-M305Pete-12	120362	20/11/2012	0	78.933	10.723
ct92-M305Pete-12	120362	20/11/2012	-2	78.921	10.688
ct92-M305Pete-12	120362	20/11/2012	2	78.925	10.713
ct92-M305Pete-12	120362	20/11/2012	-2	78.942	10.696
ct92-M305Pete-12	120362	20/11/2012	-1	78.929	10.714
ct92-M305Pete-12	120362	20/11/2012	-2	78.946	10.765
ct92-M305Pete-12	120362	20/11/2012	-1	78.931	10.672
ct92-M305Pete-12	120362	20/11/2012	-2	78.929	10.68
ct92-M305Pete-12	120362	20/11/2012	-2	78.926	10.624
ct92-M305Pete-12	120362	20/11/2012	-2	78.924	10.642
ct92-M305Pete-12	120362	20/11/2012	-2	78.929	10.659
ct92-M305Pete-12	120362	20/11/2012	-2	78.929	10.652
ct92-M305Pete-12	120362	20/11/2012	-2	78.933	10.721
ct92-M305Pete-12	120362	20/11/2012	-2	78.918	10.73
ct92-M305Pete-12	120362	20/11/2012	-1	78.924	10.571
ct92-M305Pete-12	120362	20/11/2012	-2	78.923	10.566
ct92-M305Pete-12	120362	20/11/2012	-2	78.937	10.508
ct92-M305Pete-12	120362	20/11/2012	-2	78.922	10.565

ct92-M305Pete-12	120362	20/11/2012	-2	78.926	10.603
ct92-M305Pete-12	120362	20/11/2012	-2	78.923	10.596
ct92-M305Pete-12	120362	20/11/2012	-2	78.935	10.638
ct92-M305Pete-12	120362	20/11/2012	-2	78.939	10.615
ct92-M305Pete-12	120362	20/11/2012	-2	78.937	10.61
ct92-M305Pete-12	120362	20/11/2012	-2	78.936	10.612
ct92-M305Pete-12	120362	20/11/2012	-2	78.921	10.571
ct92-M305Pete-12	120362	20/11/2012	-2	78.928	10.53
ct92-M305Pete-12	120362	20/11/2012	-2	78.921	10.518
ct92-M305Pete-12	120362	21/11/2012	-2	78.924	10.522
ct92-M305Pete-12	120362	21/11/2012	-1	78.933	10.553
ct92-M305Pete-12	120362	21/11/2012	-2	78.934	10.554
ct92-M305Pete-12	120362	21/11/2012	-2	78.934	10.569
ct92-M305Pete-12	120362	21/11/2012	-2	78.909	10.649
ct92-M305Pete-12	120362	21/11/2012	-2	78.895	10.683
ct92-M305Pete-12	120362	21/11/2012	-2	78.894	10.687
ct92-M305Pete-12	120362	21/11/2012	-2	78.914	10.543
ct92-M305Pete-12	120362	21/11/2012	-2	78.914	10.545
ct92-M305Pete-12	120362	21/11/2012	-2	78.905	10.619
ct92-M305Pete-12	120362	21/11/2012	-2	78.914	10.552
ct92-M305Pete-12	120362	21/11/2012	-2	78.912	10.585
ct92-M305Pete-12	120362	21/11/2012	-2	78.914	10.59
ct92-M305Pete-12	120362	21/11/2012	-2	78.898	10.612
ct92-M305Pete-12	120362	21/11/2012	-2	78.897	10.626
ct92-M305Pete-12	120362	21/11/2012	-2	78.898	10.641
ct92-M305Pete-12	120362	21/11/2012	-2	78.899	10.647
ct92-M305Pete-12	120362	21/11/2012	0	78.912	10.635
ct92-M305Pete-12	120362	21/11/2012	-2	78.914	10.643
ct92-M305Pete-12	120362	21/11/2012	-2	78.915	10.641
ct92-M305Pete-12	120362	21/11/2012	-2	78.908	10.651
ct92-M305Pete-12	120362	21/11/2012	-2	78.914	10.663
ct92-M305Pete-12	120362	21/11/2012	-2	78.914	10.596
ct92-M305Pete-12	120362	21/11/2012	-2	78.931	10.602
ct92-M305Pete-12	120362	21/11/2012	-2	78.929	10.646
ct92-M305Pete-12	120362	21/11/2012	-2	78.937	10.632
ct92-M305Pete-12	120362	21/11/2012	-2	78.95	10.711
ct92-M305Pete-12	120362	21/11/2012	-1	78.951	10.72
ct92-M305Pete-12	120362	21/11/2012	-2	78.938	10.738
ct92-M305Pete-12	120362	21/11/2012	-2	78.926	10.722
ct92-M305Pete-12	120362	21/11/2012	-2	78.94	10.764
ct92-M305Pete-12	120362	21/11/2012	-2	78.94	10.762
ct92-M305Pete-12	120362	21/11/2012	-2	78.949	10.751
ct92-M305Pete-12	120362	21/11/2012	-2	78.945	10.747
ct92-M305Pete-12	120362	21/11/2012	-2	78.95	10.738
ct92-M305Pete-12	120362	21/11/2012	-2	78.939	10.744
ct92-M305Pete-12	120362	21/11/2012	-2	78.934	10.727
ct92-M305Pete-12	120362	21/11/2012	-1	78.953	10.758
ct92-M305Pete-12	120362	21/11/2012	-2	78.971	10.834
ct92-M305Pete-12	120362	21/11/2012	-2	78.941	10.606
ct92-M305Pete-12	120362	21/11/2012	-2	78.949	10.629
ct92-M305Pete-12	120362	21/11/2012	-2	78.932	10.576
ct92-M305Pete-12	120362	21/11/2012	-2	78.934	10.582
ct92-M305Pete-12	120362	21/11/2012	-2	78.938	10.591
ct92-M305Pete-12	120362	21/11/2012	-2	78.943	10.589
ct92-M305Pete-12	120362	21/11/2012	-2	78.941	10.587
ct92-M305Pete-12	120362	21/11/2012	-2	78.936	10.549
ct92-M305Pete-12	120362	21/11/2012	-2	78.932	10.572
ct92-M305Pete-12	120362	21/11/2012	-2	78.932	10.57
ct92-M305Pete-12	120362	21/11/2012	-1	78.925	10.536
ct92-M305Pete-12	120362	21/11/2012	-2	78.915	10.579
ct92-M305Pete-12	120362	21/11/2012	-2	78.91	10.538
ct92-M305Pete-12	120362	21/11/2012	-2	78.909	10.462
ct92-M305Pete-12	120362	21/11/2012	-2	78.923	10.459
ct92-M305Pete-12	120362	21/11/2012	-1	78.944	10.609
ct92-M305Pete-12	120362	21/11/2012	-2	78.917	10.551
ct92-M305Pete-12	120362	21/11/2012	-2	78.918	10.544
ct92-M305Pete-12	120362	21/11/2012	-2	78.923	10.551
ct92-M305Pete-12	120362	21/11/2012	-2	78.925	10.529

ct92-M305Pete-12	120362	21/11/2012	-2	78.936	10.565
ct92-M305Pete-12	120362	21/11/2012	-2	78.936	10.565
ct92-M305Pete-12	120362	21/11/2012	-2	78.921	10.467
ct92-M305Pete-12	120362	21/11/2012	-2	78.921	10.522
ct92-M305Pete-12	120362	21/11/2012	-2	78.919	10.529
ct92-M305Pete-12	120362	21/11/2012	-2	78.919	10.528
ct92-M305Pete-12	120362	21/11/2012	-2	78.932	10.577
ct92-M305Pete-12	120362	21/11/2012	-2	78.933	10.576
ct92-M305Pete-12	120362	21/11/2012	-2	78.934	10.6
ct92-M305Pete-12	120362	21/11/2012	-2	78.935	10.604
ct92-M305Pete-12	120362	21/11/2012	-2	78.936	10.602
ct92-M305Pete-12	120362	21/11/2012	-2	78.935	10.601
ct92-M305Pete-12	120362	21/11/2012	-2	78.935	10.6
ct92-M305Pete-12	120362	21/11/2012	-2	78.93	10.62
ct92-M305Pete-12	120362	22/11/2012	-2	78.933	10.679
ct92-M305Pete-12	120362	22/11/2012	-2	78.928	10.665
ct92-M305Pete-12	120362	22/11/2012	-2	78.928	10.672
ct92-M305Pete-12	120362	22/11/2012	-2	78.928	10.669
ct92-M305Pete-12	120362	22/11/2012	-2	78.928	10.674
ct92-M305Pete-12	120362	22/11/2012	-2	78.928	10.665
ct92-M305Pete-12	120362	22/11/2012	-2	78.928	10.561
ct92-M305Pete-12	120362	22/11/2012	-2	78.934	10.573
ct92-M305Pete-12	120362	22/11/2012	-2	78.933	10.559
ct92-M305Pete-12	120362	22/11/2012	-2	78.916	10.592
ct92-M305Pete-12	120362	22/11/2012	-2	78.916	10.59
ct92-M305Pete-12	120362	22/11/2012	-2	78.912	10.558
ct92-M305Pete-12	120362	22/11/2012	-2	78.911	10.525
ct92-M305Pete-12	120362	22/11/2012	-2	78.911	10.523
ct92-M305Pete-12	120362	22/11/2012	-2	78.923	10.571
ct92-M305Pete-12	120362	22/11/2012	-2	78.924	10.567
ct92-M305Pete-12	120362	22/11/2012	-2	78.924	10.569
ct92-M305Pete-12	120362	22/11/2012	-2	78.912	10.629
ct92-M305Pete-12	120362	22/11/2012	-2	78.915	10.685
ct92-M305Pete-12	120362	22/11/2012	-2	78.911	10.687
ct92-M305Pete-12	120362	22/11/2012	-2	78.912	10.686
ct92-M305Pete-12	120362	22/11/2012	-2	78.938	10.537
ct92-M305Pete-12	120362	22/11/2012	-2	78.927	10.651
ct92-M305Pete-12	120362	22/11/2012	-2	78.935	10.517
ct92-M305Pete-12	120362	22/11/2012	-2	78.94	10.55
ct92-M305Pete-12	120362	22/11/2012	-2	78.94	10.559
ct92-M305Pete-12	120362	22/11/2012	-2	78.941	10.55
ct92-M305Pete-12	120362	22/11/2012	-2	78.942	10.545
ct92-M305Pete-12	120362	22/11/2012	-2	78.945	10.562
ct92-M305Pete-12	120362	22/11/2012	-2	78.944	10.528
ct92-M305Pete-12	120362	22/11/2012	-2	78.943	10.585
ct92-M305Pete-12	120362	22/11/2012	-1	78.928	10.688
ct92-M305Pete-12	120362	22/11/2012	-2	78.946	10.578
ct92-M305Pete-12	120362	22/11/2012	-2	78.928	10.702
ct92-M305Pete-12	120362	22/11/2012	-2	78.926	10.711
ct92-M305Pete-12	120362	22/11/2012	-2	78.919	10.692
ct92-M305Pete-12	120362	22/11/2012	-2	78.928	10.679
ct92-M305Pete-12	120362	22/11/2012	-2	78.925	10.681
ct92-M305Pete-12	120362	22/11/2012	-2	78.921	10.694
ct92-M305Pete-12	120362	22/11/2012	-2	78.935	10.755
ct92-M305Pete-12	120362	22/11/2012	-2	78.935	10.754
ct92-M305Pete-12	120362	22/11/2012	-2	78.936	10.772
ct92-M305Pete-12	120362	22/11/2012	-1	78.931	10.765
ct92-M305Pete-12	120362	22/11/2012	-1	78.936	10.766
ct92-M305Pete-12	120362	22/11/2012	-2	78.937	10.739
ct92-M305Pete-12	120362	22/11/2012	-2	78.912	10.8
ct92-M305Pete-12	120362	22/11/2012	-2	78.941	10.763
ct92-M305Pete-12	120362	22/11/2012	-2	78.94	10.724
ct92-M305Pete-12	120362	22/11/2012	-2	78.911	10.739
ct92-M305Pete-12	120362	22/11/2012	-2	78.908	10.784
ct92-M305Pete-12	120362	22/11/2012	-2	78.912	10.813
ct92-M305Pete-12	120362	22/11/2012	-2	78.888	10.837
ct92-M305Pete-12	120362	22/11/2012	-1	78.885	10.82
ct92-M305Pete-12	120362	22/11/2012	-2	78.885	10.824

ct92-M305Pete-12	120362	22/11/2012	-2	78.893	10.83
ct92-M305Pete-12	120362	22/11/2012	0	78.885	10.847
ct92-M305Pete-12	120362	22/11/2012	-2	78.885	10.846
ct92-M305Pete-12	120362	22/11/2012	-2	78.89	10.811
ct92-M305Pete-12	120362	22/11/2012	-2	78.891	10.842
ct92-M305Pete-12	120362	22/11/2012	-2	78.889	10.77
ct92-M305Pete-12	120362	22/11/2012	-2	78.89	10.839
ct92-M305Pete-12	120362	22/11/2012	-2	78.89	10.836
ct92-M305Pete-12	120362	22/11/2012	-2	78.869	10.861
ct92-M305Pete-12	120362	22/11/2012	-1	78.878	10.869
ct92-M305Pete-12	120362	22/11/2012	-2	78.879	10.86
ct92-M305Pete-12	120362	22/11/2012	-2	78.879	10.862
ct92-M305Pete-12	120362	22/11/2012	-2	78.861	10.878
ct92-M305Pete-12	120362	22/11/2012	-2	78.874	10.867
ct92-M305Pete-12	120362	22/11/2012	-2	78.904	10.839
ct92-M305Pete-12	120362	22/11/2012	-2	78.896	10.772
ct92-M305Pete-12	120362	22/11/2012	-2	78.888	10.765
ct92-M305Pete-12	120362	22/11/2012	-2	78.885	10.775
ct92-M305Pete-12	120362	22/11/2012	-2	78.881	10.805
ct92-M305Pete-12	120362	22/11/2012	-2	78.892	10.785
ct92-M305Pete-12	120362	22/11/2012	-2	78.891	10.784
ct92-M305Pete-12	120362	22/11/2012	-2	78.896	10.811
ct92-M305Pete-12	120362	23/11/2012	-2	78.897	10.715
ct92-M305Pete-12	120362	23/11/2012	-1	78.895	10.758
ct92-M305Pete-12	120362	23/11/2012	-2	78.895	10.751
ct92-M305Pete-12	120362	23/11/2012	-2	78.894	10.749
ct92-M305Pete-12	120362	23/11/2012	-2	78.893	10.748
ct92-M305Pete-12	120362	23/11/2012	-2	78.913	10.705
ct92-M305Pete-12	120362	23/11/2012	-2	78.912	10.736
ct92-M305Pete-12	120362	23/11/2012	-2	78.913	10.738
ct92-M305Pete-12	120362	23/11/2012	-1	78.906	10.729
ct92-M305Pete-12	120362	23/11/2012	-1	78.908	10.684
ct92-M305Pete-12	120362	23/11/2012	-2	78.903	10.646
ct92-M305Pete-12	120362	23/11/2012	-2	78.906	10.688
ct92-M305Pete-12	120362	23/11/2012	-2	78.905	10.685
ct92-M305Pete-12	120362	23/11/2012	-2	78.904	10.676
ct92-M305Pete-12	120362	23/11/2012	-2	78.903	10.68
ct92-M305Pete-12	120362	23/11/2012	-2	78.902	10.68
ct92-M305Pete-12	120362	23/11/2012	-2	78.901	10.646
ct92-M305Pete-12	120362	23/11/2012	-2	78.9	10.646
ct92-M305Pete-12	120362	23/11/2012	-2	78.898	10.648
ct92-M305Pete-12	120362	23/11/2012	-2	78.899	10.647
ct92-M305Pete-12	120362	23/11/2012	-2	78.899	10.646
ct92-M305Pete-12	120362	23/11/2012	-2	78.897	10.648
ct92-M305Pete-12	120362	23/11/2012	-2	78.896	10.62
ct92-M305Pete-12	120362	23/11/2012	-2	78.896	10.621
ct92-M305Pete-12	120362	23/11/2012	-2	78.898	10.622
ct92-M305Pete-12	120362	23/11/2012	-2	78.899	10.575
ct92-M305Pete-12	120362	23/11/2012	-2	78.898	10.621
ct92-M305Pete-12	120362	23/11/2012	-2	78.896	10.622
ct92-M305Pete-12	120362	23/11/2012	-2	78.896	10.623
ct92-M305Pete-12	120362	23/11/2012	-2	78.896	10.622
ct92-M305Pete-12	120362	23/11/2012	-2	78.9	10.624
ct92-M305Pete-12	120362	23/11/2012	-2	78.9	10.606
ct92-M305Pete-12	120362	23/11/2012	-2	78.899	10.612
ct92-M305Pete-12	120362	23/11/2012	-2	78.899	10.601
ct92-M305Pete-12	120362	23/11/2012	-2	78.9	10.606
ct92-M305Pete-12	120362	23/11/2012	-2	78.902	10.574
ct92-M305Pete-12	120362	23/11/2012	-2	78.904	10.551
ct92-M305Pete-12	120362	23/11/2012	-2	78.906	10.559
ct92-M305Pete-12	120362	23/11/2012	-2	78.905	10.605
ct92-M305Pete-12	120362	23/11/2012	-2	78.91	10.568
ct92-M305Pete-12	120362	23/11/2012	-2	78.91	10.571
ct92-M305Pete-12	120362	23/11/2012	-2	78.911	10.567
ct92-M305Pete-12	120362	23/11/2012	-2	78.911	10.567
ct92-M305Pete-12	120362	23/11/2012	-2	78.917	10.539
ct92-M305Pete-12	120362	23/11/2012	-2	78.924	10.538
ct92-M305Pete-12	120362	23/11/2012	-2	78.922	10.537

ct92-M305Pete-12	120362	23/11/2012	-2	78.913	10.477
ct92-M305Pete-12	120362	23/11/2012	-2	78.917	10.416
ct92-M305Pete-12	120362	23/11/2012	-1	78.948	10.51
ct92-M305Pete-12	120362	23/11/2012	-2	78.945	10.531
ct92-M305Pete-12	120362	23/11/2012	-2	78.937	10.561
ct92-M305Pete-12	120362	23/11/2012	-2	78.931	10.597
ct92-M305Pete-12	120362	23/11/2012	-2	78.924	10.572
ct92-M305Pete-12	120362	23/11/2012	-2	78.923	10.571
ct92-M305Pete-12	120362	23/11/2012	-2	78.917	10.548
ct92-M305Pete-12	120362	23/11/2012	-2	78.916	10.547
ct92-M305Pete-12	120362	23/11/2012	-2	78.917	10.548
ct92-M305Pete-12	120362	23/11/2012	-2	78.918	10.541
ct92-M305Pete-12	120362	24/11/2012	-2	78.922	10.545
ct92-M305Pete-12	120362	24/11/2012	-1	78.944	10.597
ct92-M305Pete-12	120362	24/11/2012	-2	78.912	10.584
ct92-M305Pete-12	120362	24/11/2012	-2	78.913	10.588
ct92-M305Pete-12	120362	24/11/2012	-2	78.916	10.598
ct92-M305Pete-12	120362	24/11/2012	-2	78.918	10.63
ct92-M305Pete-12	120362	24/11/2012	-2	78.93	10.589
ct92-M305Pete-12	120362	24/11/2012	-2	78.927	10.693
ct92-M305Pete-12	120362	24/11/2012	-2	78.932	10.688
ct92-M305Pete-12	120362	24/11/2012	-2	78.936	10.68
ct92-M305Pete-12	120362	24/11/2012	-2	78.927	10.685
ct92-M305Pete-12	120362	24/11/2012	-2	78.938	10.624
ct92-M305Pete-12	120362	24/11/2012	-2	78.944	10.563
ct92-M305Pete-12	120362	24/11/2012	-2	78.934	10.59
ct92-M305Pete-12	120362	24/11/2012	-1	78.943	10.532
ct92-M305Pete-12	120362	24/11/2012	-2	78.939	10.551
ct92-M305Pete-12	120362	24/11/2012	-1	78.926	10.53
ct92-M305Pete-12	120362	24/11/2012	-2	78.925	10.579
ct92-M305Pete-12	120362	24/11/2012	-1	78.918	10.606
ct92-M305Pete-12	120362	24/11/2012	-1	78.918	10.531
ct92-M305Pete-12	120362	24/11/2012	-2	78.916	10.584
ct92-M305Pete-12	120362	24/11/2012	-2	78.916	10.573
ct92-M305Pete-12	120362	24/11/2012	-2	78.91	10.534
ct92-M305Pete-12	120362	24/11/2012	-2	78.922	10.488
ct92-M305Pete-12	120362	24/11/2012	-2	78.938	10.494
ct92-M305Pete-12	120362	24/11/2012	1	78.915	10.445
ct92-M305Pete-12	120362	24/11/2012	-2	78.922	10.427
ct92-M305Pete-12	120362	24/11/2012	-2	78.913	10.424
ct92-M305Pete-12	120362	24/11/2012	-2	78.909	10.422
ct92-M305Pete-12	120362	24/11/2012	-2	78.909	10.406
ct92-M305Pete-12	120362	24/11/2012	-2	78.914	10.389
ct92-M305Pete-12	120362	24/11/2012	-2	78.913	10.426
ct92-M305Pete-12	120362	24/11/2012	-2	78.914	10.381
ct92-M305Pete-12	120362	24/11/2012	-2	78.919	10.395
ct92-M305Pete-12	120362	24/11/2012	-2	78.904	10.416
ct92-M305Pete-12	120362	24/11/2012	-2	78.907	10.407
ct92-M305Pete-12	120362	24/11/2012	-2	78.921	10.443
ct92-M305Pete-12	120362	24/11/2012	-2	78.925	10.405
ct92-M305Pete-12	120362	24/11/2012	-2	78.926	10.429
ct92-M305Pete-12	120362	24/11/2012	-1	78.935	10.496
ct92-M305Pete-12	120362	24/11/2012	-2	78.934	10.486
ct92-M305Pete-12	120362	24/11/2012	-2	78.923	10.457
ct92-M305Pete-12	120362	24/11/2012	-2	78.918	10.479
ct92-M305Pete-12	120362	24/11/2012	0	78.919	10.451
ct92-M305Pete-12	120362	24/11/2012	-2	78.934	10.505
ct92-M305Pete-12	120362	24/11/2012	-2	78.934	10.505
ct92-M305Pete-12	120362	24/11/2012	-2	78.937	10.48
ct92-M305Pete-12	120362	24/11/2012	-2	78.921	10.442
ct92-M305Pete-12	120362	24/11/2012	-1	78.913	10.397
ct92-M305Pete-12	120362	24/11/2012	-1	78.943	10.389
ct92-M305Pete-12	120362	24/11/2012	-2	78.934	10.339
ct92-M305Pete-12	120362	24/11/2012	-2	78.933	10.403
ct92-M305Pete-12	120362	24/11/2012	-2	78.935	10.348
ct92-M305Pete-12	120362	24/11/2012	-2	78.928	10.389
ct92-M305Pete-12	120362	24/11/2012	-2	78.923	10.367
ct92-M305Pete-12	120362	24/11/2012	-2	78.932	10.393

ct92-M305Pete-12	120362	24/11/2012	-2	78.931	10.4
ct92-M305Pete-12	120362	24/11/2012	-2	78.94	10.473
ct92-M305Pete-12	120362	24/11/2012	-2	78.935	10.484
ct92-M305Pete-12	120362	24/11/2012	-1	78.948	10.63
ct92-M305Pete-12	120362	24/11/2012	-2	78.939	10.607
ct92-M305Pete-12	120362	24/11/2012	-2	78.937	10.594
ct92-M305Pete-12	120362	24/11/2012	-2	78.934	10.606
ct92-M305Pete-12	120362	24/11/2012	-2	78.935	10.614
ct92-M305Pete-12	120362	24/11/2012	-2	78.929	10.585
ct92-M305Pete-12	120362	24/11/2012	-2	78.929	10.598
ct92-M305Pete-12	120362	24/11/2012	-2	78.923	10.624
ct92-M305Pete-12	120362	24/11/2012	-2	78.914	10.676
ct92-M305Pete-12	120362	24/11/2012	-2	78.912	10.669
ct92-M305Pete-12	120362	24/11/2012	-2	78.921	10.638
ct92-M305Pete-12	120362	24/11/2012	-2	78.917	10.636
ct92-M305Pete-12	120362	24/11/2012	-2	78.918	10.637
ct92-M305Pete-12	120362	24/11/2012	-2	78.932	10.655
ct92-M305Pete-12	120362	24/11/2012	-2	78.931	10.665
ct92-M305Pete-12	120362	24/11/2012	-2	78.924	10.7
ct92-M305Pete-12	120362	24/11/2012	-2	78.926	10.77
ct92-M305Pete-12	120362	24/11/2012	-2	78.923	10.784
ct92-M305Pete-12	120362	24/11/2012	-1	78.925	10.756
ct92-M305Pete-12	120362	24/11/2012	-2	78.925	10.711
ct92-M305Pete-12	120362	25/11/2012	-2	78.928	10.71
ct92-M305Pete-12	120362	25/11/2012	-2	78.929	10.713
ct92-M305Pete-12	120362	25/11/2012	-2	78.904	10.771
ct92-M305Pete-12	120362	25/11/2012	-2	78.908	10.717
ct92-M305Pete-12	120362	25/11/2012	-2	78.911	10.719
ct92-M305Pete-12	120362	25/11/2012	-2	78.891	10.755
ct92-M305Pete-12	120362	25/11/2012	1	78.928	10.685
ct92-M305Pete-12	120362	25/11/2012	-2	78.924	10.693
ct92-M305Pete-12	120362	25/11/2012	-2	78.932	10.681
ct92-M305Pete-12	120362	25/11/2012	-1	78.926	10.762
ct92-M305Pete-12	120362	25/11/2012	-2	78.927	10.743
ct92-M305Pete-12	120362	25/11/2012	-2	78.914	10.712
ct92-M305Pete-12	120362	25/11/2012	-2	78.934	10.724
ct92-M305Pete-12	120362	25/11/2012	-2	78.917	10.73
ct92-M305Pete-12	120362	25/11/2012	-2	78.895	10.773
ct92-M305Pete-12	120362	25/11/2012	-2	78.912	10.833
ct92-M305Pete-12	120362	25/11/2012	-2	78.922	10.83
ct92-M305Pete-12	120362	25/11/2012	-2	78.92	10.828
ct92-M305Pete-12	120362	25/11/2012	-2	78.927	10.707
ct92-M305Pete-12	120362	25/11/2012	-1	78.906	10.814
ct92-M305Pete-12	120362	25/11/2012	-1	78.913	10.835
ct92-M305Pete-12	120362	25/11/2012	-2	78.905	10.773
ct92-M305Pete-12	120362	25/11/2012	-2	78.9	10.779
ct92-M305Pete-12	120362	25/11/2012	-2	78.901	10.786
ct92-M305Pete-12	120362	25/11/2012	0	78.94	10.643
ct92-M305Pete-12	120362	25/11/2012	-2	78.925	10.606
ct92-M305Pete-12	120362	25/11/2012	-2	78.943	10.624
ct92-M305Pete-12	120362	25/11/2012	-2	78.942	10.686
ct92-M305Pete-12	120362	25/11/2012	-2	78.943	10.701
ct92-M305Pete-12	120362	25/11/2012	-2	78.915	10.731
ct92-M305Pete-12	120362	25/11/2012	-1	78.916	10.736
ct92-M305Pete-12	120362	25/11/2012	1	78.92	10.659
ct92-M305Pete-12	120362	25/11/2012	-1	78.913	10.643
ct92-M305Pete-12	120362	25/11/2012	-2	78.914	10.639
ct92-M305Pete-12	120362	25/11/2012	1	78.913	10.66
ct92-M305Pete-12	120362	25/11/2012	-2	78.922	10.667
ct92-M305Pete-12	120362	25/11/2012	-2	78.913	10.623
ct92-M305Pete-12	120362	25/11/2012	-2	78.91	10.64
ct92-M305Pete-12	120362	25/11/2012	-2	78.908	10.593
ct92-M305Pete-12	120362	25/11/2012	-2	78.909	10.582
ct92-M305Pete-12	120362	25/11/2012	-2	78.908	10.624
ct92-M305Pete-12	120362	25/11/2012	-2	78.91	10.539
ct92-M305Pete-12	120362	25/11/2012	-1	78.902	10.608
ct92-M305Pete-12	120362	25/11/2012	-2	78.903	10.609
ct92-M305Pete-12	120362	25/11/2012	-2	78.914	10.553

ct92-M305Pete-12	120362	25/11/2012	2	78.925	10.585
ct92-M305Pete-12	120362	25/11/2012	1	78.907	10.524
ct92-M305Pete-12	120362	25/11/2012	-1	78.934	10.592
ct92-M305Pete-12	120362	25/11/2012	-1	78.925	10.561
ct92-M305Pete-12	120362	25/11/2012	-1	78.919	10.524
ct92-M305Pete-12	120362	25/11/2012	-2	78.923	10.514
ct92-M305Pete-12	120362	25/11/2012	-2	78.926	10.482
ct92-M305Pete-12	120362	25/11/2012	-2	78.933	10.489
ct92-M305Pete-12	120362	25/11/2012	-2	78.909	10.614
ct92-M305Pete-12	120362	25/11/2012	-2	78.915	10.618
ct92-M305Pete-12	120362	25/11/2012	-2	78.915	10.629
ct92-M305Pete-12	120362	25/11/2012	-2	78.922	10.607
ct92-M305Pete-12	120362	25/11/2012	-2	78.908	10.541
ct92-M305Pete-12	120362	25/11/2012	-2	78.893	10.574
ct92-M305Pete-12	120362	25/11/2012	-2	78.913	10.53
ct92-M305Pete-12	120362	25/11/2012	-2	78.92	10.516
ct92-M305Pete-12	120362	25/11/2012	-1	78.927	10.565
ct92-M305Pete-12	120362	25/11/2012	-2	78.916	10.622
ct92-M305Pete-12	120362	25/11/2012	-2	78.916	10.622
ct92-M305Pete-12	120362	25/11/2012	-2	78.916	10.634
ct92-M305Pete-12	120362	25/11/2012	-2	78.914	10.642
ct92-M305Pete-12	120362	25/11/2012	-2	78.926	10.678
ct92-M305Pete-12	120362	25/11/2012	-2	78.915	10.644
ct92-M305Pete-12	120362	25/11/2012	-2	78.92	10.643
ct92-M305Pete-12	120362	25/11/2012	-2	78.946	10.833
ct92-M305Pete-12	120362	25/11/2012	-2	78.916	10.491
ct92-M305Pete-12	120362	25/11/2012	-1	78.931	10.544
ct92-M305Pete-12	120362	25/11/2012	-2	78.932	10.586
ct92-M305Pete-12	120362	25/11/2012	-2	78.92	10.538
ct92-M305Pete-12	120362	25/11/2012	1	78.923	10.689
ct92-M305Pete-12	120362	25/11/2012	-2	78.923	10.675
ct92-M305Pete-12	120362	26/11/2012	-2	78.933	10.586
ct92-M305Pete-12	120362	26/11/2012	-2	78.938	10.582
ct92-M305Pete-12	120362	26/11/2012	-2	78.931	10.581
ct92-M305Pete-12	120362	26/11/2012	-2	78.938	10.588
ct92-M305Pete-12	120362	26/11/2012	-2	78.935	10.59
ct92-M305Pete-12	120362	26/11/2012	-2	78.945	10.631
ct92-M305Pete-12	120362	26/11/2012	1	78.918	10.62
ct92-M305Pete-12	120362	26/11/2012	-2	78.922	10.618
ct92-M305Pete-12	120362	26/11/2012	-1	78.905	10.668
ct92-M305Pete-12	120362	26/11/2012	-2	78.915	10.609
ct92-M305Pete-12	120362	26/11/2012	-2	78.908	10.663
ct92-M305Pete-12	120362	26/11/2012	-2	78.911	10.668
ct92-M305Pete-12	120362	26/11/2012	-2	78.912	10.66
ct92-M305Pete-12	120362	26/11/2012	-1	78.919	10.577
ct92-M305Pete-12	120362	26/11/2012	-1	78.913	10.562
ct92-M305Pete-12	120362	26/11/2012	-2	78.899	10.69
ct92-M305Pete-12	120362	26/11/2012	-2	78.914	10.562
ct92-M305Pete-12	120362	26/11/2012	-2	78.908	10.542
ct92-M305Pete-12	120362	26/11/2012	-1	78.906	10.55
ct92-M305Pete-12	120362	26/11/2012	-9	80.659	30.64
ct92-M305Pete-12	120362	26/11/2012	-1	78.894	10.573
ct92-M305Pete-12	120362	26/11/2012	-2	78.894	10.576
ct92-M305Pete-12	120362	26/11/2012	-2	78.924	10.512
ct92-M305Pete-12	120362	26/11/2012	-2	78.922	10.503
ct92-M305Pete-12	120362	26/11/2012	-2	78.921	10.507
ct92-M305Pete-12	120362	26/11/2012	-2	78.911	10.438
ct92-M305Pete-12	120362	26/11/2012	0	78.923	10.518
ct92-M305Pete-12	120362	26/11/2012	-2	78.909	10.471
ct92-M305Pete-12	120362	26/11/2012	-2	78.925	10.525
ct92-M305Pete-12	120362	26/11/2012	-1	78.93	10.432
ct92-M305Pete-12	120362	26/11/2012	-2	78.932	10.453
ct92-M305Pete-12	120362	26/11/2012	-1	78.92	10.379
ct92-M305Pete-12	120362	26/11/2012	-2	78.916	10.492
ct92-M305Pete-12	120362	26/11/2012	-2	78.912	10.471
ct92-M305Pete-12	120362	26/11/2012	-2	78.941	10.364
ct92-M305Pete-12	120362	26/11/2012	-2	78.934	10.335
ct92-M305Pete-12	120362	26/11/2012	-2	78.933	10.339

ct92-M305Pete-12	120362	26/11/2012	-1	78.934	10.44
ct92-M305Pete-12	120362	26/11/2012	-2	78.918	10.496
ct92-M305Pete-12	120362	26/11/2012	-2	78.935	10.364
ct92-M305Pete-12	120362	26/11/2012	-2	78.936	10.409
ct92-M305Pete-12	120362	26/11/2012	-2	78.928	10.35
ct92-M305Pete-12	120362	26/11/2012	-1	78.929	10.452
ct92-M305Pete-12	120362	26/11/2012	-2	78.937	10.541
ct92-M305Pete-12	120362	26/11/2012	-1	78.935	10.482
ct92-M305Pete-12	120362	26/11/2012	-1	78.933	10.515
ct92-M305Pete-12	120362	26/11/2012	-2	78.925	10.539
ct92-M305Pete-12	120362	26/11/2012	-2	78.927	10.513
ct92-M305Pete-12	120362	26/11/2012	-2	78.927	10.517
ct92-M305Pete-12	120362	26/11/2012	0	78.934	10.519
ct92-M305Pete-12	120362	26/11/2012	2	78.95	10.535
ct92-M305Pete-12	120362	26/11/2012	-1	78.948	10.521
ct92-M305Pete-12	120362	26/11/2012	-2	78.948	10.509
ct92-M305Pete-12	120362	26/11/2012	-2	78.947	10.514
ct92-M305Pete-12	120362	26/11/2012	-2	78.946	10.52
ct92-M305Pete-12	120362	26/11/2012	-1	78.961	10.525
ct92-M305Pete-12	120362	26/11/2012	-2	78.961	10.534
ct92-M305Pete-12	120362	26/11/2012	-2	78.958	10.535
ct92-M305Pete-12	120362	26/11/2012	-2	78.957	10.548
ct92-M305Pete-12	120362	26/11/2012	-2	78.958	10.555
ct92-M305Pete-12	120362	26/11/2012	-2	78.957	10.564
ct92-M305Pete-12	120362	27/11/2012	-2	78.943	10.595
ct92-M305Pete-12	120362	27/11/2012	-2	78.941	10.598
ct92-M305Pete-12	120362	27/11/2012	-1	78.949	10.607
ct92-M305Pete-12	120362	27/11/2012	-1	78.943	10.601
ct92-M305Pete-12	120362	27/11/2012	-2	78.943	10.602
ct92-M305Pete-12	120362	27/11/2012	-2	78.942	10.651
ct92-M305Pete-12	120362	27/11/2012	-1	78.942	10.576
ct92-M305Pete-12	120362	27/11/2012	-1	78.95	10.613
ct92-M305Pete-12	120362	27/11/2012	-2	78.936	10.599
ct92-M305Pete-12	120362	27/11/2012	-2	78.943	10.643
ct92-M305Pete-12	120362	27/11/2012	-2	78.932	10.573
ct92-M305Pete-12	120362	27/11/2012	-2	78.938	10.615
ct92-M305Pete-12	120362	27/11/2012	-2	78.93	10.63
ct92-M305Pete-12	120362	27/11/2012	-1	78.932	10.604
ct92-M305Pete-12	120362	27/11/2012	-2	78.94	10.611
ct92-M305Pete-12	120362	27/11/2012	-2	78.933	10.607
ct92-M305Pete-12	120362	27/11/2012	-2	78.931	10.591
ct92-M305Pete-12	120362	27/11/2012	-1	78.928	10.56
ct92-M305Pete-12	120362	27/11/2012	-2	78.927	10.57
ct92-M305Pete-12	120362	27/11/2012	-2	78.926	10.572
ct92-M305Pete-12	120362	27/11/2012	1	78.928	10.581
ct92-M305Pete-12	120362	27/11/2012	-1	78.928	10.568
ct92-M305Pete-12	120362	27/11/2012	0	78.925	10.547
ct92-M305Pete-12	120362	27/11/2012	-1	78.933	10.493
ct92-M305Pete-12	120362	27/11/2012	-1	78.933	10.531
ct92-M305Pete-12	120362	27/11/2012	-1	78.935	10.509
ct92-M305Pete-12	120362	27/11/2012	1	78.949	10.504
ct92-M305Pete-12	120362	27/11/2012	-1	78.938	10.44
ct92-M305Pete-12	120362	27/11/2012	-2	78.935	10.442
ct92-M305Pete-12	120362	27/11/2012	1	78.936	10.434
ct92-M305Pete-12	120362	27/11/2012	-2	78.932	10.421
ct92-M305Pete-12	120362	27/11/2012	-2	78.931	10.417
ct92-M305Pete-12	120362	27/11/2012	-2	78.914	10.463
ct92-M305Pete-12	120362	27/11/2012	-2	78.913	10.501
ct92-M305Pete-12	120362	27/11/2012	-2	78.911	10.436
ct92-M305Pete-12	120362	27/11/2012	1	78.934	10.425
ct92-M305Pete-12	120362	27/11/2012	-2	78.938	10.445
ct92-M305Pete-12	120362	27/11/2012	-2	78.938	10.463
ct92-M305Pete-12	120362	27/11/2012	-2	78.937	10.462
ct92-M305Pete-12	120362	27/11/2012	0	78.934	10.566
ct92-M305Pete-12	120362	27/11/2012	-2	78.932	10.443
ct92-M305Pete-12	120362	27/11/2012	0	78.934	10.5
ct92-M305Pete-12	120362	27/11/2012	-2	78.946	10.506
ct92-M305Pete-12	120362	27/11/2012	-2	78.946	10.395

ct92-M305Pete-12	120362	27/11/2012	-2	78.949	10.451
ct92-M305Pete-12	120362	27/11/2012	-2	78.946	10.454
ct92-M305Pete-12	120362	27/11/2012	-2	78.948	10.481
ct92-M305Pete-12	120362	27/11/2012	-2	78.937	10.382
ct92-M305Pete-12	120362	27/11/2012	1	78.926	10.402
ct92-M305Pete-12	120362	27/11/2012	-2	78.931	10.398
ct92-M305Pete-12	120362	27/11/2012	-2	78.937	10.41
ct92-M305Pete-12	120362	27/11/2012	-2	78.938	10.4
ct92-M305Pete-12	120362	27/11/2012	-2	78.935	10.414
ct92-M305Pete-12	120362	27/11/2012	-2	78.939	10.369
ct92-M305Pete-12	120362	27/11/2012	-2	78.937	10.38
ct92-M305Pete-12	120362	27/11/2012	-1	78.926	10.31
ct92-M305Pete-12	120362	27/11/2012	-2	78.939	10.381
ct92-M305Pete-12	120362	27/11/2012	-2	78.938	10.373
ct92-M305Pete-12	120362	27/11/2012	-2	78.943	10.267
ct92-M305Pete-12	120362	27/11/2012	-2	78.943	10.251
ct92-M305Pete-12	120362	27/11/2012	-2	78.941	10.332
ct92-M305Pete-12	120362	27/11/2012	-1	78.922	10.3
ct92-M305Pete-12	120362	27/11/2012	-1	78.927	10.299
ct92-M305Pete-12	120362	27/11/2012	-2	78.925	10.229
ct92-M305Pete-12	120362	27/11/2012	-2	78.928	10.235
ct92-M305Pete-12	120362	27/11/2012	-2	78.928	10.24
ct92-M305Pete-12	120362	27/11/2012	-2	78.93	10.243
ct92-M305Pete-12	120362	27/11/2012	-2	78.937	10.241
ct92-M305Pete-12	120362	27/11/2012	-2	78.947	10.287
ct92-M305Pete-12	120362	28/11/2012	-2	78.942	10.282
ct92-M305Pete-12	120362	28/11/2012	-2	78.947	10.32
ct92-M305Pete-12	120362	28/11/2012	-2	78.956	10.269
ct92-M305Pete-12	120362	28/11/2012	-2	78.915	10.484
ct92-M305Pete-12	120362	28/11/2012	-1	78.933	10.695
ct92-M305Pete-12	120362	28/11/2012	-2	78.936	10.759
ct92-M305Pete-12	120362	28/11/2012	-2	78.928	10.875
ct92-M305Pete-12	120362	28/11/2012	-2	78.921	10.966
ct92-M305Pete-12	120362	28/11/2012	-2	78.934	10.644
ct92-M305Pete-12	120362	28/11/2012	-2	78.929	10.772
ct92-M305Pete-12	120362	28/11/2012	-1	78.947	10.638
ct92-M305Pete-12	120362	28/11/2012	-2	78.955	10.6
ct92-M305Pete-12	120362	28/11/2012	-2	78.957	10.606
ct92-M305Pete-12	120362	28/11/2012	1	78.954	10.656
ct92-M305Pete-12	120362	28/11/2012	0	78.946	10.642
ct92-M305Pete-12	120362	28/11/2012	-2	78.937	10.776
ct92-M305Pete-12	120362	28/11/2012	-1	78.953	10.655
ct92-M305Pete-12	120362	28/11/2012	-2	78.953	10.707
ct92-M305Pete-12	120362	28/11/2012	-2	78.954	10.695
ct92-M305Pete-12	120362	28/11/2012	-2	78.948	10.681
ct92-M305Pete-12	120362	28/11/2012	-2	78.951	10.66
ct92-M305Pete-12	120362	28/11/2012	-1	78.942	10.606
ct92-M305Pete-12	120362	28/11/2012	-2	78.947	10.602
ct92-M305Pete-12	120362	28/11/2012	-2	78.947	10.615
ct92-M305Pete-12	120362	28/11/2012	-2	78.94	10.613
ct92-M305Pete-12	120362	28/11/2012	-2	78.944	10.581
ct92-M305Pete-12	120362	28/11/2012	-2	78.941	10.672
ct92-M305Pete-12	120362	28/11/2012	-2	78.952	10.661
ct92-M305Pete-12	120362	28/11/2012	-2	78.945	10.683
ct92-M305Pete-12	120362	28/11/2012	-2	78.94	10.65
ct92-M305Pete-12	120362	28/11/2012	-2	78.947	10.595
ct92-M305Pete-12	120362	28/11/2012	-2	78.936	10.55
ct92-M305Pete-12	120362	28/11/2012	-1	78.939	10.514
ct92-M305Pete-12	120362	28/11/2012	-2	78.931	10.482
ct92-M305Pete-12	120362	28/11/2012	-2	78.932	10.439
ct92-M305Pete-12	120362	28/11/2012	-2	78.932	10.44
ct92-M305Pete-12	120362	28/11/2012	-2	78.922	10.515
ct92-M305Pete-12	120362	28/11/2012	1	78.944	10.402
ct92-M305Pete-12	120362	28/11/2012	-2	78.944	10.397
ct92-M305Pete-12	120362	28/11/2012	-2	78.942	10.411
ct92-M305Pete-12	120362	28/11/2012	-2	78.936	10.328
ct92-M305Pete-12	120362	28/11/2012	-2	78.932	10.348
ct92-M305Pete-12	120362	28/11/2012	-2	78.865	10.592

ct92-M305Pete-12	120362	28/11/2012	-2	78.902	10.513
ct92-M305Pete-12	120362	28/11/2012	-2	78.916	10.449
ct92-M305Pete-12	120362	28/11/2012	-2	78.925	10.427
ct92-M305Pete-12	120362	28/11/2012	-2	78.923	10.446
ct92-M305Pete-12	120362	28/11/2012	-2	78.924	10.446
ct92-M305Pete-12	120362	28/11/2012	-2	78.921	10.517
ct92-M305Pete-12	120362	28/11/2012	-2	78.922	10.52
ct92-M305Pete-12	120362	28/11/2012	-2	78.926	10.555
ct92-M305Pete-12	120362	28/11/2012	-2	78.929	10.545
ct92-M305Pete-12	120362	28/11/2012	-2	78.93	10.557
ct92-M305Pete-12	120362	28/11/2012	-1	78.93	10.555
ct92-M305Pete-12	120362	29/11/2012	-2	78.936	10.575
ct92-M305Pete-12	120362	29/11/2012	-2	78.939	10.575
ct92-M305Pete-12	120362	29/11/2012	-2	78.936	10.561
ct92-M305Pete-12	120362	29/11/2012	-1	78.931	10.618
ct92-M305Pete-12	120362	29/11/2012	-1	78.936	10.607
ct92-M305Pete-12	120362	29/11/2012	-2	78.936	10.609
ct92-M305Pete-12	120362	29/11/2012	-2	78.938	10.567
ct92-M305Pete-12	120362	29/11/2012	-1	78.949	10.621
ct92-M305Pete-12	120362	29/11/2012	-2	78.937	10.567
ct92-M305Pete-12	120362	29/11/2012	-2	78.944	10.607
ct92-M305Pete-12	120362	29/11/2012	-2	78.94	10.573
ct92-M305Pete-12	120362	29/11/2012	-1	78.923	10.5
ct92-M305Pete-12	120362	29/11/2012	-1	78.923	10.557
ct92-M305Pete-12	120362	29/11/2012	-2	78.925	10.553
ct92-M305Pete-12	120362	29/11/2012	-2	78.924	10.547
ct92-M305Pete-12	120362	29/11/2012	-2	78.917	10.51
ct92-M305Pete-12	120362	29/11/2012	-2	78.918	10.511
ct92-M305Pete-12	120362	29/11/2012	-2	78.92	10.501
ct92-M305Pete-12	120362	29/11/2012	-1	78.928	10.469
ct92-M305Pete-12	120362	29/11/2012	-1	78.922	10.501
ct92-M305Pete-12	120362	29/11/2012	-2	78.922	10.488
ct92-M305Pete-12	120362	29/11/2012	-2	78.918	10.483
ct92-M305Pete-12	120362	29/11/2012	-2	78.912	10.478
ct92-M305Pete-12	120362	29/11/2012	-2	78.921	10.45
ct92-M305Pete-12	120362	29/11/2012	-2	78.914	10.477
ct92-M305Pete-12	120362	29/11/2012	-2	78.917	10.473
ct92-M305Pete-12	120362	29/11/2012	-2	78.924	10.424
ct92-M305Pete-12	120362	29/11/2012	-2	78.924	10.453
ct92-M305Pete-12	120362	29/11/2012	1	78.924	10.452
ct92-M305Pete-12	120362	29/11/2012	-2	78.924	10.441
ct92-M305Pete-12	120362	29/11/2012	-1	78.93	10.425
ct92-M305Pete-12	120362	29/11/2012	-2	78.929	10.476
ct92-M305Pete-12	120362	29/11/2012	-1	78.935	10.424
ct92-M305Pete-12	120362	29/11/2012	-1	78.934	10.398
ct92-M305Pete-12	120362	29/11/2012	-2	78.936	10.38
ct92-M305Pete-12	120362	29/11/2012	-2	78.935	10.389
ct92-M305Pete-12	120362	29/11/2012	-2	78.94	10.432
ct92-M305Pete-12	120362	29/11/2012	-2	78.937	10.408
ct92-M305Pete-12	120362	29/11/2012	-2	78.934	10.42
ct92-M305Pete-12	120362	29/11/2012	-2	78.937	10.405
ct92-M305Pete-12	120362	29/11/2012	-1	78.934	10.409
ct92-M305Pete-12	120362	29/11/2012	-2	78.936	10.392
ct92-M305Pete-12	120362	29/11/2012	-2	78.937	10.405
ct92-M305Pete-12	120362	29/11/2012	-2	78.937	10.402
ct92-M305Pete-12	120362	29/11/2012	-2	78.936	10.407
ct92-M305Pete-12	120362	29/11/2012	-2	78.935	10.405
ct92-M305Pete-12	120362	29/11/2012	-1	78.935	10.385
ct92-M305Pete-12	120362	29/11/2012	-2	78.934	10.398
ct92-M305Pete-12	120362	29/11/2012	-2	78.94	10.3
ct92-M305Pete-12	120362	29/11/2012	-2	78.938	10.347
ct92-M305Pete-12	120362	29/11/2012	-2	78.937	10.349
ct92-M305Pete-12	120362	30/11/2012	-1	78.939	10.42
ct92-M305Pete-12	120362	30/11/2012	-2	78.94	10.349
ct92-M305Pete-12	120362	30/11/2012	-2	78.942	10.36
ct92-M305Pete-12	120362	30/11/2012	-2	78.943	10.482
ct92-M305Pete-12	120362	30/11/2012	-2	78.942	10.465
ct92-M305Pete-12	120362	30/11/2012	-2	78.94	10.446

ct92-M305Pete-12	120362	30/11/2012	-2	78.938	10.461
ct92-M305Pete-12	120362	30/11/2012	-2	78.94	10.448
ct92-M305Pete-12	120362	30/11/2012	-1	78.932	10.523
ct92-M305Pete-12	120362	30/11/2012	-2	78.932	10.524
ct92-M305Pete-12	120362	30/11/2012	-1	78.928	10.492
ct92-M305Pete-12	120362	30/11/2012	-2	78.93	10.499
ct92-M305Pete-12	120362	30/11/2012	-2	78.935	10.546
ct92-M305Pete-12	120362	30/11/2012	-2	78.933	10.517
ct92-M305Pete-12	120362	30/11/2012	1	78.929	10.546
ct92-M305Pete-12	120362	30/11/2012	-1	78.933	10.544
ct92-M305Pete-12	120362	30/11/2012	-1	78.93	10.541
ct92-M305Pete-12	120362	30/11/2012	-1	78.934	10.508
ct92-M305Pete-12	120362	30/11/2012	1	78.936	10.482
ct92-M305Pete-12	120362	30/11/2012	-2	78.937	10.482
ct92-M305Pete-12	120362	30/11/2012	-2	78.938	10.49
ct92-M305Pete-12	120362	30/11/2012	-2	78.934	10.527
ct92-M305Pete-12	120362	30/11/2012	-2	78.944	10.429
ct92-M305Pete-12	120362	30/11/2012	0	78.936	10.518
ct92-M305Pete-12	120362	30/11/2012	-1	78.932	10.512
ct92-M305Pete-12	120362	30/11/2012	-1	78.926	10.503
ct92-M305Pete-12	120362	30/11/2012	-2	78.933	10.518
ct92-M305Pete-12	120362	30/11/2012	-1	78.933	10.47
ct92-M305Pete-12	120362	30/11/2012	-1	78.926	10.422
ct92-M305Pete-12	120362	30/11/2012	-2	78.938	10.474
ct92-M305Pete-12	120362	30/11/2012	-2	78.936	10.424
ct92-M305Pete-12	120362	30/11/2012	-2	78.927	10.45
ct92-M305Pete-12	120362	30/11/2012	1	78.949	10.488
ct92-M305Pete-12	120362	30/11/2012	-1	78.934	10.513
ct92-M305Pete-12	120362	30/11/2012	-2	78.935	10.534
ct92-M305Pete-12	120362	30/11/2012	1	78.932	10.505
ct92-M305Pete-12	120362	30/11/2012	-1	78.935	10.509
ct92-M305Pete-12	120362	30/11/2012	-1	78.937	10.498
ct92-M305Pete-12	120362	30/11/2012	0	78.931	10.495
ct92-M305Pete-12	120362	30/11/2012	-2	78.936	10.483
ct92-M305Pete-12	120362	30/11/2012	-2	78.925	10.495
ct92-M305Pete-12	120362	30/11/2012	-2	78.918	10.502
ct92-M305Pete-12	120362	30/11/2012	0	78.919	10.438
ct92-M305Pete-12	120362	30/11/2012	-2	78.917	10.435
ct92-M305Pete-12	120362	30/11/2012	-1	78.917	10.406
ct92-M305Pete-12	120362	30/11/2012	-2	78.92	10.388
ct92-M305Pete-12	120362	30/11/2012	-1	78.932	10.364
ct92-M305Pete-12	120362	30/11/2012	-2	78.933	10.361
ct92-M305Pete-12	120362	30/11/2012	0	78.949	10.429
ct92-M305Pete-12	120362	30/11/2012	-1	78.939	10.438
ct92-M305Pete-12	120362	30/11/2012	-2	78.938	10.448
ct92-M305Pete-12	120362	30/11/2012	-2	78.933	10.388
ct92-M305Pete-12	120362	30/11/2012	-1	78.958	10.377
ct92-M305Pete-12	120362	30/11/2012	-1	78.951	10.251
ct92-M305Pete-12	120362	30/11/2012	-2	78.961	10.34
ct92-M305Pete-12	120362	30/11/2012	-1	78.925	10.295
ct92-M305Pete-12	120362	30/11/2012	-2	78.925	10.287
ct92-M305Pete-12	120362	30/11/2012	-2	78.934	10.286
ct92-M305Pete-12	120362	30/11/2012	-1	78.929	10.355
ct92-M305Pete-12	120362	30/11/2012	-2	78.926	10.349
ct92-M305Pete-12	120362	30/11/2012	1	78.928	10.365
ct92-M305Pete-12	120362	30/11/2012	-2	78.93	10.34
ct92-M305Pete-12	120362	30/11/2012	-2	78.91	10.344
ct92-M305Pete-12	120362	30/11/2012	-1	78.913	10.42
ct92-M305Pete-12	120362	30/11/2012	1	78.945	10.397
ct92-M305Pete-12	120362	30/11/2012	-1	78.951	10.448
ct92-M305Pete-12	120362	30/11/2012	-1	78.924	10.372
ct92-M305Pete-12	120362	30/11/2012	-2	78.926	10.373
ct92-M305Pete-12	120362	30/11/2012	-1	78.917	10.382
ct92-M305Pete-12	120362	30/11/2012	-1	78.919	10.357
ct92-M305Pete-12	120362	30/11/2012	2	78.917	10.353
ct92-M305Pete-12	120362	30/11/2012	-2	78.917	10.353
ct92-M305Pete-12	120362	30/11/2012	-1	78.916	10.392
ct92-M305Pete-12	120362	01/12/2012	-2	78.924	10.382

ct92-M305Pete-12	120362	01/12/2012	-2	78.933	10.537
ct92-M305Pete-12	120362	01/12/2012	-2	78.934	10.589
ct92-M305Pete-12	120362	01/12/2012	-2	78.935	10.613
ct92-M305Pete-12	120362	01/12/2012	-2	78.929	10.465
ct92-M305Pete-12	120362	01/12/2012	-2	78.932	10.506
ct92-M305Pete-12	120362	01/12/2012	-2	78.932	10.499
ct92-M305Pete-12	120362	01/12/2012	-2	78.915	10.525
ct92-M305Pete-12	120362	01/12/2012	-1	78.926	10.431
ct92-M305Pete-12	120362	01/12/2012	-2	78.928	10.465
ct92-M305Pete-12	120362	01/12/2012	-1	78.923	10.502
ct92-M305Pete-12	120362	01/12/2012	-1	78.919	10.314
ct92-M305Pete-12	120362	01/12/2012	-2	78.919	10.359
ct92-M305Pete-12	120362	01/12/2012	-2	78.918	10.355
ct92-M305Pete-12	120362	01/12/2012	0	78.914	10.314
ct92-M305Pete-12	120362	01/12/2012	-2	78.918	10.323
ct92-M305Pete-12	120362	01/12/2012	-1	78.917	10.31
ct92-M305Pete-12	120362	01/12/2012	1	78.927	10.319
ct92-M305Pete-12	120362	01/12/2012	-2	78.92	10.421
ct92-M305Pete-12	120362	01/12/2012	-2	78.921	10.296
ct92-M305Pete-12	120362	01/12/2012	-1	78.923	10.297
ct92-M305Pete-12	120362	01/12/2012	-2	78.922	10.296
ct92-M305Pete-12	120362	01/12/2012	-1	78.931	10.32
ct92-M305Pete-12	120362	01/12/2012	-2	78.929	10.314
ct92-M305Pete-12	120362	01/12/2012	-2	78.922	10.303
ct92-M305Pete-12	120362	01/12/2012	-2	78.921	10.299
ct92-M305Pete-12	120362	01/12/2012	-2	78.921	10.307
ct92-M305Pete-12	120362	01/12/2012	-2	78.913	10.345
ct92-M305Pete-12	120362	01/12/2012	-2	78.934	10.338
ct92-M305Pete-12	120362	01/12/2012	-2	78.945	10.315
ct92-M305Pete-12	120362	01/12/2012	-2	78.94	10.251
ct92-M305Pete-12	120362	01/12/2012	-2	78.904	10.316
ct92-M305Pete-12	120362	01/12/2012	-2	78.918	10.326
ct92-M305Pete-12	120362	01/12/2012	-2	78.919	10.351
ct92-M305Pete-12	120362	01/12/2012	-2	78.907	10.291
ct92-M305Pete-12	120362	01/12/2012	-2	78.908	10.24
ct92-M305Pete-12	120362	01/12/2012	-2	78.919	10.256
ct92-M305Pete-12	120362	01/12/2012	-2	78.921	10.326
ct92-M305Pete-12	120362	01/12/2012	-2	78.921	10.324
ct92-M305Pete-12	120362	01/12/2012	-2	78.921	10.32
ct92-M305Pete-12	120362	01/12/2012	-2	78.938	10.381
ct92-M305Pete-12	120362	01/12/2012	-2	78.937	10.365
ct92-M305Pete-12	120362	01/12/2012	-2	78.93	10.386
ct92-M305Pete-12	120362	01/12/2012	-2	78.927	10.384
ct92-M305Pete-12	120362	01/12/2012	-2	78.913	10.371
ct92-M305Pete-12	120362	01/12/2012	-2	78.92	10.44
ct92-M305Pete-12	120362	01/12/2012	-2	78.927	10.373
ct92-M305Pete-12	120362	01/12/2012	-1	78.925	10.401
ct92-M305Pete-12	120362	01/12/2012	-2	78.91	10.4
ct92-M305Pete-12	120362	01/12/2012	-2	78.911	10.404
ct92-M305Pete-12	120362	02/12/2012	-1	78.925	10.272
ct92-M305Pete-12	120362	02/12/2012	-1	78.945	10.402
ct92-M305Pete-12	120362	02/12/2012	-2	78.926	10.213
ct92-M305Pete-12	120362	02/12/2012	-1	78.954	10.381
ct92-M305Pete-12	120362	02/12/2012	-2	78.952	10.377
ct92-M305Pete-12	120362	02/12/2012	-2	78.95	10.337
ct92-M305Pete-12	120362	02/12/2012	-2	78.95	10.353
ct92-M305Pete-12	120362	02/12/2012	-2	78.95	10.319
ct92-M305Pete-12	120362	02/12/2012	-2	78.955	10.375
ct92-M305Pete-12	120362	02/12/2012	-2	78.956	10.355
ct92-M305Pete-12	120362	02/12/2012	-1	78.95	10.386
ct92-M305Pete-12	120362	02/12/2012	-2	78.952	10.427
ct92-M305Pete-12	120362	02/12/2012	-2	78.951	10.44
ct92-M305Pete-12	120362	02/12/2012	-2	78.95	10.447
ct92-M305Pete-12	120362	02/12/2012	-2	78.952	10.439
ct92-M305Pete-12	120362	02/12/2012	-2	78.934	10.531
ct92-M305Pete-12	120362	02/12/2012	-2	78.961	10.562
ct92-M305Pete-12	120362	02/12/2012	-2	78.954	10.448
ct92-M305Pete-12	120362	02/12/2012	-2	78.924	10.527

ct92-M305Pete-12	120362	02/12/2012	-2	78.915	10.533
ct92-M305Pete-12	120362	02/12/2012	-1	78.92	10.518
ct92-M305Pete-12	120362	02/12/2012	-2	78.932	10.521
ct92-M305Pete-12	120362	02/12/2012	-1	78.922	10.531
ct92-M305Pete-12	120362	02/12/2012	-2	78.925	10.518
ct92-M305Pete-12	120362	02/12/2012	-2	78.932	10.508
ct92-M305Pete-12	120362	02/12/2012	-2	78.932	10.514
ct92-M305Pete-12	120362	02/12/2012	-2	78.932	10.53
ct92-M305Pete-12	120362	02/12/2012	-2	78.942	10.572
ct92-M305Pete-12	120362	02/12/2012	-1	78.928	10.6
ct92-M305Pete-12	120362	02/12/2012	-2	78.929	10.584
ct92-M305Pete-12	120362	02/12/2012	-2	78.921	10.55
ct92-M305Pete-12	120362	02/12/2012	-1	78.931	10.518
ct92-M305Pete-12	120362	02/12/2012	-2	78.92	10.589
ct92-M305Pete-12	120362	02/12/2012	-2	78.92	10.608
ct92-M305Pete-12	120362	02/12/2012	-2	78.92	10.607
ct92-M305Pete-12	120362	02/12/2012	-2	78.921	10.605
ct92-M305Pete-12	120362	02/12/2012	-2	78.92	10.581
ct92-M305Pete-12	120362	02/12/2012	1	78.913	10.623
ct92-M305Pete-12	120362	02/12/2012	-2	78.922	10.617
ct92-M305Pete-12	120362	02/12/2012	-2	78.93	10.576
ct92-M305Pete-12	120362	02/12/2012	-2	78.926	10.564
ct92-M305Pete-12	120362	02/12/2012	-2	78.925	10.566
ct92-M305Pete-12	120362	02/12/2012	-1	79.042	10.231
ct92-M305Pete-12	120362	02/12/2012	-2	78.91	10.613
ct92-M305Pete-12	120362	02/12/2012	-2	78.975	10.362
ct92-M305Pete-12	120362	02/12/2012	-2	78.96	10.448
ct92-M305Pete-12	120362	02/12/2012	-2	78.948	10.511
ct92-M305Pete-12	120362	02/12/2012	-2	78.944	10.536
ct92-M305Pete-12	120362	02/12/2012	-2	78.921	10.599
ct92-M305Pete-12	120362	02/12/2012	-2	78.931	10.581
ct92-M305Pete-12	120362	02/12/2012	-1	78.924	10.567
ct92-M305Pete-12	120362	02/12/2012	2	78.92	10.527
ct92-M305Pete-12	120362	02/12/2012	-2	78.92	10.527
ct92-M305Pete-12	120362	02/12/2012	-2	78.918	10.489
ct92-M305Pete-12	120362	02/12/2012	-1	78.917	10.433
ct92-M305Pete-12	120362	02/12/2012	0	78.939	10.429
ct92-M305Pete-12	120362	02/12/2012	0	78.912	10.488
ct92-M305Pete-12	120362	02/12/2012	-2	78.912	10.51
ct92-M305Pete-12	120362	02/12/2012	-2	78.907	10.506
ct92-M305Pete-12	120362	02/12/2012	-1	78.915	10.458
ct92-M305Pete-12	120362	02/12/2012	-2	78.93	10.459
ct92-M305Pete-12	120362	02/12/2012	-2	78.927	10.437
ct92-M305Pete-12	120362	02/12/2012	-2	78.931	10.451
ct92-M305Pete-12	120362	02/12/2012	-2	78.925	10.419
ct92-M305Pete-12	120362	02/12/2012	-1	78.916	10.451
ct92-M305Pete-12	120362	02/12/2012	-2	78.917	10.456
ct92-M305Pete-12	120362	02/12/2012	-2	78.918	10.402
ct92-M305Pete-12	120362	02/12/2012	-2	78.92	10.522
ct92-M305Pete-12	120362	02/12/2012	-2	78.924	10.441
ct92-M305Pete-12	120362	02/12/2012	-2	78.923	10.438
ct92-M305Pete-12	120362	02/12/2012	-2	78.923	10.441
ct92-M305Pete-12	120362	02/12/2012	-2	78.93	10.449
ct92-M305Pete-12	120362	02/12/2012	-2	78.921	10.484
ct92-M305Pete-12	120362	02/12/2012	-2	78.914	10.377
ct92-M305Pete-12	120362	02/12/2012	-2	78.92	10.462
ct92-M305Pete-12	120362	02/12/2012	-2	78.919	10.46
ct92-M305Pete-12	120362	02/12/2012	-2	78.904	10.449
ct92-M305Pete-12	120362	02/12/2012	-1	78.917	10.421
ct92-M305Pete-12	120362	02/12/2012	-1	78.916	10.479
ct92-M305Pete-12	120362	02/12/2012	-2	78.916	10.459
ct92-M305Pete-12	120362	02/12/2012	-2	78.924	10.515
ct92-M305Pete-12	120362	03/12/2012	-2	78.924	10.497
ct92-M305Pete-12	120362	03/12/2012	-2	78.927	10.492
ct92-M305Pete-12	120362	03/12/2012	-2	78.922	10.502
ct92-M305Pete-12	120362	03/12/2012	-2	78.922	10.502
ct92-M305Pete-12	120362	03/12/2012	-2	78.925	10.511
ct92-M305Pete-12	120362	03/12/2012	-1	78.916	10.518

ct92-M305Pete-12	120362	03/12/2012	-2	78.914	10.65
ct92-M305Pete-12	120362	03/12/2012	-2	78.91	10.662
ct92-M305Pete-12	120362	03/12/2012	-2	78.913	10.659
ct92-M305Pete-12	120362	03/12/2012	-2	78.917	10.684
ct92-M305Pete-12	120362	03/12/2012	-2	78.92	10.657
ct92-M305Pete-12	120362	03/12/2012	-2	78.918	10.689
ct92-M305Pete-12	120362	03/12/2012	-2	78.921	10.728
ct92-M305Pete-12	120362	03/12/2012	-2	78.922	10.724
ct92-M305Pete-12	120362	03/12/2012	-2	78.925	10.714
ct92-M305Pete-12	120362	03/12/2012	1	78.929	10.721
ct92-M305Pete-12	120362	03/12/2012	-2	78.923	10.716
ct92-M305Pete-12	120362	03/12/2012	-2	78.929	10.752
ct92-M305Pete-12	120362	03/12/2012	-2	78.928	10.771
ct92-M305Pete-12	120362	03/12/2012	-2	78.934	10.702
ct92-M305Pete-12	120362	03/12/2012	-2	78.924	10.782
ct92-M305Pete-12	120362	03/12/2012	-1	78.93	10.709
ct92-M305Pete-12	120362	03/12/2012	-2	78.92	10.701
ct92-M305Pete-12	120362	03/12/2012	-2	78.919	10.734
ct92-M305Pete-12	120362	03/12/2012	-2	78.913	10.768
ct92-M305Pete-12	120362	03/12/2012	-2	78.923	10.746
ct92-M305Pete-12	120362	03/12/2012	-2	78.917	10.748
ct92-M305Pete-12	120362	03/12/2012	-2	78.907	10.763
ct92-M305Pete-12	120362	03/12/2012	0	78.908	10.701
ct92-M305Pete-12	120362	03/12/2012	-2	78.915	10.692
ct92-M305Pete-12	120362	03/12/2012	-1	78.912	10.681
ct92-M305Pete-12	120362	03/12/2012	-2	78.913	10.728
ct92-M305Pete-12	120362	03/12/2012	-2	78.912	10.735
ct92-M305Pete-12	120362	03/12/2012	-2	78.898	10.718
ct92-M305Pete-12	120362	03/12/2012	-2	78.902	10.734
ct92-M305Pete-12	120362	03/12/2012	-2	78.894	10.74
ct92-M305Pete-12	120362	03/12/2012	-2	78.904	10.762
ct92-M305Pete-12	120362	03/12/2012	-2	78.905	10.748
ct92-M305Pete-12	120362	03/12/2012	-2	78.894	10.691
ct92-M305Pete-12	120362	03/12/2012	-2	78.905	10.64
ct92-M305Pete-12	120362	03/12/2012	-2	78.893	10.745
ct92-M305Pete-12	120362	03/12/2012	-2	78.904	10.715
ct92-M305Pete-12	120362	03/12/2012	-2	78.906	10.716
ct92-M305Pete-12	120362	03/12/2012	-2	78.91	10.706
ct92-M305Pete-12	120362	03/12/2012	-1	78.889	10.728
ct92-M305Pete-12	120362	03/12/2012	-2	78.904	10.743
ct92-M305Pete-12	120362	03/12/2012	0	78.895	10.744
ct92-M305Pete-12	120362	03/12/2012	-1	78.901	10.716
ct92-M305Pete-12	120362	03/12/2012	-2	78.904	10.77
ct92-M305Pete-12	120362	03/12/2012	-2	78.904	10.758
ct92-M305Pete-12	120362	03/12/2012	-2	78.902	10.755
ct92-M305Pete-12	120362	03/12/2012	-2	78.91	10.685
ct92-M305Pete-12	120362	03/12/2012	-2	78.91	10.685
ct92-M305Pete-12	120362	03/12/2012	-2	78.91	10.685
ct92-M305Pete-12	120362	03/12/2012	-2	78.909	10.7
ct92-M305Pete-12	120362	03/12/2012	-2	78.909	10.694
ct92-M305Pete-12	120362	03/12/2012	-2	78.921	10.716
ct92-M305Pete-12	120362	03/12/2012	-2	78.92	10.709
ct92-M305Pete-12	120362	03/12/2012	-2	78.888	10.677
ct92-M305Pete-12	120362	03/12/2012	-2	78.89	10.654
ct92-M305Pete-12	120362	03/12/2012	-2	78.906	10.644
ct92-M305Pete-12	120362	03/12/2012	-2	78.907	10.639
ct92-M305Pete-12	120362	03/12/2012	-2	78.913	10.657
ct92-M305Pete-12	120362	03/12/2012	-2	78.91	10.688
ct92-M305Pete-12	120362	03/12/2012	-2	78.91	10.689
ct92-M305Pete-12	120362	03/12/2012	-2	78.911	10.672
ct92-M305Pete-12	120362	03/12/2012	-2	78.911	10.676
ct92-M305Pete-12	120362	03/12/2012	-2	78.914	10.661
ct92-M305Pete-12	120362	03/12/2012	-2	78.922	10.724
ct92-M305Pete-12	120362	03/12/2012	-2	78.911	10.712
ct92-M305Pete-12	120362	03/12/2012	-2	78.919	10.73
ct92-M305Pete-12	120362	03/12/2012	-2	78.91	10.706
ct92-M305Pete-12	120362	03/12/2012	-2	78.919	10.739
ct92-M305Pete-12	120362	03/12/2012	-2	78.917	10.74

ct92-M305Pete-12	120362	03/12/2012	-2	78.916	10.641
ct92-M305Pete-12	120362	03/12/2012	-2	78.931	10.684
ct92-M305Pete-12	120362	03/12/2012	-2	78.932	10.606
ct92-M305Pete-12	120362	04/12/2012	-2	78.929	10.553
ct92-M305Pete-12	120362	04/12/2012	-2	78.927	10.568
ct92-M305Pete-12	120362	04/12/2012	-2	78.914	10.595
ct92-M305Pete-12	120362	04/12/2012	-2	78.913	10.596
ct92-M305Pete-12	120362	04/12/2012	-2	78.912	10.541
ct92-M305Pete-12	120362	04/12/2012	-2	78.923	10.682
ct92-M305Pete-12	120362	04/12/2012	-2	78.91	10.615
ct92-M305Pete-12	120362	04/12/2012	-2	78.911	10.595
ct92-M305Pete-12	120362	04/12/2012	-2	78.916	10.594
ct92-M305Pete-12	120362	04/12/2012	-2	78.914	10.568
ct92-M305Pete-12	120362	04/12/2012	-2	78.91	10.578
ct92-M305Pete-12	120362	04/12/2012	-2	78.895	10.577
ct92-M305Pete-12	120362	04/12/2012	-2	78.907	10.595
ct92-M305Pete-12	120362	04/12/2012	0	78.92	10.551
ct92-M305Pete-12	120362	04/12/2012	-2	78.915	10.553
ct92-M305Pete-12	120362	04/12/2012	-2	78.914	10.552
ct92-M305Pete-12	120362	04/12/2012	0	78.899	10.592
ct92-M305Pete-12	120362	04/12/2012	-2	78.903	10.597
ct92-M305Pete-12	120362	04/12/2012	-2	78.909	10.59
ct92-M305Pete-12	120362	04/12/2012	-2	78.923	10.601
ct92-M305Pete-12	120362	04/12/2012	-2	78.901	10.552
ct92-M305Pete-12	120362	04/12/2012	-2	78.9	10.542
ct92-M305Pete-12	120362	04/12/2012	-1	78.925	10.455
ct92-M305Pete-12	120362	04/12/2012	-2	78.919	10.492
ct92-M305Pete-12	120362	04/12/2012	-2	78.928	10.534
ct92-M305Pete-12	120362	04/12/2012	-2	78.929	10.532
ct92-M305Pete-12	120362	04/12/2012	-2	78.919	10.465
ct92-M305Pete-12	120362	04/12/2012	-2	78.914	10.484
ct92-M305Pete-12	120362	04/12/2012	1	78.924	10.506
ct92-M305Pete-12	120362	04/12/2012	0	78.92	10.465
ct92-M305Pete-12	120362	04/12/2012	-1	78.925	10.483
ct92-M305Pete-12	120362	04/12/2012	-1	78.928	10.477
ct92-M305Pete-12	120362	04/12/2012	-2	78.925	10.455
ct92-M305Pete-12	120362	04/12/2012	-1	78.923	10.416
ct92-M305Pete-12	120362	04/12/2012	2	78.92	10.5
ct92-M305Pete-12	120362	04/12/2012	-2	78.919	10.438
ct92-M305Pete-12	120362	04/12/2012	-2	78.922	10.483
ct92-M305Pete-12	120362	04/12/2012	-2	78.926	10.467
ct92-M305Pete-12	120362	04/12/2012	1	78.926	10.515
ct92-M305Pete-12	120362	04/12/2012	-2	78.922	10.412
ct92-M305Pete-12	120362	04/12/2012	-1	78.923	10.472
ct92-M305Pete-12	120362	04/12/2012	-1	78.927	10.438
ct92-M305Pete-12	120362	04/12/2012	0	78.91	10.438
ct92-M305Pete-12	120362	04/12/2012	-1	78.913	10.452
ct92-M305Pete-12	120362	04/12/2012	-2	78.914	10.452
ct92-M305Pete-12	120362	04/12/2012	-2	78.914	10.442
ct92-M305Pete-12	120362	04/12/2012	1	78.916	10.44
ct92-M305Pete-12	120362	04/12/2012	-1	78.912	10.417
ct92-M305Pete-12	120362	04/12/2012	-2	78.916	10.482
ct92-M305Pete-12	120362	04/12/2012	-2	78.912	10.492
ct92-M305Pete-12	120362	04/12/2012	-2	78.912	10.491
ct92-M305Pete-12	120362	04/12/2012	-2	78.913	10.476
ct92-M305Pete-12	120362	04/12/2012	-1	78.924	10.423
ct92-M305Pete-12	120362	04/12/2012	-1	78.918	10.431
ct92-M305Pete-12	120362	04/12/2012	-2	78.923	10.429
ct92-M305Pete-12	120362	04/12/2012	-2	78.925	10.461
ct92-M305Pete-12	120362	04/12/2012	-2	78.919	10.446
ct92-M305Pete-12	120362	04/12/2012	-2	78.918	10.448
ct92-M305Pete-12	120362	04/12/2012	-1	78.923	10.474
ct92-M305Pete-12	120362	04/12/2012	-1	78.921	10.515
ct92-M305Pete-12	120362	04/12/2012	-2	78.916	10.482
ct92-M305Pete-12	120362	04/12/2012	-1	78.924	10.492
ct92-M305Pete-12	120362	04/12/2012	-2	78.912	10.399
ct92-M305Pete-12	120362	04/12/2012	-2	78.922	10.469
ct92-M305Pete-12	120362	04/12/2012	2	78.918	10.459

ct92-M305Pete-12	120362	04/12/2012	-2	78.923	10.446
ct92-M305Pete-12	120362	04/12/2012	-2	78.922	10.457
ct92-M305Pete-12	120362	04/12/2012	-2	78.921	10.46
ct92-M305Pete-12	120362	04/12/2012	-1	78.927	10.504
ct92-M305Pete-12	120362	04/12/2012	-1	78.892	10.443
ct92-M305Pete-12	120362	04/12/2012	-2	78.923	10.429
ct92-M305Pete-12	120362	04/12/2012	-2	78.933	10.508
ct92-M305Pete-12	120362	04/12/2012	-2	78.929	10.514
ct92-M305Pete-12	120362	04/12/2012	-1	78.92	10.388
ct92-M305Pete-12	120362	05/12/2012	-2	78.92	10.396
ct92-M305Pete-12	120362	05/12/2012	0	78.918	10.538
ct92-M305Pete-12	120362	05/12/2012	-2	78.918	10.532
ct92-M305Pete-12	120362	05/12/2012	-2	78.931	10.519
ct92-M305Pete-12	120362	05/12/2012	-1	78.928	10.426
ct92-M305Pete-12	120362	05/12/2012	-2	78.931	10.455
ct92-M305Pete-12	120362	05/12/2012	1	78.909	10.481
ct92-M305Pete-12	120362	05/12/2012	-2	78.927	10.463
ct92-M305Pete-12	120362	05/12/2012	-2	78.925	10.486
ct92-M305Pete-12	120362	05/12/2012	-2	78.923	10.486
ct92-M305Pete-12	120362	05/12/2012	-1	78.902	10.513
ct92-M305Pete-12	120362	05/12/2012	-2	78.942	10.496
ct92-M305Pete-12	120362	05/12/2012	-2	78.943	10.509
ct92-M305Pete-12	120362	05/12/2012	-2	78.94	10.493
ct92-M305Pete-12	120362	05/12/2012	-2	78.945	10.697
ct92-M305Pete-12	120362	05/12/2012	0	78.98	10.323
ct92-M305Pete-12	120362	05/12/2012	-2	78.955	10.667
ct92-M305Pete-12	120362	05/12/2012	-2	78.922	10.578
ct92-M305Pete-12	120362	05/12/2012	-2	78.954	10.665
ct92-M305Pete-12	120362	05/12/2012	-2	78.947	10.646
ct92-M305Pete-12	120362	05/12/2012	-2	78.934	10.64
ct92-M305Pete-12	120362	05/12/2012	-2	78.921	10.548
ct92-M305Pete-12	120362	05/12/2012	-2	78.933	10.663
ct92-M305Pete-12	120362	05/12/2012	-2	78.93	10.641
ct92-M305Pete-12	120362	05/12/2012	-2	78.924	10.541
ct92-M305Pete-12	120362	05/12/2012	-2	78.924	10.54
ct92-M305Pete-12	120362	05/12/2012	-2	78.917	10.515
ct92-M305Pete-12	120362	05/12/2012	0	78.912	10.423
ct92-M305Pete-12	120362	05/12/2012	-2	78.916	10.563
ct92-M305Pete-12	120362	05/12/2012	-1	78.924	10.52
ct92-M305Pete-12	120362	05/12/2012	-2	78.916	10.563
ct92-M305Pete-12	120362	05/12/2012	-2	78.914	10.53
ct92-M305Pete-12	120362	05/12/2012	-2	78.898	10.565
ct92-M305Pete-12	120362	05/12/2012	-2	78.901	10.567
ct92-M305Pete-12	120362	05/12/2012	-2	78.912	10.519
ct92-M305Pete-12	120362	05/12/2012	-1	78.921	10.513
ct92-M305Pete-12	120362	05/12/2012	-2	78.932	10.477
ct92-M305Pete-12	120362	05/12/2012	-2	78.931	10.468
ct92-M305Pete-12	120362	05/12/2012	-2	78.923	10.472
ct92-M305Pete-12	120362	05/12/2012	-2	78.915	10.483
ct92-M305Pete-12	120362	05/12/2012	-2	78.913	10.495
ct92-M305Pete-12	120362	05/12/2012	-1	78.915	10.439
ct92-M305Pete-12	120362	05/12/2012	-2	78.911	10.496
ct92-M305Pete-12	120362	05/12/2012	-2	78.907	10.429
ct92-M305Pete-12	120362	05/12/2012	-1	78.922	10.442
ct92-M305Pete-12	120362	05/12/2012	-2	78.918	10.489
ct92-M305Pete-12	120362	05/12/2012	-2	78.921	10.505
ct92-M305Pete-12	120362	05/12/2012	-2	78.92	10.535
ct92-M305Pete-12	120362	05/12/2012	-2	78.918	10.545
ct92-M305Pete-12	120362	05/12/2012	-1	78.914	10.537
ct92-M305Pete-12	120362	05/12/2012	-2	78.912	10.541
ct92-M305Pete-12	120362	05/12/2012	-2	78.921	10.612
ct92-M305Pete-12	120362	05/12/2012	-2	78.916	10.615
ct92-M305Pete-12	120362	05/12/2012	-2	78.912	10.57
ct92-M305Pete-12	120362	05/12/2012	-2	78.91	10.58
ct92-M305Pete-12	120362	05/12/2012	-2	78.91	10.568
ct92-M305Pete-12	120362	05/12/2012	-2	78.909	10.584
ct92-M305Pete-12	120362	05/12/2012	-2	78.909	10.603
ct92-M305Pete-12	120362	05/12/2012	-2	78.909	10.596

ct92-M305Pete-12	120362	05/12/2012	-2	78.909	10.603
ct92-M305Pete-12	120362	05/12/2012	-2	78.91	10.598
ct92-M305Pete-12	120362	05/12/2012	-2	78.908	10.601
ct92-M305Pete-12	120362	06/12/2012	-2	78.909	10.592
ct92-M305Pete-12	120362	06/12/2012	-2	78.908	10.596
ct92-M305Pete-12	120362	06/12/2012	-2	78.908	10.599
ct92-M305Pete-12	120362	06/12/2012	-2	78.908	10.601
ct92-M305Pete-12	120362	06/12/2012	-2	78.91	10.603
ct92-M305Pete-12	120362	06/12/2012	-2	78.916	10.623
ct92-M305Pete-12	120362	06/12/2012	-2	78.917	10.626
ct92-M305Pete-12	120362	06/12/2012	-2	78.918	10.617
ct92-M305Pete-12	120362	06/12/2012	-2	78.918	10.615
ct92-M305Pete-12	120362	06/12/2012	-2	78.921	10.633
ct92-M305Pete-12	120362	06/12/2012	-2	78.925	10.644
ct92-M305Pete-12	120362	06/12/2012	-2	78.927	10.656
ct92-M305Pete-12	120362	06/12/2012	-2	78.923	10.638
ct92-M305Pete-12	120362	06/12/2012	-2	78.93	10.649
ct92-M305Pete-12	120362	06/12/2012	-2	78.93	10.679
ct92-M305Pete-12	120362	06/12/2012	-2	78.932	10.674
ct92-M305Pete-12	120362	06/12/2012	-2	78.928	10.657
ct92-M305Pete-12	120362	06/12/2012	-2	78.93	10.665
ct92-M305Pete-12	120362	06/12/2012	-2	78.934	10.686
ct92-M305Pete-12	120362	06/12/2012	-2	78.937	10.678
ct92-M305Pete-12	120362	06/12/2012	-2	78.944	10.721
ct92-M305Pete-12	120362	06/12/2012	-2	78.943	10.679
ct92-M305Pete-12	120362	06/12/2012	-2	78.945	10.686
ct92-M305Pete-12	120362	06/12/2012	-2	78.931	10.489
ct92-M305Pete-12	120362	06/12/2012	-2	78.932	10.489
ct92-M305Pete-12	120362	06/12/2012	-2	78.934	10.494
ct92-M305Pete-12	120362	06/12/2012	-2	78.937	10.502
ct92-M305Pete-12	120362	06/12/2012	-2	78.936	10.501
ct92-M305Pete-12	120362	06/12/2012	-1	78.934	10.519
ct92-M305Pete-12	120362	06/12/2012	-2	78.934	10.516
ct92-M305Pete-12	120362	06/12/2012	-2	78.934	10.518
ct92-M305Pete-12	120362	06/12/2012	-2	78.933	10.519
ct92-M305Pete-12	120362	06/12/2012	-2	78.931	10.567
ct92-M305Pete-12	120362	06/12/2012	-2	78.926	10.555
ct92-M305Pete-12	120362	06/12/2012	-1	78.923	10.562
ct92-M305Pete-12	120362	06/12/2012	-1	78.911	10.587
ct92-M305Pete-12	120362	06/12/2012	-1	78.91	10.578
ct92-M305Pete-12	120362	06/12/2012	-2	78.916	10.599
ct92-M305Pete-12	120362	06/12/2012	-2	78.912	10.596
ct92-M305Pete-12	120362	06/12/2012	-2	78.913	10.613
ct92-M305Pete-12	120362	06/12/2012	-2	78.914	10.665
ct92-M305Pete-12	120362	06/12/2012	-2	78.913	10.619
ct92-M305Pete-12	120362	06/12/2012	-2	78.901	10.613
ct92-M305Pete-12	120362	06/12/2012	-2	78.897	10.683
ct92-M305Pete-12	120362	06/12/2012	1	78.899	10.74
ct92-M305Pete-12	120362	06/12/2012	2	78.896	10.745
ct92-M305Pete-12	120362	06/12/2012	-1	78.907	10.792
ct92-M305Pete-12	120362	06/12/2012	-2	78.909	10.792
ct92-M305Pete-12	120362	06/12/2012	-1	78.887	10.76
ct92-M305Pete-12	120362	06/12/2012	-1	78.887	10.715
ct92-M305Pete-12	120362	06/12/2012	-2	78.894	10.725
ct92-M305Pete-12	120362	06/12/2012	-2	78.896	10.73
ct92-M305Pete-12	120362	06/12/2012	-1	78.898	10.764
ct92-M305Pete-12	120362	06/12/2012	-1	78.898	10.767
ct92-M305Pete-12	120362	06/12/2012	-1	78.905	10.773
ct92-M305Pete-12	120362	06/12/2012	-2	78.901	10.768
ct92-M305Pete-12	120362	06/12/2012	-2	78.896	10.769
ct92-M305Pete-12	120362	06/12/2012	-2	78.899	10.767
ct92-M305Pete-12	120362	06/12/2012	-2	78.896	10.777
ct92-M305Pete-12	120362	06/12/2012	-2	78.897	10.78
ct92-M305Pete-12	120362	06/12/2012	-2	78.887	10.784
ct92-M305Pete-12	120362	06/12/2012	-2	78.887	10.785
ct92-M305Pete-12	120362	06/12/2012	-2	78.886	10.781
ct92-M305Pete-12	120362	06/12/2012	-2	78.88	10.761
ct92-M305Pete-12	120362	06/12/2012	-2	78.896	10.756

ct92-M305Pete-12	120362	06/12/2012	-2	78.896	10.752
ct92-M305Pete-12	120362	06/12/2012	-2	78.893	10.675
ct92-M305Pete-12	120362	06/12/2012	-2	78.87	10.668
ct92-M305Pete-12	120362	07/12/2012	-2	78.893	10.689
ct92-M305Pete-12	120362	07/12/2012	-2	78.908	10.733
ct92-M305Pete-12	120362	07/12/2012	-2	78.895	10.682
ct92-M305Pete-12	120362	07/12/2012	-2	78.884	10.69
ct92-M305Pete-12	120362	07/12/2012	-2	78.89	10.734
ct92-M305Pete-12	120362	07/12/2012	-2	78.896	10.693
ct92-M305Pete-12	120362	07/12/2012	-2	78.909	10.843
ct92-M305Pete-12	120362	07/12/2012	-2	78.911	10.846
ct92-M305Pete-12	120362	07/12/2012	-2	78.913	10.818
ct92-M305Pete-12	120362	07/12/2012	-2	78.913	10.816
ct92-M305Pete-12	120362	07/12/2012	-2	78.907	10.845
ct92-M305Pete-12	120362	07/12/2012	-2	78.915	10.907
ct92-M305Pete-12	120362	07/12/2012	-2	78.912	10.891
ct92-M305Pete-12	120362	07/12/2012	-2	78.913	10.805
ct92-M305Pete-12	120362	07/12/2012	-2	78.91	10.871
ct92-M305Pete-12	120362	07/12/2012	-2	78.914	10.835
ct92-M305Pete-12	120362	07/12/2012	-2	78.901	10.794
ct92-M305Pete-12	120362	07/12/2012	-2	78.916	10.765
ct92-M305Pete-12	120362	07/12/2012	-2	78.917	10.744
ct92-M305Pete-12	120362	07/12/2012	-2	78.923	10.731
ct92-M305Pete-12	120362	07/12/2012	-2	78.92	10.772
ct92-M305Pete-12	120362	07/12/2012	-2	78.938	10.684
ct92-M305Pete-12	120362	07/12/2012	-2	78.903	10.682
ct92-M305Pete-12	120362	07/12/2012	-2	78.928	10.65
ct92-M305Pete-12	120362	07/12/2012	-2	78.929	10.64
ct92-M305Pete-12	120362	07/12/2012	-2	78.926	10.635
ct92-M305Pete-12	120362	07/12/2012	-2	78.926	10.634
ct92-M305Pete-12	120362	07/12/2012	-2	78.926	10.634
ct92-M305Pete-12	120362	07/12/2012	-2	78.928	10.647
ct92-M305Pete-12	120362	07/12/2012	-2	78.928	10.648
ct92-M305Pete-12	120362	07/12/2012	-2	78.93	10.662
ct92-M305Pete-12	120362	07/12/2012	-2	78.936	10.629
ct92-M305Pete-12	120362	07/12/2012	-2	78.929	10.663
ct92-M305Pete-12	120362	07/12/2012	-2	78.928	10.614
ct92-M305Pete-12	120362	07/12/2012	-2	78.927	10.634
ct92-M305Pete-12	120362	07/12/2012	-2	78.928	10.613
ct92-M305Pete-12	120362	07/12/2012	-2	78.945	10.567
ct92-M305Pete-12	120362	07/12/2012	-2	78.932	10.592
ct92-M305Pete-12	120362	07/12/2012	-2	78.948	10.559
ct92-M305Pete-12	120362	07/12/2012	-2	78.925	10.522
ct92-M305Pete-12	120362	07/12/2012	-2	78.928	10.488
ct92-M305Pete-12	120362	07/12/2012	-2	78.929	10.489
ct92-M305Pete-12	120362	07/12/2012	-2	78.925	10.585
ct92-M305Pete-12	120362	07/12/2012	2	78.932	10.517
ct92-M305Pete-12	120362	07/12/2012	-1	78.92	10.475
ct92-M305Pete-12	120362	07/12/2012	-2	78.918	10.479
ct92-M305Pete-12	120362	07/12/2012	-2	78.915	10.473
ct92-M305Pete-12	120362	07/12/2012	-2	78.915	10.458
ct92-M305Pete-12	120362	07/12/2012	-2	78.918	10.446
ct92-M305Pete-12	120362	07/12/2012	-2	78.936	10.438
ct92-M305Pete-12	120362	07/12/2012	-2	78.918	10.444
ct92-M305Pete-12	120362	07/12/2012	-1	78.901	10.341
ct92-M305Pete-12	120362	07/12/2012	-1	78.933	10.434
ct92-M305Pete-12	120362	07/12/2012	-2	78.917	10.408
ct92-M305Pete-12	120362	07/12/2012	-2	78.921	10.403
ct92-M305Pete-12	120362	07/12/2012	-2	78.921	10.401
ct92-M305Pete-12	120362	07/12/2012	-2	78.926	10.405
ct92-M305Pete-12	120362	07/12/2012	-2	78.929	10.409
ct92-M305Pete-12	120362	07/12/2012	-2	78.943	10.397
ct92-M305Pete-12	120362	08/12/2012	-2	78.962	10.634
ct92-M305Pete-12	120362	08/12/2012	-2	78.941	10.536
ct92-M305Pete-12	120362	08/12/2012	-2	78.944	10.566
ct92-M305Pete-12	120362	08/12/2012	-2	78.936	10.582
ct92-M305Pete-12	120362	08/12/2012	-2	78.936	10.571
ct92-M305Pete-12	120362	08/12/2012	-2	78.944	10.577

ct92-M305Pete-12	120362	08/12/2012	-2	78.941	10.593
ct92-M305Pete-12	120362	08/12/2012	-2	78.942	10.6
ct92-M305Pete-12	120362	08/12/2012	-2	78.946	10.607
ct92-M305Pete-12	120362	08/12/2012	-2	78.942	10.6
ct92-M305Pete-12	120362	08/12/2012	-2	78.948	10.611
ct92-M305Pete-12	120362	08/12/2012	-1	78.945	10.523
ct92-M305Pete-12	120362	08/12/2012	-2	78.939	10.561
ct92-M305Pete-12	120362	08/12/2012	-2	78.951	10.547
ct92-M305Pete-12	120362	08/12/2012	-2	78.952	10.553
ct92-M305Pete-12	120362	08/12/2012	-2	78.95	10.552
ct92-M305Pete-12	120362	08/12/2012	-2	78.958	10.576
ct92-M305Pete-12	120362	08/12/2012	-2	78.962	10.558
ct92-M305Pete-12	120362	08/12/2012	-2	78.947	10.507
ct92-M305Pete-12	120362	08/12/2012	-2	78.963	10.539
ct92-M305Pete-12	120362	08/12/2012	-2	78.969	10.544
ct92-M305Pete-12	120362	08/12/2012	-2	78.961	10.542
ct92-M305Pete-12	120362	08/12/2012	-2	78.979	10.592
ct92-M305Pete-12	120362	08/12/2012	-2	78.977	10.583
ct92-M305Pete-12	120362	08/12/2012	-2	78.947	10.456
ct92-M305Pete-12	120362	08/12/2012	0	78.938	10.515
ct92-M305Pete-12	120362	08/12/2012	-2	78.938	10.503
ct92-M305Pete-12	120362	08/12/2012	-2	78.938	10.503
ct92-M305Pete-12	120362	08/12/2012	-2	78.938	10.503
ct92-M305Pete-12	120362	08/12/2012	-1	78.926	10.395
ct92-M305Pete-12	120362	08/12/2012	-2	78.937	10.422
ct92-M305Pete-12	120362	08/12/2012	-2	78.939	10.426
ct92-M305Pete-12	120362	08/12/2012	-2	78.941	10.426
ct92-M305Pete-12	120362	08/12/2012	-1	78.917	10.368
ct92-M305Pete-12	120362	08/12/2012	-2	78.913	10.377
ct92-M305Pete-12	120362	08/12/2012	-2	78.909	10.368
ct92-M305Pete-12	120362	08/12/2012	-2	78.914	10.445
ct92-M305Pete-12	120362	08/12/2012	-2	78.948	10.502
ct92-M305Pete-12	120362	08/12/2012	-2	78.948	10.504
ct92-M305Pete-12	120362	08/12/2012	-2	78.93	10.526
ct92-M305Pete-12	120362	08/12/2012	-1	78.93	10.571
ct92-M305Pete-12	120362	08/12/2012	-2	78.932	10.53
ct92-M305Pete-12	120362	08/12/2012	-1	78.931	10.618
ct92-M305Pete-12	120362	08/12/2012	-2	78.93	10.607
ct92-M305Pete-12	120362	08/12/2012	-2	78.93	10.613
ct92-M305Pete-12	120362	08/12/2012	-2	78.923	10.6
ct92-M305Pete-12	120362	08/12/2012	-2	78.924	10.599
ct92-M305Pete-12	120362	08/12/2012	-1	78.927	10.583
ct92-M305Pete-12	120362	08/12/2012	-2	78.922	10.597
ct92-M305Pete-12	120362	08/12/2012	-2	78.926	10.565
ct92-M305Pete-12	120362	08/12/2012	-2	78.926	10.571
ct92-M305Pete-12	120362	08/12/2012	-2	78.921	10.55
ct92-M305Pete-12	120362	08/12/2012	-1	78.926	10.564
ct92-M305Pete-12	120362	08/12/2012	-2	78.942	10.547
ct92-M305Pete-12	120362	08/12/2012	-2	78.933	10.435
ct92-M305Pete-12	120362	08/12/2012	-1	78.92	10.495
ct92-M305Pete-12	120362	08/12/2012	-2	78.937	10.446
ct92-M305Pete-12	120362	08/12/2012	-2	78.926	10.525
ct92-M305Pete-12	120362	08/12/2012	-2	78.956	10.567
ct92-M305Pete-12	120362	08/12/2012	-2	78.94	10.536
ct92-M305Pete-12	120362	08/12/2012	-2	78.925	10.579
ct92-M305Pete-12	120362	08/12/2012	-2	78.935	10.631
ct92-M305Pete-12	120362	08/12/2012	-2	78.925	10.578
ct92-M305Pete-12	120362	08/12/2012	-2	78.915	10.672
ct92-M305Pete-12	120362	08/12/2012	-2	78.916	10.67
ct92-M305Pete-12	120362	08/12/2012	-2	78.92	10.602
ct92-M305Pete-12	120362	08/12/2012	-2	78.91	10.571
ct92-M305Pete-12	120362	08/12/2012	-2	78.912	10.576
ct92-M305Pete-12	120362	08/12/2012	-2	78.914	10.582
ct92-M305Pete-12	120362	08/12/2012	-2	78.912	10.577
ct92-M305Pete-12	120362	08/12/2012	-2	78.919	10.584
ct92-M305Pete-12	120362	08/12/2012	-2	78.915	10.577
ct92-M305Pete-12	120362	08/12/2012	-2	78.916	10.578
ct92-M305Pete-12	120362	09/12/2012	-2	78.915	10.593

ct92-M305Pete-12	120362	09/12/2012	-2	78.92	10.607
ct92-M305Pete-12	120362	09/12/2012	-2	78.917	10.599
ct92-M305Pete-12	120362	09/12/2012	-2	78.921	10.611
ct92-M305Pete-12	120362	09/12/2012	-2	78.928	10.623
ct92-M305Pete-12	120362	09/12/2012	-2	78.916	10.616
ct92-M305Pete-12	120362	09/12/2012	-2	78.916	10.616
ct92-M305Pete-12	120362	09/12/2012	-2	78.909	10.65
ct92-M305Pete-12	120362	09/12/2012	-2	78.918	10.61
ct92-M305Pete-12	120362	09/12/2012	-2	78.922	10.619
ct92-M305Pete-12	120362	09/12/2012	-2	78.918	10.597
ct92-M305Pete-12	120362	09/12/2012	-2	78.919	10.6
ct92-M305Pete-12	120362	09/12/2012	-2	78.927	10.563
ct92-M305Pete-12	120362	09/12/2012	-2	78.931	10.48
ct92-M305Pete-12	120362	09/12/2012	-2	78.941	10.507
ct92-M305Pete-12	120362	09/12/2012	-2	78.936	10.507
ct92-M305Pete-12	120362	09/12/2012	-2	78.931	10.542
ct92-M305Pete-12	120362	09/12/2012	-2	78.934	10.528
ct92-M305Pete-12	120362	09/12/2012	-2	78.951	10.509
ct92-M305Pete-12	120362	09/12/2012	-2	78.946	10.672
ct92-M305Pete-12	120362	09/12/2012	-2	78.923	10.59
ct92-M305Pete-12	120362	09/12/2012	-2	78.962	10.629
ct92-M305Pete-12	120362	09/12/2012	-2	78.933	10.611
ct92-M305Pete-12	120362	09/12/2012	-2	78.926	10.605
ct92-M305Pete-12	120362	09/12/2012	-2	78.926	10.678
ct92-M305Pete-12	120362	09/12/2012	-2	78.923	10.606
ct92-M305Pete-12	120362	09/12/2012	-2	78.934	10.638
ct92-M305Pete-12	120362	09/12/2012	-2	78.934	10.611
ct92-M305Pete-12	120362	09/12/2012	-2	78.927	10.631
ct92-M305Pete-12	120362	09/12/2012	-2	78.927	10.631
ct92-M305Pete-12	120362	09/12/2012	-2	78.933	10.639
ct92-M305Pete-12	120362	09/12/2012	-2	78.936	10.665
ct92-M305Pete-12	120362	09/12/2012	-2	78.936	10.656
ct92-M305Pete-12	120362	09/12/2012	-2	78.941	10.662
ct92-M305Pete-12	120362	09/12/2012	-2	78.932	10.711
ct92-M305Pete-12	120362	09/12/2012	-2	78.93	10.655
ct92-M305Pete-12	120362	09/12/2012	-2	78.93	10.658
ct92-M305Pete-12	120362	09/12/2012	-2	78.932	10.67
ct92-M305Pete-12	120362	09/12/2012	-2	78.937	10.678
ct92-M305Pete-12	120362	09/12/2012	-2	78.935	10.691
ct92-M305Pete-12	120362	09/12/2012	-2	78.925	10.77
ct92-M305Pete-12	120362	09/12/2012	-2	78.924	10.76
ct92-M305Pete-12	120362	09/12/2012	-1	78.92	10.707
ct92-M305Pete-12	120362	09/12/2012	-2	78.921	10.685
ct92-M305Pete-12	120362	09/12/2012	-2	78.921	10.684
ct92-M305Pete-12	120362	09/12/2012	-2	78.914	10.642
ct92-M305Pete-12	120362	09/12/2012	-2	78.915	10.596
ct92-M305Pete-12	120362	09/12/2012	-2	78.923	10.688
ct92-M305Pete-12	120362	09/12/2012	-2	78.914	10.668
ct92-M305Pete-12	120362	09/12/2012	-2	78.921	10.651
ct92-M305Pete-12	120362	09/12/2012	-2	78.921	10.577
ct92-M305Pete-12	120362	09/12/2012	-2	78.919	10.65
ct92-M305Pete-12	120362	09/12/2012	-2	78.915	10.61
ct92-M305Pete-12	120362	09/12/2012	-2	78.92	10.561
ct92-M305Pete-12	120362	09/12/2012	-2	78.932	10.501
ct92-M305Pete-12	120362	09/12/2012	-1	78.931	10.546
ct92-M305Pete-12	120362	09/12/2012	-1	78.947	10.622
ct92-M305Pete-12	120362	09/12/2012	-1	78.939	10.58
ct92-M305Pete-12	120362	09/12/2012	-2	78.932	10.627
ct92-M305Pete-12	120362	09/12/2012	-2	78.945	10.586
ct92-M305Pete-12	120362	09/12/2012	-2	78.942	10.599
ct92-M305Pete-12	120362	09/12/2012	-2	78.947	10.591
ct92-M305Pete-12	120362	09/12/2012	-1	78.931	10.562
ct92-M305Pete-12	120362	09/12/2012	-2	78.941	10.594
ct92-M305Pete-12	120362	10/12/2012	-2	78.931	10.623
ct92-M305Pete-12	120362	10/12/2012	-1	78.929	10.622
ct92-M305Pete-12	120362	10/12/2012	-2	78.925	10.606
ct92-M305Pete-12	120362	10/12/2012	-2	78.93	10.617
ct92-M305Pete-12	120362	10/12/2012	-2	78.933	10.571

ct92-M305Pete-12	120362	10/12/2012	-2	78.931	10.572
ct92-M305Pete-12	120362	10/12/2012	-2	78.922	10.568
ct92-M305Pete-12	120362	10/12/2012	-2	78.92	10.547
ct92-M305Pete-12	120362	10/12/2012	-2	78.922	10.557
ct92-M305Pete-12	120362	10/12/2012	-2	78.922	10.535
ct92-M305Pete-12	120362	10/12/2012	-2	78.922	10.561
ct92-M305Pete-12	120362	10/12/2012	-2	78.922	10.528
ct92-M305Pete-12	120362	10/12/2012	-2	78.92	10.548
ct92-M305Pete-12	120362	10/12/2012	-2	78.922	10.517
ct92-M305Pete-12	120362	10/12/2012	-2	78.928	10.494
ct92-M305Pete-12	120362	10/12/2012	-2	78.924	10.446
ct92-M305Pete-12	120362	10/12/2012	-2	78.928	10.455
ct92-M305Pete-12	120362	10/12/2012	-2	78.919	10.52
ct92-M305Pete-12	120362	10/12/2012	-2	78.921	10.527
ct92-M305Pete-12	120362	10/12/2012	-2	78.922	10.524
ct92-M305Pete-12	120362	10/12/2012	-2	78.924	10.527
ct92-M305Pete-12	120362	10/12/2012	-2	78.927	10.441
ct92-M305Pete-12	120362	10/12/2012	-2	78.922	10.524
ct92-M305Pete-12	120362	10/12/2012	-2	78.922	10.516
ct92-M305Pete-12	120362	10/12/2012	-2	78.929	10.555
ct92-M305Pete-12	120362	10/12/2012	-2	78.922	10.515
ct92-M305Pete-12	120362	10/12/2012	-2	78.921	10.534
ct92-M305Pete-12	120362	10/12/2012	-2	78.929	10.53
ct92-M305Pete-12	120362	10/12/2012	-2	78.936	10.547
ct92-M305Pete-12	120362	10/12/2012	-2	78.936	10.547
ct92-M305Pete-12	120362	10/12/2012	-2	78.93	10.545
ct92-M305Pete-12	120362	10/12/2012	-1	78.917	10.511
ct92-M305Pete-12	120362	10/12/2012	-2	78.926	10.551
ct92-M305Pete-12	120362	10/12/2012	-1	78.924	10.619
ct92-M305Pete-12	120362	10/12/2012	-2	78.926	10.625
ct92-M305Pete-12	120362	10/12/2012	-2	78.941	10.598
ct92-M305Pete-12	120362	10/12/2012	-2	78.943	10.601
ct92-M305Pete-12	120362	10/12/2012	-2	78.943	10.602
ct92-M305Pete-12	120362	10/12/2012	-2	78.929	10.66
ct92-M305Pete-12	120362	10/12/2012	-2	78.943	10.601
ct92-M305Pete-12	120362	10/12/2012	-2	78.928	10.659
ct92-M305Pete-12	120362	10/12/2012	-2	78.928	10.663
ct92-M305Pete-12	120362	10/12/2012	-2	78.939	10.616
ct92-M305Pete-12	120362	10/12/2012	-2	78.929	10.693
ct92-M305Pete-12	120362	10/12/2012	-2	78.932	10.632
ct92-M305Pete-12	120362	10/12/2012	-2	78.931	10.63
ct92-M305Pete-12	120362	10/12/2012	-2	78.923	10.626
ct92-M305Pete-12	120362	10/12/2012	-2	78.922	10.626
ct92-M305Pete-12	120362	10/12/2012	-2	78.925	10.628
ct92-M305Pete-12	120362	10/12/2012	-2	78.921	10.483
ct92-M305Pete-12	120362	10/12/2012	-2	78.924	10.49
ct92-M305Pete-12	120362	10/12/2012	-2	78.916	10.585
ct92-M305Pete-12	120362	10/12/2012	-1	78.916	10.582
ct92-M305Pete-12	120362	10/12/2012	-2	78.927	10.587
ct92-M305Pete-12	120362	10/12/2012	-1	78.914	10.526
ct92-M305Pete-12	120362	10/12/2012	-2	78.916	10.528
ct92-M305Pete-12	120362	10/12/2012	-2	78.918	10.534
ct92-M305Pete-12	120362	10/12/2012	-2	78.918	10.537
ct92-M305Pete-12	120362	10/12/2012	-2	78.925	10.579
ct92-M305Pete-12	120362	10/12/2012	-2	78.922	10.584
ct92-M305Pete-12	120362	10/12/2012	-2	78.918	10.55
ct92-M305Pete-12	120362	10/12/2012	-1	78.898	10.684
ct92-M305Pete-12	120362	10/12/2012	-2	78.898	10.691
ct92-M305Pete-12	120362	10/12/2012	-2	78.897	10.788
ct92-M305Pete-12	120362	10/12/2012	-2	78.897	10.786
ct92-M305Pete-12	120362	10/12/2012	-2	78.9	10.795
ct92-M305Pete-12	120362	10/12/2012	-2	78.901	10.66
ct92-M305Pete-12	120362	10/12/2012	-2	78.903	10.607
ct92-M305Pete-12	120362	10/12/2012	-2	78.905	10.684
ct92-M305Pete-12	120362	11/12/2012	-2	78.905	10.641
ct92-M305Pete-12	120362	11/12/2012	-1	78.909	10.695
ct92-M305Pete-12	120362	11/12/2012	-2	78.909	10.701
ct92-M305Pete-12	120362	11/12/2012	-2	78.908	10.702

ct92-M305Pete-12	120362	11/12/2012	-2	78.894	10.674
ct92-M305Pete-12	120362	11/12/2012	-2	78.914	10.668
ct92-M305Pete-12	120362	11/12/2012	-2	78.906	10.628
ct92-M305Pete-12	120362	11/12/2012	-2	78.914	10.687
ct92-M305Pete-12	120362	11/12/2012	-2	78.908	10.672
ct92-M305Pete-12	120362	11/12/2012	-2	78.914	10.691
ct92-M305Pete-12	120362	11/12/2012	-1	78.912	10.676
ct92-M305Pete-12	120362	11/12/2012	-2	78.91	10.679
ct92-M305Pete-12	120362	11/12/2012	-2	78.911	10.681
ct92-M305Pete-12	120362	11/12/2012	-2	78.914	10.67
ct92-M305Pete-12	120362	11/12/2012	-1	78.91	10.624
ct92-M305Pete-12	120362	11/12/2012	-2	78.906	10.642
ct92-M305Pete-12	120362	11/12/2012	-2	78.918	10.576
ct92-M305Pete-12	120362	11/12/2012	-2	78.918	10.575
ct92-M305Pete-12	120362	11/12/2012	-2	78.92	10.568
ct92-M305Pete-12	120362	11/12/2012	-2	78.922	10.605
ct92-M305Pete-12	120362	11/12/2012	-2	78.923	10.613
ct92-M305Pete-12	120362	11/12/2012	-2	78.926	10.6
ct92-M305Pete-12	120362	11/12/2012	-2	78.925	10.609
ct92-M305Pete-12	120362	11/12/2012	-2	78.912	10.616
ct92-M305Pete-12	120362	11/12/2012	-2	78.911	10.685
ct92-M305Pete-12	120362	11/12/2012	-2	78.924	10.678
ct92-M305Pete-12	120362	11/12/2012	-2	78.9	10.695
ct92-M305Pete-12	120362	11/12/2012	-2	78.905	10.669
ct92-M305Pete-12	120362	11/12/2012	-2	78.906	10.681
ct92-M305Pete-12	120362	11/12/2012	-2	78.927	10.692
ct92-M305Pete-12	120362	11/12/2012	-2	78.904	10.667
ct92-M305Pete-12	120362	11/12/2012	-2	78.928	10.696
ct92-M305Pete-12	120362	11/12/2012	-2	78.913	10.712
ct92-M305Pete-12	120362	11/12/2012	-2	78.908	10.714
ct92-M305Pete-12	120362	11/12/2012	-2	78.908	10.714
ct92-M305Pete-12	120362	11/12/2012	-2	78.899	10.646
ct92-M305Pete-12	120362	11/12/2012	-2	78.9	10.646
ct92-M305Pete-12	120362	11/12/2012	-2	78.9	10.649
ct92-M305Pete-12	120362	11/12/2012	-2	78.904	10.67
ct92-M305Pete-12	120362	11/12/2012	-2	78.905	10.655
ct92-M305Pete-12	120362	11/12/2012	-2	78.904	10.651
ct92-M305Pete-12	120362	11/12/2012	-2	78.906	10.669
ct92-M305Pete-12	120362	11/12/2012	-2	78.907	10.669
ct92-M305Pete-12	120362	11/12/2012	-2	78.906	10.648
ct92-M305Pete-12	120362	11/12/2012	-1	78.879	10.625
ct92-M305Pete-12	120362	11/12/2012	-2	78.857	10.812
ct92-M305Pete-12	120362	11/12/2012	-2	78.849	10.79
ct92-M305Pete-12	120362	11/12/2012	-2	78.85	10.64
ct92-M305Pete-12	120362	11/12/2012	-2	78.853	10.651
ct92-M305Pete-12	120362	11/12/2012	-2	78.894	10.526
ct92-M305Pete-12	120362	11/12/2012	-2	78.856	10.653
ct92-M305Pete-12	120362	11/12/2012	-2	78.868	10.693
ct92-M305Pete-12	120362	11/12/2012	-2	78.902	10.605
ct92-M305Pete-12	120362	11/12/2012	-2	78.928	10.612
ct92-M305Pete-12	120362	11/12/2012	-2	78.892	10.888
ct92-M305Pete-12	120362	11/12/2012	-1	78.921	10.592
ct92-M305Pete-12	120362	11/12/2012	-2	78.935	10.528
ct92-M305Pete-12	120362	11/12/2012	-1	78.929	10.524
ct92-M305Pete-12	120362	11/12/2012	-2	78.938	10.497
ct92-M305Pete-12	120362	11/12/2012	-2	78.934	10.519
ct92-M305Pete-12	120362	11/12/2012	-2	78.937	10.498
ct92-M305Pete-12	120362	11/12/2012	-2	78.946	10.441
ct92-M305Pete-12	120362	11/12/2012	-2	78.939	10.476
ct92-M305Pete-12	120362	11/12/2012	-2	78.952	10.406
ct92-M305Pete-12	120362	11/12/2012	-2	78.945	10.457
ct92-M305Pete-12	120362	11/12/2012	-1	78.927	10.651
ct92-M305Pete-12	120362	11/12/2012	-1	78.93	10.683
ct92-M305Pete-12	120362	11/12/2012	-1	78.931	10.687
ct92-M305Pete-12	120362	11/12/2012	-2	78.93	10.687
ct92-M305Pete-12	120362	12/12/2012	-2	78.938	10.628
ct92-M305Pete-12	120362	12/12/2012	-2	78.934	10.631
ct92-M305Pete-12	120362	12/12/2012	-2	78.931	10.628

ct92-M305Pete-12	120362	12/12/2012	-2	78.935	10.622
ct92-M305Pete-12	120362	12/12/2012	-2	78.939	10.598
ct92-M305Pete-12	120362	12/12/2012	-2	78.958	10.594
ct92-M305Pete-12	120362	12/12/2012	-2	78.937	10.591
ct92-M305Pete-12	120362	12/12/2012	-2	78.936	10.604
ct92-M305Pete-12	120362	12/12/2012	-2	78.951	10.589
ct92-M305Pete-12	120362	12/12/2012	-2	78.948	10.603
ct92-M305Pete-12	120362	12/12/2012	-2	78.945	10.613
ct92-M305Pete-12	120362	12/12/2012	-1	78.949	10.586
ct92-M305Pete-12	120362	12/12/2012	-2	78.951	10.571
ct92-M305Pete-12	120362	12/12/2012	-2	78.934	10.598
ct92-M305Pete-12	120362	12/12/2012	-1	78.926	10.624
ct92-M305Pete-12	120362	12/12/2012	-2	78.927	10.601
ct92-M305Pete-12	120362	12/12/2012	-1	78.931	10.564
ct92-M305Pete-12	120362	12/12/2012	-2	78.926	10.546
ct92-M305Pete-12	120362	12/12/2012	-2	78.94	10.577
ct92-M305Pete-12	120362	12/12/2012	-2	78.947	10.617
ct92-M305Pete-12	120362	12/12/2012	-2	78.928	10.538
ct92-M305Pete-12	120362	12/12/2012	-2	78.922	10.55
ct92-M305Pete-12	120362	12/12/2012	-1	78.913	10.545
ct92-M305Pete-12	120362	12/12/2012	-2	78.912	10.54
ct92-M305Pete-12	120362	12/12/2012	-1	78.927	10.479
ct92-M305Pete-12	120362	12/12/2012	-1	78.913	10.548
ct92-M305Pete-12	120362	12/12/2012	-1	78.918	10.564
ct92-M305Pete-12	120362	12/12/2012	-2	78.946	10.641
ct92-M305Pete-12	120362	12/12/2012	-2	78.925	10.598
ct92-M305Pete-12	120362	12/12/2012	-2	78.925	10.587
ct92-M305Pete-12	120362	12/12/2012	-1	78.931	10.586
ct92-M305Pete-12	120362	12/12/2012	0	78.952	10.42
ct92-M305Pete-12	120362	12/12/2012	-1	78.938	10.45
ct92-M305Pete-12	120362	12/12/2012	-1	78.928	10.657
ct92-M305Pete-12	120362	12/12/2012	-1	78.915	10.587
ct92-M305Pete-12	120362	12/12/2012	-1	78.918	10.458
ct92-M305Pete-12	120362	12/12/2012	-2	78.921	10.647
ct92-M305Pete-12	120362	12/12/2012	-2	78.925	10.518
ct92-M305Pete-12	120362	12/12/2012	-1	78.94	10.379
ct92-M305Pete-12	120362	12/12/2012	-2	78.916	10.591
ct92-M305Pete-12	120362	12/12/2012	-2	78.919	10.532
ct92-M305Pete-12	120362	12/12/2012	-2	78.922	10.524
ct92-M305Pete-12	120362	12/12/2012	-2	78.908	10.458
ct92-M305Pete-12	120362	12/12/2012	-2	78.907	10.459
ct92-M305Pete-12	120362	12/12/2012	-2	78.915	10.517
ct92-M305Pete-12	120362	12/12/2012	-2	78.909	10.511
ct92-M305Pete-12	120362	12/12/2012	-2	78.91	10.538
ct92-M305Pete-12	120362	12/12/2012	-2	78.916	10.514
ct92-M305Pete-12	120362	12/12/2012	0	78.927	10.543
ct92-M305Pete-12	120362	12/12/2012	-2	78.927	10.542
ct92-M305Pete-12	120362	12/12/2012	-2	78.927	10.541
ct92-M305Pete-12	120362	12/12/2012	-2	78.925	10.538
ct92-M305Pete-12	120362	12/12/2012	-2	78.924	10.536
ct92-M305Pete-12	120362	12/12/2012	-2	78.925	10.546
ct92-M305Pete-12	120362	12/12/2012	-2	78.925	10.54
ct92-M305Pete-12	120362	12/12/2012	-2	78.929	10.533
ct92-M305Pete-12	120362	12/12/2012	-2	78.926	10.541
ct92-M305Pete-12	120362	12/12/2012	-1	78.918	10.455
ct92-M305Pete-12	120362	12/12/2012	-2	78.907	10.449
ct92-M305Pete-12	120362	12/12/2012	-2	78.91	10.456
ct92-M305Pete-12	120362	12/12/2012	-2	78.909	10.493
ct92-M305Pete-12	120362	12/12/2012	-2	78.909	10.49
ct92-M305Pete-12	120362	12/12/2012	-2	78.907	10.477
ct92-M305Pete-12	120362	12/12/2012	-2	78.908	10.476
ct92-M305Pete-12	120362	12/12/2012	-2	78.913	10.382
ct92-M305Pete-12	120362	12/12/2012	-2	78.898	10.477
ct92-M305Pete-12	120362	12/12/2012	-2	78.901	10.363
ct92-M305Pete-12	120362	12/12/2012	-2	78.9	10.455
ct92-M305Pete-12	120362	12/12/2012	-2	78.906	10.45
ct92-M305Pete-12	120362	12/12/2012	2	78.917	10.458
ct92-M305Pete-12	120362	13/12/2012	1	78.922	10.403

ct92-M305Pete-12	120362	13/12/2012	-2	78.921	10.406
ct92-M305Pete-12	120362	13/12/2012	-2	78.928	10.495
ct92-M305Pete-12	120362	13/12/2012	-2	78.911	10.506
ct92-M305Pete-12	120362	13/12/2012	-2	78.92	10.51
ct92-M305Pete-12	120362	13/12/2012	-2	78.921	10.533
ct92-M305Pete-12	120362	13/12/2012	-2	78.92	10.535
ct92-M305Pete-12	120362	13/12/2012	-2	78.92	10.535
ct92-M305Pete-12	120362	13/12/2012	-2	78.914	10.556
ct92-M305Pete-12	120362	13/12/2012	-2	78.915	10.559
ct92-M305Pete-12	120362	13/12/2012	-2	78.913	10.599
ct92-M305Pete-12	120362	13/12/2012	-2	78.914	10.602
ct92-M305Pete-12	120362	13/12/2012	-2	78.913	10.608
ct92-M305Pete-12	120362	13/12/2012	-2	78.912	10.613
ct92-M305Pete-12	120362	13/12/2012	-2	78.911	10.616
ct92-M305Pete-12	120362	13/12/2012	-1	78.909	10.574
ct92-M305Pete-12	120362	13/12/2012	-2	78.903	10.719
ct92-M305Pete-12	120362	13/12/2012	-2	78.893	10.716
ct92-M305Pete-12	120362	13/12/2012	-2	78.907	10.637
ct92-M305Pete-12	120362	13/12/2012	0	78.856	10.611
ct92-M305Pete-12	120362	13/12/2012	-2	78.854	10.664
ct92-M305Pete-12	120362	13/12/2012	-1	78.864	10.632
ct92-M305Pete-12	120362	13/12/2012	-2	78.905	10.59
ct92-M305Pete-12	120362	13/12/2012	-1	78.913	10.606
ct92-M305Pete-12	120362	13/12/2012	-2	78.918	10.584
ct92-M305Pete-12	120362	13/12/2012	-2	78.913	10.607
ct92-M305Pete-12	120362	13/12/2012	-2	78.916	10.63
ct92-M305Pete-12	120362	13/12/2012	-2	78.867	10.614
ct92-M305Pete-12	120362	13/12/2012	-2	78.911	10.615
ct92-M305Pete-12	120362	13/12/2012	-2	78.911	10.66
ct92-M305Pete-12	120362	13/12/2012	-2	78.923	10.634
ct92-M305Pete-12	120362	13/12/2012	-1	78.921	10.618
ct92-M305Pete-12	120362	13/12/2012	-2	78.917	10.634
ct92-M305Pete-12	120362	13/12/2012	-2	78.915	10.663
ct92-M305Pete-12	120362	13/12/2012	-2	78.909	10.666
ct92-M305Pete-12	120362	13/12/2012	-2	78.917	10.648
ct92-M305Pete-12	120362	13/12/2012	-2	78.909	10.641
ct92-M305Pete-12	120362	13/12/2012	-2	78.905	10.673
ct92-M305Pete-12	120362	13/12/2012	-1	78.91	10.662
ct92-M305Pete-12	120362	13/12/2012	-2	78.903	10.687
ct92-M305Pete-12	120362	13/12/2012	-2	78.906	10.676
ct92-M305Pete-12	120362	13/12/2012	-2	78.904	10.678
ct92-M305Pete-12	120362	13/12/2012	-2	78.895	10.749
ct92-M305Pete-12	120362	13/12/2012	-2	78.905	10.733
ct92-M305Pete-12	120362	13/12/2012	-2	78.89	10.78
ct92-M305Pete-12	120362	13/12/2012	-2	78.899	10.689
ct92-M305Pete-12	120362	13/12/2012	-2	78.928	10.698
ct92-M305Pete-12	120362	13/12/2012	-2	78.929	10.811
ct92-M305Pete-12	120362	13/12/2012	-2	78.935	10.77
ct92-M305Pete-12	120362	13/12/2012	-2	78.938	10.779
ct92-M305Pete-12	120362	13/12/2012	-2	78.93	10.752
ct92-M305Pete-12	120362	13/12/2012	-2	78.916	10.527
ct92-M305Pete-12	120362	13/12/2012	-2	78.915	10.558
ct92-M305Pete-12	120362	13/12/2012	-2	78.905	10.585
ct92-M305Pete-12	120362	13/12/2012	-2	78.904	10.594
ct92-M305Pete-12	120362	13/12/2012	-2	78.91	10.702
ct92-M305Pete-12	120362	13/12/2012	-2	78.91	10.711
ct92-M305Pete-12	120362	13/12/2012	-2	78.93	10.76
ct92-M305Pete-12	120362	13/12/2012	-2	78.931	10.781
ct92-M305Pete-12	120362	13/12/2012	-2	78.931	10.781
ct92-M305Pete-12	120362	13/12/2012	-2	78.939	10.817
ct92-M305Pete-12	120362	13/12/2012	-2	78.931	10.729
ct92-M305Pete-12	120362	13/12/2012	-2	78.925	10.729
ct92-M305Pete-12	120362	13/12/2012	-2	78.931	10.663
ct92-M305Pete-12	120362	14/12/2012	-2	78.932	10.633
ct92-M305Pete-12	120362	14/12/2012	-2	78.921	10.638
ct92-M305Pete-12	120362	14/12/2012	-2	78.908	10.635
ct92-M305Pete-12	120362	14/12/2012	-1	78.904	10.63
ct92-M305Pete-12	120362	14/12/2012	-2	78.9	10.625

ct92-M305Pete-12	120362	14/12/2012	-2	78.903	10.598
ct92-M305Pete-12	120362	14/12/2012	-2	78.888	10.654
ct92-M305Pete-12	120362	14/12/2012	-2	78.903	10.684
ct92-M305Pete-12	120362	14/12/2012	-2	78.901	10.652
ct92-M305Pete-12	120362	14/12/2012	-2	78.906	10.697
ct92-M305Pete-12	120362	14/12/2012	-1	78.909	10.744
ct92-M305Pete-12	120362	14/12/2012	-1	78.909	10.745
ct92-M305Pete-12	120362	14/12/2012	1	78.906	10.722
ct92-M305Pete-12	120362	14/12/2012	2	78.9	10.741
ct92-M305Pete-12	120362	14/12/2012	-2	78.904	10.7
ct92-M305Pete-12	120362	14/12/2012	-2	78.904	10.691
ct92-M305Pete-12	120362	14/12/2012	-2	78.899	10.754
ct92-M305Pete-12	120362	14/12/2012	-1	78.896	10.726
ct92-M305Pete-12	120362	14/12/2012	-2	78.892	10.678
ct92-M305Pete-12	120362	14/12/2012	-2	78.9	10.649
ct92-M305Pete-12	120362	14/12/2012	-2	78.9	10.649
ct92-M305Pete-12	120362	14/12/2012	-2	78.91	10.621
ct92-M305Pete-12	120362	14/12/2012	-2	78.909	10.621
ct92-M305Pete-12	120362	14/12/2012	-2	78.908	10.61
ct92-M305Pete-12	120362	14/12/2012	-2	78.897	10.674
ct92-M305Pete-12	120362	14/12/2012	-2	78.88	10.678
ct92-M305Pete-12	120362	14/12/2012	-2	78.897	10.671
ct92-M305Pete-12	120362	14/12/2012	-2	78.917	10.633
ct92-M305Pete-12	120362	14/12/2012	-2	78.902	10.652
ct92-M305Pete-12	120362	14/12/2012	-2	78.894	10.697
ct92-M305Pete-12	120362	14/12/2012	-2	78.896	10.672
ct92-M305Pete-12	120362	14/12/2012	-2	78.898	10.672
ct92-M305Pete-12	120362	14/12/2012	-2	78.899	10.671
ct92-M305Pete-12	120362	14/12/2012	-2	78.898	10.672
ct92-M305Pete-12	120362	14/12/2012	-2	78.91	10.664
ct92-M305Pete-12	120362	14/12/2012	-2	78.911	10.665
ct92-M305Pete-12	120362	14/12/2012	-2	78.909	10.652
ct92-M305Pete-12	120362	14/12/2012	-2	78.898	10.669
ct92-M305Pete-12	120362	14/12/2012	-2	78.898	10.669
ct92-M305Pete-12	120362	14/12/2012	-2	78.897	10.67
ct92-M305Pete-12	120362	14/12/2012	-2	78.894	10.673
ct92-M305Pete-12	120362	14/12/2012	-2	78.894	10.66
ct92-M305Pete-12	120362	14/12/2012	-2	78.896	10.675
ct92-M305Pete-12	120362	14/12/2012	-2	78.897	10.678
ct92-M305Pete-12	120362	14/12/2012	-2	78.905	10.608
ct92-M305Pete-12	120362	14/12/2012	-2	78.931	10.63
ct92-M305Pete-12	120362	14/12/2012	-2	78.931	10.628
ct92-M305Pete-12	120362	14/12/2012	-2	78.921	10.665
ct92-M305Pete-12	120362	14/12/2012	-2	78.941	10.665
ct92-M305Pete-12	120362	14/12/2012	-2	78.934	10.662
ct92-M305Pete-12	120362	14/12/2012	-2	78.939	10.657
ct92-M305Pete-12	120362	14/12/2012	-2	78.932	10.663
ct92-M305Pete-12	120362	14/12/2012	-2	78.926	10.667
ct92-M305Pete-12	120362	14/12/2012	-2	78.935	10.707
ct92-M305Pete-12	120362	14/12/2012	-2	78.933	10.661
ct92-M305Pete-12	120362	14/12/2012	-2	78.936	10.74
ct92-M305Pete-12	120362	14/12/2012	-2	78.925	10.718
ct92-M305Pete-12	120362	14/12/2012	-2	78.922	10.567
ct92-M305Pete-12	120362	14/12/2012	-2	78.922	10.569
ct92-M305Pete-12	120362	14/12/2012	-2	78.924	10.574
ct92-M305Pete-12	120362	15/12/2012	-2	78.929	10.488
ct92-M305Pete-12	120362	15/12/2012	-2	78.939	10.496
ct92-M305Pete-12	120362	15/12/2012	-2	78.944	10.475
ct92-M305Pete-12	120362	15/12/2012	-2	78.94	10.481
ct92-M305Pete-12	120362	15/12/2012	-2	78.941	10.474
ct92-M305Pete-12	120362	15/12/2012	-2	78.941	10.474
ct92-M305Pete-12	120362	15/12/2012	-2	78.952	10.488
ct92-M305Pete-12	120362	15/12/2012	-2	78.952	10.49
ct92-M305Pete-12	120362	15/12/2012	-2	78.948	10.487
ct92-M305Pete-12	120362	15/12/2012	-1	78.948	10.525
ct92-M305Pete-12	120362	15/12/2012	-1	78.943	10.489
ct92-M305Pete-12	120362	15/12/2012	-1	78.959	10.452
ct92-M305Pete-12	120362	15/12/2012	-2	78.956	10.451

ct92-M305Pete-12	120362	15/12/2012	-2	78.951	10.444
ct92-M305Pete-12	120362	15/12/2012	-1	78.953	10.468
ct92-M305Pete-12	120362	15/12/2012	-2	78.944	10.452
ct92-M305Pete-12	120362	15/12/2012	-1	78.943	10.474
ct92-M305Pete-12	120362	15/12/2012	0	78.955	10.53
ct92-M305Pete-12	120362	15/12/2012	-2	78.953	10.518
ct92-M305Pete-12	120362	15/12/2012	-2	78.952	10.549
ct92-M305Pete-12	120362	15/12/2012	0	78.937	10.545
ct92-M305Pete-12	120362	15/12/2012	-2	78.952	10.548
ct92-M305Pete-12	120362	15/12/2012	-1	78.954	10.556
ct92-M305Pete-12	120362	15/12/2012	-2	78.95	10.543
ct92-M305Pete-12	120362	15/12/2012	-1	78.938	10.5
ct92-M305Pete-12	120362	15/12/2012	-2	78.939	10.504
ct92-M305Pete-12	120362	15/12/2012	-2	78.95	10.567
ct92-M305Pete-12	120362	15/12/2012	-2	78.948	10.557
ct92-M305Pete-12	120362	15/12/2012	-1	78.942	10.501
ct92-M305Pete-12	120362	15/12/2012	-2	78.939	10.49
ct92-M305Pete-12	120362	15/12/2012	-2	78.938	10.475
ct92-M305Pete-12	120362	15/12/2012	-2	78.939	10.475
ct92-M305Pete-12	120362	15/12/2012	-1	78.932	10.515
ct92-M305Pete-12	120362	15/12/2012	0	78.925	10.425
ct92-M305Pete-12	120362	15/12/2012	-1	78.948	10.509
ct92-M305Pete-12	120362	15/12/2012	-2	78.946	10.487
ct92-M305Pete-12	120362	15/12/2012	-2	78.943	10.501
ct92-M305Pete-12	120362	15/12/2012	-2	78.94	10.495
ct92-M305Pete-12	120362	15/12/2012	-2	78.93	10.448
ct92-M305Pete-12	120362	15/12/2012	-1	78.916	10.416
ct92-M305Pete-12	120362	15/12/2012	-1	78.916	10.416
ct92-M305Pete-12	120362	15/12/2012	-1	78.942	10.417
ct92-M305Pete-12	120362	15/12/2012	1	78.936	10.455
ct92-M305Pete-12	120362	15/12/2012	-2	78.94	10.476
ct92-M305Pete-12	120362	15/12/2012	-2	78.927	10.406
ct92-M305Pete-12	120362	15/12/2012	-2	78.947	10.554
ct92-M305Pete-12	120362	15/12/2012	-2	78.891	10.276
ct92-M305Pete-12	120362	15/12/2012	-1	78.916	10.446
ct92-M305Pete-12	120362	15/12/2012	-2	78.931	10.385
ct92-M305Pete-12	120362	15/12/2012	-2	78.945	10.432
ct92-M305Pete-12	120362	15/12/2012	-2	78.944	10.439
ct92-M305Pete-12	120362	15/12/2012	-2	78.946	10.45
ct92-M305Pete-12	120362	15/12/2012	-2	78.952	10.445
ct92-M305Pete-12	120362	15/12/2012	-2	78.946	10.509
ct92-M305Pete-12	120362	15/12/2012	-2	78.948	10.516
ct92-M305Pete-12	120362	15/12/2012	-2	78.958	10.4
ct92-M305Pete-12	120362	15/12/2012	-2	78.939	10.412
ct92-M305Pete-12	120362	15/12/2012	-2	78.947	10.407
ct92-M305Pete-12	120362	15/12/2012	-2	78.949	10.334
ct92-M305Pete-12	120362	15/12/2012	-2	78.945	10.35
ct92-M305Pete-12	120362	15/12/2012	-2	78.956	10.412
ct92-M305Pete-12	120362	15/12/2012	-2	78.967	10.36
ct92-M305Pete-12	120362	15/12/2012	-2	78.982	10.296
ct92-M305Pete-12	120362	16/12/2012	-2	78.929	10.334
ct92-M305Pete-12	120362	16/12/2012	-2	79.021	10.174
ct92-M305Pete-12	120362	16/12/2012	-2	79.006	10.243
ct92-M305Pete-12	120362	16/12/2012	-1	78.932	10.794
ct92-M305Pete-12	120362	16/12/2012	-2	78.931	10.786
ct92-M305Pete-12	120362	16/12/2012	-2	78.89	10.878
ct92-M305Pete-12	120362	16/12/2012	-2	78.864	11.019
ct92-M305Pete-12	120362	16/12/2012	-2	78.878	10.961
ct92-M305Pete-12	120362	16/12/2012	-2	78.914	10.773
ct92-M305Pete-12	120362	16/12/2012	-2	78.896	10.825
ct92-M305Pete-12	120362	16/12/2012	-2	78.905	10.91
ct92-M305Pete-12	120362	16/12/2012	-2	78.838	11.18
ct92-M305Pete-12	120362	16/12/2012	-1	78.851	10.838
ct92-M305Pete-12	120362	16/12/2012	-2	78.851	10.838
ct92-M305Pete-12	120362	16/12/2012	-2	78.852	10.837
ct92-M305Pete-12	120362	16/12/2012	-2	78.823	10.958
ct92-M305Pete-12	120362	16/12/2012	-2	78.888	10.711
ct92-M305Pete-12	120362	16/12/2012	-2	78.905	10.756

ct92-M305Pete-12	120362	16/12/2012	0	78.875	10.76
ct92-M305Pete-12	120362	16/12/2012	1	78.916	10.717
ct92-M305Pete-12	120362	16/12/2012	-1	78.895	10.719
ct92-M305Pete-12	120362	16/12/2012	-1	78.924	10.788
ct92-M305Pete-12	120362	16/12/2012	-1	78.91	10.796
ct92-M305Pete-12	120362	16/12/2012	-2	78.92	10.792
ct92-M305Pete-12	120362	16/12/2012	-2	78.919	10.801
ct92-M305Pete-12	120362	16/12/2012	-2	78.852	10.859
ct92-M305Pete-12	120362	16/12/2012	-2	78.903	10.745
ct92-M305Pete-12	120362	16/12/2012	-1	78.886	10.775
ct92-M305Pete-12	120362	16/12/2012	-2	78.885	10.792
ct92-M305Pete-12	120362	16/12/2012	-2	78.887	10.784
ct92-M305Pete-12	120362	16/12/2012	0	78.88	10.619
ct92-M305Pete-12	120362	16/12/2012	-2	78.876	10.643
ct92-M305Pete-12	120362	16/12/2012	-1	78.893	10.699
ct92-M305Pete-12	120362	16/12/2012	-1	78.898	10.628
ct92-M305Pete-12	120362	16/12/2012	-2	78.851	10.905
ct92-M305Pete-12	120362	16/12/2012	-2	78.887	10.682
ct92-M305Pete-12	120362	16/12/2012	-1	78.884	10.679
ct92-M305Pete-12	120362	16/12/2012	-2	78.896	10.63
ct92-M305Pete-12	120362	16/12/2012	-2	78.904	10.659
ct92-M305Pete-12	120362	16/12/2012	-1	78.901	10.669
ct92-M305Pete-12	120362	16/12/2012	-1	78.906	10.656
ct92-M305Pete-12	120362	16/12/2012	-1	78.891	10.583
ct92-M305Pete-12	120362	16/12/2012	-2	78.896	10.639
ct92-M305Pete-12	120362	16/12/2012	-2	78.879	10.689
ct92-M305Pete-12	120362	16/12/2012	1	78.898	10.632
ct92-M305Pete-12	120362	16/12/2012	-2	78.9	10.614
ct92-M305Pete-12	120362	16/12/2012	-1	78.896	10.653
ct92-M305Pete-12	120362	16/12/2012	-2	78.903	10.696
ct92-M305Pete-12	120362	16/12/2012	-1	78.908	10.621
ct92-M305Pete-12	120362	16/12/2012	-2	78.908	10.633
ct92-M305Pete-12	120362	16/12/2012	-2	78.901	10.662
ct92-M305Pete-12	120362	16/12/2012	-2	78.906	10.646
ct92-M305Pete-12	120362	16/12/2012	-2	78.871	10.509
ct92-M305Pete-12	120362	16/12/2012	-1	78.922	10.654
ct92-M305Pete-12	120362	16/12/2012	-2	78.892	10.603
ct92-M305Pete-12	120362	16/12/2012	-2	78.891	10.653
ct92-M305Pete-12	120362	16/12/2012	-2	78.906	10.645
ct92-M305Pete-12	120362	16/12/2012	-2	78.889	10.646
ct92-M305Pete-12	120362	16/12/2012	-2	78.917	10.65
ct92-M305Pete-12	120362	16/12/2012	-1	78.923	10.612
ct92-M305Pete-12	120362	16/12/2012	-2	78.92	10.605
ct92-M305Pete-12	120362	16/12/2012	-2	78.915	10.571
ct92-M305Pete-12	120362	16/12/2012	-2	78.917	10.579
ct92-M305Pete-12	120362	16/12/2012	-2	78.91	10.562
ct92-M305Pete-12	120362	16/12/2012	-2	78.933	10.571
ct92-M305Pete-12	120362	16/12/2012	-2	78.935	10.549
ct92-M305Pete-12	120362	17/12/2012	-2	78.926	10.522
ct92-M305Pete-12	120362	17/12/2012	-2	78.929	10.492
ct92-M305Pete-12	120362	17/12/2012	-2	78.933	10.534
ct92-M305Pete-12	120362	17/12/2012	-2	78.929	10.498
ct92-M305Pete-12	120362	17/12/2012	-2	78.938	10.558
ct92-M305Pete-12	120362	17/12/2012	-2	78.911	10.604
ct92-M305Pete-12	120362	17/12/2012	-2	78.906	10.611
ct92-M305Pete-12	120362	17/12/2012	-2	78.904	10.65
ct92-M305Pete-12	120362	17/12/2012	-2	78.892	10.624
ct92-M305Pete-12	120362	17/12/2012	-2	78.885	10.626
ct92-M305Pete-12	120362	17/12/2012	-2	78.886	10.62
ct92-M305Pete-12	120362	17/12/2012	-2	78.891	10.601
ct92-M305Pete-12	120362	17/12/2012	-2	78.859	10.615
ct92-M305Pete-12	120362	17/12/2012	-2	78.889	10.685
ct92-M305Pete-12	120362	17/12/2012	-2	78.879	10.684
ct92-M305Pete-12	120362	17/12/2012	-2	78.885	10.676
ct92-M305Pete-12	120362	17/12/2012	-2	78.867	10.679
ct92-M305Pete-12	120362	17/12/2012	-2	78.881	10.665
ct92-M305Pete-12	120362	17/12/2012	-2	78.922	10.677
ct92-M305Pete-12	120362	17/12/2012	-2	78.889	10.691

ct92-M305Pete-12	120362	17/12/2012	-2	78.949	10.589
ct92-M305Pete-12	120362	17/12/2012	-2	78.894	10.571
ct92-M305Pete-12	120362	17/12/2012	-2	78.947	10.595
ct92-M305Pete-12	120362	17/12/2012	-2	78.886	10.539
ct92-M305Pete-12	120362	17/12/2012	-2	78.903	10.56
ct92-M305Pete-12	120362	17/12/2012	-2	78.884	10.7
ct92-M305Pete-12	120362	17/12/2012	-2	78.887	10.691
ct92-M305Pete-12	120362	17/12/2012	-2	78.92	10.506
ct92-M305Pete-12	120362	17/12/2012	-2	78.92	10.464
ct92-M305Pete-12	120362	17/12/2012	-2	78.927	10.427
ct92-M305Pete-12	120362	17/12/2012	-2	78.948	10.471
ct92-M305Pete-12	120362	17/12/2012	0	78.985	10.39
ct92-M305Pete-12	120362	17/12/2012	-1	78.95	10.553
ct92-M305Pete-12	120362	17/12/2012	-2	78.946	10.634
ct92-M305Pete-12	120362	17/12/2012	0	78.967	10.697
ct92-M305Pete-12	120362	17/12/2012	-2	78.964	10.577
ct92-M305Pete-12	120362	17/12/2012	1	78.938	10.626
ct92-M305Pete-12	120362	17/12/2012	0	78.925	10.617
ct92-M305Pete-12	120362	17/12/2012	-2	78.944	10.616
ct92-M305Pete-12	120362	17/12/2012	-2	78.956	10.593
ct92-M305Pete-12	120362	17/12/2012	-2	78.965	10.589
ct92-M305Pete-12	120362	17/12/2012	-2	78.969	10.583
ct92-M305Pete-12	120362	17/12/2012	-2	78.944	10.606
ct92-M305Pete-12	120362	17/12/2012	-2	78.95	10.595
ct92-M305Pete-12	120362	17/12/2012	-2	78.955	10.559
ct92-M305Pete-12	120362	17/12/2012	-1	78.955	10.575
ct92-M305Pete-12	120362	17/12/2012	-2	78.966	10.486
ct92-M305Pete-12	120362	17/12/2012	-2	78.971	10.541
ct92-M305Pete-12	120362	17/12/2012	0	78.944	10.542
ct92-M305Pete-12	120362	17/12/2012	-2	78.942	10.531
ct92-M305Pete-12	120362	17/12/2012	-2	78.944	10.535
ct92-M305Pete-12	120362	17/12/2012	-2	78.944	10.52
ct92-M305Pete-12	120362	17/12/2012	-1	78.94	10.456
ct92-M305Pete-12	120362	17/12/2012	-2	78.943	10.484
ct92-M305Pete-12	120362	17/12/2012	-2	78.943	10.482
ct92-M305Pete-12	120362	17/12/2012	-2	78.949	10.454
ct92-M305Pete-12	120362	17/12/2012	-2	78.94	10.466
ct92-M305Pete-12	120362	17/12/2012	-2	78.947	10.425
ct92-M305Pete-12	120362	17/12/2012	-2	78.951	10.458
ct92-M305Pete-12	120362	17/12/2012	-2	78.944	10.507
ct92-M305Pete-12	120362	17/12/2012	-1	78.941	10.556
ct92-M305Pete-12	120362	17/12/2012	-1	78.961	10.377
ct92-M305Pete-12	120362	17/12/2012	-2	78.956	10.587
ct92-M305Pete-12	120362	17/12/2012	-1	78.939	10.588
ct92-M305Pete-12	120362	17/12/2012	-1	78.94	10.589
ct92-M305Pete-12	120362	17/12/2012	-2	78.939	10.577
ct92-M305Pete-12	120362	17/12/2012	-1	78.947	10.425
ct92-M305Pete-12	120362	17/12/2012	-2	78.938	10.51
ct92-M305Pete-12	120362	17/12/2012	-2	78.946	10.437
ct92-M305Pete-12	120362	17/12/2012	-2	78.945	10.413
ct92-M305Pete-12	120362	17/12/2012	-1	78.941	10.449
ct92-M305Pete-12	120362	17/12/2012	-1	78.956	10.586
ct92-M305Pete-12	120362	18/12/2012	-2	78.932	10.56
ct92-M305Pete-12	120362	18/12/2012	-2	78.94	10.419
ct92-M305Pete-12	120362	18/12/2012	-1	78.94	10.52
ct92-M305Pete-12	120362	18/12/2012	-2	78.939	10.492
ct92-M305Pete-12	120362	18/12/2012	-2	78.94	10.502
ct92-M305Pete-12	120362	18/12/2012	-1	78.944	10.667
ct92-M305Pete-12	120362	18/12/2012	-2	78.942	10.668
ct92-M305Pete-12	120362	18/12/2012	-1	78.926	10.443
ct92-M305Pete-12	120362	18/12/2012	-2	78.929	10.51
ct92-M305Pete-12	120362	18/12/2012	-2	78.924	10.459
ct92-M305Pete-12	120362	18/12/2012	-2	78.929	10.516
ct92-M305Pete-12	120362	18/12/2012	-2	78.93	10.527
ct92-M305Pete-12	120362	18/12/2012	-2	78.929	10.522
ct92-M305Pete-12	120362	18/12/2012	-2	78.915	10.531
ct92-M305Pete-12	120362	18/12/2012	-1	78.919	10.666
ct92-M305Pete-12	120362	18/12/2012	0	78.918	10.675

ct92-M305Pete-12	120362	18/12/2012	-2	78.917	10.687
ct92-M305Pete-12	120362	18/12/2012	-2	78.916	10.7
ct92-M305Pete-12	120362	18/12/2012	-2	78.916	10.718
ct92-M305Pete-12	120362	18/12/2012	-2	78.9	10.807
ct92-M305Pete-12	120362	18/12/2012	-1	78.931	10.669
ct92-M305Pete-12	120362	18/12/2012	-2	78.931	10.669
ct92-M305Pete-12	120362	18/12/2012	-2	78.939	10.684
ct92-M305Pete-12	120362	18/12/2012	-2	78.936	10.702
ct92-M305Pete-12	120362	18/12/2012	-2	78.939	10.704
ct92-M305Pete-12	120362	18/12/2012	-2	78.94	10.657
ct92-M305Pete-12	120362	18/12/2012	-2	78.945	10.634
ct92-M305Pete-12	120362	18/12/2012	-2	78.942	10.645
ct92-M305Pete-12	120362	18/12/2012	-2	78.956	10.686
ct92-M305Pete-12	120362	18/12/2012	-2	78.937	10.66
ct92-M305Pete-12	120362	18/12/2012	-2	78.937	10.646
ct92-M305Pete-12	120362	18/12/2012	-1	78.935	10.696
ct92-M305Pete-12	120362	18/12/2012	-2	78.954	10.671
ct92-M305Pete-12	120362	18/12/2012	-2	78.96	10.659
ct92-M305Pete-12	120362	18/12/2012	-2	78.956	10.672
ct92-M305Pete-12	120362	18/12/2012	-2	78.949	10.677
ct92-M305Pete-12	120362	18/12/2012	-2	78.961	10.679
ct92-M305Pete-12	120362	18/12/2012	-1	78.949	10.724
ct92-M305Pete-12	120362	18/12/2012	-2	78.953	10.664
ct92-M305Pete-12	120362	18/12/2012	-2	78.952	10.676
ct92-M305Pete-12	120362	18/12/2012	-2	78.949	10.719
ct92-M305Pete-12	120362	18/12/2012	-2	78.947	10.725
ct92-M305Pete-12	120362	18/12/2012	-1	78.938	10.651
ct92-M305Pete-12	120362	18/12/2012	-2	78.928	10.588
ct92-M305Pete-12	120362	18/12/2012	-2	78.929	10.573
ct92-M305Pete-12	120362	18/12/2012	-2	78.93	10.576
ct92-M305Pete-12	120362	18/12/2012	-2	78.927	10.581
ct92-M305Pete-12	120362	18/12/2012	-2	78.948	10.758
ct92-M305Pete-12	120362	18/12/2012	-2	78.949	10.763
ct92-M305Pete-12	120362	18/12/2012	-2	78.947	10.742
ct92-M305Pete-12	120362	18/12/2012	-1	78.922	10.604
ct92-M305Pete-12	120362	18/12/2012	-1	78.92	10.605
ct92-M305Pete-12	120362	18/12/2012	-1	78.921	10.626
ct92-M305Pete-12	120362	18/12/2012	-2	78.922	10.644
ct92-M305Pete-12	120362	18/12/2012	-2	78.948	10.632
ct92-M305Pete-12	120362	18/12/2012	-2	78.921	10.635
ct92-M305Pete-12	120362	18/12/2012	-2	78.917	10.645
ct92-M305Pete-12	120362	18/12/2012	-2	78.917	10.649
ct92-M305Pete-12	120362	18/12/2012	-2	78.931	10.676
ct92-M305Pete-12	120362	18/12/2012	-2	78.931	10.675
ct92-M305Pete-12	120362	18/12/2012	-2	78.93	10.669
ct92-M305Pete-12	120362	18/12/2012	-2	78.923	10.643
ct92-M305Pete-12	120362	18/12/2012	-2	78.921	10.637
ct92-M305Pete-12	120362	18/12/2012	-2	78.926	10.588
ct92-M305Pete-12	120362	18/12/2012	-2	78.922	10.665
ct92-M305Pete-12	120362	18/12/2012	-2	78.92	10.654
ct92-M305Pete-12	120362	19/12/2012	-2	78.917	10.589
ct92-M305Pete-12	120362	19/12/2012	-2	78.921	10.65
ct92-M305Pete-12	120362	19/12/2012	-2	78.923	10.657
ct92-M305Pete-12	120362	19/12/2012	-1	78.915	10.694
ct92-M305Pete-12	120362	19/12/2012	-1	78.915	10.7
ct92-M305Pete-12	120362	19/12/2012	-2	78.918	10.699
ct92-M305Pete-12	120362	19/12/2012	-2	78.901	10.653
ct92-M305Pete-12	120362	19/12/2012	-2	78.907	10.706
ct92-M305Pete-12	120362	19/12/2012	-2	78.907	10.705
ct92-M305Pete-12	120362	19/12/2012	-2	78.905	10.697
ct92-M305Pete-12	120362	19/12/2012	-2	78.904	10.713
ct92-M305Pete-12	120362	19/12/2012	-2	78.916	10.69
ct92-M305Pete-12	120362	19/12/2012	-2	78.9	10.692
ct92-M305Pete-12	120362	19/12/2012	-2	78.902	10.697
ct92-M305Pete-12	120362	19/12/2012	-2	78.9	10.692
ct92-M305Pete-12	120362	19/12/2012	-2	78.904	10.729
ct92-M305Pete-12	120362	19/12/2012	-2	78.906	10.733
ct92-M305Pete-12	120362	19/12/2012	-1	78.915	10.723

ct92-M305Pete-12	120362	19/12/2012	-2	78.91	10.745
ct92-M305Pete-12	120362	19/12/2012	-2	78.902	10.754
ct92-M305Pete-12	120362	19/12/2012	0	78.908	10.74
ct92-M305Pete-12	120362	19/12/2012	-2	78.909	10.737
ct92-M305Pete-12	120362	19/12/2012	-2	78.91	10.729
ct92-M305Pete-12	120362	19/12/2012	-2	78.91	10.727
ct92-M305Pete-12	120362	19/12/2012	-2	78.91	10.724
ct92-M305Pete-12	120362	19/12/2012	-2	78.91	10.724
ct92-M305Pete-12	120362	19/12/2012	-2	78.908	10.688
ct92-M305Pete-12	120362	19/12/2012	-1	78.924	10.76
ct92-M305Pete-12	120362	19/12/2012	-2	78.926	10.75
ct92-M305Pete-12	120362	19/12/2012	-2	78.922	10.745
ct92-M305Pete-12	120362	19/12/2012	-2	78.915	10.643
ct92-M305Pete-12	120362	19/12/2012	-2	78.917	10.643
ct92-M305Pete-12	120362	19/12/2012	-2	78.917	10.62
ct92-M305Pete-12	120362	19/12/2012	-2	78.931	10.66
ct92-M305Pete-12	120362	19/12/2012	-2	78.93	10.683
ct92-M305Pete-12	120362	19/12/2012	-2	78.932	10.664
ct92-M305Pete-12	120362	19/12/2012	-2	78.941	10.647
ct92-M305Pete-12	120362	19/12/2012	-2	78.943	10.636
ct92-M305Pete-12	120362	19/12/2012	-2	78.947	10.583
ct92-M305Pete-12	120362	19/12/2012	-2	78.944	10.602
ct92-M305Pete-12	120362	19/12/2012	-1	78.937	10.597
ct92-M305Pete-12	120362	19/12/2012	-1	78.937	10.597
ct92-M305Pete-12	120362	19/12/2012	-1	78.943	10.541
ct92-M305Pete-12	120362	19/12/2012	-2	78.943	10.504
ct92-M305Pete-12	120362	19/12/2012	-2	78.944	10.513
ct92-M305Pete-12	120362	19/12/2012	-2	78.943	10.49
ct92-M305Pete-12	120362	19/12/2012	1	78.941	10.441
ct92-M305Pete-12	120362	19/12/2012	-1	78.942	10.425
ct92-M305Pete-12	120362	19/12/2012	-1	78.928	10.395
ct92-M305Pete-12	120362	19/12/2012	-2	78.931	10.419
ct92-M305Pete-12	120362	19/12/2012	-2	78.924	10.473
ct92-M305Pete-12	120362	19/12/2012	-1	78.93	10.497
ct92-M305Pete-12	120362	19/12/2012	-2	78.929	10.437
ct92-M305Pete-12	120362	19/12/2012	-2	78.934	10.566
ct92-M305Pete-12	120362	19/12/2012	-2	78.924	10.523
ct92-M305Pete-12	120362	19/12/2012	-2	78.931	10.552
ct92-M305Pete-12	120362	19/12/2012	-2	78.923	10.641
ct92-M305Pete-12	120362	19/12/2012	-2	78.917	10.68
ct92-M305Pete-12	120362	19/12/2012	-2	78.92	10.628
ct92-M305Pete-12	120362	19/12/2012	-2	78.934	10.608
ct92-M305Pete-12	120362	19/12/2012	-2	78.935	10.618
ct92-M305Pete-12	120362	19/12/2012	-2	78.935	10.613
ct92-M305Pete-12	120362	19/12/2012	-2	78.933	10.571
ct92-M305Pete-12	120362	19/12/2012	-2	78.929	10.562
ct92-M305Pete-12	120362	19/12/2012	-2	78.929	10.565
ct92-M305Pete-12	120362	19/12/2012	-2	78.919	10.611
ct92-M305Pete-12	120362	19/12/2012	-2	78.914	10.611
ct92-M305Pete-12	120362	19/12/2012	-1	78.929	10.551
ct92-M305Pete-12	120362	19/12/2012	-1	78.928	10.557
ct92-M305Pete-12	120362	19/12/2012	-1	78.928	10.544
ct92-M305Pete-12	120362	19/12/2012	-2	78.933	10.531
ct92-M305Pete-12	120362	19/12/2012	-2	78.93	10.59
ct92-M305Pete-12	120362	20/12/2012	-2	78.914	10.641
ct92-M305Pete-12	120362	20/12/2012	-2	78.92	10.675
ct92-M305Pete-12	120362	20/12/2012	-2	78.915	10.636
ct92-M305Pete-12	120362	20/12/2012	-2	78.912	10.672
ct92-M305Pete-12	120362	20/12/2012	0	78.928	10.863
ct92-M305Pete-12	120362	20/12/2012	-2	78.902	10.633
ct92-M305Pete-12	120362	20/12/2012	-2	78.92	10.825
ct92-M305Pete-12	120362	20/12/2012	-2	78.92	10.824
ct92-M305Pete-12	120362	20/12/2012	-2	78.915	10.826
ct92-M305Pete-12	120362	20/12/2012	-2	78.921	10.815
ct92-M305Pete-12	120362	20/12/2012	-1	78.925	10.797
ct92-M305Pete-12	120362	20/12/2012	-2	78.923	10.812
ct92-M305Pete-12	120362	20/12/2012	-2	78.922	10.707
ct92-M305Pete-12	120362	20/12/2012	-1	78.933	10.7

ct92-M305Pete-12	120362	20/12/2012	-2	78.93	10.688
ct92-M305Pete-12	120362	20/12/2012	-2	78.924	10.747
ct92-M305Pete-12	120362	20/12/2012	-2	78.937	10.712
ct92-M305Pete-12	120362	20/12/2012	-2	78.937	10.701
ct92-M305Pete-12	120362	20/12/2012	-2	78.937	10.701
ct92-M305Pete-12	120362	20/12/2012	-2	78.938	10.687
ct92-M305Pete-12	120362	20/12/2012	-1	78.939	10.645
ct92-M305Pete-12	120362	20/12/2012	-2	78.937	10.637
ct92-M305Pete-12	120362	20/12/2012	-2	78.942	10.651
ct92-M305Pete-12	120362	20/12/2012	-2	78.941	10.613
ct92-M305Pete-12	120362	20/12/2012	-1	78.949	10.54
ct92-M305Pete-12	120362	20/12/2012	-2	78.945	10.579
ct92-M305Pete-12	120362	20/12/2012	-2	78.948	10.529
ct92-M305Pete-12	120362	20/12/2012	-1	78.943	10.532
ct92-M305Pete-12	120362	20/12/2012	-2	78.949	10.523
ct92-M305Pete-12	120362	20/12/2012	-2	78.941	10.563
ct92-M305Pete-12	120362	20/12/2012	-2	78.942	10.58
ct92-M305Pete-12	120362	20/12/2012	-2	78.933	10.606
ct92-M305Pete-12	120362	20/12/2012	-2	78.933	10.611
ct92-M305Pete-12	120362	20/12/2012	-2	78.939	10.597
ct92-M305Pete-12	120362	20/12/2012	-2	78.939	10.598
ct92-M305Pete-12	120362	20/12/2012	-1	78.93	10.655
ct92-M305Pete-12	120362	20/12/2012	-2	78.93	10.575
ct92-M305Pete-12	120362	20/12/2012	-2	78.931	10.58
ct92-M305Pete-12	120362	20/12/2012	-2	78.932	10.628
ct92-M305Pete-12	120362	20/12/2012	0	78.944	10.641
ct92-M305Pete-12	120362	20/12/2012	-1	78.947	10.646
ct92-M305Pete-12	120362	20/12/2012	-2	78.934	10.566
ct92-M305Pete-12	120362	20/12/2012	-2	78.944	10.592
ct92-M305Pete-12	120362	20/12/2012	-2	78.945	10.593
ct92-M305Pete-12	120362	20/12/2012	-2	78.944	10.59
ct92-M305Pete-12	120362	20/12/2012	-2	78.945	10.612
ct92-M305Pete-12	120362	20/12/2012	-2	78.935	10.632
ct92-M305Pete-12	120362	20/12/2012	-2	78.929	10.64
ct92-M305Pete-12	120362	20/12/2012	-2	78.933	10.655
ct92-M305Pete-12	120362	20/12/2012	-1	78.935	10.655
ct92-M305Pete-12	120362	20/12/2012	-2	78.935	10.656
ct92-M305Pete-12	120362	20/12/2012	-2	78.933	10.639
ct92-M305Pete-12	120362	20/12/2012	-1	78.939	10.698
ct92-M305Pete-12	120362	20/12/2012	-2	78.937	10.706
ct92-M305Pete-12	120362	20/12/2012	-1	78.936	10.754
ct92-M305Pete-12	120362	20/12/2012	-2	78.936	10.725
ct92-M305Pete-12	120362	20/12/2012	-2	78.931	10.76
ct92-M305Pete-12	120362	20/12/2012	-2	78.93	10.747
ct92-M305Pete-12	120362	20/12/2012	-2	78.929	10.751
ct92-M305Pete-12	120362	20/12/2012	-2	78.93	10.746
ct92-M305Pete-12	120362	20/12/2012	-2	78.928	10.755
ct92-M305Pete-12	120362	20/12/2012	-1	78.917	10.703
ct92-M305Pete-12	120362	20/12/2012	-1	78.916	10.709
ct92-M305Pete-12	120362	20/12/2012	-2	78.917	10.708
ct92-M305Pete-12	120362	20/12/2012	-2	78.915	10.706
ct92-M305Pete-12	120362	20/12/2012	-1	78.932	10.652
ct92-M305Pete-12	120362	21/12/2012	-2	78.921	10.658
ct92-M305Pete-12	120362	21/12/2012	-2	78.922	10.662
ct92-M305Pete-12	120362	21/12/2012	-2	78.921	10.69
ct92-M305Pete-12	120362	21/12/2012	-1	78.916	10.661
ct92-M305Pete-12	120362	21/12/2012	-2	78.916	10.662
ct92-M305Pete-12	120362	21/12/2012	-2	78.903	10.663
ct92-M305Pete-12	120362	21/12/2012	-2	78.905	10.643
ct92-M305Pete-12	120362	21/12/2012	0	78.909	10.63
ct92-M305Pete-12	120362	21/12/2012	-2	78.909	10.631
ct92-M305Pete-12	120362	21/12/2012	-2	78.903	10.626
ct92-M305Pete-12	120362	21/12/2012	-2	78.903	10.63
ct92-M305Pete-12	120362	21/12/2012	-2	78.909	10.624
ct92-M305Pete-12	120362	21/12/2012	-1	78.906	10.703
ct92-M305Pete-12	120362	21/12/2012	-1	78.933	10.95
ct92-M305Pete-12	120362	21/12/2012	-2	78.873	10.906
ct92-M305Pete-12	120362	21/12/2012	-2	78.876	10.914

ct92-M305Pete-12	120362	21/12/2012	-2	78.904	10.744
ct92-M305Pete-12	120362	21/12/2012	-2	78.909	10.751
ct92-M305Pete-12	120362	21/12/2012	-2	78.908	10.705
ct92-M305Pete-12	120362	21/12/2012	-2	78.901	10.626
ct92-M305Pete-12	120362	21/12/2012	-2	78.904	10.646
ct92-M305Pete-12	120362	21/12/2012	-1	78.903	10.681
ct92-M305Pete-12	120362	21/12/2012	-2	78.864	10.754
ct92-M305Pete-12	120362	21/12/2012	-2	78.891	10.588
ct92-M305Pete-12	120362	21/12/2012	-2	78.895	10.593
ct92-M305Pete-12	120362	21/12/2012	1	78.922	10.613
ct92-M305Pete-12	120362	21/12/2012	-2	78.885	10.57
ct92-M305Pete-12	120362	21/12/2012	-2	78.922	10.584
ct92-M305Pete-12	120362	21/12/2012	-2	78.923	10.535
ct92-M305Pete-12	120362	21/12/2012	-2	78.888	10.579
ct92-M305Pete-12	120362	21/12/2012	-2	78.919	10.487
ct92-M305Pete-12	120362	21/12/2012	-1	78.859	10.626
ct92-M305Pete-12	120362	21/12/2012	-2	78.855	10.621
ct92-M305Pete-12	120362	21/12/2012	-2	78.858	10.622
ct92-M305Pete-12	120362	21/12/2012	-2	78.857	10.594
ct92-M305Pete-12	120362	21/12/2012	-2	78.88	10.575
ct92-M305Pete-12	120362	21/12/2012	-2	78.855	10.529
ct92-M305Pete-12	120362	21/12/2012	-2	78.861	10.531
ct92-M305Pete-12	120362	21/12/2012	-2	78.832	10.599
ct92-M305Pete-12	120362	21/12/2012	-2	78.912	10.535
ct92-M305Pete-12	120362	21/12/2012	-2	78.898	10.39
ct92-M305Pete-12	120362	21/12/2012	-2	78.88	10.61
ct92-M305Pete-12	120362	21/12/2012	-2	78.892	10.439
ct92-M305Pete-12	120362	21/12/2012	-2	78.89	10.427
ct92-M305Pete-12	120362	21/12/2012	-2	78.891	10.43
ct92-M305Pete-12	120362	21/12/2012	-2	78.895	10.417
ct92-M305Pete-12	120362	21/12/2012	-1	78.888	10.379
ct92-M305Pete-12	120362	21/12/2012	-2	78.878	10.359
ct92-M305Pete-12	120362	21/12/2012	-2	78.881	10.368
ct92-M305Pete-12	120362	21/12/2012	-2	78.898	10.397
ct92-M305Pete-12	120362	21/12/2012	-2	78.887	10.42
ct92-M305Pete-12	120362	21/12/2012	-2	78.895	10.462
ct92-M305Pete-12	120362	21/12/2012	-2	78.883	10.576
ct92-M305Pete-12	120362	21/12/2012	-1	78.906	10.643
ct92-M305Pete-12	120362	21/12/2012	-2	78.895	10.51
ct92-M305Pete-12	120362	21/12/2012	-2	78.906	10.6
ct92-M305Pete-12	120362	21/12/2012	-2	78.904	10.585
ct92-M305Pete-12	120362	21/12/2012	-2	78.904	10.617
ct92-M305Pete-12	120362	21/12/2012	-2	78.9	10.612
ct92-M305Pete-12	120362	21/12/2012	-2	78.914	10.551
ct92-M305Pete-12	120362	21/12/2012	-2	78.913	10.556
ct92-M305Pete-12	120362	21/12/2012	-2	78.903	10.547
ct92-M305Pete-12	120362	21/12/2012	-1	78.886	10.565
ct92-M305Pete-12	120362	21/12/2012	-2	78.903	10.628
ct92-M305Pete-12	120362	21/12/2012	-2	78.909	10.624
ct92-M305Pete-12	120362	21/12/2012	-2	78.907	10.623
ct92-M305Pete-12	120362	21/12/2012	-2	78.908	10.613
ct92-M305Pete-12	120362	21/12/2012	-2	78.91	10.611
ct92-M305Pete-12	120362	21/12/2012	-2	78.92	10.427
ct92-M305Pete-12	120362	21/12/2012	-2	78.921	10.418
ct92-M305Pete-12	120362	21/12/2012	-2	78.932	10.406
ct92-M305Pete-12	120362	21/12/2012	-2	78.912	10.405
ct92-M305Pete-12	120362	21/12/2012	-2	78.911	10.568
ct92-M305Pete-12	120362	21/12/2012	-2	78.884	10.492
ct92-M305Pete-12	120362	22/12/2012	-2	78.876	10.494
ct92-M305Pete-12	120362	22/12/2012	-2	78.876	10.468
ct92-M305Pete-12	120362	22/12/2012	-2	78.873	10.482
ct92-M305Pete-12	120362	22/12/2012	-2	78.913	10.564
ct92-M305Pete-12	120362	22/12/2012	-2	78.913	10.569
ct92-M305Pete-12	120362	22/12/2012	-2	78.909	10.438
ct92-M305Pete-12	120362	22/12/2012	-2	78.937	10.512
ct92-M305Pete-12	120362	22/12/2012	-2	78.932	10.604
ct92-M305Pete-12	120362	22/12/2012	0	78.926	10.599
ct92-M305Pete-12	120362	22/12/2012	-2	78.923	10.575

ct92-M305Pete-12	120362	22/12/2012	-2	78.912	10.552
ct92-M305Pete-12	120362	22/12/2012	-2	78.919	10.585
ct92-M305Pete-12	120362	22/12/2012	-2	78.914	10.587
ct92-M305Pete-12	120362	22/12/2012	-2	78.914	10.568
ct92-M305Pete-12	120362	22/12/2012	-2	78.913	10.565
ct92-M305Pete-12	120362	22/12/2012	-2	78.906	10.542
ct92-M305Pete-12	120362	22/12/2012	-2	78.901	10.542
ct92-M305Pete-12	120362	22/12/2012	-2	78.902	10.55
ct92-M305Pete-12	120362	22/12/2012	-1	78.908	10.486
ct92-M305Pete-12	120362	22/12/2012	-2	78.924	10.631
ct92-M305Pete-12	120362	22/12/2012	-2	78.886	10.559
ct92-M305Pete-12	120362	22/12/2012	-2	78.914	10.633
ct92-M305Pete-12	120362	22/12/2012	-2	78.904	10.607
ct92-M305Pete-12	120362	22/12/2012	-2	78.913	10.573
ct92-M305Pete-12	120362	22/12/2012	-2	78.899	10.605
ct92-M305Pete-12	120362	22/12/2012	0	78.915	10.623
ct92-M305Pete-12	120362	22/12/2012	-2	78.915	10.62
ct92-M305Pete-12	120362	22/12/2012	1	78.91	10.615
ct92-M305Pete-12	120362	22/12/2012	0	78.918	10.628
ct92-M305Pete-12	120362	22/12/2012	-2	78.909	10.697
ct92-M305Pete-12	120362	22/12/2012	-2	78.909	10.698
ct92-M305Pete-12	120362	22/12/2012	-2	78.909	10.619
ct92-M305Pete-12	120362	22/12/2012	-1	78.903	10.62
ct92-M305Pete-12	120362	22/12/2012	0	78.915	10.646
ct92-M305Pete-12	120362	22/12/2012	-2	78.91	10.6
ct92-M305Pete-12	120362	22/12/2012	-1	78.9	10.63
ct92-M305Pete-12	120362	22/12/2012	-2	78.906	10.664
ct92-M305Pete-12	120362	22/12/2012	-1	78.903	10.657
ct92-M305Pete-12	120362	22/12/2012	1	78.924	10.69
ct92-M305Pete-12	120362	22/12/2012	0	78.916	10.695
ct92-M305Pete-12	120362	22/12/2012	0	78.914	10.686
ct92-M305Pete-12	120362	22/12/2012	-2	78.914	10.685
ct92-M305Pete-12	120362	22/12/2012	-1	78.902	10.688
ct92-M305Pete-12	120362	22/12/2012	-2	78.914	10.601
ct92-M305Pete-12	120362	22/12/2012	-2	78.914	10.682
ct92-M305Pete-12	120362	22/12/2012	-2	78.914	10.631
ct92-M305Pete-12	120362	22/12/2012	-1	78.914	10.612
ct92-M305Pete-12	120362	22/12/2012	-2	78.914	10.363
ct92-M305Pete-12	120362	22/12/2012	-2	78.917	10.369
ct92-M305Pete-12	120362	22/12/2012	-2	78.916	10.354
ct92-M305Pete-12	120362	22/12/2012	-2	78.917	10.35
ct92-M305Pete-12	120362	22/12/2012	-2	78.917	10.352
ct92-M305Pete-12	120362	22/12/2012	-1	78.931	10.311
ct92-M305Pete-12	120362	22/12/2012	-2	78.93	10.301
ct92-M305Pete-12	120362	22/12/2012	-2	78.935	10.387
ct92-M305Pete-12	120362	22/12/2012	-2	78.935	10.383
ct92-M305Pete-12	120362	22/12/2012	-1	78.935	10.451
ct92-M305Pete-12	120362	22/12/2012	-2	78.941	10.475
ct92-M305Pete-12	120362	22/12/2012	-2	78.941	10.473
ct92-M305Pete-12	120362	22/12/2012	-2	78.94	10.468
ct92-M305Pete-12	120362	22/12/2012	-2	78.94	10.447
ct92-M305Pete-12	120362	23/12/2012	-2	78.938	10.455
ct92-M305Pete-12	120362	23/12/2012	-2	78.938	10.459
ct92-M305Pete-12	120362	23/12/2012	-2	78.936	10.446
ct92-M305Pete-12	120362	23/12/2012	-1	78.951	10.497
ct92-M305Pete-12	120362	23/12/2012	-2	78.952	10.495
ct92-M305Pete-12	120362	23/12/2012	-2	78.943	10.484
ct92-M305Pete-12	120362	23/12/2012	-2	78.953	10.504
ct92-M305Pete-12	120362	23/12/2012	-2	78.946	10.486
ct92-M305Pete-12	120362	23/12/2012	-2	78.95	10.506
ct92-M305Pete-12	120362	23/12/2012	-2	78.948	10.49
ct92-M305Pete-12	120362	23/12/2012	-1	78.939	10.596
ct92-M305Pete-12	120362	23/12/2012	-2	78.939	10.589
ct92-M305Pete-12	120362	23/12/2012	-2	78.952	10.515
ct92-M305Pete-12	120362	23/12/2012	-2	78.952	10.515
ct92-M305Pete-12	120362	23/12/2012	-2	78.952	10.516
ct92-M305Pete-12	120362	23/12/2012	-2	78.953	10.521
ct92-M305Pete-12	120362	23/12/2012	-2	78.953	10.522

ct92-M305Pete-12	120362	23/12/2012	-2	78.951	10.522
ct92-M305Pete-12	120362	23/12/2012	-2	78.932	10.541
ct92-M305Pete-12	120362	23/12/2012	-2	78.933	10.541
ct92-M305Pete-12	120362	23/12/2012	-2	78.932	10.534
ct92-M305Pete-12	120362	23/12/2012	-2	78.929	10.536
ct92-M305Pete-12	120362	23/12/2012	-2	78.951	10.518
ct92-M305Pete-12	120362	23/12/2012	-2	78.947	10.541
ct92-M305Pete-12	120362	23/12/2012	-2	78.918	10.552
ct92-M305Pete-12	120362	23/12/2012	-2	78.921	10.539
ct92-M305Pete-12	120362	23/12/2012	-2	78.923	10.515
ct92-M305Pete-12	120362	23/12/2012	-2	78.928	10.515
ct92-M305Pete-12	120362	23/12/2012	-2	78.928	10.475
ct92-M305Pete-12	120362	23/12/2012	-2	78.93	10.497
ct92-M305Pete-12	120362	23/12/2012	-2	78.933	10.504
ct92-M305Pete-12	120362	23/12/2012	-2	78.933	10.505
ct92-M305Pete-12	120362	23/12/2012	0	78.951	10.562
ct92-M305Pete-12	120362	23/12/2012	-2	78.944	10.546
ct92-M305Pete-12	120362	23/12/2012	-2	78.945	10.582
ct92-M305Pete-12	120362	23/12/2012	-1	78.946	10.566
ct92-M305Pete-12	120362	23/12/2012	1	78.949	10.526
ct92-M305Pete-12	120362	23/12/2012	-1	78.944	10.553
ct92-M305Pete-12	120362	23/12/2012	-2	78.95	10.527
ct92-M305Pete-12	120362	23/12/2012	-2	78.941	10.538
ct92-M305Pete-12	120362	23/12/2012	1	78.945	10.587
ct92-M305Pete-12	120362	23/12/2012	-2	78.943	10.586
ct92-M305Pete-12	120362	23/12/2012	-2	78.943	10.588
ct92-M305Pete-12	120362	23/12/2012	-2	78.944	10.588
ct92-M305Pete-12	120362	23/12/2012	-2	78.944	10.582
ct92-M305Pete-12	120362	23/12/2012	-1	78.945	10.621
ct92-M305Pete-12	120362	23/12/2012	0	78.942	10.646
ct92-M305Pete-12	120362	23/12/2012	-2	78.948	10.643
ct92-M305Pete-12	120362	23/12/2012	-2	78.947	10.655
ct92-M305Pete-12	120362	23/12/2012	0	78.907	10.334
ct92-M305Pete-12	120362	23/12/2012	-1	78.934	10.62
ct92-M305Pete-12	120362	23/12/2012	-2	78.948	10.669
ct92-M305Pete-12	120362	23/12/2012	2	78.933	10.738
ct92-M305Pete-12	120362	23/12/2012	0	78.926	10.74
ct92-M305Pete-12	120362	23/12/2012	1	78.931	10.752
ct92-M305Pete-12	120362	24/12/2012	-2	78.917	10.9
ct92-M305Pete-12	120362	24/12/2012	-2	78.957	10.616
ct92-M305Pete-12	120362	24/12/2012	-2	78.96	10.606
ct92-M305Pete-12	120362	24/12/2012	-2	78.956	10.667
ct92-M305Pete-12	120362	24/12/2012	-2	78.954	10.673
ct92-M305Pete-12	120362	24/12/2012	-2	78.955	10.675
ct92-M305Pete-12	120362	24/12/2012	-2	78.952	10.652
ct92-M305Pete-12	120362	24/12/2012	-2	78.94	10.665
ct92-M305Pete-12	120362	24/12/2012	-2	78.939	10.66
ct92-M305Pete-12	120362	24/12/2012	-2	78.94	10.659
ct92-M305Pete-12	120362	24/12/2012	-2	78.929	10.7
ct92-M305Pete-12	120362	24/12/2012	-2	78.928	10.688
ct92-M305Pete-12	120362	24/12/2012	-2	78.924	10.654
ct92-M305Pete-12	120362	24/12/2012	-2	78.928	10.628
ct92-M305Pete-12	120362	24/12/2012	-2	78.933	10.566
ct92-M305Pete-12	120362	24/12/2012	-2	78.936	10.573
ct92-M305Pete-12	120362	24/12/2012	-1	78.913	10.607
ct92-M305Pete-12	120362	24/12/2012	-2	78.914	10.613
ct92-M305Pete-12	120362	24/12/2012	-2	78.927	10.638
ct92-M305Pete-12	120362	24/12/2012	-2	78.903	10.666
ct92-M305Pete-12	120362	24/12/2012	-2	78.92	10.649
ct92-M305Pete-12	120362	24/12/2012	-2	78.918	10.649
ct92-M305Pete-12	120362	24/12/2012	-2	78.918	10.652
ct92-M305Pete-12	120362	24/12/2012	-1	78.921	10.611
ct92-M305Pete-12	120362	24/12/2012	-2	78.917	10.571
ct92-M305Pete-12	120362	24/12/2012	-1	78.911	10.564
ct92-M305Pete-12	120362	24/12/2012	-2	78.911	10.559
ct92-M305Pete-12	120362	24/12/2012	-2	78.917	10.58
ct92-M305Pete-12	120362	24/12/2012	-1	78.917	10.514
ct92-M305Pete-12	120362	24/12/2012	-1	78.893	10.564

ct92-M305Pete-12	120362	24/12/2012	-2	78.907	10.57
ct92-M305Pete-12	120362	24/12/2012	0	78.944	10.82
ct92-M305Pete-12	120362	24/12/2012	-2	78.933	10.794
ct92-M305Pete-12	120362	24/12/2012	-2	78.91	10.536
ct92-M305Pete-12	120362	24/12/2012	-2	78.904	10.543
ct92-M305Pete-12	120362	24/12/2012	-1	78.906	10.601
ct92-M305Pete-12	120362	24/12/2012	-2	78.897	10.603
ct92-M305Pete-12	120362	24/12/2012	-2	78.902	10.509
ct92-M305Pete-12	120362	24/12/2012	-2	78.905	10.567
ct92-M305Pete-12	120362	24/12/2012	-1	78.905	10.574
ct92-M305Pete-12	120362	24/12/2012	-2	78.909	10.636
ct92-M305Pete-12	120362	24/12/2012	1	78.898	10.695
ct92-M305Pete-12	120362	24/12/2012	-2	78.908	10.63
ct92-M305Pete-12	120362	24/12/2012	-1	78.919	10.577
ct92-M305Pete-12	120362	24/12/2012	-2	78.917	10.551
ct92-M305Pete-12	120362	24/12/2012	-2	78.917	10.555
ct92-M305Pete-12	120362	24/12/2012	-1	78.915	10.55
ct92-M305Pete-12	120362	24/12/2012	-1	78.915	10.55
ct92-M305Pete-12	120362	24/12/2012	-2	78.914	10.532
ct92-M305Pete-12	120362	24/12/2012	-1	78.908	10.582
ct92-M305Pete-12	120362	24/12/2012	-1	78.905	10.585
ct92-M305Pete-12	120362	24/12/2012	-2	78.909	10.573
ct92-M305Pete-12	120362	24/12/2012	-2	78.91	10.557
ct92-M305Pete-12	120362	24/12/2012	-2	78.907	10.575
ct92-M305Pete-12	120362	24/12/2012	-2	78.905	10.547
ct92-M305Pete-12	120362	24/12/2012	-1	78.907	10.557
ct92-M305Pete-12	120362	24/12/2012	-2	78.906	10.548
ct92-M305Pete-12	120362	24/12/2012	-1	78.913	10.6
ct92-M305Pete-12	120362	24/12/2012	-2	78.913	10.599
ct92-M305Pete-12	120362	24/12/2012	-2	78.911	10.559
ct92-M305Pete-12	120362	24/12/2012	-2	78.924	10.584
ct92-M305Pete-12	120362	24/12/2012	-2	78.915	10.63
ct92-M305Pete-12	120362	24/12/2012	-2	78.916	10.609
ct92-M305Pete-12	120362	24/12/2012	-2	78.919	10.633
ct92-M305Pete-12	120362	24/12/2012	1	78.922	10.57
ct92-M305Pete-12	120362	24/12/2012	-2	78.918	10.589
ct92-M305Pete-12	120362	24/12/2012	1	78.912	10.56
ct92-M305Pete-12	120362	24/12/2012	-1	78.917	10.563
ct92-M305Pete-12	120362	24/12/2012	-2	78.911	10.564
ct92-M305Pete-12	120362	24/12/2012	-2	78.918	10.54
ct92-M305Pete-12	120362	24/12/2012	-1	78.912	10.555
ct92-M305Pete-12	120362	24/12/2012	-2	78.917	10.552
ct92-M305Pete-12	120362	24/12/2012	-1	78.922	10.611
ct92-M305Pete-12	120362	25/12/2012	-2	78.922	10.572
ct92-M305Pete-12	120362	25/12/2012	-2	78.922	10.58
ct92-M305Pete-12	120362	25/12/2012	-2	78.915	10.545
ct92-M305Pete-12	120362	25/12/2012	-2	78.902	10.521
ct92-M305Pete-12	120362	25/12/2012	-2	78.898	10.612
ct92-M305Pete-12	120362	25/12/2012	-2	78.888	10.542
ct92-M305Pete-12	120362	25/12/2012	-2	78.896	10.574
ct92-M305Pete-12	120362	25/12/2012	-2	78.895	10.638
ct92-M305Pete-12	120362	25/12/2012	-2	78.898	10.632
ct92-M305Pete-12	120362	25/12/2012	-2	78.913	10.602
ct92-M305Pete-12	120362	25/12/2012	-2	78.921	10.589
ct92-M305Pete-12	120362	25/12/2012	-2	78.911	10.542
ct92-M305Pete-12	120362	25/12/2012	-1	78.956	10.617
ct92-M305Pete-12	120362	25/12/2012	-2	78.948	10.634
ct92-M305Pete-12	120362	25/12/2012	-2	78.967	10.585
ct92-M305Pete-12	120362	25/12/2012	-2	78.959	10.527
ct92-M305Pete-12	120362	25/12/2012	-1	78.962	10.597
ct92-M305Pete-12	120362	25/12/2012	-2	78.947	10.648
ct92-M305Pete-12	120362	25/12/2012	-1	78.947	10.658
ct92-M305Pete-12	120362	25/12/2012	-2	78.942	10.66
ct92-M305Pete-12	120362	25/12/2012	-2	78.953	10.658
ct92-M305Pete-12	120362	25/12/2012	-1	78.962	10.573
ct92-M305Pete-12	120362	25/12/2012	-2	78.938	10.587
ct92-M305Pete-12	120362	25/12/2012	0	78.949	10.541
ct92-M305Pete-12	120362	25/12/2012	-1	78.943	10.536

ct92-M305Pete-12	120362	25/12/2012	0	78.932	10.536
ct92-M305Pete-12	120362	25/12/2012	-2	78.939	10.536
ct92-M305Pete-12	120362	25/12/2012	-2	78.941	10.535
ct92-M305Pete-12	120362	25/12/2012	-2	78.931	10.534
ct92-M305Pete-12	120362	25/12/2012	-1	78.934	10.452
ct92-M305Pete-12	120362	25/12/2012	-1	78.94	10.498
ct92-M305Pete-12	120362	25/12/2012	-1	78.933	10.482
ct92-M305Pete-12	120362	25/12/2012	-2	78.94	10.504
ct92-M305Pete-12	120362	25/12/2012	-1	78.941	10.501
ct92-M305Pete-12	120362	25/12/2012	-1	78.942	10.482
ct92-M305Pete-12	120362	25/12/2012	-2	78.934	10.505
ct92-M305Pete-12	120362	25/12/2012	1	78.943	10.516
ct92-M305Pete-12	120362	25/12/2012	1	78.946	10.532
ct92-M305Pete-12	120362	25/12/2012	-1	78.943	10.53
ct92-M305Pete-12	120362	25/12/2012	1	78.944	10.555
ct92-M305Pete-12	120362	25/12/2012	-2	78.946	10.508
ct92-M305Pete-12	120362	25/12/2012	-2	78.936	10.485
ct92-M305Pete-12	120362	25/12/2012	-2	78.926	10.482
ct92-M305Pete-12	120362	25/12/2012	-2	78.93	10.504
ct92-M305Pete-12	120362	25/12/2012	-2	78.928	10.509
ct92-M305Pete-12	120362	25/12/2012	-2	78.924	10.507
ct92-M305Pete-12	120362	25/12/2012	-2	78.925	10.515
ct92-M305Pete-12	120362	25/12/2012	-1	78.945	10.548
ct92-M305Pete-12	120362	25/12/2012	1	78.952	10.492
ct92-M305Pete-12	120362	25/12/2012	-1	78.945	10.495
ct92-M305Pete-12	120362	25/12/2012	-2	78.944	10.487
ct92-M305Pete-12	120362	25/12/2012	0	78.924	10.531
ct92-M305Pete-12	120362	25/12/2012	-2	78.915	10.501
ct92-M305Pete-12	120362	25/12/2012	-2	78.903	10.559
ct92-M305Pete-12	120362	25/12/2012	-1	78.896	10.602
ct92-M305Pete-12	120362	25/12/2012	-2	78.893	10.59
ct92-M305Pete-12	120362	25/12/2012	-1	78.92	10.589
ct92-M305Pete-12	120362	25/12/2012	-2	78.914	10.609
ct92-M305Pete-12	120362	25/12/2012	-2	78.915	10.616
ct92-M305Pete-12	120362	25/12/2012	-1	78.884	10.51
ct92-M305Pete-12	120362	25/12/2012	-2	78.907	10.761
ct92-M305Pete-12	120362	25/12/2012	-2	78.919	10.753
ct92-M305Pete-12	120362	25/12/2012	-1	78.923	10.746
ct92-M305Pete-12	120362	25/12/2012	-2	78.924	10.745
ct92-M305Pete-12	120362	25/12/2012	-1	78.932	10.8
ct92-M305Pete-12	120362	25/12/2012	-2	78.921	10.626
ct92-M305Pete-12	120362	25/12/2012	-2	78.916	10.627
ct92-M305Pete-12	120362	25/12/2012	-2	78.912	10.639
ct92-M305Pete-12	120362	26/12/2012	-2	78.911	10.647
ct92-M305Pete-12	120362	26/12/2012	-1	78.919	10.666
ct92-M305Pete-12	120362	26/12/2012	-2	78.909	10.634
ct92-M305Pete-12	120362	26/12/2012	-2	78.912	10.625
ct92-M305Pete-12	120362	26/12/2012	2	78.904	10.692
ct92-M305Pete-12	120362	26/12/2012	-2	78.903	10.68
ct92-M305Pete-12	120362	26/12/2012	-2	78.904	10.674
ct92-M305Pete-12	120362	26/12/2012	-2	78.926	10.678
ct92-M305Pete-12	120362	26/12/2012	-2	78.916	10.557
ct92-M305Pete-12	120362	26/12/2012	-2	78.914	10.589
ct92-M305Pete-12	120362	26/12/2012	-2	78.918	10.643
ct92-M305Pete-12	120362	26/12/2012	-2	78.918	10.605
ct92-M305Pete-12	120362	26/12/2012	-2	78.918	10.621
ct92-M305Pete-12	120362	26/12/2012	-2	78.917	10.587
ct92-M305Pete-12	120362	26/12/2012	-1	78.931	10.526
ct92-M305Pete-12	120362	26/12/2012	-2	78.93	10.515
ct92-M305Pete-12	120362	26/12/2012	-2	78.93	10.504
ct92-M305Pete-12	120362	26/12/2012	-2	78.93	10.515
ct92-M305Pete-12	120362	26/12/2012	-2	78.942	10.479
ct92-M305Pete-12	120362	26/12/2012	-2	78.931	10.52
ct92-M305Pete-12	120362	26/12/2012	-2	78.927	10.499
ct92-M305Pete-12	120362	26/12/2012	-2	78.943	10.461
ct92-M305Pete-12	120362	26/12/2012	-2	78.938	10.496
ct92-M305Pete-12	120362	26/12/2012	-2	78.939	10.492
ct92-M305Pete-12	120362	26/12/2012	-2	78.964	10.446

ct92-M305Pete-12	120362	26/12/2012	-2	78.972	10.556
ct92-M305Pete-12	120362	26/12/2012	-2	78.964	10.586
ct92-M305Pete-12	120362	26/12/2012	-2	78.964	10.608
ct92-M305Pete-12	120362	26/12/2012	-2	78.952	10.606
ct92-M305Pete-12	120362	26/12/2012	-2	78.95	10.603
ct92-M305Pete-12	120362	26/12/2012	-1	78.954	10.623
ct92-M305Pete-12	120362	26/12/2012	-2	78.951	10.634
ct92-M305Pete-12	120362	26/12/2012	-1	78.934	10.688
ct92-M305Pete-12	120362	26/12/2012	-2	78.941	10.667
ct92-M305Pete-12	120362	26/12/2012	-1	78.94	10.656
ct92-M305Pete-12	120362	26/12/2012	-2	78.943	10.634
ct92-M305Pete-12	120362	26/12/2012	-2	78.947	10.666
ct92-M305Pete-12	120362	26/12/2012	-1	78.944	10.613
ct92-M305Pete-12	120362	26/12/2012	-2	78.944	10.685
ct92-M305Pete-12	120362	26/12/2012	-2	78.938	10.624
ct92-M305Pete-12	120362	26/12/2012	-2	78.938	10.623
ct92-M305Pete-12	120362	26/12/2012	-2	78.936	10.649
ct92-M305Pete-12	120362	26/12/2012	-2	78.935	10.668
ct92-M305Pete-12	120362	26/12/2012	-1	78.926	10.626
ct92-M305Pete-12	120362	26/12/2012	-2	78.934	10.602
ct92-M305Pete-12	120362	26/12/2012	-2	78.936	10.603
ct92-M305Pete-12	120362	26/12/2012	-2	78.934	10.599
ct92-M305Pete-12	120362	26/12/2012	-2	78.913	10.681
ct92-M305Pete-12	120362	26/12/2012	-2	78.928	10.643
ct92-M305Pete-12	120362	26/12/2012	-2	78.917	10.567
ct92-M305Pete-12	120362	26/12/2012	-2	78.916	10.571
ct92-M305Pete-12	120362	26/12/2012	-2	78.915	10.554
ct92-M305Pete-12	120362	26/12/2012	-2	78.922	10.535
ct92-M305Pete-12	120362	26/12/2012	-2	78.913	10.567
ct92-M305Pete-12	120362	26/12/2012	-2	78.912	10.569
ct92-M305Pete-12	120362	26/12/2012	-2	78.907	10.567
ct92-M305Pete-12	120362	26/12/2012	-2	78.908	10.556
ct92-M305Pete-12	120362	26/12/2012	-2	78.91	10.533
ct92-M305Pete-12	120362	26/12/2012	-2	78.91	10.533
ct92-M305Pete-12	120362	26/12/2012	-2	78.934	10.489
ct92-M305Pete-12	120362	26/12/2012	-2	78.906	10.581
ct92-M305Pete-12	120362	26/12/2012	-2	78.925	10.62
ct92-M305Pete-12	120362	26/12/2012	-2	78.934	10.572
ct92-M305Pete-12	120362	26/12/2012	-2	78.928	10.623
ct92-M305Pete-12	120362	26/12/2012	-2	78.929	10.619
ct92-M305Pete-12	120362	26/12/2012	-2	78.931	10.629
ct92-M305Pete-12	120362	27/12/2012	-2	78.928	10.614
ct92-M305Pete-12	120362	27/12/2012	-2	78.955	10.47
ct92-M305Pete-12	120362	27/12/2012	-2	78.955	10.463
ct92-M305Pete-12	120362	27/12/2012	-2	78.948	10.502
ct92-M305Pete-12	120362	27/12/2012	-2	78.948	10.5
ct92-M305Pete-12	120362	27/12/2012	-2	78.946	10.492
ct92-M305Pete-12	120362	27/12/2012	-1	78.948	10.493
ct92-M305Pete-12	120362	27/12/2012	-2	78.937	10.461
ct92-M305Pete-12	120362	27/12/2012	-2	78.941	10.435
ct92-M305Pete-12	120362	27/12/2012	-2	78.939	10.428
ct92-M305Pete-12	120362	27/12/2012	-2	78.938	10.403
ct92-M305Pete-12	120362	27/12/2012	-1	78.927	10.468
ct92-M305Pete-12	120362	27/12/2012	1	78.927	10.455
ct92-M305Pete-12	120362	27/12/2012	-2	78.923	10.484
ct92-M305Pete-12	120362	27/12/2012	-2	78.916	10.407
ct92-M305Pete-12	120362	27/12/2012	-2	78.92	10.463
ct92-M305Pete-12	120362	27/12/2012	-2	78.924	10.462
ct92-M305Pete-12	120362	27/12/2012	-2	78.926	10.447
ct92-M305Pete-12	120362	27/12/2012	-2	78.903	10.477
ct92-M305Pete-12	120362	27/12/2012	-2	78.899	10.401
ct92-M305Pete-12	120362	27/12/2012	-2	78.899	10.42
ct92-M305Pete-12	120362	27/12/2012	-2	78.903	10.397
ct92-M305Pete-12	120362	27/12/2012	-2	78.894	10.412
ct92-M305Pete-12	120362	27/12/2012	-2	78.894	10.411
ct92-M305Pete-12	120362	27/12/2012	-2	78.894	10.41
ct92-M305Pete-12	120362	27/12/2012	-1	78.9	10.42
ct92-M305Pete-12	120362	27/12/2012	-2	78.908	10.39

ct92-M305Pete-12	120362	27/12/2012	-2	78.895	10.301
ct92-M305Pete-12	120362	27/12/2012	-1	78.918	10.417
ct92-M305Pete-12	120362	27/12/2012	-1	78.925	10.397
ct92-M305Pete-12	120362	27/12/2012	-1	78.897	10.3
ct92-M305Pete-12	120362	27/12/2012	-2	78.9	10.607
ct92-M305Pete-12	120362	27/12/2012	-1	78.927	10.528
ct92-M305Pete-12	120362	27/12/2012	-2	78.938	10.49
ct92-M305Pete-12	120362	27/12/2012	-2	78.926	10.541
ct92-M305Pete-12	120362	27/12/2012	-1	78.924	10.475
ct92-M305Pete-12	120362	27/12/2012	-1	78.926	10.529
ct92-M305Pete-12	120362	27/12/2012	-2	78.922	10.502
ct92-M305Pete-12	120362	27/12/2012	-1	78.868	10.584
ct92-M305Pete-12	120362	27/12/2012	-1	78.927	10.501
ct92-M305Pete-12	120362	27/12/2012	-2	78.921	10.517
ct92-M305Pete-12	120362	27/12/2012	-2	78.909	10.509
ct92-M305Pete-12	120362	27/12/2012	-2	78.891	10.479
ct92-M305Pete-12	120362	27/12/2012	-2	78.904	10.525
ct92-M305Pete-12	120362	27/12/2012	-2	78.901	10.549
ct92-M305Pete-12	120362	27/12/2012	-2	78.916	10.479
ct92-M305Pete-12	120362	27/12/2012	-1	78.904	10.541
ct92-M305Pete-12	120362	27/12/2012	-2	78.893	10.56
ct92-M305Pete-12	120362	27/12/2012	-2	78.898	10.547
ct92-M305Pete-12	120362	27/12/2012	-2	78.901	10.54
ct92-M305Pete-12	120362	27/12/2012	-2	78.924	10.498
ct92-M305Pete-12	120362	27/12/2012	-2	78.902	10.534
ct92-M305Pete-12	120362	27/12/2012	-2	78.91	10.506
ct92-M305Pete-12	120362	27/12/2012	-2	78.89	10.622
ct92-M305Pete-12	120362	27/12/2012	-2	78.911	10.562
ct92-M305Pete-12	120362	27/12/2012	-2	78.913	10.561
ct92-M305Pete-12	120362	27/12/2012	-2	78.91	10.46
ct92-M305Pete-12	120362	27/12/2012	-2	78.925	10.594
ct92-M305Pete-12	120362	27/12/2012	1	78.929	10.604
ct92-M305Pete-12	120362	27/12/2012	-2	78.93	10.594
ct92-M305Pete-12	120362	27/12/2012	-2	78.932	10.622
ct92-M305Pete-12	120362	27/12/2012	2	78.934	10.514
ct92-M305Pete-12	120362	27/12/2012	-2	78.934	10.538
ct92-M305Pete-12	120362	27/12/2012	-2	78.936	10.636
ct92-M305Pete-12	120362	27/12/2012	-2	78.936	10.648
ct92-M305Pete-12	120362	27/12/2012	-2	78.935	10.626
ct92-M305Pete-12	120362	27/12/2012	-2	78.939	10.659
ct92-M305Pete-12	120362	27/12/2012	-2	78.938	10.64
ct92-M305Pete-12	120362	27/12/2012	-1	78.942	10.584
ct92-M305Pete-12	120362	28/12/2012	-2	78.947	10.642
ct92-M305Pete-12	120362	28/12/2012	-1	78.941	10.636
ct92-M305Pete-12	120362	28/12/2012	-2	78.95	10.583
ct92-M305Pete-12	120362	28/12/2012	-2	78.941	10.636
ct92-M305Pete-12	120362	28/12/2012	-2	78.944	10.659
ct92-M305Pete-12	120362	28/12/2012	-2	78.933	10.614
ct92-M305Pete-12	120362	28/12/2012	-2	78.934	10.632
ct92-M305Pete-12	120362	28/12/2012	0	78.956	10.661
ct92-M305Pete-12	120362	28/12/2012	-2	78.962	10.628
ct92-M305Pete-12	120362	28/12/2012	-2	78.962	10.636
ct92-M305Pete-12	120362	28/12/2012	-2	78.962	10.668
ct92-M305Pete-12	120362	28/12/2012	-1	78.954	10.526
ct92-M305Pete-12	120362	28/12/2012	0	78.956	10.588
ct92-M305Pete-12	120362	28/12/2012	-2	78.959	10.62
ct92-M305Pete-12	120362	28/12/2012	-1	78.939	10.569
ct92-M305Pete-12	120362	28/12/2012	-1	78.938	10.568
ct92-M305Pete-12	120362	28/12/2012	-2	78.941	10.569
ct92-M305Pete-12	120362	28/12/2012	-2	78.941	10.583
ct92-M305Pete-12	120362	28/12/2012	-1	78.925	10.477
ct92-M305Pete-12	120362	28/12/2012	-2	78.923	10.462
ct92-M305Pete-12	120362	28/12/2012	-2	78.924	10.459
ct92-M305Pete-12	120362	28/12/2012	-2	78.925	10.508
ct92-M305Pete-12	120362	28/12/2012	-2	78.924	10.533
ct92-M305Pete-12	120362	28/12/2012	-2	78.9	10.418
ct92-M305Pete-12	120362	28/12/2012	-2	78.899	10.425
ct92-M305Pete-12	120362	28/12/2012	-2	78.925	10.477

ct92-M305Pete-12	120362	28/12/2012	-2	78.925	10.485
ct92-M305Pete-12	120362	28/12/2012	-1	78.871	10.428
ct92-M305Pete-12	120362	28/12/2012	-2	78.86	10.428
ct92-M305Pete-12	120362	28/12/2012	1	78.855	10.422
ct92-M305Pete-12	120362	28/12/2012	0	78.823	10.324
ct92-M305Pete-12	120362	28/12/2012	-2	78.828	10.337
ct92-M305Pete-12	120362	28/12/2012	-1	78.833	10.521
ct92-M305Pete-12	120362	28/12/2012	-1	78.828	10.386
ct92-M305Pete-12	120362	28/12/2012	0	78.813	10.396
ct92-M305Pete-12	120362	28/12/2012	-1	78.789	10.492
ct92-M305Pete-12	120362	28/12/2012	-1	78.797	10.482
ct92-M305Pete-12	120362	28/12/2012	-2	78.798	10.483
ct92-M305Pete-12	120362	28/12/2012	-2	78.782	10.442
ct92-M305Pete-12	120362	28/12/2012	-2	78.778	10.456
ct92-M305Pete-12	120362	28/12/2012	-2	78.766	10.469
ct92-M305Pete-12	120362	28/12/2012	-2	78.761	10.519
ct92-M305Pete-12	120362	28/12/2012	-2	78.741	10.467
ct92-M305Pete-12	120362	28/12/2012	-2	78.738	10.468
ct92-M305Pete-12	120362	28/12/2012	-2	78.735	10.469
ct92-M305Pete-12	120362	28/12/2012	-2	78.73	10.474
ct92-M305Pete-12	120362	28/12/2012	-2	78.72	10.489
ct92-M305Pete-12	120362	28/12/2012	-1	78.706	10.634
ct92-M305Pete-12	120362	28/12/2012	-2	78.73	10.527
ct92-M305Pete-12	120362	28/12/2012	-2	78.724	10.556
ct92-M305Pete-12	120362	28/12/2012	-2	78.701	10.581
ct92-M305Pete-12	120362	28/12/2012	-2	78.718	10.554
ct92-M305Pete-12	120362	28/12/2012	-2	78.695	10.671
ct92-M305Pete-12	120362	28/12/2012	-2	78.7	10.682
ct92-M305Pete-12	120362	28/12/2012	-2	78.697	10.702
ct92-M305Pete-12	120362	28/12/2012	-2	78.652	10.751
ct92-M305Pete-12	120362	28/12/2012	-2	78.669	10.785
ct92-M305Pete-12	120362	28/12/2012	-2	78.645	10.626
ct92-M305Pete-12	120362	28/12/2012	-2	78.604	10.751
ct92-M305Pete-12	120362	28/12/2012	-2	78.595	10.777
ct92-M305Pete-12	120362	28/12/2012	-2	78.607	10.764
ct92-M305Pete-12	120362	28/12/2012	-2	78.591	10.794
ct92-M305Pete-12	120362	28/12/2012	-2	78.592	10.801
ct92-M305Pete-12	120362	28/12/2012	-2	78.569	11.009
ct92-M305Pete-12	120362	28/12/2012	2	78.555	10.947
ct92-M305Pete-12	120362	28/12/2012	2	78.556	10.947
ct92-M305Pete-12	120362	28/12/2012	-2	78.556	10.95
ct92-M305Pete-12	120362	28/12/2012	-2	78.558	10.939
ct92-M305Pete-12	120362	28/12/2012	-2	78.555	10.938
ct92-M305Pete-12	120362	28/12/2012	-2	78.538	10.958
ct92-M305Pete-12	120362	28/12/2012	-2	78.495	10.98
ct92-M305Pete-12	120362	28/12/2012	-2	78.507	10.979
ct92-M305Pete-12	120362	28/12/2012	-2	78.492	10.984
ct92-M305Pete-12	120362	28/12/2012	-2	78.441	11.043
ct92-M305Pete-12	120362	28/12/2012	-2	78.432	11.046
ct92-M305Pete-12	120362	28/12/2012	-2	78.436	11.08
ct92-M305Pete-12	120362	28/12/2012	-1	78.424	11.318
ct92-M305Pete-12	120362	29/12/2012	-2	78.431	11.295
ct92-M305Pete-12	120362	29/12/2012	-2	78.431	11.296
ct92-M305Pete-12	120362	29/12/2012	-2	78.42	11.302
ct92-M305Pete-12	120362	29/12/2012	-2	78.421	11.308
ct92-M305Pete-12	120362	29/12/2012	-2	78.422	11.33
ct92-M305Pete-12	120362	29/12/2012	-2	78.381	11.32
ct92-M305Pete-12	120362	29/12/2012	-2	78.346	11.438
ct92-M305Pete-12	120362	29/12/2012	-2	78.37	11.474
ct92-M305Pete-12	120362	29/12/2012	-2	78.35	11.474
ct92-M305Pete-12	120362	29/12/2012	-2	78.352	11.498
ct92-M305Pete-12	120362	29/12/2012	-2	78.342	11.507
ct92-M305Pete-12	120362	29/12/2012	-2	78.356	11.499
ct92-M305Pete-12	120362	29/12/2012	-2	78.303	11.594
ct92-M305Pete-12	120362	29/12/2012	-2	78.324	11.559
ct92-M305Pete-12	120362	29/12/2012	-2	78.33	11.556
ct92-M305Pete-12	120362	29/12/2012	-2	78.336	11.531
ct92-M305Pete-12	120362	29/12/2012	-1	78.323	11.621

ct92-M305Pete-12	120362	29/12/2012	-2	78.326	11.602
ct92-M305Pete-12	120362	29/12/2012	-1	78.344	11.536
ct92-M305Pete-12	120362	29/12/2012	-1	78.323	11.702
ct92-M305Pete-12	120362	29/12/2012	-1	78.328	11.674
ct92-M305Pete-12	120362	29/12/2012	-2	78.333	11.585
ct92-M305Pete-12	120362	29/12/2012	-2	78.286	11.567
ct92-M305Pete-12	120362	29/12/2012	-2	78.313	11.565
ct92-M305Pete-12	120362	29/12/2012	-2	78.367	11.887
ct92-M305Pete-12	120362	29/12/2012	-2	78.274	11.637
ct92-M305Pete-12	120362	29/12/2012	-1	78.35	11.526
ct92-M305Pete-12	120362	29/12/2012	-2	78.347	11.544
ct92-M305Pete-12	120362	29/12/2012	-2	78.345	11.545
ct92-M305Pete-12	120362	29/12/2012	-1	78.335	11.529
ct92-M305Pete-12	120362	29/12/2012	-2	78.371	11.985
ct92-M305Pete-12	120362	29/12/2012	-2	78.371	12.007
ct92-M305Pete-12	120362	29/12/2012	-1	78.344	11.533
ct92-M305Pete-12	120362	29/12/2012	-1	78.347	11.563
ct92-M305Pete-12	120362	29/12/2012	-2	78.336	11.496
ct92-M305Pete-12	120362	29/12/2012	-2	78.352	11.518
ct92-M305Pete-12	120362	29/12/2012	-2	78.35	11.392
ct92-M305Pete-12	120362	29/12/2012	-2	78.327	11.562
ct92-M305Pete-12	120362	29/12/2012	-2	78.346	11.386
ct92-M305Pete-12	120362	29/12/2012	-2	78.339	11.607
ct92-M305Pete-12	120362	29/12/2012	-2	78.343	11.64
ct92-M305Pete-12	120362	29/12/2012	-2	78.337	11.655
ct92-M305Pete-12	120362	29/12/2012	-2	78.334	11.643
ct92-M305Pete-12	120362	29/12/2012	-2	78.337	11.594
ct92-M305Pete-12	120362	29/12/2012	-2	78.334	11.594
ct92-M305Pete-12	120362	29/12/2012	-2	78.332	11.595
ct92-M305Pete-12	120362	29/12/2012	-2	78.326	11.668
ct92-M305Pete-12	120362	29/12/2012	-2	78.356	11.598
ct92-M305Pete-12	120362	29/12/2012	-1	78.341	11.509
ct92-M305Pete-12	120362	29/12/2012	-2	78.341	11.466
ct92-M305Pete-12	120362	29/12/2012	-2	78.341	11.45
ct92-M305Pete-12	120362	29/12/2012	-2	78.344	11.45
ct92-M305Pete-12	120362	29/12/2012	-2	78.335	11.467
ct92-M305Pete-12	120362	29/12/2012	-2	78.338	11.459
ct92-M305Pete-12	120362	29/12/2012	-2	78.321	11.407
ct92-M305Pete-12	120362	29/12/2012	-2	78.346	11.446
ct92-M305Pete-12	120362	29/12/2012	-2	78.344	11.442
ct92-M305Pete-12	120362	29/12/2012	-2	78.355	11.381
ct92-M305Pete-12	120362	29/12/2012	-2	78.356	11.378
ct92-M305Pete-12	120362	29/12/2012	-1	78.369	11.419
ct92-M305Pete-12	120362	29/12/2012	-2	78.356	11.38
ct92-M305Pete-12	120362	29/12/2012	-2	78.359	11.374
ct92-M305Pete-12	120362	29/12/2012	-2	78.36	11.368
ct92-M305Pete-12	120362	30/12/2012	-1	78.373	11.335
ct92-M305Pete-12	120362	30/12/2012	-2	78.393	11.342
ct92-M305Pete-12	120362	30/12/2012	-2	78.403	11.316
ct92-M305Pete-12	120362	30/12/2012	-1	78.38	11.428
ct92-M305Pete-12	120362	30/12/2012	-2	78.363	11.359
ct92-M305Pete-12	120362	30/12/2012	-2	78.367	11.285
ct92-M305Pete-12	120362	30/12/2012	-1	78.389	11.131
ct92-M305Pete-12	120362	30/12/2012	-2	78.384	11.205
ct92-M305Pete-12	120362	30/12/2012	-2	78.389	11.206
ct92-M305Pete-12	120362	30/12/2012	-1	78.389	11.203
ct92-M305Pete-12	120362	30/12/2012	-2	78.378	11.268
ct92-M305Pete-12	120362	30/12/2012	-2	78.386	11.298
ct92-M305Pete-12	120362	30/12/2012	-2	78.382	11.291
ct92-M305Pete-12	120362	30/12/2012	-2	78.381	11.294
ct92-M305Pete-12	120362	30/12/2012	-1	78.38	11.285
ct92-M305Pete-12	120362	30/12/2012	-2	78.381	11.284
ct92-M305Pete-12	120362	30/12/2012	-2	78.38	11.276
ct92-M305Pete-12	120362	30/12/2012	-2	78.394	11.254
ct92-M305Pete-12	120362	30/12/2012	-2	78.395	11.267
ct92-M305Pete-12	120362	30/12/2012	-1	78.395	11.249
ct92-M305Pete-12	120362	30/12/2012	-2	78.397	11.245
ct92-M305Pete-12	120362	30/12/2012	-2	78.399	11.253

ct92-M305Pete-12	120362	30/12/2012	-2	78.404	11.251
ct92-M305Pete-12	120362	30/12/2012	-2	78.407	11.252
ct92-M305Pete-12	120362	30/12/2012	-2	78.408	11.242
ct92-M305Pete-12	120362	30/12/2012	-2	78.409	11.249
ct92-M305Pete-12	120362	30/12/2012	-2	78.407	11.245
ct92-M305Pete-12	120362	30/12/2012	-2	78.415	11.189
ct92-M305Pete-12	120362	30/12/2012	-2	78.414	11.158
ct92-M305Pete-12	120362	30/12/2012	0	78.418	11.212
ct92-M305Pete-12	120362	30/12/2012	-1	78.416	11.166
ct92-M305Pete-12	120362	30/12/2012	-2	78.412	11.175
ct92-M305Pete-12	120362	30/12/2012	-2	78.421	11.166
ct92-M305Pete-12	120362	30/12/2012	-2	78.417	11.174
ct92-M305Pete-12	120362	30/12/2012	-2	78.428	11.096
ct92-M305Pete-12	120362	30/12/2012	1	78.436	11.058
ct92-M305Pete-12	120362	30/12/2012	2	78.426	11.067
ct92-M305Pete-12	120362	30/12/2012	2	78.426	11.065
ct92-M305Pete-12	120362	30/12/2012	2	78.435	11.036
ct92-M305Pete-12	120362	30/12/2012	-2	78.455	10.878
ct92-M305Pete-12	120362	30/12/2012	-2	78.472	10.895
ct92-M305Pete-12	120362	30/12/2012	-2	78.475	10.88
ct92-M305Pete-12	120362	30/12/2012	-2	78.486	10.848
ct92-M305Pete-12	120362	30/12/2012	-2	78.494	10.808
ct92-M305Pete-12	120362	30/12/2012	-2	78.495	10.835
ct92-M305Pete-12	120362	30/12/2012	-2	78.51	10.771
ct92-M305Pete-12	120362	30/12/2012	-2	78.504	10.822
ct92-M305Pete-12	120362	30/12/2012	-2	78.54	10.793
ct92-M305Pete-12	120362	30/12/2012	-2	78.546	10.866
ct92-M305Pete-12	120362	30/12/2012	-2	78.546	10.879
ct92-M305Pete-12	120362	30/12/2012	-2	78.571	10.852
ct92-M305Pete-12	120362	30/12/2012	-2	78.578	10.831
ct92-M305Pete-12	120362	30/12/2012	-2	78.579	10.838
ct92-M305Pete-12	120362	30/12/2012	-2	78.579	10.838
ct92-M305Pete-12	120362	30/12/2012	-2	78.558	10.835
ct92-M305Pete-12	120362	30/12/2012	-2	78.563	10.809
ct92-M305Pete-12	120362	30/12/2012	-2	78.561	10.814
ct92-M305Pete-12	120362	30/12/2012	-2	78.574	10.78
ct92-M305Pete-12	120362	30/12/2012	-2	78.585	10.76
ct92-M305Pete-12	120362	30/12/2012	-2	78.585	10.76
ct92-M305Pete-12	120362	30/12/2012	-1	78.601	10.787
ct92-M305Pete-12	120362	30/12/2012	-2	78.607	10.778
ct92-M305Pete-12	120362	30/12/2012	-2	78.618	10.776
ct92-M305Pete-12	120362	31/12/2012	-2	78.624	10.775
ct92-M305Pete-12	120362	31/12/2012	-2	78.622	10.758
ct92-M305Pete-12	120362	31/12/2012	-2	78.634	10.723
ct92-M305Pete-12	120362	31/12/2012	-1	78.623	10.709
ct92-M305Pete-12	120362	31/12/2012	-2	78.647	10.666
ct92-M305Pete-12	120362	31/12/2012	-2	78.655	10.681
ct92-M305Pete-12	120362	31/12/2012	-2	78.659	10.655
ct92-M305Pete-12	120362	31/12/2012	-2	78.618	10.546
ct92-M305Pete-12	120362	31/12/2012	-2	78.63	10.524
ct92-M305Pete-12	120362	31/12/2012	-2	78.663	10.516
ct92-M305Pete-12	120362	31/12/2012	-1	78.665	10.67
ct92-M305Pete-12	120362	31/12/2012	-2	78.665	10.673
ct92-M305Pete-12	120362	31/12/2012	-2	78.673	10.663
ct92-M305Pete-12	120362	31/12/2012	-2	78.714	10.536
ct92-M305Pete-12	120362	31/12/2012	-2	78.692	10.529
ct92-M305Pete-12	120362	31/12/2012	-1	78.722	10.531
ct92-M305Pete-12	120362	31/12/2012	-2	78.739	10.553
ct92-M305Pete-12	120362	31/12/2012	-1	78.761	10.24
ct92-M305Pete-12	120362	31/12/2012	-2	78.741	10.382
ct92-M305Pete-12	120362	31/12/2012	-2	78.754	10.441
ct92-M305Pete-12	120362	31/12/2012	-2	78.752	10.481
ct92-M305Pete-12	120362	31/12/2012	-2	78.762	10.435
ct92-M305Pete-12	120362	31/12/2012	-2	78.771	10.401
ct92-M305Pete-12	120362	31/12/2012	-2	78.78	10.342
ct92-M305Pete-12	120362	31/12/2012	-1	78.786	10.343
ct92-M305Pete-12	120362	31/12/2012	-2	78.791	10.32
ct92-M305Pete-12	120362	31/12/2012	-2	78.8	10.28

ct92-M305Pete-12	120362	31/12/2012	-2	78.798	10.298
ct92-M305Pete-12	120362	31/12/2012	-2	78.803	10.286
ct92-M305Pete-12	120362	31/12/2012	-2	78.831	10.404
ct92-M305Pete-12	120362	31/12/2012	-2	78.838	10.42
ct92-M305Pete-12	120362	31/12/2012	-2	78.843	10.401
ct92-M305Pete-12	120362	31/12/2012	-2	78.866	10.344
ct92-M305Pete-12	120362	31/12/2012	-1	78.856	10.351
ct92-M305Pete-12	120362	31/12/2012	-2	78.865	10.331
ct92-M305Pete-12	120362	31/12/2012	-2	78.882	10.369
ct92-M305Pete-12	120362	31/12/2012	-2	78.885	10.398
ct92-M305Pete-12	120362	31/12/2012	-2	78.897	10.391
ct92-M305Pete-12	120362	31/12/2012	-2	78.926	10.247
ct92-M305Pete-12	120362	31/12/2012	-2	78.891	10.335
ct92-M305Pete-12	120362	31/12/2012	-2	78.945	10.598
ct92-M305Pete-12	120362	31/12/2012	-2	78.961	10.644
ct92-M305Pete-12	120362	31/12/2012	-2	78.912	10.504
ct92-M305Pete-12	120362	31/12/2012	-2	78.93	10.526
ct92-M305Pete-12	120362	31/12/2012	-2	78.907	10.519
ct92-M305Pete-12	120362	31/12/2012	-2	78.906	10.52
ct92-M305Pete-12	120362	31/12/2012	-2	78.913	10.533
ct92-M305Pete-12	120362	31/12/2012	-2	78.921	10.561
ct92-M305Pete-12	120362	31/12/2012	-2	78.934	10.592
ct92-M305Pete-12	120362	31/12/2012	-2	78.928	10.614
ct92-M305Pete-12	120362	31/12/2012	-2	78.935	10.65
ct92-M305Pete-12	120362	31/12/2012	-2	78.92	10.564
ct92-M305Pete-12	120362	31/12/2012	-2	78.9	10.579
ct92-M305Pete-12	120362	31/12/2012	-2	78.902	10.587
ct92-M305Pete-12	120362	31/12/2012	-2	78.915	10.604
ct92-M305Pete-12	120362	31/12/2012	-2	78.911	10.601
ct92-M305Pete-12	120362	31/12/2012	-2	78.919	10.619
ct92-M305Pete-12	120362	31/12/2012	-2	78.933	10.646
ct92-M305Pete-12	120362	31/12/2012	-2	78.929	10.618
ct92-M305Pete-12	120362	31/12/2012	-2	78.931	10.618
ct92-M305Pete-12	120362	31/12/2012	-2	78.945	10.653
ct92-M305Pete-12	120362	31/12/2012	-2	78.968	10.668
ct92-M305Pete-12	120362	31/12/2012	-2	78.927	10.58
ct92-M305Pete-12	120362	31/12/2012	-1	78.934	10.603
ct92-M305Pete-12	120362	31/12/2012	-2	78.942	10.63
ct92-M305Pete-12	120362	31/12/2012	-2	78.928	10.59
ct92-M305Pete-12	120362	31/12/2012	-2	78.935	10.616
ct92-M305Pete-12	120362	01/01/2013	-2	78.962	10.634
ct92-M305Pete-12	120362	01/01/2013	-2	78.953	10.609
ct92-M305Pete-12	120362	01/01/2013	-1	78.953	10.618
ct92-M305Pete-12	120362	01/01/2013	-2	78.954	10.624
ct92-M305Pete-12	120362	01/01/2013	-1	78.951	10.623
ct92-M305Pete-12	120362	01/01/2013	-2	78.955	10.589
ct92-M305Pete-12	120362	01/01/2013	-2	78.958	10.591
ct92-M305Pete-12	120362	01/01/2013	-2	78.958	10.592
ct92-M305Pete-12	120362	01/01/2013	-2	78.958	10.595
ct92-M305Pete-12	120362	01/01/2013	-2	78.934	10.618
ct92-M305Pete-12	120362	01/01/2013	-1	78.941	10.604
ct92-M305Pete-12	120362	01/01/2013	-2	78.956	10.581
ct92-M305Pete-12	120362	01/01/2013	-2	78.961	10.606
ct92-M305Pete-12	120362	01/01/2013	-1	78.942	10.537
ct92-M305Pete-12	120362	01/01/2013	-2	78.958	10.554
ct92-M305Pete-12	120362	01/01/2013	-2	78.946	10.408
ct92-M305Pete-12	120362	01/01/2013	-2	78.968	10.528
ct92-M305Pete-12	120362	01/01/2013	-2	78.964	10.574
ct92-M305Pete-12	120362	01/01/2013	-2	78.963	10.583
ct92-M305Pete-12	120362	01/01/2013	-9	79.042	10.113
ct92-M305Pete-12	120362	01/01/2013	0	78.94	10.471
ct92-M305Pete-12	120362	01/01/2013	-1	78.882	10.846
ct92-M305Pete-12	120362	01/01/2013	-2	78.955	10.517
ct92-M305Pete-12	120362	01/01/2013	-2	78.955	10.577
ct92-M305Pete-12	120362	01/01/2013	-2	78.956	10.552
ct92-M305Pete-12	120362	01/01/2013	-1	78.927	10.556
ct92-M305Pete-12	120362	01/01/2013	0	78.909	10.496
ct92-M305Pete-12	120362	01/01/2013	0	78.909	10.496

ct92-M305Pete-12	120362	01/01/2013	-2	78.931	10.573
ct92-M305Pete-12	120362	01/01/2013	-2	78.95	10.515
ct92-M305Pete-12	120362	01/01/2013	-2	78.926	10.551
ct92-M305Pete-12	120362	01/01/2013	-2	78.924	10.559
ct92-M305Pete-12	120362	01/01/2013	-1	78.928	10.614
ct92-M305Pete-12	120362	01/01/2013	-1	-4.546	95.104
ct92-M305Pete-12	120362	01/01/2013	-9	-4.523	95.128
ct92-M305Pete-12	120362	01/01/2013	-2	78.93	10.615
ct92-M305Pete-12	120362	01/01/2013	-2	78.912	10.587
ct92-M305Pete-12	120362	01/01/2013	-2	78.911	10.595
ct92-M305Pete-12	120362	01/01/2013	-1	78.902	10.635
ct92-M305Pete-12	120362	01/01/2013	-1	78.902	10.634
ct92-M305Pete-12	120362	01/01/2013	-9	-4.205	95.176
ct92-M305Pete-12	120362	01/01/2013	-2	78.903	10.624
ct92-M305Pete-12	120362	01/01/2013	-2	78.905	10.637
ct92-M305Pete-12	120362	01/01/2013	-2	78.905	10.638
ct92-M305Pete-12	120362	01/01/2013	-9	79.578	8.209
ct92-M305Pete-12	120362	01/01/2013	-2	78.909	10.622
ct92-M305Pete-12	120362	01/01/2013	-2	78.91	10.646
ct92-M305Pete-12	120362	01/01/2013	-2	78.914	10.654
ct92-M305Pete-12	120362	01/01/2013	-9	79.052	12.381
ct92-M305Pete-12	120362	01/01/2013	-2	78.91	10.649
ct92-M305Pete-12	120362	01/01/2013	-2	78.915	10.658
ct92-M305Pete-12	120362	01/01/2013	-2	78.915	10.662
ct92-M305Pete-12	120362	01/01/2013	-9	80.528	-10.568
ct92-M305Pete-12	120362	01/01/2013	-2	78.909	10.615
ct92-M305Pete-12	120362	01/01/2013	-2	78.913	10.564
ct92-M305Pete-12	120362	01/01/2013	-2	78.908	10.565
ct92-M305Pete-12	120362	02/01/2013	-2	78.924	10.712
ct92-M305Pete-12	120362	02/01/2013	-2	78.93	10.709
ct92-M305Pete-12	120362	02/01/2013	-2	78.958	10.608
ct92-M305Pete-12	120362	02/01/2013	-2	78.947	10.555
ct92-M305Pete-12	120362	02/01/2013	-2	78.946	10.459
ct92-M305Pete-12	120362	02/01/2013	-2	78.951	10.472
ct92-M305Pete-12	120362	02/01/2013	-2	78.951	10.472
ct92-M305Pete-12	120362	02/01/2013	-2	78.952	10.472
ct92-M305Pete-12	120362	02/01/2013	-2	78.952	10.481
ct92-M305Pete-12	120362	02/01/2013	-2	78.951	10.453
ct92-M305Pete-12	120362	02/01/2013	-1	0.57	116.226
ct92-M305Pete-12	120362	02/01/2013	-2	78.948	10.437
ct92-M305Pete-12	120362	02/01/2013	-9	-0.019	115.957
ct92-M305Pete-12	120362	02/01/2013	-2	79.103	12.253
ct92-M305Pete-12	120362	02/01/2013	-2	78.898	11.454
ct92-M305Pete-12	120362	02/01/2013	-1	78.927	10.366
ct92-M305Pete-12	120362	02/01/2013	-1	78.901	10.516
ct92-M305Pete-12	120362	02/01/2013	-1	79.969	10.503
ct92-M305Pete-12	120362	02/01/2013	-1	79.117	10.761
ct92-M305Pete-12	120362	02/01/2013	-2	78.962	10.32
ct92-M305Pete-12	120362	02/01/2013	-2	78.962	10.32
ct92-M305Pete-12	120362	02/01/2013	-2	78.917	10.268
ct92-M305Pete-12	120362	02/01/2013	-2	78.961	10.321
ct92-M305Pete-12	120362	02/01/2013	-2	78.961	10.313
ct92-M305Pete-12	120362	02/01/2013	-2	78.947	10.305
ct92-M305Pete-12	120362	02/01/2013	-2	78.947	10.305
ct92-M305Pete-12	120362	02/01/2013	-2	78.94	10.373
ct92-M305Pete-12	120362	02/01/2013	-2	78.937	10.369
ct92-M305Pete-12	120362	02/01/2013	-2	78.938	10.377
ct92-M305Pete-12	120362	02/01/2013	-2	78.926	10.395
ct92-M305Pete-12	120362	02/01/2013	-2	78.925	10.399
ct92-M305Pete-12	120362	02/01/2013	-2	78.926	10.432
ct92-M305Pete-12	120362	02/01/2013	-1	78.914	10.552
ct92-M305Pete-12	120362	02/01/2013	-2	78.917	10.492
ct92-M305Pete-12	120362	02/01/2013	-2	78.917	10.479
ct92-M305Pete-12	120362	02/01/2013	-2	78.923	10.518
ct92-M305Pete-12	120362	02/01/2013	-2	78.924	10.514
ct92-M305Pete-12	120362	02/01/2013	-2	78.924	10.494
ct92-M305Pete-12	120362	02/01/2013	-2	78.924	10.487
ct92-M305Pete-12	120362	02/01/2013	-2	78.924	10.548

ct92-M305Pete-12	120362	02/01/2013	-2	78.911	10.474
ct92-M305Pete-12	120362	02/01/2013	-2	78.924	10.547
ct92-M305Pete-12	120362	02/01/2013	-2	78.929	10.558
ct92-M305Pete-12	120362	02/01/2013	-2	78.916	10.625
ct92-M305Pete-12	120362	02/01/2013	-1	78.915	10.627
ct92-M305Pete-12	120362	02/01/2013	-2	78.918	10.605
ct92-M305Pete-12	120362	02/01/2013	-1	78.912	10.671
ct92-M305Pete-12	120362	02/01/2013	-2	78.92	10.598
ct92-M305Pete-12	120362	02/01/2013	-2	78.912	10.591
ct92-M305Pete-12	120362	02/01/2013	-2	78.917	10.652
ct92-M305Pete-12	120362	02/01/2013	-1	78.902	10.656
ct92-M305Pete-12	120362	02/01/2013	-2	78.899	10.638
ct92-M305Pete-12	120362	02/01/2013	-2	78.909	10.547
ct92-M305Pete-12	120362	02/01/2013	-2	78.938	10.629
ct92-M305Pete-12	120362	02/01/2013	-2	78.936	10.619
ct92-M305Pete-12	120362	02/01/2013	-2	78.929	10.584
ct92-M305Pete-12	120362	02/01/2013	-2	78.907	10.462
ct92-M305Pete-12	120362	02/01/2013	-2	78.892	10.361
ct92-M305Pete-12	120362	03/01/2013	-2	78.874	10.238
ct92-M305Pete-12	120362	03/01/2013	-2	78.879	10.272
ct92-M305Pete-12	120362	03/01/2013	-2	78.885	10.259
ct92-M305Pete-12	120362	03/01/2013	-2	78.916	10.484
ct92-M305Pete-12	120362	03/01/2013	-2	78.888	10.257
ct92-M305Pete-12	120362	03/01/2013	-2	78.921	10.255
ct92-M305Pete-12	120362	03/01/2013	-2	78.92	10.23
ct92-M305Pete-12	120362	03/01/2013	-2	78.926	10.191
ct92-M305Pete-12	120362	03/01/2013	-2	78.922	10.235
ct92-M305Pete-12	120362	03/01/2013	2	78.917	10.327
ct92-M305Pete-12	120362	03/01/2013	-2	78.914	10.339
ct92-M305Pete-12	120362	03/01/2013	-2	78.917	10.369
ct92-M305Pete-12	120362	03/01/2013	-2	78.927	10.486
ct92-M305Pete-12	120362	03/01/2013	-2	78.917	10.365
ct92-M305Pete-12	120362	03/01/2013	-2	78.917	10.38
ct92-M305Pete-12	120362	03/01/2013	-2	78.917	10.384
ct92-M305Pete-12	120362	03/01/2013	-1	78.926	10.438
ct92-M305Pete-12	120362	03/01/2013	-2	78.929	10.434
ct92-M305Pete-12	120362	03/01/2013	-2	78.919	10.342
ct92-M305Pete-12	120362	03/01/2013	-2	78.924	10.354
ct92-M305Pete-12	120362	03/01/2013	1	78.927	10.361
ct92-M305Pete-12	120362	03/01/2013	-2	78.927	10.354
ct92-M305Pete-12	120362	03/01/2013	-2	78.939	10.412
ct92-M305Pete-12	120362	03/01/2013	-2	78.921	10.287
ct92-M305Pete-12	120362	03/01/2013	-1	78.949	10.443
ct92-M305Pete-12	120362	03/01/2013	-2	78.948	10.442
ct92-M305Pete-12	120362	03/01/2013	-2	78.921	10.355
ct92-M305Pete-12	120362	03/01/2013	-2	78.931	10.419
ct92-M305Pete-12	120362	03/01/2013	0	78.918	10.257
ct92-M305Pete-12	120362	03/01/2013	-1	78.945	10.413
ct92-M305Pete-12	120362	03/01/2013	-2	78.94	10.38
ct92-M305Pete-12	120362	03/01/2013	-1	78.942	10.409
ct92-M305Pete-12	120362	03/01/2013	-2	78.916	10.399
ct92-M305Pete-12	120362	03/01/2013	-2	78.915	10.485
ct92-M305Pete-12	120362	03/01/2013	-2	78.898	10.379
ct92-M305Pete-12	120362	03/01/2013	-1	78.897	10.381
ct92-M305Pete-12	120362	03/01/2013	-1	78.875	10.386
ct92-M305Pete-12	120362	03/01/2013	-2	78.872	10.394
ct92-M305Pete-12	120362	03/01/2013	-2	78.861	10.422
ct92-M305Pete-12	120362	03/01/2013	-1	78.856	10.408
ct92-M305Pete-12	120362	03/01/2013	-2	78.855	10.397
ct92-M305Pete-12	120362	03/01/2013	-2	78.85	10.4
ct92-M305Pete-12	120362	03/01/2013	-2	78.848	10.396
ct92-M305Pete-12	120362	03/01/2013	-2	78.82	10.382
ct92-M305Pete-12	120362	03/01/2013	-2	78.817	10.381
ct92-M305Pete-12	120362	03/01/2013	-2	78.808	10.379
ct92-M305Pete-12	120362	03/01/2013	-2	78.812	10.377
ct92-M305Pete-12	120362	03/01/2013	-2	78.802	10.372
ct92-M305Pete-12	120362	03/01/2013	-2	78.803	10.376
ct92-M305Pete-12	120362	03/01/2013	-2	78.803	10.376

ct92-M305Pete-12	120362	03/01/2013	-1	78.794	10.281
ct92-M305Pete-12	120362	03/01/2013	-2	78.75	10.374
ct92-M305Pete-12	120362	03/01/2013	-1	78.754	10.216
ct92-M305Pete-12	120362	03/01/2013	-2	78.756	10.232
ct92-M305Pete-12	120362	03/01/2013	-2	78.727	10.284
ct92-M305Pete-12	120362	03/01/2013	-2	78.764	10.29
ct92-M305Pete-12	120362	03/01/2013	-1	78.772	10.382
ct92-M305Pete-12	120362	03/01/2013	-1	78.745	10.332
ct92-M305Pete-12	120362	03/01/2013	-2	78.767	10.411
ct92-M305Pete-12	120362	03/01/2013	-1	78.773	10.414
ct92-M305Pete-12	120362	03/01/2013	-2	78.778	10.423
ct92-M305Pete-12	120362	03/01/2013	-2	78.778	10.428
ct92-M305Pete-12	120362	03/01/2013	-2	78.775	10.448
ct92-M305Pete-12	120362	03/01/2013	-2	78.794	10.446
ct92-M305Pete-12	120362	04/01/2013	-2	78.794	10.443
ct92-M305Pete-12	120362	04/01/2013	-2	78.792	10.463
ct92-M305Pete-12	120362	04/01/2013	-2	78.792	10.352
ct92-M305Pete-12	120362	04/01/2013	-2	78.79	10.338
ct92-M305Pete-12	120362	04/01/2013	-2	78.817	10.314
ct92-M305Pete-12	120362	04/01/2013	-2	78.818	10.31
ct92-M305Pete-12	120362	04/01/2013	-1	78.821	10.352
ct92-M305Pete-12	120362	04/01/2013	-2	78.831	10.31
ct92-M305Pete-12	120362	04/01/2013	-2	78.829	10.297
ct92-M305Pete-12	120362	04/01/2013	-2	78.829	10.272
ct92-M305Pete-12	120362	04/01/2013	-2	78.828	10.294
ct92-M305Pete-12	120362	04/01/2013	-2	78.828	10.289
ct92-M305Pete-12	120362	04/01/2013	-1	78.835	10.272
ct92-M305Pete-12	120362	04/01/2013	-1	78.855	10.379
ct92-M305Pete-12	120362	04/01/2013	-1	78.843	10.33
ct92-M305Pete-12	120362	04/01/2013	-2	78.85	10.325
ct92-M305Pete-12	120362	04/01/2013	-2	78.855	10.329
ct92-M305Pete-12	120362	04/01/2013	-2	78.855	10.323
ct92-M305Pete-12	120362	04/01/2013	-2	78.843	10.356
ct92-M305Pete-12	120362	04/01/2013	-2	78.844	10.363
ct92-M305Pete-12	120362	04/01/2013	-2	78.84	10.339
ct92-M305Pete-12	120362	04/01/2013	-2	78.853	10.341
ct92-M305Pete-12	120362	04/01/2013	-2	78.832	10.329
ct92-M305Pete-12	120362	04/01/2013	-2	78.846	10.36
ct92-M305Pete-12	120362	04/01/2013	-2	78.88	10.3
ct92-M305Pete-12	120362	04/01/2013	-1	78.873	10.311
ct92-M305Pete-12	120362	04/01/2013	-2	78.879	10.36
ct92-M305Pete-12	120362	04/01/2013	-2	78.871	10.355
ct92-M305Pete-12	120362	04/01/2013	-2	78.836	10.407
ct92-M305Pete-12	120362	04/01/2013	-2	78.872	10.346
ct92-M305Pete-12	120362	04/01/2013	-2	78.878	10.351
ct92-M305Pete-12	120362	04/01/2013	-2	78.878	10.351
ct92-M305Pete-12	120362	04/01/2013	-1	78.885	10.3
ct92-M305Pete-12	120362	04/01/2013	-1	78.855	10.323
ct92-M305Pete-12	120362	04/01/2013	-2	78.862	10.323
ct92-M305Pete-12	120362	04/01/2013	-2	78.863	10.326
ct92-M305Pete-12	120362	04/01/2013	-1	78.862	10.41
ct92-M305Pete-12	120362	04/01/2013	-2	78.902	10.347
ct92-M305Pete-12	120362	04/01/2013	-2	78.864	10.401
ct92-M305Pete-12	120362	04/01/2013	-1	78.901	10.339
ct92-M305Pete-12	120362	04/01/2013	-2	78.901	10.366
ct92-M305Pete-12	120362	04/01/2013	0	78.906	10.36
ct92-M305Pete-12	120362	04/01/2013	-1	78.911	10.417
ct92-M305Pete-12	120362	04/01/2013	-1	78.912	10.417
ct92-M305Pete-12	120362	04/01/2013	-1	78.872	10.3
ct92-M305Pete-12	120362	04/01/2013	-1	78.92	10.435
ct92-M305Pete-12	120362	04/01/2013	-2	78.929	10.477
ct92-M305Pete-12	120362	04/01/2013	-2	78.933	10.407
ct92-M305Pete-12	120362	04/01/2013	-2	78.914	10.411
ct92-M305Pete-12	120362	04/01/2013	-2	78.913	10.414
ct92-M305Pete-12	120362	04/01/2013	-2	78.917	10.397
ct92-M305Pete-12	120362	04/01/2013	-2	78.925	10.365
ct92-M305Pete-12	120362	04/01/2013	-1	78.931	10.364
ct92-M305Pete-12	120362	04/01/2013	-2	78.933	10.372

ct92-M305Pete-12	120362	04/01/2013	-2	78.926	10.37
ct92-M305Pete-12	120362	04/01/2013	-2	78.932	10.372
ct92-M305Pete-12	120362	04/01/2013	-2	78.924	10.36
ct92-M305Pete-12	120362	04/01/2013	1	78.925	10.374
ct92-M305Pete-12	120362	04/01/2013	-2	78.927	10.37
ct92-M305Pete-12	120362	04/01/2013	-2	78.925	10.363
ct92-M305Pete-12	120362	04/01/2013	-2	78.927	10.393
ct92-M305Pete-12	120362	04/01/2013	-2	78.919	10.347
ct92-M305Pete-12	120362	04/01/2013	2	78.947	10.486
ct92-M305Pete-12	120362	04/01/2013	3	78.948	10.49
ct92-M305Pete-12	120362	04/01/2013	1	78.953	10.452
ct92-M305Pete-12	120362	04/01/2013	1	78.953	10.502
ct92-M305Pete-12	120362	04/01/2013	3	78.955	10.499
ct92-M305Pete-12	120362	05/01/2013	-2	78.971	10.555
ct92-M305Pete-12	120362	05/01/2013	-2	78.949	10.536
ct92-M305Pete-12	120362	05/01/2013	-2	78.943	10.599
ct92-M305Pete-12	120362	05/01/2013	-1	78.925	10.467
ct92-M305Pete-12	120362	05/01/2013	-2	78.941	10.602
ct92-M305Pete-12	120362	05/01/2013	-2	78.937	10.538
ct92-M305Pete-12	120362	05/01/2013	-2	78.937	10.578
ct92-M305Pete-12	120362	05/01/2013	-2	78.937	10.576
ct92-M305Pete-12	120362	05/01/2013	-2	78.938	10.573
ct92-M305Pete-12	120362	05/01/2013	-2	78.935	10.524
ct92-M305Pete-12	120362	05/01/2013	-2	78.939	10.518
ct92-M305Pete-12	120362	05/01/2013	-2	78.938	10.509
ct92-M305Pete-12	120362	05/01/2013	-1	78.933	10.59
ct92-M305Pete-12	120362	05/01/2013	-1	78.953	10.452
ct92-M305Pete-12	120362	05/01/2013	-2	78.948	10.453
ct92-M305Pete-12	120362	05/01/2013	-2	78.952	10.463
ct92-M305Pete-12	120362	05/01/2013	-2	78.951	10.475
ct92-M305Pete-12	120362	05/01/2013	-1	78.973	10.413
ct92-M305Pete-12	120362	05/01/2013	-1	78.971	10.421
ct92-M305Pete-12	120362	05/01/2013	-2	78.976	10.389
ct92-M305Pete-12	120362	05/01/2013	-2	78.98	10.361
ct92-M305Pete-12	120362	05/01/2013	-2	78.975	10.391
ct92-M305Pete-12	120362	05/01/2013	-1	78.96	10.492
ct92-M305Pete-12	120362	05/01/2013	-1	78.938	10.463
ct92-M305Pete-12	120362	05/01/2013	-1	78.945	10.349
ct92-M305Pete-12	120362	05/01/2013	-2	78.931	10.383
ct92-M305Pete-12	120362	05/01/2013	-2	78.933	10.356
ct92-M305Pete-12	120362	05/01/2013	-2	78.93	10.384
ct92-M305Pete-12	120362	05/01/2013	0	78.93	10.445
ct92-M305Pete-12	120362	05/01/2013	-1	78.939	10.282
ct92-M305Pete-12	120362	05/01/2013	-1	78.945	10.414
ct92-M305Pete-12	120362	05/01/2013	-2	78.928	10.441
ct92-M305Pete-12	120362	05/01/2013	-2	78.95	10.319
ct92-M305Pete-12	120362	05/01/2013	1	78.923	10.474
ct92-M305Pete-12	120362	05/01/2013	-2	78.921	10.487
ct92-M305Pete-12	120362	05/01/2013	-1	78.908	10.457
ct92-M305Pete-12	120362	05/01/2013	-2	78.926	10.478
ct92-M305Pete-12	120362	05/01/2013	-1	78.912	10.499
ct92-M305Pete-12	120362	05/01/2013	-2	78.936	10.413
ct92-M305Pete-12	120362	05/01/2013	-2	78.921	10.567
ct92-M305Pete-12	120362	05/01/2013	0	78.908	10.612
ct92-M305Pete-12	120362	05/01/2013	-2	78.915	10.602
ct92-M305Pete-12	120362	05/01/2013	-1	78.924	10.634
ct92-M305Pete-12	120362	05/01/2013	-2	78.916	10.598
ct92-M305Pete-12	120362	05/01/2013	-2	78.904	10.627
ct92-M305Pete-12	120362	05/01/2013	-1	78.912	10.583
ct92-M305Pete-12	120362	05/01/2013	-2	78.91	10.616
ct92-M305Pete-12	120362	05/01/2013	-1	78.915	10.611
ct92-M305Pete-12	120362	05/01/2013	-2	78.915	10.6
ct92-M305Pete-12	120362	05/01/2013	-2	78.914	10.601
ct92-M305Pete-12	120362	05/01/2013	-1	78.908	10.616
ct92-M305Pete-12	120362	05/01/2013	-2	78.911	10.657
ct92-M305Pete-12	120362	05/01/2013	-2	78.908	10.617
ct92-M305Pete-12	120362	05/01/2013	-2	78.911	10.654
ct92-M305Pete-12	120362	05/01/2013	-2	78.907	10.636

ct92-M305Pete-12	120362	05/01/2013	-2	78.908	10.633
ct92-M305Pete-12	120362	05/01/2013	-2	78.908	10.635
ct92-M305Pete-12	120362	05/01/2013	-2	78.911	10.633
ct92-M305Pete-12	120362	05/01/2013	-2	78.906	10.642
ct92-M305Pete-12	120362	05/01/2013	-2	78.906	10.634
ct92-M305Pete-12	120362	05/01/2013	-2	78.904	10.576
ct92-M305Pete-12	120362	05/01/2013	-2	78.891	10.647
ct92-M305Pete-12	120362	05/01/2013	-1	78.905	10.652
ct92-M305Pete-12	120362	05/01/2013	-2	78.903	10.663
ct92-M305Pete-12	120362	05/01/2013	-2	78.9	10.66
ct92-M305Pete-12	120362	05/01/2013	-2	78.903	10.643
ct92-M305Pete-12	120362	06/01/2013	-2	78.899	10.674
ct92-M305Pete-12	120362	06/01/2013	-2	78.894	10.636
ct92-M305Pete-12	120362	06/01/2013	-2	78.905	10.551
ct92-M305Pete-12	120362	06/01/2013	-1	78.907	10.613
ct92-M305Pete-12	120362	06/01/2013	-2	78.897	10.595
ct92-M305Pete-12	120362	06/01/2013	-2	78.898	10.592
ct92-M305Pete-12	120362	06/01/2013	-1	78.903	10.694
ct92-M305Pete-12	120362	06/01/2013	-1	78.912	10.578
ct92-M305Pete-12	120362	06/01/2013	-2	78.912	10.575
ct92-M305Pete-12	120362	06/01/2013	-2	78.914	10.573
ct92-M305Pete-12	120362	06/01/2013	-1	78.911	10.566
ct92-M305Pete-12	120362	06/01/2013	-2	78.922	10.541
ct92-M305Pete-12	120362	06/01/2013	-1	78.918	10.551
ct92-M305Pete-12	120362	06/01/2013	-2	78.916	10.545
ct92-M305Pete-12	120362	06/01/2013	-2	78.915	10.551
ct92-M305Pete-12	120362	06/01/2013	-1	78.9	10.651
ct92-M305Pete-12	120362	06/01/2013	-2	78.902	10.652
ct92-M305Pete-12	120362	06/01/2013	-2	78.902	10.645
ct92-M305Pete-12	120362	06/01/2013	-2	78.884	10.596
ct92-M305Pete-12	120362	06/01/2013	-2	78.911	10.666
ct92-M305Pete-12	120362	06/01/2013	-2	78.934	10.675
ct92-M305Pete-12	120362	06/01/2013	-2	78.925	10.716
ct92-M305Pete-12	120362	06/01/2013	-2	78.907	10.597
ct92-M305Pete-12	120362	06/01/2013	-2	78.925	10.699
ct92-M305Pete-12	120362	06/01/2013	-2	78.925	10.698
ct92-M305Pete-12	120362	06/01/2013	-2	78.891	10.611
ct92-M305Pete-12	120362	06/01/2013	-2	78.909	10.618
ct92-M305Pete-12	120362	06/01/2013	-1	78.878	10.723
ct92-M305Pete-12	120362	06/01/2013	-2	78.918	10.702
ct92-M305Pete-12	120362	06/01/2013	-2	78.924	10.7
ct92-M305Pete-12	120362	06/01/2013	-2	78.918	10.64
ct92-M305Pete-12	120362	06/01/2013	-2	78.917	10.639
ct92-M305Pete-12	120362	06/01/2013	-2	78.923	10.659
ct92-M305Pete-12	120362	06/01/2013	-2	78.91	10.591
ct92-M305Pete-12	120362	06/01/2013	-2	78.918	10.733
ct92-M305Pete-12	120362	06/01/2013	-1	78.91	10.725
ct92-M305Pete-12	120362	06/01/2013	-2	78.921	10.715
ct92-M305Pete-12	120362	06/01/2013	-2	78.919	10.702
ct92-M305Pete-12	120362	06/01/2013	0	78.939	10.694
ct92-M305Pete-12	120362	06/01/2013	-2	78.926	10.719
ct92-M305Pete-12	120362	06/01/2013	-2	78.931	10.76
ct92-M305Pete-12	120362	06/01/2013	-2	78.908	10.732
ct92-M305Pete-12	120362	06/01/2013	-2	78.917	10.792
ct92-M305Pete-12	120362	06/01/2013	-2	78.907	10.731
ct92-M305Pete-12	120362	06/01/2013	-2	78.913	10.819
ct92-M305Pete-12	120362	06/01/2013	-2	78.911	10.755
ct92-M305Pete-12	120362	06/01/2013	-1	78.927	10.794
ct92-M305Pete-12	120362	06/01/2013	-2	78.908	10.749
ct92-M305Pete-12	120362	06/01/2013	-2	78.924	10.862
ct92-M305Pete-12	120362	06/01/2013	-1	78.903	10.831
ct92-M305Pete-12	120362	06/01/2013	-1	78.906	10.953
ct92-M305Pete-12	120362	06/01/2013	-2	78.916	10.898
ct92-M305Pete-12	120362	06/01/2013	-2	78.893	10.828
ct92-M305Pete-12	120362	06/01/2013	-2	78.915	10.822
ct92-M305Pete-12	120362	06/01/2013	-2	78.902	10.804
ct92-M305Pete-12	120362	06/01/2013	-2	78.914	10.794
ct92-M305Pete-12	120362	06/01/2013	-2	78.913	10.872

ct92-M305Pete-12	120362	06/01/2013	-2	78.926	10.886
ct92-M305Pete-12	120362	06/01/2013	-2	78.921	10.883
ct92-M305Pete-12	120362	06/01/2013	-2	78.916	10.726
ct92-M305Pete-12	120362	06/01/2013	-2	78.913	10.723
ct92-M305Pete-12	120362	06/01/2013	-2	78.89	10.806
ct92-M305Pete-12	120362	06/01/2013	-2	78.924	10.74
ct92-M305Pete-12	120362	06/01/2013	-2	78.9	10.833
ct92-M305Pete-12	120362	06/01/2013	-2	78.901	10.834
ct92-M305Pete-12	120362	06/01/2013	-2	78.904	10.855
ct92-M305Pete-12	120362	06/01/2013	-1	78.899	10.84
ct92-M305Pete-12	120362	06/01/2013	-1	78.899	10.844
ct92-M305Pete-12	120362	06/01/2013	-2	78.903	10.84
ct92-M305Pete-12	120362	06/01/2013	-2	78.899	10.824
ct92-M305Pete-12	120362	06/01/2013	-2	78.925	10.816
ct92-M305Pete-12	120362	06/01/2013	-2	78.925	10.824
ct92-M305Pete-12	120362	06/01/2013	-2	78.927	10.773
ct92-M305Pete-12	120362	06/01/2013	-2	78.925	10.794
ct92-M305Pete-12	120362	06/01/2013	-2	78.915	10.777
ct92-M305Pete-12	120362	06/01/2013	-2	78.917	10.742
ct92-M305Pete-12	120362	06/01/2013	-2	78.925	10.801
ct92-M305Pete-12	120362	06/01/2013	-2	78.921	10.81
ct92-M305Pete-12	120362	06/01/2013	-2	78.925	10.807
ct92-M305Pete-12	120362	06/01/2013	-2	78.921	10.758
ct92-M305Pete-12	120362	06/01/2013	-2	78.918	10.752
ct92-M305Pete-12	120362	07/01/2013	-2	78.928	10.745
ct92-M305Pete-12	120362	07/01/2013	-2	78.927	10.741
ct92-M305Pete-12	120362	07/01/2013	-2	78.937	10.737
ct92-M305Pete-12	120362	07/01/2013	-2	78.942	10.754
ct92-M305Pete-12	120362	07/01/2013	-2	78.942	10.802
ct92-M305Pete-12	120362	07/01/2013	-2	78.938	10.779
ct92-M305Pete-12	120362	07/01/2013	-2	78.94	10.782
ct92-M305Pete-12	120362	07/01/2013	-2	78.978	10.614
ct92-M305Pete-12	120362	07/01/2013	-2	78.95	10.698
ct92-M305Pete-12	120362	07/01/2013	-2	78.945	10.707
ct92-M305Pete-12	120362	07/01/2013	-2	78.924	10.792
ct92-M305Pete-12	120362	07/01/2013	-2	78.926	10.794
ct92-M305Pete-12	120362	07/01/2013	-2	78.9	10.908
ct92-M305Pete-12	120362	07/01/2013	-2	78.899	10.901
ct92-M305Pete-12	120362	07/01/2013	-2	78.923	10.886
ct92-M305Pete-12	120362	07/01/2013	-2	78.918	10.88
ct92-M305Pete-12	120362	07/01/2013	-2	78.94	10.772
ct92-M305Pete-12	120362	07/01/2013	-2	78.909	10.774
ct92-M305Pete-12	120362	07/01/2013	-2	78.889	10.838
ct92-M305Pete-12	120362	07/01/2013	-2	78.884	10.851
ct92-M305Pete-12	120362	07/01/2013	-1	78.885	10.822
ct92-M305Pete-12	120362	07/01/2013	-1	78.895	10.863
ct92-M305Pete-12	120362	07/01/2013	-2	78.892	10.782
ct92-M305Pete-12	120362	07/01/2013	-2	78.895	10.882
ct92-M305Pete-12	120362	07/01/2013	-2	78.895	10.882
ct92-M305Pete-12	120362	07/01/2013	-2	78.884	10.89
ct92-M305Pete-12	120362	07/01/2013	-2	78.884	10.884
ct92-M305Pete-12	120362	07/01/2013	-2	78.889	10.835
ct92-M305Pete-12	120362	07/01/2013	-2	78.859	10.893
ct92-M305Pete-12	120362	07/01/2013	-1	78.881	10.807
ct92-M305Pete-12	120362	07/01/2013	-2	78.891	10.828
ct92-M305Pete-12	120362	07/01/2013	-2	78.892	10.893
ct92-M305Pete-12	120362	07/01/2013	-2	78.894	10.886
ct92-M305Pete-12	120362	07/01/2013	-2	78.894	10.876
ct92-M305Pete-12	120362	07/01/2013	-2	78.887	10.907
ct92-M305Pete-12	120362	07/01/2013	0	78.891	10.894
ct92-M305Pete-12	120362	07/01/2013	-2	78.894	10.872
ct92-M305Pete-12	120362	07/01/2013	-2	78.887	10.871
ct92-M305Pete-12	120362	07/01/2013	-1	78.886	10.858
ct92-M305Pete-12	120362	07/01/2013	-2	78.886	10.828
ct92-M305Pete-12	120362	07/01/2013	1	78.908	10.876
ct92-M305Pete-12	120362	07/01/2013	-2	78.888	10.849
ct92-M305Pete-12	120362	07/01/2013	1	78.896	10.922
ct92-M305Pete-12	120362	07/01/2013	-2	78.902	10.91

ct92-M305Pete-12	120362	07/01/2013	-2	78.902	10.901
ct92-M305Pete-12	120362	07/01/2013	-1	78.908	10.856
ct92-M305Pete-12	120362	07/01/2013	-2	78.876	10.851
ct92-M305Pete-12	120362	07/01/2013	-2	78.907	10.866
ct92-M305Pete-12	120362	07/01/2013	-1	78.876	10.891
ct92-M305Pete-12	120362	07/01/2013	-2	78.878	10.897
ct92-M305Pete-12	120362	07/01/2013	-2	78.887	10.897
ct92-M305Pete-12	120362	07/01/2013	-2	78.891	10.884
ct92-M305Pete-12	120362	07/01/2013	-2	78.884	10.877
ct92-M305Pete-12	120362	07/01/2013	-1	78.883	10.876
ct92-M305Pete-12	120362	07/01/2013	-2	78.888	10.81
ct92-M305Pete-12	120362	07/01/2013	-2	78.878	10.809
ct92-M305Pete-12	120362	07/01/2013	-2	78.874	10.814
ct92-M305Pete-12	120362	07/01/2013	-2	78.887	10.813
ct92-M305Pete-12	120362	07/01/2013	-2	78.886	10.815
ct92-M305Pete-12	120362	07/01/2013	-2	78.889	10.817
ct92-M305Pete-12	120362	07/01/2013	-2	78.886	10.81
ct92-M305Pete-12	120362	07/01/2013	-2	78.884	10.815
ct92-M305Pete-12	120362	07/01/2013	-2	78.888	10.818
ct92-M305Pete-12	120362	07/01/2013	-2	78.884	10.799
ct92-M305Pete-12	120362	07/01/2013	-1	78.894	10.746
ct92-M305Pete-12	120362	07/01/2013	-2	78.894	10.742
ct92-M305Pete-12	120362	07/01/2013	-2	78.892	10.729
ct92-M305Pete-12	120362	07/01/2013	0	78.891	10.73
ct92-M305Pete-12	120362	07/01/2013	-1	78.893	10.665
ct92-M305Pete-12	120362	08/01/2013	-2	78.89	10.698
ct92-M305Pete-12	120362	08/01/2013	-2	78.891	10.674
ct92-M305Pete-12	120362	08/01/2013	-2	78.892	10.684
ct92-M305Pete-12	120362	08/01/2013	1	78.896	10.669
ct92-M305Pete-12	120362	08/01/2013	1	78.896	10.667
ct92-M305Pete-12	120362	08/01/2013	-1	78.903	10.744
ct92-M305Pete-12	120362	08/01/2013	-2	78.904	10.753
ct92-M305Pete-12	120362	08/01/2013	-1	78.91	10.813
ct92-M305Pete-12	120362	08/01/2013	-2	78.91	10.811
ct92-M305Pete-12	120362	08/01/2013	-2	78.912	10.824
ct92-M305Pete-12	120362	08/01/2013	-2	78.912	10.84
ct92-M305Pete-12	120362	08/01/2013	-1	78.894	10.904
ct92-M305Pete-12	120362	08/01/2013	-1	78.906	10.907
ct92-M305Pete-12	120362	08/01/2013	-2	78.899	10.857
ct92-M305Pete-12	120362	08/01/2013	-2	78.896	10.862
ct92-M305Pete-12	120362	08/01/2013	-2	78.895	10.826
ct92-M305Pete-12	120362	08/01/2013	-1	78.894	10.855
ct92-M305Pete-12	120362	08/01/2013	-1	78.919	10.558
ct92-M305Pete-12	120362	08/01/2013	-2	78.904	10.646
ct92-M305Pete-12	120362	08/01/2013	-1	78.9	10.857
ct92-M305Pete-12	120362	08/01/2013	-2	78.899	10.852
ct92-M305Pete-12	120362	08/01/2013	-2	78.886	10.84
ct92-M305Pete-12	120362	08/01/2013	-1	78.882	10.881
ct92-M305Pete-12	120362	08/01/2013	-1	78.887	10.841
ct92-M305Pete-12	120362	08/01/2013	-1	78.882	10.869
ct92-M305Pete-12	120362	08/01/2013	-1	78.886	10.871
ct92-M305Pete-12	120362	08/01/2013	-2	78.882	10.87
ct92-M305Pete-12	120362	08/01/2013	-2	78.899	11.004
ct92-M305Pete-12	120362	08/01/2013	-2	78.899	10.996
ct92-M305Pete-12	120362	08/01/2013	-2	78.893	10.979
ct92-M305Pete-12	120362	08/01/2013	-2	78.885	10.98
ct92-M305Pete-12	120362	08/01/2013	-2	78.885	10.986
ct92-M305Pete-12	120362	08/01/2013	-2	78.894	10.858
ct92-M305Pete-12	120362	08/01/2013	-2	78.89	10.823
ct92-M305Pete-12	120362	08/01/2013	-2	78.901	11.029
ct92-M305Pete-12	120362	08/01/2013	-2	78.882	10.81
ct92-M305Pete-12	120362	08/01/2013	-2	78.884	10.801
ct92-M305Pete-12	120362	08/01/2013	-2	78.886	10.965
ct92-M305Pete-12	120362	08/01/2013	-2	78.886	10.948
ct92-M305Pete-12	120362	08/01/2013	-2	78.884	10.858
ct92-M305Pete-12	120362	08/01/2013	-2	78.882	10.856
ct92-M305Pete-12	120362	08/01/2013	-1	78.881	10.718
ct92-M305Pete-12	120362	08/01/2013	-2	78.906	10.698

ct92-M305Pete-12	120362	08/01/2013	-2	78.911	10.776
ct92-M305Pete-12	120362	08/01/2013	-2	78.913	10.667
ct92-M305Pete-12	120362	08/01/2013	-1	78.896	10.665
ct92-M305Pete-12	120362	08/01/2013	-2	78.9	10.673
ct92-M305Pete-12	120362	08/01/2013	-2	78.895	10.692
ct92-M305Pete-12	120362	08/01/2013	-2	78.897	10.712
ct92-M305Pete-12	120362	08/01/2013	-2	78.899	10.704
ct92-M305Pete-12	120362	08/01/2013	-2	78.896	10.709
ct92-M305Pete-12	120362	08/01/2013	-2	78.896	10.706
ct92-M305Pete-12	120362	08/01/2013	-2	78.897	10.704
ct92-M305Pete-12	120362	08/01/2013	-2	78.899	10.685
ct92-M305Pete-12	120362	08/01/2013	-2	78.901	10.675
ct92-M305Pete-12	120362	08/01/2013	-2	78.931	10.73
ct92-M305Pete-12	120362	08/01/2013	-2	78.894	10.716
ct92-M305Pete-12	120362	08/01/2013	-2	78.932	10.731
ct92-M305Pete-12	120362	08/01/2013	-2	78.93	10.761
ct92-M305Pete-12	120362	08/01/2013	-2	78.93	10.672
ct92-M305Pete-12	120362	08/01/2013	-2	78.93	10.67
ct92-M305Pete-12	120362	08/01/2013	-2	78.93	10.675
ct92-M305Pete-12	120362	08/01/2013	-2	78.939	10.526
ct92-M305Pete-12	120362	08/01/2013	-2	78.905	10.601
ct92-M305Pete-12	120362	08/01/2013	-2	78.909	10.616
ct92-M305Pete-12	120362	08/01/2013	-2	78.886	10.708
ct92-M305Pete-12	120362	08/01/2013	-2	78.906	10.663
ct92-M305Pete-12	120362	08/01/2013	-2	78.904	10.619
ct92-M305Pete-12	120362	08/01/2013	-2	78.911	10.608
ct92-M305Pete-12	120362	08/01/2013	-2	78.908	10.664
ct92-M305Pete-12	120362	08/01/2013	-2	78.926	10.715
ct92-M305Pete-12	120362	08/01/2013	-2	78.919	10.626
ct92-M305Pete-12	120362	08/01/2013	-2	78.925	10.654
ct92-M305Pete-12	120362	08/01/2013	-2	78.918	10.629
ct92-M305Pete-12	120362	09/01/2013	-2	78.918	10.69
ct92-M305Pete-12	120362	09/01/2013	-2	78.919	10.668
ct92-M305Pete-12	120362	09/01/2013	-2	78.941	10.625
ct92-M305Pete-12	120362	09/01/2013	-2	78.941	10.608
ct92-M305Pete-12	120362	09/01/2013	-2	78.943	10.477
ct92-M305Pete-12	120362	09/01/2013	-2	78.941	10.431
ct92-M305Pete-12	120362	09/01/2013	-2	78.945	10.413
ct92-M305Pete-12	120362	09/01/2013	-2	78.945	10.428
ct92-M305Pete-12	120362	09/01/2013	-1	78.929	10.488
ct92-M305Pete-12	120362	09/01/2013	-2	78.93	10.471
ct92-M305Pete-12	120362	09/01/2013	-2	78.938	10.439
ct92-M305Pete-12	120362	09/01/2013	0	78.937	10.408
ct92-M305Pete-12	120362	09/01/2013	-2	78.939	10.404
ct92-M305Pete-12	120362	09/01/2013	-2	78.939	10.417
ct92-M305Pete-12	120362	09/01/2013	-2	78.936	10.417
ct92-M305Pete-12	120362	09/01/2013	0	78.956	10.449
ct92-M305Pete-12	120362	09/01/2013	-2	78.97	10.393
ct92-M305Pete-12	120362	09/01/2013	-2	78.935	10.581
ct92-M305Pete-12	120362	09/01/2013	-2	78.954	10.466
ct92-M305Pete-12	120362	09/01/2013	-2	78.97	10.395
ct92-M305Pete-12	120362	09/01/2013	-2	78.955	10.491
ct92-M305Pete-12	120362	09/01/2013	-2	78.945	10.49
ct92-M305Pete-12	120362	09/01/2013	-2	78.946	10.483
ct92-M305Pete-12	120362	09/01/2013	-1	78.948	10.572
ct92-M305Pete-12	120362	09/01/2013	-2	78.957	10.505
ct92-M305Pete-12	120362	09/01/2013	-2	78.955	10.51
ct92-M305Pete-12	120362	09/01/2013	-2	78.934	10.615
ct92-M305Pete-12	120362	09/01/2013	-1	78.944	10.633
ct92-M305Pete-12	120362	09/01/2013	-1	78.935	10.587
ct92-M305Pete-12	120362	09/01/2013	-2	78.927	10.569
ct92-M305Pete-12	120362	09/01/2013	-2	78.948	10.596
ct92-M305Pete-12	120362	09/01/2013	-2	78.907	10.53
ct92-M305Pete-12	120362	09/01/2013	-2	78.915	10.481
ct92-M305Pete-12	120362	09/01/2013	-2	78.923	10.485
ct92-M305Pete-12	120362	09/01/2013	-1	78.934	10.449
ct92-M305Pete-12	120362	09/01/2013	-2	78.913	10.488
ct92-M305Pete-12	120362	09/01/2013	-2	78.926	10.504

ct92-M305Pete-12	120362	09/01/2013	-2	78.93	10.525
ct92-M305Pete-12	120362	09/01/2013	-2	78.925	10.496
ct92-M305Pete-12	120362	09/01/2013	-2	78.917	10.46
ct92-M305Pete-12	120362	09/01/2013	-2	78.921	10.466
ct92-M305Pete-12	120362	09/01/2013	-1	78.928	10.44
ct92-M305Pete-12	120362	09/01/2013	-1	78.927	10.43
ct92-M305Pete-12	120362	09/01/2013	-2	78.925	10.424
ct92-M305Pete-12	120362	09/01/2013	0	78.943	10.336
ct92-M305Pete-12	120362	09/01/2013	-1	78.923	10.428
ct92-M305Pete-12	120362	09/01/2013	1	78.926	10.469
ct92-M305Pete-12	120362	09/01/2013	-2	78.928	10.45
ct92-M305Pete-12	120362	09/01/2013	-2	78.925	10.469
ct92-M305Pete-12	120362	09/01/2013	-1	78.928	10.546
ct92-M305Pete-12	120362	09/01/2013	-2	78.914	10.441
ct92-M305Pete-12	120362	09/01/2013	-2	78.912	10.502
ct92-M305Pete-12	120362	09/01/2013	-2	78.913	10.574
ct92-M305Pete-12	120362	09/01/2013	-2	78.91	10.677
ct92-M305Pete-12	120362	10/01/2013	-2	78.906	10.695
ct92-M305Pete-12	120362	10/01/2013	-2	78.916	10.718
ct92-M305Pete-12	120362	10/01/2013	-1	78.911	10.838
ct92-M305Pete-12	120362	10/01/2013	-2	78.918	10.742
ct92-M305Pete-12	120362	10/01/2013	-2	78.94	10.693
ct92-M305Pete-12	120362	10/01/2013	-2	78.944	10.69
ct92-M305Pete-12	120362	10/01/2013	-2	78.94	10.66
ct92-M305Pete-12	120362	10/01/2013	-2	78.944	10.651
ct92-M305Pete-12	120362	10/01/2013	-2	78.943	10.641
ct92-M305Pete-12	120362	10/01/2013	-1	78.942	10.635
ct92-M305Pete-12	120362	10/01/2013	-2	78.931	10.708
ct92-M305Pete-12	120362	10/01/2013	-2	78.927	10.657
ct92-M305Pete-12	120362	10/01/2013	-2	78.92	10.618
ct92-M305Pete-12	120362	10/01/2013	-2	78.92	10.62
ct92-M305Pete-12	120362	10/01/2013	-2	78.933	10.646
ct92-M305Pete-12	120362	10/01/2013	-2	78.935	10.55
ct92-M305Pete-12	120362	10/01/2013	-1	78.937	10.547
ct92-M305Pete-12	120362	10/01/2013	-2	78.928	10.545
ct92-M305Pete-12	120362	10/01/2013	-2	78.934	10.57
ct92-M305Pete-12	120362	10/01/2013	-2	78.935	10.564
ct92-M305Pete-12	120362	10/01/2013	-2	78.948	10.479
ct92-M305Pete-12	120362	10/01/2013	-1	78.964	10.581
ct92-M305Pete-12	120362	10/01/2013	-2	78.928	10.53
ct92-M305Pete-12	120362	10/01/2013	-2	78.942	10.604
ct92-M305Pete-12	120362	10/01/2013	-2	78.94	10.608
ct92-M305Pete-12	120362	10/01/2013	-2	78.946	10.641
ct92-M305Pete-12	120362	10/01/2013	-1	78.944	10.686
ct92-M305Pete-12	120362	10/01/2013	-2	78.944	10.689
ct92-M305Pete-12	120362	10/01/2013	-2	78.933	10.618
ct92-M305Pete-12	120362	10/01/2013	-1	78.918	10.586
ct92-M305Pete-12	120362	10/01/2013	-2	78.911	10.615
ct92-M305Pete-12	120362	10/01/2013	-2	78.945	10.664
ct92-M305Pete-12	120362	10/01/2013	-2	78.945	10.661
ct92-M305Pete-12	120362	10/01/2013	-2	78.933	10.624
ct92-M305Pete-12	120362	10/01/2013	-2	78.931	10.586
ct92-M305Pete-12	120362	10/01/2013	-1	78.932	10.582
ct92-M305Pete-12	120362	10/01/2013	-2	78.923	10.554
ct92-M305Pete-12	120362	10/01/2013	-2	78.915	10.641
ct92-M305Pete-12	120362	10/01/2013	-2	78.919	10.569
ct92-M305Pete-12	120362	10/01/2013	-2	78.924	10.605
ct92-M305Pete-12	120362	10/01/2013	-2	78.923	10.63
ct92-M305Pete-12	120362	10/01/2013	-2	78.919	10.599
ct92-M305Pete-12	120362	10/01/2013	-2	78.904	10.645
ct92-M305Pete-12	120362	10/01/2013	-2	78.902	10.627
ct92-M305Pete-12	120362	10/01/2013	-2	78.906	10.634
ct92-M305Pete-12	120362	10/01/2013	-2	78.917	10.659
ct92-M305Pete-12	120362	10/01/2013	-2	78.918	10.645
ct92-M305Pete-12	120362	10/01/2013	-2	78.909	10.625
ct92-M305Pete-12	120362	10/01/2013	-2	78.893	10.583
ct92-M305Pete-12	120362	10/01/2013	-2	78.907	10.598
ct92-M305Pete-12	120362	10/01/2013	-2	78.896	10.589

ct92-M305Pete-12	120362	10/01/2013	-2	78.907	10.604
ct92-M305Pete-12	120362	10/01/2013	-2	78.888	10.568
ct92-M305Pete-12	120362	10/01/2013	-2	78.905	10.593
ct92-M305Pete-12	120362	10/01/2013	-2	78.903	10.587
ct92-M305Pete-12	120362	10/01/2013	-2	78.906	10.539
ct92-M305Pete-12	120362	10/01/2013	-2	78.907	10.538
ct92-M305Pete-12	120362	10/01/2013	-2	78.902	10.6
ct92-M305Pete-12	120362	11/01/2013	-2	78.938	10.729
ct92-M305Pete-12	120362	11/01/2013	-1	78.962	10.677
ct92-M305Pete-12	120362	11/01/2013	-1	78.943	10.762
ct92-M305Pete-12	120362	11/01/2013	-1	78.965	10.581
ct92-M305Pete-12	120362	11/01/2013	-2	78.972	10.592
ct92-M305Pete-12	120362	11/01/2013	-2	78.969	10.626
ct92-M305Pete-12	120362	11/01/2013	-2	78.971	10.567
ct92-M305Pete-12	120362	11/01/2013	-2	78.959	10.566
ct92-M305Pete-12	120362	11/01/2013	-1	78.972	10.578
ct92-M305Pete-12	120362	11/01/2013	-2	78.937	10.595
ct92-M305Pete-12	120362	11/01/2013	-1	78.963	10.564
ct92-M305Pete-12	120362	11/01/2013	-2	78.947	10.573
ct92-M305Pete-12	120362	11/01/2013	-2	78.948	10.587
ct92-M305Pete-12	120362	11/01/2013	-2	78.946	10.6
ct92-M305Pete-12	120362	11/01/2013	-2	78.946	10.609
ct92-M305Pete-12	120362	11/01/2013	-2	78.945	10.616
ct92-M305Pete-12	120362	11/01/2013	-2	78.947	10.668
ct92-M305Pete-12	120362	11/01/2013	-2	78.959	10.655
ct92-M305Pete-12	120362	11/01/2013	-1	78.936	10.606
ct92-M305Pete-12	120362	11/01/2013	1	78.952	10.559
ct92-M305Pete-12	120362	11/01/2013	-2	78.932	10.622
ct92-M305Pete-12	120362	11/01/2013	-2	78.941	10.619
ct92-M305Pete-12	120362	11/01/2013	-2	78.93	10.51
ct92-M305Pete-12	120362	11/01/2013	-2	78.945	10.46
ct92-M305Pete-12	120362	11/01/2013	-2	78.952	10.423
ct92-M305Pete-12	120362	11/01/2013	-2	78.941	10.442
ct92-M305Pete-12	120362	11/01/2013	-2	78.936	10.559
ct92-M305Pete-12	120362	11/01/2013	-2	78.933	10.544
ct92-M305Pete-12	120362	11/01/2013	-2	78.945	10.493
ct92-M305Pete-12	120362	11/01/2013	-2	78.939	10.565
ct92-M305Pete-12	120362	11/01/2013	-2	78.959	10.484
ct92-M305Pete-12	120362	11/01/2013	-2	78.96	10.455
ct92-M305Pete-12	120362	11/01/2013	-1	78.915	10.633
ct92-M305Pete-12	120362	11/01/2013	-2	78.948	10.561
ct92-M305Pete-12	120362	11/01/2013	-2	78.946	10.594
ct92-M305Pete-12	120362	11/01/2013	-2	78.949	10.514
ct92-M305Pete-12	120362	11/01/2013	0	78.938	10.541
ct92-M305Pete-12	120362	11/01/2013	-2	78.946	10.491
ct92-M305Pete-12	120362	11/01/2013	0	78.958	10.564
ct92-M305Pete-12	120362	11/01/2013	-1	78.941	10.614
ct92-M305Pete-12	120362	11/01/2013	-2	78.94	10.613
ct92-M305Pete-12	120362	11/01/2013	-2	78.951	10.512
ct92-M305Pete-12	120362	11/01/2013	1	78.911	10.612
ct92-M305Pete-12	120362	11/01/2013	-1	78.911	10.575
ct92-M305Pete-12	120362	11/01/2013	-2	78.916	10.594
ct92-M305Pete-12	120362	11/01/2013	0	78.961	10.765
ct92-M305Pete-12	120362	11/01/2013	-1	78.961	10.711
ct92-M305Pete-12	120362	11/01/2013	-2	78.933	10.673
ct92-M305Pete-12	120362	11/01/2013	-2	78.93	10.696
ct92-M305Pete-12	120362	11/01/2013	-2	78.933	10.677
ct92-M305Pete-12	120362	11/01/2013	-2	78.931	10.654
ct92-M305Pete-12	120362	11/01/2013	-2	78.917	10.731
ct92-M305Pete-12	120362	11/01/2013	-2	78.914	10.719
ct92-M305Pete-12	120362	11/01/2013	-2	78.908	10.676
ct92-M305Pete-12	120362	11/01/2013	-2	78.913	10.746
ct92-M305Pete-12	120362	11/01/2013	-2	78.918	10.715
ct92-M305Pete-12	120362	11/01/2013	-2	78.911	10.746
ct92-M305Pete-12	120362	11/01/2013	-2	78.908	10.791
ct92-M305Pete-12	120362	11/01/2013	-1	78.903	10.629
ct92-M305Pete-12	120362	11/01/2013	-2	78.904	10.626
ct92-M305Pete-12	120362	11/01/2013	-2	78.917	10.68

ct92-M305Pete-12	120362	11/01/2013	-2	78.917	10.684
ct92-M305Pete-12	120362	11/01/2013	-1	78.911	10.619
ct92-M305Pete-12	120362	11/01/2013	-2	78.907	10.642
ct92-M305Pete-12	120362	11/01/2013	-2	78.919	10.581
ct92-M305Pete-12	120362	11/01/2013	-2	78.91	10.612
ct92-M305Pete-12	120362	11/01/2013	-2	78.92	10.595
ct92-M305Pete-12	120362	11/01/2013	-2	78.919	10.601
ct92-M305Pete-12	120362	11/01/2013	-2	78.914	10.554
ct92-M305Pete-12	120362	11/01/2013	-1	78.909	10.565
ct92-M305Pete-12	120362	11/01/2013	-1	78.914	10.619
ct92-M305Pete-12	120362	11/01/2013	-1	78.898	10.622
ct92-M305Pete-12	120362	11/01/2013	-1	78.901	10.682
ct92-M305Pete-12	120362	11/01/2013	-1	78.905	10.696
ct92-M305Pete-12	120362	12/01/2013	-2	78.908	10.65
ct92-M305Pete-12	120362	12/01/2013	-2	78.908	10.649
ct92-M305Pete-12	120362	12/01/2013	-1	78.918	10.648
ct92-M305Pete-12	120362	12/01/2013	-2	78.919	10.647
ct92-M305Pete-12	120362	12/01/2013	-1	78.917	10.66
ct92-M305Pete-12	120362	12/01/2013	-2	78.917	10.657
ct92-M305Pete-12	120362	12/01/2013	-2	78.911	10.631
ct92-M305Pete-12	120362	12/01/2013	-2	78.906	10.497
ct92-M305Pete-12	120362	12/01/2013	-2	78.904	10.502
ct92-M305Pete-12	120362	12/01/2013	-2	78.892	10.291
ct92-M305Pete-12	120362	12/01/2013	-2	78.903	10.498
ct92-M305Pete-12	120362	12/01/2013	-2	78.899	10.486
ct92-M305Pete-12	120362	12/01/2013	-2	78.895	10.373
ct92-M305Pete-12	120362	12/01/2013	-1	78.89	10.299
ct92-M305Pete-12	120362	12/01/2013	-2	78.891	10.263
ct92-M305Pete-12	120362	12/01/2013	-2	78.884	10.26
ct92-M305Pete-12	120362	12/01/2013	-2	78.897	10.381
ct92-M305Pete-12	120362	12/01/2013	-2	78.884	10.235
ct92-M305Pete-12	120362	12/01/2013	-1	78.882	10.353
ct92-M305Pete-12	120362	12/01/2013	-2	78.88	10.335
ct92-M305Pete-12	120362	12/01/2013	-1	78.89	10.25
ct92-M305Pete-12	120362	12/01/2013	-1	78.894	10.276
ct92-M305Pete-12	120362	12/01/2013	-2	78.89	10.252
ct92-M305Pete-12	120362	12/01/2013	-2	78.889	10.313
ct92-M305Pete-12	120362	12/01/2013	-2	78.889	10.244
ct92-M305Pete-12	120362	12/01/2013	-2	78.9	10.215
ct92-M305Pete-12	120362	12/01/2013	-2	78.871	10.147
ct92-M305Pete-12	120362	12/01/2013	-1	78.911	10.124
ct92-M305Pete-12	120362	12/01/2013	-2	78.915	10.4
ct92-M305Pete-12	120362	12/01/2013	-2	78.912	10.42
ct92-M305Pete-12	120362	12/01/2013	-1	78.943	10.287
ct92-M305Pete-12	120362	12/01/2013	-2	78.924	10.477
ct92-M305Pete-12	120362	12/01/2013	-2	78.93	10.371
ct92-M305Pete-12	120362	12/01/2013	-1	78.924	10.422
ct92-M305Pete-12	120362	12/01/2013	-2	78.927	10.453
ct92-M305Pete-12	120362	12/01/2013	-2	78.927	10.447
ct92-M305Pete-12	120362	12/01/2013	-2	78.925	10.449
ct92-M305Pete-12	120362	12/01/2013	-2	78.931	10.44
ct92-M305Pete-12	120362	12/01/2013	-2	78.93	10.448
ct92-M305Pete-12	120362	12/01/2013	-2	78.929	10.444
ct92-M305Pete-12	120362	12/01/2013	-1	78.931	10.435
ct92-M305Pete-12	120362	12/01/2013	-1	78.931	10.436
ct92-M305Pete-12	120362	12/01/2013	-2	78.944	10.465
ct92-M305Pete-12	120362	12/01/2013	-2	78.94	10.452
ct92-M305Pete-12	120362	12/01/2013	-2	78.94	10.453
ct92-M305Pete-12	120362	12/01/2013	-2	78.937	10.453
ct92-M305Pete-12	120362	12/01/2013	-1	78.951	10.626
ct92-M305Pete-12	120362	12/01/2013	-1	78.954	10.653
ct92-M305Pete-12	120362	12/01/2013	-2	78.947	10.549
ct92-M305Pete-12	120362	12/01/2013	-2	78.944	10.531
ct92-M305Pete-12	120362	12/01/2013	-1	78.942	10.494
ct92-M305Pete-12	120362	12/01/2013	-2	78.956	10.552
ct92-M305Pete-12	120362	12/01/2013	-2	78.957	10.566
ct92-M305Pete-12	120362	12/01/2013	-2	78.962	10.611
ct92-M305Pete-12	120362	12/01/2013	-2	78.961	10.606

ct92-M305Pete-12	120362	12/01/2013	-2	78.961	10.664
ct92-M305Pete-12	120362	12/01/2013	-2	78.953	10.672
ct92-M305Pete-12	120362	12/01/2013	-2	78.979	10.772
ct92-M305Pete-12	120362	12/01/2013	-2	78.984	10.821
ct92-M305Pete-12	120362	12/01/2013	-1	78.994	10.776
ct92-M305Pete-12	120362	12/01/2013	-2	79.005	10.841
ct92-M305Pete-12	120362	12/01/2013	-2	79.006	10.846
ct92-M305Pete-12	120362	13/01/2013	-2	79.008	10.933
ct92-M305Pete-12	120362	13/01/2013	-2	79.011	10.878
ct92-M305Pete-12	120362	13/01/2013	-2	79.014	10.906
ct92-M305Pete-12	120362	13/01/2013	-2	79.023	10.965
ct92-M305Pete-12	120362	13/01/2013	-2	79.099	10.975
ct92-M305Pete-12	120362	13/01/2013	-2	79.107	11.004
ct92-M305Pete-12	120362	13/01/2013	-2	79.107	11.003
ct92-M305Pete-12	120362	13/01/2013	-2	79.112	11.025
ct92-M305Pete-12	120362	13/01/2013	-2	79.136	10.904
ct92-M305Pete-12	120362	13/01/2013	-2	79.113	11.027
ct92-M305Pete-12	120362	13/01/2013	-2	79.118	11.004
ct92-M305Pete-12	120362	13/01/2013	-2	79.133	11.052
ct92-M305Pete-12	120362	13/01/2013	-2	79.128	11.047
ct92-M305Pete-12	120362	13/01/2013	-2	79.153	11.068
ct92-M305Pete-12	120362	13/01/2013	-2	79.153	11.119
ct92-M305Pete-12	120362	13/01/2013	-2	79.159	11.136
ct92-M305Pete-12	120362	13/01/2013	-2	79.249	11.111
ct92-M305Pete-12	120362	13/01/2013	-2	79.249	11.109
ct92-M305Pete-12	120362	13/01/2013	-2	79.16	10.947
ct92-M305Pete-12	120362	13/01/2013	-2	79.155	10.986
ct92-M305Pete-12	120362	13/01/2013	-2	79.248	11.015
ct92-M305Pete-12	120362	13/01/2013	-2	79.202	11.02
ct92-M305Pete-12	120362	13/01/2013	-2	79.259	10.834
ct92-M305Pete-12	120362	13/01/2013	-2	79.265	10.844
ct92-M305Pete-12	120362	13/01/2013	-2	79.278	10.865
ct92-M305Pete-12	120362	13/01/2013	-2	79.271	10.855
ct92-M305Pete-12	120362	13/01/2013	-2	79.251	10.905
ct92-M305Pete-12	120362	13/01/2013	-2	79.292	10.886
ct92-M305Pete-12	120362	13/01/2013	-2	79.28	10.944
ct92-M305Pete-12	120362	13/01/2013	-2	79.279	10.94
ct92-M305Pete-12	120362	13/01/2013	-2	79.324	11.077
ct92-M305Pete-12	120362	13/01/2013	-2	79.299	10.862
ct92-M305Pete-12	120362	13/01/2013	-2	79.298	10.881
ct92-M305Pete-12	120362	13/01/2013	-2	79.28	10.914
ct92-M305Pete-12	120362	13/01/2013	-2	79.278	10.951
ct92-M305Pete-12	120362	13/01/2013	-2	79.274	10.898
ct92-M305Pete-12	120362	13/01/2013	-2	79.295	10.85
ct92-M305Pete-12	120362	13/01/2013	-2	79.294	10.871
ct92-M305Pete-12	120362	13/01/2013	-2	79.262	10.914
ct92-M305Pete-12	120362	13/01/2013	-2	79.263	10.917
ct92-M305Pete-12	120362	13/01/2013	-2	79.267	10.939
ct92-M305Pete-12	120362	13/01/2013	-2	79.265	10.944
ct92-M305Pete-12	120362	13/01/2013	-2	79.264	10.937
ct92-M305Pete-12	120362	13/01/2013	-2	79.271	10.96
ct92-M305Pete-12	120362	13/01/2013	-2	79.257	10.899
ct92-M305Pete-12	120362	13/01/2013	-1	79.234	10.936
ct92-M305Pete-12	120362	13/01/2013	-1	79.246	10.908
ct92-M305Pete-12	120362	13/01/2013	0	79.242	10.892
ct92-M305Pete-12	120362	13/01/2013	-1	79.243	10.925
ct92-M305Pete-12	120362	13/01/2013	-2	79.226	10.95
ct92-M305Pete-12	120362	13/01/2013	-2	79.24	10.968
ct92-M305Pete-12	120362	13/01/2013	-2	79.238	10.968
ct92-M305Pete-12	120362	13/01/2013	-2	79.239	10.985
ct92-M305Pete-12	120362	13/01/2013	-2	79.235	11.001
ct92-M305Pete-12	120362	13/01/2013	-2	79.183	10.996
ct92-M305Pete-12	120362	13/01/2013	-2	79.234	10.998
ct92-M305Pete-12	120362	13/01/2013	-2	79.188	10.992
ct92-M305Pete-12	120362	13/01/2013	-2	79.189	11.008
ct92-M305Pete-12	120362	13/01/2013	-2	79.206	11.032
ct92-M305Pete-12	120362	13/01/2013	-2	79.205	11.007
ct92-M305Pete-12	120362	13/01/2013	-2	79.199	10.91

ct92-M305Pete-12	120362	13/01/2013	-2	79.201	11.071
ct92-M305Pete-12	120362	13/01/2013	-2	79.182	11.056
ct92-M305Pete-12	120362	13/01/2013	-1	79.174	11.088
ct92-M305Pete-12	120362	13/01/2013	-2	79.16	11.138
ct92-M305Pete-12	120362	13/01/2013	-2	79.16	11.134
ct92-M305Pete-12	120362	13/01/2013	-2	79.16	11.231
ct92-M305Pete-12	120362	13/01/2013	-2	79.163	11.123
ct92-M305Pete-12	120362	13/01/2013	-2	79.162	11.122
ct92-M305Pete-12	120362	13/01/2013	-2	79.156	10.938
ct92-M305Pete-12	120362	13/01/2013	-2	79.163	11.121
ct92-M305Pete-12	120362	13/01/2013	-2	79.158	10.948
ct92-M305Pete-12	120362	13/01/2013	-2	79.153	11.13
ct92-M305Pete-12	120362	13/01/2013	-2	79.154	11.092
ct92-M305Pete-12	120362	14/01/2013	-2	79.141	11.069
ct92-M305Pete-12	120362	14/01/2013	-2	79.138	11.114
ct92-M305Pete-12	120362	14/01/2013	-2	79.137	11.114
ct92-M305Pete-12	120362	14/01/2013	-2	79.151	11.058
ct92-M305Pete-12	120362	14/01/2013	-2	79.149	11.147
ct92-M305Pete-12	120362	14/01/2013	-1	79.125	11.07
ct92-M305Pete-12	120362	14/01/2013	-2	79.118	11.061
ct92-M305Pete-12	120362	14/01/2013	-2	79.119	11.06
ct92-M305Pete-12	120362	14/01/2013	-2	79.118	11.054
ct92-M305Pete-12	120362	14/01/2013	-2	79.115	11.122
ct92-M305Pete-12	120362	14/01/2013	-1	79.078	11.232
ct92-M305Pete-12	120362	14/01/2013	-2	79.093	10.973
ct92-M305Pete-12	120362	14/01/2013	-1	79.043	11.161
ct92-M305Pete-12	120362	14/01/2013	-2	79.065	11.067
ct92-M305Pete-12	120362	14/01/2013	-1	79.045	11.092
ct92-M305Pete-12	120362	14/01/2013	-2	79.03	11.151
ct92-M305Pete-12	120362	14/01/2013	-2	79.029	11.147
ct92-M305Pete-12	120362	14/01/2013	-2	79.027	11.136
ct92-M305Pete-12	120362	14/01/2013	-2	79.026	11.152
ct92-M305Pete-12	120362	14/01/2013	-2	79.014	11.153
ct92-M305Pete-12	120362	14/01/2013	-2	79.012	11.131
ct92-M305Pete-12	120362	14/01/2013	-2	79.019	11.066
ct92-M305Pete-12	120362	14/01/2013	-2	79.018	11.053
ct92-M305Pete-12	120362	14/01/2013	-2	78.978	11.038
ct92-M305Pete-12	120362	14/01/2013	-2	78.974	11.112
ct92-M305Pete-12	120362	14/01/2013	-2	78.946	11.02
ct92-M305Pete-12	120362	14/01/2013	-2	78.971	11.087
ct92-M305Pete-12	120362	14/01/2013	-2	78.968	10.997
ct92-M305Pete-12	120362	14/01/2013	-2	78.952	10.938
ct92-M305Pete-12	120362	14/01/2013	-2	78.929	11.074
ct92-M305Pete-12	120362	14/01/2013	-2	78.921	11.043
ct92-M305Pete-12	120362	14/01/2013	-2	78.89	10.94
ct92-M305Pete-12	120362	14/01/2013	-2	78.956	10.847
ct92-M305Pete-12	120362	14/01/2013	-2	78.884	10.906
ct92-M305Pete-12	120362	14/01/2013	-2	78.879	10.879
ct92-M305Pete-12	120362	14/01/2013	-2	78.884	10.9
ct92-M305Pete-12	120362	14/01/2013	-2	78.884	10.909
ct92-M305Pete-12	120362	14/01/2013	-1	78.902	10.805
ct92-M305Pete-12	120362	14/01/2013	-2	78.898	10.819
ct92-M305Pete-12	120362	14/01/2013	-1	78.91	10.75
ct92-M305Pete-12	120362	14/01/2013	1	78.9	10.78
ct92-M305Pete-12	120362	14/01/2013	-2	78.884	10.662
ct92-M305Pete-12	120362	14/01/2013	-2	78.891	10.648
ct92-M305Pete-12	120362	14/01/2013	-2	78.87	10.644
ct92-M305Pete-12	120362	14/01/2013	-1	78.894	10.618
ct92-M305Pete-12	120362	14/01/2013	-1	78.889	10.35
ct92-M305Pete-12	120362	14/01/2013	2	78.894	10.428
ct92-M305Pete-12	120362	14/01/2013	-2	78.893	10.442
ct92-M305Pete-12	120362	14/01/2013	-2	78.886	10.454
ct92-M305Pete-12	120362	14/01/2013	-2	78.871	10.446
ct92-M305Pete-12	120362	14/01/2013	-2	78.867	10.499
ct92-M305Pete-12	120362	14/01/2013	-2	78.864	10.388
ct92-M305Pete-12	120362	14/01/2013	-2	78.85	10.378
ct92-M305Pete-12	120362	14/01/2013	-2	78.832	10.482
ct92-M305Pete-12	120362	14/01/2013	-2	78.828	10.474

ct92-M305Pete-12	120362	14/01/2013	-2	78.813	10.415
ct92-M305Pete-12	120362	14/01/2013	-2	78.805	10.322
ct92-M305Pete-12	120362	14/01/2013	-2	78.807	10.291
ct92-M305Pete-12	120362	14/01/2013	-2	78.802	10.254
ct92-M305Pete-12	120362	14/01/2013	-2	78.754	10.325
ct92-M305Pete-12	120362	14/01/2013	-2	78.74	10.339
ct92-M305Pete-12	120362	14/01/2013	-2	78.747	10.413
ct92-M305Pete-12	120362	14/01/2013	-2	78.733	10.423
ct92-M305Pete-12	120362	14/01/2013	-2	78.713	10.411
ct92-M305Pete-12	120362	14/01/2013	-2	78.726	10.223
ct92-M305Pete-12	120362	14/01/2013	-2	78.675	10.447
ct92-M305Pete-12	120362	14/01/2013	-2	78.668	10.447
ct92-M305Pete-12	120362	14/01/2013	-2	78.648	10.616
ct92-M305Pete-12	120362	14/01/2013	-2	78.635	10.637
ct92-M305Pete-12	120362	14/01/2013	-2	78.652	10.636
ct92-M305Pete-12	120362	14/01/2013	-2	78.643	10.651
ct92-M305Pete-12	120362	14/01/2013	-2	78.666	10.53
ct92-M305Pete-12	120362	14/01/2013	-2	78.621	10.79
ct92-M305Pete-12	120362	15/01/2013	-2	78.59	10.858
ct92-M305Pete-12	120362	15/01/2013	-2	78.601	10.832
ct92-M305Pete-12	120362	15/01/2013	-2	78.591	10.856
ct92-M305Pete-12	120362	15/01/2013	-2	78.559	10.909
ct92-M305Pete-12	120362	15/01/2013	-2	78.549	10.92
ct92-M305Pete-12	120362	15/01/2013	-2	78.549	10.927
ct92-M305Pete-12	120362	15/01/2013	-2	78.546	10.93
ct92-M305Pete-12	120362	15/01/2013	-2	78.547	10.927
ct92-M305Pete-12	120362	15/01/2013	-2	78.52	10.968
ct92-M305Pete-12	120362	15/01/2013	-2	78.527	10.957
ct92-M305Pete-12	120362	15/01/2013	-2	78.514	10.992
ct92-M305Pete-12	120362	15/01/2013	-2	78.48	11.067
ct92-M305Pete-12	120362	15/01/2013	-1	78.515	10.962
ct92-M305Pete-12	120362	15/01/2013	-1	78.513	10.928
ct92-M305Pete-12	120362	15/01/2013	-1	78.489	10.969
ct92-M305Pete-12	120362	15/01/2013	-1	78.511	10.957
ct92-M305Pete-12	120362	15/01/2013	-2	78.493	10.965
ct92-M305Pete-12	120362	15/01/2013	-2	78.519	10.953
ct92-M305Pete-12	120362	15/01/2013	-2	78.508	10.962
ct92-M305Pete-12	120362	15/01/2013	-2	78.502	10.992
ct92-M305Pete-12	120362	15/01/2013	-1	78.512	10.957
ct92-M305Pete-12	120362	15/01/2013	-2	78.513	10.884
ct92-M305Pete-12	120362	15/01/2013	-2	78.456	10.831
ct92-M305Pete-12	120362	15/01/2013	-2	78.441	10.841
ct92-M305Pete-12	120362	15/01/2013	-2	78.463	10.941
ct92-M305Pete-12	120362	15/01/2013	-2	78.486	10.845
ct92-M305Pete-12	120362	15/01/2013	-2	78.51	11.07
ct92-M305Pete-12	120362	15/01/2013	-2	78.493	11.023
ct92-M305Pete-12	120362	15/01/2013	-2	78.493	11.015
ct92-M305Pete-12	120362	15/01/2013	-2	78.492	11.004
ct92-M305Pete-12	120362	15/01/2013	-2	78.48	10.985
ct92-M305Pete-12	120362	15/01/2013	-2	78.504	11.078
ct92-M305Pete-12	120362	15/01/2013	-2	78.513	11.095
ct92-M305Pete-12	120362	15/01/2013	-2	78.501	10.985
ct92-M305Pete-12	120362	15/01/2013	-2	78.513	11.031
ct92-M305Pete-12	120362	15/01/2013	-2	78.501	10.908
ct92-M305Pete-12	120362	15/01/2013	-2	78.477	10.944
ct92-M305Pete-12	120362	15/01/2013	-2	78.467	10.964
ct92-M305Pete-12	120362	15/01/2013	-2	78.465	10.965
ct92-M305Pete-12	120362	15/01/2013	-2	78.461	10.955
ct92-M305Pete-12	120362	15/01/2013	-2	78.461	10.954
ct92-M305Pete-12	120362	15/01/2013	-2	78.464	10.951
ct92-M305Pete-12	120362	15/01/2013	-2	78.465	11.09
ct92-M305Pete-12	120362	15/01/2013	-2	78.469	11.078
ct92-M305Pete-12	120362	15/01/2013	-2	78.471	11.033
ct92-M305Pete-12	120362	15/01/2013	-2	78.488	10.998
ct92-M305Pete-12	120362	15/01/2013	-2	78.489	11.048
ct92-M305Pete-12	120362	15/01/2013	-2	78.48	11.055
ct92-M305Pete-12	120362	15/01/2013	-1	78.497	11.123
ct92-M305Pete-12	120362	15/01/2013	-2	78.493	11.141

ct92-M305Pete-12	120362	15/01/2013	-1	78.486	10.908
ct92-M305Pete-12	120362	15/01/2013	-1	78.49	10.952
ct92-M305Pete-12	120362	15/01/2013	-2	78.504	11.022
ct92-M305Pete-12	120362	15/01/2013	-2	78.493	11.031
ct92-M305Pete-12	120362	15/01/2013	-2	78.503	10.892
ct92-M305Pete-12	120362	15/01/2013	-1	78.486	10.85
ct92-M305Pete-12	120362	15/01/2013	-2	78.5	10.893
ct92-M305Pete-12	120362	15/01/2013	-1	78.493	10.877
ct92-M305Pete-12	120362	15/01/2013	-2	78.492	10.867
ct92-M305Pete-12	120362	15/01/2013	-2	78.504	10.944
ct92-M305Pete-12	120362	15/01/2013	-1	78.524	10.987
ct92-M305Pete-12	120362	15/01/2013	-2	78.525	10.978
ct92-M305Pete-12	120362	15/01/2013	-1	78.54	10.883
ct92-M305Pete-12	120362	15/01/2013	-2	78.526	10.835
ct92-M305Pete-12	120362	15/01/2013	-2	78.54	10.883
ct92-M305Pete-12	120362	15/01/2013	-2	78.526	10.835
ct92-M305Pete-12	120362	15/01/2013	-1	78.528	10.836
ct92-M305Pete-12	120362	15/01/2013	-2	78.528	10.836
ct92-M305Pete-12	120362	15/01/2013	-2	78.55	10.891
ct92-M305Pete-12	120362	15/01/2013	-2	78.564	10.831
ct92-M305Pete-12	120362	15/01/2013	-2	78.564	10.827
ct92-M305Pete-12	120362	16/01/2013	-2	78.567	10.872
ct92-M305Pete-12	120362	16/01/2013	-2	78.586	10.883
ct92-M305Pete-12	120362	16/01/2013	-2	78.569	10.874
ct92-M305Pete-12	120362	16/01/2013	-2	78.576	10.874
ct92-M305Pete-12	120362	16/01/2013	-2	78.575	10.869
ct92-M305Pete-12	120362	16/01/2013	-2	78.64	10.781
ct92-M305Pete-12	120362	16/01/2013	-2	78.64	10.778
ct92-M305Pete-12	120362	16/01/2013	-2	78.651	10.797
ct92-M305Pete-12	120362	16/01/2013	-2	78.655	10.788
ct92-M305Pete-12	120362	16/01/2013	-2	78.657	10.78
ct92-M305Pete-12	120362	16/01/2013	-1	78.681	10.699
ct92-M305Pete-12	120362	16/01/2013	-1	78.651	10.737
ct92-M305Pete-12	120362	16/01/2013	-2	78.653	10.725
ct92-M305Pete-12	120362	16/01/2013	-2	78.665	10.675
ct92-M305Pete-12	120362	16/01/2013	-2	78.67	10.657
ct92-M305Pete-12	120362	16/01/2013	-2	78.657	10.712
ct92-M305Pete-12	120362	16/01/2013	-2	78.666	10.632
ct92-M305Pete-12	120362	16/01/2013	-2	78.662	10.702
ct92-M305Pete-12	120362	16/01/2013	-2	78.746	10.49
ct92-M305Pete-12	120362	16/01/2013	-2	78.75	10.417
ct92-M305Pete-12	120362	16/01/2013	-2	78.748	10.422
ct92-M305Pete-12	120362	16/01/2013	-2	78.76	10.478
ct92-M305Pete-12	120362	16/01/2013	-2	78.738	10.533
ct92-M305Pete-12	120362	16/01/2013	-2	78.745	10.531
ct92-M305Pete-12	120362	16/01/2013	-2	78.754	10.443
ct92-M305Pete-12	120362	16/01/2013	-2	78.76	10.457
ct92-M305Pete-12	120362	16/01/2013	-2	78.762	10.457
ct92-M305Pete-12	120362	16/01/2013	-1	78.789	10.35
ct92-M305Pete-12	120362	16/01/2013	-2	78.749	10.412
ct92-M305Pete-12	120362	16/01/2013	-2	78.757	10.506
ct92-M305Pete-12	120362	16/01/2013	-2	78.779	10.396
ct92-M305Pete-12	120362	16/01/2013	-2	78.778	10.394
ct92-M305Pete-12	120362	16/01/2013	-2	78.775	10.373
ct92-M305Pete-12	120362	16/01/2013	-2	78.777	10.395
ct92-M305Pete-12	120362	16/01/2013	-2	78.791	10.437
ct92-M305Pete-12	120362	16/01/2013	-2	78.793	10.403
ct92-M305Pete-12	120362	16/01/2013	-2	78.795	10.407
ct92-M305Pete-12	120362	16/01/2013	-2	78.794	10.405
ct92-M305Pete-12	120362	16/01/2013	-2	78.8	10.397
ct92-M305Pete-12	120362	16/01/2013	-2	78.798	10.393
ct92-M305Pete-12	120362	16/01/2013	-2	78.805	10.405
ct92-M305Pete-12	120362	16/01/2013	-2	78.815	10.415
ct92-M305Pete-12	120362	16/01/2013	-2	78.834	10.364
ct92-M305Pete-12	120362	16/01/2013	-2	78.82	10.389
ct92-M305Pete-12	120362	16/01/2013	-2	78.829	10.379
ct92-M305Pete-12	120362	16/01/2013	-1	78.828	10.314
ct92-M305Pete-12	120362	16/01/2013	-2	78.832	10.325

ct92-M305Pete-12	120362	16/01/2013	-2	78.852	10.363
ct92-M305Pete-12	120362	16/01/2013	-2	78.838	10.323
ct92-M305Pete-12	120362	16/01/2013	-2	78.837	10.327
ct92-M305Pete-12	120362	16/01/2013	-1	78.822	10.356
ct92-M305Pete-12	120362	16/01/2013	-2	78.872	10.4
ct92-M305Pete-12	120362	16/01/2013	-2	78.869	10.391
ct92-M305Pete-12	120362	16/01/2013	-2	78.88	10.274
ct92-M305Pete-12	120362	16/01/2013	-1	78.872	10.378
ct92-M305Pete-12	120362	16/01/2013	-2	78.887	10.34
ct92-M305Pete-12	120362	16/01/2013	-2	78.889	10.337
ct92-M305Pete-12	120362	16/01/2013	-2	78.89	10.344
ct92-M305Pete-12	120362	16/01/2013	-2	78.887	10.403
ct92-M305Pete-12	120362	16/01/2013	-2	78.883	10.323
ct92-M305Pete-12	120362	16/01/2013	-2	78.894	10.335
ct92-M305Pete-12	120362	16/01/2013	-2	78.895	10.147
ct92-M305Pete-12	120362	16/01/2013	-2	78.895	10.148
ct92-M305Pete-12	120362	16/01/2013	-2	78.942	10.305
ct92-M305Pete-12	120362	16/01/2013	-2	78.909	10.036
ct92-M305Pete-12	120362	17/01/2013	-2	78.96	10.333
ct92-M305Pete-12	120362	17/01/2013	-2	78.947	10.388
ct92-M305Pete-12	120362	17/01/2013	-2	78.942	10.41
ct92-M305Pete-12	120362	17/01/2013	-2	78.944	10.41
ct92-M305Pete-12	120362	17/01/2013	-2	78.911	10.545
ct92-M305Pete-12	120362	17/01/2013	-2	78.951	10.411
ct92-M305Pete-12	120362	17/01/2013	-2	78.964	10.407
ct92-M305Pete-12	120362	17/01/2013	-2	78.955	10.429
ct92-M305Pete-12	120362	17/01/2013	-1	78.933	10.442
ct92-M305Pete-12	120362	17/01/2013	-2	78.933	10.444
ct92-M305Pete-12	120362	17/01/2013	-2	78.929	10.533
ct92-M305Pete-12	120362	17/01/2013	-2	78.958	10.501
ct92-M305Pete-12	120362	17/01/2013	-2	78.932	10.588
ct92-M305Pete-12	120362	17/01/2013	-2	78.934	10.59
ct92-M305Pete-12	120362	17/01/2013	-2	78.935	10.597
ct92-M305Pete-12	120362	17/01/2013	-2	79.006	10.775
ct92-M305Pete-12	120362	17/01/2013	-2	78.98	10.728
ct92-M305Pete-12	120362	17/01/2013	-2	78.977	10.732
ct92-M305Pete-12	120362	17/01/2013	-2	78.938	10.57
ct92-M305Pete-12	120362	17/01/2013	-1	78.945	10.658
ct92-M305Pete-12	120362	17/01/2013	-2	78.923	10.424
ct92-M305Pete-12	120362	17/01/2013	-2	78.927	10.521
ct92-M305Pete-12	120362	17/01/2013	-2	78.94	10.527
ct92-M305Pete-12	120362	17/01/2013	-2	78.937	10.519
ct92-M305Pete-12	120362	17/01/2013	-2	78.942	10.524
ct92-M305Pete-12	120362	17/01/2013	-2	78.945	10.531
ct92-M305Pete-12	120362	17/01/2013	-1	78.978	10.414
ct92-M305Pete-12	120362	17/01/2013	-1	78.961	10.454
ct92-M305Pete-12	120362	17/01/2013	-2	78.939	10.49
ct92-M305Pete-12	120362	17/01/2013	-2	78.939	10.458
ct92-M305Pete-12	120362	17/01/2013	-2	78.941	10.45
ct92-M305Pete-12	120362	17/01/2013	-2	78.944	10.446
ct92-M305Pete-12	120362	17/01/2013	-2	78.961	10.537
ct92-M305Pete-12	120362	17/01/2013	-2	78.958	10.529
ct92-M305Pete-12	120362	17/01/2013	-2	78.956	10.508
ct92-M305Pete-12	120362	17/01/2013	-2	78.963	10.489
ct92-M305Pete-12	120362	17/01/2013	-2	78.958	10.511
ct92-M305Pete-12	120362	17/01/2013	-2	78.969	10.49
ct92-M305Pete-12	120362	17/01/2013	-2	78.961	10.534
ct92-M305Pete-12	120362	17/01/2013	-2	78.956	10.506
ct92-M305Pete-12	120362	17/01/2013	-2	78.956	10.437
ct92-M305Pete-12	120362	17/01/2013	-1	78.945	10.451
ct92-M305Pete-12	120362	17/01/2013	-2	78.939	10.513
ct92-M305Pete-12	120362	17/01/2013	-2	78.941	10.528
ct92-M305Pete-12	120362	17/01/2013	-1	78.942	10.543
ct92-M305Pete-12	120362	17/01/2013	-1	78.932	10.497
ct92-M305Pete-12	120362	17/01/2013	-2	78.952	10.428
ct92-M305Pete-12	120362	17/01/2013	-2	78.935	10.511
ct92-M305Pete-12	120362	17/01/2013	-2	78.937	10.509
ct92-M305Pete-12	120362	17/01/2013	-2	78.966	10.444

ct92-M305Pete-12	120362	17/01/2013	-2	78.941	10.51
ct92-M305Pete-12	120362	17/01/2013	-2	78.942	10.513
ct92-M305Pete-12	120362	17/01/2013	-2	78.959	10.519
ct92-M305Pete-12	120362	17/01/2013	-2	78.934	10.509
ct92-M305Pete-12	120362	17/01/2013	-2	78.95	10.527
ct92-M305Pete-12	120362	17/01/2013	-2	78.948	10.532
ct92-M305Pete-12	120362	18/01/2013	2	78.91	10.778
ct92-M305Pete-12	120362	18/01/2013	1	78.921	10.778
ct92-M305Pete-12	120362	18/01/2013	-2	78.944	10.721
ct92-M305Pete-12	120362	18/01/2013	-2	78.938	10.75
ct92-M305Pete-12	120362	18/01/2013	-2	78.934	10.757
ct92-M305Pete-12	120362	18/01/2013	-2	78.935	10.746
ct92-M305Pete-12	120362	18/01/2013	-1	78.927	10.645
ct92-M305Pete-12	120362	18/01/2013	-2	78.937	10.733
ct92-M305Pete-12	120362	18/01/2013	-2	78.94	10.73
ct92-M305Pete-12	120362	18/01/2013	-2	78.946	10.677
ct92-M305Pete-12	120362	18/01/2013	-2	78.942	10.651
ct92-M305Pete-12	120362	18/01/2013	-1	78.929	10.658
ct92-M305Pete-12	120362	18/01/2013	-2	78.944	10.668
ct92-M305Pete-12	120362	18/01/2013	-2	78.946	10.624
ct92-M305Pete-12	120362	18/01/2013	-2	78.946	10.652
ct92-M305Pete-12	120362	18/01/2013	-2	78.941	10.641
ct92-M305Pete-12	120362	18/01/2013	-1	78.944	10.608
ct92-M305Pete-12	120362	18/01/2013	-2	78.944	10.604
ct92-M305Pete-12	120362	18/01/2013	-2	78.947	10.595
ct92-M305Pete-12	120362	18/01/2013	-2	78.947	10.587
ct92-M305Pete-12	120362	18/01/2013	-2	78.939	10.59
ct92-M305Pete-12	120362	18/01/2013	-2	78.949	10.561
ct92-M305Pete-12	120362	18/01/2013	-2	78.954	10.557
ct92-M305Pete-12	120362	18/01/2013	-1	78.96	10.542
ct92-M305Pete-12	120362	18/01/2013	-2	78.963	10.531
ct92-M305Pete-12	120362	18/01/2013	-2	78.945	10.577
ct92-M305Pete-12	120362	18/01/2013	-2	78.965	10.563
ct92-M305Pete-12	120362	18/01/2013	-2	78.956	10.475
ct92-M305Pete-12	120362	18/01/2013	-1	78.949	10.573
ct92-M305Pete-12	120362	18/01/2013	-2	78.935	10.599
ct92-M305Pete-12	120362	18/01/2013	-1	78.941	10.568
ct92-M305Pete-12	120362	18/01/2013	-1	78.937	10.532
ct92-M305Pete-12	120362	18/01/2013	-2	78.933	10.537
ct92-M305Pete-12	120362	18/01/2013	-2	78.928	10.539
ct92-M305Pete-12	120362	18/01/2013	-2	78.953	10.643
ct92-M305Pete-12	120362	18/01/2013	-2	78.932	10.535
ct92-M305Pete-12	120362	18/01/2013	-2	78.932	10.527
ct92-M305Pete-12	120362	18/01/2013	-1	78.927	10.578
ct92-M305Pete-12	120362	18/01/2013	-2	78.926	10.578
ct92-M305Pete-12	120362	18/01/2013	-2	78.926	10.579
ct92-M305Pete-12	120362	18/01/2013	-2	78.933	10.566
ct92-M305Pete-12	120362	18/01/2013	-2	78.933	10.566
ct92-M305Pete-12	120362	18/01/2013	-1	78.931	10.548
ct92-M305Pete-12	120362	18/01/2013	-2	78.921	10.58
ct92-M305Pete-12	120362	19/01/2013	-2	78.916	10.609
ct92-M305Pete-12	120362	19/01/2013	-2	78.911	10.598
ct92-M305Pete-12	120362	19/01/2013	-2	78.91	10.589
ct92-M305Pete-12	120362	19/01/2013	-2	78.904	10.56
ct92-M305Pete-12	120362	19/01/2013	0	78.904	10.376
ct92-M305Pete-12	120362	19/01/2013	-2	78.889	10.406
ct92-M305Pete-12	120362	19/01/2013	-1	78.89	10.722
ct92-M305Pete-12	120362	19/01/2013	-1	78.884	10.73
ct92-M305Pete-12	120362	19/01/2013	-1	78.879	10.731
ct92-M305Pete-12	120362	19/01/2013	-2	78.883	10.72
ct92-M305Pete-12	120362	19/01/2013	-2	78.885	10.753
ct92-M305Pete-12	120362	19/01/2013	-2	78.889	10.743
ct92-M305Pete-12	120362	19/01/2013	-1	78.893	10.801
ct92-M305Pete-12	120362	19/01/2013	-2	78.888	10.755
ct92-M305Pete-12	120362	19/01/2013	-2	78.88	10.844
ct92-M305Pete-12	120362	19/01/2013	2	78.878	10.827
ct92-M305Pete-12	120362	19/01/2013	-2	78.876	10.833
ct92-M305Pete-12	120362	19/01/2013	-2	78.866	10.859

ct92-M305Pete-12	120362	19/01/2013	-2	78.868	10.845
ct92-M305Pete-12	120362	19/01/2013	-2	78.898	10.777
ct92-M305Pete-12	120362	19/01/2013	-2	78.891	10.802
ct92-M305Pete-12	120362	19/01/2013	-2	78.922	10.661
ct92-M305Pete-12	120362	19/01/2013	-2	78.908	10.588
ct92-M305Pete-12	120362	19/01/2013	0	78.867	10.659
ct92-M305Pete-12	120362	19/01/2013	-1	78.911	10.547
ct92-M305Pete-12	120362	19/01/2013	-2	78.887	10.602
ct92-M305Pete-12	120362	19/01/2013	-2	78.887	10.571
ct92-M305Pete-12	120362	19/01/2013	-1	78.898	10.619
ct92-M305Pete-12	120362	19/01/2013	-1	78.911	10.448
ct92-M305Pete-12	120362	19/01/2013	-2	78.914	10.476
ct92-M305Pete-12	120362	19/01/2013	-2	78.915	10.624
ct92-M305Pete-12	120362	19/01/2013	-2	78.906	10.656
ct92-M305Pete-12	120362	19/01/2013	-2	78.908	10.639
ct92-M305Pete-12	120362	19/01/2013	-1	78.946	10.437
ct92-M305Pete-12	120362	19/01/2013	-1	78.942	10.438
ct92-M305Pete-12	120362	19/01/2013	-2	78.947	10.425
ct92-M305Pete-12	120362	19/01/2013	-2	78.965	10.542
ct92-M305Pete-12	120362	19/01/2013	-2	78.983	10.517
ct92-M305Pete-12	120362	19/01/2013	-2	78.957	10.421
ct92-M305Pete-12	120362	19/01/2013	-2	78.954	10.438
ct92-M305Pete-12	120362	19/01/2013	-2	78.952	10.382
ct92-M305Pete-12	120362	19/01/2013	-2	78.964	10.495
ct92-M305Pete-12	120362	19/01/2013	-2	78.964	10.35
ct92-M305Pete-12	120362	19/01/2013	1	78.949	10.569
ct92-M305Pete-12	120362	19/01/2013	-1	78.952	10.526
ct92-M305Pete-12	120362	19/01/2013	1	78.94	10.516
ct92-M305Pete-12	120362	19/01/2013	2	78.945	10.535
ct92-M305Pete-12	120362	19/01/2013	2	78.945	10.534
ct92-M305Pete-12	120362	19/01/2013	-2	78.947	10.504
ct92-M305Pete-12	120362	19/01/2013	-2	78.945	10.53
ct92-M305Pete-12	120362	19/01/2013	-2	78.957	10.578
ct92-M305Pete-12	120362	19/01/2013	0	78.943	10.582
ct92-M305Pete-12	120362	19/01/2013	-1	78.942	10.577
ct92-M305Pete-12	120362	19/01/2013	-1	78.944	10.629
ct92-M305Pete-12	120362	19/01/2013	-2	78.945	10.578
ct92-M305Pete-12	120362	19/01/2013	1	78.944	10.575
ct92-M305Pete-12	120362	19/01/2013	-1	78.941	10.524
ct92-M305Pete-12	120362	19/01/2013	-1	78.944	10.508
ct92-M305Pete-12	120362	19/01/2013	-2	78.941	10.501
ct92-M305Pete-12	120362	19/01/2013	-2	78.95	10.573
ct92-M305Pete-12	120362	19/01/2013	-2	78.938	10.564
ct92-M305Pete-12	120362	19/01/2013	-2	78.938	10.542
ct92-M305Pete-12	120362	19/01/2013	-2	78.94	10.561
ct92-M305Pete-12	120362	19/01/2013	-2	78.936	10.553
ct92-M305Pete-12	120362	19/01/2013	-2	78.939	10.564
ct92-M305Pete-12	120362	19/01/2013	-2	78.936	10.534
ct92-M305Pete-12	120362	19/01/2013	-2	78.935	10.518
ct92-M305Pete-12	120362	19/01/2013	-2	78.939	10.566
ct92-M305Pete-12	120362	19/01/2013	0	78.95	10.573
ct92-M305Pete-12	120362	19/01/2013	-2	78.941	10.561
ct92-M305Pete-12	120362	19/01/2013	-2	78.951	10.573
ct92-M305Pete-12	120362	19/01/2013	-2	78.941	10.557
ct92-M305Pete-12	120362	19/01/2013	-2	78.941	10.56
ct92-M305Pete-12	120362	19/01/2013	-2	78.945	10.463
ct92-M305Pete-12	120362	19/01/2013	-2	78.946	10.465
ct92-M305Pete-12	120362	19/01/2013	-1	78.943	10.613
ct92-M305Pete-12	120362	19/01/2013	-1	78.942	10.569
ct92-M305Pete-12	120362	20/01/2013	-2	78.944	10.602
ct92-M305Pete-12	120362	20/01/2013	-2	78.95	10.583
ct92-M305Pete-12	120362	20/01/2013	-2	78.943	10.606
ct92-M305Pete-12	120362	20/01/2013	-2	78.942	10.603
ct92-M305Pete-12	120362	20/01/2013	-2	78.94	10.605
ct92-M305Pete-12	120362	20/01/2013	-2	78.926	10.71
ct92-M305Pete-12	120362	20/01/2013	-2	78.919	10.664
ct92-M305Pete-12	120362	20/01/2013	-1	78.936	10.459
ct92-M305Pete-12	120362	20/01/2013	-2	78.935	10.564

ct92-M305Pete-12	120362	20/01/2013	-1	78.938	10.576
ct92-M305Pete-12	120362	20/01/2013	-2	78.928	10.551
ct92-M305Pete-12	120362	20/01/2013	-2	78.928	10.509
ct92-M305Pete-12	120362	20/01/2013	-2	78.957	10.472
ct92-M305Pete-12	120362	20/01/2013	-2	78.954	10.458
ct92-M305Pete-12	120362	20/01/2013	-2	78.961	10.473
ct92-M305Pete-12	120362	20/01/2013	-2	78.951	10.535
ct92-M305Pete-12	120362	20/01/2013	-2	78.927	10.646
ct92-M305Pete-12	120362	20/01/2013	-2	78.935	10.625
ct92-M305Pete-12	120362	20/01/2013	-2	78.928	10.618
ct92-M305Pete-12	120362	20/01/2013	-2	78.931	10.612
ct92-M305Pete-12	120362	20/01/2013	-2	78.93	10.616
ct92-M305Pete-12	120362	20/01/2013	-1	78.931	10.59
ct92-M305Pete-12	120362	20/01/2013	-2	78.93	10.592
ct92-M305Pete-12	120362	20/01/2013	-1	78.931	10.632
ct92-M305Pete-12	120362	20/01/2013	-2	78.929	10.576
ct92-M305Pete-12	120362	20/01/2013	-2	78.927	10.599
ct92-M305Pete-12	120362	20/01/2013	-2	78.943	10.651
ct92-M305Pete-12	120362	20/01/2013	-2	78.945	10.574
ct92-M305Pete-12	120362	20/01/2013	-2	78.912	10.649
ct92-M305Pete-12	120362	20/01/2013	-2	78.908	10.677
ct92-M305Pete-12	120362	20/01/2013	-2	78.912	10.688
ct92-M305Pete-12	120362	20/01/2013	-2	78.916	10.707
ct92-M305Pete-12	120362	20/01/2013	-1	78.932	10.693
ct92-M305Pete-12	120362	20/01/2013	-2	78.916	10.778
ct92-M305Pete-12	120362	20/01/2013	-2	78.934	10.681
ct92-M305Pete-12	120362	20/01/2013	-2	78.92	10.667
ct92-M305Pete-12	120362	20/01/2013	-2	78.928	10.778
ct92-M305Pete-12	120362	20/01/2013	-2	78.928	10.779
ct92-M305Pete-12	120362	20/01/2013	-2	78.926	10.764
ct92-M305Pete-12	120362	20/01/2013	-2	78.927	10.716
ct92-M305Pete-12	120362	20/01/2013	0	78.972	10.728
ct92-M305Pete-12	120362	20/01/2013	-1	78.91	10.62
ct92-M305Pete-12	120362	20/01/2013	0	78.956	10.853
ct92-M305Pete-12	120362	20/01/2013	-2	78.956	10.853
ct92-M305Pete-12	120362	20/01/2013	-2	78.924	10.718
ct92-M305Pete-12	120362	20/01/2013	-2	78.964	10.878
ct92-M305Pete-12	120362	20/01/2013	-2	78.928	10.73
ct92-M305Pete-12	120362	20/01/2013	-2	78.94	10.644
ct92-M305Pete-12	120362	20/01/2013	-1	78.942	10.647
ct92-M305Pete-12	120362	20/01/2013	-2	78.918	10.729
ct92-M305Pete-12	120362	20/01/2013	-2	78.918	10.731
ct92-M305Pete-12	120362	20/01/2013	-2	78.948	10.56
ct92-M305Pete-12	120362	20/01/2013	-2	78.93	10.67
ct92-M305Pete-12	120362	20/01/2013	-2	78.933	10.691
ct92-M305Pete-12	120362	20/01/2013	-2	78.948	10.536
ct92-M305Pete-12	120362	20/01/2013	-2	78.925	10.436
ct92-M305Pete-12	120362	20/01/2013	-2	78.924	10.444
ct92-M305Pete-12	120362	20/01/2013	-2	78.933	10.581
ct92-M305Pete-12	120362	20/01/2013	-2	78.932	10.585
ct92-M305Pete-12	120362	20/01/2013	-2	78.933	10.578
ct92-M305Pete-12	120362	20/01/2013	-2	78.915	10.588
ct92-M305Pete-12	120362	20/01/2013	-1	78.91	10.615
ct92-M305Pete-12	120362	20/01/2013	-2	78.901	10.553
ct92-M305Pete-12	120362	20/01/2013	-2	78.906	10.63
ct92-M305Pete-12	120362	20/01/2013	-2	78.914	10.527
ct92-M305Pete-12	120362	20/01/2013	-2	78.913	10.514
ct92-M305Pete-12	120362	20/01/2013	-2	78.914	10.507
ct92-M305Pete-12	120362	20/01/2013	-2	78.914	10.526
ct92-M305Pete-12	120362	20/01/2013	-2	78.929	10.496
ct92-M305Pete-12	120362	21/01/2013	-2	78.936	10.459
ct92-M305Pete-12	120362	21/01/2013	-2	78.936	10.46
ct92-M305Pete-12	120362	21/01/2013	-2	78.95	10.48
ct92-M305Pete-12	120362	21/01/2013	-2	78.947	10.513
ct92-M305Pete-12	120362	21/01/2013	-2	78.947	10.521
ct92-M305Pete-12	120362	21/01/2013	-1	78.948	10.486
ct92-M305Pete-12	120362	21/01/2013	1	78.935	10.524
ct92-M305Pete-12	120362	21/01/2013	-2	78.948	10.506

ct92-M305Pete-12	120362	21/01/2013	-2	78.956	10.529
ct92-M305Pete-12	120362	21/01/2013	-2	78.948	10.576
ct92-M305Pete-12	120362	21/01/2013	-1	78.949	10.51
ct92-M305Pete-12	120362	21/01/2013	-2	78.953	10.57
ct92-M305Pete-12	120362	21/01/2013	-2	78.949	10.565
ct92-M305Pete-12	120362	21/01/2013	-2	78.947	10.562
ct92-M305Pete-12	120362	21/01/2013	-2	78.945	10.576
ct92-M305Pete-12	120362	21/01/2013	2	78.934	10.559
ct92-M305Pete-12	120362	21/01/2013	-2	78.934	10.563
ct92-M305Pete-12	120362	21/01/2013	-2	78.934	10.562
ct92-M305Pete-12	120362	21/01/2013	-1	78.935	10.558
ct92-M305Pete-12	120362	21/01/2013	-2	78.94	10.562
ct92-M305Pete-12	120362	21/01/2013	-2	78.947	10.55
ct92-M305Pete-12	120362	21/01/2013	-2	78.948	10.543
ct92-M305Pete-12	120362	21/01/2013	-2	78.948	10.544
ct92-M305Pete-12	120362	21/01/2013	-2	78.948	10.551
ct92-M305Pete-12	120362	21/01/2013	-2	78.949	10.547
ct92-M305Pete-12	120362	21/01/2013	-2	78.938	10.546
ct92-M305Pete-12	120362	21/01/2013	-1	78.954	10.51
ct92-M305Pete-12	120362	21/01/2013	-2	78.944	10.522
ct92-M305Pete-12	120362	21/01/2013	1	78.946	10.525
ct92-M305Pete-12	120362	21/01/2013	-2	78.952	10.539
ct92-M305Pete-12	120362	21/01/2013	-1	78.946	10.57
ct92-M305Pete-12	120362	21/01/2013	-2	78.951	10.619
ct92-M305Pete-12	120362	21/01/2013	-2	78.928	10.586
ct92-M305Pete-12	120362	21/01/2013	-1	78.914	10.562
ct92-M305Pete-12	120362	21/01/2013	-1	78.905	10.612
ct92-M305Pete-12	120362	21/01/2013	-1	78.912	10.617
ct92-M305Pete-12	120362	21/01/2013	-1	78.905	10.614
ct92-M305Pete-12	120362	21/01/2013	0	78.926	10.784
ct92-M305Pete-12	120362	21/01/2013	-2	78.911	10.729
ct92-M305Pete-12	120362	21/01/2013	-1	78.893	10.656
ct92-M305Pete-12	120362	21/01/2013	-1	78.904	10.657
ct92-M305Pete-12	120362	21/01/2013	1	78.925	10.632
ct92-M305Pete-12	120362	21/01/2013	-2	78.923	10.627
ct92-M305Pete-12	120362	21/01/2013	-2	78.921	10.624
ct92-M305Pete-12	120362	21/01/2013	-1	78.914	10.613
ct92-M305Pete-12	120362	21/01/2013	-1	78.903	10.634
ct92-M305Pete-12	120362	21/01/2013	-1	78.903	10.635
ct92-M305Pete-12	120362	21/01/2013	1	78.894	10.607
ct92-M305Pete-12	120362	21/01/2013	-2	78.894	10.603
ct92-M305Pete-12	120362	21/01/2013	0	78.898	10.664
ct92-M305Pete-12	120362	21/01/2013	-2	78.892	10.652
ct92-M305Pete-12	120362	21/01/2013	-1	78.899	10.658
ct92-M305Pete-12	120362	21/01/2013	-2	78.899	10.66
ct92-M305Pete-12	120362	21/01/2013	-2	78.899	10.66
ct92-M305Pete-12	120362	21/01/2013	-1	78.89	10.57
ct92-M305Pete-12	120362	21/01/2013	1	78.892	10.503
ct92-M305Pete-12	120362	21/01/2013	1	78.893	10.505
ct92-M305Pete-12	120362	21/01/2013	0	78.895	10.493
ct92-M305Pete-12	120362	21/01/2013	1	78.907	10.497
ct92-M305Pete-12	120362	21/01/2013	1	78.9	10.471
ct92-M305Pete-12	120362	21/01/2013	-2	78.9	10.486
ct92-M305Pete-12	120362	21/01/2013	-2	78.899	10.481
ct92-M305Pete-12	120362	21/01/2013	-2	78.886	10.465
ct92-M305Pete-12	120362	21/01/2013	-1	78.896	10.461
ct92-M305Pete-12	120362	21/01/2013	1	78.905	10.453
ct92-M305Pete-12	120362	21/01/2013	-2	78.905	10.45
ct92-M305Pete-12	120362	21/01/2013	-2	78.905	10.45
ct92-M305Pete-12	120362	21/01/2013	2	78.914	10.382
ct92-M305Pete-12	120362	21/01/2013	-2	78.906	10.291
ct92-M305Pete-12	120362	21/01/2013	-2	78.909	10.415
ct92-M305Pete-12	120362	22/01/2013	-2	78.917	10.432
ct92-M305Pete-12	120362	22/01/2013	-2	78.908	10.445
ct92-M305Pete-12	120362	22/01/2013	-2	78.909	10.446
ct92-M305Pete-12	120362	22/01/2013	-2	78.907	10.497
ct92-M305Pete-12	120362	22/01/2013	-2	78.928	10.659
ct92-M305Pete-12	120362	22/01/2013	-2	78.906	10.493

ct92-M305Pete-12	120362	22/01/2013	-2	78.907	10.496
ct92-M305Pete-12	120362	22/01/2013	-2	78.91	10.501
ct92-M305Pete-12	120362	22/01/2013	-2	78.91	10.5
ct92-M305Pete-12	120362	22/01/2013	-2	78.917	10.478
ct92-M305Pete-12	120362	22/01/2013	-2	78.916	10.525
ct92-M305Pete-12	120362	22/01/2013	-2	78.922	10.534
ct92-M305Pete-12	120362	22/01/2013	-2	78.92	10.544
ct92-M305Pete-12	120362	22/01/2013	-2	78.921	10.544
ct92-M305Pete-12	120362	22/01/2013	-2	78.926	10.545
ct92-M305Pete-12	120362	22/01/2013	-2	78.922	10.612
ct92-M305Pete-12	120362	22/01/2013	-1	78.904	10.662
ct92-M305Pete-12	120362	22/01/2013	-2	78.919	10.595
ct92-M305Pete-12	120362	22/01/2013	-2	78.917	10.608
ct92-M305Pete-12	120362	22/01/2013	-2	78.923	10.633
ct92-M305Pete-12	120362	22/01/2013	-2	78.92	10.61
ct92-M305Pete-12	120362	22/01/2013	-2	78.937	10.54
ct92-M305Pete-12	120362	22/01/2013	-2	78.942	10.521
ct92-M305Pete-12	120362	22/01/2013	0	78.948	10.626
ct92-M305Pete-12	120362	22/01/2013	-2	78.938	10.683
ct92-M305Pete-12	120362	22/01/2013	-2	78.937	10.689
ct92-M305Pete-12	120362	22/01/2013	-2	78.937	10.685
ct92-M305Pete-12	120362	22/01/2013	-2	78.938	10.664
ct92-M305Pete-12	120362	22/01/2013	-2	78.933	10.617
ct92-M305Pete-12	120362	22/01/2013	-2	78.934	10.626
ct92-M305Pete-12	120362	22/01/2013	-2	78.93	10.655
ct92-M305Pete-12	120362	22/01/2013	-1	78.95	10.647
ct92-M305Pete-12	120362	22/01/2013	-2	78.947	10.628
ct92-M305Pete-12	120362	22/01/2013	-2	78.931	10.698
ct92-M305Pete-12	120362	22/01/2013	-2	78.919	10.698
ct92-M305Pete-12	120362	22/01/2013	-2	78.922	10.717
ct92-M305Pete-12	120362	22/01/2013	-2	78.919	10.715
ct92-M305Pete-12	120362	22/01/2013	-2	78.927	10.731
ct92-M305Pete-12	120362	22/01/2013	-2	78.925	10.743
ct92-M305Pete-12	120362	22/01/2013	-2	78.932	10.654
ct92-M305Pete-12	120362	22/01/2013	-2	78.914	10.631
ct92-M305Pete-12	120362	22/01/2013	-2	78.92	10.65
ct92-M305Pete-12	120362	22/01/2013	-2	78.924	10.652
ct92-M305Pete-12	120362	22/01/2013	-2	78.919	10.659
ct92-M305Pete-12	120362	22/01/2013	-1	78.91	10.634
ct92-M305Pete-12	120362	22/01/2013	-2	78.909	10.612
ct92-M305Pete-12	120362	22/01/2013	-2	78.908	10.612
ct92-M305Pete-12	120362	22/01/2013	-2	78.911	10.583
ct92-M305Pete-12	120362	22/01/2013	-2	78.916	10.66
ct92-M305Pete-12	120362	22/01/2013	-1	78.917	10.646
ct92-M305Pete-12	120362	22/01/2013	-2	78.917	10.659
ct92-M305Pete-12	120362	22/01/2013	-2	78.918	10.649
ct92-M305Pete-12	120362	22/01/2013	-2	78.905	10.595
ct92-M305Pete-12	120362	22/01/2013	-2	78.89	10.657
ct92-M305Pete-12	120362	22/01/2013	-2	78.911	10.57
ct92-M305Pete-12	120362	22/01/2013	-2	78.895	10.661
ct92-M305Pete-12	120362	22/01/2013	-2	78.902	10.619
ct92-M305Pete-12	120362	22/01/2013	-2	78.904	10.649
ct92-M305Pete-12	120362	22/01/2013	-2	78.904	10.686
ct92-M305Pete-12	120362	22/01/2013	-2	78.904	10.69
ct92-M305Pete-12	120362	22/01/2013	-2	78.91	10.653
ct92-M305Pete-12	120362	22/01/2013	-1	78.906	10.654
ct92-M305Pete-12	120362	22/01/2013	-2	78.898	10.771
ct92-M305Pete-12	120362	22/01/2013	-2	78.889	10.728
ct92-M305Pete-12	120362	22/01/2013	-2	78.895	10.722
ct92-M305Pete-12	120362	22/01/2013	-2	78.903	10.606
ct92-M305Pete-12	120362	22/01/2013	-2	78.912	10.566
ct92-M305Pete-12	120362	22/01/2013	-2	78.894	10.698
ct92-M305Pete-12	120362	22/01/2013	-2	78.895	10.699
ct92-M305Pete-12	120362	22/01/2013	-2	78.9	10.632
ct92-M305Pete-12	120362	22/01/2013	-2	78.9	10.631
ct92-M305Pete-12	120362	22/01/2013	-2	78.869	10.619
ct92-M305Pete-12	120362	22/01/2013	-2	78.869	10.619
ct92-M305Pete-12	120362	22/01/2013	-2	78.886	10.66

ct92-M305Pete-12	120362	23/01/2013	-2	78.887	10.654
ct92-M305Pete-12	120362	23/01/2013	-2	78.884	10.688
ct92-M305Pete-12	120362	23/01/2013	-2	78.898	10.667
ct92-M305Pete-12	120362	23/01/2013	-2	78.895	10.674
ct92-M305Pete-12	120362	23/01/2013	2	78.898	10.62
ct92-M305Pete-12	120362	23/01/2013	-2	78.913	10.64
ct92-M305Pete-12	120362	23/01/2013	-2	78.913	10.643
ct92-M305Pete-12	120362	23/01/2013	-2	78.913	10.638
ct92-M305Pete-12	120362	23/01/2013	-2	78.912	10.647
ct92-M305Pete-12	120362	23/01/2013	-2	78.91	10.634
ct92-M305Pete-12	120362	23/01/2013	-2	78.928	10.522
ct92-M305Pete-12	120362	23/01/2013	-2	78.913	10.56
ct92-M305Pete-12	120362	23/01/2013	-2	78.929	10.498
ct92-M305Pete-12	120362	23/01/2013	-2	78.904	10.419
ct92-M305Pete-12	120362	23/01/2013	-2	78.918	10.469
ct92-M305Pete-12	120362	23/01/2013	-2	78.894	10.703
ct92-M305Pete-12	120362	23/01/2013	-2	78.841	10.605
ct92-M305Pete-12	120362	23/01/2013	-2	78.841	10.428
ct92-M305Pete-12	120362	23/01/2013	-2	78.84	10.429
ct92-M305Pete-12	120362	23/01/2013	0	78.821	10.458
ct92-M305Pete-12	120362	23/01/2013	-2	78.822	10.41
ct92-M305Pete-12	120362	23/01/2013	-2	78.81	10.421
ct92-M305Pete-12	120362	23/01/2013	-2	78.788	10.386
ct92-M305Pete-12	120362	23/01/2013	-2	78.798	10.386
ct92-M305Pete-12	120362	23/01/2013	-2	78.783	10.344
ct92-M305Pete-12	120362	23/01/2013	-2	78.791	10.323
ct92-M305Pete-12	120362	23/01/2013	-2	78.784	10.338
ct92-M305Pete-12	120362	23/01/2013	-2	78.782	10.366
ct92-M305Pete-12	120362	23/01/2013	-2	78.791	10.314
ct92-M305Pete-12	120362	23/01/2013	-2	78.777	10.306
ct92-M305Pete-12	120362	23/01/2013	-2	78.774	10.318
ct92-M305Pete-12	120362	23/01/2013	-2	78.777	10.375
ct92-M305Pete-12	120362	23/01/2013	-2	78.775	10.369
ct92-M305Pete-12	120362	23/01/2013	-2	78.772	10.346
ct92-M305Pete-12	120362	23/01/2013	-2	78.772	10.346
ct92-M305Pete-12	120362	23/01/2013	-2	78.757	10.354
ct92-M305Pete-12	120362	23/01/2013	-2	78.773	10.346
ct92-M305Pete-12	120362	23/01/2013	-2	78.773	10.338
ct92-M305Pete-12	120362	23/01/2013	-2	78.768	10.376
ct92-M305Pete-12	120362	23/01/2013	-2	78.764	10.371
ct92-M305Pete-12	120362	23/01/2013	-2	78.752	10.379
ct92-M305Pete-12	120362	23/01/2013	-2	78.756	10.323
ct92-M305Pete-12	120362	23/01/2013	-2	78.754	10.422
ct92-M305Pete-12	120362	23/01/2013	-2	78.754	10.421
ct92-M305Pete-12	120362	23/01/2013	-2	78.749	10.351
ct92-M305Pete-12	120362	23/01/2013	-1	78.758	10.468
ct92-M305Pete-12	120362	23/01/2013	-2	78.748	10.298
ct92-M305Pete-12	120362	23/01/2013	-2	78.732	10.364
ct92-M305Pete-12	120362	23/01/2013	-2	78.73	10.428
ct92-M305Pete-12	120362	23/01/2013	-2	78.715	10.554
ct92-M305Pete-12	120362	23/01/2013	-2	78.725	10.551
ct92-M305Pete-12	120362	23/01/2013	-2	78.716	10.564
ct92-M305Pete-12	120362	23/01/2013	-2	78.714	10.538
ct92-M305Pete-12	120362	23/01/2013	-2	78.71	10.575
ct92-M305Pete-12	120362	23/01/2013	-2	78.704	10.643
ct92-M305Pete-12	120362	23/01/2013	-1	78.68	10.622
ct92-M305Pete-12	120362	23/01/2013	-2	78.687	10.607
ct92-M305Pete-12	120362	23/01/2013	-1	78.68	10.615
ct92-M305Pete-12	120362	23/01/2013	-1	78.669	10.707
ct92-M305Pete-12	120362	23/01/2013	-2	78.681	10.615
ct92-M305Pete-12	120362	23/01/2013	-2	78.655	10.629
ct92-M305Pete-12	120362	23/01/2013	-2	78.635	10.687
ct92-M305Pete-12	120362	23/01/2013	-2	78.611	10.826
ct92-M305Pete-12	120362	23/01/2013	-2	78.65	10.726
ct92-M305Pete-12	120362	23/01/2013	-2	78.612	10.831
ct92-M305Pete-12	120362	23/01/2013	-2	78.612	10.837
ct92-M305Pete-12	120362	23/01/2013	-2	78.593	10.874
ct92-M305Pete-12	120362	23/01/2013	-1	78.568	10.93

ct92-M305Pete-12	120362	23/01/2013	-2	78.553	10.899
ct92-M305Pete-12	120362	23/01/2013	-2	78.525	10.964
ct92-M305Pete-12	120362	24/01/2013	-2	78.515	10.985
ct92-M305Pete-12	120362	24/01/2013	-2	78.504	10.902
ct92-M305Pete-12	120362	24/01/2013	-2	78.497	10.917
ct92-M305Pete-12	120362	24/01/2013	-2	78.447	10.937
ct92-M305Pete-12	120362	24/01/2013	-2	78.443	10.941
ct92-M305Pete-12	120362	24/01/2013	-2	78.442	11.025
ct92-M305Pete-12	120362	24/01/2013	-2	78.444	11.038
ct92-M305Pete-12	120362	24/01/2013	-2	78.445	11.011
ct92-M305Pete-12	120362	24/01/2013	-2	78.416	11.066
ct92-M305Pete-12	120362	24/01/2013	-2	78.417	11.059
ct92-M305Pete-12	120362	24/01/2013	-2	78.413	11.061
ct92-M305Pete-12	120362	24/01/2013	-2	78.398	11.127
ct92-M305Pete-12	120362	24/01/2013	-2	78.408	11.067
ct92-M305Pete-12	120362	24/01/2013	-2	78.408	11.065
ct92-M305Pete-12	120362	24/01/2013	-1	78.432	11.107
ct92-M305Pete-12	120362	24/01/2013	-2	78.429	11.107
ct92-M305Pete-12	120362	24/01/2013	-2	78.414	11.266
ct92-M305Pete-12	120362	24/01/2013	-2	78.407	11.286
ct92-M305Pete-12	120362	24/01/2013	-2	78.392	11.33
ct92-M305Pete-12	120362	24/01/2013	-2	78.364	11.337
ct92-M305Pete-12	120362	24/01/2013	-2	78.372	11.264
ct92-M305Pete-12	120362	24/01/2013	-2	78.363	11.391
ct92-M305Pete-12	120362	24/01/2013	-2	78.33	11.536
ct92-M305Pete-12	120362	24/01/2013	-2	78.344	11.46
ct92-M305Pete-12	120362	24/01/2013	-1	78.305	11.56
ct92-M305Pete-12	120362	24/01/2013	-2	78.349	11.51
ct92-M305Pete-12	120362	24/01/2013	-2	78.314	11.546
ct92-M305Pete-12	120362	24/01/2013	-2	78.334	11.495
ct92-M305Pete-12	120362	24/01/2013	-2	78.33	11.502
ct92-M305Pete-12	120362	24/01/2013	-2	78.271	11.602
ct92-M305Pete-12	120362	24/01/2013	-2	78.329	11.504
ct92-M305Pete-12	120362	24/01/2013	-2	78.328	11.508
ct92-M305Pete-12	120362	24/01/2013	-2	78.327	11.521
ct92-M305Pete-12	120362	24/01/2013	-2	78.31	11.561
ct92-M305Pete-12	120362	24/01/2013	-2	78.302	11.583
ct92-M305Pete-12	120362	24/01/2013	-2	78.28	11.622
ct92-M305Pete-12	120362	24/01/2013	-2	78.302	11.582
ct92-M305Pete-12	120362	24/01/2013	-2	78.299	11.592
ct92-M305Pete-12	120362	24/01/2013	-1	78.299	11.677
ct92-M305Pete-12	120362	24/01/2013	-2	78.266	11.743
ct92-M305Pete-12	120362	24/01/2013	-2	78.257	11.752
ct92-M305Pete-12	120362	24/01/2013	-2	78.249	11.763
ct92-M305Pete-12	120362	24/01/2013	-2	78.253	11.791
ct92-M305Pete-12	120362	24/01/2013	-2	78.253	11.785
ct92-M305Pete-12	120362	24/01/2013	-2	78.255	11.784
ct92-M305Pete-12	120362	24/01/2013	-1	78.221	11.845
ct92-M305Pete-12	120362	24/01/2013	-2	78.22	11.844
ct92-M305Pete-12	120362	24/01/2013	-2	78.22	11.844
ct92-M305Pete-12	120362	24/01/2013	-2	78.201	11.939
ct92-M305Pete-12	120362	24/01/2013	-2	78.197	11.954
ct92-M305Pete-12	120362	24/01/2013	-2	78.21	11.903
ct92-M305Pete-12	120362	24/01/2013	-2	78.181	12.016
ct92-M305Pete-12	120362	24/01/2013	-2	78.122	12.193
ct92-M305Pete-12	120362	24/01/2013	-2	78.149	12.109
ct92-M305Pete-12	120362	24/01/2013	-2	78.086	12.366
ct92-M305Pete-12	120362	24/01/2013	-2	78.095	12.377
ct92-M305Pete-12	120362	24/01/2013	-2	78.051	12.527
ct92-M305Pete-12	120362	24/01/2013	-2	78.16	12.35
ct92-M305Pete-12	120362	24/01/2013	-2	78.174	12.484
ct92-M305Pete-12	120362	24/01/2013	-2	78.171	12.324
ct92-M305Pete-12	120362	24/01/2013	-2	78.187	12.793
ct92-M305Pete-12	120362	24/01/2013	-2	78.189	12.823
ct92-M305Pete-12	120362	25/01/2013	-2	78.186	12.861
ct92-M305Pete-12	120362	25/01/2013	-2	78.182	12.908
ct92-M305Pete-12	120362	25/01/2013	-2	78.173	13.064
ct92-M305Pete-12	120362	25/01/2013	-2	78.168	13.125

ct92-M305Pete-12	120362	25/01/2013	-2	78.186	13.102
ct92-M305Pete-12	120362	25/01/2013	-2	78.166	13.211
ct92-M305Pete-12	120362	25/01/2013	-2	78.167	13.215
ct92-M305Pete-12	120362	25/01/2013	-2	78.186	13.27
ct92-M305Pete-12	120362	25/01/2013	-2	78.09	13.968
ct92-M305Pete-12	120362	25/01/2013	-2	78.118	13.709
ct92-M305Pete-12	120362	25/01/2013	-2	78.132	13.637
ct92-M305Pete-12	120362	25/01/2013	-2	78.129	13.621
ct92-M305Pete-12	120362	25/01/2013	-2	78.13	13.641
ct92-M305Pete-12	120362	25/01/2013	-2	78.12	13.823
ct92-M305Pete-12	120362	25/01/2013	-1	78.071	13.769
ct92-M305Pete-12	120362	25/01/2013	-2	78.071	13.771
ct92-M305Pete-12	120362	25/01/2013	-2	78.091	13.749
ct92-M305Pete-12	120362	25/01/2013	-2	78.084	13.64
ct92-M305Pete-12	120362	25/01/2013	-1	78.064	13.6
ct92-M305Pete-12	120362	25/01/2013	-2	78.017	13.57
ct92-M305Pete-12	120362	25/01/2013	-2	78.009	13.6
ct92-M305Pete-12	120362	25/01/2013	-2	78.019	13.641
ct92-M305Pete-12	120362	25/01/2013	-2	78.002	13.575
ct92-M305Pete-12	120362	25/01/2013	-2	78.008	13.578
ct92-M305Pete-12	120362	25/01/2013	-2	77.955	13.587
ct92-M305Pete-12	120362	25/01/2013	-2	78.01	13.603
ct92-M305Pete-12	120362	25/01/2013	-2	77.945	13.597
ct92-M305Pete-12	120362	25/01/2013	-2	77.94	13.552
ct92-M305Pete-12	120362	25/01/2013	-2	77.944	13.544
ct92-M305Pete-12	120362	25/01/2013	-2	77.959	13.638
ct92-M305Pete-12	120362	25/01/2013	-2	77.945	13.684
ct92-M305Pete-12	120362	25/01/2013	-2	77.948	13.661
ct92-M305Pete-12	120362	25/01/2013	-2	77.928	13.706
ct92-M305Pete-12	120362	25/01/2013	-2	77.928	13.677
ct92-M305Pete-12	120362	25/01/2013	-2	77.925	13.605
ct92-M305Pete-12	120362	25/01/2013	-2	77.873	13.584
ct92-M305Pete-12	120362	25/01/2013	1	77.883	13.581
ct92-M305Pete-12	120362	25/01/2013	2	77.87	13.583
ct92-M305Pete-12	120362	25/01/2013	-1	77.866	13.55
ct92-M305Pete-12	120362	25/01/2013	-2	77.866	13.608
ct92-M305Pete-12	120362	25/01/2013	-2	77.866	13.605
ct92-M305Pete-12	120362	25/01/2013	-2	77.831	13.585
ct92-M305Pete-12	120362	25/01/2013	-1	77.816	13.633
ct92-M305Pete-12	120362	25/01/2013	-2	77.824	13.637
ct92-M305Pete-12	120362	25/01/2013	-2	77.823	13.679
ct92-M305Pete-12	120362	25/01/2013	-2	77.818	13.682
ct92-M305Pete-12	120362	25/01/2013	-2	77.806	13.67
ct92-M305Pete-12	120362	25/01/2013	-2	77.778	13.688
ct92-M305Pete-12	120362	25/01/2013	-2	77.772	13.692
ct92-M305Pete-12	120362	25/01/2013	-2	77.767	13.632
ct92-M305Pete-12	120362	25/01/2013	-2	77.745	13.643
ct92-M305Pete-12	120362	25/01/2013	-2	77.753	13.625
ct92-M305Pete-12	120362	25/01/2013	-2	77.733	13.656
ct92-M305Pete-12	120362	25/01/2013	-1	77.705	13.72
ct92-M305Pete-12	120362	25/01/2013	-2	77.69	13.976
ct92-M305Pete-12	120362	25/01/2013	-2	77.726	13.647
ct92-M305Pete-12	120362	25/01/2013	-2	77.695	13.929
ct92-M305Pete-12	120362	25/01/2013	-2	77.664	13.95
ct92-M305Pete-12	120362	25/01/2013	-2	77.716	13.864
ct92-M305Pete-12	120362	25/01/2013	-2	77.715	13.861
ct92-M305Pete-12	120362	26/01/2013	-2	77.722	13.846
ct92-M305Pete-12	120362	26/01/2013	-2	77.706	13.918
ct92-M305Pete-12	120362	26/01/2013	-2	77.708	13.829
ct92-M305Pete-12	120362	26/01/2013	-2	77.678	13.947
ct92-M305Pete-12	120362	26/01/2013	-2	77.694	13.916
ct92-M305Pete-12	120362	26/01/2013	-2	77.698	13.907
ct92-M305Pete-12	120362	26/01/2013	-2	77.698	13.966
ct92-M305Pete-12	120362	26/01/2013	-2	77.682	13.968
ct92-M305Pete-12	120362	26/01/2013	-2	77.696	13.952
ct92-M305Pete-12	120362	26/01/2013	-2	77.695	13.928
ct92-M305Pete-12	120362	26/01/2013	-2	77.69	13.968
ct92-M305Pete-12	120362	26/01/2013	-2	77.702	13.891

ct92-M305Pete-12	120362	26/01/2013	-1	77.679	13.909
ct92-M305Pete-12	120362	26/01/2013	-2	77.688	13.908
ct92-M305Pete-12	120362	26/01/2013	-2	77.682	13.898
ct92-M305Pete-12	120362	26/01/2013	-2	77.73	13.865
ct92-M305Pete-12	120362	26/01/2013	-2	77.728	13.943
ct92-M305Pete-12	120362	26/01/2013	-2	77.71	13.92
ct92-M305Pete-12	120362	26/01/2013	-2	77.68	13.878
ct92-M305Pete-12	120362	26/01/2013	-2	77.672	13.884
ct92-M305Pete-12	120362	26/01/2013	-2	77.688	13.869
ct92-M305Pete-12	120362	26/01/2013	-2	77.695	13.922
ct92-M305Pete-12	120362	26/01/2013	-2	77.684	13.878
ct92-M305Pete-12	120362	26/01/2013	-2	77.708	13.958
ct92-M305Pete-12	120362	26/01/2013	-2	77.691	13.918
ct92-M305Pete-12	120362	26/01/2013	-2	77.694	13.918
ct92-M305Pete-12	120362	26/01/2013	-2	77.688	13.916
ct92-M305Pete-12	120362	26/01/2013	-2	77.685	13.933
ct92-M305Pete-12	120362	26/01/2013	-2	77.7	13.825
ct92-M305Pete-12	120362	26/01/2013	-2	77.663	13.884
ct92-M305Pete-12	120362	26/01/2013	-2	77.704	13.83
ct92-M305Pete-12	120362	26/01/2013	-2	77.709	13.838
ct92-M305Pete-12	120362	26/01/2013	-2	77.686	13.799
ct92-M305Pete-12	120362	26/01/2013	-2	77.703	13.688
ct92-M305Pete-12	120362	26/01/2013	-2	77.723	13.742
ct92-M305Pete-12	120362	26/01/2013	1	77.719	13.779
ct92-M305Pete-12	120362	26/01/2013	0	77.719	13.778
ct92-M305Pete-12	120362	26/01/2013	-2	77.701	13.718
ct92-M305Pete-12	120362	26/01/2013	-2	77.703	13.723
ct92-M305Pete-12	120362	26/01/2013	-2	77.702	13.716
ct92-M305Pete-12	120362	26/01/2013	-2	77.702	13.724
ct92-M305Pete-12	120362	26/01/2013	-2	77.701	13.734
ct92-M305Pete-12	120362	26/01/2013	-2	77.707	13.711
ct92-M305Pete-12	120362	26/01/2013	-2	77.711	13.738
ct92-M305Pete-12	120362	26/01/2013	-2	77.709	13.68
ct92-M305Pete-12	120362	26/01/2013	-2	77.723	13.729
ct92-M305Pete-12	120362	26/01/2013	-2	77.716	13.711
ct92-M305Pete-12	120362	26/01/2013	-2	77.724	13.638
ct92-M305Pete-12	120362	26/01/2013	-2	77.722	13.628
ct92-M305Pete-12	120362	26/01/2013	-1	77.695	13.657
ct92-M305Pete-12	120362	26/01/2013	-2	77.693	13.739
ct92-M305Pete-12	120362	26/01/2013	-1	77.726	13.648
ct92-M305Pete-12	120362	26/01/2013	-2	77.7	13.523
ct92-M305Pete-12	120362	26/01/2013	-2	77.684	13.582
ct92-M305Pete-12	120362	26/01/2013	-2	77.684	13.582
ct92-M305Pete-12	120362	26/01/2013	-1	77.67	13.552
ct92-M305Pete-12	120362	26/01/2013	1	77.684	13.491
ct92-M305Pete-12	120362	26/01/2013	-2	77.676	13.455
ct92-M305Pete-12	120362	26/01/2013	-2	77.68	13.313
ct92-M305Pete-12	120362	26/01/2013	-2	77.682	13.314
ct92-M305Pete-12	120362	26/01/2013	-2	77.681	13.327
ct92-M305Pete-12	120362	27/01/2013	-2	77.705	13.346
ct92-M305Pete-12	120362	27/01/2013	-2	77.706	13.26
ct92-M305Pete-12	120362	27/01/2013	-2	77.708	13.244
ct92-M305Pete-12	120362	27/01/2013	-2	77.708	13.284
ct92-M305Pete-12	120362	27/01/2013	-2	77.713	13.302
ct92-M305Pete-12	120362	27/01/2013	-2	77.713	13.223
ct92-M305Pete-12	120362	27/01/2013	-2	77.711	13.33
ct92-M305Pete-12	120362	27/01/2013	-1	77.726	13.32
ct92-M305Pete-12	120362	27/01/2013	-1	77.721	13.337
ct92-M305Pete-12	120362	27/01/2013	-2	77.728	13.314
ct92-M305Pete-12	120362	27/01/2013	-2	77.73	13.289
ct92-M305Pete-12	120362	27/01/2013	-2	77.729	13.301
ct92-M305Pete-12	120362	27/01/2013	-2	77.736	13.246
ct92-M305Pete-12	120362	27/01/2013	-1	77.731	13.246
ct92-M305Pete-12	120362	27/01/2013	-1	77.727	13.255
ct92-M305Pete-12	120362	27/01/2013	-2	77.724	13.283
ct92-M305Pete-12	120362	27/01/2013	-2	77.734	13.322
ct92-M305Pete-12	120362	27/01/2013	-2	77.742	13.193
ct92-M305Pete-12	120362	27/01/2013	-2	77.731	13.312

ct92-M305Pete-12	120362	27/01/2013	-2	77.73	13.315
ct92-M305Pete-12	120362	27/01/2013	-2	77.733	13.334
ct92-M305Pete-12	120362	27/01/2013	-2	77.737	13.306
ct92-M305Pete-12	120362	27/01/2013	-2	77.735	13.354
ct92-M305Pete-12	120362	27/01/2013	-2	77.736	13.315
ct92-M305Pete-12	120362	27/01/2013	-2	77.737	13.314
ct92-M305Pete-12	120362	27/01/2013	-2	77.739	13.346
ct92-M305Pete-12	120362	27/01/2013	-2	77.758	13.372
ct92-M305Pete-12	120362	27/01/2013	-2	77.751	13.456
ct92-M305Pete-12	120362	27/01/2013	-2	77.747	13.433
ct92-M305Pete-12	120362	27/01/2013	-2	77.747	13.432
ct92-M305Pete-12	120362	27/01/2013	-2	77.751	13.456
ct92-M305Pete-12	120362	27/01/2013	-2	77.747	13.437
ct92-M305Pete-12	120362	27/01/2013	-2	77.76	13.536
ct92-M305Pete-12	120362	27/01/2013	-1	77.753	13.564
ct92-M305Pete-12	120362	27/01/2013	-2	77.759	13.528
ct92-M305Pete-12	120362	27/01/2013	-2	77.759	13.596
ct92-M305Pete-12	120362	27/01/2013	-2	77.749	13.624
ct92-M305Pete-12	120362	27/01/2013	-2	77.749	13.561
ct92-M305Pete-12	120362	27/01/2013	-2	77.744	13.714
ct92-M305Pete-12	120362	27/01/2013	-1	77.721	13.64
ct92-M305Pete-12	120362	27/01/2013	-2	77.738	13.596
ct92-M305Pete-12	120362	27/01/2013	0	77.722	13.621
ct92-M305Pete-12	120362	27/01/2013	-2	77.722	13.636
ct92-M305Pete-12	120362	27/01/2013	-2	77.723	13.673
ct92-M305Pete-12	120362	27/01/2013	-2	77.722	13.633
ct92-M305Pete-12	120362	27/01/2013	-2	77.717	13.724
ct92-M305Pete-12	120362	27/01/2013	-2	77.696	13.726
ct92-M305Pete-12	120362	27/01/2013	-2	77.716	13.735
ct92-M305Pete-12	120362	27/01/2013	-2	77.716	13.738
ct92-M305Pete-12	120362	27/01/2013	-2	77.706	13.736
ct92-M305Pete-12	120362	27/01/2013	-2	77.704	13.748
ct92-M305Pete-12	120362	27/01/2013	-2	77.704	13.746
ct92-M305Pete-12	120362	27/01/2013	-2	77.702	13.768
ct92-M305Pete-12	120362	27/01/2013	-2	77.7	13.805
ct92-M305Pete-12	120362	27/01/2013	-2	77.702	13.808
ct92-M305Pete-12	120362	27/01/2013	-2	77.701	13.812
ct92-M305Pete-12	120362	27/01/2013	-2	77.702	13.809
ct92-M305Pete-12	120362	27/01/2013	-2	77.719	13.983
ct92-M305Pete-12	120362	27/01/2013	-2	77.723	13.983
ct92-M305Pete-12	120362	27/01/2013	-2	77.706	13.846
ct92-M305Pete-12	120362	27/01/2013	-2	77.706	13.876
ct92-M305Pete-12	120362	27/01/2013	-2	77.714	13.922
ct92-M305Pete-12	120362	27/01/2013	-2	77.714	13.996
ct92-M305Pete-12	120362	27/01/2013	-2	77.732	13.959
ct92-M305Pete-12	120362	27/01/2013	-2	77.716	13.913
ct92-M305Pete-12	120362	27/01/2013	-2	77.719	13.919
ct92-M305Pete-12	120362	27/01/2013	-2	77.719	13.918
ct92-M305Pete-12	120362	27/01/2013	-2	77.719	13.957
ct92-M305Pete-12	120362	28/01/2013	-2	77.762	13.685
ct92-M305Pete-12	120362	28/01/2013	-2	77.726	13.974
ct92-M305Pete-12	120362	28/01/2013	-2	77.723	14.088
ct92-M305Pete-12	120362	28/01/2013	-2	77.725	14.092
ct92-M305Pete-12	120362	28/01/2013	-2	77.743	14.111
ct92-M305Pete-12	120362	28/01/2013	-2	77.743	14.125
ct92-M305Pete-12	120362	28/01/2013	-2	77.728	14.093
ct92-M305Pete-12	120362	28/01/2013	-2	77.732	14.119
ct92-M305Pete-12	120362	28/01/2013	-2	77.732	14.119
ct92-M305Pete-12	120362	28/01/2013	-2	77.751	14.184
ct92-M305Pete-12	120362	28/01/2013	-2	77.754	14.209
ct92-M305Pete-12	120362	28/01/2013	-2	77.741	14.207
ct92-M305Pete-12	120362	28/01/2013	-2	77.748	14.208
ct92-M305Pete-12	120362	28/01/2013	-2	77.75	14.221
ct92-M305Pete-12	120362	28/01/2013	-2	77.752	14.221
ct92-M305Pete-12	120362	28/01/2013	-2	77.748	14.182
ct92-M305Pete-12	120362	28/01/2013	-2	77.748	14.182
ct92-M305Pete-12	120362	28/01/2013	-2	77.75	14.19
ct92-M305Pete-12	120362	28/01/2013	-2	77.75	14.19

ct92-M305Pete-12	120362	28/01/2013	-2	77.752	14.205
ct92-M305Pete-12	120362	28/01/2013	-2	77.749	14.203
ct92-M305Pete-12	120362	28/01/2013	-2	77.754	14.299
ct92-M305Pete-12	120362	28/01/2013	-2	77.74	14.307
ct92-M305Pete-12	120362	28/01/2013	-2	77.746	14.277
ct92-M305Pete-12	120362	28/01/2013	-2	77.744	14.273
ct92-M305Pete-12	120362	28/01/2013	-2	77.738	14.311
ct92-M305Pete-12	120362	28/01/2013	-2	77.738	14.307
ct92-M305Pete-12	120362	28/01/2013	-2	77.742	14.321
ct92-M305Pete-12	120362	28/01/2013	-2	77.75	14.271
ct92-M305Pete-12	120362	28/01/2013	-2	77.742	14.33
ct92-M305Pete-12	120362	28/01/2013	-2	77.746	14.323
ct92-M305Pete-12	120362	28/01/2013	-2	77.746	14.326
ct92-M305Pete-12	120362	28/01/2013	-2	77.743	14.309
ct92-M305Pete-12	120362	28/01/2013	-2	77.743	14.333
ct92-M305Pete-12	120362	28/01/2013	-2	77.755	14.254
ct92-M305Pete-12	120362	28/01/2013	-2	77.766	14.361
ct92-M305Pete-12	120362	28/01/2013	-2	77.766	14.358
ct92-M305Pete-12	120362	28/01/2013	-2	77.759	14.365
ct92-M305Pete-12	120362	28/01/2013	-2	77.748	14.247
ct92-M305Pete-12	120362	28/01/2013	-2	77.751	14.253
ct92-M305Pete-12	120362	28/01/2013	-2	77.748	14.23
ct92-M305Pete-12	120362	28/01/2013	-2	77.742	14.186
ct92-M305Pete-12	120362	28/01/2013	-2	77.741	14.146
ct92-M305Pete-12	120362	28/01/2013	-2	77.742	14.138
ct92-M305Pete-12	120362	28/01/2013	-2	77.746	14.096
ct92-M305Pete-12	120362	28/01/2013	-2	77.746	14.075
ct92-M305Pete-12	120362	28/01/2013	-2	77.727	14.011
ct92-M305Pete-12	120362	28/01/2013	-2	77.729	13.973
ct92-M305Pete-12	120362	28/01/2013	-2	77.727	14
ct92-M305Pete-12	120362	28/01/2013	-2	77.723	13.802
ct92-M305Pete-12	120362	28/01/2013	-2	77.711	13.781
ct92-M305Pete-12	120362	28/01/2013	-2	77.709	13.771
ct92-M305Pete-12	120362	28/01/2013	-2	77.708	13.768
ct92-M305Pete-12	120362	28/01/2013	-2	77.71	13.771
ct92-M305Pete-12	120362	28/01/2013	-2	77.709	13.773
ct92-M305Pete-12	120362	28/01/2013	-2	77.709	13.785
ct92-M305Pete-12	120362	28/01/2013	-2	77.705	13.749
ct92-M305Pete-12	120362	28/01/2013	-2	77.725	13.768
ct92-M305Pete-12	120362	28/01/2013	-2	77.725	13.797
ct92-M305Pete-12	120362	28/01/2013	-2	77.725	13.768
ct92-M305Pete-12	120362	28/01/2013	-2	77.724	13.799
ct92-M305Pete-12	120362	28/01/2013	-2	77.725	13.77
ct92-M305Pete-12	120362	28/01/2013	-2	77.723	13.781
ct92-M305Pete-12	120362	28/01/2013	-2	77.725	13.766
ct92-M305Pete-12	120362	28/01/2013	-2	77.725	13.79
ct92-M305Pete-12	120362	29/01/2013	-2	77.717	13.754
ct92-M305Pete-12	120362	29/01/2013	-2	77.731	13.829
ct92-M305Pete-12	120362	29/01/2013	-2	77.718	13.828
ct92-M305Pete-12	120362	29/01/2013	-2	77.713	13.852
ct92-M305Pete-12	120362	29/01/2013	-2	77.714	13.858
ct92-M305Pete-12	120362	29/01/2013	-2	77.72	13.827
ct92-M305Pete-12	120362	29/01/2013	-2	77.715	13.862
ct92-M305Pete-12	120362	29/01/2013	-2	77.722	13.868
ct92-M305Pete-12	120362	29/01/2013	2	77.727	13.834
ct92-M305Pete-12	120362	29/01/2013	-2	77.723	13.845
ct92-M305Pete-12	120362	29/01/2013	-2	77.726	13.845
ct92-M305Pete-12	120362	29/01/2013	-2	77.726	13.848
ct92-M305Pete-12	120362	29/01/2013	-2	77.735	13.883
ct92-M305Pete-12	120362	29/01/2013	0	77.729	13.876
ct92-M305Pete-12	120362	29/01/2013	-2	77.736	13.881
ct92-M305Pete-12	120362	29/01/2013	-2	77.734	13.89
ct92-M305Pete-12	120362	29/01/2013	-2	77.734	13.902
ct92-M305Pete-12	120362	29/01/2013	-2	77.716	13.882
ct92-M305Pete-12	120362	29/01/2013	-2	77.721	13.855
ct92-M305Pete-12	120362	29/01/2013	0	77.69	13.932
ct92-M305Pete-12	120362	29/01/2013	-2	77.679	13.928
ct92-M305Pete-12	120362	29/01/2013	-2	77.681	13.929

ct92-M305Pete-12	120362	29/01/2013	-2	77.683	13.915
ct92-M305Pete-12	120362	29/01/2013	-2	77.677	13.945
ct92-M305Pete-12	120362	29/01/2013	-2	77.696	13.991
ct92-M305Pete-12	120362	29/01/2013	-2	77.706	13.922
ct92-M305Pete-12	120362	29/01/2013	-2	77.678	13.948
ct92-M305Pete-12	120362	29/01/2013	-2	77.652	13.959
ct92-M305Pete-12	120362	29/01/2013	-2	77.633	14.089
ct92-M305Pete-12	120362	29/01/2013	-2	77.646	14.146
ct92-M305Pete-12	120362	29/01/2013	-2	77.604	14.087
ct92-M305Pete-12	120362	29/01/2013	-2	77.601	14.099
ct92-M305Pete-12	120362	29/01/2013	-2	77.599	14.068
ct92-M305Pete-12	120362	29/01/2013	-2	77.597	14.075
ct92-M305Pete-12	120362	29/01/2013	-2	77.592	14.086
ct92-M305Pete-12	120362	29/01/2013	-2	77.589	14.068
ct92-M305Pete-12	120362	29/01/2013	-2	77.581	14.09
ct92-M305Pete-12	120362	29/01/2013	-2	77.583	14.032
ct92-M305Pete-12	120362	29/01/2013	-2	77.576	14.048
ct92-M305Pete-12	120362	29/01/2013	-2	77.576	14.031
ct92-M305Pete-12	120362	29/01/2013	-2	77.57	14.046
ct92-M305Pete-12	120362	29/01/2013	-2	77.576	14.024
ct92-M305Pete-12	120362	29/01/2013	-2	77.577	14.019
ct92-M305Pete-12	120362	29/01/2013	-2	77.547	14.031
ct92-M305Pete-12	120362	29/01/2013	-2	77.547	14.004
ct92-M305Pete-12	120362	29/01/2013	-1	77.544	13.973
ct92-M305Pete-12	120362	29/01/2013	-1	77.543	13.972
ct92-M305Pete-12	120362	29/01/2013	-2	77.547	13.978
ct92-M305Pete-12	120362	29/01/2013	-2	77.549	13.964
ct92-M305Pete-12	120362	29/01/2013	-2	77.547	13.942
ct92-M305Pete-12	120362	29/01/2013	-2	77.53	13.903
ct92-M305Pete-12	120362	29/01/2013	-2	77.524	13.894
ct92-M305Pete-12	120362	29/01/2013	-2	77.516	13.923
ct92-M305Pete-12	120362	29/01/2013	-2	77.504	13.996
ct92-M305Pete-12	120362	29/01/2013	-1	77.499	13.907
ct92-M305Pete-12	120362	29/01/2013	-2	77.498	13.893
ct92-M305Pete-12	120362	29/01/2013	-2	77.497	13.876
ct92-M305Pete-12	120362	29/01/2013	-1	77.503	13.847
ct92-M305Pete-12	120362	29/01/2013	-1	77.487	13.791
ct92-M305Pete-12	120362	29/01/2013	-2	77.492	13.84
ct92-M305Pete-12	120362	29/01/2013	-2	77.482	13.787
ct92-M305Pete-12	120362	29/01/2013	-2	77.477	13.783
ct92-M305Pete-12	120362	29/01/2013	-2	77.448	13.77
ct92-M305Pete-12	120362	29/01/2013	-2	77.47	13.773
ct92-M305Pete-12	120362	29/01/2013	-2	77.467	13.791
ct92-M305Pete-12	120362	29/01/2013	-2	77.468	13.769
ct92-M305Pete-12	120362	29/01/2013	-2	77.468	13.769
ct92-M305Pete-12	120362	29/01/2013	-2	77.462	13.771
ct92-M305Pete-12	120362	29/01/2013	-2	77.461	13.771
ct92-M305Pete-12	120362	29/01/2013	3	77.464	13.819
ct92-M305Pete-12	120362	29/01/2013	2	77.454	13.839
ct92-M305Pete-12	120362	29/01/2013	-2	77.454	13.838
ct92-M305Pete-12	120362	29/01/2013	-2	77.456	13.827
ct92-M305Pete-12	120362	29/01/2013	-2	77.452	13.831
ct92-M305Pete-12	120362	29/01/2013	-2	77.454	13.829
ct92-M305Pete-12	120362	30/01/2013	-2	77.458	13.842
ct92-M305Pete-12	120362	30/01/2013	-2	77.434	13.836
ct92-M305Pete-12	120362	30/01/2013	-2	77.428	13.833
ct92-M305Pete-12	120362	30/01/2013	-2	77.398	13.772
ct92-M305Pete-12	120362	30/01/2013	-2	77.393	13.782
ct92-M305Pete-12	120362	30/01/2013	-2	77.4	13.78
ct92-M305Pete-12	120362	30/01/2013	-2	77.38	13.79
ct92-M305Pete-12	120362	30/01/2013	-2	77.384	13.843
ct92-M305Pete-12	120362	30/01/2013	-2	77.379	13.785
ct92-M305Pete-12	120362	30/01/2013	0	77.382	13.84
ct92-M305Pete-12	120362	30/01/2013	-1	77.367	13.911
ct92-M305Pete-12	120362	30/01/2013	-2	77.362	13.91
ct92-M305Pete-12	120362	30/01/2013	-1	77.333	14.024
ct92-M305Pete-12	120362	30/01/2013	3	77.335	14.03
ct92-M305Pete-12	120362	30/01/2013	-2	77.352	13.924

ct92-M305Pete-12	120362	30/01/2013	-2	77.346	13.941
ct92-M305Pete-12	120362	30/01/2013	-1	77.353	13.91
ct92-M305Pete-12	120362	30/01/2013	-2	77.31	13.98
ct92-M305Pete-12	120362	30/01/2013	-2	77.284	13.93
ct92-M305Pete-12	120362	30/01/2013	0	77.272	13.998
ct92-M305Pete-12	120362	30/01/2013	-2	77.275	13.974
ct92-M305Pete-12	120362	30/01/2013	2	77.258	14.163
ct92-M305Pete-12	120362	30/01/2013	2	77.258	14.163
ct92-M305Pete-12	120362	30/01/2013	-2	77.256	14.162
ct92-M305Pete-12	120362	30/01/2013	-2	77.238	14.171
ct92-M305Pete-12	120362	30/01/2013	-1	77.197	14.354
ct92-M305Pete-12	120362	30/01/2013	0	77.251	14.201
ct92-M305Pete-12	120362	30/01/2013	-2	77.206	14.304
ct92-M305Pete-12	120362	30/01/2013	-2	77.201	14.31
ct92-M305Pete-12	120362	30/01/2013	-2	77.202	14.315
ct92-M305Pete-12	120362	30/01/2013	3	77.178	14.309
ct92-M305Pete-12	120362	30/01/2013	2	77.173	14.308
ct92-M305Pete-12	120362	30/01/2013	3	77.169	14.309
ct92-M305Pete-12	120362	30/01/2013	3	77.17	14.33
ct92-M305Pete-12	120362	30/01/2013	-2	77.167	14.327
ct92-M305Pete-12	120362	30/01/2013	1	77.165	14.292
ct92-M305Pete-12	120362	30/01/2013	3	77.17	14.292
ct92-M305Pete-12	120362	30/01/2013	3	77.169	14.284
ct92-M305Pete-12	120362	30/01/2013	3	77.169	14.285
ct92-M305Pete-12	120362	30/01/2013	3	77.171	14.269
ct92-M305Pete-12	120362	30/01/2013	1	77.175	14.223
ct92-M305Pete-12	120362	30/01/2013	3	77.17	14.258
ct92-M305Pete-12	120362	30/01/2013	-2	77.167	14.326
ct92-M305Pete-12	120362	30/01/2013	-2	77.118	14.291
ct92-M305Pete-12	120362	30/01/2013	-2	77.13	14.284
ct92-M305Pete-12	120362	30/01/2013	-2	77.135	14.313
ct92-M305Pete-12	120362	30/01/2013	-2	77.132	14.31
ct92-M305Pete-12	120362	30/01/2013	-2	77.116	14.353
ct92-M305Pete-12	120362	30/01/2013	-2	77.102	14.233
ct92-M305Pete-12	120362	30/01/2013	-2	77.087	14.244
ct92-M305Pete-12	120362	30/01/2013	-2	77.11	14.288
ct92-M305Pete-12	120362	30/01/2013	-2	77.101	14.303
ct92-M305Pete-12	120362	30/01/2013	-2	77.068	14.395
ct92-M305Pete-12	120362	30/01/2013	-2	77.078	14.47
ct92-M305Pete-12	120362	30/01/2013	-2	77.092	14.515
ct92-M305Pete-12	120362	30/01/2013	-2	77.058	14.495
ct92-M305Pete-12	120362	30/01/2013	-2	77.076	14.479
ct92-M305Pete-12	120362	30/01/2013	-2	77.058	14.511
ct92-M305Pete-12	120362	30/01/2013	-2	77.06	14.506
ct92-M305Pete-12	120362	30/01/2013	-2	77.087	14.482
ct92-M305Pete-12	120362	30/01/2013	-2	77.071	14.508
ct92-M305Pete-12	120362	31/01/2013	-2	77.036	14.603
ct92-M305Pete-12	120362	31/01/2013	-2	77.036	14.603
ct92-M305Pete-12	120362	31/01/2013	-2	77.037	14.591
ct92-M305Pete-12	120362	31/01/2013	-2	77.025	14.679
ct92-M305Pete-12	120362	31/01/2013	-2	77.029	14.665
ct92-M305Pete-12	120362	31/01/2013	-2	77.028	14.734
ct92-M305Pete-12	120362	31/01/2013	-2	77.021	14.766
ct92-M305Pete-12	120362	31/01/2013	-2	77.03	14.861
ct92-M305Pete-12	120362	31/01/2013	-2	77.032	14.889
ct92-M305Pete-12	120362	31/01/2013	-1	77.032	14.886
ct92-M305Pete-12	120362	31/01/2013	-1	77.041	14.864
ct92-M305Pete-12	120362	31/01/2013	-2	77.041	14.885
ct92-M305Pete-12	120362	31/01/2013	-2	77.032	14.916
ct92-M305Pete-12	120362	31/01/2013	-2	77.024	14.953
ct92-M305Pete-12	120362	31/01/2013	-2	77.016	14.878
ct92-M305Pete-12	120362	31/01/2013	-1	77.024	14.935
ct92-M305Pete-12	120362	31/01/2013	-1	77.021	14.948
ct92-M305Pete-12	120362	31/01/2013	-2	77.023	14.964
ct92-M305Pete-12	120362	31/01/2013	-2	77.023	14.957
ct92-M305Pete-12	120362	31/01/2013	-2	77.021	14.988
ct92-M305Pete-12	120362	31/01/2013	-2	77.022	14.919
ct92-M305Pete-12	120362	31/01/2013	-2	77.015	15.039

ct92-M305Pete-12	120362	31/01/2013	-2	77.022	14.963
ct92-M305Pete-12	120362	31/01/2013	-2	77.015	15.053
ct92-M305Pete-12	120362	31/01/2013	-2	77.021	15.059
ct92-M305Pete-12	120362	31/01/2013	-2	77.011	15.079
ct92-M305Pete-12	120362	31/01/2013	-2	77.006	15.095
ct92-M305Pete-12	120362	31/01/2013	-2	76.996	15.164
ct92-M305Pete-12	120362	31/01/2013	-2	77	15.168
ct92-M305Pete-12	120362	31/01/2013	-1	76.996	15.114
ct92-M305Pete-12	120362	31/01/2013	-2	77.002	15.166
ct92-M305Pete-12	120362	31/01/2013	-2	76.997	15.207
ct92-M305Pete-12	120362	31/01/2013	-2	76.996	15.21
ct92-M305Pete-12	120362	31/01/2013	-1	77.025	15.142
ct92-M305Pete-12	120362	31/01/2013	-2	76.989	15.131
ct92-M305Pete-12	120362	31/01/2013	-1	76.97	15.122
ct92-M305Pete-12	120362	31/01/2013	-2	76.965	15.181
ct92-M305Pete-12	120362	31/01/2013	-2	76.992	15.146
ct92-M305Pete-12	120362	31/01/2013	-2	76.987	15.18
ct92-M305Pete-12	120362	31/01/2013	-2	76.988	15.18
ct92-M305Pete-12	120362	31/01/2013	-2	76.979	15.249
ct92-M305Pete-12	120362	31/01/2013	-2	76.942	15.274
ct92-M305Pete-12	120362	31/01/2013	-2	76.94	15.225
ct92-M305Pete-12	120362	31/01/2013	-2	76.933	15.274
ct92-M305Pete-12	120362	31/01/2013	-2	76.963	15.369
ct92-M305Pete-12	120362	31/01/2013	-2	76.963	15.368
ct92-M305Pete-12	120362	31/01/2013	-1	76.886	15.274
ct92-M305Pete-12	120362	31/01/2013	-2	76.919	15.255
ct92-M305Pete-12	120362	31/01/2013	-2	76.92	15.259
ct92-M305Pete-12	120362	31/01/2013	-2	76.909	15.357
ct92-M305Pete-12	120362	31/01/2013	-2	76.903	15.426
ct92-M305Pete-12	120362	31/01/2013	-2	76.873	15.375
ct92-M305Pete-12	120362	31/01/2013	-2	76.863	15.326
ct92-M305Pete-12	120362	31/01/2013	-2	76.882	15.234
ct92-M305Pete-12	120362	31/01/2013	-2	76.855	15.412
ct92-M305Pete-12	120362	31/01/2013	-2	76.848	15.341
ct92-M305Pete-12	120362	31/01/2013	-2	76.852	15.344
ct92-M305Pete-12	120362	31/01/2013	-2	76.853	15.384
ct92-M305Pete-12	120362	31/01/2013	-2	76.85	15.402
ct92-M305Pete-12	120362	31/01/2013	-2	76.849	15.387
ct92-M305Pete-12	120362	31/01/2013	-1	76.842	15.331
ct92-M305Pete-12	120362	31/01/2013	-2	76.823	15.419
ct92-M305Pete-12	120362	31/01/2013	-2	76.837	15.384
ct92-M305Pete-12	120362	31/01/2013	-2	76.835	15.357
ct92-M305Pete-12	120362	31/01/2013	-2	76.835	15.394
ct92-M305Pete-12	120362	01/02/2013	-2	76.851	15.278
ct92-M305Pete-12	120362	01/02/2013	-2	76.832	15.395
ct92-M305Pete-12	120362	01/02/2013	-2	76.853	15.266
ct92-M305Pete-12	120362	01/02/2013	-2	76.854	15.303
ct92-M305Pete-12	120362	01/02/2013	-2	76.839	15.283
ct92-M305Pete-12	120362	01/02/2013	-1	76.84	15.247
ct92-M305Pete-12	120362	01/02/2013	-1	76.84	15.246
ct92-M305Pete-12	120362	01/02/2013	-2	76.84	15.257
ct92-M305Pete-12	120362	01/02/2013	-2	76.841	15.261
ct92-M305Pete-12	120362	01/02/2013	-2	76.853	15.291
ct92-M305Pete-12	120362	01/02/2013	-2	76.852	15.277
ct92-M305Pete-12	120362	01/02/2013	-2	76.853	15.27
ct92-M305Pete-12	120362	01/02/2013	-2	76.848	15.267
ct92-M305Pete-12	120362	01/02/2013	-2	76.847	15.264
ct92-M305Pete-12	120362	01/02/2013	-2	76.851	15.245
ct92-M305Pete-12	120362	01/02/2013	-1	76.817	15.238
ct92-M305Pete-12	120362	01/02/2013	-2	76.849	15.251
ct92-M305Pete-12	120362	01/02/2013	-2	76.848	15.251
ct92-M305Pete-12	120362	01/02/2013	-2	76.848	15.249
ct92-M305Pete-12	120362	01/02/2013	-2	76.848	15.243
ct92-M305Pete-12	120362	01/02/2013	-2	76.834	15.272
ct92-M305Pete-12	120362	01/02/2013	-2	76.841	15.283
ct92-M305Pete-12	120362	01/02/2013	-2	76.849	15.281
ct92-M305Pete-12	120362	01/02/2013	1	76.846	15.281
ct92-M305Pete-12	120362	01/02/2013	-2	76.846	15.279

ct92-M305Pete-12	120362	01/02/2013	-1	76.842	15.264
ct92-M305Pete-12	120362	01/02/2013	-1	76.841	15.27
ct92-M305Pete-12	120362	01/02/2013	-2	76.846	15.274
ct92-M305Pete-12	120362	01/02/2013	-1	76.835	15.272
ct92-M305Pete-12	120362	01/02/2013	-1	76.837	15.262
ct92-M305Pete-12	120362	01/02/2013	-1	76.835	15.257
ct92-M305Pete-12	120362	01/02/2013	-1	76.833	15.25
ct92-M305Pete-12	120362	01/02/2013	1	76.838	15.249
ct92-M305Pete-12	120362	01/02/2013	-2	76.85	15.244
ct92-M305Pete-12	120362	01/02/2013	-1	76.828	15.239
ct92-M305Pete-12	120362	01/02/2013	-2	76.823	15.283
ct92-M305Pete-12	120362	01/02/2013	2	76.823	15.274
ct92-M305Pete-12	120362	01/02/2013	-1	76.818	15.248
ct92-M305Pete-12	120362	01/02/2013	-2	76.819	15.232
ct92-M305Pete-12	120362	01/02/2013	-2	76.831	15.221
ct92-M305Pete-12	120362	01/02/2013	-1	76.821	15.218
ct92-M305Pete-12	120362	01/02/2013	-2	76.821	15.22
ct92-M305Pete-12	120362	01/02/2013	-2	76.817	15.236
ct92-M305Pete-12	120362	01/02/2013	-2	76.817	15.239
ct92-M305Pete-12	120362	01/02/2013	-2	76.82	15.211
ct92-M305Pete-12	120362	01/02/2013	2	76.819	15.242
ct92-M305Pete-12	120362	01/02/2013	1	76.813	15.23
ct92-M305Pete-12	120362	01/02/2013	-1	76.805	15.215
ct92-M305Pete-12	120362	01/02/2013	-1	76.816	15.288
ct92-M305Pete-12	120362	01/02/2013	-2	76.817	15.281
ct92-M305Pete-12	120362	01/02/2013	-2	76.808	15.304
ct92-M305Pete-12	120362	01/02/2013	-2	76.795	15.05
ct92-M305Pete-12	120362	01/02/2013	-2	76.795	15.128
ct92-M305Pete-12	120362	01/02/2013	-1	76.785	15.067
ct92-M305Pete-12	120362	01/02/2013	-2	76.786	15.075
ct92-M305Pete-12	120362	01/02/2013	-2	76.781	15.079
ct92-M305Pete-12	120362	01/02/2013	-2	76.812	15.235
ct92-M305Pete-12	120362	01/02/2013	-2	76.812	15.234
ct92-M305Pete-12	120362	01/02/2013	-2	76.777	15.233
ct92-M305Pete-12	120362	01/02/2013	-1	76.772	15.144
ct92-M305Pete-12	120362	01/02/2013	-2	76.77	15.088
ct92-M305Pete-12	120362	01/02/2013	-2	76.771	15.101
ct92-M305Pete-12	120362	01/02/2013	-1	76.777	15.316
ct92-M305Pete-12	120362	01/02/2013	1	76.779	15.177
ct92-M305Pete-12	120362	02/02/2013	-2	76.78	15.183
ct92-M305Pete-12	120362	02/02/2013	-2	76.782	15.178
ct92-M305Pete-12	120362	02/02/2013	-2	76.771	15.164
ct92-M305Pete-12	120362	02/02/2013	-2	76.765	15.138
ct92-M305Pete-12	120362	02/02/2013	-2	76.755	15.063
ct92-M305Pete-12	120362	02/02/2013	-2	76.753	15.058
ct92-M305Pete-12	120362	02/02/2013	-2	76.749	15.051
ct92-M305Pete-12	120362	02/02/2013	-2	76.738	15.039
ct92-M305Pete-12	120362	02/02/2013	-2	76.752	15.048
ct92-M305Pete-12	120362	02/02/2013	-2	76.747	15.03
ct92-M305Pete-12	120362	02/02/2013	-2	76.74	15.061
ct92-M305Pete-12	120362	02/02/2013	-2	76.741	15.067
ct92-M305Pete-12	120362	02/02/2013	-2	76.737	15.059
ct92-M305Pete-12	120362	02/02/2013	-2	76.717	15.068
ct92-M305Pete-12	120362	02/02/2013	-2	76.744	15.132
ct92-M305Pete-12	120362	02/02/2013	-1	76.731	15.109
ct92-M305Pete-12	120362	02/02/2013	-2	76.724	15.097
ct92-M305Pete-12	120362	02/02/2013	-1	76.734	15.075
ct92-M305Pete-12	120362	02/02/2013	1	76.721	15.116
ct92-M305Pete-12	120362	02/02/2013	-1	76.723	15.143
ct92-M305Pete-12	120362	02/02/2013	-1	76.718	15.148
ct92-M305Pete-12	120362	02/02/2013	-1	76.719	15.156
ct92-M305Pete-12	120362	02/02/2013	2	76.71	15.176
ct92-M305Pete-12	120362	02/02/2013	2	76.713	15.176
ct92-M305Pete-12	120362	02/02/2013	2	76.713	15.181
ct92-M305Pete-12	120362	02/02/2013	-2	76.713	15.184
ct92-M305Pete-12	120362	02/02/2013	-1	76.708	15.185
ct92-M305Pete-12	120362	02/02/2013	-1	76.687	15.319
ct92-M305Pete-12	120362	02/02/2013	3	76.685	15.29

ct92-M305Pete-12	120362	02/02/2013	1	76.704	15.296
ct92-M305Pete-12	120362	02/02/2013	1	76.684	15.248
ct92-M305Pete-12	120362	02/02/2013	1	76.699	15.255
ct92-M305Pete-12	120362	02/02/2013	-2	76.697	15.276
ct92-M305Pete-12	120362	02/02/2013	2	76.706	15.311
ct92-M305Pete-12	120362	02/02/2013	2	76.706	15.298
ct92-M305Pete-12	120362	02/02/2013	1	76.709	15.315
ct92-M305Pete-12	120362	02/02/2013	1	76.715	15.323
ct92-M305Pete-12	120362	02/02/2013	3	76.706	15.279
ct92-M305Pete-12	120362	02/02/2013	3	76.706	15.276
ct92-M305Pete-12	120362	02/02/2013	2	76.745	15.168
ct92-M305Pete-12	120362	02/02/2013	1	76.746	15.145
ct92-M305Pete-12	120362	02/02/2013	0	76.745	15.149
ct92-M305Pete-12	120362	02/02/2013	-1	76.749	15.122
ct92-M305Pete-12	120362	02/02/2013	3	76.747	15.167
ct92-M305Pete-12	120362	02/02/2013	3	76.747	15.166
ct92-M305Pete-12	120362	02/02/2013	3	76.75	15.158
ct92-M305Pete-12	120362	02/02/2013	-1	76.745	15.199
ct92-M305Pete-12	120362	02/02/2013	-1	76.759	15.159
ct92-M305Pete-12	120362	02/02/2013	-1	76.759	15.153
ct92-M305Pete-12	120362	02/02/2013	-2	76.749	15.128
ct92-M305Pete-12	120362	03/02/2013	3	76.781	15.206
ct92-M305Pete-12	120362	03/02/2013	3	76.781	15.197
ct92-M305Pete-12	120362	03/02/2013	3	76.783	15.204
ct92-M305Pete-12	120362	03/02/2013	-2	76.784	15.216
ct92-M305Pete-12	120362	03/02/2013	3	76.811	15.116
ct92-M305Pete-12	120362	03/02/2013	2	76.811	15.114
ct92-M305Pete-12	120362	03/02/2013	3	76.814	15.104
ct92-M305Pete-12	120362	03/02/2013	3	76.816	15.098
ct92-M305Pete-12	120362	03/02/2013	2	76.815	15.096
ct92-M305Pete-12	120362	03/02/2013	-2	76.813	15.099
ct92-M305Pete-12	120362	03/02/2013	0	76.821	15.084
ct92-M305Pete-12	120362	03/02/2013	3	76.801	14.961
ct92-M305Pete-12	120362	03/02/2013	3	76.799	14.949
ct92-M305Pete-12	120362	03/02/2013	3	76.794	14.941
ct92-M305Pete-12	120362	03/02/2013	2	76.793	14.939
ct92-M305Pete-12	120362	03/02/2013	-2	76.793	14.945
ct92-M305Pete-12	120362	03/02/2013	-2	76.786	14.872
ct92-M305Pete-12	120362	03/02/2013	3	76.782	14.882
ct92-M305Pete-12	120362	03/02/2013	3	76.781	14.878
ct92-M305Pete-12	120362	03/02/2013	2	76.785	14.888
ct92-M305Pete-12	120362	03/02/2013	1	76.785	14.897
ct92-M305Pete-12	120362	03/02/2013	3	76.784	14.878
ct92-M305Pete-12	120362	03/02/2013	-1	76.833	14.818
ct92-M305Pete-12	120362	03/02/2013	-2	76.794	15.024
ct92-M305Pete-12	120362	03/02/2013	-1	76.778	14.903
ct92-M305Pete-12	120362	03/02/2013	-1	76.778	14.903
ct92-M305Pete-12	120362	03/02/2013	0	76.753	14.873
ct92-M305Pete-12	120362	03/02/2013	1	76.746	14.864
ct92-M305Pete-12	120362	03/02/2013	-2	76.734	14.977
ct92-M305Pete-12	120362	03/02/2013	-2	76.755	14.881
ct92-M305Pete-12	120362	03/02/2013	2	76.733	14.861
ct92-M305Pete-12	120362	03/02/2013	-2	76.737	14.895
ct92-M305Pete-12	120362	03/02/2013	-2	76.733	14.861
ct92-M305Pete-12	120362	03/02/2013	-2	76.733	14.862
ct92-M305Pete-12	120362	03/02/2013	-2	76.733	14.862
ct92-M305Pete-12	120362	04/02/2013	-2	76.731	14.877
ct92-M305Pete-12	120362	04/02/2013	-2	76.731	14.874
ct92-M305Pete-12	120362	04/02/2013	-1	76.726	14.917
ct92-M305Pete-12	120362	04/02/2013	1	76.735	14.905
ct92-M305Pete-12	120362	04/02/2013	1	76.766	14.618
ct92-M305Pete-12	120362	04/02/2013	-2	76.766	14.629
ct92-M305Pete-12	120362	04/02/2013	-1	76.744	14.946
ct92-M305Pete-12	120362	04/02/2013	-1	76.751	15.074
ct92-M305Pete-12	120362	04/02/2013	0	76.736	15.065
ct92-M305Pete-12	120362	04/02/2013	-2	76.735	15.1
ct92-M305Pete-12	120362	04/02/2013	-2	76.734	15.1
ct92-M305Pete-12	120362	04/02/2013	-2	76.727	15.126

ct92-M305Pete-12	120362	04/02/2013	-1	76.738	15.052
ct92-M305Pete-12	120362	04/02/2013	-2	76.742	15.054
ct92-M305Pete-12	120362	04/02/2013	-2	76.743	15.061
ct92-M305Pete-12	120362	04/02/2013	-2	76.753	15.059
ct92-M305Pete-12	120362	04/02/2013	-1	76.76	15.086
ct92-M305Pete-12	120362	04/02/2013	-2	76.752	15.069
ct92-M305Pete-12	120362	04/02/2013	-2	76.742	15.064
ct92-M305Pete-12	120362	04/02/2013	-1	76.788	15.105
ct92-M305Pete-12	120362	04/02/2013	-2	76.778	15.072
ct92-M305Pete-12	120362	04/02/2013	-1	76.771	15.111
ct92-M305Pete-12	120362	04/02/2013	-1	76.781	15.151
ct92-M305Pete-12	120362	04/02/2013	-2	76.762	15.115
ct92-M305Pete-12	120362	04/02/2013	-2	76.763	15.113
ct92-M305Pete-12	120362	04/02/2013	1	76.781	15.17
ct92-M305Pete-12	120362	04/02/2013	1	76.782	15.173
ct92-M305Pete-12	120362	04/02/2013	-1	76.777	15.187
ct92-M305Pete-12	120362	04/02/2013	-2	76.765	15.144
ct92-M305Pete-12	120362	04/02/2013	0	76.784	15.22
ct92-M305Pete-12	120362	04/02/2013	-1	76.784	15.19
ct92-M305Pete-12	120362	04/02/2013	-2	76.785	15.194
ct92-M305Pete-12	120362	04/02/2013	-2	76.787	15.214
ct92-M305Pete-12	120362	04/02/2013	-2	76.789	15.209
ct92-M305Pete-12	120362	04/02/2013	-1	76.793	15.197
ct92-M305Pete-12	120362	04/02/2013	-2	76.784	15.201
ct92-M305Pete-12	120362	04/02/2013	-1	76.794	15.173
ct92-M305Pete-12	120362	04/02/2013	-2	76.792	15.162
ct92-M305Pete-12	120362	04/02/2013	-2	76.791	15.197
ct92-M305Pete-12	120362	04/02/2013	-2	76.788	15.175
ct92-M305Pete-12	120362	04/02/2013	-2	76.796	15.199
ct92-M305Pete-12	120362	04/02/2013	-2	76.8	15.193
ct92-M305Pete-12	120362	04/02/2013	-2	76.793	15.18
ct92-M305Pete-12	120362	04/02/2013	-1	76.803	15.174
ct92-M305Pete-12	120362	04/02/2013	-2	76.804	15.173
ct92-M305Pete-12	120362	04/02/2013	-2	76.809	15.177
ct92-M305Pete-12	120362	04/02/2013	-2	76.812	15.194
ct92-M305Pete-12	120362	04/02/2013	-1	76.792	15.124
ct92-M305Pete-12	120362	04/02/2013	-2	76.793	15.123
ct92-M305Pete-12	120362	04/02/2013	-1	76.797	15.108
ct92-M305Pete-12	120362	04/02/2013	-2	76.795	15.123
ct92-M305Pete-12	120362	04/02/2013	-1	76.777	15.243
ct92-M305Pete-12	120362	04/02/2013	-2	76.787	15.022
ct92-M305Pete-12	120362	04/02/2013	-2	76.785	15.047
ct92-M305Pete-12	120362	04/02/2013	-1	76.801	15.076
ct92-M305Pete-12	120362	04/02/2013	-2	76.8	15.078
ct92-M305Pete-12	120362	04/02/2013	-2	76.795	15.096
ct92-M305Pete-12	120362	04/02/2013	-2	76.796	15.095
ct92-M305Pete-12	120362	04/02/2013	-2	76.793	15.105
ct92-M305Pete-12	120362	04/02/2013	-2	76.788	15.128
ct92-M305Pete-12	120362	04/02/2013	1	76.789	15.067
ct92-M305Pete-12	120362	04/02/2013	1	76.781	15.134
ct92-M305Pete-12	120362	04/02/2013	-2	76.785	15.133
ct92-M305Pete-12	120362	04/02/2013	1	76.796	15.109
ct92-M305Pete-12	120362	05/02/2013	-2	76.787	15.124
ct92-M305Pete-12	120362	05/02/2013	-2	76.789	15.126
ct92-M305Pete-12	120362	05/02/2013	-2	76.79	15.117
ct92-M305Pete-12	120362	05/02/2013	-2	76.792	15.035
ct92-M305Pete-12	120362	05/02/2013	-2	76.809	15.205
ct92-M305Pete-12	120362	05/02/2013	-2	76.81	15.196
ct92-M305Pete-12	120362	05/02/2013	-2	76.794	15.248
ct92-M305Pete-12	120362	05/02/2013	-2	76.806	15.203
ct92-M305Pete-12	120362	05/02/2013	-1	76.824	15.082
ct92-M305Pete-12	120362	05/02/2013	-2	76.824	15.083
ct92-M305Pete-12	120362	05/02/2013	-2	76.817	15.107
ct92-M305Pete-12	120362	05/02/2013	-2	76.824	15.096
ct92-M305Pete-12	120362	05/02/2013	-1	76.826	15.126
ct92-M305Pete-12	120362	05/02/2013	-2	76.816	15.104
ct92-M305Pete-12	120362	05/02/2013	-1	76.821	15.057
ct92-M305Pete-12	120362	05/02/2013	-1	76.817	15.189

ct92-M305Pete-12	120362	05/02/2013	-2	76.813	15.076
ct92-M305Pete-12	120362	05/02/2013	-1	76.814	15.111
ct92-M305Pete-12	120362	05/02/2013	-1	76.814	15.121
ct92-M305Pete-12	120362	05/02/2013	-2	76.814	15.128
ct92-M305Pete-12	120362	05/02/2013	-2	76.814	15.128
ct92-M305Pete-12	120362	05/02/2013	-1	76.808	15.098
ct92-M305Pete-12	120362	05/02/2013	-2	76.8	15.119
ct92-M305Pete-12	120362	05/02/2013	-2	76.794	15.134
ct92-M305Pete-12	120362	05/02/2013	-2	76.795	15.131
ct92-M305Pete-12	120362	05/02/2013	-2	76.792	15.124
ct92-M305Pete-12	120362	05/02/2013	-2	76.793	15.115
ct92-M305Pete-12	120362	05/02/2013	-2	76.791	15.102
ct92-M305Pete-12	120362	05/02/2013	-2	76.794	15.109
ct92-M305Pete-12	120362	05/02/2013	-2	76.794	15.113
ct92-M305Pete-12	120362	05/02/2013	-2	76.787	15.087
ct92-M305Pete-12	120362	05/02/2013	-2	76.785	15.066
ct92-M305Pete-12	120362	05/02/2013	-2	76.771	15.078
ct92-M305Pete-12	120362	05/02/2013	-1	76.781	15.161
ct92-M305Pete-12	120362	05/02/2013	-1	76.804	15.16
ct92-M305Pete-12	120362	05/02/2013	-2	76.796	15.12
ct92-M305Pete-12	120362	05/02/2013	-1	76.783	15.085
ct92-M305Pete-12	120362	05/02/2013	-1	76.795	15.115
ct92-M305Pete-12	120362	05/02/2013	-1	76.799	15.077
ct92-M305Pete-12	120362	05/02/2013	-1	76.794	15.148
ct92-M305Pete-12	120362	05/02/2013	-1	76.798	15.107
ct92-M305Pete-12	120362	05/02/2013	-1	76.803	15.134
ct92-M305Pete-12	120362	05/02/2013	-2	76.8	15.138
ct92-M305Pete-12	120362	05/02/2013	-2	76.8	15.139
ct92-M305Pete-12	120362	05/02/2013	-2	76.791	15.158
ct92-M305Pete-12	120362	05/02/2013	-2	76.791	15.162
ct92-M305Pete-12	120362	05/02/2013	-2	76.785	15.17
ct92-M305Pete-12	120362	05/02/2013	-1	76.784	15.17
ct92-M305Pete-12	120362	05/02/2013	-1	76.79	15.204
ct92-M305Pete-12	120362	05/02/2013	-2	76.777	15.168
ct92-M305Pete-12	120362	05/02/2013	-1	76.777	15.152
ct92-M305Pete-12	120362	06/02/2013	-2	76.722	14.864
ct92-M305Pete-12	120362	06/02/2013	-2	76.734	15.133
ct92-M305Pete-12	120362	06/02/2013	1	76.75	14.939
ct92-M305Pete-12	120362	06/02/2013	2	76.754	14.917
ct92-M305Pete-12	120362	06/02/2013	2	76.772	14.871
ct92-M305Pete-12	120362	06/02/2013	-1	76.774	14.856
ct92-M305Pete-12	120362	06/02/2013	-2	76.774	14.859
ct92-M305Pete-12	120362	06/02/2013	-1	76.776	14.877
ct92-M305Pete-12	120362	06/02/2013	3	76.774	14.873
ct92-M305Pete-12	120362	06/02/2013	3	76.79	14.802
ct92-M305Pete-12	120362	06/02/2013	-2	76.79	14.798
ct92-M305Pete-12	120362	06/02/2013	3	76.792	14.797
ct92-M305Pete-12	120362	06/02/2013	-1	76.787	14.825
ct92-M305Pete-12	120362	06/02/2013	3	76.791	14.784
ct92-M305Pete-12	120362	06/02/2013	3	76.791	14.788
ct92-M305Pete-12	120362	06/02/2013	2	76.782	14.716
ct92-M305Pete-12	120362	06/02/2013	2	76.782	14.713
ct92-M305Pete-12	120362	06/02/2013	2	76.783	14.715
ct92-M305Pete-12	120362	06/02/2013	3	76.782	14.707
ct92-M305Pete-12	120362	06/02/2013	3	76.78	14.703
ct92-M305Pete-12	120362	06/02/2013	3	76.782	14.71
ct92-M305Pete-12	120362	06/02/2013	-1	76.814	14.741
ct92-M305Pete-12	120362	06/02/2013	2	76.757	14.537
ct92-M305Pete-12	120362	06/02/2013	-2	76.759	14.532
ct92-M305Pete-12	120362	06/02/2013	-2	76.767	14.524
ct92-M305Pete-12	120362	06/02/2013	-2	76.768	14.522
ct92-M305Pete-12	120362	06/02/2013	-2	76.797	14.437
ct92-M305Pete-12	120362	06/02/2013	1	76.786	14.431
ct92-M305Pete-12	120362	07/02/2013	-2	76.795	14.298
ct92-M305Pete-12	120362	07/02/2013	-2	76.788	14.238
ct92-M305Pete-12	120362	07/02/2013	-2	76.787	14.35
ct92-M305Pete-12	120362	07/02/2013	3	76.805	14.363
ct92-M305Pete-12	120362	07/02/2013	-2	76.805	14.36

ct92-M305Pete-12	120362	07/02/2013	3	76.804	14.36
ct92-M305Pete-12	120362	07/02/2013	-2	76.79	14.329
ct92-M305Pete-12	120362	07/02/2013	-2	76.861	14.265
ct92-M305Pete-12	120362	07/02/2013	-2	76.832	14.249
ct92-M305Pete-12	120362	07/02/2013	-1	76.846	14.246
ct92-M305Pete-12	120362	07/02/2013	-1	76.86	14.24
ct92-M305Pete-12	120362	07/02/2013	-1	76.878	14.214
ct92-M305Pete-12	120362	07/02/2013	-2	76.884	14.211
ct92-M305Pete-12	120362	07/02/2013	1	76.866	14.214
ct92-M305Pete-12	120362	07/02/2013	-2	76.874	14.192
ct92-M305Pete-12	120362	07/02/2013	-1	76.868	14.287
ct92-M305Pete-12	120362	07/02/2013	-1	76.911	14.31
ct92-M305Pete-12	120362	07/02/2013	-1	76.915	14.318
ct92-M305Pete-12	120362	07/02/2013	-1	76.913	14.316
ct92-M305Pete-12	120362	07/02/2013	-2	76.932	14.301
ct92-M305Pete-12	120362	07/02/2013	-2	76.933	14.306
ct92-M305Pete-12	120362	07/02/2013	-2	76.929	14.308
ct92-M305Pete-12	120362	07/02/2013	-2	76.934	14.28
ct92-M305Pete-12	120362	07/02/2013	-2	76.946	14.27
ct92-M305Pete-12	120362	07/02/2013	-2	76.942	14.267
ct92-M305Pete-12	120362	07/02/2013	-2	76.963	14.264
ct92-M305Pete-12	120362	07/02/2013	0	76.952	14.253
ct92-M305Pete-12	120362	07/02/2013	0	76.997	14.378
ct92-M305Pete-12	120362	07/02/2013	-1	77.007	14.22
ct92-M305Pete-12	120362	07/02/2013	-2	77.016	14.357
ct92-M305Pete-12	120362	07/02/2013	-1	76.991	14.299
ct92-M305Pete-12	120362	07/02/2013	-1	76.984	14.267
ct92-M305Pete-12	120362	07/02/2013	-1	77.004	14.297
ct92-M305Pete-12	120362	07/02/2013	-1	77.014	14.08
ct92-M305Pete-12	120362	07/02/2013	-1	77.008	14.025
ct92-M305Pete-12	120362	07/02/2013	-1	76.983	14.265
ct92-M305Pete-12	120362	07/02/2013	-2	76.992	14.346
ct92-M305Pete-12	120362	07/02/2013	-1	76.998	14.306
ct92-M305Pete-12	120362	07/02/2013	1	77.001	14.345
ct92-M305Pete-12	120362	07/02/2013	-1	76.998	14.365
ct92-M305Pete-12	120362	07/02/2013	-2	76.98	14.262
ct92-M305Pete-12	120362	08/02/2013	-2	76.957	14.249
ct92-M305Pete-12	120362	08/02/2013	-1	77.004	14.394
ct92-M305Pete-12	120362	08/02/2013	-2	76.968	14.207
ct92-M305Pete-12	120362	08/02/2013	-2	76.988	14.346
ct92-M305Pete-12	120362	08/02/2013	-2	76.969	14.207
ct92-M305Pete-12	120362	08/02/2013	-1	76.977	14.264
ct92-M305Pete-12	120362	08/02/2013	-2	76.985	14.268
ct92-M305Pete-12	120362	08/02/2013	-2	76.988	14.258
ct92-M305Pete-12	120362	08/02/2013	-2	76.996	14.283
ct92-M305Pete-12	120362	08/02/2013	-2	76.997	14.251
ct92-M305Pete-12	120362	08/02/2013	-2	77.003	14.291
ct92-M305Pete-12	120362	08/02/2013	-2	77.004	14.284
ct92-M305Pete-12	120362	08/02/2013	-2	77.007	14.285
ct92-M305Pete-12	120362	08/02/2013	-2	77.018	14.275
ct92-M305Pete-12	120362	08/02/2013	-1	77.037	14.241
ct92-M305Pete-12	120362	08/02/2013	-2	77.008	14.263
ct92-M305Pete-12	120362	08/02/2013	-2	77.027	14.283
ct92-M305Pete-12	120362	08/02/2013	-2	77.034	14.184
ct92-M305Pete-12	120362	08/02/2013	-2	77.035	14.188
ct92-M305Pete-12	120362	08/02/2013	-1	77.055	14.205
ct92-M305Pete-12	120362	08/02/2013	-1	77.059	14.15
ct92-M305Pete-12	120362	08/02/2013	-1	77.076	14.137
ct92-M305Pete-12	120362	08/02/2013	-2	77.079	14.1
ct92-M305Pete-12	120362	08/02/2013	-1	77.088	14.157
ct92-M305Pete-12	120362	08/02/2013	-2	77.097	14.129
ct92-M305Pete-12	120362	08/02/2013	-2	77.093	14.172
ct92-M305Pete-12	120362	08/02/2013	-2	77.095	14.165
ct92-M305Pete-12	120362	08/02/2013	-2	77.098	14.162
ct92-M305Pete-12	120362	08/02/2013	-2	77.099	14.145
ct92-M305Pete-12	120362	08/02/2013	-1	77.096	14.143
ct92-M305Pete-12	120362	08/02/2013	-1	77.099	14.094
ct92-M305Pete-12	120362	08/02/2013	-1	77.114	14.121

ct92-M305Pete-12	120362	08/02/2013	-1	77.116	14.085
ct92-M305Pete-12	120362	08/02/2013	-2	77.118	14.133
ct92-M305Pete-12	120362	08/02/2013	-2	77.119	14.118
ct92-M305Pete-12	120362	08/02/2013	-2	77.122	14.11
ct92-M305Pete-12	120362	08/02/2013	-1	77.137	14.039
ct92-M305Pete-12	120362	08/02/2013	-1	77.129	14.082
ct92-M305Pete-12	120362	08/02/2013	-2	77.131	14.057
ct92-M305Pete-12	120362	08/02/2013	-2	77.121	13.979
ct92-M305Pete-12	120362	08/02/2013	-2	77.129	14.081
ct92-M305Pete-12	120362	08/02/2013	-2	77.144	14.082
ct92-M305Pete-12	120362	08/02/2013	-1	77.146	14.069
ct92-M305Pete-12	120362	08/02/2013	-2	77.141	14.12
ct92-M305Pete-12	120362	08/02/2013	-2	77.169	14.084
ct92-M305Pete-12	120362	08/02/2013	-1	77.151	14.115
ct92-M305Pete-12	120362	08/02/2013	-2	77.15	14.136
ct92-M305Pete-12	120362	08/02/2013	-2	77.156	14.117
ct92-M305Pete-12	120362	08/02/2013	-2	77.159	14.096
ct92-M305Pete-12	120362	08/02/2013	0	77.162	14.048
ct92-M305Pete-12	120362	08/02/2013	1	77.156	14.056
ct92-M305Pete-12	120362	08/02/2013	1	77.156	14.055
ct92-M305Pete-12	120362	08/02/2013	1	77.17	14.045
ct92-M305Pete-12	120362	08/02/2013	1	77.189	14.029
ct92-M305Pete-12	120362	08/02/2013	1	77.181	13.983
ct92-M305Pete-12	120362	08/02/2013	3	77.172	14.097
ct92-M305Pete-12	120362	08/02/2013	-2	77.179	14.072
ct92-M305Pete-12	120362	08/02/2013	3	77.173	14.089
ct92-M305Pete-12	120362	09/02/2013	-2	77.194	14.068
ct92-M305Pete-12	120362	09/02/2013	3	77.192	14.088
ct92-M305Pete-12	120362	09/02/2013	2	77.19	14.12
ct92-M305Pete-12	120362	09/02/2013	-2	77.188	14.11
ct92-M305Pete-12	120362	09/02/2013	3	77.201	14.092
ct92-M305Pete-12	120362	09/02/2013	-2	77.225	13.937
ct92-M305Pete-12	120362	09/02/2013	-2	77.217	13.975
ct92-M305Pete-12	120362	09/02/2013	-2	77.221	13.996
ct92-M305Pete-12	120362	09/02/2013	-1	77.209	13.99
ct92-M305Pete-12	120362	09/02/2013	-2	77.23	13.943
ct92-M305Pete-12	120362	09/02/2013	-2	77.223	13.972
ct92-M305Pete-12	120362	09/02/2013	-2	77.222	13.971
ct92-M305Pete-12	120362	09/02/2013	-1	77.228	13.887
ct92-M305Pete-12	120362	09/02/2013	-2	77.229	13.869
ct92-M305Pete-12	120362	09/02/2013	-2	77.234	13.821
ct92-M305Pete-12	120362	09/02/2013	-2	77.228	13.789
ct92-M305Pete-12	120362	09/02/2013	-1	77.225	13.766
ct92-M305Pete-12	120362	09/02/2013	-2	77.225	13.768
ct92-M305Pete-12	120362	09/02/2013	-2	77.225	13.763
ct92-M305Pete-12	120362	09/02/2013	-2	77.224	13.75
ct92-M305Pete-12	120362	09/02/2013	-1	77.236	13.766
ct92-M305Pete-12	120362	09/02/2013	-2	77.242	13.732
ct92-M305Pete-12	120362	09/02/2013	-2	77.247	13.749
ct92-M305Pete-12	120362	09/02/2013	-2	77.247	13.749
ct92-M305Pete-12	120362	09/02/2013	-2	77.238	13.791
ct92-M305Pete-12	120362	09/02/2013	-2	77.238	13.789
ct92-M305Pete-12	120362	09/02/2013	-1	77.233	13.777
ct92-M305Pete-12	120362	09/02/2013	-1	77.225	13.747
ct92-M305Pete-12	120362	09/02/2013	-1	77.23	13.766
ct92-M305Pete-12	120362	09/02/2013	-2	77.233	13.749
ct92-M305Pete-12	120362	09/02/2013	-1	77.234	13.753
ct92-M305Pete-12	120362	09/02/2013	-2	77.235	13.766
ct92-M305Pete-12	120362	09/02/2013	-2	77.233	13.77
ct92-M305Pete-12	120362	09/02/2013	-2	77.233	13.767
ct92-M305Pete-12	120362	09/02/2013	-2	77.237	13.738
ct92-M305Pete-12	120362	09/02/2013	-1	77.236	13.731
ct92-M305Pete-12	120362	09/02/2013	-1	77.227	13.796
ct92-M305Pete-12	120362	09/02/2013	-2	77.231	13.862
ct92-M305Pete-12	120362	09/02/2013	-2	77.248	13.717
ct92-M305Pete-12	120362	09/02/2013	-2	77.235	13.757
ct92-M305Pete-12	120362	09/02/2013	-1	77.242	13.756
ct92-M305Pete-12	120362	09/02/2013	-2	77.244	13.758

ct92-M305Pete-12	120362	09/02/2013	-2	77.238	13.775
ct92-M305Pete-12	120362	09/02/2013	-2	77.245	13.739
ct92-M305Pete-12	120362	09/02/2013	-2	77.236	13.725
ct92-M305Pete-12	120362	09/02/2013	-2	77.236	13.74
ct92-M305Pete-12	120362	09/02/2013	0	77.249	13.696
ct92-M305Pete-12	120362	09/02/2013	1	77.25	13.81
ct92-M305Pete-12	120362	09/02/2013	1	77.244	13.753
ct92-M305Pete-12	120362	09/02/2013	-2	77.246	13.725
ct92-M305Pete-12	120362	09/02/2013	-2	77.258	13.72
ct92-M305Pete-12	120362	09/02/2013	-2	77.257	13.722
ct92-M305Pete-12	120362	09/02/2013	-2	77.245	13.722
ct92-M305Pete-12	120362	10/02/2013	-2	77.245	13.711
ct92-M305Pete-12	120362	10/02/2013	-2	77.224	13.829
ct92-M305Pete-12	120362	10/02/2013	-2	77.224	13.801
ct92-M305Pete-12	120362	10/02/2013	-1	77.254	13.794
ct92-M305Pete-12	120362	10/02/2013	-2	77.257	13.783
ct92-M305Pete-12	120362	10/02/2013	-2	77.267	13.799
ct92-M305Pete-12	120362	10/02/2013	-2	77.269	13.793
ct92-M305Pete-12	120362	10/02/2013	-2	77.332	13.803
ct92-M305Pete-12	120362	10/02/2013	-2	77.345	13.935
ct92-M305Pete-12	120362	10/02/2013	-2	77.345	13.935
ct92-M305Pete-12	120362	10/02/2013	-2	77.353	13.953
ct92-M305Pete-12	120362	10/02/2013	-2	77.351	13.965
ct92-M305Pete-12	120362	10/02/2013	-2	77.36	13.976
ct92-M305Pete-12	120362	10/02/2013	-2	77.378	13.936
ct92-M305Pete-12	120362	10/02/2013	-2	77.366	14.004
ct92-M305Pete-12	120362	10/02/2013	-2	77.361	13.846
ct92-M305Pete-12	120362	10/02/2013	-2	77.376	13.853
ct92-M305Pete-12	120362	10/02/2013	-2	77.368	13.861
ct92-M305Pete-12	120362	10/02/2013	-2	77.389	13.759
ct92-M305Pete-12	120362	10/02/2013	-2	77.395	13.781
ct92-M305Pete-12	120362	10/02/2013	-2	77.389	13.822
ct92-M305Pete-12	120362	10/02/2013	-2	77.393	13.828
ct92-M305Pete-12	120362	10/02/2013	-2	77.416	13.853
ct92-M305Pete-12	120362	10/02/2013	-2	77.429	13.792
ct92-M305Pete-12	120362	10/02/2013	-2	77.394	13.831
ct92-M305Pete-12	120362	10/02/2013	-2	77.395	13.855
ct92-M305Pete-12	120362	10/02/2013	-2	77.409	13.82
ct92-M305Pete-12	120362	10/02/2013	-2	77.416	13.822
ct92-M305Pete-12	120362	10/02/2013	-1	77.44	13.799
ct92-M305Pete-12	120362	10/02/2013	-2	77.454	13.82
ct92-M305Pete-12	120362	10/02/2013	-2	77.449	13.821
ct92-M305Pete-12	120362	10/02/2013	-2	77.449	13.822
ct92-M305Pete-12	120362	10/02/2013	-2	77.447	13.88
ct92-M305Pete-12	120362	10/02/2013	-2	77.458	13.892
ct92-M305Pete-12	120362	10/02/2013	-2	77.453	13.903
ct92-M305Pete-12	120362	10/02/2013	-2	77.452	13.903
ct92-M305Pete-12	120362	10/02/2013	-2	77.451	13.892
ct92-M305Pete-12	120362	10/02/2013	-2	77.464	13.836
ct92-M305Pete-12	120362	10/02/2013	-2	77.475	13.873
ct92-M305Pete-12	120362	10/02/2013	-2	77.481	13.865
ct92-M305Pete-12	120362	10/02/2013	-2	77.495	13.901
ct92-M305Pete-12	120362	10/02/2013	-2	77.484	13.761
ct92-M305Pete-12	120362	10/02/2013	-2	77.52	13.852
ct92-M305Pete-12	120362	10/02/2013	-2	77.528	13.862
ct92-M305Pete-12	120362	10/02/2013	-2	77.529	13.862
ct92-M305Pete-12	120362	10/02/2013	-2	77.546	13.947
ct92-M305Pete-12	120362	10/02/2013	-2	77.547	13.936
ct92-M305Pete-12	120362	10/02/2013	-2	77.522	14.001
ct92-M305Pete-12	120362	11/02/2013	-2	77.531	13.995
ct92-M305Pete-12	120362	11/02/2013	-2	77.552	14.05
ct92-M305Pete-12	120362	11/02/2013	-2	77.55	14.036
ct92-M305Pete-12	120362	11/02/2013	-2	77.558	14.048
ct92-M305Pete-12	120362	11/02/2013	-2	77.562	13.984
ct92-M305Pete-12	120362	11/02/2013	-2	77.567	14.076
ct92-M305Pete-12	120362	11/02/2013	-2	77.569	14.007
ct92-M305Pete-12	120362	11/02/2013	-2	77.573	14.025
ct92-M305Pete-12	120362	11/02/2013	-2	77.583	14.137

ct92-M305Pete-12	120362	11/02/2013	-2	77.575	14.102
ct92-M305Pete-12	120362	11/02/2013	-2	77.6	14.036
ct92-M305Pete-12	120362	11/02/2013	-2	77.681	14.011
ct92-M305Pete-12	120362	11/02/2013	-2	77.679	13.991
ct92-M305Pete-12	120362	11/02/2013	-2	77.677	13.967
ct92-M305Pete-12	120362	11/02/2013	-2	77.686	14.029
ct92-M305Pete-12	120362	11/02/2013	-2	77.683	13.986
ct92-M305Pete-12	120362	11/02/2013	-2	77.705	13.994
ct92-M305Pete-12	120362	11/02/2013	-2	77.716	13.977
ct92-M305Pete-12	120362	11/02/2013	-2	77.727	13.916
ct92-M305Pete-12	120362	11/02/2013	-2	77.722	13.91
ct92-M305Pete-12	120362	11/02/2013	-2	77.742	13.997
ct92-M305Pete-12	120362	11/02/2013	-2	77.731	13.992
ct92-M305Pete-12	120362	11/02/2013	-2	77.756	13.782
ct92-M305Pete-12	120362	11/02/2013	-2	77.726	13.739
ct92-M305Pete-12	120362	11/02/2013	-2	77.753	13.899
ct92-M305Pete-12	120362	11/02/2013	-2	77.724	13.743
ct92-M305Pete-12	120362	11/02/2013	-2	77.742	13.666
ct92-M305Pete-12	120362	11/02/2013	-2	77.756	13.715
ct92-M305Pete-12	120362	11/02/2013	-2	77.771	13.78
ct92-M305Pete-12	120362	11/02/2013	-2	77.739	13.686
ct92-M305Pete-12	120362	11/02/2013	-2	77.75	13.72
ct92-M305Pete-12	120362	11/02/2013	-1	77.737	13.698
ct92-M305Pete-12	120362	11/02/2013	-2	77.751	13.668
ct92-M305Pete-12	120362	11/02/2013	-2	77.758	13.642
ct92-M305Pete-12	120362	11/02/2013	-2	77.755	13.65
ct92-M305Pete-12	120362	11/02/2013	-2	77.759	13.661
ct92-M305Pete-12	120362	11/02/2013	-1	77.796	13.691
ct92-M305Pete-12	120362	11/02/2013	-2	77.819	13.64
ct92-M305Pete-12	120362	11/02/2013	-2	77.829	13.615
ct92-M305Pete-12	120362	11/02/2013	-2	77.782	13.69
ct92-M305Pete-12	120362	11/02/2013	-2	77.782	13.684
ct92-M305Pete-12	120362	11/02/2013	-2	77.806	13.619
ct92-M305Pete-12	120362	11/02/2013	-2	77.814	13.606
ct92-M305Pete-12	120362	11/02/2013	-2	77.793	13.614
ct92-M305Pete-12	120362	11/02/2013	-2	77.795	13.606
ct92-M305Pete-12	120362	11/02/2013	-2	77.809	13.565
ct92-M305Pete-12	120362	11/02/2013	-2	77.826	13.606
ct92-M305Pete-12	120362	11/02/2013	-2	77.837	13.616
ct92-M305Pete-12	120362	11/02/2013	-2	77.835	13.624
ct92-M305Pete-12	120362	11/02/2013	-2	77.826	13.653
ct92-M305Pete-12	120362	11/02/2013	-2	77.825	13.66
ct92-M305Pete-12	120362	11/02/2013	-2	77.832	13.653
ct92-M305Pete-12	120362	11/02/2013	-2	77.848	13.483
ct92-M305Pete-12	120362	11/02/2013	-2	77.832	13.333
ct92-M305Pete-12	120362	11/02/2013	-2	77.891	13.223
ct92-M305Pete-12	120362	11/02/2013	-2	77.871	13.634
ct92-M305Pete-12	120362	11/02/2013	-2	77.861	13.304
ct92-M305Pete-12	120362	11/02/2013	-2	77.87	13.268
ct92-M305Pete-12	120362	11/02/2013	-2	77.877	13.263
ct92-M305Pete-12	120362	11/02/2013	-2	77.881	13.257
ct92-M305Pete-12	120362	11/02/2013	-2	77.885	13.322
ct92-M305Pete-12	120362	11/02/2013	-2	77.89	13.316
ct92-M305Pete-12	120362	12/02/2013	-2	77.896	13.288
ct92-M305Pete-12	120362	12/02/2013	-2	77.9	13.481
ct92-M305Pete-12	120362	12/02/2013	-2	77.898	13.493
ct92-M305Pete-12	120362	12/02/2013	-1	77.948	13.356
ct92-M305Pete-12	120362	12/02/2013	-2	77.955	13.347
ct92-M305Pete-12	120362	12/02/2013	-2	77.94	13.369
ct92-M305Pete-12	120362	12/02/2013	-2	77.966	13.296
ct92-M305Pete-12	120362	12/02/2013	-1	77.968	13.373
ct92-M305Pete-12	120362	12/02/2013	-2	77.97	13.375
ct92-M305Pete-12	120362	12/02/2013	-2	77.991	13.341
ct92-M305Pete-12	120362	12/02/2013	-2	77.995	13.335
ct92-M305Pete-12	120362	12/02/2013	-2	78.013	13.309
ct92-M305Pete-12	120362	12/02/2013	-2	78.028	13.321
ct92-M305Pete-12	120362	12/02/2013	-2	78.034	13.3
ct92-M305Pete-12	120362	12/02/2013	-2	78.046	13.303

ct92-M305Pete-12	120362	12/02/2013	-2	78.055	13.286
ct92-M305Pete-12	120362	12/02/2013	-2	78.052	13.287
ct92-M305Pete-12	120362	12/02/2013	-2	78.061	13.273
ct92-M305Pete-12	120362	12/02/2013	-2	78.055	13.275
ct92-M305Pete-12	120362	12/02/2013	-2	78.049	13.242
ct92-M305Pete-12	120362	12/02/2013	-2	78.061	13.249
ct92-M305Pete-12	120362	12/02/2013	-2	78.089	13.233
ct92-M305Pete-12	120362	12/02/2013	-2	78.077	13.247
ct92-M305Pete-12	120362	12/02/2013	-2	78.078	13.247
ct92-M305Pete-12	120362	12/02/2013	-2	78.098	13.217
ct92-M305Pete-12	120362	12/02/2013	-2	78.125	13.215
ct92-M305Pete-12	120362	12/02/2013	-2	78.123	13.249
ct92-M305Pete-12	120362	12/02/2013	-2	78.138	13.225
ct92-M305Pete-12	120362	12/02/2013	-2	78.196	12.974
ct92-M305Pete-12	120362	12/02/2013	2	78.205	12.954
ct92-M305Pete-12	120362	12/02/2013	2	78.2	12.943
ct92-M305Pete-12	120362	12/02/2013	2	78.207	12.968
ct92-M305Pete-12	120362	12/02/2013	-2	78.232	12.906
ct92-M305Pete-12	120362	12/02/2013	-2	78.22	12.922
ct92-M305Pete-12	120362	12/02/2013	-2	78.203	12.962
ct92-M305Pete-12	120362	12/02/2013	1	78.223	12.975
ct92-M305Pete-12	120362	12/02/2013	-2	78.184	12.948
ct92-M305Pete-12	120362	12/02/2013	-2	78.203	12.91
ct92-M305Pete-12	120362	12/02/2013	-2	78.207	12.897
ct92-M305Pete-12	120362	12/02/2013	-2	78.204	12.894
ct92-M305Pete-12	120362	12/02/2013	-2	78.232	12.82
ct92-M305Pete-12	120362	12/02/2013	-2	78.221	12.823
ct92-M305Pete-12	120362	12/02/2013	-2	78.244	12.764
ct92-M305Pete-12	120362	12/02/2013	-2	78.29	12.594
ct92-M305Pete-12	120362	12/02/2013	-2	78.224	12.568
ct92-M305Pete-12	120362	12/02/2013	-2	78.303	12.251
ct92-M305Pete-12	120362	12/02/2013	-2	78.295	12.39
ct92-M305Pete-12	120362	12/02/2013	-2	78.277	12.452
ct92-M305Pete-12	120362	13/02/2013	-2	78.297	11.931
ct92-M305Pete-12	120362	13/02/2013	-2	78.283	12.331
ct92-M305Pete-12	120362	13/02/2013	-2	78.288	12.201
ct92-M305Pete-12	120362	13/02/2013	-2	78.316	12.023
ct92-M305Pete-12	120362	13/02/2013	-2	78.345	12.001
ct92-M305Pete-12	120362	13/02/2013	-2	78.351	11.884
ct92-M305Pete-12	120362	13/02/2013	-2	78.355	11.87
ct92-M305Pete-12	120362	13/02/2013	-2	78.356	11.868
ct92-M305Pete-12	120362	13/02/2013	-2	78.358	12.065
ct92-M305Pete-12	120362	13/02/2013	-2	78.372	11.896
ct92-M305Pete-12	120362	13/02/2013	-2	78.372	11.876
ct92-M305Pete-12	120362	13/02/2013	-2	78.379	11.971
ct92-M305Pete-12	120362	13/02/2013	-2	78.388	11.938
ct92-M305Pete-12	120362	13/02/2013	-2	78.386	11.955
ct92-M305Pete-12	120362	13/02/2013	-2	78.394	11.882
ct92-M305Pete-12	120362	13/02/2013	-2	78.39	11.91
ct92-M305Pete-12	120362	13/02/2013	-2	78.393	11.906
ct92-M305Pete-12	120362	13/02/2013	-2	78.405	11.883
ct92-M305Pete-12	120362	13/02/2013	-2	78.415	11.829
ct92-M305Pete-12	120362	13/02/2013	-2	78.399	11.906
ct92-M305Pete-12	120362	13/02/2013	-2	78.296	12.186
ct92-M305Pete-12	120362	13/02/2013	-2	78.461	11.797
ct92-M305Pete-12	120362	13/02/2013	-2	78.427	11.698
ct92-M305Pete-12	120362	13/02/2013	-2	78.46	11.824
ct92-M305Pete-12	120362	13/02/2013	-2	78.464	11.808
ct92-M305Pete-12	120362	13/02/2013	-2	78.482	11.752
ct92-M305Pete-12	120362	13/02/2013	-2	78.488	11.768
ct92-M305Pete-12	120362	13/02/2013	-2	78.496	11.758
ct92-M305Pete-12	120362	13/02/2013	-2	78.498	11.731
ct92-M305Pete-12	120362	13/02/2013	-2	78.499	11.738
ct92-M305Pete-12	120362	13/02/2013	-2	78.502	11.725
ct92-M305Pete-12	120362	13/02/2013	-2	78.512	11.689
ct92-M305Pete-12	120362	13/02/2013	-2	78.527	11.689
ct92-M305Pete-12	120362	13/02/2013	-2	78.528	11.689
ct92-M305Pete-12	120362	13/02/2013	-2	78.53	11.68

ct92-M305Pete-12	120362	13/02/2013	-2	78.539	11.722
ct92-M305Pete-12	120362	13/02/2013	-2	78.548	11.693
ct92-M305Pete-12	120362	13/02/2013	-1	78.544	11.711
ct92-M305Pete-12	120362	13/02/2013	-2	78.551	11.691
ct92-M305Pete-12	120362	13/02/2013	-2	78.566	11.668
ct92-M305Pete-12	120362	13/02/2013	-2	78.567	11.621
ct92-M305Pete-12	120362	13/02/2013	-2	78.567	11.668
ct92-M305Pete-12	120362	13/02/2013	-2	78.58	11.625
ct92-M305Pete-12	120362	13/02/2013	-2	78.591	11.599
ct92-M305Pete-12	120362	13/02/2013	-2	78.594	11.602
ct92-M305Pete-12	120362	13/02/2013	-2	78.599	11.56
ct92-M305Pete-12	120362	13/02/2013	-2	78.606	11.539
ct92-M305Pete-12	120362	13/02/2013	-2	78.608	11.533
ct92-M305Pete-12	120362	13/02/2013	-2	78.617	11.518
ct92-M305Pete-12	120362	13/02/2013	-2	78.615	11.53
ct92-M305Pete-12	120362	13/02/2013	-2	78.633	11.484
ct92-M305Pete-12	120362	13/02/2013	-2	78.627	11.499
ct92-M305Pete-12	120362	13/02/2013	-2	78.64	11.473
ct92-M305Pete-12	120362	13/02/2013	-2	78.649	11.44
ct92-M305Pete-12	120362	13/02/2013	-2	78.66	11.369
ct92-M305Pete-12	120362	13/02/2013	-2	78.682	11.296
ct92-M305Pete-12	120362	13/02/2013	-2	78.676	11.281
ct92-M305Pete-12	120362	13/02/2013	-2	78.688	11.328
ct92-M305Pete-12	120362	13/02/2013	-2	78.718	11.265
ct92-M305Pete-12	120362	13/02/2013	-2	78.727	11.299
ct92-M305Pete-12	120362	13/02/2013	-2	78.732	11.301
ct92-M305Pete-12	120362	13/02/2013	-2	78.715	11.283
ct92-M305Pete-12	120362	13/02/2013	-2	78.746	11.217
ct92-M305Pete-12	120362	13/02/2013	-2	78.749	11.223
ct92-M305Pete-12	120362	13/02/2013	-2	78.736	11.231
ct92-M305Pete-12	120362	13/02/2013	-2	78.757	11.187
ct92-M305Pete-12	120362	13/02/2013	-2	78.769	11.148
ct92-M305Pete-12	120362	13/02/2013	-2	78.757	11.184
ct92-M305Pete-12	120362	13/02/2013	-2	78.801	11.153
ct92-M305Pete-12	120362	13/02/2013	-2	78.793	11.104
ct92-M305Pete-12	120362	13/02/2013	-2	78.792	11.113
ct92-M305Pete-12	120362	13/02/2013	-2	78.791	11.147
ct92-M305Pete-12	120362	13/02/2013	-2	78.818	11.099
ct92-M305Pete-12	120362	13/02/2013	-2	78.803	11.113
ct92-M305Pete-12	120362	13/02/2013	-2	78.8	11.134
ct92-M305Pete-12	120362	14/02/2013	-2	78.831	10.99
ct92-M305Pete-12	120362	14/02/2013	-2	78.807	10.993
ct92-M305Pete-12	120362	14/02/2013	-2	78.813	10.975
ct92-M305Pete-12	120362	14/02/2013	-2	78.831	11.019
ct92-M305Pete-12	120362	14/02/2013	-2	78.839	10.975
ct92-M305Pete-12	120362	14/02/2013	-2	78.842	10.957
ct92-M305Pete-12	120362	14/02/2013	-2	78.838	11.035
ct92-M305Pete-12	120362	14/02/2013	-2	78.862	10.913
ct92-M305Pete-12	120362	14/02/2013	-2	78.863	10.977
ct92-M305Pete-12	120362	14/02/2013	-2	78.863	10.98
ct92-M305Pete-12	120362	14/02/2013	-2	78.863	11.05
ct92-M305Pete-12	120362	14/02/2013	-2	78.888	10.783
ct92-M305Pete-12	120362	14/02/2013	-2	78.897	10.761
ct92-M305Pete-12	120362	14/02/2013	-2	78.907	10.718
ct92-M305Pete-12	120362	14/02/2013	-2	78.914	10.655
ct92-M305Pete-12	120362	14/02/2013	-2	78.903	10.686
ct92-M305Pete-12	120362	14/02/2013	-2	78.907	10.701
ct92-M305Pete-12	120362	14/02/2013	-2	78.947	10.661
ct92-M305Pete-12	120362	14/02/2013	-2	78.907	10.806
ct92-M305Pete-12	120362	14/02/2013	-2	78.934	10.539
ct92-M305Pete-12	120362	14/02/2013	-2	78.916	10.717
ct92-M305Pete-12	120362	14/02/2013	-2	78.927	10.824
ct92-M305Pete-12	120362	14/02/2013	-1	78.957	10.654
ct92-M305Pete-12	120362	14/02/2013	-2	78.983	10.703
ct92-M305Pete-12	120362	14/02/2013	-2	78.946	10.791
ct92-M305Pete-12	120362	14/02/2013	-2	78.946	10.792
ct92-M305Pete-12	120362	14/02/2013	-2	78.935	10.784
ct92-M305Pete-12	120362	14/02/2013	-2	78.946	10.76

ct92-M305Pete-12	120362	14/02/2013	-2	78.942	10.753
ct92-M305Pete-12	120362	14/02/2013	-2	78.942	10.75
ct92-M305Pete-12	120362	14/02/2013	-2	78.948	10.741
ct92-M305Pete-12	120362	14/02/2013	-2	78.946	10.745
ct92-M305Pete-12	120362	14/02/2013	-2	78.957	10.725
ct92-M305Pete-12	120362	14/02/2013	-2	78.943	10.637
ct92-M305Pete-12	120362	14/02/2013	-2	78.943	10.621
ct92-M305Pete-12	120362	14/02/2013	-2	78.922	10.6
ct92-M305Pete-12	120362	14/02/2013	-2	78.94	10.63
ct92-M305Pete-12	120362	14/02/2013	-2	78.942	10.593
ct92-M305Pete-12	120362	14/02/2013	-1	78.923	10.617
ct92-M305Pete-12	120362	14/02/2013	-2	78.913	10.644
ct92-M305Pete-12	120362	14/02/2013	-2	78.932	10.576
ct92-M305Pete-12	120362	14/02/2013	-2	78.966	10.559
ct92-M305Pete-12	120362	14/02/2013	-2	78.93	10.584
ct92-M305Pete-12	120362	14/02/2013	-2	78.943	10.607
ct92-M305Pete-12	120362	14/02/2013	-2	78.914	10.523
ct92-M305Pete-12	120362	14/02/2013	-2	78.925	10.483
ct92-M305Pete-12	120362	14/02/2013	-2	78.928	10.571
ct92-M305Pete-12	120362	14/02/2013	-2	78.927	10.498
ct92-M305Pete-12	120362	14/02/2013	-2	78.929	10.488
ct92-M305Pete-12	120362	14/02/2013	-2	78.929	10.503
ct92-M305Pete-12	120362	14/02/2013	-1	78.914	10.436
ct92-M305Pete-12	120362	14/02/2013	-2	78.949	10.459
ct92-M305Pete-12	120362	14/02/2013	-2	78.915	10.401
ct92-M305Pete-12	120362	14/02/2013	2	78.916	10.457
ct92-M305Pete-12	120362	14/02/2013	3	78.913	10.46
ct92-M305Pete-12	120362	14/02/2013	-2	78.915	10.453
ct92-M305Pete-12	120362	14/02/2013	-2	78.92	10.308
ct92-M305Pete-12	120362	14/02/2013	-2	78.918	10.369
ct92-M305Pete-12	120362	14/02/2013	-2	78.919	10.352
ct92-M305Pete-12	120362	14/02/2013	-2	78.918	10.33
ct92-M305Pete-12	120362	14/02/2013	-1	78.907	10.639
ct92-M305Pete-12	120362	14/02/2013	-2	78.919	10.327
ct92-M305Pete-12	120362	15/02/2013	-2	78.907	10.614
ct92-M305Pete-12	120362	15/02/2013	-2	78.907	10.366
ct92-M305Pete-12	120362	15/02/2013	-2	78.911	10.487
ct92-M305Pete-12	120362	15/02/2013	-2	78.911	10.49
ct92-M305Pete-12	120362	15/02/2013	-2	78.91	10.645
ct92-M305Pete-12	120362	15/02/2013	-2	78.918	10.546
ct92-M305Pete-12	120362	15/02/2013	-2	78.919	10.582
ct92-M305Pete-12	120362	15/02/2013	-2	78.919	10.583
ct92-M305Pete-12	120362	15/02/2013	-2	78.92	10.579
ct92-M305Pete-12	120362	15/02/2013	-2	78.915	10.551
ct92-M305Pete-12	120362	15/02/2013	-2	78.899	10.659
ct92-M305Pete-12	120362	15/02/2013	-2	78.91	10.564
ct92-M305Pete-12	120362	15/02/2013	-2	78.91	10.569
ct92-M305Pete-12	120362	15/02/2013	-2	78.91	10.568
ct92-M305Pete-12	120362	15/02/2013	-2	78.91	10.612
ct92-M305Pete-12	120362	15/02/2013	-2	78.929	10.614
ct92-M305Pete-12	120362	15/02/2013	-2	78.907	10.619
ct92-M305Pete-12	120362	15/02/2013	-2	78.904	10.624
ct92-M305Pete-12	120362	15/02/2013	-1	78.905	10.622
ct92-M305Pete-12	120362	15/02/2013	-2	78.907	10.626
ct92-M305Pete-12	120362	15/02/2013	-2	78.91	10.648
ct92-M305Pete-12	120362	15/02/2013	-2	78.91	10.648
ct92-M305Pete-12	120362	15/02/2013	-2	78.951	10.599
ct92-M305Pete-12	120362	15/02/2013	-2	78.901	10.656
ct92-M305Pete-12	120362	15/02/2013	-2	78.893	10.679
ct92-M305Pete-12	120362	15/02/2013	-2	78.893	10.681
ct92-M305Pete-12	120362	15/02/2013	-2	78.896	10.68
ct92-M305Pete-12	120362	15/02/2013	-2	78.901	10.666
ct92-M305Pete-12	120362	15/02/2013	-2	78.902	10.649
ct92-M305Pete-12	120362	15/02/2013	-2	78.9	10.666
ct92-M305Pete-12	120362	15/02/2013	-2	78.913	10.625
ct92-M305Pete-12	120362	15/02/2013	-2	78.911	10.611
ct92-M305Pete-12	120362	15/02/2013	-2	78.913	10.623
ct92-M305Pete-12	120362	15/02/2013	-2	78.909	10.605

ct92-M305Pete-12	120362	15/02/2013	-1	78.917	10.573
ct92-M305Pete-12	120362	15/02/2013	-2	78.912	10.633
ct92-M305Pete-12	120362	15/02/2013	-2	78.913	10.576
ct92-M305Pete-12	120362	15/02/2013	-2	78.917	10.628
ct92-M305Pete-12	120362	15/02/2013	-2	78.917	10.636
ct92-M305Pete-12	120362	15/02/2013	-1	78.906	10.705
ct92-M305Pete-12	120362	15/02/2013	0	78.9	10.704
ct92-M305Pete-12	120362	15/02/2013	-2	78.9	10.705
ct92-M305Pete-12	120362	15/02/2013	-1	78.93	10.634
ct92-M305Pete-12	120362	15/02/2013	-2	78.907	10.691
ct92-M305Pete-12	120362	15/02/2013	-2	78.912	10.642
ct92-M305Pete-12	120362	15/02/2013	-2	78.913	10.679
ct92-M305Pete-12	120362	15/02/2013	-2	78.913	10.679
ct92-M305Pete-12	120362	15/02/2013	-2	78.917	10.656
ct92-M305Pete-12	120362	15/02/2013	-2	78.905	10.568
ct92-M305Pete-12	120362	15/02/2013	-2	78.908	10.562
ct92-M305Pete-12	120362	15/02/2013	-2	78.908	10.579
ct92-M305Pete-12	120362	15/02/2013	-1	78.92	10.528
ct92-M305Pete-12	120362	15/02/2013	-2	78.902	10.506
ct92-M305Pete-12	120362	15/02/2013	-2	78.903	10.505
ct92-M305Pete-12	120362	15/02/2013	-2	78.902	10.514
ct92-M305Pete-12	120362	15/02/2013	-2	78.902	10.489
ct92-M305Pete-12	120362	15/02/2013	-2	78.901	10.517
ct92-M305Pete-12	120362	15/02/2013	-2	78.903	10.558
ct92-M305Pete-12	120362	15/02/2013	-2	78.893	10.543
ct92-M305Pete-12	120362	15/02/2013	-2	78.903	10.524
ct92-M305Pete-12	120362	15/02/2013	-1	78.901	10.536
ct92-M305Pete-12	120362	15/02/2013	-2	78.907	10.507
ct92-M305Pete-12	120362	15/02/2013	-2	78.917	10.605
ct92-M305Pete-12	120362	15/02/2013	-2	78.916	10.592
ct92-M305Pete-12	120362	15/02/2013	-2	78.926	10.61
ct92-M305Pete-12	120362	15/02/2013	-2	78.93	10.591
ct92-M305Pete-12	120362	15/02/2013	-2	78.93	10.587
ct92-M305Pete-12	120362	15/02/2013	-2	78.93	10.588
ct92-M305Pete-12	120362	15/02/2013	-2	78.93	10.587
ct92-M305Pete-12	120362	15/02/2013	-2	78.929	10.59
ct92-M305Pete-12	120362	15/02/2013	-2	78.919	10.54
ct92-M305Pete-12	120362	16/02/2013	3	78.915	10.374
ct92-M305Pete-12	120362	16/02/2013	-1	78.915	10.375
ct92-M305Pete-12	120362	16/02/2013	-1	78.909	10.37
ct92-M305Pete-12	120362	16/02/2013	-2	78.91	10.372
ct92-M305Pete-12	120362	16/02/2013	-2	78.917	10.317
ct92-M305Pete-12	120362	16/02/2013	-2	78.913	10.288
ct92-M305Pete-12	120362	16/02/2013	-2	78.919	10.276
ct92-M305Pete-12	120362	16/02/2013	-2	78.919	10.271
ct92-M305Pete-12	120362	16/02/2013	-2	78.921	10.276
ct92-M305Pete-12	120362	16/02/2013	-2	78.935	10.248
ct92-M305Pete-12	120362	16/02/2013	-2	78.897	10.38
ct92-M305Pete-12	120362	16/02/2013	-2	78.921	10.364
ct92-M305Pete-12	120362	16/02/2013	-2	78.943	10.357
ct92-M305Pete-12	120362	16/02/2013	-2	78.933	10.395
ct92-M305Pete-12	120362	16/02/2013	-2	78.903	10.394
ct92-M305Pete-12	120362	16/02/2013	-2	78.901	10.385
ct92-M305Pete-12	120362	16/02/2013	-2	78.908	10.363
ct92-M305Pete-12	120362	16/02/2013	-2	78.931	10.383
ct92-M305Pete-12	120362	16/02/2013	-2	78.934	10.419
ct92-M305Pete-12	120362	16/02/2013	-2	78.932	10.419
ct92-M305Pete-12	120362	16/02/2013	-2	78.931	10.406
ct92-M305Pete-12	120362	16/02/2013	-2	78.932	10.432
ct92-M305Pete-12	120362	16/02/2013	-1	78.921	10.448
ct92-M305Pete-12	120362	16/02/2013	-2	78.931	10.407
ct92-M305Pete-12	120362	16/02/2013	-2	78.932	10.409
ct92-M305Pete-12	120362	16/02/2013	-1	78.924	10.437
ct92-M305Pete-12	120362	16/02/2013	-2	78.926	10.437
ct92-M305Pete-12	120362	16/02/2013	-2	78.925	10.448
ct92-M305Pete-12	120362	16/02/2013	-2	78.927	10.443
ct92-M305Pete-12	120362	16/02/2013	-2	78.939	10.359
ct92-M305Pete-12	120362	16/02/2013	-2	78.918	10.562

ct92-M305Pete-12	120362	16/02/2013	-2	78.918	10.571
ct92-M305Pete-12	120362	16/02/2013	-2	78.942	10.556
ct92-M305Pete-12	120362	16/02/2013	-2	78.924	10.503
ct92-M305Pete-12	120362	16/02/2013	-2	78.938	10.619
ct92-M305Pete-12	120362	16/02/2013	-2	78.936	10.602
ct92-M305Pete-12	120362	16/02/2013	-2	78.937	10.591
ct92-M305Pete-12	120362	16/02/2013	-2	78.939	10.597
ct92-M305Pete-12	120362	16/02/2013	-1	78.93	10.668
ct92-M305Pete-12	120362	16/02/2013	-2	78.924	10.696
ct92-M305Pete-12	120362	16/02/2013	-2	78.917	10.69
ct92-M305Pete-12	120362	16/02/2013	-2	78.917	10.69
ct92-M305Pete-12	120362	16/02/2013	-2	78.917	10.689
ct92-M305Pete-12	120362	16/02/2013	-1	78.926	10.773
ct92-M305Pete-12	120362	16/02/2013	2	78.922	10.734
ct92-M305Pete-12	120362	16/02/2013	2	78.921	10.739
ct92-M305Pete-12	120362	16/02/2013	2	78.929	10.755
ct92-M305Pete-12	120362	16/02/2013	-2	78.927	10.78
ct92-M305Pete-12	120362	16/02/2013	-2	78.926	10.724
ct92-M305Pete-12	120362	16/02/2013	-1	78.926	10.797
ct92-M305Pete-12	120362	17/02/2013	-2	78.932	10.742
ct92-M305Pete-12	120362	17/02/2013	-2	78.93	10.757
ct92-M305Pete-12	120362	17/02/2013	-2	78.919	10.669
ct92-M305Pete-12	120362	17/02/2013	-1	78.918	10.677
ct92-M305Pete-12	120362	17/02/2013	-2	78.917	10.791
ct92-M305Pete-12	120362	17/02/2013	-2	78.924	10.779
ct92-M305Pete-12	120362	17/02/2013	-2	78.917	10.688
ct92-M305Pete-12	120362	17/02/2013	-2	78.918	10.783
ct92-M305Pete-12	120362	17/02/2013	-2	78.917	10.753
ct92-M305Pete-12	120362	17/02/2013	-2	78.919	10.771
ct92-M305Pete-12	120362	17/02/2013	-2	78.922	10.749
ct92-M305Pete-12	120362	17/02/2013	-2	78.921	10.739
ct92-M305Pete-12	120362	17/02/2013	-2	78.919	10.735
ct92-M305Pete-12	120362	17/02/2013	-2	78.917	10.802
ct92-M305Pete-12	120362	17/02/2013	-2	78.921	10.781
ct92-M305Pete-12	120362	17/02/2013	-2	78.922	10.757
ct92-M305Pete-12	120362	17/02/2013	-2	78.92	10.897
ct92-M305Pete-12	120362	17/02/2013	-2	78.903	10.811
ct92-M305Pete-12	120362	17/02/2013	-2	78.923	10.76
ct92-M305Pete-12	120362	17/02/2013	-2	78.929	10.678
ct92-M305Pete-12	120362	17/02/2013	-1	78.922	10.599
ct92-M305Pete-12	120362	17/02/2013	-1	78.918	10.661
ct92-M305Pete-12	120362	17/02/2013	-1	78.914	10.716
ct92-M305Pete-12	120362	17/02/2013	-2	78.893	10.81
ct92-M305Pete-12	120362	17/02/2013	-2	78.933	10.685
ct92-M305Pete-12	120362	17/02/2013	-2	78.919	10.693
ct92-M305Pete-12	120362	17/02/2013	-2	78.919	10.75
ct92-M305Pete-12	120362	17/02/2013	-2	78.918	10.734
ct92-M305Pete-12	120362	17/02/2013	-2	78.922	10.771
ct92-M305Pete-12	120362	17/02/2013	-2	78.886	10.883
ct92-M305Pete-12	120362	17/02/2013	-2	78.906	10.794
ct92-M305Pete-12	120362	17/02/2013	-2	78.91	10.783
ct92-M305Pete-12	120362	17/02/2013	-2	78.916	10.89
ct92-M305Pete-12	120362	17/02/2013	-2	78.926	10.805
ct92-M305Pete-12	120362	17/02/2013	-2	78.916	10.749
ct92-M305Pete-12	120362	17/02/2013	-2	78.902	10.92
ct92-M305Pete-12	120362	17/02/2013	-2	78.916	10.752
ct92-M305Pete-12	120362	17/02/2013	-2	78.916	10.748
ct92-M305Pete-12	120362	17/02/2013	-2	78.91	10.758
ct92-M305Pete-12	120362	17/02/2013	-2	78.911	10.743
ct92-M305Pete-12	120362	17/02/2013	-2	78.91	10.663
ct92-M305Pete-12	120362	17/02/2013	-1	78.912	10.689
ct92-M305Pete-12	120362	17/02/2013	-1	78.915	10.636
ct92-M305Pete-12	120362	17/02/2013	-2	78.914	10.631
ct92-M305Pete-12	120362	17/02/2013	-2	78.912	10.723
ct92-M305Pete-12	120362	17/02/2013	-2	78.916	10.623
ct92-M305Pete-12	120362	17/02/2013	-2	78.922	10.664
ct92-M305Pete-12	120362	17/02/2013	-2	78.925	10.769
ct92-M305Pete-12	120362	17/02/2013	-2	78.925	10.669

ct92-M305Pete-12	120362	17/02/2013	-2	78.932	10.58
ct92-M305Pete-12	120362	17/02/2013	-2	78.926	10.692
ct92-M305Pete-12	120362	17/02/2013	-2	78.922	10.647
ct92-M305Pete-12	120362	17/02/2013	-1	78.909	10.572
ct92-M305Pete-12	120362	17/02/2013	-2	78.926	10.601
ct92-M305Pete-12	120362	17/02/2013	-2	78.918	10.581
ct92-M305Pete-12	120362	17/02/2013	-2	78.918	10.587
ct92-M305Pete-12	120362	17/02/2013	-2	78.918	10.583
ct92-M305Pete-12	120362	17/02/2013	-2	78.917	10.635
ct92-M305Pete-12	120362	17/02/2013	-2	78.917	10.611
ct92-M305Pete-12	120362	18/02/2013	-2	78.937	10.563
ct92-M305Pete-12	120362	18/02/2013	-2	78.912	10.579
ct92-M305Pete-12	120362	18/02/2013	-2	78.911	10.583
ct92-M305Pete-12	120362	18/02/2013	-2	78.913	10.586
ct92-M305Pete-12	120362	18/02/2013	-1	78.909	10.584
ct92-M305Pete-12	120362	18/02/2013	-2	78.92	10.634
ct92-M305Pete-12	120362	18/02/2013	-2	78.92	10.629
ct92-M305Pete-12	120362	18/02/2013	-2	78.918	10.671
ct92-M305Pete-12	120362	18/02/2013	-2	78.918	10.673
ct92-M305Pete-12	120362	18/02/2013	-2	78.923	10.708
ct92-M305Pete-12	120362	18/02/2013	-2	78.92	10.687
ct92-M305Pete-12	120362	18/02/2013	-2	78.932	10.672
ct92-M305Pete-12	120362	18/02/2013	-2	78.926	10.631
ct92-M305Pete-12	120362	18/02/2013	-2	78.92	10.619
ct92-M305Pete-12	120362	18/02/2013	-2	78.92	10.63
ct92-M305Pete-12	120362	18/02/2013	-2	78.916	10.621
ct92-M305Pete-12	120362	18/02/2013	-2	78.927	10.625
ct92-M305Pete-12	120362	18/02/2013	-2	78.933	10.686
ct92-M305Pete-12	120362	18/02/2013	-2	78.938	10.703
ct92-M305Pete-12	120362	18/02/2013	-2	78.951	10.581
ct92-M305Pete-12	120362	18/02/2013	-2	78.952	10.586
ct92-M305Pete-12	120362	18/02/2013	-2	78.946	10.601
ct92-M305Pete-12	120362	18/02/2013	-2	78.924	10.692
ct92-M305Pete-12	120362	18/02/2013	-2	78.914	10.559
ct92-M305Pete-12	120362	18/02/2013	-2	78.914	10.56
ct92-M305Pete-12	120362	18/02/2013	-2	78.914	10.567
ct92-M305Pete-12	120362	18/02/2013	-1	78.913	10.627
ct92-M305Pete-12	120362	18/02/2013	-2	78.91	10.607
ct92-M305Pete-12	120362	18/02/2013	-2	78.908	10.656
ct92-M305Pete-12	120362	18/02/2013	-2	78.916	10.651
ct92-M305Pete-12	120362	18/02/2013	-2	78.913	10.641
ct92-M305Pete-12	120362	18/02/2013	-2	78.913	10.641
ct92-M305Pete-12	120362	18/02/2013	-2	78.912	10.694
ct92-M305Pete-12	120362	18/02/2013	-2	78.908	10.702
ct92-M305Pete-12	120362	18/02/2013	-2	78.9	10.626
ct92-M305Pete-12	120362	18/02/2013	-2	78.911	10.697
ct92-M305Pete-12	120362	18/02/2013	-2	78.899	10.635
ct92-M305Pete-12	120362	18/02/2013	-1	78.94	10.715
ct92-M305Pete-12	120362	18/02/2013	-2	78.905	10.754
ct92-M305Pete-12	120362	18/02/2013	-2	78.918	10.716
ct92-M305Pete-12	120362	18/02/2013	-2	78.896	10.655
ct92-M305Pete-12	120362	18/02/2013	-2	78.914	10.596
ct92-M305Pete-12	120362	18/02/2013	-2	78.898	10.711
ct92-M305Pete-12	120362	18/02/2013	-2	78.903	10.518
ct92-M305Pete-12	120362	18/02/2013	-2	78.916	10.602
ct92-M305Pete-12	120362	18/02/2013	-2	78.904	10.52
ct92-M305Pete-12	120362	18/02/2013	-2	78.896	10.758
ct92-M305Pete-12	120362	18/02/2013	-2	78.914	10.748
ct92-M305Pete-12	120362	18/02/2013	-2	78.928	10.599
ct92-M305Pete-12	120362	18/02/2013	-2	78.929	10.459
ct92-M305Pete-12	120362	18/02/2013	-2	78.906	10.469
ct92-M305Pete-12	120362	18/02/2013	-2	78.926	10.488
ct92-M305Pete-12	120362	18/02/2013	-2	78.927	10.546
ct92-M305Pete-12	120362	18/02/2013	-2	78.927	10.585
ct92-M305Pete-12	120362	18/02/2013	-2	78.927	10.593
ct92-M305Pete-12	120362	18/02/2013	-2	78.929	10.614
ct92-M305Pete-12	120362	18/02/2013	-2	78.927	10.607
ct92-M305Pete-12	120362	18/02/2013	-2	78.926	10.618

ct92-M305Pete-12	120362	18/02/2013	-2	78.927	10.611
ct92-M305Pete-12	120362	18/02/2013	-2	78.912	10.634
ct92-M305Pete-12	120362	18/02/2013	-2	78.915	10.652
ct92-M305Pete-12	120362	18/02/2013	-2	78.904	10.686
ct92-M305Pete-12	120362	19/02/2013	-2	78.907	10.699
ct92-M305Pete-12	120362	19/02/2013	-2	78.911	10.741
ct92-M305Pete-12	120362	19/02/2013	-2	78.9	10.686
ct92-M305Pete-12	120362	19/02/2013	-1	78.901	10.657
ct92-M305Pete-12	120362	19/02/2013	-2	78.913	10.642
ct92-M305Pete-12	120362	19/02/2013	-2	78.915	10.649
ct92-M305Pete-12	120362	19/02/2013	-2	78.917	10.656
ct92-M305Pete-12	120362	19/02/2013	-2	78.898	10.667
ct92-M305Pete-12	120362	19/02/2013	-2	78.926	10.69
ct92-M305Pete-12	120362	19/02/2013	-2	78.924	10.707
ct92-M305Pete-12	120362	19/02/2013	-2	78.888	10.667
ct92-M305Pete-12	120362	19/02/2013	-1	78.913	10.733
ct92-M305Pete-12	120362	19/02/2013	-2	78.909	10.741
ct92-M305Pete-12	120362	19/02/2013	-2	78.911	10.738
ct92-M305Pete-12	120362	19/02/2013	-2	78.907	10.747
ct92-M305Pete-12	120362	19/02/2013	-2	78.902	10.698
ct92-M305Pete-12	120362	19/02/2013	-2	78.906	10.747
ct92-M305Pete-12	120362	19/02/2013	-2	78.906	10.739
ct92-M305Pete-12	120362	19/02/2013	-1	78.911	10.675
ct92-M305Pete-12	120362	19/02/2013	-2	78.928	10.66
ct92-M305Pete-12	120362	19/02/2013	-2	78.903	10.626
ct92-M305Pete-12	120362	19/02/2013	-2	78.917	10.642
ct92-M305Pete-12	120362	19/02/2013	-2	78.924	10.678
ct92-M305Pete-12	120362	19/02/2013	-2	78.92	10.656
ct92-M305Pete-12	120362	19/02/2013	-2	78.917	10.64
ct92-M305Pete-12	120362	19/02/2013	-2	78.917	10.64
ct92-M305Pete-12	120362	19/02/2013	-2	78.918	10.624
ct92-M305Pete-12	120362	19/02/2013	-2	78.917	10.639
ct92-M305Pete-12	120362	19/02/2013	-1	78.915	10.669
ct92-M305Pete-12	120362	19/02/2013	-2	78.915	10.668
ct92-M305Pete-12	120362	19/02/2013	-2	78.908	10.61
ct92-M305Pete-12	120362	19/02/2013	-2	78.915	10.592
ct92-M305Pete-12	120362	19/02/2013	-2	78.913	10.599
ct92-M305Pete-12	120362	19/02/2013	-2	78.914	10.648
ct92-M305Pete-12	120362	19/02/2013	-2	78.913	10.642
ct92-M305Pete-12	120362	19/02/2013	-2	78.915	10.669
ct92-M305Pete-12	120362	19/02/2013	-2	78.931	10.69
ct92-M305Pete-12	120362	19/02/2013	-2	78.911	10.724
ct92-M305Pete-12	120362	19/02/2013	-2	78.916	10.641
ct92-M305Pete-12	120362	19/02/2013	-2	78.92	10.647
ct92-M305Pete-12	120362	19/02/2013	-2	78.917	10.651
ct92-M305Pete-12	120362	19/02/2013	-2	78.92	10.647
ct92-M305Pete-12	120362	19/02/2013	-2	78.923	10.677
ct92-M305Pete-12	120362	19/02/2013	-2	78.917	10.694
ct92-M305Pete-12	120362	19/02/2013	-1	78.927	10.745
ct92-M305Pete-12	120362	19/02/2013	-2	78.944	10.73
ct92-M305Pete-12	120362	19/02/2013	-2	78.943	10.73
ct92-M305Pete-12	120362	19/02/2013	-2	78.922	10.804
ct92-M305Pete-12	120362	19/02/2013	-1	78.918	10.757
ct92-M305Pete-12	120362	19/02/2013	-2	78.921	10.755
ct92-M305Pete-12	120362	19/02/2013	-2	78.926	10.693
ct92-M305Pete-12	120362	19/02/2013	-2	78.925	10.604
ct92-M305Pete-12	120362	19/02/2013	-2	78.924	10.667
ct92-M305Pete-12	120362	19/02/2013	-1	78.918	10.585
ct92-M305Pete-12	120362	19/02/2013	-2	78.919	10.569
ct92-M305Pete-12	120362	19/02/2013	-2	78.918	10.582
ct92-M305Pete-12	120362	19/02/2013	-1	78.91	10.607
ct92-M305Pete-12	120362	19/02/2013	-2	78.917	10.623
ct92-M305Pete-12	120362	19/02/2013	-2	78.918	10.628
ct92-M305Pete-12	120362	19/02/2013	-2	78.918	10.624
ct92-M305Pete-12	120362	19/02/2013	-2	78.917	10.611
ct92-M305Pete-12	120362	19/02/2013	-2	78.918	10.606
ct92-M305Pete-12	120362	19/02/2013	-1	78.928	10.586
ct92-M305Pete-12	120362	19/02/2013	-1	78.927	10.587

ct92-M305Pete-12	120362	20/02/2013	-2	78.927	10.58
ct92-M305Pete-12	120362	20/02/2013	-2	78.927	10.566
ct92-M305Pete-12	120362	20/02/2013	-2	78.909	10.651
ct92-M305Pete-12	120362	20/02/2013	-2	78.907	10.644
ct92-M305Pete-12	120362	20/02/2013	-2	78.925	10.599
ct92-M305Pete-12	120362	20/02/2013	-2	78.931	10.543
ct92-M305Pete-12	120362	20/02/2013	-1	78.909	10.674
ct92-M305Pete-12	120362	20/02/2013	-2	78.931	10.568
ct92-M305Pete-12	120362	20/02/2013	-2	78.92	10.613
ct92-M305Pete-12	120362	20/02/2013	-2	78.921	10.592
ct92-M305Pete-12	120362	20/02/2013	-2	78.92	10.589
ct92-M305Pete-12	120362	20/02/2013	-2	78.929	10.754
ct92-M305Pete-12	120362	20/02/2013	-2	78.922	10.591
ct92-M305Pete-12	120362	20/02/2013	-2	78.909	10.695
ct92-M305Pete-12	120362	20/02/2013	-2	78.916	10.679
ct92-M305Pete-12	120362	20/02/2013	-2	78.901	10.649
ct92-M305Pete-12	120362	20/02/2013	-2	78.916	10.676
ct92-M305Pete-12	120362	20/02/2013	-2	78.916	10.673
ct92-M305Pete-12	120362	20/02/2013	-2	78.917	10.665
ct92-M305Pete-12	120362	20/02/2013	-2	78.917	10.665
ct92-M305Pete-12	120362	20/02/2013	-2	78.916	10.65
ct92-M305Pete-12	120362	20/02/2013	-2	78.917	10.665
ct92-M305Pete-12	120362	20/02/2013	-1	78.902	10.735
ct92-M305Pete-12	120362	20/02/2013	-1	78.92	10.695
ct92-M305Pete-12	120362	20/02/2013	-2	78.921	10.611
ct92-M305Pete-12	120362	20/02/2013	-2	78.912	10.648
ct92-M305Pete-12	120362	20/02/2013	-2	78.919	10.598
ct92-M305Pete-12	120362	20/02/2013	-2	78.912	10.654
ct92-M305Pete-12	120362	20/02/2013	-1	78.917	10.682
ct92-M305Pete-12	120362	20/02/2013	-1	78.913	10.656
ct92-M305Pete-12	120362	20/02/2013	-1	78.91	10.609
ct92-M305Pete-12	120362	20/02/2013	-1	78.897	10.634
ct92-M305Pete-12	120362	20/02/2013	-2	78.897	10.637
ct92-M305Pete-12	120362	20/02/2013	-2	78.907	10.626
ct92-M305Pete-12	120362	20/02/2013	-2	78.909	10.675
ct92-M305Pete-12	120362	20/02/2013	-2	78.914	10.671
ct92-M305Pete-12	120362	20/02/2013	-2	78.907	10.601
ct92-M305Pete-12	120362	20/02/2013	0	78.916	10.602
ct92-M305Pete-12	120362	20/02/2013	-1	78.915	10.725
ct92-M305Pete-12	120362	20/02/2013	0	78.946	10.729
ct92-M305Pete-12	120362	20/02/2013	-2	78.902	10.591
ct92-M305Pete-12	120362	20/02/2013	-1	78.913	10.691
ct92-M305Pete-12	120362	20/02/2013	-2	78.92	10.681
ct92-M305Pete-12	120362	20/02/2013	-2	78.918	10.735
ct92-M305Pete-12	120362	20/02/2013	-2	78.916	10.748
ct92-M305Pete-12	120362	20/02/2013	-2	78.92	10.742
ct92-M305Pete-12	120362	20/02/2013	-2	78.92	10.742
ct92-M305Pete-12	120362	20/02/2013	-2	78.913	10.814
ct92-M305Pete-12	120362	20/02/2013	-2	78.914	10.789
ct92-M305Pete-12	120362	20/02/2013	-2	78.911	10.694
ct92-M305Pete-12	120362	20/02/2013	-2	78.907	10.698
ct92-M305Pete-12	120362	20/02/2013	-2	78.911	10.777
ct92-M305Pete-12	120362	20/02/2013	-2	78.911	10.788
ct92-M305Pete-12	120362	20/02/2013	-2	78.9	10.726
ct92-M305Pete-12	120362	20/02/2013	-1	78.912	10.766
ct92-M305Pete-12	120362	20/02/2013	-2	78.9	10.727
ct92-M305Pete-12	120362	20/02/2013	-2	78.899	10.733
ct92-M305Pete-12	120362	20/02/2013	-2	78.9	10.726
ct92-M305Pete-12	120362	20/02/2013	-2	78.9	10.729
ct92-M305Pete-12	120362	20/02/2013	-2	78.9	10.791
ct92-M305Pete-12	120362	20/02/2013	-2	78.894	10.685
ct92-M305Pete-12	120362	20/02/2013	-2	78.893	10.678
ct92-M305Pete-12	120362	20/02/2013	-2	78.896	10.683
ct92-M305Pete-12	120362	20/02/2013	-2	78.894	10.697
ct92-M305Pete-12	120362	20/02/2013	-2	78.901	10.679
ct92-M305Pete-12	120362	20/02/2013	-2	78.9	10.674
ct92-M305Pete-12	120362	21/02/2013	-2	78.916	10.738
ct92-M305Pete-12	120362	21/02/2013	-2	78.896	10.671

ct92-M305Pete-12	120362	21/02/2013	-2	78.893	10.707
ct92-M305Pete-12	120362	21/02/2013	-2	78.899	10.69
ct92-M305Pete-12	120362	21/02/2013	-2	78.897	10.688
ct92-M305Pete-12	120362	21/02/2013	-2	78.901	10.708
ct92-M305Pete-12	120362	21/02/2013	-2	78.905	10.696
ct92-M305Pete-12	120362	21/02/2013	-2	78.885	10.651
ct92-M305Pete-12	120362	21/02/2013	-2	78.926	10.484
ct92-M305Pete-12	120362	21/02/2013	-2	78.936	10.703
ct92-M305Pete-12	120362	21/02/2013	-2	78.935	10.667
ct92-M305Pete-12	120362	21/02/2013	-2	78.912	10.632
ct92-M305Pete-12	120362	21/02/2013	-2	78.933	10.668
ct92-M305Pete-12	120362	21/02/2013	-2	78.934	10.659
ct92-M305Pete-12	120362	21/02/2013	-2	78.913	10.565
ct92-M305Pete-12	120362	21/02/2013	-2	78.902	10.616
ct92-M305Pete-12	120362	21/02/2013	-2	78.929	10.539
ct92-M305Pete-12	120362	21/02/2013	-2	78.931	10.53
ct92-M305Pete-12	120362	21/02/2013	-2	78.914	10.638
ct92-M305Pete-12	120362	21/02/2013	-2	78.916	10.615
ct92-M305Pete-12	120362	21/02/2013	-2	78.903	10.602
ct92-M305Pete-12	120362	21/02/2013	-2	78.904	10.6
ct92-M305Pete-12	120362	21/02/2013	-2	78.914	10.617
ct92-M305Pete-12	120362	21/02/2013	-2	78.898	10.601
ct92-M305Pete-12	120362	21/02/2013	-2	78.888	10.62
ct92-M305Pete-12	120362	21/02/2013	-2	78.904	10.646
ct92-M305Pete-12	120362	21/02/2013	-2	78.9	10.569
ct92-M305Pete-12	120362	21/02/2013	-2	78.901	10.603
ct92-M305Pete-12	120362	21/02/2013	-2	78.905	10.615
ct92-M305Pete-12	120362	21/02/2013	-2	78.915	10.63
ct92-M305Pete-12	120362	21/02/2013	-2	78.921	10.634
ct92-M305Pete-12	120362	21/02/2013	-2	78.925	10.625
ct92-M305Pete-12	120362	21/02/2013	-2	78.925	10.636
ct92-M305Pete-12	120362	21/02/2013	-2	78.924	10.636
ct92-M305Pete-12	120362	21/02/2013	-2	78.915	10.615
ct92-M305Pete-12	120362	21/02/2013	-2	78.919	10.574
ct92-M305Pete-12	120362	21/02/2013	-2	78.926	10.577
ct92-M305Pete-12	120362	21/02/2013	-2	78.939	10.701
ct92-M305Pete-12	120362	21/02/2013	-2	78.929	10.762
ct92-M305Pete-12	120362	21/02/2013	-2	78.945	10.641
ct92-M305Pete-12	120362	21/02/2013	-2	78.946	10.651
ct92-M305Pete-12	120362	21/02/2013	-2	78.949	10.599
ct92-M305Pete-12	120362	21/02/2013	-2	78.946	10.714
ct92-M305Pete-12	120362	21/02/2013	-2	78.959	10.591
ct92-M305Pete-12	120362	21/02/2013	-2	78.956	10.536
ct92-M305Pete-12	120362	21/02/2013	-2	78.935	10.47
ct92-M305Pete-12	120362	21/02/2013	-2	78.95	10.491
ct92-M305Pete-12	120362	21/02/2013	-2	78.945	10.358
ct92-M305Pete-12	120362	21/02/2013	-2	78.953	10.445
ct92-M305Pete-12	120362	21/02/2013	-2	78.926	10.463
ct92-M305Pete-12	120362	21/02/2013	-2	78.93	10.527
ct92-M305Pete-12	120362	21/02/2013	-2	78.93	10.518
ct92-M305Pete-12	120362	21/02/2013	-2	78.926	10.44
ct92-M305Pete-12	120362	21/02/2013	-2	78.932	10.481
ct92-M305Pete-12	120362	21/02/2013	-2	78.924	10.466
ct92-M305Pete-12	120362	21/02/2013	-2	78.923	10.451
ct92-M305Pete-12	120362	21/02/2013	-2	78.925	10.481
ct92-M305Pete-12	120362	21/02/2013	-2	78.924	10.475
ct92-M305Pete-12	120362	22/02/2013	-2	78.922	10.453
ct92-M305Pete-12	120362	22/02/2013	-1	78.931	10.464
ct92-M305Pete-12	120362	22/02/2013	-2	78.929	10.483
ct92-M305Pete-12	120362	22/02/2013	-2	78.925	10.424
ct92-M305Pete-12	120362	22/02/2013	-2	78.926	10.411
ct92-M305Pete-12	120362	22/02/2013	-2	78.927	10.25
ct92-M305Pete-12	120362	22/02/2013	-2	78.931	10.271
ct92-M305Pete-12	120362	22/02/2013	-2	78.946	10.257
ct92-M305Pete-12	120362	22/02/2013	-1	78.919	10.407
ct92-M305Pete-12	120362	22/02/2013	-2	78.923	10.37
ct92-M305Pete-12	120362	22/02/2013	-2	78.925	10.369
ct92-M305Pete-12	120362	22/02/2013	-2	78.927	10.362

ct92-M305Pete-12	120362	22/02/2013	-2	78.912	10.42
ct92-M305Pete-12	120362	22/02/2013	-1	78.907	10.395
ct92-M305Pete-12	120362	22/02/2013	-2	78.913	10.387
ct92-M305Pete-12	120362	22/02/2013	-2	78.914	10.381
ct92-M305Pete-12	120362	22/02/2013	-2	78.913	10.379
ct92-M305Pete-12	120362	22/02/2013	-2	78.913	10.38
ct92-M305Pete-12	120362	22/02/2013	-1	78.916	10.423
ct92-M305Pete-12	120362	22/02/2013	-2	78.916	10.423
ct92-M305Pete-12	120362	22/02/2013	-1	78.915	10.417
ct92-M305Pete-12	120362	22/02/2013	-1	78.92	10.4
ct92-M305Pete-12	120362	22/02/2013	-2	78.915	10.419
ct92-M305Pete-12	120362	22/02/2013	-2	78.926	10.419
ct92-M305Pete-12	120362	22/02/2013	-2	78.918	10.412
ct92-M305Pete-12	120362	22/02/2013	0	78.904	10.497
ct92-M305Pete-12	120362	22/02/2013	-2	78.923	10.458
ct92-M305Pete-12	120362	22/02/2013	-2	78.923	10.455
ct92-M305Pete-12	120362	22/02/2013	-2	78.916	10.422
ct92-M305Pete-12	120362	22/02/2013	-2	78.927	10.422
ct92-M305Pete-12	120362	22/02/2013	-1	78.959	10.474
ct92-M305Pete-12	120362	22/02/2013	0	78.935	10.399
ct92-M305Pete-12	120362	22/02/2013	-1	78.934	10.435
ct92-M305Pete-12	120362	22/02/2013	-2	78.914	10.461
ct92-M305Pete-12	120362	22/02/2013	-1	78.932	10.574
ct92-M305Pete-12	120362	22/02/2013	-2	78.924	10.467
ct92-M305Pete-12	120362	22/02/2013	-2	78.917	10.356
ct92-M305Pete-12	120362	22/02/2013	-2	78.927	10.512
ct92-M305Pete-12	120362	22/02/2013	-1	78.938	10.555
ct92-M305Pete-12	120362	22/02/2013	0	78.948	10.548
ct92-M305Pete-12	120362	22/02/2013	1	78.923	10.57
ct92-M305Pete-12	120362	22/02/2013	-2	78.929	10.624
ct92-M305Pete-12	120362	22/02/2013	-2	78.922	10.57
ct92-M305Pete-12	120362	22/02/2013	-2	78.923	10.605
ct92-M305Pete-12	120362	22/02/2013	0	78.915	10.701
ct92-M305Pete-12	120362	22/02/2013	0	78.946	10.649
ct92-M305Pete-12	120362	22/02/2013	-2	78.921	10.652
ct92-M305Pete-12	120362	22/02/2013	-2	78.924	10.779
ct92-M305Pete-12	120362	22/02/2013	-1	78.926	10.762
ct92-M305Pete-12	120362	22/02/2013	-2	78.918	10.798
ct92-M305Pete-12	120362	22/02/2013	-2	78.929	10.834
ct92-M305Pete-12	120362	22/02/2013	-1	78.905	10.786
ct92-M305Pete-12	120362	22/02/2013	-1	78.909	10.726
ct92-M305Pete-12	120362	22/02/2013	-1	78.898	10.705
ct92-M305Pete-12	120362	22/02/2013	-2	78.916	10.661
ct92-M305Pete-12	120362	22/02/2013	-2	78.915	10.685
ct92-M305Pete-12	120362	22/02/2013	-2	78.915	10.685
ct92-M305Pete-12	120362	22/02/2013	-2	78.904	10.728
ct92-M305Pete-12	120362	22/02/2013	-2	78.9	10.743
ct92-M305Pete-12	120362	22/02/2013	-2	78.907	10.712
ct92-M305Pete-12	120362	22/02/2013	-2	78.907	10.71
ct92-M305Pete-12	120362	22/02/2013	-2	78.893	10.7
ct92-M305Pete-12	120362	22/02/2013	-2	78.9	10.682
ct92-M305Pete-12	120362	22/02/2013	-2	78.894	10.674
ct92-M305Pete-12	120362	22/02/2013	-2	78.888	10.693
ct92-M305Pete-12	120362	22/02/2013	-2	78.909	10.782
ct92-M305Pete-12	120362	22/02/2013	-2	78.909	10.772
ct92-M305Pete-12	120362	22/02/2013	-2	78.906	10.79
ct92-M305Pete-12	120362	22/02/2013	-2	78.919	10.48
ct92-M305Pete-12	120362	22/02/2013	-2	78.896	10.766
ct92-M305Pete-12	120362	22/02/2013	-2	78.897	10.73
ct92-M305Pete-12	120362	23/02/2013	-2	78.92	10.515
ct92-M305Pete-12	120362	23/02/2013	-2	78.911	10.499
ct92-M305Pete-12	120362	23/02/2013	-2	78.907	10.564
ct92-M305Pete-12	120362	23/02/2013	-2	78.931	10.529
ct92-M305Pete-12	120362	23/02/2013	-2	78.924	10.554
ct92-M305Pete-12	120362	23/02/2013	-2	78.923	10.544
ct92-M305Pete-12	120362	23/02/2013	-2	78.918	10.579
ct92-M305Pete-12	120362	23/02/2013	-2	78.897	10.628
ct92-M305Pete-12	120362	23/02/2013	-2	78.898	10.633

ct92-M305Pete-12	120362	23/02/2013	-2	78.893	10.661
ct92-M305Pete-12	120362	23/02/2013	-2	78.888	10.748
ct92-M305Pete-12	120362	23/02/2013	-1	78.925	10.717
ct92-M305Pete-12	120362	23/02/2013	-2	78.925	10.654
ct92-M305Pete-12	120362	23/02/2013	-2	78.923	10.605
ct92-M305Pete-12	120362	23/02/2013	-2	78.917	10.689
ct92-M305Pete-12	120362	23/02/2013	-2	78.915	10.665
ct92-M305Pete-12	120362	23/02/2013	-2	78.912	10.74
ct92-M305Pete-12	120362	23/02/2013	-2	78.91	10.536
ct92-M305Pete-12	120362	23/02/2013	-2	78.929	10.66
ct92-M305Pete-12	120362	23/02/2013	-2	78.91	10.708
ct92-M305Pete-12	120362	23/02/2013	-2	78.901	10.599
ct92-M305Pete-12	120362	23/02/2013	-2	78.913	10.619
ct92-M305Pete-12	120362	23/02/2013	-1	78.903	10.753
ct92-M305Pete-12	120362	23/02/2013	-2	78.928	10.646
ct92-M305Pete-12	120362	23/02/2013	-2	78.921	10.674
ct92-M305Pete-12	120362	23/02/2013	-1	78.909	10.67
ct92-M305Pete-12	120362	23/02/2013	-2	78.91	10.666
ct92-M305Pete-12	120362	23/02/2013	-2	78.91	10.653
ct92-M305Pete-12	120362	23/02/2013	-2	78.905	10.646
ct92-M305Pete-12	120362	23/02/2013	-2	78.912	10.666
ct92-M305Pete-12	120362	23/02/2013	-2	78.921	10.668
ct92-M305Pete-12	120362	23/02/2013	-2	78.919	10.649
ct92-M305Pete-12	120362	23/02/2013	-2	78.922	10.64
ct92-M305Pete-12	120362	23/02/2013	-2	78.923	10.64
ct92-M305Pete-12	120362	23/02/2013	-1	78.94	10.794
ct92-M305Pete-12	120362	23/02/2013	-2	78.939	10.819
ct92-M305Pete-12	120362	23/02/2013	-2	78.923	10.659
ct92-M305Pete-12	120362	23/02/2013	-2	78.92	10.652
ct92-M305Pete-12	120362	23/02/2013	-2	78.92	10.691
ct92-M305Pete-12	120362	23/02/2013	-2	78.923	10.679
ct92-M305Pete-12	120362	23/02/2013	-2	78.922	10.706
ct92-M305Pete-12	120362	23/02/2013	-2	78.91	10.692
ct92-M305Pete-12	120362	23/02/2013	-2	78.91	10.701
ct92-M305Pete-12	120362	23/02/2013	-2	78.916	10.704
ct92-M305Pete-12	120362	23/02/2013	-2	78.901	10.716
ct92-M305Pete-12	120362	23/02/2013	-2	78.921	10.743
ct92-M305Pete-12	120362	23/02/2013	-2	78.916	10.757
ct92-M305Pete-12	120362	23/02/2013	-1	78.918	10.71
ct92-M305Pete-12	120362	23/02/2013	-2	78.913	10.728
ct92-M305Pete-12	120362	23/02/2013	-2	78.907	10.711
ct92-M305Pete-12	120362	23/02/2013	-1	78.908	10.728
ct92-M305Pete-12	120362	23/02/2013	-2	78.912	10.742
ct92-M305Pete-12	120362	23/02/2013	-2	78.912	10.741
ct92-M305Pete-12	120362	23/02/2013	-2	78.916	10.718
ct92-M305Pete-12	120362	23/02/2013	-2	78.905	10.731
ct92-M305Pete-12	120362	23/02/2013	-2	78.915	10.719
ct92-M305Pete-12	120362	23/02/2013	-2	78.904	10.747
ct92-M305Pete-12	120362	23/02/2013	-2	78.902	10.802
ct92-M305Pete-12	120362	23/02/2013	-2	78.902	10.802
ct92-M305Pete-12	120362	23/02/2013	-2	78.901	10.806
ct92-M305Pete-12	120362	23/02/2013	-2	78.909	10.837
ct92-M305Pete-12	120362	23/02/2013	-2	78.906	10.86
ct92-M305Pete-12	120362	23/02/2013	-2	78.907	10.894
ct92-M305Pete-12	120362	23/02/2013	-2	78.91	10.871
ct92-M305Pete-12	120362	23/02/2013	-2	78.897	10.855
ct92-M305Pete-12	120362	23/02/2013	-2	78.898	10.842
ct92-M305Pete-12	120362	23/02/2013	-2	78.899	10.854
ct92-M305Pete-12	120362	23/02/2013	-2	78.902	10.806
ct92-M305Pete-12	120362	23/02/2013	-2	78.889	10.865
ct92-M305Pete-12	120362	24/02/2013	-2	78.899	10.833
ct92-M305Pete-12	120362	24/02/2013	-2	78.886	10.869
ct92-M305Pete-12	120362	24/02/2013	-2	78.893	10.918
ct92-M305Pete-12	120362	24/02/2013	-2	78.891	10.874
ct92-M305Pete-12	120362	24/02/2013	-1	78.897	10.83
ct92-M305Pete-12	120362	24/02/2013	-2	78.898	10.9
ct92-M305Pete-12	120362	24/02/2013	-2	78.892	10.877
ct92-M305Pete-12	120362	24/02/2013	-2	78.891	10.877

ct92-M305Pete-12	120362	24/02/2013	-2	78.893	10.865
ct92-M305Pete-12	120362	24/02/2013	-2	78.886	10.882
ct92-M305Pete-12	120362	24/02/2013	-2	78.884	10.893
ct92-M305Pete-12	120362	24/02/2013	-2	78.887	10.91
ct92-M305Pete-12	120362	24/02/2013	-2	78.879	10.905
ct92-M305Pete-12	120362	24/02/2013	-2	78.887	10.912
ct92-M305Pete-12	120362	24/02/2013	-1	78.886	10.841
ct92-M305Pete-12	120362	24/02/2013	-2	78.889	10.831
ct92-M305Pete-12	120362	24/02/2013	-2	78.898	10.835
ct92-M305Pete-12	120362	24/02/2013	-2	78.884	10.84
ct92-M305Pete-12	120362	24/02/2013	-2	78.885	10.885
ct92-M305Pete-12	120362	24/02/2013	-2	78.883	10.873
ct92-M305Pete-12	120362	24/02/2013	-2	78.89	10.885
ct92-M305Pete-12	120362	24/02/2013	-2	78.891	10.887
ct92-M305Pete-12	120362	24/02/2013	-2	78.896	10.867
ct92-M305Pete-12	120362	24/02/2013	-2	78.895	10.851
ct92-M305Pete-12	120362	24/02/2013	-2	78.894	10.86
ct92-M305Pete-12	120362	24/02/2013	-2	78.889	10.849
ct92-M305Pete-12	120362	24/02/2013	-2	78.894	10.854
ct92-M305Pete-12	120362	24/02/2013	-2	78.893	10.853
ct92-M305Pete-12	120362	24/02/2013	-2	78.899	10.861
ct92-M305Pete-12	120362	24/02/2013	-1	78.901	10.807
ct92-M305Pete-12	120362	24/02/2013	-2	78.901	10.8
ct92-M305Pete-12	120362	24/02/2013	-2	78.89	10.759
ct92-M305Pete-12	120362	24/02/2013	-2	78.912	10.788
ct92-M305Pete-12	120362	24/02/2013	-2	78.89	10.755
ct92-M305Pete-12	120362	24/02/2013	-2	78.895	10.74
ct92-M305Pete-12	120362	24/02/2013	-2	78.894	10.752
ct92-M305Pete-12	120362	24/02/2013	-2	78.895	10.751
ct92-M305Pete-12	120362	24/02/2013	-1	78.906	10.798
ct92-M305Pete-12	120362	24/02/2013	-1	78.889	10.81
ct92-M305Pete-12	120362	24/02/2013	-1	78.934	10.746
ct92-M305Pete-12	120362	24/02/2013	-1	78.934	10.745
ct92-M305Pete-12	120362	24/02/2013	-2	78.912	10.785
ct92-M305Pete-12	120362	24/02/2013	-2	78.912	10.783
ct92-M305Pete-12	120362	24/02/2013	-1	78.909	10.826
ct92-M305Pete-12	120362	24/02/2013	-2	78.913	10.765
ct92-M305Pete-12	120362	24/02/2013	-2	78.91	10.76
ct92-M305Pete-12	120362	24/02/2013	-2	78.912	10.768
ct92-M305Pete-12	120362	24/02/2013	-1	78.914	10.77
ct92-M305Pete-12	120362	24/02/2013	-2	78.914	10.773
ct92-M305Pete-12	120362	24/02/2013	-2	78.913	10.789
ct92-M305Pete-12	120362	24/02/2013	-2	78.905	10.876
ct92-M305Pete-12	120362	24/02/2013	-2	78.898	10.841
ct92-M305Pete-12	120362	24/02/2013	-2	78.9	10.818
ct92-M305Pete-12	120362	24/02/2013	-2	78.91	10.889
ct92-M305Pete-12	120362	24/02/2013	-2	78.91	10.883
ct92-M305Pete-12	120362	24/02/2013	-2	78.903	10.86
ct92-M305Pete-12	120362	24/02/2013	-2	78.903	10.855
ct92-M305Pete-12	120362	24/02/2013	-2	78.898	10.842
ct92-M305Pete-12	120362	24/02/2013	-2	78.911	10.877
ct92-M305Pete-12	120362	24/02/2013	-2	78.901	10.879
ct92-M305Pete-12	120362	24/02/2013	-2	78.901	10.84
ct92-M305Pete-12	120362	24/02/2013	-1	78.898	10.838
ct92-M305Pete-12	120362	24/02/2013	-2	78.89	10.853
ct92-M305Pete-12	120362	24/02/2013	-2	78.89	10.911
ct92-M305Pete-12	120362	24/02/2013	-2	78.894	10.856
ct92-M305Pete-12	120362	24/02/2013	-1	78.896	10.836
ct92-M305Pete-12	120362	24/02/2013	-1	78.898	10.87
ct92-M305Pete-12	120362	24/02/2013	-2	78.895	10.869
ct92-M305Pete-12	120362	24/02/2013	-2	78.892	10.871
ct92-M305Pete-12	120362	24/02/2013	-2	78.892	10.875
ct92-M305Pete-12	120362	24/02/2013	-2	78.894	10.874
ct92-M305Pete-12	120362	24/02/2013	-2	78.893	10.873
ct92-M305Pete-12	120362	25/02/2013	-2	78.883	10.94
ct92-M305Pete-12	120362	25/02/2013	-2	78.891	10.901
ct92-M305Pete-12	120362	25/02/2013	-2	78.894	10.916
ct92-M305Pete-12	120362	25/02/2013	-2	78.893	10.919

ct92-M305Pete-12	120362	25/02/2013	-2	78.895	10.921
ct92-M305Pete-12	120362	25/02/2013	-2	78.891	10.927
ct92-M305Pete-12	120362	25/02/2013	-1	78.907	10.837
ct92-M305Pete-12	120362	25/02/2013	-1	78.894	10.882
ct92-M305Pete-12	120362	25/02/2013	-2	78.895	10.88
ct92-M305Pete-12	120362	25/02/2013	-2	78.894	10.875
ct92-M305Pete-12	120362	25/02/2013	-2	78.889	10.841
ct92-M305Pete-12	120362	25/02/2013	-2	78.89	10.84
ct92-M305Pete-12	120362	25/02/2013	-2	78.888	10.849
ct92-M305Pete-12	120362	25/02/2013	-2	78.919	10.852
ct92-M305Pete-12	120362	25/02/2013	-2	78.889	10.815
ct92-M305Pete-12	120362	25/02/2013	-2	78.884	10.821
ct92-M305Pete-12	120362	25/02/2013	-2	78.9	10.77
ct92-M305Pete-12	120362	25/02/2013	-2	78.897	10.783
ct92-M305Pete-12	120362	25/02/2013	-2	78.88	10.819
ct92-M305Pete-12	120362	25/02/2013	-2	78.901	10.771
ct92-M305Pete-12	120362	25/02/2013	-2	78.9	10.775
ct92-M305Pete-12	120362	25/02/2013	-2	78.884	10.813
ct92-M305Pete-12	120362	25/02/2013	-2	78.901	10.781
ct92-M305Pete-12	120362	25/02/2013	-1	78.893	10.716
ct92-M305Pete-12	120362	25/02/2013	-1	78.89	10.713
ct92-M305Pete-12	120362	25/02/2013	-2	78.91	10.731
ct92-M305Pete-12	120362	25/02/2013	-2	78.893	10.732
ct92-M305Pete-12	120362	25/02/2013	-2	78.91	10.737
ct92-M305Pete-12	120362	25/02/2013	-2	78.899	10.7
ct92-M305Pete-12	120362	25/02/2013	-2	78.91	10.738
ct92-M305Pete-12	120362	25/02/2013	-2	78.91	10.738
ct92-M305Pete-12	120362	25/02/2013	-1	78.91	10.754
ct92-M305Pete-12	120362	25/02/2013	-1	78.911	10.749
ct92-M305Pete-12	120362	25/02/2013	-2	78.912	10.742
ct92-M305Pete-12	120362	25/02/2013	-2	78.92	10.722
ct92-M305Pete-12	120362	25/02/2013	-2	78.917	10.694
ct92-M305Pete-12	120362	25/02/2013	-1	78.924	10.752
ct92-M305Pete-12	120362	25/02/2013	-1	78.923	10.78
ct92-M305Pete-12	120362	25/02/2013	-2	78.922	10.619
ct92-M305Pete-12	120362	25/02/2013	-2	78.922	10.616
ct92-M305Pete-12	120362	25/02/2013	-2	78.926	10.734
ct92-M305Pete-12	120362	25/02/2013	-2	78.938	10.72
ct92-M305Pete-12	120362	25/02/2013	-2	78.915	10.781
ct92-M305Pete-12	120362	25/02/2013	-2	78.915	10.769
ct92-M305Pete-12	120362	25/02/2013	-2	78.923	10.807
ct92-M305Pete-12	120362	25/02/2013	-2	78.933	10.702
ct92-M305Pete-12	120362	25/02/2013	-2	78.933	10.699
ct92-M305Pete-12	120362	25/02/2013	-2	78.93	10.725
ct92-M305Pete-12	120362	25/02/2013	-2	78.921	10.747
ct92-M305Pete-12	120362	25/02/2013	-2	78.93	10.767
ct92-M305Pete-12	120362	25/02/2013	-2	78.921	10.75
ct92-M305Pete-12	120362	25/02/2013	-2	78.916	10.729
ct92-M305Pete-12	120362	25/02/2013	-1	78.911	10.746
ct92-M305Pete-12	120362	25/02/2013	-2	78.917	10.742
ct92-M305Pete-12	120362	25/02/2013	-2	78.918	10.748
ct92-M305Pete-12	120362	25/02/2013	-2	78.91	10.847
ct92-M305Pete-12	120362	25/02/2013	-2	78.907	10.843
ct92-M305Pete-12	120362	25/02/2013	-2	78.907	10.843
ct92-M305Pete-12	120362	25/02/2013	-1	78.885	10.9
ct92-M305Pete-12	120362	25/02/2013	-2	78.885	10.899
ct92-M305Pete-12	120362	25/02/2013	-2	78.884	10.954
ct92-M305Pete-12	120362	25/02/2013	-2	78.885	10.888
ct92-M305Pete-12	120362	25/02/2013	-2	78.885	10.893
ct92-M305Pete-12	120362	25/02/2013	-2	78.898	10.955
ct92-M305Pete-12	120362	26/02/2013	-1	78.883	10.976
ct92-M305Pete-12	120362	26/02/2013	-1	78.88	10.982
ct92-M305Pete-12	120362	26/02/2013	-2	78.896	10.853
ct92-M305Pete-12	120362	26/02/2013	-2	78.896	10.856
ct92-M305Pete-12	120362	26/02/2013	-1	78.894	10.885
ct92-M305Pete-12	120362	26/02/2013	-2	78.896	10.847
ct92-M305Pete-12	120362	26/02/2013	-2	78.896	10.817
ct92-M305Pete-12	120362	26/02/2013	-2	78.895	10.825

ct92-M305Pete-12	120362	26/02/2013	-2	78.897	10.863
ct92-M305Pete-12	120362	26/02/2013	-2	78.894	10.872
ct92-M305Pete-12	120362	26/02/2013	-2	78.894	10.869
ct92-M305Pete-12	120362	26/02/2013	-2	78.894	10.869
ct92-M305Pete-12	120362	26/02/2013	-2	78.889	10.868
ct92-M305Pete-12	120362	26/02/2013	-2	78.9	10.86
ct92-M305Pete-12	120362	26/02/2013	-2	78.901	10.858
ct92-M305Pete-12	120362	26/02/2013	-2	78.906	10.895
ct92-M305Pete-12	120362	26/02/2013	-2	78.869	10.854
ct92-M305Pete-12	120362	26/02/2013	-2	78.89	10.716
ct92-M305Pete-12	120362	26/02/2013	-2	78.896	10.709
ct92-M305Pete-12	120362	26/02/2013	-2	78.908	10.681
ct92-M305Pete-12	120362	26/02/2013	-1	78.909	10.61
ct92-M305Pete-12	120362	26/02/2013	-1	78.901	10.584
ct92-M305Pete-12	120362	26/02/2013	-2	78.865	10.743
ct92-M305Pete-12	120362	26/02/2013	-2	78.874	10.684
ct92-M305Pete-12	120362	26/02/2013	-2	78.866	10.729
ct92-M305Pete-12	120362	26/02/2013	-1	78.893	10.558
ct92-M305Pete-12	120362	26/02/2013	-1	78.89	10.544
ct92-M305Pete-12	120362	26/02/2013	-2	78.891	10.532
ct92-M305Pete-12	120362	26/02/2013	-2	78.856	10.733
ct92-M305Pete-12	120362	26/02/2013	-2	78.859	10.728
ct92-M305Pete-12	120362	26/02/2013	-2	78.892	10.463
ct92-M305Pete-12	120362	26/02/2013	-1	78.877	10.587
ct92-M305Pete-12	120362	26/02/2013	-1	78.914	10.639
ct92-M305Pete-12	120362	26/02/2013	-1	78.913	10.648
ct92-M305Pete-12	120362	26/02/2013	0	78.921	10.608
ct92-M305Pete-12	120362	26/02/2013	-2	78.914	10.652
ct92-M305Pete-12	120362	26/02/2013	-1	78.92	10.686
ct92-M305Pete-12	120362	26/02/2013	0	78.915	10.596
ct92-M305Pete-12	120362	26/02/2013	-1	78.917	10.627
ct92-M305Pete-12	120362	26/02/2013	-1	78.918	10.627
ct92-M305Pete-12	120362	26/02/2013	-1	78.913	10.676
ct92-M305Pete-12	120362	26/02/2013	-1	78.911	10.6
ct92-M305Pete-12	120362	26/02/2013	-2	78.916	10.689
ct92-M305Pete-12	120362	26/02/2013	-2	78.923	10.504
ct92-M305Pete-12	120362	26/02/2013	-2	78.921	10.504
ct92-M305Pete-12	120362	26/02/2013	-1	78.926	10.505
ct92-M305Pete-12	120362	26/02/2013	-1	78.913	10.502
ct92-M305Pete-12	120362	26/02/2013	-2	78.917	10.487
ct92-M305Pete-12	120362	26/02/2013	-2	78.914	10.494
ct92-M305Pete-12	120362	26/02/2013	-2	78.918	10.5
ct92-M305Pete-12	120362	26/02/2013	-2	78.914	10.512
ct92-M305Pete-12	120362	26/02/2013	-2	78.921	10.495
ct92-M305Pete-12	120362	26/02/2013	-2	78.923	10.483
ct92-M305Pete-12	120362	26/02/2013	-1	78.908	10.578
ct92-M305Pete-12	120362	26/02/2013	-1	78.949	10.524
ct92-M305Pete-12	120362	26/02/2013	-2	78.94	10.574
ct92-M305Pete-12	120362	26/02/2013	-2	78.949	10.517
ct92-M305Pete-12	120362	26/02/2013	-2	78.923	10.641
ct92-M305Pete-12	120362	26/02/2013	-2	78.941	10.573
ct92-M305Pete-12	120362	26/02/2013	-2	78.937	10.562
ct92-M305Pete-12	120362	26/02/2013	-2	78.934	10.592
ct92-M305Pete-12	120362	26/02/2013	-2	78.932	10.608
ct92-M305Pete-12	120362	26/02/2013	-2	78.949	10.602
ct92-M305Pete-12	120362	26/02/2013	-2	78.93	10.589
ct92-M305Pete-12	120362	26/02/2013	-2	78.93	10.597
ct92-M305Pete-12	120362	26/02/2013	-2	78.929	10.604
ct92-M305Pete-12	120362	26/02/2013	-1	78.933	10.567
ct92-M305Pete-12	120362	27/02/2013	-1	78.93	10.6
ct92-M305Pete-12	120362	27/02/2013	-2	78.928	10.603
ct92-M305Pete-12	120362	27/02/2013	-2	78.94	10.589
ct92-M305Pete-12	120362	27/02/2013	-2	78.919	10.592
ct92-M305Pete-12	120362	27/02/2013	-2	78.914	10.608
ct92-M305Pete-12	120362	27/02/2013	-2	78.908	10.529
ct92-M305Pete-12	120362	27/02/2013	-2	78.91	10.528
ct92-M305Pete-12	120362	27/02/2013	-2	78.914	10.601
ct92-M305Pete-12	120362	27/02/2013	-2	78.921	10.576

ct92-M305Pete-12	120362	27/02/2013	-2	78.919	10.563
ct92-M305Pete-12	120362	27/02/2013	-2	78.944	10.607
ct92-M305Pete-12	120362	27/02/2013	-2	78.943	10.617
ct92-M305Pete-12	120362	27/02/2013	-2	78.941	10.604
ct92-M305Pete-12	120362	27/02/2013	-2	78.942	10.603
ct92-M305Pete-12	120362	27/02/2013	-1	78.926	10.549
ct92-M305Pete-12	120362	27/02/2013	-2	78.933	10.562
ct92-M305Pete-12	120362	27/02/2013	-2	78.932	10.564
ct92-M305Pete-12	120362	27/02/2013	-2	78.932	10.568
ct92-M305Pete-12	120362	27/02/2013	-2	78.931	10.565
ct92-M305Pete-12	120362	27/02/2013	-2	78.931	10.619
ct92-M305Pete-12	120362	27/02/2013	-2	78.925	10.563
ct92-M305Pete-12	120362	27/02/2013	-2	78.923	10.567
ct92-M305Pete-12	120362	27/02/2013	-2	78.923	10.572
ct92-M305Pete-12	120362	27/02/2013	-2	78.91	10.641
ct92-M305Pete-12	120362	27/02/2013	-2	78.935	10.558
ct92-M305Pete-12	120362	27/02/2013	-2	78.91	10.642
ct92-M305Pete-12	120362	27/02/2013	-2	78.927	10.538
ct92-M305Pete-12	120362	27/02/2013	-2	78.923	10.576
ct92-M305Pete-12	120362	27/02/2013	-2	78.923	10.575
ct92-M305Pete-12	120362	27/02/2013	-2	78.94	10.692
ct92-M305Pete-12	120362	27/02/2013	-2	78.934	10.695
ct92-M305Pete-12	120362	27/02/2013	-2	78.919	10.682
ct92-M305Pete-12	120362	27/02/2013	-2	78.928	10.71
ct92-M305Pete-12	120362	27/02/2013	-2	78.925	10.686
ct92-M305Pete-12	120362	27/02/2013	-2	78.923	10.703
ct92-M305Pete-12	120362	27/02/2013	-2	78.942	10.733
ct92-M305Pete-12	120362	27/02/2013	-2	78.934	10.701
ct92-M305Pete-12	120362	27/02/2013	-2	78.923	10.693
ct92-M305Pete-12	120362	27/02/2013	-1	78.924	10.733
ct92-M305Pete-12	120362	27/02/2013	-2	78.924	10.777
ct92-M305Pete-12	120362	27/02/2013	-2	78.919	10.678
ct92-M305Pete-12	120362	27/02/2013	-1	78.92	10.681
ct92-M305Pete-12	120362	27/02/2013	-1	78.911	10.683
ct92-M305Pete-12	120362	27/02/2013	-2	78.912	10.691
ct92-M305Pete-12	120362	27/02/2013	-2	78.936	10.8
ct92-M305Pete-12	120362	27/02/2013	-2	78.921	10.716
ct92-M305Pete-12	120362	27/02/2013	-2	78.936	10.805
ct92-M305Pete-12	120362	27/02/2013	-1	78.923	10.692
ct92-M305Pete-12	120362	27/02/2013	-1	78.908	10.834
ct92-M305Pete-12	120362	27/02/2013	-1	78.923	10.754
ct92-M305Pete-12	120362	27/02/2013	-2	78.909	10.698
ct92-M305Pete-12	120362	27/02/2013	-2	78.907	10.707
ct92-M305Pete-12	120362	27/02/2013	-2	78.908	10.725
ct92-M305Pete-12	120362	27/02/2013	-2	78.896	10.72
ct92-M305Pete-12	120362	27/02/2013	-1	78.886	10.839
ct92-M305Pete-12	120362	27/02/2013	-1	78.906	10.704
ct92-M305Pete-12	120362	27/02/2013	-2	78.889	10.836
ct92-M305Pete-12	120362	27/02/2013	-2	78.898	10.905
ct92-M305Pete-12	120362	27/02/2013	-1	78.902	10.718
ct92-M305Pete-12	120362	27/02/2013	-2	78.883	10.851
ct92-M305Pete-12	120362	27/02/2013	-2	78.885	10.854
ct92-M305Pete-12	120362	27/02/2013	-2	78.886	10.854
ct92-M305Pete-12	120362	27/02/2013	-2	78.878	10.865
ct92-M305Pete-12	120362	27/02/2013	-2	78.879	10.858
ct92-M305Pete-12	120362	27/02/2013	-1	78.899	10.843
ct92-M305Pete-12	120362	27/02/2013	-2	78.888	10.832
ct92-M305Pete-12	120362	27/02/2013	-2	78.883	10.831
ct92-M305Pete-12	120362	27/02/2013	-2	78.884	10.772
ct92-M305Pete-12	120362	28/02/2013	-2	78.901	10.769
ct92-M305Pete-12	120362	28/02/2013	-2	78.904	10.806
ct92-M305Pete-12	120362	28/02/2013	-2	78.903	10.801
ct92-M305Pete-12	120362	28/02/2013	-2	78.91	10.767
ct92-M305Pete-12	120362	28/02/2013	-2	78.91	10.768
ct92-M305Pete-12	120362	28/02/2013	-2	78.911	10.774
ct92-M305Pete-12	120362	28/02/2013	-2	78.916	10.771
ct92-M305Pete-12	120362	28/02/2013	-2	78.915	10.757
ct92-M305Pete-12	120362	28/02/2013	-1	78.916	10.733

ct92-M305Pete-12	120362	28/02/2013	-1	78.911	10.639
ct92-M305Pete-12	120362	28/02/2013	-2	78.911	10.66
ct92-M305Pete-12	120362	28/02/2013	-2	78.91	10.648
ct92-M305Pete-12	120362	28/02/2013	-2	78.922	10.733
ct92-M305Pete-12	120362	28/02/2013	-2	78.922	10.74
ct92-M305Pete-12	120362	28/02/2013	0	78.877	11.248
ct92-M305Pete-12	120362	28/02/2013	-2	78.883	11.233
ct92-M305Pete-12	120362	28/02/2013	-1	78.911	10.973
ct92-M305Pete-12	120362	28/02/2013	-2	78.872	10.685
ct92-M305Pete-12	120362	28/02/2013	-1	78.873	10.667
ct92-M305Pete-12	120362	28/02/2013	-2	78.901	10.671
ct92-M305Pete-12	120362	28/02/2013	-2	78.933	10.56
ct92-M305Pete-12	120362	28/02/2013	-2	78.902	10.537
ct92-M305Pete-12	120362	28/02/2013	-2	78.914	10.658
ct92-M305Pete-12	120362	28/02/2013	-2	78.905	10.484
ct92-M305Pete-12	120362	28/02/2013	-2	78.893	10.613
ct92-M305Pete-12	120362	28/02/2013	-2	78.902	10.577
ct92-M305Pete-12	120362	28/02/2013	-2	78.938	10.6
ct92-M305Pete-12	120362	28/02/2013	-1	78.936	10.611
ct92-M305Pete-12	120362	28/02/2013	-1	78.937	10.601
ct92-M305Pete-12	120362	28/02/2013	-2	78.937	10.601
ct92-M305Pete-12	120362	28/02/2013	-1	78.943	10.581
ct92-M305Pete-12	120362	28/02/2013	-1	78.886	10.626
ct92-M305Pete-12	120362	28/02/2013	-2	78.927	10.655
ct92-M305Pete-12	120362	28/02/2013	-2	78.948	10.57
ct92-M305Pete-12	120362	28/02/2013	-1	78.947	10.616
ct92-M305Pete-12	120362	28/02/2013	1	78.949	10.551
ct92-M305Pete-12	120362	28/02/2013	0	78.948	10.613
ct92-M305Pete-12	120362	28/02/2013	-2	78.973	10.682
ct92-M305Pete-12	120362	28/02/2013	-2	79.006	10.558
ct92-M305Pete-12	120362	28/02/2013	-2	79.005	10.556
ct92-M305Pete-12	120362	28/02/2013	-2	78.977	10.516
ct92-M305Pete-12	120362	28/02/2013	-2	78.962	10.531
ct92-M305Pete-12	120362	28/02/2013	-2	78.981	10.519
ct92-M305Pete-12	120362	28/02/2013	-2	78.946	10.614
ct92-M305Pete-12	120362	28/02/2013	-2	78.929	10.562
ct92-M305Pete-12	120362	28/02/2013	-2	78.925	10.584
ct92-M305Pete-12	120362	28/02/2013	-2	78.927	10.561
ct92-M305Pete-12	120362	28/02/2013	-1	78.927	10.582
ct92-M305Pete-12	120362	28/02/2013	-2	78.947	10.556
ct92-M305Pete-12	120362	28/02/2013	-2	78.931	10.54
ct92-M305Pete-12	120362	28/02/2013	-2	78.959	10.527
ct92-M305Pete-12	120362	28/02/2013	-2	78.957	10.516
ct92-M305Pete-12	120362	28/02/2013	-2	78.933	10.516
ct92-M305Pete-12	120362	28/02/2013	-1	78.94	10.53
ct92-M305Pete-12	120362	28/02/2013	-2	78.97	10.453
ct92-M305Pete-12	120362	28/02/2013	-2	78.971	10.482
ct92-M305Pete-12	120362	28/02/2013	-2	78.958	10.464
ct92-M305Pete-12	120362	28/02/2013	-2	78.953	10.446
ct92-M305Pete-12	120362	28/02/2013	-2	78.952	10.442
ct92-M305Pete-12	120362	28/02/2013	-2	78.972	10.454
ct92-M305Pete-12	120362	28/02/2013	-2	78.952	10.462
ct92-M305Pete-12	120362	28/02/2013	-2	78.978	10.493
ct92-M305Pete-12	120362	28/02/2013	-2	78.923	10.351
ct92-M305Pete-12	120362	28/02/2013	-1	78.934	10.306
ct92-M305Pete-12	120362	28/02/2013	-1	78.947	10.36
ct92-M305Pete-12	120362	28/02/2013	-2	78.922	10.439
ct92-M305Pete-12	120362	28/02/2013	-1	78.903	10.379
ct92-M305Pete-12	120362	28/02/2013	-2	78.903	10.357
ct92-M305Pete-12	120362	28/02/2013	-2	78.914	10.603
ct92-M305Pete-12	120362	28/02/2013	-1	78.907	10.446
ct92-M305Pete-12	120362	28/02/2013	-2	78.908	10.461
ct92-M305Pete-12	120362	28/02/2013	-2	78.91	10.507
ct92-M305Pete-12	120362	28/02/2013	-1	78.908	10.537
ct92-M305Pete-12	120362	28/02/2013	-2	78.92	10.571
ct92-M305Pete-12	120362	28/02/2013	-2	78.92	10.569
ct92-M305Pete-12	120362	28/02/2013	-2	78.924	10.59
ct92-M305Pete-12	120362	01/03/2013	-2	78.921	10.582

ct92-M305Pete-12	120362	01/03/2013	-1	78.918	10.638
ct92-M305Pete-12	120362	01/03/2013	-2	78.912	10.605
ct92-M305Pete-12	120362	01/03/2013	-2	78.903	10.487
ct92-M305Pete-12	120362	01/03/2013	-2	78.913	10.516
ct92-M305Pete-12	120362	01/03/2013	-2	78.921	10.503
ct92-M305Pete-12	120362	01/03/2013	-2	78.912	10.489
ct92-M305Pete-12	120362	01/03/2013	-2	78.913	10.508
ct92-M305Pete-12	120362	01/03/2013	-2	78.9	10.489
ct92-M305Pete-12	120362	01/03/2013	-2	78.902	10.573
ct92-M305Pete-12	120362	01/03/2013	-2	78.896	10.546
ct92-M305Pete-12	120362	01/03/2013	-2	78.891	10.569
ct92-M305Pete-12	120362	01/03/2013	-2	78.889	10.544
ct92-M305Pete-12	120362	01/03/2013	-2	78.887	10.545
ct92-M305Pete-12	120362	01/03/2013	-2	78.893	10.554
ct92-M305Pete-12	120362	01/03/2013	-2	78.894	10.484
ct92-M305Pete-12	120362	01/03/2013	-2	78.88	10.522
ct92-M305Pete-12	120362	01/03/2013	-2	78.881	10.528
ct92-M305Pete-12	120362	01/03/2013	-1	78.895	10.731
ct92-M305Pete-12	120362	01/03/2013	-1	78.906	10.565
ct92-M305Pete-12	120362	01/03/2013	-2	78.906	10.612
ct92-M305Pete-12	120362	01/03/2013	-2	78.907	10.65
ct92-M305Pete-12	120362	01/03/2013	-1	78.903	10.693
ct92-M305Pete-12	120362	01/03/2013	-2	78.904	10.696
ct92-M305Pete-12	120362	01/03/2013	-2	78.908	10.704
ct92-M305Pete-12	120362	01/03/2013	-2	78.912	10.662
ct92-M305Pete-12	120362	01/03/2013	-2	78.906	10.732
ct92-M305Pete-12	120362	01/03/2013	-2	78.899	10.704
ct92-M305Pete-12	120362	01/03/2013	-2	78.924	10.7
ct92-M305Pete-12	120362	01/03/2013	-1	78.912	10.77
ct92-M305Pete-12	120362	01/03/2013	-1	78.912	10.747
ct92-M305Pete-12	120362	01/03/2013	-2	78.912	10.746
ct92-M305Pete-12	120362	01/03/2013	-2	78.91	10.788
ct92-M305Pete-12	120362	01/03/2013	-2	78.897	10.734
ct92-M305Pete-12	120362	01/03/2013	-2	78.889	10.695
ct92-M305Pete-12	120362	01/03/2013	-2	78.917	10.75
ct92-M305Pete-12	120362	01/03/2013	-2	78.897	10.741
ct92-M305Pete-12	120362	01/03/2013	-2	78.897	10.738
ct92-M305Pete-12	120362	01/03/2013	-2	78.901	10.752
ct92-M305Pete-12	120362	01/03/2013	-2	78.904	10.756
ct92-M305Pete-12	120362	01/03/2013	-2	78.904	10.752
ct92-M305Pete-12	120362	01/03/2013	-2	78.905	10.779
ct92-M305Pete-12	120362	01/03/2013	-2	78.896	10.776
ct92-M305Pete-12	120362	01/03/2013	-2	78.92	10.818
ct92-M305Pete-12	120362	01/03/2013	-2	78.907	10.862
ct92-M305Pete-12	120362	01/03/2013	-1	78.907	10.934
ct92-M305Pete-12	120362	01/03/2013	-2	78.904	10.912
ct92-M305Pete-12	120362	01/03/2013	-1	78.893	10.861
ct92-M305Pete-12	120362	01/03/2013	-2	78.9	10.874
ct92-M305Pete-12	120362	01/03/2013	-2	78.9	10.867
ct92-M305Pete-12	120362	01/03/2013	-2	78.913	10.95
ct92-M305Pete-12	120362	01/03/2013	-2	78.9	10.86
ct92-M305Pete-12	120362	01/03/2013	-2	78.904	10.85
ct92-M305Pete-12	120362	01/03/2013	-2	78.907	10.886
ct92-M305Pete-12	120362	01/03/2013	-2	78.894	10.902
ct92-M305Pete-12	120362	01/03/2013	-2	78.887	10.977
ct92-M305Pete-12	120362	01/03/2013	-2	78.877	10.96
ct92-M305Pete-12	120362	01/03/2013	-1	78.881	10.863
ct92-M305Pete-12	120362	01/03/2013	-2	78.881	10.909
ct92-M305Pete-12	120362	01/03/2013	-2	78.881	10.905
ct92-M305Pete-12	120362	01/03/2013	-2	78.881	10.895
ct92-M305Pete-12	120362	01/03/2013	-2	78.885	10.86
ct92-M305Pete-12	120362	01/03/2013	-2	78.897	10.936
ct92-M305Pete-12	120362	01/03/2013	-2	78.876	10.677
ct92-M305Pete-12	120362	01/03/2013	-2	78.883	10.695
ct92-M305Pete-12	120362	01/03/2013	-2	78.881	10.703
ct92-M305Pete-12	120362	01/03/2013	-2	78.881	10.701
ct92-M305Pete-12	120362	02/03/2013	-2	78.878	10.689
ct92-M305Pete-12	120362	02/03/2013	-1	78.873	10.692

ct92-M305Pete-12	120362	02/03/2013	-2	78.874	10.696
ct92-M305Pete-12	120362	02/03/2013	-2	78.885	10.779
ct92-M305Pete-12	120362	02/03/2013	-2	78.876	10.886
ct92-M305Pete-12	120362	02/03/2013	-2	78.888	10.763
ct92-M305Pete-12	120362	02/03/2013	-2	78.888	10.766
ct92-M305Pete-12	120362	02/03/2013	-2	78.888	10.765
ct92-M305Pete-12	120362	02/03/2013	-2	78.887	10.759
ct92-M305Pete-12	120362	02/03/2013	-2	78.891	10.731
ct92-M305Pete-12	120362	02/03/2013	-2	78.894	10.856
ct92-M305Pete-12	120362	02/03/2013	-2	78.895	10.86
ct92-M305Pete-12	120362	02/03/2013	-2	78.901	10.817
ct92-M305Pete-12	120362	02/03/2013	-2	78.9	10.816
ct92-M305Pete-12	120362	02/03/2013	-2	78.894	10.891
ct92-M305Pete-12	120362	02/03/2013	-2	78.891	10.854
ct92-M305Pete-12	120362	02/03/2013	-2	78.892	10.85
ct92-M305Pete-12	120362	02/03/2013	-2	78.891	10.848
ct92-M305Pete-12	120362	02/03/2013	-2	78.892	10.857
ct92-M305Pete-12	120362	02/03/2013	-2	78.904	10.879
ct92-M305Pete-12	120362	02/03/2013	-2	78.905	10.88
ct92-M305Pete-12	120362	02/03/2013	-2	78.911	10.825
ct92-M305Pete-12	120362	02/03/2013	-2	78.913	10.82
ct92-M305Pete-12	120362	02/03/2013	-2	78.898	10.72
ct92-M305Pete-12	120362	02/03/2013	-2	78.917	10.818
ct92-M305Pete-12	120362	02/03/2013	-2	78.916	10.813
ct92-M305Pete-12	120362	02/03/2013	-2	78.916	10.814
ct92-M305Pete-12	120362	02/03/2013	-2	78.915	10.875
ct92-M305Pete-12	120362	02/03/2013	-2	78.919	10.715
ct92-M305Pete-12	120362	02/03/2013	-2	78.902	10.723
ct92-M305Pete-12	120362	02/03/2013	-2	78.9	10.723
ct92-M305Pete-12	120362	02/03/2013	-2	78.898	10.714
ct92-M305Pete-12	120362	02/03/2013	-2	78.896	10.703
ct92-M305Pete-12	120362	02/03/2013	-1	78.913	10.556
ct92-M305Pete-12	120362	02/03/2013	-2	78.911	10.719
ct92-M305Pete-12	120362	02/03/2013	-2	78.933	10.584
ct92-M305Pete-12	120362	02/03/2013	-1	78.904	10.549
ct92-M305Pete-12	120362	02/03/2013	-2	78.903	10.552
ct92-M305Pete-12	120362	02/03/2013	-2	78.914	10.569
ct92-M305Pete-12	120362	02/03/2013	-2	78.918	10.544
ct92-M305Pete-12	120362	02/03/2013	-2	78.908	10.538
ct92-M305Pete-12	120362	02/03/2013	-2	78.908	10.538
ct92-M305Pete-12	120362	02/03/2013	0	78.913	10.633
ct92-M305Pete-12	120362	02/03/2013	-2	78.91	10.599
ct92-M305Pete-12	120362	02/03/2013	-2	78.902	10.544
ct92-M305Pete-12	120362	02/03/2013	-2	78.904	10.546
ct92-M305Pete-12	120362	02/03/2013	-2	78.903	10.547
ct92-M305Pete-12	120362	02/03/2013	-2	78.906	10.541
ct92-M305Pete-12	120362	02/03/2013	-2	78.905	10.546
ct92-M305Pete-12	120362	02/03/2013	-1	78.919	10.552
ct92-M305Pete-12	120362	02/03/2013	-2	78.924	10.572
ct92-M305Pete-12	120362	02/03/2013	-2	78.909	10.532
ct92-M305Pete-12	120362	02/03/2013	-1	78.909	10.471
ct92-M305Pete-12	120362	02/03/2013	-1	78.936	10.502
ct92-M305Pete-12	120362	02/03/2013	-2	78.935	10.51
ct92-M305Pete-12	120362	02/03/2013	-2	78.937	10.508
ct92-M305Pete-12	120362	02/03/2013	-2	78.94	10.507
ct92-M305Pete-12	120362	02/03/2013	-1	78.91	10.617
ct92-M305Pete-12	120362	02/03/2013	-2	78.899	10.541
ct92-M305Pete-12	120362	02/03/2013	-1	78.908	10.58
ct92-M305Pete-12	120362	02/03/2013	-2	78.908	10.576
ct92-M305Pete-12	120362	02/03/2013	-2	78.908	10.579
ct92-M305Pete-12	120362	02/03/2013	-2	78.912	10.571
ct92-M305Pete-12	120362	02/03/2013	-2	78.91	10.545
ct92-M305Pete-12	120362	02/03/2013	-2	78.916	10.579
ct92-M305Pete-12	120362	02/03/2013	-2	78.915	10.577
ct92-M305Pete-12	120362	02/03/2013	-2	78.914	10.572
ct92-M305Pete-12	120362	03/03/2013	-2	78.912	10.53
ct92-M305Pete-12	120362	03/03/2013	-2	78.912	10.574
ct92-M305Pete-12	120362	03/03/2013	-2	78.917	10.619

ct92-M305Pete-12	120362	03/03/2013	-1	78.917	10.524
ct92-M305Pete-12	120362	03/03/2013	-2	78.917	10.503
ct92-M305Pete-12	120362	03/03/2013	-2	78.915	10.497
ct92-M305Pete-12	120362	03/03/2013	-1	78.946	10.456
ct92-M305Pete-12	120362	03/03/2013	-2	78.946	10.456
ct92-M305Pete-12	120362	03/03/2013	-2	78.949	10.55
ct92-M305Pete-12	120362	03/03/2013	-2	78.95	10.541
ct92-M305Pete-12	120362	03/03/2013	-2	78.949	10.518
ct92-M305Pete-12	120362	03/03/2013	-2	78.943	10.549
ct92-M305Pete-12	120362	03/03/2013	-2	78.926	10.586
ct92-M305Pete-12	120362	03/03/2013	-2	78.924	10.581
ct92-M305Pete-12	120362	03/03/2013	-2	78.96	10.56
ct92-M305Pete-12	120362	03/03/2013	-2	78.957	10.436
ct92-M305Pete-12	120362	03/03/2013	-2	78.952	10.443
ct92-M305Pete-12	120362	03/03/2013	-2	78.949	10.448
ct92-M305Pete-12	120362	03/03/2013	-2	78.955	10.44
ct92-M305Pete-12	120362	03/03/2013	-2	78.931	10.48
ct92-M305Pete-12	120362	03/03/2013	-2	78.929	10.581
ct92-M305Pete-12	120362	03/03/2013	-2	78.935	10.616
ct92-M305Pete-12	120362	03/03/2013	-1	78.962	10.364
ct92-M305Pete-12	120362	03/03/2013	-2	78.956	10.396
ct92-M305Pete-12	120362	03/03/2013	-2	78.931	10.497
ct92-M305Pete-12	120362	03/03/2013	-2	78.925	10.525
ct92-M305Pete-12	120362	03/03/2013	-2	78.923	10.564
ct92-M305Pete-12	120362	03/03/2013	0	78.912	10.739
ct92-M305Pete-12	120362	03/03/2013	-1	78.95	10.574
ct92-M305Pete-12	120362	03/03/2013	0	78.9	10.568
ct92-M305Pete-12	120362	03/03/2013	-1	78.909	10.556
ct92-M305Pete-12	120362	03/03/2013	-2	78.895	10.621
ct92-M305Pete-12	120362	03/03/2013	-2	78.893	10.604
ct92-M305Pete-12	120362	03/03/2013	-1	78.932	10.538
ct92-M305Pete-12	120362	03/03/2013	-2	78.893	10.551
ct92-M305Pete-12	120362	03/03/2013	-1	78.887	10.457
ct92-M305Pete-12	120362	03/03/2013	-2	78.905	10.482
ct92-M305Pete-12	120362	03/03/2013	-2	78.893	10.465
ct92-M305Pete-12	120362	03/03/2013	-2	78.914	10.54
ct92-M305Pete-12	120362	03/03/2013	-1	78.896	10.551
ct92-M305Pete-12	120362	03/03/2013	-2	78.895	10.525
ct92-M305Pete-12	120362	03/03/2013	-2	78.9	10.68
ct92-M305Pete-12	120362	03/03/2013	-2	78.918	10.648
ct92-M305Pete-12	120362	03/03/2013	-2	78.901	10.645
ct92-M305Pete-12	120362	03/03/2013	-1	78.929	10.595
ct92-M305Pete-12	120362	03/03/2013	-2	78.929	10.625
ct92-M305Pete-12	120362	03/03/2013	-2	78.924	10.716
ct92-M305Pete-12	120362	03/03/2013	-2	78.934	10.66
ct92-M305Pete-12	120362	03/03/2013	-1	78.922	10.593
ct92-M305Pete-12	120362	03/03/2013	-2	78.915	10.586
ct92-M305Pete-12	120362	03/03/2013	-2	78.901	10.574
ct92-M305Pete-12	120362	03/03/2013	-2	78.9	10.635
ct92-M305Pete-12	120362	03/03/2013	-2	78.902	10.657
ct92-M305Pete-12	120362	03/03/2013	-2	78.889	10.497
ct92-M305Pete-12	120362	03/03/2013	-2	78.9	10.642
ct92-M305Pete-12	120362	03/03/2013	-2	78.917	10.669
ct92-M305Pete-12	120362	03/03/2013	-2	78.92	10.667
ct92-M305Pete-12	120362	03/03/2013	-2	78.915	10.682
ct92-M305Pete-12	120362	03/03/2013	-1	78.921	10.667
ct92-M305Pete-12	120362	03/03/2013	-2	78.923	10.677
ct92-M305Pete-12	120362	03/03/2013	-2	78.926	10.687
ct92-M305Pete-12	120362	03/03/2013	-1	78.923	10.641
ct92-M305Pete-12	120362	03/03/2013	0	78.936	10.677
ct92-M305Pete-12	120362	03/03/2013	-1	78.918	10.604
ct92-M305Pete-12	120362	03/03/2013	-1	78.946	10.604
ct92-M305Pete-12	120362	03/03/2013	-2	78.967	10.651
ct92-M305Pete-12	120362	04/03/2013	-2	78.983	10.736
ct92-M305Pete-12	120362	04/03/2013	-2	78.922	10.749
ct92-M305Pete-12	120362	04/03/2013	-2	78.914	10.619
ct92-M305Pete-12	120362	04/03/2013	-1	78.928	10.609
ct92-M305Pete-12	120362	04/03/2013	-2	78.909	10.678

ct92-M305Pete-12	120362	04/03/2013	-1	78.918	10.621
ct92-M305Pete-12	120362	04/03/2013	-1	78.927	10.665
ct92-M305Pete-12	120362	04/03/2013	-2	78.941	10.606
ct92-M305Pete-12	120362	04/03/2013	-2	78.934	10.591
ct92-M305Pete-12	120362	04/03/2013	-2	78.919	10.497
ct92-M305Pete-12	120362	04/03/2013	-2	78.909	10.569
ct92-M305Pete-12	120362	04/03/2013	-2	78.909	10.587
ct92-M305Pete-12	120362	04/03/2013	-2	78.92	10.629
ct92-M305Pete-12	120362	04/03/2013	-2	78.915	10.631
ct92-M305Pete-12	120362	04/03/2013	-1	78.919	10.632
ct92-M305Pete-12	120362	04/03/2013	-2	78.902	10.643
ct92-M305Pete-12	120362	04/03/2013	-2	78.9	10.658
ct92-M305Pete-12	120362	04/03/2013	-2	78.901	10.668
ct92-M305Pete-12	120362	04/03/2013	-2	78.915	10.709
ct92-M305Pete-12	120362	04/03/2013	-1	78.915	10.709
ct92-M305Pete-12	120362	04/03/2013	-2	78.914	10.736
ct92-M305Pete-12	120362	04/03/2013	-2	78.937	10.709
ct92-M305Pete-12	120362	04/03/2013	-2	78.915	10.7
ct92-M305Pete-12	120362	04/03/2013	-2	78.902	10.767
ct92-M305Pete-12	120362	04/03/2013	-2	78.903	10.751
ct92-M305Pete-12	120362	04/03/2013	-2	78.903	10.767
ct92-M305Pete-12	120362	04/03/2013	-2	78.9	10.754
ct92-M305Pete-12	120362	04/03/2013	-2	78.905	10.749
ct92-M305Pete-12	120362	04/03/2013	-1	78.914	10.777
ct92-M305Pete-12	120362	04/03/2013	-2	78.915	10.703
ct92-M305Pete-12	120362	04/03/2013	-2	78.914	10.728
ct92-M305Pete-12	120362	04/03/2013	-2	78.917	10.737
ct92-M305Pete-12	120362	04/03/2013	-2	78.916	10.655
ct92-M305Pete-12	120362	04/03/2013	-2	78.925	10.646
ct92-M305Pete-12	120362	04/03/2013	-2	78.925	10.644
ct92-M305Pete-12	120362	04/03/2013	-2	78.926	10.636
ct92-M305Pete-12	120362	04/03/2013	-2	78.925	10.625
ct92-M305Pete-12	120362	04/03/2013	-1	78.958	10.723
ct92-M305Pete-12	120362	04/03/2013	-1	78.896	10.653
ct92-M305Pete-12	120362	04/03/2013	-1	78.912	10.674
ct92-M305Pete-12	120362	04/03/2013	-2	78.923	10.632
ct92-M305Pete-12	120362	04/03/2013	-1	78.9	10.538
ct92-M305Pete-12	120362	04/03/2013	-2	78.898	10.531
ct92-M305Pete-12	120362	04/03/2013	-2	78.894	10.523
ct92-M305Pete-12	120362	04/03/2013	-1	78.909	10.539
ct92-M305Pete-12	120362	04/03/2013	-1	78.917	10.599
ct92-M305Pete-12	120362	04/03/2013	-1	78.916	10.58
ct92-M305Pete-12	120362	04/03/2013	-1	78.906	10.6
ct92-M305Pete-12	120362	04/03/2013	-2	78.907	10.602
ct92-M305Pete-12	120362	04/03/2013	-1	78.9	10.586
ct92-M305Pete-12	120362	04/03/2013	-1	78.907	10.53
ct92-M305Pete-12	120362	04/03/2013	-1	78.911	10.551
ct92-M305Pete-12	120362	04/03/2013	-2	78.917	10.6
ct92-M305Pete-12	120362	04/03/2013	-1	78.891	10.561
ct92-M305Pete-12	120362	04/03/2013	-1	78.904	10.561
ct92-M305Pete-12	120362	04/03/2013	-2	78.886	10.545
ct92-M305Pete-12	120362	04/03/2013	-2	78.92	10.727
ct92-M305Pete-12	120362	04/03/2013	-2	78.883	10.677
ct92-M305Pete-12	120362	04/03/2013	-2	78.901	10.689
ct92-M305Pete-12	120362	04/03/2013	-2	78.888	10.553
ct92-M305Pete-12	120362	04/03/2013	-2	78.891	10.51
ct92-M305Pete-12	120362	04/03/2013	-2	78.894	10.517
ct92-M305Pete-12	120362	04/03/2013	-2	78.88	10.533
ct92-M305Pete-12	120362	04/03/2013	-2	78.916	10.698
ct92-M305Pete-12	120362	04/03/2013	-2	78.912	10.711
ct92-M305Pete-12	120362	04/03/2013	-2	78.915	10.632
ct92-M305Pete-12	120362	04/03/2013	-2	78.915	10.635
ct92-M305Pete-12	120362	05/03/2013	-2	78.94	10.786
ct92-M305Pete-12	120362	05/03/2013	-1	78.933	10.711
ct92-M305Pete-12	120362	05/03/2013	-2	78.911	10.618
ct92-M305Pete-12	120362	05/03/2013	-2	78.912	10.61
ct92-M305Pete-12	120362	05/03/2013	-2	78.924	10.641
ct92-M305Pete-12	120362	05/03/2013	-2	78.917	10.617

ct92-M305Pete-12	120362	05/03/2013	-2	78.924	10.637
ct92-M305Pete-12	120362	05/03/2013	-2	78.931	10.652
ct92-M305Pete-12	120362	05/03/2013	-2	78.929	10.652
ct92-M305Pete-12	120362	05/03/2013	-2	78.911	10.618
ct92-M305Pete-12	120362	05/03/2013	-1	78.919	10.511
ct92-M305Pete-12	120362	05/03/2013	-2	78.919	10.525
ct92-M305Pete-12	120362	05/03/2013	-1	78.926	10.588
ct92-M305Pete-12	120362	05/03/2013	-1	78.902	10.581
ct92-M305Pete-12	120362	05/03/2013	-2	78.904	10.588
ct92-M305Pete-12	120362	05/03/2013	-1	78.915	10.613
ct92-M305Pete-12	120362	05/03/2013	-2	78.927	10.635
ct92-M305Pete-12	120362	05/03/2013	-2	78.915	10.64
ct92-M305Pete-12	120362	05/03/2013	-1	78.911	10.651
ct92-M305Pete-12	120362	05/03/2013	-2	78.899	10.65
ct92-M305Pete-12	120362	05/03/2013	-2	78.899	10.663
ct92-M305Pete-12	120362	05/03/2013	-1	78.898	10.671
ct92-M305Pete-12	120362	05/03/2013	-2	78.912	10.61
ct92-M305Pete-12	120362	05/03/2013	-2	78.905	10.58
ct92-M305Pete-12	120362	05/03/2013	-1	78.909	10.602
ct92-M305Pete-12	120362	05/03/2013	-2	78.926	10.652
ct92-M305Pete-12	120362	05/03/2013	-2	78.93	10.668
ct92-M305Pete-12	120362	05/03/2013	-2	78.896	10.68
ct92-M305Pete-12	120362	05/03/2013	-2	78.935	10.675
ct92-M305Pete-12	120362	05/03/2013	-1	78.907	10.526
ct92-M305Pete-12	120362	05/03/2013	-1	78.905	10.661
ct92-M305Pete-12	120362	05/03/2013	-2	78.915	10.776
ct92-M305Pete-12	120362	05/03/2013	-2	78.925	10.801
ct92-M305Pete-12	120362	05/03/2013	-2	78.91	10.688
ct92-M305Pete-12	120362	05/03/2013	-2	78.905	10.56
ct92-M305Pete-12	120362	05/03/2013	-1	78.914	10.693
ct92-M305Pete-12	120362	05/03/2013	-2	78.924	10.732
ct92-M305Pete-12	120362	05/03/2013	-2	78.911	10.613
ct92-M305Pete-12	120362	05/03/2013	-2	78.905	10.714
ct92-M305Pete-12	120362	05/03/2013	-2	78.901	10.656
ct92-M305Pete-12	120362	05/03/2013	-2	78.916	10.694
ct92-M305Pete-12	120362	05/03/2013	-1	78.914	10.678
ct92-M305Pete-12	120362	05/03/2013	-2	78.919	10.706
ct92-M305Pete-12	120362	05/03/2013	-2	78.907	10.669
ct92-M305Pete-12	120362	05/03/2013	-1	78.91	10.643
ct92-M305Pete-12	120362	05/03/2013	-1	78.924	10.68
ct92-M305Pete-12	120362	05/03/2013	1	78.92	10.637
ct92-M305Pete-12	120362	05/03/2013	-2	78.922	10.65
ct92-M305Pete-12	120362	05/03/2013	-2	78.922	10.597
ct92-M305Pete-12	120362	05/03/2013	-2	78.921	10.649
ct92-M305Pete-12	120362	05/03/2013	-2	78.921	10.598
ct92-M305Pete-12	120362	05/03/2013	-1	78.921	10.597
ct92-M305Pete-12	120362	05/03/2013	-2	78.923	10.598
ct92-M305Pete-12	120362	05/03/2013	-2	78.924	10.59
ct92-M305Pete-12	120362	05/03/2013	-2	78.923	10.596
ct92-M305Pete-12	120362	05/03/2013	-2	78.924	10.593
ct92-M305Pete-12	120362	05/03/2013	-2	78.928	10.573
ct92-M305Pete-12	120362	05/03/2013	-2	78.921	10.597
ct92-M305Pete-12	120362	06/03/2013	-2	78.93	10.564
ct92-M305Pete-12	120362	06/03/2013	-2	78.929	10.558
ct92-M305Pete-12	120362	06/03/2013	-2	78.926	10.554
ct92-M305Pete-12	120362	06/03/2013	-2	78.929	10.56
ct92-M305Pete-12	120362	06/03/2013	-2	78.918	10.488
ct92-M305Pete-12	120362	06/03/2013	-2	78.917	10.5
ct92-M305Pete-12	120362	06/03/2013	-2	78.917	10.491
ct92-M305Pete-12	120362	06/03/2013	-2	78.916	10.551
ct92-M305Pete-12	120362	06/03/2013	-2	78.931	10.547
ct92-M305Pete-12	120362	06/03/2013	-2	78.927	10.593
ct92-M305Pete-12	120362	06/03/2013	-2	78.898	10.861
ct92-M305Pete-12	120362	06/03/2013	-2	78.892	10.832
ct92-M305Pete-12	120362	06/03/2013	-2	78.897	10.862
ct92-M305Pete-12	120362	06/03/2013	-2	78.915	10.467
ct92-M305Pete-12	120362	06/03/2013	-2	78.927	10.541
ct92-M305Pete-12	120362	06/03/2013	-2	78.875	10.616

ct92-M305Pete-12	120362	06/03/2013	-2	78.876	10.557
ct92-M305Pete-12	120362	06/03/2013	-2	78.875	10.555
ct92-M305Pete-12	120362	06/03/2013	-2	78.887	10.592
ct92-M305Pete-12	120362	06/03/2013	-2	78.963	10.44
ct92-M305Pete-12	120362	06/03/2013	-2	78.917	10.491
ct92-M305Pete-12	120362	06/03/2013	-2	78.949	10.482
ct92-M305Pete-12	120362	06/03/2013	-2	78.894	10.582
ct92-M305Pete-12	120362	06/03/2013	-2	78.919	10.556
ct92-M305Pete-12	120362	06/03/2013	-2	78.926	10.588
ct92-M305Pete-12	120362	06/03/2013	-2	78.929	10.573
ct92-M305Pete-12	120362	06/03/2013	-2	78.915	10.662
ct92-M305Pete-12	120362	06/03/2013	-2	78.919	10.663
ct92-M305Pete-12	120362	06/03/2013	-2	78.918	10.662
ct92-M305Pete-12	120362	06/03/2013	-2	78.952	10.607
ct92-M305Pete-12	120362	06/03/2013	-2	78.925	10.69
ct92-M305Pete-12	120362	06/03/2013	-2	78.924	10.689
ct92-M305Pete-12	120362	06/03/2013	-2	78.929	10.594
ct92-M305Pete-12	120362	06/03/2013	-2	78.927	10.665
ct92-M305Pete-12	120362	06/03/2013	-1	78.927	10.679
ct92-M305Pete-12	120362	06/03/2013	-1	78.913	10.6
ct92-M305Pete-12	120362	06/03/2013	-2	78.921	10.579
ct92-M305Pete-12	120362	06/03/2013	-1	78.951	10.719
ct92-M305Pete-12	120362	06/03/2013	-2	78.938	10.419
ct92-M305Pete-12	120362	06/03/2013	-2	78.906	10.428
ct92-M305Pete-12	120362	06/03/2013	-2	78.91	10.449
ct92-M305Pete-12	120362	06/03/2013	-2	78.908	10.418
ct92-M305Pete-12	120362	06/03/2013	-2	78.919	10.467
ct92-M305Pete-12	120362	06/03/2013	-1	78.911	10.569
ct92-M305Pete-12	120362	06/03/2013	-2	78.914	10.562
ct92-M305Pete-12	120362	06/03/2013	-2	78.915	10.565
ct92-M305Pete-12	120362	06/03/2013	-2	78.915	10.566
ct92-M305Pete-12	120362	06/03/2013	-2	78.917	10.561
ct92-M305Pete-12	120362	06/03/2013	-2	78.929	10.587
ct92-M305Pete-12	120362	06/03/2013	-2	78.929	10.587
ct92-M305Pete-12	120362	06/03/2013	-2	78.928	10.588
ct92-M305Pete-12	120362	06/03/2013	-2	78.928	10.614
ct92-M305Pete-12	120362	06/03/2013	-2	78.939	10.661
ct92-M305Pete-12	120362	06/03/2013	-2	78.942	10.675
ct92-M305Pete-12	120362	06/03/2013	-2	78.932	10.617
ct92-M305Pete-12	120362	07/03/2013	-2	78.936	10.586
ct92-M305Pete-12	120362	07/03/2013	-2	78.94	10.515
ct92-M305Pete-12	120362	07/03/2013	-2	78.94	10.504
ct92-M305Pete-12	120362	07/03/2013	-2	78.94	10.555
ct92-M305Pete-12	120362	07/03/2013	-2	78.941	10.674
ct92-M305Pete-12	120362	07/03/2013	-2	78.939	10.669
ct92-M305Pete-12	120362	07/03/2013	-2	78.939	10.664
ct92-M305Pete-12	120362	07/03/2013	-2	78.938	10.655
ct92-M305Pete-12	120362	07/03/2013	-2	78.94	10.654
ct92-M305Pete-12	120362	07/03/2013	-2	78.937	10.664
ct92-M305Pete-12	120362	07/03/2013	-1	78.93	10.59
ct92-M305Pete-12	120362	07/03/2013	-1	78.919	10.596
ct92-M305Pete-12	120362	07/03/2013	0	78.879	10.8
ct92-M305Pete-12	120362	07/03/2013	-2	78.904	10.734
ct92-M305Pete-12	120362	07/03/2013	-1	78.914	10.649
ct92-M305Pete-12	120362	07/03/2013	-2	78.907	10.686
ct92-M305Pete-12	120362	07/03/2013	-1	78.909	10.68
ct92-M305Pete-12	120362	07/03/2013	-2	78.904	10.683
ct92-M305Pete-12	120362	07/03/2013	-2	78.908	10.681
ct92-M305Pete-12	120362	07/03/2013	-1	78.906	10.686
ct92-M305Pete-12	120362	07/03/2013	-2	78.912	10.575
ct92-M305Pete-12	120362	07/03/2013	-2	78.92	10.605
ct92-M305Pete-12	120362	07/03/2013	-2	78.912	10.576
ct92-M305Pete-12	120362	07/03/2013	-1	78.901	10.602
ct92-M305Pete-12	120362	07/03/2013	-1	78.909	10.665
ct92-M305Pete-12	120362	07/03/2013	-2	78.904	10.643
ct92-M305Pete-12	120362	07/03/2013	-2	78.901	10.685
ct92-M305Pete-12	120362	07/03/2013	-2	78.911	10.708
ct92-M305Pete-12	120362	07/03/2013	-2	78.908	10.713

ct92-M305Pete-12	120362	07/03/2013	-2	78.909	10.704
ct92-M305Pete-12	120362	07/03/2013	-2	78.902	10.668
ct92-M305Pete-12	120362	07/03/2013	-2	78.906	10.679
ct92-M305Pete-12	120362	07/03/2013	-2	78.898	10.661
ct92-M305Pete-12	120362	07/03/2013	-2	78.92	10.698
ct92-M305Pete-12	120362	07/03/2013	-2	78.92	10.7
ct92-M305Pete-12	120362	07/03/2013	-2	78.92	10.707
ct92-M305Pete-12	120362	07/03/2013	-2	78.918	10.713
ct92-M305Pete-12	120362	07/03/2013	-2	78.912	10.735
ct92-M305Pete-12	120362	07/03/2013	-2	78.908	10.737
ct92-M305Pete-12	120362	07/03/2013	-1	78.902	10.651
ct92-M305Pete-12	120362	08/03/2013	-2	78.89	10.626
ct92-M305Pete-12	120362	08/03/2013	-2	78.899	10.682
ct92-M305Pete-12	120362	08/03/2013	-2	78.904	10.728
ct92-M305Pete-12	120362	08/03/2013	-2	78.917	10.741
ct92-M305Pete-12	120362	08/03/2013	-2	78.908	10.749
ct92-M305Pete-12	120362	08/03/2013	-2	78.908	10.748
ct92-M305Pete-12	120362	08/03/2013	-2	78.913	10.702
ct92-M305Pete-12	120362	08/03/2013	-2	78.902	10.532
ct92-M305Pete-12	120362	08/03/2013	-2	78.903	10.525
ct92-M305Pete-12	120362	08/03/2013	-2	78.909	10.517
ct92-M305Pete-12	120362	08/03/2013	-2	78.908	10.482
ct92-M305Pete-12	120362	08/03/2013	-2	78.914	10.422
ct92-M305Pete-12	120362	08/03/2013	-2	78.919	10.392
ct92-M305Pete-12	120362	08/03/2013	-2	78.905	10.276
ct92-M305Pete-12	120362	08/03/2013	0	78.929	10.3
ct92-M305Pete-12	120362	08/03/2013	-2	78.929	10.276
ct92-M305Pete-12	120362	08/03/2013	-2	78.934	10.354
ct92-M305Pete-12	120362	08/03/2013	-2	78.922	10.416
ct92-M305Pete-12	120362	08/03/2013	-2	78.913	10.433
ct92-M305Pete-12	120362	08/03/2013	-1	78.908	10.443
ct92-M305Pete-12	120362	08/03/2013	-1	78.919	10.514
ct92-M305Pete-12	120362	08/03/2013	-2	78.912	10.473
ct92-M305Pete-12	120362	08/03/2013	-2	78.907	10.43
ct92-M305Pete-12	120362	08/03/2013	-2	78.92	10.466
ct92-M305Pete-12	120362	08/03/2013	-1	78.915	10.32
ct92-M305Pete-12	120362	08/03/2013	-1	78.921	10.251
ct92-M305Pete-12	120362	08/03/2013	-1	78.902	10.44
ct92-M305Pete-12	120362	08/03/2013	-2	78.907	10.455
ct92-M305Pete-12	120362	08/03/2013	-2	78.896	10.454
ct92-M305Pete-12	120362	08/03/2013	-2	78.907	10.452
ct92-M305Pete-12	120362	08/03/2013	0	78.894	10.532
ct92-M305Pete-12	120362	08/03/2013	-2	78.902	10.482
ct92-M305Pete-12	120362	08/03/2013	-2	78.902	10.475
ct92-M305Pete-12	120362	08/03/2013	-2	78.902	10.478
ct92-M305Pete-12	120362	08/03/2013	-2	78.894	10.466
ct92-M305Pete-12	120362	08/03/2013	-2	78.9	10.441
ct92-M305Pete-12	120362	08/03/2013	-2	78.9	10.444
ct92-M305Pete-12	120362	08/03/2013	-2	78.897	10.43
ct92-M305Pete-12	120362	08/03/2013	0	78.908	10.376
ct92-M305Pete-12	120362	08/03/2013	-1	78.924	10.482
ct92-M305Pete-12	120362	08/03/2013	-2	78.909	10.484
ct92-M305Pete-12	120362	08/03/2013	-2	78.905	10.609
ct92-M305Pete-12	120362	08/03/2013	-2	78.906	10.475
ct92-M305Pete-12	120362	08/03/2013	-1	78.897	10.533
ct92-M305Pete-12	120362	08/03/2013	-2	78.906	10.467
ct92-M305Pete-12	120362	08/03/2013	-2	78.906	10.474
ct92-M305Pete-12	120362	08/03/2013	-2	78.906	10.474
ct92-M305Pete-12	120362	08/03/2013	-2	78.908	10.491
ct92-M305Pete-12	120362	08/03/2013	-2	78.906	10.492
ct92-M305Pete-12	120362	08/03/2013	-2	78.907	10.487
ct92-M305Pete-12	120362	08/03/2013	-2	78.906	10.476
ct92-M305Pete-12	120362	08/03/2013	-2	78.905	10.46
ct92-M305Pete-12	120362	08/03/2013	-2	78.917	10.474
ct92-M305Pete-12	120362	08/03/2013	-2	78.913	10.509
ct92-M305Pete-12	120362	08/03/2013	-1	78.907	10.503
ct92-M305Pete-12	120362	08/03/2013	-2	78.9	10.573
ct92-M305Pete-12	120362	08/03/2013	-2	78.9	10.401

ct92-M305Pete-12	120362	08/03/2013	-1	78.906	10.63
ct92-M305Pete-12	120362	08/03/2013	-2	78.906	10.629
ct92-M305Pete-12	120362	08/03/2013	-2	78.906	10.646
ct92-M305Pete-12	120362	08/03/2013	-2	78.905	10.664
ct92-M305Pete-12	120362	08/03/2013	-2	78.905	10.667
ct92-M305Pete-12	120362	08/03/2013	-2	78.905	10.602
ct92-M305Pete-12	120362	09/03/2013	-1	78.916	10.61
ct92-M305Pete-12	120362	09/03/2013	-1	78.926	10.625
ct92-M305Pete-12	120362	09/03/2013	-2	78.902	10.626
ct92-M305Pete-12	120362	09/03/2013	-2	78.929	10.653
ct92-M305Pete-12	120362	09/03/2013	0	78.908	10.591
ct92-M305Pete-12	120362	09/03/2013	-1	78.914	10.581
ct92-M305Pete-12	120362	09/03/2013	-2	78.902	10.585
ct92-M305Pete-12	120362	09/03/2013	-1	78.9	10.568
ct92-M305Pete-12	120362	09/03/2013	-2	78.908	10.563
ct92-M305Pete-12	120362	09/03/2013	-2	78.932	10.617
ct92-M305Pete-12	120362	09/03/2013	-2	78.931	10.613
ct92-M305Pete-12	120362	09/03/2013	-2	78.913	10.528
ct92-M305Pete-12	120362	09/03/2013	-1	78.925	10.485
ct92-M305Pete-12	120362	09/03/2013	-2	78.915	10.524
ct92-M305Pete-12	120362	09/03/2013	-1	78.915	10.667
ct92-M305Pete-12	120362	09/03/2013	-1	78.902	10.633
ct92-M305Pete-12	120362	09/03/2013	-2	78.904	10.648
ct92-M305Pete-12	120362	09/03/2013	-1	78.903	10.669
ct92-M305Pete-12	120362	09/03/2013	-2	78.901	10.666
ct92-M305Pete-12	120362	09/03/2013	-1	78.896	10.596
ct92-M305Pete-12	120362	09/03/2013	-1	78.896	10.596
ct92-M305Pete-12	120362	09/03/2013	-1	78.911	10.58
ct92-M305Pete-12	120362	09/03/2013	-2	78.915	10.619
ct92-M305Pete-12	120362	09/03/2013	-2	78.921	10.578
ct92-M305Pete-12	120362	09/03/2013	-2	78.922	10.628
ct92-M305Pete-12	120362	09/03/2013	-2	78.908	10.605
ct92-M305Pete-12	120362	09/03/2013	-2	78.919	10.684
ct92-M305Pete-12	120362	09/03/2013	-2	78.919	10.658
ct92-M305Pete-12	120362	09/03/2013	-2	78.915	10.695
ct92-M305Pete-12	120362	09/03/2013	-1	78.917	10.725
ct92-M305Pete-12	120362	09/03/2013	-2	78.904	10.705
ct92-M305Pete-12	120362	09/03/2013	-2	78.919	10.615
ct92-M305Pete-12	120362	09/03/2013	-2	78.919	10.617
ct92-M305Pete-12	120362	09/03/2013	-2	78.919	10.715
ct92-M305Pete-12	120362	09/03/2013	-2	78.919	10.672
ct92-M305Pete-12	120362	09/03/2013	-2	78.918	10.673
ct92-M305Pete-12	120362	09/03/2013	-1	78.914	10.659
ct92-M305Pete-12	120362	09/03/2013	-1	78.916	10.661
ct92-M305Pete-12	120362	09/03/2013	-2	78.917	10.677
ct92-M305Pete-12	120362	09/03/2013	-2	78.913	10.602
ct92-M305Pete-12	120362	09/03/2013	-2	78.91	10.626
ct92-M305Pete-12	120362	09/03/2013	-2	78.912	10.654
ct92-M305Pete-12	120362	09/03/2013	-2	78.909	10.642
ct92-M305Pete-12	120362	09/03/2013	-2	78.909	10.65
ct92-M305Pete-12	120362	09/03/2013	-2	78.911	10.677
ct92-M305Pete-12	120362	09/03/2013	-2	78.914	10.685
ct92-M305Pete-12	120362	09/03/2013	-2	78.909	10.667
ct92-M305Pete-12	120362	09/03/2013	-2	78.914	10.69
ct92-M305Pete-12	120362	09/03/2013	-2	78.913	10.698
ct92-M305Pete-12	120362	09/03/2013	-1	78.912	10.711
ct92-M305Pete-12	120362	09/03/2013	-1	78.896	10.676
ct92-M305Pete-12	120362	09/03/2013	-1	78.898	10.669
ct92-M305Pete-12	120362	09/03/2013	-2	78.9	10.67
ct92-M305Pete-12	120362	09/03/2013	2	78.904	10.656
ct92-M305Pete-12	120362	09/03/2013	-2	78.914	10.68
ct92-M305Pete-12	120362	09/03/2013	-2	78.904	10.676
ct92-M305Pete-12	120362	09/03/2013	-2	78.908	10.638
ct92-M305Pete-12	120362	09/03/2013	-2	78.907	10.635
ct92-M305Pete-12	120362	09/03/2013	-2	78.907	10.634
ct92-M305Pete-12	120362	09/03/2013	-1	78.909	10.664
ct92-M305Pete-12	120362	09/03/2013	-1	78.92	10.625
ct92-M305Pete-12	120362	09/03/2013	-1	78.92	10.625

ct92-M305Pete-12	120362	09/03/2013	-2	78.925	10.58
ct92-M305Pete-12	120362	09/03/2013	-2	78.917	10.595
ct92-M305Pete-12	120362	09/03/2013	-2	78.928	10.583
ct92-M305Pete-12	120362	09/03/2013	-2	78.916	10.697
ct92-M305Pete-12	120362	09/03/2013	-2	78.923	10.663
ct92-M305Pete-12	120362	09/03/2013	-2	78.92	10.655
ct92-M305Pete-12	120362	09/03/2013	-2	78.894	10.625
ct92-M305Pete-12	120362	09/03/2013	-2	78.902	10.787
ct92-M305Pete-12	120362	09/03/2013	-2	78.878	10.769
ct92-M305Pete-12	120362	09/03/2013	-2	78.843	10.77
ct92-M305Pete-12	120362	09/03/2013	-2	78.888	10.482
ct92-M305Pete-12	120362	09/03/2013	-2	78.881	10.469
ct92-M305Pete-12	120362	09/03/2013	-2	78.88	10.514
ct92-M305Pete-12	120362	09/03/2013	-2	78.874	10.474
ct92-M305Pete-12	120362	09/03/2013	-2	78.88	10.456
ct92-M305Pete-12	120362	09/03/2013	-1	78.922	10.661
ct92-M305Pete-12	120362	10/03/2013	-2	78.915	10.663
ct92-M305Pete-12	120362	10/03/2013	-2	78.913	10.641
ct92-M305Pete-12	120362	10/03/2013	-2	78.911	10.612
ct92-M305Pete-12	120362	10/03/2013	-2	78.909	10.593
ct92-M305Pete-12	120362	10/03/2013	-2	78.911	10.625
ct92-M305Pete-12	120362	10/03/2013	-2	78.911	10.617
ct92-M305Pete-12	120362	10/03/2013	-2	78.907	10.587
ct92-M305Pete-12	120362	10/03/2013	-2	78.906	10.578
ct92-M305Pete-12	120362	10/03/2013	-2	78.907	10.533
ct92-M305Pete-12	120362	10/03/2013	-2	78.908	10.579
ct92-M305Pete-12	120362	10/03/2013	-2	78.918	10.604
ct92-M305Pete-12	120362	10/03/2013	-2	78.917	10.636
ct92-M305Pete-12	120362	10/03/2013	-1	78.907	10.727
ct92-M305Pete-12	120362	10/03/2013	-2	78.906	10.71
ct92-M305Pete-12	120362	10/03/2013	-2	78.904	10.706
ct92-M305Pete-12	120362	10/03/2013	-2	78.903	10.705
ct92-M305Pete-12	120362	10/03/2013	-2	78.9	10.68
ct92-M305Pete-12	120362	10/03/2013	-2	78.902	10.712
ct92-M305Pete-12	120362	10/03/2013	-2	78.914	10.689
ct92-M305Pete-12	120362	10/03/2013	-2	78.898	10.653
ct92-M305Pete-12	120362	10/03/2013	-2	78.901	10.691
ct92-M305Pete-12	120362	10/03/2013	-2	78.908	10.707
ct92-M305Pete-12	120362	10/03/2013	-2	78.91	10.7
ct92-M305Pete-12	120362	10/03/2013	-2	78.905	10.76
ct92-M305Pete-12	120362	10/03/2013	-2	78.901	10.609
ct92-M305Pete-12	120362	10/03/2013	-2	78.902	10.641
ct92-M305Pete-12	120362	10/03/2013	-2	78.928	10.614
ct92-M305Pete-12	120362	10/03/2013	-2	78.906	10.621
ct92-M305Pete-12	120362	10/03/2013	-1	78.912	10.519
ct92-M305Pete-12	120362	10/03/2013	-2	78.91	10.626
ct92-M305Pete-12	120362	10/03/2013	-2	78.909	10.656
ct92-M305Pete-12	120362	10/03/2013	-2	78.919	10.649
ct92-M305Pete-12	120362	10/03/2013	-2	78.908	10.655
ct92-M305Pete-12	120362	10/03/2013	-1	78.912	10.78
ct92-M305Pete-12	120362	10/03/2013	-1	78.913	10.777
ct92-M305Pete-12	120362	10/03/2013	-2	78.916	10.724
ct92-M305Pete-12	120362	10/03/2013	-2	78.914	10.72
ct92-M305Pete-12	120362	10/03/2013	-2	78.905	10.719
ct92-M305Pete-12	120362	10/03/2013	-2	78.915	10.723
ct92-M305Pete-12	120362	10/03/2013	-2	78.922	10.746
ct92-M305Pete-12	120362	10/03/2013	-2	78.926	10.71
ct92-M305Pete-12	120362	10/03/2013	-2	78.925	10.695
ct92-M305Pete-12	120362	10/03/2013	-1	78.941	10.751
ct92-M305Pete-12	120362	10/03/2013	-1	78.94	10.75
ct92-M305Pete-12	120362	10/03/2013	-1	78.927	10.691
ct92-M305Pete-12	120362	10/03/2013	-2	78.926	10.679
ct92-M305Pete-12	120362	10/03/2013	-1	78.919	10.655
ct92-M305Pete-12	120362	10/03/2013	-2	78.912	10.661
ct92-M305Pete-12	120362	10/03/2013	-1	78.913	10.613
ct92-M305Pete-12	120362	10/03/2013	-1	78.91	10.592
ct92-M305Pete-12	120362	10/03/2013	-2	78.911	10.596
ct92-M305Pete-12	120362	10/03/2013	-2	78.911	10.593

ct92-M305Pete-12	120362	10/03/2013	-1	78.927	10.54
ct92-M305Pete-12	120362	10/03/2013	-2	78.928	10.511
ct92-M305Pete-12	120362	10/03/2013	-2	78.929	10.512
ct92-M305Pete-12	120362	10/03/2013	-1	78.934	10.591
ct92-M305Pete-12	120362	10/03/2013	-1	78.924	10.531
ct92-M305Pete-12	120362	10/03/2013	-2	78.935	10.588
ct92-M305Pete-12	120362	10/03/2013	-2	78.935	10.583
ct92-M305Pete-12	120362	10/03/2013	-2	78.934	10.538
ct92-M305Pete-12	120362	10/03/2013	-2	78.929	10.565
ct92-M305Pete-12	120362	10/03/2013	-2	78.927	10.503
ct92-M305Pete-12	120362	10/03/2013	-2	78.928	10.503
ct92-M305Pete-12	120362	10/03/2013	-1	78.929	10.499
ct92-M305Pete-12	120362	10/03/2013	-2	78.93	10.496
ct92-M305Pete-12	120362	10/03/2013	-1	78.942	10.483
ct92-M305Pete-12	120362	10/03/2013	-1	78.941	10.484
ct92-M305Pete-12	120362	10/03/2013	-1	78.94	10.459
ct92-M305Pete-12	120362	10/03/2013	-2	78.933	10.491
ct92-M305Pete-12	120362	10/03/2013	-2	78.944	10.44
ct92-M305Pete-12	120362	10/03/2013	-2	78.94	10.422
ct92-M305Pete-12	120362	10/03/2013	-2	78.94	10.418
ct92-M305Pete-12	120362	10/03/2013	-2	78.943	10.423
ct92-M305Pete-12	120362	10/03/2013	-2	78.943	10.424
ct92-M305Pete-12	120362	10/03/2013	-2	78.939	10.356
ct92-M305Pete-12	120362	10/03/2013	-2	78.938	10.359
ct92-M305Pete-12	120362	11/03/2013	-2	78.938	10.362
ct92-M305Pete-12	120362	11/03/2013	-2	78.94	10.378
ct92-M305Pete-12	120362	11/03/2013	-2	78.942	10.37
ct92-M305Pete-12	120362	11/03/2013	-1	78.919	10.391
ct92-M305Pete-12	120362	11/03/2013	-2	78.944	10.37
ct92-M305Pete-12	120362	11/03/2013	-2	78.943	10.371
ct92-M305Pete-12	120362	11/03/2013	-2	78.935	10.408
ct92-M305Pete-12	120362	11/03/2013	-2	78.936	10.4
ct92-M305Pete-12	120362	11/03/2013	-2	78.934	10.409
ct92-M305Pete-12	120362	11/03/2013	-2	78.938	10.444
ct92-M305Pete-12	120362	11/03/2013	-2	78.939	10.442
ct92-M305Pete-12	120362	11/03/2013	-2	78.944	10.387
ct92-M305Pete-12	120362	11/03/2013	-1	78.935	10.367
ct92-M305Pete-12	120362	11/03/2013	-1	78.935	10.336
ct92-M305Pete-12	120362	11/03/2013	-2	78.944	10.406
ct92-M305Pete-12	120362	11/03/2013	-2	78.944	10.421
ct92-M305Pete-12	120362	11/03/2013	-1	78.944	10.427
ct92-M305Pete-12	120362	11/03/2013	-1	78.943	10.521
ct92-M305Pete-12	120362	11/03/2013	-1	78.922	10.564
ct92-M305Pete-12	120362	11/03/2013	-2	78.921	10.565
ct92-M305Pete-12	120362	11/03/2013	-2	78.935	10.575
ct92-M305Pete-12	120362	11/03/2013	-2	78.942	10.554
ct92-M305Pete-12	120362	11/03/2013	-1	78.941	10.568
ct92-M305Pete-12	120362	11/03/2013	-1	78.945	10.575
ct92-M305Pete-12	120362	11/03/2013	-2	78.935	10.588
ct92-M305Pete-12	120362	11/03/2013	-2	78.94	10.589
ct92-M305Pete-12	120362	11/03/2013	-2	78.931	10.624
ct92-M305Pete-12	120362	11/03/2013	-2	78.924	10.67
ct92-M305Pete-12	120362	11/03/2013	0	78.945	10.455
ct92-M305Pete-12	120362	11/03/2013	-1	78.956	10.658
ct92-M305Pete-12	120362	11/03/2013	-1	78.942	10.633
ct92-M305Pete-12	120362	11/03/2013	-2	78.931	10.621
ct92-M305Pete-12	120362	11/03/2013	-2	78.935	10.674
ct92-M305Pete-12	120362	11/03/2013	-2	78.918	10.574
ct92-M305Pete-12	120362	11/03/2013	-2	78.929	10.668
ct92-M305Pete-12	120362	11/03/2013	-2	78.925	10.72
ct92-M305Pete-12	120362	11/03/2013	-2	78.924	10.739
ct92-M305Pete-12	120362	11/03/2013	-2	78.924	10.594
ct92-M305Pete-12	120362	11/03/2013	-2	78.936	10.655
ct92-M305Pete-12	120362	11/03/2013	-2	78.932	10.606
ct92-M305Pete-12	120362	11/03/2013	-2	78.929	10.665
ct92-M305Pete-12	120362	11/03/2013	-2	78.933	10.681
ct92-M305Pete-12	120362	11/03/2013	-2	78.913	10.632
ct92-M305Pete-12	120362	11/03/2013	-2	78.903	10.825

ct92-M305Pete-12	120362	11/03/2013	-1	78.902	10.816
ct92-M305Pete-12	120362	11/03/2013	-2	78.9	10.828
ct92-M305Pete-12	120362	11/03/2013	-2	78.9	10.793
ct92-M305Pete-12	120362	11/03/2013	-2	78.905	10.789
ct92-M305Pete-12	120362	11/03/2013	-1	78.906	10.797
ct92-M305Pete-12	120362	11/03/2013	-2	78.908	10.792
ct92-M305Pete-12	120362	11/03/2013	-2	78.898	10.854
ct92-M305Pete-12	120362	11/03/2013	-2	78.91	10.793
ct92-M305Pete-12	120362	11/03/2013	-2	78.891	10.776
ct92-M305Pete-12	120362	11/03/2013	-2	78.908	10.76
ct92-M305Pete-12	120362	11/03/2013	-2	78.905	10.762
ct92-M305Pete-12	120362	11/03/2013	-2	78.911	10.748
ct92-M305Pete-12	120362	11/03/2013	-2	78.911	10.745
ct92-M305Pete-12	120362	11/03/2013	-2	78.912	10.757
ct92-M305Pete-12	120362	11/03/2013	-2	78.915	10.694
ct92-M305Pete-12	120362	11/03/2013	-2	78.916	10.559
ct92-M305Pete-12	120362	11/03/2013	-2	78.912	10.753
ct92-M305Pete-12	120362	11/03/2013	-2	78.91	10.722
ct92-M305Pete-12	120362	11/03/2013	-2	78.92	10.557
ct92-M305Pete-12	120362	11/03/2013	-2	78.911	10.729
ct92-M305Pete-12	120362	11/03/2013	-2	78.911	10.74
ct92-M305Pete-12	120362	11/03/2013	-2	78.91	10.719
ct92-M305Pete-12	120362	11/03/2013	-2	78.912	10.719
ct92-M305Pete-12	120362	11/03/2013	-2	78.929	10.621
ct92-M305Pete-12	120362	11/03/2013	-2	78.927	10.642
ct92-M305Pete-12	120362	11/03/2013	-1	78.923	10.547
ct92-M305Pete-12	120362	11/03/2013	-2	78.925	10.539
ct92-M305Pete-12	120362	11/03/2013	-2	78.927	10.605
ct92-M305Pete-12	120362	11/03/2013	-2	78.917	10.613
ct92-M305Pete-12	120362	11/03/2013	-2	78.917	10.539
ct92-M305Pete-12	120362	11/03/2013	-2	78.924	10.543
ct92-M305Pete-12	120362	11/03/2013	-2	78.915	10.525
ct92-M305Pete-12	120362	11/03/2013	-2	78.923	10.608
ct92-M305Pete-12	120362	11/03/2013	-2	78.927	10.59
ct92-M305Pete-12	120362	11/03/2013	-2	78.926	10.566
ct92-M305Pete-12	120362	11/03/2013	-2	78.915	10.47
ct92-M305Pete-12	120362	11/03/2013	-1	78.913	10.501
ct92-M305Pete-12	120362	11/03/2013	-1	78.92	10.625
ct92-M305Pete-12	120362	11/03/2013	-2	78.912	10.498
ct92-M305Pete-12	120362	11/03/2013	-2	78.912	10.499
ct92-M305Pete-12	120362	12/03/2013	-2	78.913	10.494
ct92-M305Pete-12	120362	12/03/2013	-2	78.889	10.603
ct92-M305Pete-12	120362	12/03/2013	-2	78.913	10.503
ct92-M305Pete-12	120362	12/03/2013	-2	78.885	10.493
ct92-M305Pete-12	120362	12/03/2013	-1	78.911	10.462
ct92-M305Pete-12	120362	12/03/2013	-1	78.888	10.47
ct92-M305Pete-12	120362	12/03/2013	-2	78.889	10.487
ct92-M305Pete-12	120362	12/03/2013	-1	78.891	10.568
ct92-M305Pete-12	120362	12/03/2013	-2	78.892	10.563
ct92-M305Pete-12	120362	12/03/2013	-2	78.886	10.559
ct92-M305Pete-12	120362	12/03/2013	-2	78.884	10.537
ct92-M305Pete-12	120362	12/03/2013	-1	78.907	10.506
ct92-M305Pete-12	120362	12/03/2013	-1	78.908	10.511
ct92-M305Pete-12	120362	12/03/2013	-2	78.911	10.55
ct92-M305Pete-12	120362	12/03/2013	-2	78.912	10.55
ct92-M305Pete-12	120362	12/03/2013	-2	78.903	10.568
ct92-M305Pete-12	120362	12/03/2013	-2	78.901	10.576
ct92-M305Pete-12	120362	12/03/2013	-2	78.914	10.581
ct92-M305Pete-12	120362	12/03/2013	-2	78.913	10.572
ct92-M305Pete-12	120362	12/03/2013	-2	78.935	10.569
ct92-M305Pete-12	120362	12/03/2013	-2	78.919	10.59
ct92-M305Pete-12	120362	12/03/2013	-2	78.924	10.553
ct92-M305Pete-12	120362	12/03/2013	-2	78.925	10.565
ct92-M305Pete-12	120362	12/03/2013	-2	78.905	10.579
ct92-M305Pete-12	120362	12/03/2013	-2	78.928	10.582
ct92-M305Pete-12	120362	12/03/2013	-2	78.927	10.577
ct92-M305Pete-12	120362	12/03/2013	-2	78.907	10.469
ct92-M305Pete-12	120362	12/03/2013	-2	78.896	10.537

ct92-M305Pete-12	120362	12/03/2013	-2	78.92	10.688
ct92-M305Pete-12	120362	12/03/2013	-2	78.914	10.69
ct92-M305Pete-12	120362	12/03/2013	-2	78.915	10.65
ct92-M305Pete-12	120362	12/03/2013	-2	78.925	10.552
ct92-M305Pete-12	120362	12/03/2013	-2	78.919	10.55
ct92-M305Pete-12	120362	12/03/2013	-2	78.917	10.57
ct92-M305Pete-12	120362	12/03/2013	-1	78.929	10.559
ct92-M305Pete-12	120362	12/03/2013	-2	78.92	10.576
ct92-M305Pete-12	120362	12/03/2013	-2	78.912	10.638
ct92-M305Pete-12	120362	12/03/2013	-2	78.918	10.617
ct92-M305Pete-12	120362	12/03/2013	-2	78.909	10.597
ct92-M305Pete-12	120362	12/03/2013	1	78.931	10.651
ct92-M305Pete-12	120362	12/03/2013	-2	78.94	10.672
ct92-M305Pete-12	120362	12/03/2013	-2	78.935	10.572
ct92-M305Pete-12	120362	12/03/2013	-2	78.92	10.624
ct92-M305Pete-12	120362	12/03/2013	-1	78.917	10.65
ct92-M305Pete-12	120362	12/03/2013	-1	78.911	10.639
ct92-M305Pete-12	120362	12/03/2013	-2	78.918	10.653
ct92-M305Pete-12	120362	12/03/2013	-1	78.918	10.591
ct92-M305Pete-12	120362	12/03/2013	-2	78.921	10.616
ct92-M305Pete-12	120362	12/03/2013	-2	78.918	10.631
ct92-M305Pete-12	120362	12/03/2013	-1	78.922	10.603
ct92-M305Pete-12	120362	12/03/2013	-2	78.921	10.608
ct92-M305Pete-12	120362	12/03/2013	-1	78.924	10.621
ct92-M305Pete-12	120362	12/03/2013	-1	78.924	10.621
ct92-M305Pete-12	120362	12/03/2013	-2	78.917	10.647
ct92-M305Pete-12	120362	12/03/2013	-2	78.915	10.627
ct92-M305Pete-12	120362	12/03/2013	-2	78.941	10.641
ct92-M305Pete-12	120362	12/03/2013	-1	78.923	10.599
ct92-M305Pete-12	120362	12/03/2013	-2	78.911	10.556
ct92-M305Pete-12	120362	12/03/2013	-2	78.921	10.593
ct92-M305Pete-12	120362	12/03/2013	-1	78.919	10.531
ct92-M305Pete-12	120362	12/03/2013	-2	78.915	10.556
ct92-M305Pete-12	120362	12/03/2013	-2	78.923	10.73
ct92-M305Pete-12	120362	12/03/2013	-2	78.915	10.512
ct92-M305Pete-12	120362	12/03/2013	-2	78.918	10.504
ct92-M305Pete-12	120362	13/03/2013	-2	78.934	10.618
ct92-M305Pete-12	120362	13/03/2013	-2	78.912	10.561
ct92-M305Pete-12	120362	13/03/2013	-1	78.914	10.609
ct92-M305Pete-12	120362	13/03/2013	-2	78.926	10.572
ct92-M305Pete-12	120362	13/03/2013	-2	78.939	10.609
ct92-M305Pete-12	120362	13/03/2013	-2	78.942	10.619
ct92-M305Pete-12	120362	13/03/2013	-2	78.961	10.669
ct92-M305Pete-12	120362	13/03/2013	-2	78.952	10.631
ct92-M305Pete-12	120362	13/03/2013	-1	78.901	10.797
ct92-M305Pete-12	120362	13/03/2013	-2	78.895	10.835
ct92-M305Pete-12	120362	13/03/2013	-1	78.907	10.622
ct92-M305Pete-12	120362	13/03/2013	-2	78.907	10.622
ct92-M305Pete-12	120362	13/03/2013	-2	78.911	10.622
ct92-M305Pete-12	120362	13/03/2013	-2	78.909	10.623
ct92-M305Pete-12	120362	13/03/2013	-2	78.907	10.625
ct92-M305Pete-12	120362	13/03/2013	-1	78.902	10.655
ct92-M305Pete-12	120362	13/03/2013	-2	78.902	10.662
ct92-M305Pete-12	120362	13/03/2013	-2	78.905	10.575
ct92-M305Pete-12	120362	13/03/2013	-2	78.902	10.66
ct92-M305Pete-12	120362	13/03/2013	-2	78.902	10.66
ct92-M305Pete-12	120362	13/03/2013	-1	78.91	10.565
ct92-M305Pete-12	120362	13/03/2013	-2	78.897	10.693
ct92-M305Pete-12	120362	13/03/2013	-2	78.903	10.64
ct92-M305Pete-12	120362	13/03/2013	-2	78.903	10.622
ct92-M305Pete-12	120362	13/03/2013	-1	78.885	10.636
ct92-M305Pete-12	120362	13/03/2013	-1	78.881	10.644
ct92-M305Pete-12	120362	13/03/2013	0	78.93	10.576
ct92-M305Pete-12	120362	13/03/2013	-1	78.918	10.677
ct92-M305Pete-12	120362	13/03/2013	-2	78.927	10.577
ct92-M305Pete-12	120362	13/03/2013	-2	78.936	10.569
ct92-M305Pete-12	120362	13/03/2013	1	78.909	10.641
ct92-M305Pete-12	120362	13/03/2013	-2	78.919	10.606

ct92-M305Pete-12	120362	13/03/2013	-2	78.909	10.598
ct92-M305Pete-12	120362	13/03/2013	-2	78.916	10.611
ct92-M305Pete-12	120362	13/03/2013	-2	78.922	10.647
ct92-M305Pete-12	120362	13/03/2013	-2	78.931	10.589
ct92-M305Pete-12	120362	13/03/2013	-2	78.928	10.614
ct92-M305Pete-12	120362	13/03/2013	-2	78.9	10.62
ct92-M305Pete-12	120362	13/03/2013	-1	78.912	10.616
ct92-M305Pete-12	120362	13/03/2013	-1	78.917	10.487
ct92-M305Pete-12	120362	13/03/2013	-1	78.926	10.536
ct92-M305Pete-12	120362	13/03/2013	-2	78.92	10.436
ct92-M305Pete-12	120362	13/03/2013	-2	78.915	10.404
ct92-M305Pete-12	120362	13/03/2013	-2	78.905	10.513
ct92-M305Pete-12	120362	13/03/2013	-2	78.905	10.515
ct92-M305Pete-12	120362	13/03/2013	-1	78.914	10.544
ct92-M305Pete-12	120362	13/03/2013	-1	78.913	10.6
ct92-M305Pete-12	120362	13/03/2013	-1	78.91	10.599
ct92-M305Pete-12	120362	13/03/2013	-2	78.912	10.514
ct92-M305Pete-12	120362	13/03/2013	-2	78.916	10.631
ct92-M305Pete-12	120362	13/03/2013	-1	78.913	10.434
ct92-M305Pete-12	120362	13/03/2013	-2	78.914	10.472
ct92-M305Pete-12	120362	13/03/2013	-2	78.918	10.498
ct92-M305Pete-12	120362	13/03/2013	-1	78.914	10.505
ct92-M305Pete-12	120362	13/03/2013	-2	78.909	10.528
ct92-M305Pete-12	120362	13/03/2013	-2	78.933	10.623
ct92-M305Pete-12	120362	13/03/2013	-2	78.933	10.616
ct92-M305Pete-12	120362	13/03/2013	-2	78.933	10.622
ct92-M305Pete-12	120362	13/03/2013	-2	78.933	10.568
ct92-M305Pete-12	120362	14/03/2013	-2	78.916	10.464
ct92-M305Pete-12	120362	14/03/2013	-2	78.903	10.553
ct92-M305Pete-12	120362	14/03/2013	-2	78.894	10.547
ct92-M305Pete-12	120362	14/03/2013	-2	78.898	10.557
ct92-M305Pete-12	120362	14/03/2013	-2	78.899	10.547
ct92-M305Pete-12	120362	14/03/2013	-2	78.902	10.621
ct92-M305Pete-12	120362	14/03/2013	-2	78.9	10.62
ct92-M305Pete-12	120362	14/03/2013	-2	78.9	10.632
ct92-M305Pete-12	120362	14/03/2013	-2	78.897	10.643
ct92-M305Pete-12	120362	14/03/2013	-1	78.915	10.676
ct92-M305Pete-12	120362	14/03/2013	-2	78.897	10.645
ct92-M305Pete-12	120362	14/03/2013	-1	78.919	10.664
ct92-M305Pete-12	120362	14/03/2013	-2	78.92	10.667
ct92-M305Pete-12	120362	14/03/2013	-1	78.899	10.694
ct92-M305Pete-12	120362	14/03/2013	-2	78.901	10.702
ct92-M305Pete-12	120362	14/03/2013	-2	78.918	10.678
ct92-M305Pete-12	120362	14/03/2013	-2	78.92	10.682
ct92-M305Pete-12	120362	14/03/2013	-2	78.908	10.783
ct92-M305Pete-12	120362	14/03/2013	-2	78.917	10.644
ct92-M305Pete-12	120362	14/03/2013	-2	78.917	10.643
ct92-M305Pete-12	120362	14/03/2013	-1	78.924	10.674
ct92-M305Pete-12	120362	14/03/2013	-2	78.917	10.643
ct92-M305Pete-12	120362	14/03/2013	-2	78.919	10.638
ct92-M305Pete-12	120362	14/03/2013	-2	78.922	10.638
ct92-M305Pete-12	120362	14/03/2013	-1	78.926	10.607
ct92-M305Pete-12	120362	14/03/2013	-2	78.93	10.629
ct92-M305Pete-12	120362	14/03/2013	-1	78.926	10.624
ct92-M305Pete-12	120362	14/03/2013	-1	78.921	10.665
ct92-M305Pete-12	120362	14/03/2013	-1	78.924	10.606
ct92-M305Pete-12	120362	14/03/2013	-1	78.922	10.609
ct92-M305Pete-12	120362	14/03/2013	-2	78.92	10.613
ct92-M305Pete-12	120362	14/03/2013	-2	78.927	10.638
ct92-M305Pete-12	120362	14/03/2013	-2	78.925	10.631
ct92-M305Pete-12	120362	14/03/2013	-2	78.924	10.6
ct92-M305Pete-12	120362	14/03/2013	-2	78.922	10.558
ct92-M305Pete-12	120362	14/03/2013	-1	78.908	10.571
ct92-M305Pete-12	120362	14/03/2013	-2	78.913	10.647
ct92-M305Pete-12	120362	14/03/2013	-2	78.908	10.556
ct92-M305Pete-12	120362	14/03/2013	-2	78.925	10.572
ct92-M305Pete-12	120362	14/03/2013	-1	78.924	10.554
ct92-M305Pete-12	120362	14/03/2013	-2	78.926	10.563

ct92-M305Pete-12	120362	14/03/2013	-2	78.919	10.554
ct92-M305Pete-12	120362	14/03/2013	-1	78.906	10.58
ct92-M305Pete-12	120362	14/03/2013	-1	78.899	10.477
ct92-M305Pete-12	120362	14/03/2013	-2	78.898	10.524
ct92-M305Pete-12	120362	14/03/2013	-1	78.923	10.509
ct92-M305Pete-12	120362	14/03/2013	-2	78.937	10.634
ct92-M305Pete-12	120362	14/03/2013	-2	78.939	10.615
ct92-M305Pete-12	120362	14/03/2013	-2	78.935	10.571
ct92-M305Pete-12	120362	14/03/2013	-1	78.908	10.462
ct92-M305Pete-12	120362	14/03/2013	-2	78.912	10.326
ct92-M305Pete-12	120362	14/03/2013	-2	78.903	10.374
ct92-M305Pete-12	120362	14/03/2013	-1	78.915	10.406
ct92-M305Pete-12	120362	14/03/2013	-1	78.907	10.4
ct92-M305Pete-12	120362	14/03/2013	-1	78.907	10.4
ct92-M305Pete-12	120362	14/03/2013	-2	78.876	10.356
ct92-M305Pete-12	120362	14/03/2013	-2	78.907	10.359
ct92-M305Pete-12	120362	14/03/2013	-2	78.893	10.343
ct92-M305Pete-12	120362	14/03/2013	-1	78.88	10.325
ct92-M305Pete-12	120362	15/03/2013	-2	78.879	10.306
ct92-M305Pete-12	120362	15/03/2013	-2	78.926	10.552
ct92-M305Pete-12	120362	15/03/2013	-2	78.884	10.289
ct92-M305Pete-12	120362	15/03/2013	-2	78.889	10.237
ct92-M305Pete-12	120362	15/03/2013	-2	78.926	10.371
ct92-M305Pete-12	120362	15/03/2013	-1	78.878	10.319
ct92-M305Pete-12	120362	15/03/2013	-2	78.881	10.318
ct92-M305Pete-12	120362	15/03/2013	-2	78.881	10.338
ct92-M305Pete-12	120362	15/03/2013	-1	78.922	10.586
ct92-M305Pete-12	120362	15/03/2013	-1	78.924	10.557
ct92-M305Pete-12	120362	15/03/2013	-2	78.921	10.549
ct92-M305Pete-12	120362	15/03/2013	-2	78.94	10.509
ct92-M305Pete-12	120362	15/03/2013	-2	78.94	10.509
ct92-M305Pete-12	120362	15/03/2013	-1	78.947	10.59
ct92-M305Pete-12	120362	15/03/2013	-1	78.916	10.749
ct92-M305Pete-12	120362	15/03/2013	-2	78.939	10.623
ct92-M305Pete-12	120362	15/03/2013	-2	78.935	10.709
ct92-M305Pete-12	120362	15/03/2013	-2	78.932	10.7
ct92-M305Pete-12	120362	15/03/2013	-2	78.931	10.677
ct92-M305Pete-12	120362	15/03/2013	-2	78.935	10.716
ct92-M305Pete-12	120362	15/03/2013	-2	78.945	10.69
ct92-M305Pete-12	120362	15/03/2013	-2	78.934	10.719
ct92-M305Pete-12	120362	15/03/2013	-1	78.928	10.67
ct92-M305Pete-12	120362	15/03/2013	-1	78.922	10.632
ct92-M305Pete-12	120362	15/03/2013	-1	78.923	10.632
ct92-M305Pete-12	120362	15/03/2013	-2	78.937	10.609
ct92-M305Pete-12	120362	15/03/2013	-2	78.939	10.603
ct92-M305Pete-12	120362	15/03/2013	-2	78.928	10.63
ct92-M305Pete-12	120362	15/03/2013	-2	78.93	10.655
ct92-M305Pete-12	120362	15/03/2013	-2	78.921	10.699
ct92-M305Pete-12	120362	15/03/2013	-2	78.92	10.676
ct92-M305Pete-12	120362	15/03/2013	-1	78.93	10.693
ct92-M305Pete-12	120362	15/03/2013	-2	78.91	10.613
ct92-M305Pete-12	120362	15/03/2013	-2	78.937	10.692
ct92-M305Pete-12	120362	15/03/2013	-2	78.917	10.673
ct92-M305Pete-12	120362	15/03/2013	-2	78.941	10.697
ct92-M305Pete-12	120362	15/03/2013	-2	78.959	10.716
ct92-M305Pete-12	120362	15/03/2013	-2	78.945	10.794
ct92-M305Pete-12	120362	15/03/2013	-1	78.912	10.843
ct92-M305Pete-12	120362	15/03/2013	-2	78.943	10.779
ct92-M305Pete-12	120362	15/03/2013	-2	78.932	10.711
ct92-M305Pete-12	120362	15/03/2013	-1	78.918	10.621
ct92-M305Pete-12	120362	15/03/2013	-2	78.919	10.621
ct92-M305Pete-12	120362	15/03/2013	-1	78.934	10.617
ct92-M305Pete-12	120362	15/03/2013	-2	78.933	10.643
ct92-M305Pete-12	120362	15/03/2013	-2	78.933	10.644
ct92-M305Pete-12	120362	15/03/2013	-1	78.932	10.595
ct92-M305Pete-12	120362	15/03/2013	-2	78.876	10.533
ct92-M305Pete-12	120362	15/03/2013	-2	78.931	10.595
ct92-M305Pete-12	120362	15/03/2013	-1	78.924	10.638

ct92-M305Pete-12	120362	15/03/2013	-2	78.926	10.637
ct92-M305Pete-12	120362	15/03/2013	-1	78.922	10.644
ct92-M305Pete-12	120362	15/03/2013	-2	78.927	10.637
ct92-M305Pete-12	120362	15/03/2013	-2	78.926	10.637
ct92-M305Pete-12	120362	15/03/2013	-1	78.93	10.575
ct92-M305Pete-12	120362	15/03/2013	-2	78.94	10.479
ct92-M305Pete-12	120362	15/03/2013	-2	78.93	10.574
ct92-M305Pete-12	120362	15/03/2013	1	78.937	10.511
ct92-M305Pete-12	120362	15/03/2013	1	78.929	10.587
ct92-M305Pete-12	120362	15/03/2013	1	78.93	10.59
ct92-M305Pete-12	120362	15/03/2013	-2	78.93	10.612
ct92-M305Pete-12	120362	15/03/2013	-2	78.934	10.614
ct92-M305Pete-12	120362	15/03/2013	-2	78.941	10.377
ct92-M305Pete-12	120362	15/03/2013	-2	78.948	10.546
ct92-M305Pete-12	120362	15/03/2013	-2	78.944	10.535
ct92-M305Pete-12	120362	15/03/2013	-2	78.946	10.549
ct92-M305Pete-12	120362	15/03/2013	-2	78.944	10.597
ct92-M305Pete-12	120362	15/03/2013	-2	78.942	10.6
ct92-M305Pete-12	120362	15/03/2013	-2	78.944	10.601
ct92-M305Pete-12	120362	15/03/2013	-2	78.944	10.603
ct92-M305Pete-12	120362	15/03/2013	-2	78.94	10.605
ct92-M305Pete-12	120362	15/03/2013	-2	78.944	10.601
ct92-M305Pete-12	120362	15/03/2013	-2	78.946	10.615
ct92-M305Pete-12	120362	15/03/2013	-2	78.944	10.63
ct92-M305Pete-12	120362	15/03/2013	-2	78.932	10.639
ct92-M305Pete-12	120362	15/03/2013	-2	78.931	10.553
ct92-M305Pete-12	120362	15/03/2013	-2	78.917	10.55
ct92-M305Pete-12	120362	15/03/2013	-2	78.917	10.548
ct92-M305Pete-12	120362	16/03/2013	-2	78.911	10.687
ct92-M305Pete-12	120362	16/03/2013	-2	78.926	10.709
ct92-M305Pete-12	120362	16/03/2013	-2	78.925	10.715
ct92-M305Pete-12	120362	16/03/2013	-2	78.924	10.693
ct92-M305Pete-12	120362	16/03/2013	-1	78.914	10.699
ct92-M305Pete-12	120362	16/03/2013	-1	78.915	10.686
ct92-M305Pete-12	120362	16/03/2013	-2	78.913	10.704
ct92-M305Pete-12	120362	16/03/2013	-2	78.915	10.704
ct92-M305Pete-12	120362	16/03/2013	-2	78.915	10.721
ct92-M305Pete-12	120362	16/03/2013	-2	78.917	10.716
ct92-M305Pete-12	120362	16/03/2013	-2	78.913	10.718
ct92-M305Pete-12	120362	16/03/2013	-2	78.919	10.723
ct92-M305Pete-12	120362	16/03/2013	-2	78.916	10.68
ct92-M305Pete-12	120362	16/03/2013	-1	78.925	10.688
ct92-M305Pete-12	120362	16/03/2013	-2	78.925	10.686
ct92-M305Pete-12	120362	16/03/2013	-2	78.927	10.795
ct92-M305Pete-12	120362	16/03/2013	-2	78.934	10.669
ct92-M305Pete-12	120362	16/03/2013	-1	78.924	10.635
ct92-M305Pete-12	120362	16/03/2013	-2	78.927	10.658
ct92-M305Pete-12	120362	16/03/2013	-2	78.927	10.652
ct92-M305Pete-12	120362	16/03/2013	-2	78.902	10.58
ct92-M305Pete-12	120362	16/03/2013	-2	78.9	10.598
ct92-M305Pete-12	120362	16/03/2013	-1	78.915	10.448
ct92-M305Pete-12	120362	16/03/2013	-1	78.913	10.446
ct92-M305Pete-12	120362	16/03/2013	-2	78.916	10.599
ct92-M305Pete-12	120362	16/03/2013	-2	78.927	10.61
ct92-M305Pete-12	120362	16/03/2013	-2	78.927	10.6
ct92-M305Pete-12	120362	16/03/2013	-2	78.929	10.608
ct92-M305Pete-12	120362	16/03/2013	-2	78.923	10.473
ct92-M305Pete-12	120362	16/03/2013	-2	78.919	10.463
ct92-M305Pete-12	120362	16/03/2013	-2	78.918	10.466
ct92-M305Pete-12	120362	16/03/2013	-1	78.91	10.509
ct92-M305Pete-12	120362	16/03/2013	-1	78.915	10.412
ct92-M305Pete-12	120362	16/03/2013	-2	78.914	10.383
ct92-M305Pete-12	120362	16/03/2013	-2	78.913	10.383
ct92-M305Pete-12	120362	16/03/2013	-2	78.921	10.484
ct92-M305Pete-12	120362	16/03/2013	-2	78.914	10.359
ct92-M305Pete-12	120362	16/03/2013	-1	78.897	10.337
ct92-M305Pete-12	120362	16/03/2013	-2	78.912	10.356
ct92-M305Pete-12	120362	16/03/2013	-2	78.892	10.353

ct92-M305Pete-12	120362	16/03/2013	-2	78.897	10.315
ct92-M305Pete-12	120362	16/03/2013	1	78.889	10.352
ct92-M305Pete-12	120362	16/03/2013	-1	78.873	10.389
ct92-M305Pete-12	120362	16/03/2013	-1	78.884	10.388
ct92-M305Pete-12	120362	16/03/2013	-1	78.876	10.384
ct92-M305Pete-12	120362	16/03/2013	-1	78.866	10.357
ct92-M305Pete-12	120362	16/03/2013	0	78.864	10.342
ct92-M305Pete-12	120362	16/03/2013	2	78.86	10.361
ct92-M305Pete-12	120362	16/03/2013	-2	78.864	10.357
ct92-M305Pete-12	120362	16/03/2013	3	78.861	10.347
ct92-M305Pete-12	120362	16/03/2013	-2	78.857	10.342
ct92-M305Pete-12	120362	16/03/2013	-2	78.862	10.362
ct92-M305Pete-12	120362	16/03/2013	-2	78.858	10.389
ct92-M305Pete-12	120362	16/03/2013	-2	78.857	10.367
ct92-M305Pete-12	120362	16/03/2013	-2	78.853	10.37
ct92-M305Pete-12	120362	16/03/2013	2	78.847	10.439
ct92-M305Pete-12	120362	16/03/2013	-1	78.863	10.445
ct92-M305Pete-12	120362	16/03/2013	-1	78.876	10.359
ct92-M305Pete-12	120362	16/03/2013	-2	78.884	10.376
ct92-M305Pete-12	120362	16/03/2013	-1	78.866	10.263
ct92-M305Pete-12	120362	16/03/2013	-2	78.842	10.319
ct92-M305Pete-12	120362	16/03/2013	-2	78.843	10.323
ct92-M305Pete-12	120362	16/03/2013	-2	78.831	10.317
ct92-M305Pete-12	120362	16/03/2013	-2	78.833	10.32
ct92-M305Pete-12	120362	16/03/2013	-1	78.843	10.355
ct92-M305Pete-12	120362	16/03/2013	-2	78.824	10.336
ct92-M305Pete-12	120362	16/03/2013	-2	78.876	10.431
ct92-M305Pete-12	120362	16/03/2013	-2	78.866	10.437
ct92-M305Pete-12	120362	16/03/2013	-2	78.863	10.451
ct92-M305Pete-12	120362	16/03/2013	-2	78.853	10.444
ct92-M305Pete-12	120362	16/03/2013	-2	78.868	10.406
ct92-M305Pete-12	120362	17/03/2013	-2	78.873	10.423
ct92-M305Pete-12	120362	17/03/2013	-2	78.869	10.456
ct92-M305Pete-12	120362	17/03/2013	-1	78.876	10.414
ct92-M305Pete-12	120362	17/03/2013	-1	78.889	10.433
ct92-M305Pete-12	120362	17/03/2013	-1	78.883	10.361
ct92-M305Pete-12	120362	17/03/2013	-2	78.873	10.407
ct92-M305Pete-12	120362	17/03/2013	-2	78.904	10.414
ct92-M305Pete-12	120362	17/03/2013	-2	78.873	10.406
ct92-M305Pete-12	120362	17/03/2013	-2	78.871	10.266
ct92-M305Pete-12	120362	17/03/2013	-1	78.891	10.264
ct92-M305Pete-12	120362	17/03/2013	-1	78.898	10.342
ct92-M305Pete-12	120362	17/03/2013	-2	78.897	10.333
ct92-M305Pete-12	120362	17/03/2013	-2	78.916	10.373
ct92-M305Pete-12	120362	17/03/2013	-2	78.914	10.419
ct92-M305Pete-12	120362	17/03/2013	-2	78.916	10.414
ct92-M305Pete-12	120362	17/03/2013	-2	78.921	10.416
ct92-M305Pete-12	120362	17/03/2013	-2	78.911	10.462
ct92-M305Pete-12	120362	17/03/2013	-2	78.914	10.465
ct92-M305Pete-12	120362	17/03/2013	-2	78.921	10.498
ct92-M305Pete-12	120362	17/03/2013	-2	78.944	10.411
ct92-M305Pete-12	120362	17/03/2013	-2	78.946	10.456
ct92-M305Pete-12	120362	17/03/2013	-2	78.94	10.494
ct92-M305Pete-12	120362	17/03/2013	-2	78.945	10.468
ct92-M305Pete-12	120362	17/03/2013	-1	78.936	10.647
ct92-M305Pete-12	120362	17/03/2013	-2	78.935	10.645
ct92-M305Pete-12	120362	17/03/2013	-2	78.935	10.648
ct92-M305Pete-12	120362	17/03/2013	-1	78.925	10.564
ct92-M305Pete-12	120362	17/03/2013	-1	78.935	10.524
ct92-M305Pete-12	120362	17/03/2013	-2	78.93	10.495
ct92-M305Pete-12	120362	17/03/2013	-1	78.924	10.564
ct92-M305Pete-12	120362	17/03/2013	-2	78.927	10.576
ct92-M305Pete-12	120362	17/03/2013	-2	78.912	10.765
ct92-M305Pete-12	120362	17/03/2013	-2	78.913	10.63
ct92-M305Pete-12	120362	17/03/2013	-2	78.897	10.764
ct92-M305Pete-12	120362	17/03/2013	-2	78.907	10.794
ct92-M305Pete-12	120362	17/03/2013	-1	78.905	10.771
ct92-M305Pete-12	120362	17/03/2013	-2	78.922	10.717

ct92-M305Pete-12	120362	17/03/2013	-2	78.908	10.698
ct92-M305Pete-12	120362	17/03/2013	-2	78.923	10.723
ct92-M305Pete-12	120362	17/03/2013	-2	78.89	10.796
ct92-M305Pete-12	120362	17/03/2013	-2	78.923	10.72
ct92-M305Pete-12	120362	17/03/2013	-2	78.904	10.778
ct92-M305Pete-12	120362	17/03/2013	-2	78.892	10.79
ct92-M305Pete-12	120362	17/03/2013	-2	78.895	10.778
ct92-M305Pete-12	120362	17/03/2013	1	78.89	10.843
ct92-M305Pete-12	120362	17/03/2013	-2	78.924	10.862
ct92-M305Pete-12	120362	17/03/2013	-2	78.929	10.862
ct92-M305Pete-12	120362	17/03/2013	-2	78.925	10.832
ct92-M305Pete-12	120362	17/03/2013	-2	78.933	10.849
ct92-M305Pete-12	120362	17/03/2013	-1	78.893	10.821
ct92-M305Pete-12	120362	17/03/2013	-2	78.925	10.866
ct92-M305Pete-12	120362	17/03/2013	-2	78.93	10.881
ct92-M305Pete-12	120362	17/03/2013	-2	78.912	10.889
ct92-M305Pete-12	120362	17/03/2013	-2	78.894	10.938
ct92-M305Pete-12	120362	17/03/2013	-1	78.891	10.861
ct92-M305Pete-12	120362	17/03/2013	-1	78.89	10.82
ct92-M305Pete-12	120362	17/03/2013	-1	78.888	10.83
ct92-M305Pete-12	120362	17/03/2013	-2	78.884	10.823
ct92-M305Pete-12	120362	17/03/2013	-2	78.887	10.818
ct92-M305Pete-12	120362	17/03/2013	-1	78.889	10.824
ct92-M305Pete-12	120362	17/03/2013	-2	78.896	10.926
ct92-M305Pete-12	120362	17/03/2013	-1	78.898	10.826
ct92-M305Pete-12	120362	17/03/2013	-2	78.895	10.843
ct92-M305Pete-12	120362	17/03/2013	-1	78.898	10.856
ct92-M305Pete-12	120362	17/03/2013	-2	78.902	10.866
ct92-M305Pete-12	120362	17/03/2013	-2	78.903	10.864
ct92-M305Pete-12	120362	17/03/2013	-2	78.902	10.873
ct92-M305Pete-12	120362	17/03/2013	-2	78.906	10.867
ct92-M305Pete-12	120362	17/03/2013	-2	78.9	10.833
ct92-M305Pete-12	120362	17/03/2013	-1	78.911	10.793
ct92-M305Pete-12	120362	17/03/2013	-2	78.919	10.752
ct92-M305Pete-12	120362	17/03/2013	-1	78.922	10.741
ct92-M305Pete-12	120362	17/03/2013	-2	78.922	10.767
ct92-M305Pete-12	120362	17/03/2013	-2	78.92	10.786
ct92-M305Pete-12	120362	17/03/2013	-2	78.926	10.768
ct92-M305Pete-12	120362	17/03/2013	-2	78.926	10.716
ct92-M305Pete-12	120362	18/03/2013	-2	78.941	10.725
ct92-M305Pete-12	120362	18/03/2013	-2	78.938	10.742
ct92-M305Pete-12	120362	18/03/2013	-2	78.949	10.761
ct92-M305Pete-12	120362	18/03/2013	-2	78.945	10.796
ct92-M305Pete-12	120362	18/03/2013	-2	78.96	10.747
ct92-M305Pete-12	120362	18/03/2013	-2	78.941	10.81
ct92-M305Pete-12	120362	18/03/2013	-2	78.956	10.75
ct92-M305Pete-12	120362	18/03/2013	-2	78.971	10.708
ct92-M305Pete-12	120362	18/03/2013	-1	78.949	10.879
ct92-M305Pete-12	120362	18/03/2013	-2	78.965	10.704
ct92-M305Pete-12	120362	18/03/2013	-2	78.961	10.84
ct92-M305Pete-12	120362	18/03/2013	-2	78.96	10.803
ct92-M305Pete-12	120362	18/03/2013	-2	78.958	10.839
ct92-M305Pete-12	120362	18/03/2013	-2	78.939	10.788
ct92-M305Pete-12	120362	18/03/2013	-2	78.957	10.855
ct92-M305Pete-12	120362	18/03/2013	-2	78.914	10.9
ct92-M305Pete-12	120362	18/03/2013	-2	78.915	10.902
ct92-M305Pete-12	120362	18/03/2013	-2	78.94	10.89
ct92-M305Pete-12	120362	18/03/2013	-2	78.902	10.975
ct92-M305Pete-12	120362	18/03/2013	-2	78.936	10.908
ct92-M305Pete-12	120362	18/03/2013	-2	78.962	10.98
ct92-M305Pete-12	120362	18/03/2013	-2	78.956	10.969
ct92-M305Pete-12	120362	18/03/2013	-2	78.951	10.917
ct92-M305Pete-12	120362	18/03/2013	-2	78.95	10.935
ct92-M305Pete-12	120362	18/03/2013	-1	78.891	11.172
ct92-M305Pete-12	120362	18/03/2013	-2	78.877	11.186
ct92-M305Pete-12	120362	18/03/2013	-2	78.866	11.222
ct92-M305Pete-12	120362	18/03/2013	-2	78.952	11
ct92-M305Pete-12	120362	18/03/2013	-1	78.894	11.207

ct92-M305Pete-12	120362	18/03/2013	-2	78.896	11.198
ct92-M305Pete-12	120362	18/03/2013	-2	78.918	10.999
ct92-M305Pete-12	120362	18/03/2013	-2	78.831	10.841
ct92-M305Pete-12	120362	18/03/2013	-2	78.84	10.845
ct92-M305Pete-12	120362	18/03/2013	-2	78.83	10.854
ct92-M305Pete-12	120362	18/03/2013	-2	78.85	10.866
ct92-M305Pete-12	120362	18/03/2013	-2	78.855	10.866
ct92-M305Pete-12	120362	18/03/2013	-2	78.848	10.865
ct92-M305Pete-12	120362	18/03/2013	-1	78.853	10.898
ct92-M305Pete-12	120362	18/03/2013	-1	78.854	10.877
ct92-M305Pete-12	120362	18/03/2013	-2	78.896	10.92
ct92-M305Pete-12	120362	18/03/2013	-2	78.875	10.86
ct92-M305Pete-12	120362	18/03/2013	-2	78.898	10.926
ct92-M305Pete-12	120362	18/03/2013	-2	78.84	10.842
ct92-M305Pete-12	120362	18/03/2013	-2	78.822	10.854
ct92-M305Pete-12	120362	18/03/2013	-2	78.853	10.924
ct92-M305Pete-12	120362	18/03/2013	-2	78.857	10.914
ct92-M305Pete-12	120362	18/03/2013	-2	78.858	10.918
ct92-M305Pete-12	120362	18/03/2013	-2	78.911	10.963
ct92-M305Pete-12	120362	18/03/2013	-2	78.85	10.915
ct92-M305Pete-12	120362	18/03/2013	-2	78.894	10.838
ct92-M305Pete-12	120362	18/03/2013	-1	78.943	11.018
ct92-M305Pete-12	120362	18/03/2013	-1	78.945	11.036
ct92-M305Pete-12	120362	18/03/2013	-1	78.949	10.988
ct92-M305Pete-12	120362	18/03/2013	-2	78.947	11.044
ct92-M305Pete-12	120362	18/03/2013	-2	78.836	10.866
ct92-M305Pete-12	120362	18/03/2013	-2	78.88	10.929
ct92-M305Pete-12	120362	18/03/2013	1	78.907	10.862
ct92-M305Pete-12	120362	18/03/2013	-2	78.919	10.943
ct92-M305Pete-12	120362	18/03/2013	-1	78.916	10.92
ct92-M305Pete-12	120362	18/03/2013	-1	78.921	10.863
ct92-M305Pete-12	120362	18/03/2013	-1	78.926	10.841
ct92-M305Pete-12	120362	18/03/2013	-1	78.929	10.933
ct92-M305Pete-12	120362	18/03/2013	-2	78.935	10.933
ct92-M305Pete-12	120362	18/03/2013	-2	78.933	10.93
ct92-M305Pete-12	120362	18/03/2013	-2	78.936	10.936
ct92-M305Pete-12	120362	18/03/2013	1	78.922	10.923
ct92-M305Pete-12	120362	18/03/2013	-1	78.913	10.832
ct92-M305Pete-12	120362	18/03/2013	-1	78.905	10.833
ct92-M305Pete-12	120362	18/03/2013	-1	78.927	10.86
ct92-M305Pete-12	120362	19/03/2013	-2	78.935	10.882
ct92-M305Pete-12	120362	19/03/2013	-2	78.934	10.882
ct92-M305Pete-12	120362	19/03/2013	-2	78.925	10.91
ct92-M305Pete-12	120362	19/03/2013	-2	78.933	10.887
ct92-M305Pete-12	120362	19/03/2013	-2	78.938	10.91
ct92-M305Pete-12	120362	19/03/2013	-2	78.93	10.875
ct92-M305Pete-12	120362	19/03/2013	-2	78.934	10.886
ct92-M305Pete-12	120362	19/03/2013	-2	78.934	10.886
ct92-M305Pete-12	120362	19/03/2013	-2	78.933	10.884
ct92-M305Pete-12	120362	19/03/2013	-1	78.923	10.951
ct92-M305Pete-12	120362	19/03/2013	-2	78.934	10.948
ct92-M305Pete-12	120362	19/03/2013	-2	78.934	10.933
ct92-M305Pete-12	120362	19/03/2013	-2	78.918	10.935
ct92-M305Pete-12	120362	19/03/2013	-2	78.924	10.918
ct92-M305Pete-12	120362	19/03/2013	-2	78.949	10.863
ct92-M305Pete-12	120362	19/03/2013	-2	78.929	10.935
ct92-M305Pete-12	120362	19/03/2013	-2	78.917	10.859
ct92-M305Pete-12	120362	19/03/2013	-2	78.923	10.84
ct92-M305Pete-12	120362	19/03/2013	-2	78.924	10.84
ct92-M305Pete-12	120362	19/03/2013	-2	78.918	10.833
ct92-M305Pete-12	120362	19/03/2013	-1	78.898	10.986
ct92-M305Pete-12	120362	19/03/2013	-2	78.914	10.849
ct92-M305Pete-12	120362	19/03/2013	-2	78.924	10.815
ct92-M305Pete-12	120362	19/03/2013	-2	78.924	10.816
ct92-M305Pete-12	120362	19/03/2013	-2	78.892	11.014
ct92-M305Pete-12	120362	19/03/2013	-1	78.902	10.861
ct92-M305Pete-12	120362	19/03/2013	-1	78.902	10.79
ct92-M305Pete-12	120362	19/03/2013	-1	78.91	10.8

ct92-M305Pete-12	120362	19/03/2013	-2	78.916	10.828
ct92-M305Pete-12	120362	19/03/2013	-1	78.914	10.727
ct92-M305Pete-12	120362	19/03/2013	-1	78.914	10.727
ct92-M305Pete-12	120362	19/03/2013	-2	78.926	10.633
ct92-M305Pete-12	120362	19/03/2013	-2	78.908	10.797
ct92-M305Pete-12	120362	19/03/2013	-2	78.924	10.795
ct92-M305Pete-12	120362	19/03/2013	-1	78.914	10.779
ct92-M305Pete-12	120362	19/03/2013	-2	78.908	10.709
ct92-M305Pete-12	120362	19/03/2013	-2	78.894	10.786
ct92-M305Pete-12	120362	19/03/2013	-2	78.899	10.78
ct92-M305Pete-12	120362	19/03/2013	-2	78.893	10.776
ct92-M305Pete-12	120362	19/03/2013	-2	78.902	10.788
ct92-M305Pete-12	120362	19/03/2013	-2	78.903	10.765
ct92-M305Pete-12	120362	19/03/2013	-2	78.917	10.842
ct92-M305Pete-12	120362	19/03/2013	-2	78.909	10.711
ct92-M305Pete-12	120362	19/03/2013	-2	78.92	10.827
ct92-M305Pete-12	120362	19/03/2013	-2	78.924	10.7
ct92-M305Pete-12	120362	19/03/2013	-2	78.914	10.75
ct92-M305Pete-12	120362	19/03/2013	-2	78.922	10.713
ct92-M305Pete-12	120362	19/03/2013	-1	78.914	10.744
ct92-M305Pete-12	120362	19/03/2013	-1	78.922	10.761
ct92-M305Pete-12	120362	19/03/2013	-1	78.914	10.758
ct92-M305Pete-12	120362	19/03/2013	-2	78.929	10.746
ct92-M305Pete-12	120362	19/03/2013	-2	78.957	10.775
ct92-M305Pete-12	120362	19/03/2013	-1	78.961	10.772
ct92-M305Pete-12	120362	19/03/2013	-2	78.953	10.779
ct92-M305Pete-12	120362	19/03/2013	-2	78.953	10.782
ct92-M305Pete-12	120362	19/03/2013	-2	78.947	10.804
ct92-M305Pete-12	120362	19/03/2013	-2	78.964	10.734
ct92-M305Pete-12	120362	19/03/2013	-2	78.957	10.75
ct92-M305Pete-12	120362	19/03/2013	-2	78.954	10.773
ct92-M305Pete-12	120362	19/03/2013	-2	78.954	10.935
ct92-M305Pete-12	120362	19/03/2013	-2	78.959	10.762
ct92-M305Pete-12	120362	19/03/2013	-2	78.945	10.897
ct92-M305Pete-12	120362	19/03/2013	-2	78.95	10.871
ct92-M305Pete-12	120362	19/03/2013	-1	78.946	10.852
ct92-M305Pete-12	120362	19/03/2013	-2	78.934	10.908
ct92-M305Pete-12	120362	19/03/2013	-2	78.94	10.859
ct92-M305Pete-12	120362	19/03/2013	-2	78.938	10.859
ct92-M305Pete-12	120362	19/03/2013	-2	78.953	10.806
ct92-M305Pete-12	120362	19/03/2013	-2	78.93	10.814
ct92-M305Pete-12	120362	19/03/2013	-2	78.928	10.815
ct92-M305Pete-12	120362	19/03/2013	-2	78.929	10.832
ct92-M305Pete-12	120362	19/03/2013	-2	78.931	10.811
ct92-M305Pete-12	120362	19/03/2013	-2	78.931	10.81
ct92-M305Pete-12	120362	19/03/2013	-2	78.914	10.811
ct92-M305Pete-12	120362	19/03/2013	-2	78.913	10.924
ct92-M305Pete-12	120362	19/03/2013	-2	78.923	10.907
ct92-M305Pete-12	120362	19/03/2013	-2	78.919	10.92
ct92-M305Pete-12	120362	19/03/2013	-1	78.931	10.955
ct92-M305Pete-12	120362	19/03/2013	-2	78.912	10.981
ct92-M305Pete-12	120362	19/03/2013	-2	78.922	10.942
ct92-M305Pete-12	120362	19/03/2013	-2	78.901	10.829
ct92-M305Pete-12	120362	20/03/2013	-2	78.906	11.001
ct92-M305Pete-12	120362	20/03/2013	-2	78.898	11.042
ct92-M305Pete-12	120362	20/03/2013	1	78.868	11.192
ct92-M305Pete-12	120362	20/03/2013	-2	78.868	11.189
ct92-M305Pete-12	120362	20/03/2013	-2	78.866	11.205
ct92-M305Pete-12	120362	20/03/2013	-2	78.866	11.197
ct92-M305Pete-12	120362	20/03/2013	-2	78.866	11.197
ct92-M305Pete-12	120362	20/03/2013	-2	78.84	11.341
ct92-M305Pete-12	120362	20/03/2013	-2	78.826	11.406
ct92-M305Pete-12	120362	20/03/2013	-1	78.847	11.289
ct92-M305Pete-12	120362	20/03/2013	-1	78.846	11.28
ct92-M305Pete-12	120362	20/03/2013	-2	78.832	11.244
ct92-M305Pete-12	120362	20/03/2013	-2	78.82	11.311
ct92-M305Pete-12	120362	20/03/2013	-2	78.822	11.296
ct92-M305Pete-12	120362	20/03/2013	-1	78.778	11.399

ct92-M305Pete-12	120362	20/03/2013	-1	78.791	11.313
ct92-M305Pete-12	120362	20/03/2013	-2	78.768	11.401
ct92-M305Pete-12	120362	20/03/2013	-2	78.736	11.526
ct92-M305Pete-12	120362	20/03/2013	-2	78.704	11.471
ct92-M305Pete-12	120362	20/03/2013	-2	78.718	11.59
ct92-M305Pete-12	120362	20/03/2013	-2	78.72	11.564
ct92-M305Pete-12	120362	20/03/2013	-2	78.741	11.306
ct92-M305Pete-12	120362	20/03/2013	-1	78.712	11.382
ct92-M305Pete-12	120362	20/03/2013	-1	78.713	11.379
ct92-M305Pete-12	120362	20/03/2013	-2	78.694	11.429
ct92-M305Pete-12	120362	20/03/2013	-1	78.714	11.753
ct92-M305Pete-12	120362	20/03/2013	-2	78.665	11.715
ct92-M305Pete-12	120362	20/03/2013	-2	78.701	11.773
ct92-M305Pete-12	120362	20/03/2013	-2	78.654	11.751
ct92-M305Pete-12	120362	20/03/2013	-1	78.64	11.75
ct92-M305Pete-12	120362	20/03/2013	0	78.645	11.824
ct92-M305Pete-12	120362	20/03/2013	-2	78.663	11.603
ct92-M305Pete-12	120362	20/03/2013	-2	78.657	11.624
ct92-M305Pete-12	120362	20/03/2013	-2	78.658	11.642
ct92-M305Pete-12	120362	20/03/2013	-2	78.606	11.9
ct92-M305Pete-12	120362	20/03/2013	-2	78.55	11.896
ct92-M305Pete-12	120362	20/03/2013	-2	78.605	11.936
ct92-M305Pete-12	120362	20/03/2013	-2	78.638	11.483
ct92-M305Pete-12	120362	20/03/2013	-2	78.634	11.488
ct92-M305Pete-12	120362	20/03/2013	-2	78.623	11.514
ct92-M305Pete-12	120362	20/03/2013	-2	78.591	11.721
ct92-M305Pete-12	120362	20/03/2013	1	78.568	11.675
ct92-M305Pete-12	120362	20/03/2013	1	78.568	11.674
ct92-M305Pete-12	120362	20/03/2013	-1	78.557	11.736
ct92-M305Pete-12	120362	20/03/2013	2	78.559	11.792
ct92-M305Pete-12	120362	20/03/2013	2	78.556	11.747
ct92-M305Pete-12	120362	20/03/2013	0	78.59	11.84
ct92-M305Pete-12	120362	20/03/2013	0	78.518	11.621
ct92-M305Pete-12	120362	20/03/2013	0	78.549	11.73
ct92-M305Pete-12	120362	20/03/2013	2	78.554	11.809
ct92-M305Pete-12	120362	20/03/2013	2	78.556	11.787
ct92-M305Pete-12	120362	20/03/2013	2	78.555	11.792
ct92-M305Pete-12	120362	20/03/2013	3	78.557	11.795
ct92-M305Pete-12	120362	20/03/2013	3	78.557	11.792
ct92-M305Pete-12	120362	20/03/2013	-2	78.369	12.238
ct92-M305Pete-12	120362	20/03/2013	-2	78.428	12.188
ct92-M305Pete-12	120362	20/03/2013	-2	78.431	12.152
ct92-M305Pete-12	120362	20/03/2013	-2	78.438	12.154
ct92-M305Pete-12	120362	20/03/2013	-2	78.423	12.179
ct92-M305Pete-12	120362	21/03/2013	-2	78.39	12.044
ct92-M305Pete-12	120362	21/03/2013	-2	78.384	12.087
ct92-M305Pete-12	120362	21/03/2013	-2	78.388	12.07
ct92-M305Pete-12	120362	21/03/2013	-2	78.368	12.051
ct92-M305Pete-12	120362	21/03/2013	-2	78.357	12.049
ct92-M305Pete-12	120362	21/03/2013	-2	78.369	12.051
ct92-M305Pete-12	120362	21/03/2013	-1	78.344	12.052
ct92-M305Pete-12	120362	21/03/2013	-2	78.319	12.033
ct92-M305Pete-12	120362	21/03/2013	0	78.272	11.923
ct92-M305Pete-12	120362	21/03/2013	0	78.365	11.864
ct92-M305Pete-12	120362	21/03/2013	-2	78.294	11.841
ct92-M305Pete-12	120362	21/03/2013	-2	78.295	11.845
ct92-M305Pete-12	120362	21/03/2013	-1	78.298	11.408
ct92-M305Pete-12	120362	21/03/2013	0	78.296	11.779
ct92-M305Pete-12	120362	21/03/2013	-1	78.284	12.19
ct92-M305Pete-12	120362	21/03/2013	-1	78.267	12.195
ct92-M305Pete-12	120362	21/03/2013	-2	78.242	12.267
ct92-M305Pete-12	120362	21/03/2013	-1	78.25	12.007
ct92-M305Pete-12	120362	21/03/2013	-2	78.27	12.109
ct92-M305Pete-12	120362	21/03/2013	-1	78.263	12.102
ct92-M305Pete-12	120362	21/03/2013	-2	78.252	12.105
ct92-M305Pete-12	120362	21/03/2013	1	78.253	12.108
ct92-M305Pete-12	120362	21/03/2013	-2	78.235	12.115
ct92-M305Pete-12	120362	21/03/2013	-1	78.254	12.151

ct92-M305Pete-12	120362	21/03/2013	1	78.256	12.208
ct92-M305Pete-12	120362	21/03/2013	-1	78.256	12.212
ct92-M305Pete-12	120362	21/03/2013	0	78.25	12.25
ct92-M305Pete-12	120362	21/03/2013	0	78.26	12.224
ct92-M305Pete-12	120362	21/03/2013	-2	78.24	12.179
ct92-M305Pete-12	120362	21/03/2013	-1	78.234	12.14
ct92-M305Pete-12	120362	21/03/2013	-1	78.217	12.142
ct92-M305Pete-12	120362	21/03/2013	-1	78.231	12.275
ct92-M305Pete-12	120362	21/03/2013	-2	78.204	12.174
ct92-M305Pete-12	120362	21/03/2013	-2	78.198	12.183
ct92-M305Pete-12	120362	21/03/2013	0	78.194	12.084
ct92-M305Pete-12	120362	21/03/2013	-2	78.195	12.148
ct92-M305Pete-12	120362	21/03/2013	-2	78.184	12.168
ct92-M305Pete-12	120362	21/03/2013	-1	78.183	12.16
ct92-M305Pete-12	120362	21/03/2013	-1	78.18	12.138
ct92-M305Pete-12	120362	21/03/2013	-2	78.191	11.966
ct92-M305Pete-12	120362	21/03/2013	-2	78.184	11.949
ct92-M305Pete-12	120362	21/03/2013	-2	78.183	11.944
ct92-M305Pete-12	120362	22/03/2013	-2	78.139	11.882
ct92-M305Pete-12	120362	22/03/2013	-2	78.179	11.938
ct92-M305Pete-12	120362	22/03/2013	-2	78.141	11.871
ct92-M305Pete-12	120362	22/03/2013	-2	78.141	11.86
ct92-M305Pete-12	120362	22/03/2013	-2	78.174	11.935
ct92-M305Pete-12	120362	22/03/2013	-2	78.176	11.926
ct92-M305Pete-12	120362	22/03/2013	-2	78.181	11.955
ct92-M305Pete-12	120362	22/03/2013	-2	78.144	11.906
ct92-M305Pete-12	120362	22/03/2013	-2	78.149	11.879
ct92-M305Pete-12	120362	22/03/2013	-2	78.155	11.97
ct92-M305Pete-12	120362	22/03/2013	-2	78.176	11.905
ct92-M305Pete-12	120362	22/03/2013	-2	78.171	12.005
ct92-M305Pete-12	120362	22/03/2013	-2	78.177	11.881
ct92-M305Pete-12	120362	22/03/2013	-2	78.203	11.721
ct92-M305Pete-12	120362	22/03/2013	-2	78.219	11.727
ct92-M305Pete-12	120362	22/03/2013	-2	78.229	11.712
ct92-M305Pete-12	120362	22/03/2013	-1	78.248	11.737
ct92-M305Pete-12	120362	22/03/2013	-2	78.262	11.693
ct92-M305Pete-12	120362	22/03/2013	-2	78.259	11.69
ct92-M305Pete-12	120362	22/03/2013	-2	78.259	11.693
ct92-M305Pete-12	120362	22/03/2013	-2	78.261	11.688
ct92-M305Pete-12	120362	22/03/2013	-2	78.269	11.657
ct92-M305Pete-12	120362	22/03/2013	-2	78.279	11.745
ct92-M305Pete-12	120362	22/03/2013	-2	78.261	11.732
ct92-M305Pete-12	120362	22/03/2013	-2	78.276	11.618
ct92-M305Pete-12	120362	22/03/2013	-2	78.261	11.727
ct92-M305Pete-12	120362	22/03/2013	-2	78.278	11.687
ct92-M305Pete-12	120362	22/03/2013	-2	78.273	11.698
ct92-M305Pete-12	120362	22/03/2013	-2	78.265	11.667
ct92-M305Pete-12	120362	22/03/2013	-2	78.271	11.593
ct92-M305Pete-12	120362	22/03/2013	-2	78.283	11.515
ct92-M305Pete-12	120362	22/03/2013	-2	78.279	11.56
ct92-M305Pete-12	120362	22/03/2013	-2	78.257	11.556
ct92-M305Pete-12	120362	22/03/2013	-2	78.26	11.547
ct92-M305Pete-12	120362	22/03/2013	-2	78.291	11.587
ct92-M305Pete-12	120362	22/03/2013	-2	78.315	11.487
ct92-M305Pete-12	120362	22/03/2013	-2	78.292	11.563
ct92-M305Pete-12	120362	22/03/2013	-2	78.288	11.616
ct92-M305Pete-12	120362	22/03/2013	-2	78.287	11.601
ct92-M305Pete-12	120362	22/03/2013	-2	78.278	11.507
ct92-M305Pete-12	120362	22/03/2013	-1	78.281	11.636
ct92-M305Pete-12	120362	22/03/2013	-2	78.277	11.54
ct92-M305Pete-12	120362	22/03/2013	-1	78.263	11.67
ct92-M305Pete-12	120362	22/03/2013	-2	78.28	11.506
ct92-M305Pete-12	120362	22/03/2013	-2	78.275	11.645
ct92-M305Pete-12	120362	22/03/2013	-2	78.265	11.666
ct92-M305Pete-12	120362	22/03/2013	-2	78.275	11.645
ct92-M305Pete-12	120362	22/03/2013	-2	78.274	11.652
ct92-M305Pete-12	120362	22/03/2013	-2	78.273	11.652
ct92-M305Pete-12	120362	22/03/2013	-2	78.274	11.65

ct92-M305Pete-12	120362	23/03/2013	-2	78.271	11.64
ct92-M305Pete-12	120362	23/03/2013	-2	78.266	11.632
ct92-M305Pete-12	120362	23/03/2013	-2	78.254	11.659
ct92-M305Pete-12	120362	23/03/2013	-2	78.254	11.662
ct92-M305Pete-12	120362	23/03/2013	-2	78.236	11.599
ct92-M305Pete-12	120362	23/03/2013	-2	78.234	11.603
ct92-M305Pete-12	120362	23/03/2013	-2	78.211	11.626
ct92-M305Pete-12	120362	23/03/2013	-2	78.217	11.606
ct92-M305Pete-12	120362	23/03/2013	-2	78.225	11.66
ct92-M305Pete-12	120362	23/03/2013	-2	78.246	11.755
ct92-M305Pete-12	120362	23/03/2013	-2	78.23	11.727
ct92-M305Pete-12	120362	23/03/2013	-2	78.223	11.726
ct92-M305Pete-12	120362	23/03/2013	-2	78.232	11.742
ct92-M305Pete-12	120362	23/03/2013	-2	78.236	11.718
ct92-M305Pete-12	120362	23/03/2013	-2	78.231	11.759
ct92-M305Pete-12	120362	23/03/2013	-2	78.226	11.716
ct92-M305Pete-12	120362	23/03/2013	-2	78.254	11.78
ct92-M305Pete-12	120362	23/03/2013	-2	78.253	11.783
ct92-M305Pete-12	120362	23/03/2013	-2	78.244	11.629
ct92-M305Pete-12	120362	23/03/2013	-2	78.245	11.605
ct92-M305Pete-12	120362	23/03/2013	1	78.273	11.724
ct92-M305Pete-12	120362	23/03/2013	1	78.276	11.709
ct92-M305Pete-12	120362	23/03/2013	-1	78.27	11.697
ct92-M305Pete-12	120362	23/03/2013	-2	78.228	11.541
ct92-M305Pete-12	120362	23/03/2013	-2	78.27	11.698
ct92-M305Pete-12	120362	23/03/2013	-2	78.269	11.704
ct92-M305Pete-12	120362	23/03/2013	-1	78.278	11.627
ct92-M305Pete-12	120362	23/03/2013	-2	78.275	11.61
ct92-M305Pete-12	120362	23/03/2013	-2	78.267	11.57
ct92-M305Pete-12	120362	23/03/2013	-2	78.27	11.567
ct92-M305Pete-12	120362	23/03/2013	-2	78.289	11.554
ct92-M305Pete-12	120362	23/03/2013	-2	78.292	11.548
ct92-M305Pete-12	120362	23/03/2013	-2	78.287	11.532
ct92-M305Pete-12	120362	23/03/2013	-2	78.286	11.532
ct92-M305Pete-12	120362	23/03/2013	-2	78.28	11.367
ct92-M305Pete-12	120362	23/03/2013	-2	78.294	11.49
ct92-M305Pete-12	120362	23/03/2013	-2	78.294	11.48
ct92-M305Pete-12	120362	23/03/2013	-2	78.295	11.441
ct92-M305Pete-12	120362	23/03/2013	-2	78.318	11.31
ct92-M305Pete-12	120362	23/03/2013	-2	78.297	11.481
ct92-M305Pete-12	120362	23/03/2013	-2	78.329	11.324
ct92-M305Pete-12	120362	23/03/2013	-2	78.346	11.301
ct92-M305Pete-12	120362	23/03/2013	-1	78.341	11.41
ct92-M305Pete-12	120362	23/03/2013	-2	78.347	11.37
ct92-M305Pete-12	120362	23/03/2013	-1	78.354	11.366
ct92-M305Pete-12	120362	23/03/2013	-2	78.349	11.356
ct92-M305Pete-12	120362	23/03/2013	-2	78.35	11.343
ct92-M305Pete-12	120362	23/03/2013	-2	78.348	11.328
ct92-M305Pete-12	120362	23/03/2013	-2	78.365	11.315
ct92-M305Pete-12	120362	23/03/2013	-2	78.332	11.492
ct92-M305Pete-12	120362	23/03/2013	-2	78.331	11.5
ct92-M305Pete-12	120362	23/03/2013	-2	78.36	11.549
ct92-M305Pete-12	120362	23/03/2013	-2	78.361	11.578
ct92-M305Pete-12	120362	23/03/2013	-2	78.39	11.391
ct92-M305Pete-12	120362	23/03/2013	-2	78.379	11.553
ct92-M305Pete-12	120362	23/03/2013	-2	78.398	11.402
ct92-M305Pete-12	120362	23/03/2013	-2	78.385	11.448
ct92-M305Pete-12	120362	23/03/2013	-2	78.386	11.447
ct92-M305Pete-12	120362	23/03/2013	-2	78.387	11.443
ct92-M305Pete-12	120362	23/03/2013	-2	78.393	11.424
ct92-M305Pete-12	120362	24/03/2013	-2	78.388	11.336
ct92-M305Pete-12	120362	24/03/2013	-2	78.383	11.356
ct92-M305Pete-12	120362	24/03/2013	-2	78.39	11.353
ct92-M305Pete-12	120362	24/03/2013	-2	78.393	11.409
ct92-M305Pete-12	120362	24/03/2013	-2	78.399	11.304
ct92-M305Pete-12	120362	24/03/2013	-1	78.404	11.451
ct92-M305Pete-12	120362	24/03/2013	-1	78.401	11.34
ct92-M305Pete-12	120362	24/03/2013	-2	78.409	11.355

ct92-M305Pete-12	120362	24/03/2013	-2	78.415	11.345
ct92-M305Pete-12	120362	24/03/2013	-2	78.429	11.302
ct92-M305Pete-12	120362	24/03/2013	-2	78.436	11.331
ct92-M305Pete-12	120362	24/03/2013	-2	78.413	11.361
ct92-M305Pete-12	120362	24/03/2013	-1	78.443	11.296
ct92-M305Pete-12	120362	24/03/2013	-2	78.428	11.322
ct92-M305Pete-12	120362	24/03/2013	-2	78.447	11.288
ct92-M305Pete-12	120362	24/03/2013	-2	78.441	11.291
ct92-M305Pete-12	120362	24/03/2013	-2	78.447	11.3
ct92-M305Pete-12	120362	24/03/2013	-2	78.456	11.275
ct92-M305Pete-12	120362	24/03/2013	-2	78.443	11.198
ct92-M305Pete-12	120362	24/03/2013	-2	78.442	11.177
ct92-M305Pete-12	120362	24/03/2013	-2	78.438	11.158
ct92-M305Pete-12	120362	24/03/2013	-2	78.438	11.138
ct92-M305Pete-12	120362	24/03/2013	-2	78.43	11.139
ct92-M305Pete-12	120362	24/03/2013	-2	78.432	11.143
ct92-M305Pete-12	120362	24/03/2013	-2	78.45	11.088
ct92-M305Pete-12	120362	24/03/2013	-2	78.408	11.089
ct92-M305Pete-12	120362	24/03/2013	-2	78.41	11.118
ct92-M305Pete-12	120362	24/03/2013	-2	78.416	10.986
ct92-M305Pete-12	120362	24/03/2013	-2	78.407	10.984
ct92-M305Pete-12	120362	24/03/2013	-2	78.413	11.038
ct92-M305Pete-12	120362	24/03/2013	-2	78.432	11.005
ct92-M305Pete-12	120362	24/03/2013	-2	78.41	11.089
ct92-M305Pete-12	120362	24/03/2013	-2	78.446	11.042
ct92-M305Pete-12	120362	24/03/2013	-2	78.465	10.973
ct92-M305Pete-12	120362	24/03/2013	-2	78.464	10.966
ct92-M305Pete-12	120362	24/03/2013	-2	78.47	10.919
ct92-M305Pete-12	120362	24/03/2013	-2	78.488	10.993
ct92-M305Pete-12	120362	24/03/2013	-2	78.482	11.015
ct92-M305Pete-12	120362	24/03/2013	-2	78.501	10.948
ct92-M305Pete-12	120362	24/03/2013	-2	78.501	10.947
ct92-M305Pete-12	120362	24/03/2013	-2	78.48	10.895
ct92-M305Pete-12	120362	24/03/2013	-2	78.522	10.88
ct92-M305Pete-12	120362	24/03/2013	-2	78.484	10.858
ct92-M305Pete-12	120362	24/03/2013	-2	78.527	10.848
ct92-M305Pete-12	120362	24/03/2013	-2	78.513	10.869
ct92-M305Pete-12	120362	24/03/2013	-2	78.514	10.855
ct92-M305Pete-12	120362	24/03/2013	-2	78.521	10.794
ct92-M305Pete-12	120362	24/03/2013	-2	78.529	10.751
ct92-M305Pete-12	120362	24/03/2013	-2	78.538	10.753
ct92-M305Pete-12	120362	24/03/2013	-2	78.588	10.656
ct92-M305Pete-12	120362	24/03/2013	-2	78.543	10.747
ct92-M305Pete-12	120362	24/03/2013	-2	78.544	10.79
ct92-M305Pete-12	120362	24/03/2013	-2	78.586	10.708
ct92-M305Pete-12	120362	24/03/2013	-2	78.601	10.718
ct92-M305Pete-12	120362	24/03/2013	-2	78.603	10.615
ct92-M305Pete-12	120362	24/03/2013	-2	78.614	10.645
ct92-M305Pete-12	120362	24/03/2013	-2	78.626	10.647
ct92-M305Pete-12	120362	24/03/2013	-2	78.624	10.7
ct92-M305Pete-12	120362	24/03/2013	-2	78.608	10.688
ct92-M305Pete-12	120362	24/03/2013	-2	78.631	10.65
ct92-M305Pete-12	120362	24/03/2013	-2	78.635	10.679
ct92-M305Pete-12	120362	24/03/2013	-1	78.649	10.739
ct92-M305Pete-12	120362	24/03/2013	-1	78.65	10.747
ct92-M305Pete-12	120362	24/03/2013	-2	78.661	10.682
ct92-M305Pete-12	120362	24/03/2013	-2	78.658	10.72
ct92-M305Pete-12	120362	24/03/2013	-2	78.66	10.718
ct92-M305Pete-12	120362	24/03/2013	-1	78.661	10.682
ct92-M305Pete-12	120362	24/03/2013	-2	78.673	10.688
ct92-M305Pete-12	120362	24/03/2013	-2	78.672	10.669
ct92-M305Pete-12	120362	24/03/2013	-1	78.673	10.648
ct92-M305Pete-12	120362	24/03/2013	-2	78.674	10.672
ct92-M305Pete-12	120362	24/03/2013	-1	78.671	10.843
ct92-M305Pete-12	120362	24/03/2013	-2	78.657	10.76
ct92-M305Pete-12	120362	25/03/2013	-2	78.677	10.831
ct92-M305Pete-12	120362	25/03/2013	-1	78.683	10.738
ct92-M305Pete-12	120362	25/03/2013	-2	78.694	10.756

ct92-M305Pete-12	120362	25/03/2013	-2	78.689	10.71
ct92-M305Pete-12	120362	25/03/2013	-1	78.681	10.593
ct92-M305Pete-12	120362	25/03/2013	-2	78.678	10.664
ct92-M305Pete-12	120362	25/03/2013	-2	78.7	10.501
ct92-M305Pete-12	120362	25/03/2013	-2	78.697	10.554
ct92-M305Pete-12	120362	25/03/2013	-2	78.697	10.555
ct92-M305Pete-12	120362	25/03/2013	-2	78.705	10.556
ct92-M305Pete-12	120362	25/03/2013	-2	78.697	10.555
ct92-M305Pete-12	120362	25/03/2013	-2	78.711	10.505
ct92-M305Pete-12	120362	25/03/2013	-2	78.712	10.533
ct92-M305Pete-12	120362	25/03/2013	-2	78.726	10.47
ct92-M305Pete-12	120362	25/03/2013	-2	78.729	10.467
ct92-M305Pete-12	120362	25/03/2013	-2	78.718	10.508
ct92-M305Pete-12	120362	25/03/2013	-2	78.729	10.467
ct92-M305Pete-12	120362	25/03/2013	-1	78.726	10.45
ct92-M305Pete-12	120362	25/03/2013	3	78.725	10.46
ct92-M305Pete-12	120362	25/03/2013	-2	78.726	10.452
ct92-M305Pete-12	120362	25/03/2013	-2	78.732	10.444
ct92-M305Pete-12	120362	25/03/2013	-2	78.722	10.428
ct92-M305Pete-12	120362	25/03/2013	-2	78.723	10.419
ct92-M305Pete-12	120362	25/03/2013	-2	78.724	10.419
ct92-M305Pete-12	120362	25/03/2013	-2	78.733	10.398
ct92-M305Pete-12	120362	25/03/2013	-2	78.757	10.383
ct92-M305Pete-12	120362	25/03/2013	-2	78.744	10.376
ct92-M305Pete-12	120362	25/03/2013	1	78.782	10.35
ct92-M305Pete-12	120362	25/03/2013	-1	78.733	10.34
ct92-M305Pete-12	120362	25/03/2013	-2	78.76	10.323
ct92-M305Pete-12	120362	25/03/2013	-2	78.76	10.347
ct92-M305Pete-12	120362	25/03/2013	-2	78.761	10.447
ct92-M305Pete-12	120362	25/03/2013	-1	78.755	10.293
ct92-M305Pete-12	120362	25/03/2013	-1	78.761	10.372
ct92-M305Pete-12	120362	25/03/2013	-1	78.774	10.35
ct92-M305Pete-12	120362	25/03/2013	-1	78.781	10.385
ct92-M305Pete-12	120362	25/03/2013	1	78.738	10.387
ct92-M305Pete-12	120362	25/03/2013	-1	78.78	10.28
ct92-M305Pete-12	120362	25/03/2013	-1	78.787	10.33
ct92-M305Pete-12	120362	25/03/2013	-2	78.805	10.335
ct92-M305Pete-12	120362	25/03/2013	-2	78.802	10.322
ct92-M305Pete-12	120362	25/03/2013	-1	78.774	10.304
ct92-M305Pete-12	120362	25/03/2013	-1	78.787	10.308
ct92-M305Pete-12	120362	25/03/2013	0	78.799	10.296
ct92-M305Pete-12	120362	25/03/2013	0	78.782	10.325
ct92-M305Pete-12	120362	25/03/2013	0	78.778	10.326
ct92-M305Pete-12	120362	25/03/2013	-1	78.808	10.218
ct92-M305Pete-12	120362	25/03/2013	-1	78.803	10.206
ct92-M305Pete-12	120362	25/03/2013	-2	78.836	10.241
ct92-M305Pete-12	120362	25/03/2013	-1	78.823	10.288
ct92-M305Pete-12	120362	25/03/2013	-2	78.82	10.231
ct92-M305Pete-12	120362	25/03/2013	-2	78.824	10.279
ct92-M305Pete-12	120362	25/03/2013	-1	78.822	10.29
ct92-M305Pete-12	120362	25/03/2013	-2	78.84	10.313
ct92-M305Pete-12	120362	25/03/2013	-2	78.841	10.26
ct92-M305Pete-12	120362	25/03/2013	-2	78.845	10.308
ct92-M305Pete-12	120362	25/03/2013	-2	78.844	10.305
ct92-M305Pete-12	120362	25/03/2013	-2	78.851	10.304
ct92-M305Pete-12	120362	25/03/2013	-1	78.847	10.318
ct92-M305Pete-12	120362	25/03/2013	-1	78.848	10.278
ct92-M305Pete-12	120362	25/03/2013	-1	78.861	10.432
ct92-M305Pete-12	120362	25/03/2013	-1	78.869	10.322
ct92-M305Pete-12	120362	25/03/2013	-2	78.862	10.319
ct92-M305Pete-12	120362	25/03/2013	-2	78.861	10.291
ct92-M305Pete-12	120362	25/03/2013	-2	78.862	10.311
ct92-M305Pete-12	120362	25/03/2013	-2	78.869	10.318
ct92-M305Pete-12	120362	25/03/2013	-2	78.881	10.357
ct92-M305Pete-12	120362	25/03/2013	-2	78.9	10.351
ct92-M305Pete-12	120362	25/03/2013	-2	78.901	10.347
ct92-M305Pete-12	120362	25/03/2013	-2	78.896	10.369
ct92-M305Pete-12	120362	25/03/2013	-2	78.902	10.366

ct92-M305Pete-12	120362	25/03/2013	-2	78.936	10.165
ct92-M305Pete-12	120362	25/03/2013	-2	78.937	10.185
ct92-M305Pete-12	120362	25/03/2013	-2	78.914	10.447
ct92-M305Pete-12	120362	25/03/2013	-1	78.916	10.535
ct92-M305Pete-12	120362	26/03/2013	-2	78.908	10.495
ct92-M305Pete-12	120362	26/03/2013	-2	78.912	10.521
ct92-M305Pete-12	120362	26/03/2013	-2	78.907	10.491
ct92-M305Pete-12	120362	26/03/2013	-2	78.924	10.558
ct92-M305Pete-12	120362	26/03/2013	-2	78.927	10.574
ct92-M305Pete-12	120362	26/03/2013	-2	78.895	10.51
ct92-M305Pete-12	120362	26/03/2013	-2	78.894	10.533
ct92-M305Pete-12	120362	26/03/2013	-2	78.902	10.548
ct92-M305Pete-12	120362	26/03/2013	-2	78.902	10.551
ct92-M305Pete-12	120362	26/03/2013	-2	78.914	10.494
ct92-M305Pete-12	120362	26/03/2013	-2	78.908	10.589
ct92-M305Pete-12	120362	26/03/2013	-2	78.912	10.572
ct92-M305Pete-12	120362	26/03/2013	-1	78.903	10.553
ct92-M305Pete-12	120362	26/03/2013	-2	78.901	10.531
ct92-M305Pete-12	120362	26/03/2013	-2	78.9	10.517
ct92-M305Pete-12	120362	26/03/2013	-2	78.902	10.523
ct92-M305Pete-12	120362	26/03/2013	-2	78.918	10.524
ct92-M305Pete-12	120362	26/03/2013	-2	78.903	10.564
ct92-M305Pete-12	120362	26/03/2013	-2	78.908	10.582
ct92-M305Pete-12	120362	26/03/2013	-2	78.908	10.584
ct92-M305Pete-12	120362	26/03/2013	-2	78.92	10.545
ct92-M305Pete-12	120362	26/03/2013	-2	78.916	10.549
ct92-M305Pete-12	120362	26/03/2013	-2	78.914	10.551
ct92-M305Pete-12	120362	26/03/2013	-2	78.915	10.714
ct92-M305Pete-12	120362	26/03/2013	-2	78.922	10.685
ct92-M305Pete-12	120362	26/03/2013	-2	78.937	10.596
ct92-M305Pete-12	120362	26/03/2013	-2	78.931	10.588
ct92-M305Pete-12	120362	26/03/2013	-2	78.929	10.587
ct92-M305Pete-12	120362	26/03/2013	-2	78.93	10.59
ct92-M305Pete-12	120362	26/03/2013	-1	78.925	10.522
ct92-M305Pete-12	120362	26/03/2013	-1	78.931	10.521
ct92-M305Pete-12	120362	26/03/2013	-1	78.893	10.627
ct92-M305Pete-12	120362	26/03/2013	-2	78.919	10.512
ct92-M305Pete-12	120362	26/03/2013	-2	78.919	10.527
ct92-M305Pete-12	120362	26/03/2013	-1	78.918	10.5
ct92-M305Pete-12	120362	26/03/2013	-2	78.916	10.508
ct92-M305Pete-12	120362	26/03/2013	-2	78.917	10.503
ct92-M305Pete-12	120362	26/03/2013	-2	78.928	10.496
ct92-M305Pete-12	120362	26/03/2013	-2	78.918	10.515
ct92-M305Pete-12	120362	26/03/2013	-1	78.921	10.567
ct92-M305Pete-12	120362	26/03/2013	-2	78.913	10.613
ct92-M305Pete-12	120362	26/03/2013	-2	78.918	10.587
ct92-M305Pete-12	120362	26/03/2013	-1	78.919	10.582
ct92-M305Pete-12	120362	26/03/2013	-1	78.923	10.66
ct92-M305Pete-12	120362	26/03/2013	-2	78.92	10.697
ct92-M305Pete-12	120362	26/03/2013	0	78.91	10.714
ct92-M305Pete-12	120362	26/03/2013	-2	78.932	10.697
ct92-M305Pete-12	120362	26/03/2013	-2	78.931	10.694
ct92-M305Pete-12	120362	26/03/2013	-2	78.925	10.695
ct92-M305Pete-12	120362	26/03/2013	-2	78.927	10.772
ct92-M305Pete-12	120362	26/03/2013	-2	78.921	10.679
ct92-M305Pete-12	120362	26/03/2013	-2	78.919	10.681
ct92-M305Pete-12	120362	26/03/2013	-2	78.918	10.676
ct92-M305Pete-12	120362	26/03/2013	-1	78.925	10.661
ct92-M305Pete-12	120362	26/03/2013	-2	78.922	10.655
ct92-M305Pete-12	120362	26/03/2013	-2	78.926	10.627
ct92-M305Pete-12	120362	26/03/2013	-2	78.924	10.637
ct92-M305Pete-12	120362	26/03/2013	-2	78.923	10.626
ct92-M305Pete-12	120362	26/03/2013	-2	78.904	10.43
ct92-M305Pete-12	120362	26/03/2013	-2	78.904	10.459
ct92-M305Pete-12	120362	26/03/2013	-2	78.899	10.492
ct92-M305Pete-12	120362	26/03/2013	-2	78.9	10.495
ct92-M305Pete-12	120362	26/03/2013	-2	78.903	10.491
ct92-M305Pete-12	120362	26/03/2013	-2	78.904	10.501

ct92-M305Pete-12	120362	26/03/2013	-2	78.902	10.508
ct92-M305Pete-12	120362	26/03/2013	-2	78.9	10.552
ct92-M305Pete-12	120362	26/03/2013	-2	78.894	10.597
ct92-M305Pete-12	120362	26/03/2013	-2	78.9	10.605
ct92-M305Pete-12	120362	26/03/2013	-2	78.888	10.613
ct92-M305Pete-12	120362	26/03/2013	-2	78.929	10.739
ct92-M305Pete-12	120362	27/03/2013	-2	78.905	10.611
ct92-M305Pete-12	120362	27/03/2013	-2	78.905	10.61
ct92-M305Pete-12	120362	27/03/2013	-2	78.905	10.609
ct92-M305Pete-12	120362	27/03/2013	-2	78.911	10.489
ct92-M305Pete-12	120362	27/03/2013	-2	78.926	10.482
ct92-M305Pete-12	120362	27/03/2013	-2	78.91	10.586
ct92-M305Pete-12	120362	27/03/2013	-2	78.917	10.588
ct92-M305Pete-12	120362	27/03/2013	-2	78.912	10.48
ct92-M305Pete-12	120362	27/03/2013	-2	78.914	10.46
ct92-M305Pete-12	120362	27/03/2013	-2	78.917	10.442
ct92-M305Pete-12	120362	27/03/2013	-2	78.92	10.482
ct92-M305Pete-12	120362	27/03/2013	-1	78.928	10.453
ct92-M305Pete-12	120362	27/03/2013	-2	78.937	10.523
ct92-M305Pete-12	120362	27/03/2013	-2	78.922	10.605
ct92-M305Pete-12	120362	27/03/2013	-2	78.904	10.579
ct92-M305Pete-12	120362	27/03/2013	-2	78.92	10.569
ct92-M305Pete-12	120362	27/03/2013	-2	78.903	10.578
ct92-M305Pete-12	120362	27/03/2013	-2	78.912	10.564
ct92-M305Pete-12	120362	27/03/2013	-2	78.898	10.526
ct92-M305Pete-12	120362	27/03/2013	-1	78.911	10.537
ct92-M305Pete-12	120362	27/03/2013	-2	78.908	10.584
ct92-M305Pete-12	120362	27/03/2013	-2	78.915	10.575
ct92-M305Pete-12	120362	27/03/2013	-1	78.904	10.597
ct92-M305Pete-12	120362	27/03/2013	-2	78.904	10.593
ct92-M305Pete-12	120362	27/03/2013	-2	78.901	10.6
ct92-M305Pete-12	120362	27/03/2013	-1	78.903	10.609
ct92-M305Pete-12	120362	27/03/2013	-2	78.9	10.597
ct92-M305Pete-12	120362	27/03/2013	-2	78.9	10.601
ct92-M305Pete-12	120362	27/03/2013	-2	78.901	10.56
ct92-M305Pete-12	120362	27/03/2013	-1	78.917	10.609
ct92-M305Pete-12	120362	27/03/2013	-2	78.917	10.603
ct92-M305Pete-12	120362	27/03/2013	-2	78.895	10.579
ct92-M305Pete-12	120362	27/03/2013	-2	78.912	10.552
ct92-M305Pete-12	120362	27/03/2013	-2	78.91	10.55
ct92-M305Pete-12	120362	27/03/2013	-2	78.91	10.551
ct92-M305Pete-12	120362	27/03/2013	-2	78.91	10.55
ct92-M305Pete-12	120362	27/03/2013	-2	78.91	10.55
ct92-M305Pete-12	120362	27/03/2013	-2	78.899	10.491
ct92-M305Pete-12	120362	27/03/2013	-2	78.913	10.651
ct92-M305Pete-12	120362	27/03/2013	-2	78.929	10.636
ct92-M305Pete-12	120362	27/03/2013	-2	78.915	10.668
ct92-M305Pete-12	120362	27/03/2013	-2	78.917	10.675
ct92-M305Pete-12	120362	27/03/2013	-2	78.917	10.618
ct92-M305Pete-12	120362	27/03/2013	-2	78.915	10.64
ct92-M305Pete-12	120362	27/03/2013	-2	78.9	10.65
ct92-M305Pete-12	120362	27/03/2013	-2	78.885	10.652
ct92-M305Pete-12	120362	27/03/2013	-2	78.884	10.644
ct92-M305Pete-12	120362	27/03/2013	-2	78.923	10.659
ct92-M305Pete-12	120362	27/03/2013	-2	78.897	10.614
ct92-M305Pete-12	120362	27/03/2013	-2	78.897	10.607
ct92-M305Pete-12	120362	27/03/2013	-2	78.903	10.619
ct92-M305Pete-12	120362	27/03/2013	-2	78.883	10.644
ct92-M305Pete-12	120362	27/03/2013	-2	78.884	10.648
ct92-M305Pete-12	120362	27/03/2013	-2	78.908	10.603
ct92-M305Pete-12	120362	27/03/2013	-2	78.911	10.591
ct92-M305Pete-12	120362	27/03/2013	-1	78.921	10.622
ct92-M305Pete-12	120362	27/03/2013	-1	78.921	10.626
ct92-M305Pete-12	120362	27/03/2013	-1	78.918	10.566
ct92-M305Pete-12	120362	27/03/2013	-2	78.916	10.559
ct92-M305Pete-12	120362	27/03/2013	-2	78.928	10.591
ct92-M305Pete-12	120362	27/03/2013	-2	78.932	10.583
ct92-M305Pete-12	120362	27/03/2013	-2	78.93	10.598

ct92-M305Pete-12	120362	27/03/2013	-2	78.927	10.603
ct92-M305Pete-12	120362	27/03/2013	-2	78.93	10.592
ct92-M305Pete-12	120362	27/03/2013	-2	78.931	10.589
ct92-M305Pete-12	120362	27/03/2013	-2	78.925	10.602
ct92-M305Pete-12	120362	27/03/2013	-2	78.925	10.56
ct92-M305Pete-12	120362	27/03/2013	-2	78.924	10.552
ct92-M305Pete-12	120362	28/03/2013	-2	78.933	10.573
ct92-M305Pete-12	120362	28/03/2013	-2	78.934	10.58
ct92-M305Pete-12	120362	28/03/2013	-2	78.923	10.536
ct92-M305Pete-12	120362	28/03/2013	-2	78.919	10.485
ct92-M305Pete-12	120362	28/03/2013	-1	78.944	10.444
ct92-M305Pete-12	120362	28/03/2013	-2	78.949	10.436
ct92-M305Pete-12	120362	28/03/2013	-2	78.931	10.257
ct92-M305Pete-12	120362	28/03/2013	-2	78.973	10.466
ct92-M305Pete-12	120362	28/03/2013	-2	79.002	10.488
ct92-M305Pete-12	120362	28/03/2013	-2	78.95	10.643
ct92-M305Pete-12	120362	28/03/2013	-2	78.961	10.652
ct92-M305Pete-12	120362	28/03/2013	-2	78.924	10.808
ct92-M305Pete-12	120362	28/03/2013	-2	78.916	10.724
ct92-M305Pete-12	120362	28/03/2013	-2	78.922	10.689
ct92-M305Pete-12	120362	28/03/2013	-2	78.924	10.698
ct92-M305Pete-12	120362	28/03/2013	-2	78.922	10.688
ct92-M305Pete-12	120362	28/03/2013	-2	78.923	10.669
ct92-M305Pete-12	120362	28/03/2013	-2	78.912	10.732
ct92-M305Pete-12	120362	28/03/2013	-2	78.911	10.729
ct92-M305Pete-12	120362	28/03/2013	-2	78.909	10.728
ct92-M305Pete-12	120362	28/03/2013	-2	78.911	10.706
ct92-M305Pete-12	120362	28/03/2013	-2	78.914	10.655
ct92-M305Pete-12	120362	28/03/2013	-2	78.92	10.663
ct92-M305Pete-12	120362	28/03/2013	-2	78.925	10.672
ct92-M305Pete-12	120362	28/03/2013	-2	78.912	10.753
ct92-M305Pete-12	120362	28/03/2013	-2	78.927	10.733
ct92-M305Pete-12	120362	28/03/2013	-2	78.92	10.754
ct92-M305Pete-12	120362	28/03/2013	-2	78.921	10.736
ct92-M305Pete-12	120362	28/03/2013	-2	78.925	10.683
ct92-M305Pete-12	120362	28/03/2013	-2	78.928	10.704
ct92-M305Pete-12	120362	28/03/2013	-2	78.926	10.704
ct92-M305Pete-12	120362	28/03/2013	-1	78.926	10.806
ct92-M305Pete-12	120362	28/03/2013	-2	78.924	10.804
ct92-M305Pete-12	120362	28/03/2013	-2	78.927	10.812
ct92-M305Pete-12	120362	28/03/2013	-2	78.926	10.82
ct92-M305Pete-12	120362	28/03/2013	-2	78.928	10.684
ct92-M305Pete-12	120362	28/03/2013	-1	78.931	10.682
ct92-M305Pete-12	120362	28/03/2013	-2	78.924	10.714
ct92-M305Pete-12	120362	28/03/2013	-2	78.924	10.707
ct92-M305Pete-12	120362	28/03/2013	-2	78.924	10.714
ct92-M305Pete-12	120362	28/03/2013	-1	78.93	10.789
ct92-M305Pete-12	120362	28/03/2013	-2	78.918	10.937
ct92-M305Pete-12	120362	28/03/2013	-2	78.904	10.892
ct92-M305Pete-12	120362	28/03/2013	-2	78.904	10.875
ct92-M305Pete-12	120362	28/03/2013	-2	78.908	10.87
ct92-M305Pete-12	120362	28/03/2013	-1	78.911	10.816
ct92-M305Pete-12	120362	28/03/2013	-2	78.904	10.857
ct92-M305Pete-12	120362	28/03/2013	-2	78.9	10.818
ct92-M305Pete-12	120362	28/03/2013	-2	78.896	10.864
ct92-M305Pete-12	120362	28/03/2013	-2	78.896	10.787
ct92-M305Pete-12	120362	28/03/2013	-2	78.902	10.873
ct92-M305Pete-12	120362	28/03/2013	-2	78.901	10.869
ct92-M305Pete-12	120362	28/03/2013	-2	78.892	10.856
ct92-M305Pete-12	120362	28/03/2013	-2	78.901	10.871
ct92-M305Pete-12	120362	28/03/2013	-1	78.88	10.91
ct92-M305Pete-12	120362	28/03/2013	-1	78.895	10.875
ct92-M305Pete-12	120362	28/03/2013	-1	78.9	10.809
ct92-M305Pete-12	120362	28/03/2013	-2	78.897	10.806
ct92-M305Pete-12	120362	28/03/2013	-2	78.898	10.803
ct92-M305Pete-12	120362	28/03/2013	-2	78.896	10.816
ct92-M305Pete-12	120362	28/03/2013	-2	78.895	10.818
ct92-M305Pete-12	120362	28/03/2013	-2	78.896	10.817

ct92-M305Pete-12	120362	28/03/2013	-2	78.908	10.902
ct92-M305Pete-12	120362	28/03/2013	-2	78.903	10.868
ct92-M305Pete-12	120362	28/03/2013	-2	78.927	10.778
ct92-M305Pete-12	120362	28/03/2013	-2	78.927	10.774
ct92-M305Pete-12	120362	29/03/2013	-2	78.937	10.838
ct92-M305Pete-12	120362	29/03/2013	-2	78.937	10.827
ct92-M305Pete-12	120362	29/03/2013	-1	78.929	10.887
ct92-M305Pete-12	120362	29/03/2013	-2	78.93	10.889
ct92-M305Pete-12	120362	29/03/2013	-2	78.948	10.911
ct92-M305Pete-12	120362	29/03/2013	-2	78.948	10.921
ct92-M305Pete-12	120362	29/03/2013	-2	78.951	10.918
ct92-M305Pete-12	120362	29/03/2013	-2	78.949	10.874
ct92-M305Pete-12	120362	29/03/2013	-2	78.954	10.927
ct92-M305Pete-12	120362	29/03/2013	-2	78.959	10.938
ct92-M305Pete-12	120362	29/03/2013	-2	78.947	10.842
ct92-M305Pete-12	120362	29/03/2013	-2	78.942	10.884
ct92-M305Pete-12	120362	29/03/2013	-2	78.947	10.876
ct92-M305Pete-12	120362	29/03/2013	-1	78.947	10.892
ct92-M305Pete-12	120362	29/03/2013	-1	78.948	10.784
ct92-M305Pete-12	120362	29/03/2013	-1	78.946	10.791
ct92-M305Pete-12	120362	29/03/2013	-1	78.945	10.784
ct92-M305Pete-12	120362	29/03/2013	-2	78.951	10.731
ct92-M305Pete-12	120362	29/03/2013	-2	78.953	10.712
ct92-M305Pete-12	120362	29/03/2013	-2	78.965	10.45
ct92-M305Pete-12	120362	29/03/2013	-2	78.926	10.587
ct92-M305Pete-12	120362	29/03/2013	-2	78.922	10.689
ct92-M305Pete-12	120362	29/03/2013	-2	78.928	10.617
ct92-M305Pete-12	120362	29/03/2013	-2	78.927	10.62
ct92-M305Pete-12	120362	29/03/2013	-2	78.949	10.456
ct92-M305Pete-12	120362	29/03/2013	-2	78.913	10.617
ct92-M305Pete-12	120362	29/03/2013	-2	78.91	10.632
ct92-M305Pete-12	120362	29/03/2013	-2	78.91	10.636
ct92-M305Pete-12	120362	29/03/2013	-1	78.915	10.655
ct92-M305Pete-12	120362	29/03/2013	-2	78.895	10.74
ct92-M305Pete-12	120362	29/03/2013	-2	78.924	10.755
ct92-M305Pete-12	120362	29/03/2013	-1	78.919	10.702
ct92-M305Pete-12	120362	29/03/2013	-1	78.887	10.719
ct92-M305Pete-12	120362	29/03/2013	-1	78.922	10.7
ct92-M305Pete-12	120362	29/03/2013	-2	78.911	10.723
ct92-M305Pete-12	120362	29/03/2013	-2	78.903	10.743
ct92-M305Pete-12	120362	29/03/2013	-2	78.907	10.735
ct92-M305Pete-12	120362	29/03/2013	-2	78.908	10.717
ct92-M305Pete-12	120362	29/03/2013	-2	78.906	10.739
ct92-M305Pete-12	120362	29/03/2013	-2	78.903	10.743
ct92-M305Pete-12	120362	29/03/2013	-2	78.899	10.753
ct92-M305Pete-12	120362	29/03/2013	-2	78.919	10.795
ct92-M305Pete-12	120362	29/03/2013	-2	78.915	10.772
ct92-M305Pete-12	120362	29/03/2013	-1	78.926	10.932
ct92-M305Pete-12	120362	29/03/2013	-1	78.935	10.899
ct92-M305Pete-12	120362	29/03/2013	-1	78.934	10.908
ct92-M305Pete-12	120362	29/03/2013	-2	78.934	10.898
ct92-M305Pete-12	120362	29/03/2013	-2	78.947	10.917
ct92-M305Pete-12	120362	29/03/2013	-2	78.945	10.94
ct92-M305Pete-12	120362	29/03/2013	-2	78.947	10.919
ct92-M305Pete-12	120362	29/03/2013	-2	78.947	10.925
ct92-M305Pete-12	120362	29/03/2013	-2	78.932	10.942
ct92-M305Pete-12	120362	29/03/2013	-2	78.929	10.954
ct92-M305Pete-12	120362	29/03/2013	-2	78.929	10.949
ct92-M305Pete-12	120362	29/03/2013	-2	78.927	10.951
ct92-M305Pete-12	120362	29/03/2013	-2	78.927	10.967
ct92-M305Pete-12	120362	29/03/2013	-2	78.93	10.894
ct92-M305Pete-12	120362	29/03/2013	-2	78.924	10.983
ct92-M305Pete-12	120362	29/03/2013	-2	78.925	10.986
ct92-M305Pete-12	120362	30/03/2013	-2	78.918	10.915
ct92-M305Pete-12	120362	30/03/2013	-2	78.918	10.915
ct92-M305Pete-12	120362	30/03/2013	-2	78.913	10.895
ct92-M305Pete-12	120362	30/03/2013	-2	78.908	10.952
ct92-M305Pete-12	120362	30/03/2013	-2	78.903	10.944

ct92-M305Pete-12	120362	30/03/2013	-1	78.911	10.837
ct92-M305Pete-12	120362	30/03/2013	-2	78.911	10.836
ct92-M305Pete-12	120362	30/03/2013	-2	78.907	10.816
ct92-M305Pete-12	120362	30/03/2013	-2	78.901	10.874
ct92-M305Pete-12	120362	30/03/2013	-2	78.906	10.753
ct92-M305Pete-12	120362	30/03/2013	-2	78.912	10.753
ct92-M305Pete-12	120362	30/03/2013	-1	78.925	10.627
ct92-M305Pete-12	120362	30/03/2013	-2	78.902	10.699
ct92-M305Pete-12	120362	30/03/2013	-2	78.89	10.69
ct92-M305Pete-12	120362	30/03/2013	-2	78.889	10.683
ct92-M305Pete-12	120362	30/03/2013	-1	78.917	10.57
ct92-M305Pete-12	120362	30/03/2013	-1	78.879	10.536
ct92-M305Pete-12	120362	30/03/2013	-1	78.911	10.558
ct92-M305Pete-12	120362	30/03/2013	-2	78.917	10.571
ct92-M305Pete-12	120362	30/03/2013	-1	78.926	10.491
ct92-M305Pete-12	120362	30/03/2013	0	78.905	10.767
ct92-M305Pete-12	120362	30/03/2013	-2	78.924	10.411
ct92-M305Pete-12	120362	30/03/2013	-2	78.923	10.425
ct92-M305Pete-12	120362	30/03/2013	-2	78.9	10.528
ct92-M305Pete-12	120362	30/03/2013	-2	78.897	10.525
ct92-M305Pete-12	120362	30/03/2013	-1	78.885	10.332
ct92-M305Pete-12	120362	30/03/2013	-1	78.899	10.515
ct92-M305Pete-12	120362	30/03/2013	-1	78.896	10.305
ct92-M305Pete-12	120362	30/03/2013	-1	78.909	10.39
ct92-M305Pete-12	120362	30/03/2013	-2	78.911	10.334
ct92-M305Pete-12	120362	30/03/2013	2	78.899	10.402
ct92-M305Pete-12	120362	30/03/2013	-1	78.914	10.35
ct92-M305Pete-12	120362	30/03/2013	-1	78.92	10.343
ct92-M305Pete-12	120362	30/03/2013	1	78.912	10.32
ct92-M305Pete-12	120362	30/03/2013	-1	78.908	10.352
ct92-M305Pete-12	120362	30/03/2013	0	78.902	10.35
ct92-M305Pete-12	120362	30/03/2013	-2	78.891	10.319
ct92-M305Pete-12	120362	30/03/2013	-2	78.863	10.452
ct92-M305Pete-12	120362	30/03/2013	0	78.88	10.338
ct92-M305Pete-12	120362	30/03/2013	-2	78.874	10.294
ct92-M305Pete-12	120362	30/03/2013	-2	78.86	10.394
ct92-M305Pete-12	120362	30/03/2013	-2	78.849	10.354
ct92-M305Pete-12	120362	30/03/2013	-2	78.85	10.346
ct92-M305Pete-12	120362	30/03/2013	-2	78.878	10.212
ct92-M305Pete-12	120362	30/03/2013	-2	78.846	10.326
ct92-M305Pete-12	120362	30/03/2013	-2	78.853	10.354
ct92-M305Pete-12	120362	30/03/2013	-2	78.841	10.338
ct92-M305Pete-12	120362	30/03/2013	-2	78.816	10.322
ct92-M305Pete-12	120362	30/03/2013	-2	78.843	10.364
ct92-M305Pete-12	120362	30/03/2013	-2	78.83	10.328
ct92-M305Pete-12	120362	30/03/2013	-2	78.798	10.34
ct92-M305Pete-12	120362	30/03/2013	-2	78.792	10.396
ct92-M305Pete-12	120362	30/03/2013	-2	78.772	10.265
ct92-M305Pete-12	120362	30/03/2013	-2	78.78	10.366
ct92-M305Pete-12	120362	30/03/2013	-2	78.779	10.352
ct92-M305Pete-12	120362	30/03/2013	-2	78.77	10.307
ct92-M305Pete-12	120362	30/03/2013	-1	78.747	10.283
ct92-M305Pete-12	120362	30/03/2013	0	78.758	10.268
ct92-M305Pete-12	120362	30/03/2013	-1	78.738	10.43
ct92-M305Pete-12	120362	30/03/2013	-2	78.737	10.311
ct92-M305Pete-12	120362	30/03/2013	-2	78.724	10.302
ct92-M305Pete-12	120362	30/03/2013	-2	78.715	10.45
ct92-M305Pete-12	120362	30/03/2013	-2	78.715	10.458
ct92-M305Pete-12	120362	30/03/2013	-2	78.703	10.339
ct92-M305Pete-12	120362	30/03/2013	-2	78.706	10.498
ct92-M305Pete-12	120362	30/03/2013	-2	78.709	10.491
ct92-M305Pete-12	120362	30/03/2013	2	78.693	10.542
ct92-M305Pete-12	120362	30/03/2013	-1	78.669	10.548
ct92-M305Pete-12	120362	30/03/2013	-2	78.68	10.503
ct92-M305Pete-12	120362	31/03/2013	-1	78.667	10.631
ct92-M305Pete-12	120362	31/03/2013	-2	78.648	10.587
ct92-M305Pete-12	120362	31/03/2013	-2	78.648	10.586
ct92-M305Pete-12	120362	31/03/2013	-2	78.644	10.606

ct92-M305Pete-12	120362	31/03/2013	2	78.639	10.644
ct92-M305Pete-12	120362	31/03/2013	-1	78.623	10.758
ct92-M305Pete-12	120362	31/03/2013	-2	78.639	10.649
ct92-M305Pete-12	120362	31/03/2013	-2	78.614	10.772
ct92-M305Pete-12	120362	31/03/2013	-2	78.62	10.678
ct92-M305Pete-12	120362	31/03/2013	-2	78.602	10.708
ct92-M305Pete-12	120362	31/03/2013	-2	78.584	10.718
ct92-M305Pete-12	120362	31/03/2013	-2	78.582	10.753
ct92-M305Pete-12	120362	31/03/2013	-1	78.559	10.839
ct92-M305Pete-12	120362	31/03/2013	-2	78.549	10.868
ct92-M305Pete-12	120362	31/03/2013	-2	78.56	10.798
ct92-M305Pete-12	120362	31/03/2013	-2	78.548	10.813
ct92-M305Pete-12	120362	31/03/2013	-2	78.543	10.82
ct92-M305Pete-12	120362	31/03/2013	-1	78.532	10.942
ct92-M305Pete-12	120362	31/03/2013	-1	78.525	10.914
ct92-M305Pete-12	120362	31/03/2013	-2	78.503	10.933
ct92-M305Pete-12	120362	31/03/2013	-2	78.501	10.953
ct92-M305Pete-12	120362	31/03/2013	-2	78.514	10.903
ct92-M305Pete-12	120362	31/03/2013	-2	78.486	10.969
ct92-M305Pete-12	120362	31/03/2013	-2	78.472	10.922
ct92-M305Pete-12	120362	31/03/2013	-2	78.486	10.983
ct92-M305Pete-12	120362	31/03/2013	-2	78.451	10.893
ct92-M305Pete-12	120362	31/03/2013	-2	78.448	10.891
ct92-M305Pete-12	120362	31/03/2013	-2	78.449	10.89
ct92-M305Pete-12	120362	31/03/2013	-2	78.432	10.906
ct92-M305Pete-12	120362	31/03/2013	-2	78.402	10.952
ct92-M305Pete-12	120362	31/03/2013	-2	78.428	10.938
ct92-M305Pete-12	120362	31/03/2013	-2	78.405	11.057
ct92-M305Pete-12	120362	31/03/2013	-2	78.413	11.038
ct92-M305Pete-12	120362	31/03/2013	-2	78.476	10.91
ct92-M305Pete-12	120362	31/03/2013	-2	78.358	10.999
ct92-M305Pete-12	120362	31/03/2013	-2	78.378	11.136
ct92-M305Pete-12	120362	31/03/2013	-2	78.317	11.246
ct92-M305Pete-12	120362	31/03/2013	-2	78.277	11.301
ct92-M305Pete-12	120362	31/03/2013	-2	78.296	11.226
ct92-M305Pete-12	120362	31/03/2013	-2	78.35	11.332
ct92-M305Pete-12	120362	31/03/2013	-2	78.345	11.35
ct92-M305Pete-12	120362	31/03/2013	-2	78.315	11.407
ct92-M305Pete-12	120362	31/03/2013	-1	78.347	11.452
ct92-M305Pete-12	120362	31/03/2013	-2	78.334	11.473
ct92-M305Pete-12	120362	31/03/2013	-2	78.346	11.45
ct92-M305Pete-12	120362	31/03/2013	-2	78.33	11.506
ct92-M305Pete-12	120362	31/03/2013	-2	78.325	11.521
ct92-M305Pete-12	120362	31/03/2013	-2	78.35	11.574
ct92-M305Pete-12	120362	31/03/2013	-2	78.359	11.553
ct92-M305Pete-12	120362	31/03/2013	-2	78.353	11.582
ct92-M305Pete-12	120362	31/03/2013	-2	78.355	11.556
ct92-M305Pete-12	120362	31/03/2013	-2	78.318	11.501
ct92-M305Pete-12	120362	31/03/2013	-2	78.378	11.46
ct92-M305Pete-12	120362	31/03/2013	-2	78.369	11.42
ct92-M305Pete-12	120362	31/03/2013	-2	78.393	11.525
ct92-M305Pete-12	120362	31/03/2013	-2	78.366	11.461
ct92-M305Pete-12	120362	31/03/2013	-2	78.373	11.406
ct92-M305Pete-12	120362	31/03/2013	-2	78.37	11.398
ct92-M305Pete-12	120362	31/03/2013	-2	78.396	11.429
ct92-M305Pete-12	120362	31/03/2013	-2	78.395	11.415
ct92-M305Pete-12	120362	31/03/2013	-2	78.419	11.324
ct92-M305Pete-12	120362	31/03/2013	-2	78.406	11.381
ct92-M305Pete-12	120362	31/03/2013	-2	78.416	11.353
ct92-M305Pete-12	120362	31/03/2013	-2	78.444	11.311
ct92-M305Pete-12	120362	01/04/2013	-2	78.439	11.242
ct92-M305Pete-12	120362	01/04/2013	-2	78.436	11.259
ct92-M305Pete-12	120362	01/04/2013	-2	78.429	11.405
ct92-M305Pete-12	120362	01/04/2013	-2	78.397	11.252
ct92-M305Pete-12	120362	01/04/2013	-2	78.402	11.228
ct92-M305Pete-12	120362	01/04/2013	-2	78.422	11.447
ct92-M305Pete-12	120362	01/04/2013	-2	78.422	11.254
ct92-M305Pete-12	120362	01/04/2013	-2	78.441	11.224

ct92-M305Pete-12	120362	01/04/2013	-2	78.422	11.248
ct92-M305Pete-12	120362	01/04/2013	-2	78.434	11.209
ct92-M305Pete-12	120362	01/04/2013	-2	78.426	11.166
ct92-M305Pete-12	120362	01/04/2013	-2	78.424	11.184
ct92-M305Pete-12	120362	01/04/2013	-2	78.433	11.143
ct92-M305Pete-12	120362	01/04/2013	-2	78.453	11.177
ct92-M305Pete-12	120362	01/04/2013	-2	78.459	11.115
ct92-M305Pete-12	120362	01/04/2013	-2	78.459	11.093
ct92-M305Pete-12	120362	01/04/2013	-2	78.438	10.974
ct92-M305Pete-12	120362	01/04/2013	-2	78.448	11.003
ct92-M305Pete-12	120362	01/04/2013	-2	78.446	10.946
ct92-M305Pete-12	120362	01/04/2013	-2	78.456	10.903
ct92-M305Pete-12	120362	01/04/2013	-2	78.448	10.972
ct92-M305Pete-12	120362	01/04/2013	-2	78.472	11.084
ct92-M305Pete-12	120362	01/04/2013	-2	78.494	10.927
ct92-M305Pete-12	120362	01/04/2013	-2	78.462	11
ct92-M305Pete-12	120362	01/04/2013	-1	78.517	11.246
ct92-M305Pete-12	120362	01/04/2013	-1	78.531	10.815
ct92-M305Pete-12	120362	01/04/2013	-1	78.543	10.733
ct92-M305Pete-12	120362	01/04/2013	-2	78.547	10.913
ct92-M305Pete-12	120362	01/04/2013	-2	78.542	10.899
ct92-M305Pete-12	120362	01/04/2013	-2	78.528	10.949
ct92-M305Pete-12	120362	01/04/2013	-2	78.542	10.927
ct92-M305Pete-12	120362	01/04/2013	-1	78.569	10.92
ct92-M305Pete-12	120362	01/04/2013	-2	78.569	10.919
ct92-M305Pete-12	120362	01/04/2013	-1	78.568	10.8
ct92-M305Pete-12	120362	01/04/2013	-1	78.567	10.534
ct92-M305Pete-12	120362	01/04/2013	-2	78.562	10.441
ct92-M305Pete-12	120362	01/04/2013	-2	78.555	10.906
ct92-M305Pete-12	120362	01/04/2013	-2	78.56	10.799
ct92-M305Pete-12	120362	01/04/2013	-1	78.572	10.501
ct92-M305Pete-12	120362	01/04/2013	-1	78.569	10.788
ct92-M305Pete-12	120362	01/04/2013	-2	78.568	10.784
ct92-M305Pete-12	120362	01/04/2013	-1	78.576	10.765
ct92-M305Pete-12	120362	01/04/2013	-2	78.576	10.748
ct92-M305Pete-12	120362	01/04/2013	-1	78.588	10.745
ct92-M305Pete-12	120362	01/04/2013	-1	78.587	10.736
ct92-M305Pete-12	120362	01/04/2013	-1	78.605	10.748
ct92-M305Pete-12	120362	01/04/2013	-1	78.61	10.716
ct92-M305Pete-12	120362	01/04/2013	-2	78.61	10.713
ct92-M305Pete-12	120362	01/04/2013	2	78.596	10.682
ct92-M305Pete-12	120362	01/04/2013	3	78.602	10.712
ct92-M305Pete-12	120362	01/04/2013	1	78.594	10.68
ct92-M305Pete-12	120362	01/04/2013	-1	78.596	10.683
ct92-M305Pete-12	120362	01/04/2013	-2	78.603	10.654
ct92-M305Pete-12	120362	01/04/2013	-2	78.602	10.661
ct92-M305Pete-12	120362	01/04/2013	-2	78.602	10.648
ct92-M305Pete-12	120362	01/04/2013	-2	78.617	10.65
ct92-M305Pete-12	120362	01/04/2013	-2	78.602	10.623
ct92-M305Pete-12	120362	01/04/2013	-2	78.63	10.65
ct92-M305Pete-12	120362	01/04/2013	1	78.642	10.699
ct92-M305Pete-12	120362	01/04/2013	-2	78.644	10.687
ct92-M305Pete-12	120362	01/04/2013	-2	78.652	10.658
ct92-M305Pete-12	120362	02/04/2013	-2	78.658	10.638
ct92-M305Pete-12	120362	02/04/2013	-2	78.653	10.673
ct92-M305Pete-12	120362	02/04/2013	-2	78.67	10.663
ct92-M305Pete-12	120362	02/04/2013	-2	78.679	10.708
ct92-M305Pete-12	120362	02/04/2013	-2	78.683	10.649
ct92-M305Pete-12	120362	02/04/2013	-2	78.698	10.638
ct92-M305Pete-12	120362	02/04/2013	-2	78.693	10.565
ct92-M305Pete-12	120362	02/04/2013	-1	78.718	10.491
ct92-M305Pete-12	120362	02/04/2013	-2	78.743	10.467
ct92-M305Pete-12	120362	02/04/2013	-2	78.737	10.483
ct92-M305Pete-12	120362	02/04/2013	-2	78.739	10.496
ct92-M305Pete-12	120362	02/04/2013	-2	78.742	10.496
ct92-M305Pete-12	120362	02/04/2013	-2	78.755	10.42
ct92-M305Pete-12	120362	02/04/2013	-2	78.742	10.342
ct92-M305Pete-12	120362	02/04/2013	-2	78.751	10.459

ct92-M305Pete-12	120362	02/04/2013	-2	78.731	10.45
ct92-M305Pete-12	120362	02/04/2013	-2	78.731	10.457
ct92-M305Pete-12	120362	02/04/2013	-2	78.733	10.457
ct92-M305Pete-12	120362	02/04/2013	-2	78.752	10.381
ct92-M305Pete-12	120362	02/04/2013	-2	78.756	10.375
ct92-M305Pete-12	120362	02/04/2013	-2	78.756	10.375
ct92-M305Pete-12	120362	02/04/2013	-1	78.703	10.406
ct92-M305Pete-12	120362	02/04/2013	-2	78.752	10.427
ct92-M305Pete-12	120362	02/04/2013	-2	78.74	10.495
ct92-M305Pete-12	120362	02/04/2013	-2	78.736	10.488
ct92-M305Pete-12	120362	02/04/2013	1	78.702	10.543
ct92-M305Pete-12	120362	02/04/2013	-1	78.701	10.537
ct92-M305Pete-12	120362	02/04/2013	0	78.712	10.497
ct92-M305Pete-12	120362	02/04/2013	-2	78.697	10.547
ct92-M305Pete-12	120362	02/04/2013	-2	78.692	10.579
ct92-M305Pete-12	120362	02/04/2013	-2	78.68	10.63
ct92-M305Pete-12	120362	02/04/2013	-2	78.667	10.649
ct92-M305Pete-12	120362	02/04/2013	-2	78.674	10.637
ct92-M305Pete-12	120362	02/04/2013	-2	78.664	10.65
ct92-M305Pete-12	120362	02/04/2013	-2	78.659	10.672
ct92-M305Pete-12	120362	02/04/2013	-2	78.67	10.738
ct92-M305Pete-12	120362	02/04/2013	-2	78.646	10.701
ct92-M305Pete-12	120362	02/04/2013	-2	78.659	10.775
ct92-M305Pete-12	120362	02/04/2013	-2	78.633	10.751
ct92-M305Pete-12	120362	02/04/2013	-2	78.673	10.537
ct92-M305Pete-12	120362	02/04/2013	-2	78.7	10.573
ct92-M305Pete-12	120362	02/04/2013	-2	78.625	10.712
ct92-M305Pete-12	120362	02/04/2013	-2	78.699	10.487
ct92-M305Pete-12	120362	02/04/2013	-2	78.694	10.521
ct92-M305Pete-12	120362	02/04/2013	-1	78.751	10.581
ct92-M305Pete-12	120362	02/04/2013	-1	78.765	10.409
ct92-M305Pete-12	120362	02/04/2013	-2	78.668	10.586
ct92-M305Pete-12	120362	02/04/2013	-2	78.773	10.433
ct92-M305Pete-12	120362	02/04/2013	-2	78.774	10.446
ct92-M305Pete-12	120362	02/04/2013	-2	78.779	10.453
ct92-M305Pete-12	120362	02/04/2013	-2	78.779	10.468
ct92-M305Pete-12	120362	02/04/2013	-2	78.773	10.451
ct92-M305Pete-12	120362	02/04/2013	-2	78.775	10.468
ct92-M305Pete-12	120362	02/04/2013	-2	78.77	10.446
ct92-M305Pete-12	120362	02/04/2013	-2	78.772	10.452
ct92-M305Pete-12	120362	02/04/2013	-2	78.789	10.371
ct92-M305Pete-12	120362	02/04/2013	-2	78.8	10.487
ct92-M305Pete-12	120362	02/04/2013	-2	78.792	10.419
ct92-M305Pete-12	120362	02/04/2013	-2	78.808	10.396
ct92-M305Pete-12	120362	02/04/2013	-2	78.8	10.441
ct92-M305Pete-12	120362	02/04/2013	-2	78.801	10.428
ct92-M305Pete-12	120362	02/04/2013	-2	78.791	10.428
ct92-M305Pete-12	120362	03/04/2013	-2	78.794	10.418
ct92-M305Pete-12	120362	03/04/2013	-2	78.794	10.446
ct92-M305Pete-12	120362	03/04/2013	-2	78.795	10.457
ct92-M305Pete-12	120362	03/04/2013	-2	78.798	10.464
ct92-M305Pete-12	120362	03/04/2013	-2	78.796	10.449
ct92-M305Pete-12	120362	03/04/2013	-2	78.795	10.447
ct92-M305Pete-12	120362	03/04/2013	2	78.799	10.417
ct92-M305Pete-12	120362	03/04/2013	-2	78.799	10.422
ct92-M305Pete-12	120362	03/04/2013	-2	78.799	10.441
ct92-M305Pete-12	120362	03/04/2013	-2	78.8	10.441
ct92-M305Pete-12	120362	03/04/2013	-2	78.809	10.486
ct92-M305Pete-12	120362	03/04/2013	-2	78.84	10.448
ct92-M305Pete-12	120362	03/04/2013	-2	78.811	10.5
ct92-M305Pete-12	120362	03/04/2013	-2	78.813	10.484
ct92-M305Pete-12	120362	03/04/2013	-2	78.813	10.481
ct92-M305Pete-12	120362	03/04/2013	-2	78.826	10.442
ct92-M305Pete-12	120362	03/04/2013	-1	78.862	10.445
ct92-M305Pete-12	120362	03/04/2013	-1	78.884	10.435
ct92-M305Pete-12	120362	03/04/2013	-2	78.884	10.415
ct92-M305Pete-12	120362	03/04/2013	-2	78.906	10.367
ct92-M305Pete-12	120362	03/04/2013	-2	78.916	10.412

ct92-M305Pete-12	120362	03/04/2013	-2	78.911	10.402
ct92-M305Pete-12	120362	03/04/2013	-2	78.917	10.415
ct92-M305Pete-12	120362	03/04/2013	-2	78.89	10.548
ct92-M305Pete-12	120362	03/04/2013	-2	78.923	10.531
ct92-M305Pete-12	120362	03/04/2013	-2	78.931	10.416
ct92-M305Pete-12	120362	03/04/2013	-2	78.954	10.475
ct92-M305Pete-12	120362	03/04/2013	-2	84.787	83.175
ct92-M305Pete-12	120362	03/04/2013	-9	81.086	23.556
ct92-M305Pete-12	120362	03/04/2013	-2	78.651	9.531
ct92-M305Pete-12	120362	03/04/2013	1	78.939	10.524
ct92-M305Pete-12	120362	03/04/2013	-2	78.935	10.509
ct92-M305Pete-12	120362	03/04/2013	-2	78.933	10.483
ct92-M305Pete-12	120362	03/04/2013	-2	78.955	10.686
ct92-M305Pete-12	120362	03/04/2013	-2	78.95	10.688
ct92-M305Pete-12	120362	03/04/2013	-2	78.951	10.692
ct92-M305Pete-12	120362	03/04/2013	-2	78.939	10.715
ct92-M305Pete-12	120362	03/04/2013	-2	78.934	10.761
ct92-M305Pete-12	120362	03/04/2013	-2	78.934	10.757
ct92-M305Pete-12	120362	03/04/2013	-2	78.907	10.746
ct92-M305Pete-12	120362	03/04/2013	0	78.953	10.98
ct92-M305Pete-12	120362	03/04/2013	2	78.936	10.922
ct92-M305Pete-12	120362	03/04/2013	-2	78.93	10.935
ct92-M305Pete-12	120362	03/04/2013	-1	78.914	11.028
ct92-M305Pete-12	120362	03/04/2013	-2	78.904	11.054
ct92-M305Pete-12	120362	03/04/2013	1	78.899	11.111
ct92-M305Pete-12	120362	03/04/2013	0	78.894	11.078
ct92-M305Pete-12	120362	03/04/2013	-1	78.909	11.091
ct92-M305Pete-12	120362	03/04/2013	-2	78.9	11.135
ct92-M305Pete-12	120362	03/04/2013	-2	78.896	11.176
ct92-M305Pete-12	120362	03/04/2013	-2	78.884	11.181
ct92-M305Pete-12	120362	03/04/2013	-2	78.875	11.241
ct92-M305Pete-12	120362	03/04/2013	-1	78.865	11.153
ct92-M305Pete-12	120362	03/04/2013	-2	78.873	11.236
ct92-M305Pete-12	120362	03/04/2013	-1	78.863	11.223
ct92-M305Pete-12	120362	03/04/2013	-2	78.86	11.243
ct92-M305Pete-12	120362	03/04/2013	-2	78.857	11.32
ct92-M305Pete-12	120362	03/04/2013	-2	78.856	11.311
ct92-M305Pete-12	120362	03/04/2013	-2	78.858	11.304
ct92-M305Pete-12	120362	03/04/2013	-2	78.841	11.386
ct92-M305Pete-12	120362	03/04/2013	-2	78.845	11.385
ct92-M305Pete-12	120362	03/04/2013	-2	78.844	11.391
ct92-M305Pete-12	120362	03/04/2013	-2	78.832	11.359
ct92-M305Pete-12	120362	03/04/2013	-1	78.825	11.484
ct92-M305Pete-12	120362	03/04/2013	-2	78.821	11.433
ct92-M305Pete-12	120362	03/04/2013	-2	78.813	11.447
ct92-M305Pete-12	120362	03/04/2013	-2	78.806	11.492
ct92-M305Pete-12	120362	03/04/2013	-2	78.798	11.479
ct92-M305Pete-12	120362	04/04/2013	-2	78.789	11.512
ct92-M305Pete-12	120362	04/04/2013	-2	78.79	11.523
ct92-M305Pete-12	120362	04/04/2013	-2	78.783	11.493
ct92-M305Pete-12	120362	04/04/2013	-1	78.76	11.493
ct92-M305Pete-12	120362	04/04/2013	-2	78.763	11.49
ct92-M305Pete-12	120362	04/04/2013	-2	78.757	11.502
ct92-M305Pete-12	120362	04/04/2013	-2	78.751	11.51
ct92-M305Pete-12	120362	04/04/2013	-2	78.75	11.469
ct92-M305Pete-12	120362	04/04/2013	-1	78.726	11.362
ct92-M305Pete-12	120362	04/04/2013	-2	78.727	11.359
ct92-M305Pete-12	120362	04/04/2013	-2	78.723	11.363
ct92-M305Pete-12	120362	04/04/2013	-2	78.69	11.342
ct92-M305Pete-12	120362	04/04/2013	-2	78.664	11.545
ct92-M305Pete-12	120362	04/04/2013	-2	78.69	11.296
ct92-M305Pete-12	120362	04/04/2013	-2	78.657	11.55
ct92-M305Pete-12	120362	04/04/2013	-2	78.628	11.684
ct92-M305Pete-12	120362	04/04/2013	-2	78.64	11.873
ct92-M305Pete-12	120362	04/04/2013	-1	78.638	11.593
ct92-M305Pete-12	120362	04/04/2013	0	78.62	11.52
ct92-M305Pete-12	120362	04/04/2013	-2	78.635	11.445
ct92-M305Pete-12	120362	04/04/2013	-2	78.637	11.388

ct92-M305Pete-12	120362	04/04/2013	-2	78.632	11.399
ct92-M305Pete-12	120362	04/04/2013	-2	78.641	11.439
ct92-M305Pete-12	120362	04/04/2013	0	78.633	11.755
ct92-M305Pete-12	120362	04/04/2013	0	78.613	11.75
ct92-M305Pete-12	120362	04/04/2013	0	78.663	11.77
ct92-M305Pete-12	120362	04/04/2013	1	78.659	11.688
ct92-M305Pete-12	120362	04/04/2013	2	78.632	11.794
ct92-M305Pete-12	120362	04/04/2013	3	78.629	11.804
ct92-M305Pete-12	120362	04/04/2013	1	78.641	11.775
ct92-M305Pete-12	120362	04/04/2013	1	78.635	11.729
ct92-M305Pete-12	120362	04/04/2013	1	78.639	11.736
ct92-M305Pete-12	120362	04/04/2013	1	78.617	11.758
ct92-M305Pete-12	120362	04/04/2013	2	78.629	11.822
ct92-M305Pete-12	120362	04/04/2013	3	78.623	11.815
ct92-M305Pete-12	120362	04/04/2013	3	78.623	11.815
ct92-M305Pete-12	120362	04/04/2013	2	78.63	11.797
ct92-M305Pete-12	120362	04/04/2013	3	78.624	11.782
ct92-M305Pete-12	120362	04/04/2013	2	78.625	11.777
ct92-M305Pete-12	120362	04/04/2013	2	78.625	11.775
ct92-M305Pete-12	120362	04/04/2013	3	78.624	11.787
ct92-M305Pete-12	120362	04/04/2013	2	78.623	11.777
ct92-M305Pete-12	120362	04/04/2013	1	78.627	11.774
ct92-M305Pete-12	120362	04/04/2013	-2	78.627	11.774
ct92-M305Pete-12	120362	04/04/2013	3	78.624	11.777
ct92-M305Pete-12	120362	04/04/2013	1	78.625	11.782
ct92-M305Pete-12	120362	04/04/2013	2	78.63	11.759
ct92-M305Pete-12	120362	04/04/2013	3	78.625	11.774
ct92-M305Pete-12	120362	04/04/2013	3	78.623	11.774
ct92-M305Pete-12	120362	04/04/2013	-1	78.595	11.756
ct92-M305Pete-12	120362	05/04/2013	-2	78.606	11.718
ct92-M305Pete-12	120362	05/04/2013	-1	78.616	11.784
ct92-M305Pete-12	120362	05/04/2013	-2	78.608	11.702
ct92-M305Pete-12	120362	05/04/2013	-1	78.609	11.691
ct92-M305Pete-12	120362	05/04/2013	-2	78.619	11.696
ct92-M305Pete-12	120362	05/04/2013	1	78.614	11.721
ct92-M305Pete-12	120362	05/04/2013	-2	78.608	11.702
ct92-M305Pete-12	120362	05/04/2013	-2	78.616	11.728
ct92-M305Pete-12	120362	05/04/2013	2	78.625	11.626
ct92-M305Pete-12	120362	05/04/2013	-1	78.653	11.575
ct92-M305Pete-12	120362	05/04/2013	-2	78.621	11.579
ct92-M305Pete-12	120362	05/04/2013	-1	78.623	11.559
ct92-M305Pete-12	120362	05/04/2013	-2	78.655	11.533
ct92-M305Pete-12	120362	05/04/2013	-1	78.637	11.674
ct92-M305Pete-12	120362	05/04/2013	-1	78.622	11.715
ct92-M305Pete-12	120362	05/04/2013	1	78.627	11.729
ct92-M305Pete-12	120362	05/04/2013	-2	78.609	11.642
ct92-M305Pete-12	120362	05/04/2013	1	78.634	11.762
ct92-M305Pete-12	120362	05/04/2013	2	78.627	11.755
ct92-M305Pete-12	120362	05/04/2013	2	78.623	11.718
ct92-M305Pete-12	120362	05/04/2013	2	78.62	11.704
ct92-M305Pete-12	120362	05/04/2013	3	78.627	11.703
ct92-M305Pete-12	120362	05/04/2013	1	78.621	11.708
ct92-M305Pete-12	120362	05/04/2013	-1	78.611	11.723
ct92-M305Pete-12	120362	05/04/2013	-1	78.624	11.786
ct92-M305Pete-12	120362	05/04/2013	-1	78.614	11.796
ct92-M305Pete-12	120362	05/04/2013	-2	78.614	11.801
ct92-M305Pete-12	120362	05/04/2013	-1	78.62	11.838
ct92-M305Pete-12	120362	05/04/2013	-2	78.62	11.839
ct92-M305Pete-12	120362	05/04/2013	-2	78.625	11.838
ct92-M305Pete-12	120362	05/04/2013	-1	78.64	11.769
ct92-M305Pete-12	120362	05/04/2013	-2	78.642	11.774
ct92-M305Pete-12	120362	05/04/2013	-1	78.631	11.778
ct92-M305Pete-12	120362	05/04/2013	-2	78.635	11.766
ct92-M305Pete-12	120362	05/04/2013	-2	78.659	11.745
ct92-M305Pete-12	120362	05/04/2013	-2	78.661	11.737
ct92-M305Pete-12	120362	05/04/2013	-2	78.661	11.732
ct92-M305Pete-12	120362	05/04/2013	-2	78.67	11.756
ct92-M305Pete-12	120362	05/04/2013	1	78.689	11.665

ct92-M305Pete-12	120362	05/04/2013	0	78.667	11.744
ct92-M305Pete-12	120362	05/04/2013	-2	78.696	11.648
ct92-M305Pete-12	120362	05/04/2013	-2	78.671	11.735
ct92-M305Pete-12	120362	05/04/2013	-2	78.684	11.763
ct92-M305Pete-12	120362	05/04/2013	-2	78.699	11.728
ct92-M305Pete-12	120362	05/04/2013	-2	78.706	11.62
ct92-M305Pete-12	120362	05/04/2013	-2	78.712	11.596
ct92-M305Pete-12	120362	05/04/2013	-2	78.701	11.613
ct92-M305Pete-12	120362	05/04/2013	-2	78.709	11.574
ct92-M305Pete-12	120362	05/04/2013	-2	78.7	11.551
ct92-M305Pete-12	120362	05/04/2013	-1	78.708	11.511
ct92-M305Pete-12	120362	05/04/2013	-2	78.715	11.479
ct92-M305Pete-12	120362	05/04/2013	-2	78.707	11.503
ct92-M305Pete-12	120362	05/04/2013	-2	78.732	11.44
ct92-M305Pete-12	120362	05/04/2013	-2	78.739	11.416
ct92-M305Pete-12	120362	05/04/2013	-2	78.737	11.42
ct92-M305Pete-12	120362	05/04/2013	-2	78.743	11.406
ct92-M305Pete-12	120362	05/04/2013	-2	78.741	11.412
ct92-M305Pete-12	120362	06/04/2013	-2	78.779	11.181
ct92-M305Pete-12	120362	06/04/2013	-2	78.784	11.159
ct92-M305Pete-12	120362	06/04/2013	-2	78.78	11.106
ct92-M305Pete-12	120362	06/04/2013	-2	78.776	11.122
ct92-M305Pete-12	120362	06/04/2013	-2	78.78	11.103
ct92-M305Pete-12	120362	06/04/2013	-2	78.801	11.007
ct92-M305Pete-12	120362	06/04/2013	-2	78.8	11.012
ct92-M305Pete-12	120362	06/04/2013	-2	78.803	10.99
ct92-M305Pete-12	120362	06/04/2013	-2	78.799	10.995
ct92-M305Pete-12	120362	06/04/2013	-2	78.832	10.872
ct92-M305Pete-12	120362	06/04/2013	-2	78.83	10.909
ct92-M305Pete-12	120362	06/04/2013	-2	78.81	11.07
ct92-M305Pete-12	120362	06/04/2013	-2	78.832	10.974
ct92-M305Pete-12	120362	06/04/2013	-2	78.821	11.012
ct92-M305Pete-12	120362	06/04/2013	-2	78.832	10.97
ct92-M305Pete-12	120362	06/04/2013	-2	78.843	10.941
ct92-M305Pete-12	120362	06/04/2013	-2	78.84	10.934
ct92-M305Pete-12	120362	06/04/2013	-2	78.833	10.979
ct92-M305Pete-12	120362	06/04/2013	-2	78.829	10.995
ct92-M305Pete-12	120362	06/04/2013	-2	78.879	10.806
ct92-M305Pete-12	120362	06/04/2013	-2	78.865	10.87
ct92-M305Pete-12	120362	06/04/2013	-2	78.836	10.963
ct92-M305Pete-12	120362	06/04/2013	-2	78.844	10.953
ct92-M305Pete-12	120362	06/04/2013	-2	78.86	11.003
ct92-M305Pete-12	120362	06/04/2013	-1	78.85	11.118
ct92-M305Pete-12	120362	06/04/2013	-2	78.862	11.087
ct92-M305Pete-12	120362	06/04/2013	-2	78.875	10.974
ct92-M305Pete-12	120362	06/04/2013	-2	78.871	10.974
ct92-M305Pete-12	120362	06/04/2013	-2	78.87	10.975
ct92-M305Pete-12	120362	06/04/2013	-1	78.871	10.927
ct92-M305Pete-12	120362	06/04/2013	-1	78.88	10.934
ct92-M305Pete-12	120362	06/04/2013	-2	78.891	10.888
ct92-M305Pete-12	120362	06/04/2013	-1	78.891	10.954
ct92-M305Pete-12	120362	06/04/2013	-2	78.886	11.039
ct92-M305Pete-12	120362	06/04/2013	-2	78.855	11.012
ct92-M305Pete-12	120362	06/04/2013	-2	78.832	11.222
ct92-M305Pete-12	120362	06/04/2013	-2	78.892	10.889
ct92-M305Pete-12	120362	06/04/2013	-2	78.82	10.872
ct92-M305Pete-12	120362	06/04/2013	-2	78.89	10.899
ct92-M305Pete-12	120362	06/04/2013	-1	78.895	10.927
ct92-M305Pete-12	120362	06/04/2013	-2	78.909	10.852
ct92-M305Pete-12	120362	06/04/2013	-2	78.9	10.884
ct92-M305Pete-12	120362	06/04/2013	-2	78.929	10.793
ct92-M305Pete-12	120362	06/04/2013	-2	78.926	10.73
ct92-M305Pete-12	120362	06/04/2013	-2	78.929	10.773
ct92-M305Pete-12	120362	06/04/2013	-2	78.928	10.815
ct92-M305Pete-12	120362	06/04/2013	-2	78.921	10.857
ct92-M305Pete-12	120362	06/04/2013	-2	78.899	10.875
ct92-M305Pete-12	120362	06/04/2013	-2	78.891	10.899
ct92-M305Pete-12	120362	06/04/2013	-2	78.929	10.73

ct92-M305Pete-12	120362	06/04/2013	-2	78.95	10.525
ct92-M305Pete-12	120362	06/04/2013	-2	78.912	10.796
ct92-M305Pete-12	120362	06/04/2013	-2	78.897	10.908
ct92-M305Pete-12	120362	06/04/2013	-2	78.895	10.906
ct92-M305Pete-12	120362	06/04/2013	-2	78.938	10.818
ct92-M305Pete-12	120362	06/04/2013	-2	78.947	10.762
ct92-M305Pete-12	120362	06/04/2013	-2	78.913	10.923
ct92-M305Pete-12	120362	06/04/2013	-2	78.911	10.85
ct92-M305Pete-12	120362	06/04/2013	-2	78.904	10.741
ct92-M305Pete-12	120362	06/04/2013	-2	78.905	10.849
ct92-M305Pete-12	120362	06/04/2013	-2	78.894	10.803
ct92-M305Pete-12	120362	06/04/2013	-2	78.894	10.76
ct92-M305Pete-12	120362	06/04/2013	-2	78.891	10.771
ct92-M305Pete-12	120362	06/04/2013	-2	78.896	10.768
ct92-M305Pete-12	120362	06/04/2013	-2	78.899	10.776
ct92-M305Pete-12	120362	06/04/2013	-2	78.902	10.775
ct92-M305Pete-12	120362	06/04/2013	-2	78.898	10.722
ct92-M305Pete-12	120362	06/04/2013	-2	78.887	10.79
ct92-M305Pete-12	120362	06/04/2013	-2	78.884	10.859
ct92-M305Pete-12	120362	07/04/2013	-1	78.884	10.893
ct92-M305Pete-12	120362	07/04/2013	-2	78.888	10.762
ct92-M305Pete-12	120362	07/04/2013	-2	78.888	10.799
ct92-M305Pete-12	120362	07/04/2013	-2	78.892	10.925
ct92-M305Pete-12	120362	07/04/2013	-2	78.886	10.812
ct92-M305Pete-12	120362	07/04/2013	-2	78.901	10.87
ct92-M305Pete-12	120362	07/04/2013	-2	78.901	10.877
ct92-M305Pete-12	120362	07/04/2013	-2	78.902	10.816
ct92-M305Pete-12	120362	07/04/2013	-2	78.901	10.817
ct92-M305Pete-12	120362	07/04/2013	-2	78.903	10.798
ct92-M305Pete-12	120362	07/04/2013	-2	78.935	10.891
ct92-M305Pete-12	120362	07/04/2013	-2	78.926	10.928
ct92-M305Pete-12	120362	07/04/2013	-2	78.909	10.863
ct92-M305Pete-12	120362	07/04/2013	-2	78.91	10.81
ct92-M305Pete-12	120362	07/04/2013	-2	78.911	10.8
ct92-M305Pete-12	120362	07/04/2013	-2	78.912	10.735
ct92-M305Pete-12	120362	07/04/2013	-2	78.924	10.752
ct92-M305Pete-12	120362	07/04/2013	-2	78.911	10.812
ct92-M305Pete-12	120362	07/04/2013	-2	78.913	10.731
ct92-M305Pete-12	120362	07/04/2013	-2	78.913	10.751
ct92-M305Pete-12	120362	07/04/2013	-2	78.913	10.744
ct92-M305Pete-12	120362	07/04/2013	-2	78.932	10.728
ct92-M305Pete-12	120362	07/04/2013	-2	78.933	10.746
ct92-M305Pete-12	120362	07/04/2013	-2	78.922	10.581
ct92-M305Pete-12	120362	07/04/2013	-2	78.941	10.351
ct92-M305Pete-12	120362	07/04/2013	-1	78.934	10.522
ct92-M305Pete-12	120362	07/04/2013	-2	78.934	10.47
ct92-M305Pete-12	120362	07/04/2013	-2	78.913	10.549
ct92-M305Pete-12	120362	07/04/2013	-2	78.908	10.517
ct92-M305Pete-12	120362	07/04/2013	-2	78.908	10.517
ct92-M305Pete-12	120362	07/04/2013	-2	78.906	10.475
ct92-M305Pete-12	120362	07/04/2013	-2	78.897	10.525
ct92-M305Pete-12	120362	07/04/2013	-2	78.906	10.552
ct92-M305Pete-12	120362	07/04/2013	-2	78.86	10.263
ct92-M305Pete-12	120362	07/04/2013	-2	78.893	10.485
ct92-M305Pete-12	120362	07/04/2013	-2	78.894	10.565
ct92-M305Pete-12	120362	07/04/2013	-2	78.912	10.634
ct92-M305Pete-12	120362	07/04/2013	-2	78.907	10.584
ct92-M305Pete-12	120362	07/04/2013	-1	78.897	10.564
ct92-M305Pete-12	120362	07/04/2013	-2	78.896	10.575
ct92-M305Pete-12	120362	07/04/2013	-2	78.898	10.594
ct92-M305Pete-12	120362	07/04/2013	-2	78.899	10.584
ct92-M305Pete-12	120362	07/04/2013	-2	78.898	10.605
ct92-M305Pete-12	120362	07/04/2013	-2	78.891	10.578
ct92-M305Pete-12	120362	07/04/2013	-2	78.916	10.605
ct92-M305Pete-12	120362	07/04/2013	-2	78.913	10.57
ct92-M305Pete-12	120362	07/04/2013	-2	78.913	10.58
ct92-M305Pete-12	120362	07/04/2013	-2	78.907	10.617
ct92-M305Pete-12	120362	07/04/2013	-2	78.924	10.649

ct92-M305Pete-12	120362	07/04/2013	-2	78.924	10.638
ct92-M305Pete-12	120362	07/04/2013	-2	78.929	10.639
ct92-M305Pete-12	120362	07/04/2013	-2	78.927	10.654
ct92-M305Pete-12	120362	07/04/2013	-2	78.933	10.628
ct92-M305Pete-12	120362	07/04/2013	-1	78.926	10.579
ct92-M305Pete-12	120362	07/04/2013	-2	78.929	10.581
ct92-M305Pete-12	120362	07/04/2013	-2	78.931	10.588
ct92-M305Pete-12	120362	07/04/2013	-2	78.928	10.603
ct92-M305Pete-12	120362	07/04/2013	-2	78.93	10.602
ct92-M305Pete-12	120362	07/04/2013	-2	78.928	10.584
ct92-M305Pete-12	120362	07/04/2013	-2	78.914	10.619
ct92-M305Pete-12	120362	07/04/2013	-2	78.915	10.608
ct92-M305Pete-12	120362	07/04/2013	-2	78.919	10.638
ct92-M305Pete-12	120362	07/04/2013	-2	78.897	10.57
ct92-M305Pete-12	120362	07/04/2013	-2	78.899	10.619
ct92-M305Pete-12	120362	08/04/2013	-2	78.904	10.498
ct92-M305Pete-12	120362	08/04/2013	-2	78.905	10.501
ct92-M305Pete-12	120362	08/04/2013	-2	78.906	10.485
ct92-M305Pete-12	120362	08/04/2013	-2	78.912	10.521
ct92-M305Pete-12	120362	08/04/2013	-2	78.897	10.593
ct92-M305Pete-12	120362	08/04/2013	-2	78.898	10.577
ct92-M305Pete-12	120362	08/04/2013	-1	78.913	10.598
ct92-M305Pete-12	120362	08/04/2013	-2	78.917	10.583
ct92-M305Pete-12	120362	08/04/2013	-2	78.917	10.58
ct92-M305Pete-12	120362	08/04/2013	-1	78.902	10.584
ct92-M305Pete-12	120362	08/04/2013	-2	78.921	10.578
ct92-M305Pete-12	120362	08/04/2013	-2	78.915	10.595
ct92-M305Pete-12	120362	08/04/2013	-2	78.925	10.578
ct92-M305Pete-12	120362	08/04/2013	-2	78.923	10.584
ct92-M305Pete-12	120362	08/04/2013	-2	78.919	10.586
ct92-M305Pete-12	120362	08/04/2013	-1	78.925	10.598
ct92-M305Pete-12	120362	08/04/2013	-2	78.927	10.596
ct92-M305Pete-12	120362	08/04/2013	-2	78.926	10.604
ct92-M305Pete-12	120362	08/04/2013	-2	78.928	10.606
ct92-M305Pete-12	120362	08/04/2013	-2	78.926	10.606
ct92-M305Pete-12	120362	08/04/2013	-2	78.918	10.631
ct92-M305Pete-12	120362	08/04/2013	-2	78.838	11.383
ct92-M305Pete-12	120362	08/04/2013	-2	78.941	10.667
ct92-M305Pete-12	120362	08/04/2013	-2	78.93	10.681
ct92-M305Pete-12	120362	08/04/2013	-2	78.92	10.649
ct92-M305Pete-12	120362	08/04/2013	-1	78.921	10.604
ct92-M305Pete-12	120362	08/04/2013	-2	78.902	10.678
ct92-M305Pete-12	120362	08/04/2013	-2	78.923	10.594
ct92-M305Pete-12	120362	08/04/2013	-2	78.907	10.631
ct92-M305Pete-12	120362	08/04/2013	-2	78.908	10.627
ct92-M305Pete-12	120362	08/04/2013	-2	78.901	10.667
ct92-M305Pete-12	120362	08/04/2013	-2	78.893	10.617
ct92-M305Pete-12	120362	08/04/2013	-2	78.902	10.673
ct92-M305Pete-12	120362	08/04/2013	-2	78.901	10.677
ct92-M305Pete-12	120362	08/04/2013	-2	78.897	10.633
ct92-M305Pete-12	120362	08/04/2013	-2	78.896	10.627
ct92-M305Pete-12	120362	08/04/2013	-2	78.919	10.612
ct92-M305Pete-12	120362	08/04/2013	-2	78.904	10.6
ct92-M305Pete-12	120362	08/04/2013	-1	78.909	10.581
ct92-M305Pete-12	120362	08/04/2013	-2	78.907	10.565
ct92-M305Pete-12	120362	08/04/2013	-2	78.906	10.556
ct92-M305Pete-12	120362	08/04/2013	-1	78.912	10.569
ct92-M305Pete-12	120362	08/04/2013	-1	78.91	10.576
ct92-M305Pete-12	120362	08/04/2013	-2	78.911	10.565
ct92-M305Pete-12	120362	08/04/2013	-2	78.909	10.567
ct92-M305Pete-12	120362	08/04/2013	-2	78.91	10.572
ct92-M305Pete-12	120362	08/04/2013	-2	78.909	10.572
ct92-M305Pete-12	120362	08/04/2013	-2	78.891	10.578
ct92-M305Pete-12	120362	08/04/2013	-2	78.907	10.554
ct92-M305Pete-12	120362	08/04/2013	-2	78.912	10.542
ct92-M305Pete-12	120362	08/04/2013	-2	78.918	10.513
ct92-M305Pete-12	120362	08/04/2013	1	78.916	10.504
ct92-M305Pete-12	120362	08/04/2013	-2	78.914	10.512

ct92-M305Pete-12	120362	08/04/2013	-2	78.909	10.512
ct92-M305Pete-12	120362	08/04/2013	-2	78.909	10.542
ct92-M305Pete-12	120362	08/04/2013	-1	78.914	10.538
ct92-M305Pete-12	120362	08/04/2013	-1	78.912	10.5
ct92-M305Pete-12	120362	08/04/2013	-2	78.909	10.5
ct92-M305Pete-12	120362	08/04/2013	-2	78.908	10.508
ct92-M305Pete-12	120362	08/04/2013	-2	78.918	10.549
ct92-M305Pete-12	120362	08/04/2013	-2	78.917	10.55
ct92-M305Pete-12	120362	08/04/2013	-2	78.899	10.52
ct92-M305Pete-12	120362	08/04/2013	-2	78.898	10.532
ct92-M305Pete-12	120362	08/04/2013	-2	78.918	10.632
ct92-M305Pete-12	120362	08/04/2013	-2	78.918	10.63
ct92-M305Pete-12	120362	08/04/2013	-2	78.892	10.6
ct92-M305Pete-12	120362	08/04/2013	-2	78.891	10.601
ct92-M305Pete-12	120362	08/04/2013	-2	78.89	10.598
ct92-M305Pete-12	120362	08/04/2013	-2	78.884	10.593
ct92-M305Pete-12	120362	08/04/2013	-2	78.912	10.594
ct92-M305Pete-12	120362	08/04/2013	-1	78.909	10.489
ct92-M305Pete-12	120362	08/04/2013	-1	78.906	10.655
ct92-M305Pete-12	120362	08/04/2013	-2	78.891	10.544
ct92-M305Pete-12	120362	09/04/2013	-2	78.891	10.537
ct92-M305Pete-12	120362	09/04/2013	-2	78.901	10.576
ct92-M305Pete-12	120362	09/04/2013	-1	78.923	10.639
ct92-M305Pete-12	120362	09/04/2013	-2	78.894	10.563
ct92-M305Pete-12	120362	09/04/2013	-2	78.922	10.597
ct92-M305Pete-12	120362	09/04/2013	-2	78.899	10.582
ct92-M305Pete-12	120362	09/04/2013	-2	78.899	10.583
ct92-M305Pete-12	120362	09/04/2013	-2	78.894	10.571
ct92-M305Pete-12	120362	09/04/2013	-2	78.9	10.553
ct92-M305Pete-12	120362	09/04/2013	-2	78.905	10.577
ct92-M305Pete-12	120362	09/04/2013	-2	78.906	10.584
ct92-M305Pete-12	120362	09/04/2013	-1	78.909	10.604
ct92-M305Pete-12	120362	09/04/2013	-2	78.933	10.548
ct92-M305Pete-12	120362	09/04/2013	-2	78.942	10.475
ct92-M305Pete-12	120362	09/04/2013	-2	78.941	10.468
ct92-M305Pete-12	120362	09/04/2013	-2	78.944	10.492
ct92-M305Pete-12	120362	09/04/2013	-1	78.924	10.597
ct92-M305Pete-12	120362	09/04/2013	-2	78.923	10.602
ct92-M305Pete-12	120362	09/04/2013	-2	78.909	10.616
ct92-M305Pete-12	120362	09/04/2013	-2	78.91	10.634
ct92-M305Pete-12	120362	09/04/2013	-2	78.91	10.611
ct92-M305Pete-12	120362	09/04/2013	-2	78.909	10.608
ct92-M305Pete-12	120362	09/04/2013	-2	78.91	10.635
ct92-M305Pete-12	120362	09/04/2013	-2	78.91	10.637
ct92-M305Pete-12	120362	09/04/2013	-2	78.915	10.645
ct92-M305Pete-12	120362	09/04/2013	-2	78.917	10.624
ct92-M305Pete-12	120362	09/04/2013	-2	78.921	10.627
ct92-M305Pete-12	120362	09/04/2013	-1	78.914	10.633
ct92-M305Pete-12	120362	09/04/2013	-2	78.913	10.626
ct92-M305Pete-12	120362	09/04/2013	-2	78.92	10.62
ct92-M305Pete-12	120362	09/04/2013	-2	78.92	10.59
ct92-M305Pete-12	120362	09/04/2013	-2	78.958	10.683
ct92-M305Pete-12	120362	09/04/2013	-2	78.903	10.614
ct92-M305Pete-12	120362	09/04/2013	1	78.939	10.89
ct92-M305Pete-12	120362	09/04/2013	-2	78.948	10.903
ct92-M305Pete-12	120362	09/04/2013	-2	78.948	10.902
ct92-M305Pete-12	120362	09/04/2013	-2	78.95	10.904
ct92-M305Pete-12	120362	09/04/2013	-2	78.922	10.701
ct92-M305Pete-12	120362	09/04/2013	-2	78.918	10.728
ct92-M305Pete-12	120362	09/04/2013	-2	78.918	10.719
ct92-M305Pete-12	120362	09/04/2013	-2	78.917	10.695
ct92-M305Pete-12	120362	09/04/2013	-2	78.916	10.65
ct92-M305Pete-12	120362	09/04/2013	-2	78.918	10.668
ct92-M305Pete-12	120362	09/04/2013	-2	78.916	10.614
ct92-M305Pete-12	120362	09/04/2013	-2	78.917	10.612
ct92-M305Pete-12	120362	09/04/2013	-2	78.918	10.597
ct92-M305Pete-12	120362	09/04/2013	-2	78.925	10.621
ct92-M305Pete-12	120362	09/04/2013	-2	78.925	10.639

ct92-M305Pete-12	120362	09/04/2013	-2	78.931	10.614
ct92-M305Pete-12	120362	09/04/2013	-2	78.929	10.599
ct92-M305Pete-12	120362	09/04/2013	-2	78.926	10.662
ct92-M305Pete-12	120362	09/04/2013	-2	78.925	10.681
ct92-M305Pete-12	120362	10/04/2013	-2	78.924	10.65
ct92-M305Pete-12	120362	10/04/2013	-2	78.925	10.605
ct92-M305Pete-12	120362	10/04/2013	-2	78.918	10.662
ct92-M305Pete-12	120362	10/04/2013	-2	78.916	10.623
ct92-M305Pete-12	120362	10/04/2013	-2	78.928	10.695
ct92-M305Pete-12	120362	10/04/2013	-2	78.923	10.711
ct92-M305Pete-12	120362	10/04/2013	-2	78.917	10.627
ct92-M305Pete-12	120362	10/04/2013	-1	78.911	10.772
ct92-M305Pete-12	120362	10/04/2013	-1	78.93	10.731
ct92-M305Pete-12	120362	10/04/2013	-2	78.91	10.78
ct92-M305Pete-12	120362	10/04/2013	-2	78.913	10.788
ct92-M305Pete-12	120362	10/04/2013	-2	78.916	10.778
ct92-M305Pete-12	120362	10/04/2013	-2	78.913	10.813
ct92-M305Pete-12	120362	10/04/2013	-2	78.91	10.75
ct92-M305Pete-12	120362	10/04/2013	-2	78.915	10.778
ct92-M305Pete-12	120362	10/04/2013	-2	78.915	10.788
ct92-M305Pete-12	120362	10/04/2013	-1	78.913	10.783
ct92-M305Pete-12	120362	10/04/2013	-2	78.935	10.681
ct92-M305Pete-12	120362	10/04/2013	-2	78.924	10.687
ct92-M305Pete-12	120362	10/04/2013	-2	78.934	10.676
ct92-M305Pete-12	120362	10/04/2013	-1	78.897	10.664
ct92-M305Pete-12	120362	10/04/2013	-2	78.892	10.639
ct92-M305Pete-12	120362	10/04/2013	-2	78.914	10.718
ct92-M305Pete-12	120362	10/04/2013	-2	78.914	10.721
ct92-M305Pete-12	120362	10/04/2013	-2	78.921	10.741
ct92-M305Pete-12	120362	10/04/2013	-1	78.916	10.724
ct92-M305Pete-12	120362	10/04/2013	-1	78.915	10.767
ct92-M305Pete-12	120362	10/04/2013	-2	78.91	10.684
ct92-M305Pete-12	120362	10/04/2013	-2	78.921	10.779
ct92-M305Pete-12	120362	10/04/2013	-1	78.866	10.465
ct92-M305Pete-12	120362	10/04/2013	-2	78.866	10.686
ct92-M305Pete-12	120362	10/04/2013	-2	78.921	10.747
ct92-M305Pete-12	120362	10/04/2013	-2	78.92	10.761
ct92-M305Pete-12	120362	10/04/2013	-2	78.92	10.77
ct92-M305Pete-12	120362	10/04/2013	-1	78.862	10.32
ct92-M305Pete-12	120362	10/04/2013	-2	78.892	10.426
ct92-M305Pete-12	120362	10/04/2013	-2	78.909	10.562
ct92-M305Pete-12	120362	10/04/2013	-2	78.908	10.564
ct92-M305Pete-12	120362	10/04/2013	-2	78.907	10.539
ct92-M305Pete-12	120362	10/04/2013	-2	78.907	10.542
ct92-M305Pete-12	120362	10/04/2013	-2	78.903	10.517
ct92-M305Pete-12	120362	10/04/2013	-2	78.902	10.525
ct92-M305Pete-12	120362	10/04/2013	-2	78.902	10.536
ct92-M305Pete-12	120362	10/04/2013	-2	78.937	10.31
ct92-M305Pete-12	120362	10/04/2013	-2	78.906	10.47
ct92-M305Pete-12	120362	10/04/2013	-2	78.904	10.457
ct92-M305Pete-12	120362	10/04/2013	-2	78.935	10.493
ct92-M305Pete-12	120362	10/04/2013	-2	78.952	10.489
ct92-M305Pete-12	120362	10/04/2013	-2	78.954	10.507
ct92-M305Pete-12	120362	10/04/2013	-2	78.942	10.387
ct92-M305Pete-12	120362	10/04/2013	-2	78.944	10.338
ct92-M305Pete-12	120362	10/04/2013	-2	78.938	10.376
ct92-M305Pete-12	120362	10/04/2013	-2	78.931	10.395
ct92-M305Pete-12	120362	11/04/2013	-2	78.927	10.493
ct92-M305Pete-12	120362	11/04/2013	-2	78.922	10.491
ct92-M305Pete-12	120362	11/04/2013	-2	78.921	10.489
ct92-M305Pete-12	120362	11/04/2013	-2	78.902	10.507
ct92-M305Pete-12	120362	11/04/2013	-2	78.895	10.516
ct92-M305Pete-12	120362	11/04/2013	-2	78.903	10.566
ct92-M305Pete-12	120362	11/04/2013	-2	78.891	10.536
ct92-M305Pete-12	120362	11/04/2013	-2	78.89	10.523
ct92-M305Pete-12	120362	11/04/2013	-2	78.885	10.527
ct92-M305Pete-12	120362	11/04/2013	-2	78.85	10.49
ct92-M305Pete-12	120362	11/04/2013	-2	78.889	10.371

ct92-M305Pete-12	120362	11/04/2013	-2	78.916	10.262
ct92-M305Pete-12	120362	11/04/2013	-2	78.875	10.492
ct92-M305Pete-12	120362	11/04/2013	-2	78.911	10.475
ct92-M305Pete-12	120362	11/04/2013	-2	78.92	10.479
ct92-M305Pete-12	120362	11/04/2013	-2	78.923	10.493
ct92-M305Pete-12	120362	11/04/2013	-2	78.924	10.478
ct92-M305Pete-12	120362	11/04/2013	-2	78.932	10.494
ct92-M305Pete-12	120362	11/04/2013	-2	78.923	10.569
ct92-M305Pete-12	120362	11/04/2013	-2	78.937	10.569
ct92-M305Pete-12	120362	11/04/2013	-2	78.934	10.588
ct92-M305Pete-12	120362	11/04/2013	-2	78.934	10.593
ct92-M305Pete-12	120362	11/04/2013	-1	78.947	10.592
ct92-M305Pete-12	120362	11/04/2013	-2	78.938	10.583
ct92-M305Pete-12	120362	11/04/2013	-2	78.94	10.587
ct92-M305Pete-12	120362	11/04/2013	-2	78.931	10.571
ct92-M305Pete-12	120362	11/04/2013	-2	78.941	10.594
ct92-M305Pete-12	120362	11/04/2013	-2	78.947	10.609
ct92-M305Pete-12	120362	11/04/2013	-1	78.932	10.621
ct92-M305Pete-12	120362	11/04/2013	-2	78.937	10.651
ct92-M305Pete-12	120362	11/04/2013	-2	78.958	10.639
ct92-M305Pete-12	120362	11/04/2013	-2	78.958	10.641
ct92-M305Pete-12	120362	11/04/2013	-2	78.951	10.639
ct92-M305Pete-12	120362	11/04/2013	-2	78.951	10.663
ct92-M305Pete-12	120362	11/04/2013	-2	78.93	10.861
ct92-M305Pete-12	120362	11/04/2013	-2	78.926	10.712
ct92-M305Pete-12	120362	11/04/2013	-2	78.911	10.904
ct92-M305Pete-12	120362	11/04/2013	-1	78.919	10.893
ct92-M305Pete-12	120362	11/04/2013	-1	78.91	10.907
ct92-M305Pete-12	120362	12/04/2013	-2	78.902	10.926
ct92-M305Pete-12	120362	12/04/2013	-2	78.902	10.922
ct92-M305Pete-12	120362	12/04/2013	-2	78.913	10.913
ct92-M305Pete-12	120362	12/04/2013	-2	78.901	10.934
ct92-M305Pete-12	120362	12/04/2013	-2	78.893	10.979
ct92-M305Pete-12	120362	12/04/2013	-2	78.896	10.98
ct92-M305Pete-12	120362	12/04/2013	-1	78.898	10.964
ct92-M305Pete-12	120362	12/04/2013	-2	78.893	11.029
ct92-M305Pete-12	120362	12/04/2013	-2	78.916	11.011
ct92-M305Pete-12	120362	12/04/2013	-2	78.905	10.854
ct92-M305Pete-12	120362	12/04/2013	-2	78.91	10.806
ct92-M305Pete-12	120362	12/04/2013	-1	78.907	11.031
ct92-M305Pete-12	120362	12/04/2013	-2	78.905	11.024
ct92-M305Pete-12	120362	12/04/2013	-2	78.905	11.038
ct92-M305Pete-12	120362	12/04/2013	-2	78.901	11
ct92-M305Pete-12	120362	12/04/2013	-2	78.904	10.9
ct92-M305Pete-12	120362	12/04/2013	-2	78.883	10.89
ct92-M305Pete-12	120362	12/04/2013	-1	78.761	10.979
ct92-M305Pete-12	120362	12/04/2013	-1	78.801	10.729
ct92-M305Pete-12	120362	12/04/2013	-2	78.9	10.858
ct92-M305Pete-12	120362	12/04/2013	-1	78.911	10.888
ct92-M305Pete-12	120362	12/04/2013	-2	78.908	10.859
ct92-M305Pete-12	120362	12/04/2013	-2	78.783	10.717
ct92-M305Pete-12	120362	12/04/2013	-2	78.871	10.816
ct92-M305Pete-12	120362	12/04/2013	-2	78.873	10.802
ct92-M305Pete-12	120362	12/04/2013	-2	78.866	10.793
ct92-M305Pete-12	120362	12/04/2013	-2	78.881	10.683
ct92-M305Pete-12	120362	12/04/2013	-1	78.918	10.603
ct92-M305Pete-12	120362	12/04/2013	-2	78.919	10.587
ct92-M305Pete-12	120362	12/04/2013	-2	78.919	10.531
ct92-M305Pete-12	120362	12/04/2013	-2	78.918	10.555
ct92-M305Pete-12	120362	12/04/2013	-2	78.898	10.358
ct92-M305Pete-12	120362	12/04/2013	-2	78.9	10.328
ct92-M305Pete-12	120362	12/04/2013	-1	78.948	10.522
ct92-M305Pete-12	120362	12/04/2013	-2	78.946	10.557
ct92-M305Pete-12	120362	12/04/2013	-2	78.941	10.63
ct92-M305Pete-12	120362	12/04/2013	-2	78.917	10.504
ct92-M305Pete-12	120362	12/04/2013	-2	78.943	10.633
ct92-M305Pete-12	120362	12/04/2013	-2	78.922	10.548
ct92-M305Pete-12	120362	12/04/2013	-2	78.971	10.702

ct92-M305Pete-12	120362	12/04/2013	-1	78.977	10.617
ct92-M305Pete-12	120362	12/04/2013	-2	78.955	10.644
ct92-M305Pete-12	120362	12/04/2013	-1	78.922	10.795
ct92-M305Pete-12	120362	12/04/2013	-2	78.908	10.804
ct92-M305Pete-12	120362	12/04/2013	-2	78.914	10.794
ct92-M305Pete-12	120362	12/04/2013	-2	78.907	10.823
ct92-M305Pete-12	120362	12/04/2013	-2	78.901	10.848
ct92-M305Pete-12	120362	12/04/2013	-2	78.906	10.877
ct92-M305Pete-12	120362	12/04/2013	-2	78.919	10.857
ct92-M305Pete-12	120362	12/04/2013	-2	78.92	10.912
ct92-M305Pete-12	120362	12/04/2013	-2	78.919	10.933
ct92-M305Pete-12	120362	12/04/2013	-2	78.919	10.867
ct92-M305Pete-12	120362	12/04/2013	-2	78.918	10.906
ct92-M305Pete-12	120362	12/04/2013	-1	78.917	10.928
ct92-M305Pete-12	120362	12/04/2013	-1	78.907	10.854
ct92-M305Pete-12	120362	12/04/2013	-2	78.905	10.886
ct92-M305Pete-12	120362	12/04/2013	-2	78.893	10.891
ct92-M305Pete-12	120362	12/04/2013	-1	78.892	11.005
ct92-M305Pete-12	120362	12/04/2013	-2	78.891	10.998
ct92-M305Pete-12	120362	13/04/2013	-2	78.89	11.015
ct92-M305Pete-12	120362	13/04/2013	-2	78.872	11.066
ct92-M305Pete-12	120362	13/04/2013	-2	78.876	11.056
ct92-M305Pete-12	120362	13/04/2013	-2	78.907	10.728
ct92-M305Pete-12	120362	13/04/2013	-2	78.891	10.783
ct92-M305Pete-12	120362	13/04/2013	-2	78.895	10.809
ct92-M305Pete-12	120362	13/04/2013	-2	78.886	10.824
ct92-M305Pete-12	120362	13/04/2013	-2	78.899	10.728
ct92-M305Pete-12	120362	13/04/2013	-2	78.883	10.761
ct92-M305Pete-12	120362	13/04/2013	-2	78.888	10.796
ct92-M305Pete-12	120362	13/04/2013	-2	78.889	10.773
ct92-M305Pete-12	120362	13/04/2013	-2	78.874	10.72
ct92-M305Pete-12	120362	13/04/2013	-2	78.878	10.604
ct92-M305Pete-12	120362	13/04/2013	-2	78.896	10.384
ct92-M305Pete-12	120362	13/04/2013	-2	78.899	10.399
ct92-M305Pete-12	120362	13/04/2013	-2	78.904	10.395
ct92-M305Pete-12	120362	13/04/2013	-2	78.908	10.287
ct92-M305Pete-12	120362	13/04/2013	-2	78.907	10.267
ct92-M305Pete-12	120362	13/04/2013	-2	78.928	10.245
ct92-M305Pete-12	120362	13/04/2013	-2	78.944	10.397
ct92-M305Pete-12	120362	13/04/2013	-2	78.938	10.353
ct92-M305Pete-12	120362	13/04/2013	-2	78.943	10.393
ct92-M305Pete-12	120362	13/04/2013	-2	78.93	10.38
ct92-M305Pete-12	120362	13/04/2013	-1	78.984	10.463
ct92-M305Pete-12	120362	13/04/2013	-2	78.989	10.47
ct92-M305Pete-12	120362	13/04/2013	-1	78.98	10.444
ct92-M305Pete-12	120362	13/04/2013	-2	78.981	10.443
ct92-M305Pete-12	120362	13/04/2013	-2	78.93	10.51
ct92-M305Pete-12	120362	13/04/2013	-2	78.986	10.452
ct92-M305Pete-12	120362	13/04/2013	-1	78.928	10.135
ct92-M305Pete-12	120362	13/04/2013	-1	78.915	10.151
ct92-M305Pete-12	120362	13/04/2013	-2	78.913	10.095
ct92-M305Pete-12	120362	13/04/2013	-2	78.902	10.296
ct92-M305Pete-12	120362	13/04/2013	-2	78.888	10.209
ct92-M305Pete-12	120362	13/04/2013	-2	78.886	10.216
ct92-M305Pete-12	120362	13/04/2013	-2	78.895	10.236
ct92-M305Pete-12	120362	13/04/2013	-2	78.896	10.217
ct92-M305Pete-12	120362	13/04/2013	-1	78.896	10.264
ct92-M305Pete-12	120362	13/04/2013	-2	78.904	10.322
ct92-M305Pete-12	120362	13/04/2013	-2	78.899	10.337
ct92-M305Pete-12	120362	13/04/2013	-2	78.877	10.267
ct92-M305Pete-12	120362	13/04/2013	-2	78.899	10.374
ct92-M305Pete-12	120362	13/04/2013	-1	78.912	10.47
ct92-M305Pete-12	120362	13/04/2013	-1	78.925	10.377
ct92-M305Pete-12	120362	13/04/2013	-2	78.925	10.389
ct92-M305Pete-12	120362	13/04/2013	-2	78.923	10.417
ct92-M305Pete-12	120362	13/04/2013	-1	78.905	10.314
ct92-M305Pete-12	120362	13/04/2013	-2	78.92	10.468
ct92-M305Pete-12	120362	13/04/2013	-2	78.906	10.416

ct92-M305Pete-12	120362	13/04/2013	-2	78.907	10.382
ct92-M305Pete-12	120362	13/04/2013	-2	78.905	10.375
ct92-M305Pete-12	120362	13/04/2013	-2	78.912	10.409
ct92-M305Pete-12	120362	13/04/2013	-2	78.909	10.372
ct92-M305Pete-12	120362	13/04/2013	-2	78.909	10.388
ct92-M305Pete-12	120362	13/04/2013	-2	78.926	10.425
ct92-M305Pete-12	120362	13/04/2013	-2	78.926	10.422
ct92-M305Pete-12	120362	14/04/2013	-2	78.926	10.457
ct92-M305Pete-12	120362	14/04/2013	-2	78.918	10.426
ct92-M305Pete-12	120362	14/04/2013	-2	78.915	10.44
ct92-M305Pete-12	120362	14/04/2013	-2	78.911	10.467
ct92-M305Pete-12	120362	14/04/2013	-2	78.89	10.406
ct92-M305Pete-12	120362	14/04/2013	-2	78.904	10.49
ct92-M305Pete-12	120362	14/04/2013	-2	78.907	10.46
ct92-M305Pete-12	120362	14/04/2013	-2	78.896	10.404
ct92-M305Pete-12	120362	14/04/2013	-2	78.892	10.433
ct92-M305Pete-12	120362	14/04/2013	-2	78.898	10.331
ct92-M305Pete-12	120362	14/04/2013	-2	78.878	10.335
ct92-M305Pete-12	120362	14/04/2013	-2	78.861	10.411
ct92-M305Pete-12	120362	14/04/2013	-2	78.876	10.384
ct92-M305Pete-12	120362	14/04/2013	-2	78.876	10.39
ct92-M305Pete-12	120362	14/04/2013	-2	78.874	10.373
ct92-M305Pete-12	120362	14/04/2013	-2	78.876	10.434
ct92-M305Pete-12	120362	14/04/2013	-2	78.863	10.444
ct92-M305Pete-12	120362	14/04/2013	-2	78.864	10.447
ct92-M305Pete-12	120362	14/04/2013	-2	78.867	10.441
ct92-M305Pete-12	120362	14/04/2013	0	78.867	10.467
ct92-M305Pete-12	120362	14/04/2013	-2	78.867	10.463
ct92-M305Pete-12	120362	14/04/2013	-2	78.867	10.467
ct92-M305Pete-12	120362	14/04/2013	-2	78.863	10.481
ct92-M305Pete-12	120362	14/04/2013	-2	78.853	10.432
ct92-M305Pete-12	120362	14/04/2013	-2	78.849	10.443
ct92-M305Pete-12	120362	14/04/2013	-2	78.851	10.441
ct92-M305Pete-12	120362	14/04/2013	-1	78.841	10.42
ct92-M305Pete-12	120362	14/04/2013	-2	78.844	10.448
ct92-M305Pete-12	120362	14/04/2013	-2	78.846	10.415
ct92-M305Pete-12	120362	14/04/2013	-2	78.842	10.455
ct92-M305Pete-12	120362	14/04/2013	-2	78.84	10.435
ct92-M305Pete-12	120362	14/04/2013	-2	78.818	10.433
ct92-M305Pete-12	120362	14/04/2013	-2	78.818	10.427
ct92-M305Pete-12	120362	14/04/2013	-2	78.816	10.423
ct92-M305Pete-12	120362	14/04/2013	-2	78.816	10.426
ct92-M305Pete-12	120362	14/04/2013	-2	78.813	10.427
ct92-M305Pete-12	120362	14/04/2013	-2	78.827	10.411
ct92-M305Pete-12	120362	14/04/2013	-2	78.834	10.411
ct92-M305Pete-12	120362	14/04/2013	-2	78.832	10.437
ct92-M305Pete-12	120362	14/04/2013	-2	78.79	10.432
ct92-M305Pete-12	120362	14/04/2013	-2	78.771	10.364
ct92-M305Pete-12	120362	14/04/2013	-2	78.771	10.367
ct92-M305Pete-12	120362	14/04/2013	-2	78.751	10.325
ct92-M305Pete-12	120362	15/04/2013	-2	78.763	10.283
ct92-M305Pete-12	120362	15/04/2013	-2	78.735	10.248
ct92-M305Pete-12	120362	15/04/2013	-2	78.79	10.407
ct92-M305Pete-12	120362	15/04/2013	-2	78.8	10.449
ct92-M305Pete-12	120362	15/04/2013	-2	78.804	10.438
ct92-M305Pete-12	120362	15/04/2013	-2	78.804	10.381
ct92-M305Pete-12	120362	15/04/2013	-2	78.807	10.356
ct92-M305Pete-12	120362	15/04/2013	-2	78.812	10.369
ct92-M305Pete-12	120362	15/04/2013	-2	78.814	10.349
ct92-M305Pete-12	120362	15/04/2013	-2	78.811	10.35
ct92-M305Pete-12	120362	15/04/2013	-2	78.814	10.353
ct92-M305Pete-12	120362	15/04/2013	-2	78.815	10.348
ct92-M305Pete-12	120362	15/04/2013	-2	78.814	10.342
ct92-M305Pete-12	120362	15/04/2013	-2	78.807	10.293
ct92-M305Pete-12	120362	15/04/2013	-2	78.799	10.257
ct92-M305Pete-12	120362	15/04/2013	-2	78.807	10.306
ct92-M305Pete-12	120362	15/04/2013	-2	78.807	10.271
ct92-M305Pete-12	120362	15/04/2013	-2	78.813	10.338

ct92-M305Pete-12	120362	15/04/2013	-2	78.808	10.226
ct92-M305Pete-12	120362	15/04/2013	-2	78.815	10.285
ct92-M305Pete-12	120362	15/04/2013	-2	78.827	10.362
ct92-M305Pete-12	120362	15/04/2013	-2	78.822	10.304
ct92-M305Pete-12	120362	15/04/2013	-2	78.866	10.331
ct92-M305Pete-12	120362	15/04/2013	-2	78.867	10.323
ct92-M305Pete-12	120362	15/04/2013	-2	78.888	10.333
ct92-M305Pete-12	120362	15/04/2013	-1	78.884	10.296
ct92-M305Pete-12	120362	15/04/2013	-1	78.888	10.327
ct92-M305Pete-12	120362	15/04/2013	-2	78.918	10.312
ct92-M305Pete-12	120362	15/04/2013	-2	78.932	10.339
ct92-M305Pete-12	120362	15/04/2013	-2	78.926	10.328
ct92-M305Pete-12	120362	15/04/2013	-2	78.948	10.351
ct92-M305Pete-12	120362	15/04/2013	-2	78.943	10.294
ct92-M305Pete-12	120362	15/04/2013	-2	79.003	10.402
ct92-M305Pete-12	120362	15/04/2013	-2	78.966	10.312
ct92-M305Pete-12	120362	15/04/2013	-2	79.049	10.544
ct92-M305Pete-12	120362	15/04/2013	-1	79.004	10.551
ct92-M305Pete-12	120362	15/04/2013	1	78.931	10.319
ct92-M305Pete-12	120362	15/04/2013	3	78.932	10.328
ct92-M305Pete-12	120362	16/04/2013	-1	78.924	10.311
ct92-M305Pete-12	120362	16/04/2013	-1	78.921	10.3
ct92-M305Pete-12	120362	16/04/2013	-2	78.926	10.318
ct92-M305Pete-12	120362	16/04/2013	-2	78.934	10.286
ct92-M305Pete-12	120362	16/04/2013	-2	78.934	10.288
ct92-M305Pete-12	120362	16/04/2013	-2	78.844	10.266
ct92-M305Pete-12	120362	16/04/2013	-2	78.843	10.269
ct92-M305Pete-12	120362	16/04/2013	-2	78.828	10.397
ct92-M305Pete-12	120362	16/04/2013	-2	78.816	10.415
ct92-M305Pete-12	120362	16/04/2013	-2	78.813	10.335
ct92-M305Pete-12	120362	16/04/2013	-2	78.813	10.333
ct92-M305Pete-12	120362	16/04/2013	-2	78.759	10.158
ct92-M305Pete-12	120362	16/04/2013	-2	78.766	10.462
ct92-M305Pete-12	120362	16/04/2013	-2	78.779	10.453
ct92-M305Pete-12	120362	16/04/2013	-2	78.754	10.574
ct92-M305Pete-12	120362	16/04/2013	-1	78.738	10.543
ct92-M305Pete-12	120362	16/04/2013	-2	78.713	10.573
ct92-M305Pete-12	120362	16/04/2013	-2	78.747	10.206
ct92-M305Pete-12	120362	16/04/2013	-1	78.738	10.346
ct92-M305Pete-12	120362	16/04/2013	-2	78.81	10.192
ct92-M305Pete-12	120362	16/04/2013	-2	78.755	10.077
ct92-M305Pete-12	120362	16/04/2013	-1	78.738	10.209
ct92-M305Pete-12	120362	16/04/2013	0	78.82	10.392
ct92-M305Pete-12	120362	16/04/2013	0	78.773	10.61
ct92-M305Pete-12	120362	16/04/2013	-1	78.752	10.465
ct92-M305Pete-12	120362	16/04/2013	-2	78.752	10.467
ct92-M305Pete-12	120362	16/04/2013	-1	78.719	10.539
ct92-M305Pete-12	120362	16/04/2013	-2	78.752	10.124
ct92-M305Pete-12	120362	16/04/2013	-2	78.741	10.751
ct92-M305Pete-12	120362	16/04/2013	-1	78.766	10.463
ct92-M305Pete-12	120362	16/04/2013	-2	78.746	10.497
ct92-M305Pete-12	120362	16/04/2013	-2	78.733	10.603
ct92-M305Pete-12	120362	16/04/2013	-2	78.734	10.586
ct92-M305Pete-12	120362	16/04/2013	-2	78.732	10.602
ct92-M305Pete-12	120362	16/04/2013	-1	78.685	10.738
ct92-M305Pete-12	120362	16/04/2013	-2	78.696	10.654
ct92-M305Pete-12	120362	16/04/2013	-2	78.699	10.63
ct92-M305Pete-12	120362	16/04/2013	-2	78.7	10.687
ct92-M305Pete-12	120362	16/04/2013	-2	78.697	10.701
ct92-M305Pete-12	120362	16/04/2013	-1	78.69	10.707
ct92-M305Pete-12	120362	16/04/2013	-2	78.643	10.644
ct92-M305Pete-12	120362	16/04/2013	-2	78.662	10.633
ct92-M305Pete-12	120362	16/04/2013	-2	78.648	10.731
ct92-M305Pete-12	120362	16/04/2013	-2	78.637	10.748
ct92-M305Pete-12	120362	16/04/2013	-2	78.638	10.749
ct92-M305Pete-12	120362	16/04/2013	-2	78.609	10.835
ct92-M305Pete-12	120362	16/04/2013	-2	78.612	10.837
ct92-M305Pete-12	120362	16/04/2013	-2	78.595	10.871

ct92-M305Pete-12	120362	16/04/2013	-2	78.594	10.864
ct92-M305Pete-12	120362	16/04/2013	-2	78.566	10.922
ct92-M305Pete-12	120362	16/04/2013	-2	78.551	11.003
ct92-M305Pete-12	120362	16/04/2013	-2	78.548	10.945
ct92-M305Pete-12	120362	16/04/2013	-2	78.487	11
ct92-M305Pete-12	120362	16/04/2013	-2	78.452	11.079
ct92-M305Pete-12	120362	16/04/2013	-2	78.455	11.067
ct92-M305Pete-12	120362	17/04/2013	-2	78.481	10.992
ct92-M305Pete-12	120362	17/04/2013	-2	78.443	11.091
ct92-M305Pete-12	120362	17/04/2013	-2	78.453	11.042
ct92-M305Pete-12	120362	17/04/2013	-2	78.449	11.053
ct92-M305Pete-12	120362	17/04/2013	-1	78.458	11.076
ct92-M305Pete-12	120362	17/04/2013	-2	78.426	11.154
ct92-M305Pete-12	120362	17/04/2013	-2	78.42	11.126
ct92-M305Pete-12	120362	17/04/2013	-2	78.439	11.075
ct92-M305Pete-12	120362	17/04/2013	-2	78.454	11.022
ct92-M305Pete-12	120362	17/04/2013	-2	78.449	11.028
ct92-M305Pete-12	120362	17/04/2013	-2	78.446	10.957
ct92-M305Pete-12	120362	17/04/2013	-2	78.471	10.907
ct92-M305Pete-12	120362	17/04/2013	-2	78.366	11.107
ct92-M305Pete-12	120362	17/04/2013	-2	78.46	10.857
ct92-M305Pete-12	120362	17/04/2013	-2	78.409	10.828
ct92-M305Pete-12	120362	17/04/2013	-2	78.345	11.237
ct92-M305Pete-12	120362	17/04/2013	-2	78.401	11.365
ct92-M305Pete-12	120362	17/04/2013	-2	78.365	11.284
ct92-M305Pete-12	120362	17/04/2013	-2	78.361	11.446
ct92-M305Pete-12	120362	17/04/2013	-2	78.373	11.428
ct92-M305Pete-12	120362	17/04/2013	-2	78.367	11.444
ct92-M305Pete-12	120362	17/04/2013	-1	78.337	11.431
ct92-M305Pete-12	120362	17/04/2013	-2	78.289	11.268
ct92-M305Pete-12	120362	17/04/2013	-2	78.278	11.152
ct92-M305Pete-12	120362	17/04/2013	-2	78.295	11.188
ct92-M305Pete-12	120362	17/04/2013	-2	78.401	11.138
ct92-M305Pete-12	120362	17/04/2013	-2	78.393	11.141
ct92-M305Pete-12	120362	17/04/2013	-2	78.324	11.253
ct92-M305Pete-12	120362	17/04/2013	-2	78.312	11.295
ct92-M305Pete-12	120362	17/04/2013	-2	78.312	11.252
ct92-M305Pete-12	120362	17/04/2013	-2	78.239	11.152
ct92-M305Pete-12	120362	17/04/2013	-2	78.369	11.402
ct92-M305Pete-12	120362	17/04/2013	-2	78.333	11.53
ct92-M305Pete-12	120362	17/04/2013	-1	78.308	11.64
ct92-M305Pete-12	120362	17/04/2013	-1	78.312	11.602
ct92-M305Pete-12	120362	17/04/2013	-2	78.322	11.575
ct92-M305Pete-12	120362	17/04/2013	-2	78.253	11.669
ct92-M305Pete-12	120362	17/04/2013	-2	78.258	11.434
ct92-M305Pete-12	120362	17/04/2013	-2	78.233	11.578
ct92-M305Pete-12	120362	17/04/2013	-2	78.297	11.48
ct92-M305Pete-12	120362	17/04/2013	-2	78.259	11.498
ct92-M305Pete-12	120362	17/04/2013	-2	78.259	11.485
ct92-M305Pete-12	120362	17/04/2013	-2	78.291	11.39
ct92-M305Pete-12	120362	17/04/2013	-2	78.249	11.482
ct92-M305Pete-12	120362	17/04/2013	-2	78.279	11.427
ct92-M305Pete-12	120362	17/04/2013	-2	78.231	11.536
ct92-M305Pete-12	120362	17/04/2013	-2	78.338	11.393
ct92-M305Pete-12	120362	17/04/2013	1	78.341	11.725
ct92-M305Pete-12	120362	17/04/2013	-2	78.285	11.743
ct92-M305Pete-12	120362	17/04/2013	-2	78.33	11.693
ct92-M305Pete-12	120362	17/04/2013	-2	78.328	11.577
ct92-M305Pete-12	120362	17/04/2013	-1	78.341	11.585
ct92-M305Pete-12	120362	17/04/2013	-2	78.321	11.671
ct92-M305Pete-12	120362	17/04/2013	-2	78.334	11.644
ct92-M305Pete-12	120362	17/04/2013	-2	78.335	11.642
ct92-M305Pete-12	120362	17/04/2013	2	78.339	11.644
ct92-M305Pete-12	120362	17/04/2013	-2	78.304	11.67
ct92-M305Pete-12	120362	17/04/2013	-2	78.323	11.675
ct92-M305Pete-12	120362	17/04/2013	-2	78.33	11.708
ct92-M305Pete-12	120362	17/04/2013	-2	78.324	11.681
ct92-M305Pete-12	120362	18/04/2013	-2	78.323	11.671

ct92-M305Pete-12	120362	18/04/2013	-2	78.314	11.669
ct92-M305Pete-12	120362	18/04/2013	-2	78.313	11.686
ct92-M305Pete-12	120362	18/04/2013	-1	78.328	11.825
ct92-M305Pete-12	120362	18/04/2013	3	78.313	11.776
ct92-M305Pete-12	120362	18/04/2013	-2	78.307	11.788
ct92-M305Pete-12	120362	18/04/2013	-2	78.286	11.835
ct92-M305Pete-12	120362	18/04/2013	-2	78.284	11.835
ct92-M305Pete-12	120362	18/04/2013	-1	78.294	11.739
ct92-M305Pete-12	120362	18/04/2013	-1	78.274	11.808
ct92-M305Pete-12	120362	18/04/2013	-2	78.295	11.737
ct92-M305Pete-12	120362	18/04/2013	-2	78.266	11.781
ct92-M305Pete-12	120362	18/04/2013	-2	78.324	11.756
ct94-M74Dusty-12	120357	25-Aug-12	-2	78.566	13.18
ct94-M74Dusty-12	120357	25-Aug-12	-2	78.569	13.195
ct94-M74Dusty-12	120357	25-Aug-12	2	78.568	13.187
ct94-M74Dusty-12	120357	25-Aug-12	-2	78.568	13.205
ct94-M74Dusty-12	120357	25-Aug-12	-2	78.568	13.212
ct94-M74Dusty-12	120357	26-Aug-12	-2	78.55	13.274
ct94-M74Dusty-12	120357	26-Aug-12	-2	78.519	13.099
ct94-M74Dusty-12	120357	27-Aug-12	-2	78.324	12.822
ct94-M74Dusty-12	120357	28-Aug-12	-2	78.313	12.77
ct94-M74Dusty-12	120357	28-Aug-12	-1	78.406	13.196
ct94-M74Dusty-12	120357	28-Aug-12	1	78.24	13.936
ct94-M74Dusty-12	120357	28-Aug-12	-2	78.239	13.937
ct94-M74Dusty-12	120357	28-Aug-12	-2	78.249	13.921
ct94-M74Dusty-12	120357	28-Aug-12	-2	78.25	13.902
ct94-M74Dusty-12	120357	28-Aug-12	-2	78.252	13.911
ct94-M74Dusty-12	120357	28-Aug-12	-2	78.257	13.923
ct94-M74Dusty-12	120357	28-Aug-12	-2	78.265	13.941
ct94-M74Dusty-12	120357	28-Aug-12	-2	78.259	13.936
ct94-M74Dusty-12	120357	28-Aug-12	-1	78.269	14.163
ct94-M74Dusty-12	120357	28-Aug-12	1	78.285	14.271
ct94-M74Dusty-12	120357	28-Aug-12	2	78.286	14.345
ct94-M74Dusty-12	120357	28-Aug-12	-2	78.299	14.296
ct94-M74Dusty-12	120357	28-Aug-12	-2	78.314	14.355
ct94-M74Dusty-12	120357	29-Aug-12	-2	78.381	14.29
ct94-M74Dusty-12	120357	29-Aug-12	-2	78.349	14.314
ct94-M74Dusty-12	120357	29-Aug-12	-2	78.488	14.303
ct94-M74Dusty-12	120357	30-Aug-12	-2	78.572	14.193
ct94-M74Dusty-12	120357	30-Aug-12	-2	78.576	14.194
ct94-M74Dusty-12	120357	30-Aug-12	-2	78.56	14.271
ct94-M74Dusty-12	120357	30-Aug-12	-2	78.564	14.492
ct94-M74Dusty-12	120357	30-Aug-12	-2	78.541	14.541
ct94-M74Dusty-12	120357	30-Aug-12	-2	78.503	14.494
ct94-M74Dusty-12	120357	30-Aug-12	-2	78.506	14.477
ct94-M74Dusty-12	120357	30-Aug-12	-2	78.492	14.39
ct94-M74Dusty-12	120357	31-Aug-12	-2	78.39	15.278
ct94-M74Dusty-12	120357	31-Aug-12	-2	78.39	14.903
ct94-M74Dusty-12	120357	31-Aug-12	-2	78.369	14.885
ct94-M74Dusty-12	120357	31-Aug-12	-2	78.414	15.243
ct94-M74Dusty-12	120357	31-Aug-12	-2	78.654	14.209
ct94-M74Dusty-12	120357	31-Aug-12	-2	78.7	14.209
ct94-M74Dusty-12	120357	31-Aug-12	-2	78.707	14.188
ct94-M74Dusty-12	120357	31-Aug-12	-2	78.698	14.186
ct94-M74Dusty-12	120357	31-Aug-12	-2	78.72	14.23
ct94-M74Dusty-12	120357	31-Aug-12	-2	78.731	14.077
ct94-M74Dusty-12	120357	01-Sep-12	1	78.726	14.275
ct94-M74Dusty-12	120357	01-Sep-12	-2	78.731	14.315
ct94-M74Dusty-12	120357	01-Sep-12	1	78.735	14.306
ct94-M74Dusty-12	120357	01-Sep-12	-2	78.736	14.212
ct94-M74Dusty-12	120357	01-Sep-12	-1	78.723	14.255
ct94-M74Dusty-12	120357	01-Sep-12	2	78.718	14.306
ct94-M74Dusty-12	120357	01-Sep-12	-2	78.736	14.43
ct94-M74Dusty-12	120357	01-Sep-12	-2	78.733	14.401
ct94-M74Dusty-12	120357	01-Sep-12	-2	78.726	14.378
ct94-M74Dusty-12	120357	02-Sep-12	-2	78.764	14.846
ct94-M74Dusty-12	120357	02-Sep-12	-2	78.852	14.539
ct94-M74Dusty-12	120357	02-Sep-12	-2	78.746	14.789

ct94-M74Dusty-12	120357	02-Sep-12	-2	78.706	14.876
ct94-M74Dusty-12	120357	02-Sep-12	-2	78.721	14.871
ct94-M74Dusty-12	120357	02-Sep-12	-2	78.714	14.902
ct94-M74Dusty-12	120357	02-Sep-12	-2	78.674	14.878
ct94-M74Dusty-12	120357	02-Sep-12	-2	78.559	14.842
ct94-M74Dusty-12	120357	03-Sep-12	-2	78.55	14.82
ct94-M74Dusty-12	120357	03-Sep-12	-2	78.519	14.748
ct94-M74Dusty-12	120357	03-Sep-12	-2	78.501	14.639
ct94-M74Dusty-12	120357	03-Sep-12	-2	78.497	14.625
ct94-M74Dusty-12	120357	03-Sep-12	-2	78.471	14.548
ct94-M74Dusty-12	120357	03-Sep-12	-2	78.451	14.489
ct94-M74Dusty-12	120357	03-Sep-12	-2	78.438	14.42
ct94-M74Dusty-12	120357	03-Sep-12	-2	78.489	14.324
ct94-M74Dusty-12	120357	03-Sep-12	-2	78.433	13.793
ct94-M74Dusty-12	120357	04-Sep-12	-2	78.487	14.195
ct94-M74Dusty-12	120357	04-Sep-12	-2	78.505	14.267
ct94-M74Dusty-12	120357	04-Sep-12	-2	78.522	14.35
ct94-M74Dusty-12	120357	04-Sep-12	-2	78.51	14.287
ct94-M74Dusty-12	120357	04-Sep-12	-2	78.509	14.294
ct94-M74Dusty-12	120357	04-Sep-12	-2	78.515	14.297
ct94-M74Dusty-12	120357	05-Sep-12	-2	78.454	14.268
ct94-M74Dusty-12	120357	05-Sep-12	-2	78.46	14.265
ct94-M74Dusty-12	120357	05-Sep-12	-2	78.461	14.277
ct94-M74Dusty-12	120357	05-Sep-12	-2	78.462	14.281
ct94-M74Dusty-12	120357	05-Sep-12	-2	78.459	14.28
ct94-M74Dusty-12	120357	05-Sep-12	-2	78.459	14.291
ct94-M74Dusty-12	120357	05-Sep-12	-2	78.462	14.303
ct94-M74Dusty-12	120357	05-Sep-12	-2	78.471	14.293
ct94-M74Dusty-12	120357	05-Sep-12	-2	78.45	14.297
ct94-M74Dusty-12	120357	05-Sep-12	-2	78.467	14.303
ct94-M74Dusty-12	120357	05-Sep-12	-2	78.467	14.304
ct94-M74Dusty-12	120357	05-Sep-12	-2	78.475	14.206
ct94-M74Dusty-12	120357	05-Sep-12	-2	78.475	14.272
ct94-M74Dusty-12	120357	05-Sep-12	-2	78.521	14.142
ct94-M74Dusty-12	120357	05-Sep-12	-2	78.481	14.271
ct94-M74Dusty-12	120357	05-Sep-12	-2	78.465	14.283
ct94-M74Dusty-12	120357	05-Sep-12	-2	78.47	14.282
ct94-M74Dusty-12	120357	05-Sep-12	-2	78.463	13.935
ct94-M74Dusty-12	120357	05-Sep-12	-2	78.4	13.604
ct94-M74Dusty-12	120357	05-Sep-12	-2	78.455	14.163
ct94-M74Dusty-12	120357	05-Sep-12	-2	78.455	14.159
ct94-M74Dusty-12	120357	05-Sep-12	-2	78.482	14.216
ct94-M74Dusty-12	120357	05-Sep-12	-2	78.468	14.139
ct94-M74Dusty-12	120357	05-Sep-12	-2	78.48	14.244
ct94-M74Dusty-12	120357	06-Sep-12	-2	78.488	14.083
ct94-M74Dusty-12	120357	06-Sep-12	-2	78.476	13.997
ct94-M74Dusty-12	120357	06-Sep-12	-2	78.507	14.164
ct94-M74Dusty-12	120357	06-Sep-12	-2	78.501	14.068
ct94-M74Dusty-12	120357	06-Sep-12	-2	78.578	14.599
ct94-M74Dusty-12	120357	06-Sep-12	-1	78.595	14.595
ct94-M74Dusty-12	120357	06-Sep-12	-2	78.593	14.583
ct94-M74Dusty-12	120357	06-Sep-12	-2	78.489	14.284
ct94-M74Dusty-12	120357	06-Sep-12	-2	78.559	14.573
ct94-M74Dusty-12	120357	06-Sep-12	-2	78.538	14.476
ct94-M74Dusty-12	120357	06-Sep-12	-2	78.568	14.624
ct94-M74Dusty-12	120357	06-Sep-12	-1	78.631	14.559
ct94-M74Dusty-12	120357	06-Sep-12	-2	78.63	14.572
ct94-M74Dusty-12	120357	06-Sep-12	-2	78.618	14.615
ct94-M74Dusty-12	120357	06-Sep-12	-2	78.619	14.632
ct94-M74Dusty-12	120357	06-Sep-12	-1	78.61	14.738
ct94-M74Dusty-12	120357	06-Sep-12	-1	78.665	14.638
ct94-M74Dusty-12	120357	06-Sep-12	-2	78.648	14.783
ct94-M74Dusty-12	120357	06-Sep-12	-2	78.655	14.757
ct94-M74Dusty-12	120357	06-Sep-12	-1	78.666	14.558
ct94-M74Dusty-12	120357	06-Sep-12	-2	78.677	14.49
ct94-M74Dusty-12	120357	06-Sep-12	-1	78.672	14.466
ct94-M74Dusty-12	120357	06-Sep-12	-2	78.69	14.451
ct94-M74Dusty-12	120357	06-Sep-12	-1	78.69	14.449

ct94-M74Dusty-12	120357	06-Sep-12	-1	78.689	14.425
ct94-M74Dusty-12	120357	06-Sep-12	-2	78.702	14.421
ct94-M74Dusty-12	120357	06-Sep-12	0	78.74	14.379
ct94-M74Dusty-12	120357	06-Sep-12	-2	78.749	14.424
ct94-M74Dusty-12	120357	06-Sep-12	1	78.73	14.302
ct94-M74Dusty-12	120357	06-Sep-12	1	78.723	14.318
ct94-M74Dusty-12	120357	06-Sep-12	2	78.717	14.316
ct94-M74Dusty-12	120357	06-Sep-12	0	78.722	14.253
ct94-M74Dusty-12	120357	06-Sep-12	3	78.719	14.308
ct94-M74Dusty-12	120357	06-Sep-12	-2	78.719	14.309
ct94-M74Dusty-12	120357	06-Sep-12	1	78.715	14.336
ct94-M74Dusty-12	120357	06-Sep-12	-2	78.761	14.187
ct94-M74Dusty-12	120357	06-Sep-12	-2	78.764	14.213
ct94-M74Dusty-12	120357	07-Sep-12	-2	78.752	14.245
ct94-M74Dusty-12	120357	07-Sep-12	-2	78.714	14.349
ct94-M74Dusty-12	120357	07-Sep-12	-2	78.712	14.335
ct94-M74Dusty-12	120357	07-Sep-12	-2	78.722	14.309
ct94-M74Dusty-12	120357	07-Sep-12	-2	78.723	14.292
ct94-M74Dusty-12	120357	07-Sep-12	-2	78.694	14.427
ct94-M74Dusty-12	120357	08-Sep-12	-2	78.69	14.203
ct94-M74Dusty-12	120357	08-Sep-12	-9	78.484	14.262
ct94-M74Dusty-12	120357	08-Sep-12	-2	78.567	14.242
ct94-M74Dusty-12	120357	08-Sep-12	-2	78.499	14.276
ct94-M74Dusty-12	120357	08-Sep-12	-2	78.474	14.266
ct94-M74Dusty-12	120357	08-Sep-12	-2	78.474	14.269
ct94-M74Dusty-12	120357	08-Sep-12	-2	78.475	14.267
ct94-M74Dusty-12	120357	08-Sep-12	-2	78.453	14.259
ct94-M74Dusty-12	120357	08-Sep-12	-2	78.465	14.266
ct94-M74Dusty-12	120357	08-Sep-12	-2	78.467	14.326
ct94-M74Dusty-12	120357	08-Sep-12	-2	78.467	14.309
ct94-M74Dusty-12	120357	08-Sep-12	-2	78.477	14.308
ct94-M74Dusty-12	120357	08-Sep-12	-2	78.478	14.304
ct94-M74Dusty-12	120357	08-Sep-12	-2	78.473	14.304
ct94-M74Dusty-12	120357	08-Sep-12	-2	78.398	14.207
ct94-M74Dusty-12	120357	08-Sep-12	-2	78.469	14.259
ct94-M74Dusty-12	120357	08-Sep-12	-2	78.48	14.264
ct94-M74Dusty-12	120357	08-Sep-12	-2	78.448	14.154
ct94-M74Dusty-12	120357	08-Sep-12	-2	78.437	14.192
ct94-M74Dusty-12	120357	08-Sep-12	-2	78.454	14.173
ct94-M74Dusty-12	120357	08-Sep-12	-2	78.458	14.168
ct94-M74Dusty-12	120357	09-Sep-12	-2	78.491	14.257
ct94-M74Dusty-12	120357	09-Sep-12	-2	78.496	14.278
ct94-M74Dusty-12	120357	09-Sep-12	-2	78.488	14.299
ct94-M74Dusty-12	120357	09-Sep-12	-2	78.486	14.294
ct94-M74Dusty-12	120357	09-Sep-12	-2	78.434	14.288
ct94-M74Dusty-12	120357	09-Sep-12	-2	78.46	14.29
ct94-M74Dusty-12	120357	10-Sep-12	-2	78.443	14.251
ct94-M74Dusty-12	120357	11-Sep-12	-2	78.5	14.373
ct94-M74Dusty-12	120357	11-Sep-12	-2	78.492	14.36
ct94-M74Dusty-12	120357	12-Sep-12	-2	78.496	14.345
ct94-M74Dusty-12	120357	12-Sep-12	-2	78.359	14.519
ct94-M74Dusty-12	120357	12-Sep-12	-2	78.449	14.411
ct94-M74Dusty-12	120357	12-Sep-12	-2	78.444	14.422
ct94-M74Dusty-12	120357	12-Sep-12	-2	78.445	14.431
ct94-M74Dusty-12	120357	12-Sep-12	-2	78.44	14.421
ct94-M74Dusty-12	120357	13-Sep-12	-2	78.444	14.416
ct94-M74Dusty-12	120357	13-Sep-12	-2	78.414	14.429
ct94-M74Dusty-12	120357	13-Sep-12	-2	78.413	14.432
ct94-M74Dusty-12	120357	13-Sep-12	-2	78.427	14.469
ct94-M74Dusty-12	120357	13-Sep-12	-2	78.41	14.541
ct94-M74Dusty-12	120357	13-Sep-12	1	78.363	14.614
ct94-M74Dusty-12	120357	13-Sep-12	-2	78.356	14.619
ct94-M74Dusty-12	120357	13-Sep-12	-2	78.357	14.409
ct94-M74Dusty-12	120357	13-Sep-12	-2	78.352	14.601
ct94-M74Dusty-12	120357	13-Sep-12	-2	78.33	14.565
ct94-M74Dusty-12	120357	13-Sep-12	-2	78.371	14.597
ct94-M74Dusty-12	120357	13-Sep-12	-2	78.366	14.581
ct94-M74Dusty-12	120357	14-Sep-12	-2	78.366	14.251

ct94-M74Dusty-12	120357	14-Sep-12	-2	78.388	14.143
ct94-M74Dusty-12	120357	14-Sep-12	-1	78.373	14.614
ct94-M74Dusty-12	120357	14-Sep-12	-2	78.37	14.666
ct94-M74Dusty-12	120357	14-Sep-12	-2	78.365	14.768
ct94-M74Dusty-12	120357	14-Sep-12	-2	78.381	14.866
ct94-M74Dusty-12	120357	16-Sep-12	2	78.555	14.62
ct94-M74Dusty-12	120357	16-Sep-12	-2	78.555	14.62
ct94-M74Dusty-12	120357	17-Sep-12	-2	78.539	16.976
ct94-M74Dusty-12	120357	17-Sep-12	-2	78.531	14.418
ct94-M74Dusty-12	120357	17-Sep-12	0	78.521	14.221
ct94-M74Dusty-12	120357	17-Sep-12	-2	78.521	14.221
ct94-M74Dusty-12	120357	18-Sep-12	-2	78.537	14.454
ct94-M74Dusty-12	120357	18-Sep-12	-2	78.543	14.231
ct94-M74Dusty-12	120357	18-Sep-12	-2	78.549	14.273
ct94-M74Dusty-12	120357	18-Sep-12	-1	78.479	14.256
ct94-M74Dusty-12	120357	18-Sep-12	-1	78.49	14.2
ct94-M74Dusty-12	120357	18-Sep-12	-1	78.479	14.256
ct94-M74Dusty-12	120357	18-Sep-12	-2	78.475	14.255
ct94-M74Dusty-12	120357	20-Sep-12	-2	78.46	14.293
ct94-M74Dusty-12	120357	20-Sep-12	-2	78.466	14.248
ct94-M74Dusty-12	120357	20-Sep-12	-2	78.469	14.248
ct94-M74Dusty-12	120357	20-Sep-12	-2	78.453	14.255
ct94-M74Dusty-12	120357	20-Sep-12	-2	78.462	14.255
ct94-M74Dusty-12	120357	20-Sep-12	-2	78.462	14.258
ct94-M74Dusty-12	120357	20-Sep-12	-2	78.353	14.32
ct94-M74Dusty-12	120357	20-Sep-12	-1	78.507	14.054
ct94-M74Dusty-12	120357	20-Sep-12	-2	78.413	14.221
ct94-M74Dusty-12	120357	20-Sep-12	-2	78.448	14.222
ct94-M74Dusty-12	120357	20-Sep-12	-2	78.477	14.291
ct94-M74Dusty-12	120357	20-Sep-12	-2	78.47	14.297
ct94-M74Dusty-12	120357	20-Sep-12	-2	78.489	14.22
ct94-M74Dusty-12	120357	20-Sep-12	-2	78.487	14.217
ct94-M74Dusty-12	120357	20-Sep-12	-2	78.484	14.267
ct94-M74Dusty-12	120357	20-Sep-12	-2	78.485	14.284
ct94-M74Dusty-12	120357	20-Sep-12	-1	78.465	14.098
ct94-M74Dusty-12	120357	20-Sep-12	-2	78.486	14.318
ct94-M74Dusty-12	120357	20-Sep-12	-2	78.486	14.334
ct94-M74Dusty-12	120357	20-Sep-12	-2	78.485	14.495
ct94-M74Dusty-12	120357	20-Sep-12	-2	78.484	14.477
ct94-M74Dusty-12	120357	20-Sep-12	-2	78.477	14.526
ct94-M74Dusty-12	120357	20-Sep-12	-2	78.518	14.498
ct94-M74Dusty-12	120357	20-Sep-12	-2	78.515	14.458
ct94-M74Dusty-12	120357	20-Sep-12	-2	78.491	14.569
ct94-M74Dusty-12	120357	20-Sep-12	-2	78.466	14.647
ct94-M74Dusty-12	120357	20-Sep-12	-2	78.509	14.512
ct94-M74Dusty-12	120357	20-Sep-12	-2	78.494	14.777
ct94-M74Dusty-12	120357	20-Sep-12	-2	78.499	14.602
ct94-M74Dusty-12	120357	20-Sep-12	-2	78.495	14.83
ct94-M74Dusty-12	120357	20-Sep-12	-2	78.504	14.651
ct94-M74Dusty-12	120357	20-Sep-12	-2	78.507	14.581
ct94-M74Dusty-12	120357	20-Sep-12	-2	78.509	14.599
ct94-M74Dusty-12	120357	20-Sep-12	-2	78.512	14.607
ct94-M74Dusty-12	120357	20-Sep-12	-2	78.514	14.588
ct94-M74Dusty-12	120357	20-Sep-12	-2	78.518	14.505
ct94-M74Dusty-12	120357	20-Sep-12	-2	78.493	14.537
ct94-M74Dusty-12	120357	20-Sep-12	2	78.484	14.336
ct94-M74Dusty-12	120357	20-Sep-12	2	78.484	14.339
ct94-M74Dusty-12	120357	20-Sep-12	-2	78.484	14.336
ct94-M74Dusty-12	120357	20-Sep-12	-2	78.484	14.339
ct94-M74Dusty-12	120357	21-Sep-12	2	78.48	14.306
ct94-M74Dusty-12	120357	21-Sep-12	-2	78.48	14.305
ct94-M74Dusty-12	120357	21-Sep-12	3	78.479	14.315
ct94-M74Dusty-12	120357	21-Sep-12	3	78.479	14.319
ct94-M74Dusty-12	120357	21-Sep-12	3	78.479	14.316
ct94-M74Dusty-12	120357	21-Sep-12	1	78.475	14.336
ct94-M74Dusty-12	120357	21-Sep-12	-2	78.468	14.336
ct94-M74Dusty-12	120357	21-Sep-12	-1	78.488	14.418
ct94-M74Dusty-12	120357	21-Sep-12	-1	78.501	14.308

ct94-M74Dusty-12	120357	21-Sep-12	-2	78.484	14.345
ct94-M74Dusty-12	120357	21-Sep-12	-2	78.486	14.355
ct94-M74Dusty-12	120357	21-Sep-12	-1	78.478	14.394
ct94-M74Dusty-12	120357	21-Sep-12	-2	78.473	14.405
ct94-M74Dusty-12	120357	21-Sep-12	-2	78.487	14.422
ct94-M74Dusty-12	120357	21-Sep-12	-2	78.49	14.374
ct94-M74Dusty-12	120357	21-Sep-12	-2	78.464	14.351
ct94-M74Dusty-12	120357	21-Sep-12	-2	78.47	14.314
ct94-M74Dusty-12	120357	21-Sep-12	-2	78.469	14.358
ct94-M74Dusty-12	120357	21-Sep-12	-2	78.468	14.391
ct94-M74Dusty-12	120357	21-Sep-12	-2	78.494	14.331
ct94-M74Dusty-12	120357	21-Sep-12	-2	78.497	14.302
ct94-M74Dusty-12	120357	21-Sep-12	-2	78.495	14.286
ct94-M74Dusty-12	120357	21-Sep-12	-2	78.502	14.295
ct94-M74Dusty-12	120357	21-Sep-12	-2	78.495	14.276
ct94-M74Dusty-12	120357	21-Sep-12	-1	78.48	14.351
ct94-M74Dusty-12	120357	21-Sep-12	-2	78.496	14.29
ct94-M74Dusty-12	120357	21-Sep-12	-2	78.501	14.314
ct94-M74Dusty-12	120357	21-Sep-12	-2	78.498	14.277
ct94-M74Dusty-12	120357	21-Sep-12	-2	78.497	14.289
ct94-M74Dusty-12	120357	21-Sep-12	-2	78.48	14.295
ct94-M74Dusty-12	120357	21-Sep-12	-2	78.479	14.303
ct94-M74Dusty-12	120357	21-Sep-12	-2	78.477	14.3
ct94-M74Dusty-12	120357	21-Sep-12	-2	78.478	14.289
ct94-M74Dusty-12	120357	21-Sep-12	-2	78.481	14.277
ct94-M74Dusty-12	120357	21-Sep-12	-2	78.481	14.068
ct94-M74Dusty-12	120357	21-Sep-12	3	78.487	14.26
ct94-M74Dusty-12	120357	21-Sep-12	1	78.491	14.304
ct94-M74Dusty-12	120357	21-Sep-12	2	78.482	14.212
ct94-M74Dusty-12	120357	21-Sep-12	-1	78.476	14.268
ct94-M74Dusty-12	120357	21-Sep-12	-2	78.478	14.266
ct94-M74Dusty-12	120357	21-Sep-12	2	78.481	14.272
ct94-M74Dusty-12	120357	22-Sep-12	3	78.48	14.291
ct94-M74Dusty-12	120357	22-Sep-12	-2	78.485	14.281
ct94-M74Dusty-12	120357	22-Sep-12	-2	78.485	14.281
ct94-M74Dusty-12	120357	22-Sep-12	-1	78.483	14.291
ct94-M74Dusty-12	120357	22-Sep-12	-2	78.471	14.467
ct94-M74Dusty-12	120357	22-Sep-12	-2	78.463	14.411
ct94-M74Dusty-12	120357	22-Sep-12	-2	78.455	14.339
ct94-M74Dusty-12	120357	22-Sep-12	-2	78.472	14.26
ct94-M74Dusty-12	120357	22-Sep-12	-2	78.464	14.283
ct94-M74Dusty-12	120357	22-Sep-12	-1	78.499	14.39
ct94-M74Dusty-12	120357	22-Sep-12	-2	78.494	14.368
ct94-M74Dusty-12	120357	22-Sep-12	-2	78.476	14.264
ct94-M74Dusty-12	120357	22-Sep-12	-2	78.472	14.255
ct94-M74Dusty-12	120357	22-Sep-12	-2	78.469	14.252
ct94-M74Dusty-12	120357	22-Sep-12	-2	78.472	14.251
ct94-M74Dusty-12	120357	22-Sep-12	0	78.485	14.32
ct94-M74Dusty-12	120357	22-Sep-12	-2	78.448	14.117
ct94-M74Dusty-12	120357	22-Sep-12	-2	78.461	14.108
ct94-M74Dusty-12	120357	22-Sep-12	-2	78.477	14.141
ct94-M74Dusty-12	120357	22-Sep-12	-2	78.45	14.288
ct94-M74Dusty-12	120357	22-Sep-12	-2	78.457	14.273
ct94-M74Dusty-12	120357	22-Sep-12	-2	78.493	14.298
ct94-M74Dusty-12	120357	22-Sep-12	-2	78.488	14.28
ct94-M74Dusty-12	120357	22-Sep-12	-2	78.492	14.307
ct94-M74Dusty-12	120357	22-Sep-12	-2	78.492	14.296
ct94-M74Dusty-12	120357	22-Sep-12	-2	78.496	14.194
ct94-M74Dusty-12	120357	22-Sep-12	-2	78.457	14.37
ct94-M74Dusty-12	120357	22-Sep-12	-2	78.465	14.355
ct94-M74Dusty-12	120357	22-Sep-12	-2	78.467	14.348
ct94-M74Dusty-12	120357	22-Sep-12	-2	78.467	14.336
ct94-M74Dusty-12	120357	22-Sep-12	-2	78.467	14.316
ct94-M74Dusty-12	120357	22-Sep-12	-2	78.474	14.3
ct94-M74Dusty-12	120357	22-Sep-12	-2	78.479	14.289
ct94-M74Dusty-12	120357	22-Sep-12	-2	78.477	14.284
ct94-M74Dusty-12	120357	22-Sep-12	-2	78.476	14.273
ct94-M74Dusty-12	120357	22-Sep-12	-2	78.484	14.264

ct94-M74Dusty-12	120357	22-Sep-12	0	78.486	14.294
ct94-M74Dusty-12	120357	22-Sep-12	-2	78.491	14.299
ct94-M74Dusty-12	120357	22-Sep-12	-2	78.491	14.301
ct94-M74Dusty-12	120357	22-Sep-12	3	78.491	14.297
ct94-M74Dusty-12	120357	22-Sep-12	2	78.484	14.276
ct94-M74Dusty-12	120357	22-Sep-12	3	78.487	14.275
ct94-M74Dusty-12	120357	22-Sep-12	1	78.481	14.277
ct94-M74Dusty-12	120357	22-Sep-12	-2	78.481	14.278
ct94-M74Dusty-12	120357	22-Sep-12	0	78.487	14.272
ct94-M74Dusty-12	120357	23-Sep-12	-2	78.494	14.277
ct94-M74Dusty-12	120357	23-Sep-12	-2	78.494	14.291
ct94-M74Dusty-12	120357	23-Sep-12	-2	78.48	14.443
ct94-M74Dusty-12	120357	23-Sep-12	-2	78.497	14.252
ct94-M74Dusty-12	120357	23-Sep-12	-2	78.492	14.265
ct94-M74Dusty-12	120357	23-Sep-12	-2	78.49	14.252
ct94-M74Dusty-12	120357	23-Sep-12	-2	78.488	14.257
ct94-M74Dusty-12	120357	23-Sep-12	-2	78.489	14.247
ct94-M74Dusty-12	120357	23-Sep-12	-2	78.494	14.221
ct94-M74Dusty-12	120357	23-Sep-12	-2	78.492	14.253
ct94-M74Dusty-12	120357	23-Sep-12	-2	78.489	14.26
ct94-M74Dusty-12	120357	23-Sep-12	-2	78.49	14.259
ct94-M74Dusty-12	120357	23-Sep-12	-2	78.491	14.257
ct94-M74Dusty-12	120357	23-Sep-12	-2	78.49	14.26
ct94-M74Dusty-12	120357	23-Sep-12	-2	78.493	14.251
ct94-M74Dusty-12	120357	23-Sep-12	-2	78.491	14.275
ct94-M74Dusty-12	120357	23-Sep-12	-2	78.482	14.28
ct94-M74Dusty-12	120357	23-Sep-12	-2	78.496	14.212
ct94-M74Dusty-12	120357	23-Sep-12	-2	78.53	14.166
ct94-M74Dusty-12	120357	23-Sep-12	-1	78.532	14.158
ct94-M74Dusty-12	120357	23-Sep-12	-1	78.532	14.155
ct94-M74Dusty-12	120357	23-Sep-12	-1	78.491	14.289
ct94-M74Dusty-12	120357	23-Sep-12	-2	78.511	14.258
ct94-M74Dusty-12	120357	23-Sep-12	-2	78.511	14.271
ct94-M74Dusty-12	120357	23-Sep-12	-2	78.516	14.243
ct94-M74Dusty-12	120357	23-Sep-12	-2	78.512	14.262
ct94-M74Dusty-12	120357	23-Sep-12	-2	78.513	14.282
ct94-M74Dusty-12	120357	23-Sep-12	0	78.521	14.392
ct94-M74Dusty-12	120357	23-Sep-12	-2	78.544	14.45
ct94-M74Dusty-12	120357	23-Sep-12	-2	78.505	14.377
ct94-M74Dusty-12	120357	23-Sep-12	-2	78.514	14.306
ct94-M74Dusty-12	120357	23-Sep-12	-1	78.493	14.287
ct94-M74Dusty-12	120357	23-Sep-12	-2	78.493	14.287
ct94-M74Dusty-12	120357	23-Sep-12	-2	78.495	14.284
ct94-M74Dusty-12	120357	23-Sep-12	-2	78.496	14.281
ct94-M74Dusty-12	120357	23-Sep-12	-2	78.502	14.257
ct94-M74Dusty-12	120357	23-Sep-12	-2	78.501	14.283
ct94-M74Dusty-12	120357	23-Sep-12	-2	78.5	14.271
ct94-M74Dusty-12	120357	23-Sep-12	-2	78.498	14.271
ct94-M74Dusty-12	120357	23-Sep-12	-2	78.505	14.254
ct94-M74Dusty-12	120357	23-Sep-12	-2	78.49	14.257
ct94-M74Dusty-12	120357	23-Sep-12	-2	78.497	14.247
ct94-M74Dusty-12	120357	23-Sep-12	-2	78.504	14.248
ct94-M74Dusty-12	120357	23-Sep-12	-2	78.501	14.252
ct94-M74Dusty-12	120357	23-Sep-12	-2	78.504	14.297
ct94-M74Dusty-12	120357	23-Sep-12	-2	78.512	14.294
ct94-M74Dusty-12	120357	23-Sep-12	-2	78.505	14.283
ct94-M74Dusty-12	120357	23-Sep-12	-2	78.512	14.228
ct94-M74Dusty-12	120357	23-Sep-12	-2	78.504	14.219
ct94-M74Dusty-12	120357	23-Sep-12	-2	78.504	14.273
ct94-M74Dusty-12	120357	23-Sep-12	-2	78.476	14.438
ct94-M74Dusty-12	120357	23-Sep-12	-2	78.478	14.445
ct94-M74Dusty-12	120357	23-Sep-12	-2	78.478	14.445
ct94-M74Dusty-12	120357	24-Sep-12	-2	78.481	14.442
ct94-M74Dusty-12	120357	24-Sep-12	-2	78.47	14.263
ct94-M74Dusty-12	120357	24-Sep-12	-2	78.483	14.429
ct94-M74Dusty-12	120357	24-Sep-12	-2	78.481	14.409
ct94-M74Dusty-12	120357	24-Sep-12	-2	78.482	14.396
ct94-M74Dusty-12	120357	24-Sep-12	-2	78.48	14.404

ct94-M74Dusty-12	120357	24-Sep-12	-2	78.489	14.345
ct94-M74Dusty-12	120357	24-Sep-12	-2	78.484	14.346
ct94-M74Dusty-12	120357	24-Sep-12	-2	78.479	14.288
ct94-M74Dusty-12	120357	24-Sep-12	-2	78.488	14.337
ct94-M74Dusty-12	120357	24-Sep-12	-2	78.478	14.29
ct94-M74Dusty-12	120357	24-Sep-12	-2	78.477	14.271
ct94-M74Dusty-12	120357	24-Sep-12	-2	78.476	14.276
ct94-M74Dusty-12	120357	24-Sep-12	-2	78.484	14.264
ct94-M74Dusty-12	120357	24-Sep-12	-2	78.477	14.324
ct94-M74Dusty-12	120357	24-Sep-12	-2	78.459	14.206
ct94-M74Dusty-12	120357	24-Sep-12	-2	78.466	14.225
ct94-M74Dusty-12	120357	24-Sep-12	-2	78.467	14.195
ct94-M74Dusty-12	120357	24-Sep-12	-2	78.467	14.196
ct94-M74Dusty-12	120357	24-Sep-12	-2	78.464	14.135
ct94-M74Dusty-12	120357	24-Sep-12	-2	78.475	14.148
ct94-M74Dusty-12	120357	25-Sep-12	-2	78.485	14.191
ct94-M74Dusty-12	120357	25-Sep-12	-2	78.486	14.146
ct94-M74Dusty-12	120357	25-Sep-12	-2	78.474	14.17
ct94-M74Dusty-12	120357	25-Sep-12	-2	78.459	14.073
ct94-M74Dusty-12	120357	25-Sep-12	-2	78.453	14.127
ct94-M74Dusty-12	120357	25-Sep-12	-2	78.491	14.116
ct94-M74Dusty-12	120357	25-Sep-12	-2	78.494	14.293
ct94-M74Dusty-12	120357	25-Sep-12	-2	78.492	14.286
ct94-M74Dusty-12	120357	25-Sep-12	-2	78.499	14.326
ct94-M74Dusty-12	120357	25-Sep-12	-2	78.485	14.323
ct94-M74Dusty-12	120357	25-Sep-12	-2	78.479	14.317
ct94-M74Dusty-12	120357	25-Sep-12	-2	78.492	14.454
ct94-M74Dusty-12	120357	25-Sep-12	-2	78.497	14.422
ct94-M74Dusty-12	120357	25-Sep-12	-2	78.484	14.4
ct94-M74Dusty-12	120357	25-Sep-12	-2	78.483	14.391
ct94-M74Dusty-12	120357	25-Sep-12	-2	78.483	14.33
ct94-M74Dusty-12	120357	25-Sep-12	-2	78.475	14.343
ct94-M74Dusty-12	120357	25-Sep-12	-2	78.472	14.326
ct94-M74Dusty-12	120357	25-Sep-12	-2	78.468	14.312
ct94-M74Dusty-12	120357	25-Sep-12	-2	78.473	14.302
ct94-M74Dusty-12	120357	25-Sep-12	-2	78.478	14.294
ct94-M74Dusty-12	120357	25-Sep-12	-2	78.48	14.289
ct94-M74Dusty-12	120357	25-Sep-12	-2	78.476	14.264
ct94-M74Dusty-12	120357	25-Sep-12	-2	78.479	14.277
ct94-M74Dusty-12	120357	25-Sep-12	-2	78.48	14.279
ct94-M74Dusty-12	120357	26-Sep-12	-2	78.463	14.255
ct94-M74Dusty-12	120357	26-Sep-12	-2	78.468	14.276
ct94-M74Dusty-12	120357	26-Sep-12	-2	78.464	14.236
ct94-M74Dusty-12	120357	26-Sep-12	-2	78.466	14.263
ct94-M74Dusty-12	120357	26-Sep-12	-2	78.468	14.253
ct94-M74Dusty-12	120357	26-Sep-12	-2	78.476	14.289
ct94-M74Dusty-12	120357	26-Sep-12	-2	78.487	14.323
ct94-M74Dusty-12	120357	26-Sep-12	-2	78.492	14.341
ct94-M74Dusty-12	120357	26-Sep-12	-2	78.49	14.344
ct94-M74Dusty-12	120357	26-Sep-12	-2	78.493	14.349
ct94-M74Dusty-12	120357	26-Sep-12	-2	78.489	14.33
ct94-M74Dusty-12	120357	26-Sep-12	-2	78.48	14.275
ct94-M74Dusty-12	120357	26-Sep-12	-2	78.478	14.295
ct94-M74Dusty-12	120357	26-Sep-12	-2	78.475	14.3
ct94-M74Dusty-12	120357	26-Sep-12	-2	78.476	14.302
ct94-M74Dusty-12	120357	26-Sep-12	-2	78.482	14.214
ct94-M74Dusty-12	120357	26-Sep-12	-2	78.491	14.18
ct94-M74Dusty-12	120357	26-Sep-12	-2	78.493	14.167
ct94-M74Dusty-12	120357	26-Sep-12	-1	78.49	14.284
ct94-M74Dusty-12	120357	26-Sep-12	-1	78.495	14.259
ct94-M74Dusty-12	120357	27-Sep-12	-1	78.478	14.313
ct94-M74Dusty-12	120357	27-Sep-12	-2	78.5	14.358
ct94-M74Dusty-12	120357	27-Sep-12	-2	78.479	14.321
ct94-M74Dusty-12	120357	27-Sep-12	-2	78.479	14.336
ct94-M74Dusty-12	120357	27-Sep-12	-2	78.481	14.365
ct94-M74Dusty-12	120357	27-Sep-12	-2	78.484	14.382
ct94-M74Dusty-12	120357	27-Sep-12	-2	78.494	14.37
ct94-M74Dusty-12	120357	27-Sep-12	-2	78.49	14.347

ct94-M74Dusty-12	120357	27-Sep-12	-2	78.485	14.331
ct94-M74Dusty-12	120357	27-Sep-12	-2	78.485	14.303
ct94-M74Dusty-12	120357	27-Sep-12	-2	78.486	14.299
ct94-M74Dusty-12	120357	27-Sep-12	-2	78.496	14.261
ct94-M74Dusty-12	120357	27-Sep-12	-2	78.488	14.275
ct94-M74Dusty-12	120357	27-Sep-12	-2	78.489	14.269
ct94-M74Dusty-12	120357	27-Sep-12	-2	78.487	14.269
ct94-M74Dusty-12	120357	27-Sep-12	-2	78.484	14.262
ct94-M74Dusty-12	120357	27-Sep-12	-2	78.481	14.274
ct94-M74Dusty-12	120357	27-Sep-12	-2	78.48	14.256
ct94-M74Dusty-12	120357	27-Sep-12	-2	78.481	14.236
ct94-M74Dusty-12	120357	27-Sep-12	-2	78.481	14.231
ct94-M74Dusty-12	120357	27-Sep-12	-2	78.481	14.231
ct94-M74Dusty-12	120357	27-Sep-12	-2	78.481	14.233
ct94-M74Dusty-12	120357	27-Sep-12	-2	78.487	14.252
ct94-M74Dusty-12	120357	28-Sep-12	-2	78.487	14.25
ct94-M74Dusty-12	120357	28-Sep-12	-1	78.467	14.136
ct94-M74Dusty-12	120357	28-Sep-12	-2	78.467	14.249
ct94-M74Dusty-12	120357	28-Sep-12	-2	78.464	14.42
ct94-M74Dusty-12	120357	28-Sep-12	1	78.481	14.417
ct94-M74Dusty-12	120357	28-Sep-12	3	78.474	14.391
ct94-M74Dusty-12	120357	28-Sep-12	-2	78.474	14.391
ct94-M74Dusty-12	120357	28-Sep-12	-2	78.502	14.557
ct94-M74Dusty-12	120357	28-Sep-12	-2	78.504	14.491
ct94-M74Dusty-12	120357	28-Sep-12	-2	78.505	14.524
ct94-M74Dusty-12	120357	28-Sep-12	-2	78.507	14.501
ct94-M74Dusty-12	120357	28-Sep-12	-2	78.51	14.526
ct94-M74Dusty-12	120357	28-Sep-12	-2	78.508	14.511
ct94-M74Dusty-12	120357	28-Sep-12	-1	78.517	13.986
ct94-M74Dusty-12	120357	28-Sep-12	-1	78.505	14.427
ct94-M74Dusty-12	120357	28-Sep-12	-2	78.504	14.42
ct94-M74Dusty-12	120357	28-Sep-12	-2	78.516	14.456
ct94-M74Dusty-12	120357	28-Sep-12	-1	78.506	14.404
ct94-M74Dusty-12	120357	28-Sep-12	-2	78.505	14.379
ct94-M74Dusty-12	120357	28-Sep-12	-2	78.508	14.29
ct94-M74Dusty-12	120357	28-Sep-12	-2	78.491	14.238
ct94-M74Dusty-12	120357	28-Sep-12	-2	78.49	14.237
ct94-M74Dusty-12	120357	28-Sep-12	-2	78.492	14.286
ct94-M74Dusty-12	120357	28-Sep-12	-2	78.497	14.289
ct94-M74Dusty-12	120357	28-Sep-12	-2	78.497	14.338
ct94-M74Dusty-12	120357	28-Sep-12	-2	78.488	14.383
ct94-M74Dusty-12	120357	28-Sep-12	-2	78.487	14.378
ct94-M74Dusty-12	120357	28-Sep-12	-2	78.485	14.362
ct94-M74Dusty-12	120357	28-Sep-12	-2	78.492	14.342
ct94-M74Dusty-12	120357	28-Sep-12	-2	78.494	14.283
ct94-M74Dusty-12	120357	28-Sep-12	-2	78.493	14.255
ct94-M74Dusty-12	120357	28-Sep-12	-2	78.492	14.232
ct94-M74Dusty-12	120357	28-Sep-12	-2	78.491	14.234
ct94-M74Dusty-12	120357	28-Sep-12	-2	78.491	14.231
ct94-M74Dusty-12	120357	28-Sep-12	-2	78.491	14.232
ct94-M74Dusty-12	120357	28-Sep-12	-2	78.484	14.239
ct94-M74Dusty-12	120357	28-Sep-12	-2	78.486	14.223
ct94-M74Dusty-12	120357	28-Sep-12	-2	78.478	14.23
ct94-M74Dusty-12	120357	28-Sep-12	-2	78.473	14.194
ct94-M74Dusty-12	120357	29-Sep-12	-2	78.471	14.181
ct94-M74Dusty-12	120357	29-Sep-12	-2	78.619	14.108
ct94-M74Dusty-12	120357	29-Sep-12	-2	78.624	14.123
ct94-M74Dusty-12	120357	29-Sep-12	-2	78.506	14.59
ct94-M74Dusty-12	120357	29-Sep-12	-2	78.506	14.594
ct94-M74Dusty-12	120357	29-Sep-12	-2	78.504	14.576
ct94-M74Dusty-12	120357	29-Sep-12	-2	78.645	14.527
ct94-M74Dusty-12	120357	29-Sep-12	-2	78.485	14.317
ct94-M74Dusty-12	120357	29-Sep-12	-2	78.494	14.247
ct94-M74Dusty-12	120357	29-Sep-12	-2	78.488	14.293
ct94-M74Dusty-12	120357	29-Sep-12	-2	78.489	14.294
ct94-M74Dusty-12	120357	29-Sep-12	-2	78.482	14.326
ct94-M74Dusty-12	120357	29-Sep-12	-2	78.468	14.321
ct94-M74Dusty-12	120357	29-Sep-12	-2	78.474	14.326

ct94-M74Dusty-12	120357	29-Sep-12	-2	78.465	14.318
ct94-M74Dusty-12	120357	29-Sep-12	-2	78.364	14.245
ct94-M74Dusty-12	120357	29-Sep-12	-2	78.356	14.38
ct94-M74Dusty-12	120357	29-Sep-12	-2	78.416	14.305
ct94-M74Dusty-12	120357	30-Sep-12	-2	78.198	14.386
ct94-M74Dusty-12	120357	30-Sep-12	-2	78.406	14.278
ct94-M74Dusty-12	120357	30-Sep-12	-2	78.405	14.264
ct94-M74Dusty-12	120357	30-Sep-12	-2	78.406	14.28
ct94-M74Dusty-12	120357	30-Sep-12	-2	78.409	14.249
ct94-M74Dusty-12	120357	30-Sep-12	-2	78.41	14.286
ct94-M74Dusty-12	120357	30-Sep-12	-2	78.412	14.294
ct94-M74Dusty-12	120357	30-Sep-12	-2	78.453	14.365
ct94-M74Dusty-12	120357	30-Sep-12	-2	78.467	14.379
ct94-M74Dusty-12	120357	30-Sep-12	-2	78.474	14.323
ct94-M74Dusty-12	120357	30-Sep-12	-2	78.478	14.352
ct94-M74Dusty-12	120357	30-Sep-12	-2	78.486	14.3
ct94-M74Dusty-12	120357	30-Sep-12	-2	78.482	14.209
ct94-M74Dusty-12	120357	30-Sep-12	-2	78.483	14.261
ct94-M74Dusty-12	120357	30-Sep-12	-2	78.467	14.402
ct94-M74Dusty-12	120357	01-Oct-12	-2	78.473	14.333
ct94-M74Dusty-12	120357	01-Oct-12	-2	78.478	14.328
ct94-M74Dusty-12	120357	01-Oct-12	-2	78.482	14.302
ct94-M74Dusty-12	120357	01-Oct-12	-2	78.481	14.303
ct94-M74Dusty-12	120357	01-Oct-12	-2	78.493	14.285
ct94-M74Dusty-12	120357	01-Oct-12	-2	78.496	14.249
ct94-M74Dusty-12	120357	01-Oct-12	-2	78.483	14.287
ct94-M74Dusty-12	120357	01-Oct-12	-2	78.483	14.293
ct94-M74Dusty-12	120357	01-Oct-12	-2	78.5	14.24
ct94-M74Dusty-12	120357	01-Oct-12	-2	78.493	14.245
ct94-M74Dusty-12	120357	01-Oct-12	-2	78.491	14.252
ct94-M74Dusty-12	120357	01-Oct-12	-2	78.49	14.247
ct94-M74Dusty-12	120357	01-Oct-12	-2	78.483	14.231
ct94-M74Dusty-12	120357	01-Oct-12	-2	78.475	14.235
ct94-M74Dusty-12	120357	01-Oct-12	-2	78.474	14.232
ct94-M74Dusty-12	120357	01-Oct-12	-2	78.474	14.231
ct94-M74Dusty-12	120357	01-Oct-12	-2	78.478	14.232
ct94-M74Dusty-12	120357	01-Oct-12	-2	78.475	14.268
ct94-M74Dusty-12	120357	01-Oct-12	-2	78.475	14.268
ct94-M74Dusty-12	120357	01-Oct-12	-2	78.477	14.284
ct94-M74Dusty-12	120357	01-Oct-12	-2	78.478	14.284
ct94-M74Dusty-12	120357	01-Oct-12	-2	78.475	14.314
ct94-M74Dusty-12	120357	02-Oct-12	-2	78.466	14.289
ct94-M74Dusty-12	120357	02-Oct-12	-2	78.486	14.309
ct94-M74Dusty-12	120357	02-Oct-12	-2	78.485	14.279
ct94-M74Dusty-12	120357	02-Oct-12	-2	78.475	14.264
ct94-M74Dusty-12	120357	02-Oct-12	-2	78.482	14.195
ct94-M74Dusty-12	120357	02-Oct-12	-1	78.507	14.376
ct94-M74Dusty-12	120357	02-Oct-12	-1	78.508	14.376
ct94-M74Dusty-12	120357	02-Oct-12	-2	78.477	14.222
ct94-M74Dusty-12	120357	02-Oct-12	1	78.482	14.221
ct94-M74Dusty-12	120357	02-Oct-12	1	78.479	14.205
ct94-M74Dusty-12	120357	02-Oct-12	2	78.475	14.282
ct94-M74Dusty-12	120357	02-Oct-12	2	78.48	14.307
ct94-M74Dusty-12	120357	03-Oct-12	-2	78.471	14.239
ct94-M74Dusty-12	120357	03-Oct-12	3	78.474	14.306
ct94-M74Dusty-12	120357	03-Oct-12	-2	78.491	14.133
ct94-M74Dusty-12	120357	03-Oct-12	-2	78.511	14.173
ct94-M74Dusty-12	120357	03-Oct-12	-2	78.5	14.223
ct94-M74Dusty-12	120357	03-Oct-12	-2	78.5	14.224
ct94-M74Dusty-12	120357	03-Oct-12	-2	78.502	14.203
ct94-M74Dusty-12	120357	03-Oct-12	-2	78.493	14.247
ct94-M74Dusty-12	120357	04-Oct-12	-2	78.488	14.359
ct94-M74Dusty-12	120357	04-Oct-12	-2	78.497	14.31
ct94-M74Dusty-12	120357	04-Oct-12	-2	78.496	14.301
ct94-M74Dusty-12	120357	04-Oct-12	-2	78.495	14.3
ct94-M74Dusty-12	120357	04-Oct-12	-2	78.487	14.323
ct94-M74Dusty-12	120357	04-Oct-12	-2	78.472	14.363
ct94-M74Dusty-12	120357	04-Oct-12	-2	78.488	14.465

ct94-M74Dusty-12	120357	04-Oct-12	-2	78.469	14.402
ct94-M74Dusty-12	120357	04-Oct-12	-2	78.473	14.432
ct94-M74Dusty-12	120357	04-Oct-12	-2	78.472	14.423
ct94-M74Dusty-12	120357	04-Oct-12	-2	78.47	14.358
ct94-M74Dusty-12	120357	04-Oct-12	-2	78.466	14.376
ct94-M74Dusty-12	120357	04-Oct-12	-2	78.472	14.375
ct94-M74Dusty-12	120357	04-Oct-12	-2	78.465	14.363
