# Supplementary material for: The Argos-CLS Kalman Filter: Error Structures and State-Space Modelling Relative to Fastloc GPS Data
Source: PLoS One. 2015 Apr 23;10(4):e0124754. doi: 10.1371/journal.pone.0124754 (PMC4408085; doi:10.1371/journal.pone.0124754)
Supplement: S1 Table — The background and implementation of each state space model is detailed in text and is explained in further detail in Johnson et al. (2008), Jonsen et al. (2005) and Sumner et al. (2009). Data collected from two focal animals tracked using GPS-CTD-SRDL instruments between 19th July 2011 and 18th April 2013 along the west coast of Svalbard. These data were used to determine the precision of modelled location estimates using the adjusted quantile outlier detection method (S5 Fig) (DOCX) [file pone.0124754.s008.docx]

**S1 Table.** Parameters describing idealised (outlier-free) mean and standard deviation (±SD of estimates) of lognormal probability distributions of Mahalanobis distances derived from location error magnitude between optimally-modelled Argos location and true (GPS) locations. The background and implementation of each state space model is detailed in text and is explained in further detail in Johnson *et al.* (2008), Jonsen *et al.* (2005) and Sumner *et al.* (2009). Data collected from two focal animals tracked using GPS-CTD-SRDL instruments between 19^th^ July 2011 and 18^th^ April 2013 along the west coast of Svalbard. These data were used to determine the precision of modelled location estimates using the adjusted quantile outlier detection method (Supplementary Figure 5)

|  |  | **bearded seal** | |  | **ringed seal** | |
| --- | --- | --- | --- | --- | --- | --- |
|  | **N** | **Mean** | **SD** | **N** | **Mean** | **SD** |
| **CRAWL** | 1321 | 0.613 (± 1.005) | 3.784 (± 1.004) | 375 | 0.670 (± 1.005) | 3.858 (± 1.003) |
| **BSAM** | 677 | 0.807 (± 1.005) | 3.551 (± 1.003) | 260 | 0.659 (± 1.006) | 3.924 (± 1.004) |
| **tripEstimation** | 1097 | 0.878 (± 1.005) | 3.538 (± 1.003) | 375 | 0.778 (± 1.005) | 3.489 (± 1.003) |
